# Revision of the genus *Megacraspedus* Zeller, 1839, a challenging taxonomic tightrope of species delimitation (Lepidoptera, Gelechiidae)

**DOI:** 10.3897/zookeys.800.26292

**Published:** 2018-11-29

**Authors:** Peter Huemer, Ole Karsholt

**Affiliations:** 1 Tiroler Landesmuseen Betriebsges.m.b.H., Naturwissenschaftliche Sammlungen, Krajnc-Str. 1, A-6060 Hall, Austria Tiroler Landesmuseen Betriebsges.m.b.H. Hall Austria; 2 Zoological Museum, Natural History Museum of Denmark, Universitetsparken 15, DK-2100 Copenhagen, Denmark Zoological Museum, Natural History Museum of Denmark Copenhagen Denmark

**Keywords:** Brachyptery, DNA barcoding, Gelechiidae, Lepidoptera, *
Megacraspedus
*, new species, Palearctic, taxonomy

## Abstract

The taxonomy of the Palearctic genus *Megacraspedus* Zeller, 1839 (Lepidoptera, Gelechiidae) is revised, based on external morphology, genitalia and DNA barcodes. An integrative taxonomic approach supports the existence of 85 species which are arranged in 24 species groups (disputed taxa from other faunal regions are discussed). Morphology of all species is described and figured in detail. For 35 species both sexes are described; for 46 species only the male sex is reported, in one species the male is unknown, whereas in three species the female adult and/or genitalia morphology could not be analysed due to lack of material.

DNA barcode sequences of the COI barcode fragment with > 500 bp were obtained from 264 specimens representing 62 species or about three-quarters of the species. Species delimitation is particularly difficult in a few widely distributed species with high and allegedly intraspecific DNA barcode divergence of nearly 14%, and with up to 23 BINs in a single species. Deep intraspecific or geographical splits in DNA barcode are frequently not supported by morphology, thus indicating a complex phylogeographic history or other unresolved molecular problems.

The following 44 new species (22 of them from Europe) are described: *Megacraspedusbengtssoni***sp. n.** (Spain), *M.junnilaineni***sp. n.** (Turkey), *M.similellus***sp. n.** (Bulgaria, Romania, Turkey), *M.golestanicus***sp. n.** (Iran), *M.tokari***sp. n.** (Croatia), *M.neli***sp. n.** (France, Italy), *M.faunierensis***sp. n.** (Italy), *M.gredosensis***sp. n.** (Spain), *M.bidentatus***sp. n.** (Spain), *M.fuscus***sp. n.** (Spain), *M.trineae***sp. n.** (Portugal, Spain), *M.skoui***sp. n.** (Spain), *M.spinophallus***sp. n.** (Spain), *M.occidentellus***sp. n.** (Portugal), *M.granadensis***sp. n.** (Spain), *M.heckfordi***sp. n.** (Spain), *M.tenuiuncus***sp. n.** (France, Spain), *M.devorator***sp. n.** (Bulgaria, Romania), *M.brachypteris***sp. n.** (Albania, Greece, Macedonia, Montenegro), *M.barcodiellus***sp. n.** (Macedonia), *M.sumpichi***sp. n.** (Spain), *M.tabelli***sp. n.** (Morocco), *M.gallicus***sp. n.** (France, Spain), *M.libycus***sp. n.** (Libya, Morocco), *M.latiuncus***sp. n.** (Kazahkstan), *M.kazakhstanicus***sp. n.** (Kazahkstan), *M.knudlarseni***sp. n.** (Spain), *M.tenuignathos***sp. n.** (Morocco), *M.glaberipalpus***sp. n.** (Morocco), *M.nupponeni***sp. n.** (Russia), *M.pototskii***sp. n.** (Kyrgyzstan), *M.feminensis***sp. n.** (Kazakhstan), *M.kirgizicus***sp. n.** (Afghanistan, Kazakhstan, Kyrgyzstan), *M.ibericus***sp. n.** (Portugal, Spain), *M.steineri***sp. n.** (Morocco), *M.gibeauxi***sp. n.** (Algeria, Tunisia), *M.multipunctellus***sp. n.** (Turkey), *M.teriolensis***sp. n.** (Croatia, Greece, Italy, Slovenia), *M.korabicus***sp. n.** (Macedonia), *M.skulei***sp. n.** (Spain), *M.longivalvellus***sp. n.** (Morocco), *M.peslieri***sp. n.** (France, Spain), *M.pacificus***sp. n.** (Afghanistan), and *M.armatophallus***sp. n.** (Afghanistan). *Nevadia* Caradja, 1920, **syn. n.** (homonym), *Cauloecista* Dumont, 1928, **syn. n.**, *Reichardtiella* Filipjev, 1931, **syn. n.**, and *Vadenia* Caradja, 1933, **syn. n.** are treated as junior synonyms of *Megacraspedus*. Furthermore the following species are synonymised: *M.subdolellus* Staudinger, 1859, **syn. n**., *M.tutti* Walsingham, 1897, **syn. n**., and *M.grossisquammellus* Chrétien, 1925, **syn. n**. of *M.lanceolellus* (Zeller, 1850); *M.culminicola* Le Cerf, 1932, **syn. n.** of *M.homochroa* Le Cerf, 1932; *M.separatellus* (Fischer von Röslerstamm, 1843), **syn. n.** and *M.incertellus* Rebel, 1930, **syn. n.** of *M.dolosellus* (Zeller, 1839); *M.mareotidellus* Turati, 1924, **syn. n.** of *M.numidellus* (Chrétien, 1915); *M.litovalvellus* Junnilainen, 2010, **syn. n.** of *M.imparellus* (Fischer von Röslerstamm, 1843); *M.kaszabianus* Povolný, 1982, **syn. n.** of *M.leuca* (Filipjev, 1929); *M.chretienella* (Dumont, 1928), **syn. n.**, *M.halfella* (Dumont, 1928), **syn. n.**, and *M.arnaldi* (Turati & Krüger, 1936), **syn. n.** of *M.violacellum* (Chrétien, 1915); *M.escalerellus* Schmidt, 1941, **syn. n.** of *M.squalida* Meyrick, 1926. *Megacraspedusribbeella* (Caradja, 1920), **comb. n.**, *M.numidellus* (Chrétien, 1915), **comb. n.**, *M.albella* (Amsel, 1935), **comb. n.**, *M.violacellum* (Chrétien, 1915), **comb. n.**, and *M.grisea* (Filipjev, 1931), **comb. n.** are newly combined in *Megacraspedus*.

## Introduction

The Gelechiidae are a mega-diverse family of Lepidoptera with approximately 4,700 described species (van Nieukerken et al. 2011) on a global scale, but the vast number of still undescribed species is estimated to double this preliminary count (Karsholt et al. 2013). One of the major problems in Gelechiidae taxonomy is the general lack of experts, which may be related to lack of interest due to the small size and the frequently inconspicuous colour and wing pattern of numerous species, but also to the general difficulties in discovery and recognition of species and the lack of decent phylogenetic studies (Kaila et al. 2011). Even in the generally well explored Palearctic Lepidoptera fauna, only relatively few generic revisions (e.g., Bidzilya 2005a, 2005b, Bidzilya and Karsholt 2015, Englert 1975, Huemer 1988, Karsholt and Šumpich 2015, Karsholt and Rutten 2005, Pitkin 1984, 1988, Sattler 1976, 1979) or monographic reviews of larger taxonomic entities up to family level (e.g., Huemer and Karsholt 1999, 2010, Ponomarenko 1997, Povolný 2002) have been published in the past, and identification of Gelechiidae even today is a critical task in many unrevised genera.

*Megacraspedus* Zeller, 1839 is one of these widely neglected genera of Palearctic Gelechiidae, unknown to the vast majority of lepidopterists. Among experts in Gelechiidae it is considered as one of the taxonomically most difficult genera and few have dealt with it in the last 100 years, usually with isolated descriptions of single species (e.g., Bidzilya and Budashkin 2015, Huemer and Karsholt 1996, 2001, Junnilainen and Nupponen 2010, Varenne and Nel 2014). The reasons for this neglect are mainly due to the absence of a generic revision, the general lack of material in collections, with several species only present in the small type-series, the absence of the female sex for the majority of species, and the general problems in the delimitation of species on adult morphology.

The necessity of a monographic review became increasingly relevant when the authors of this study, in co-operation with Jan Šumpich, decided to publish an additional, third volume of the series “Microlepidoptera of Europe” (see Huemer and Karsholt 1999, 2010 for earlier volumes) dealing with the Gelechiidae/Anomologinae and including *Megacraspedus*. It was soon realized that a limited revision of the European fauna would be imperfect, as several species described from North Africa, the Near and Middle East as well as from Central Asia showed relevance to or even conspecificity with European counterparts. Therefore the geographic area was expanded to the Palearctic region. Organization of sufficient vouchers and type material proved particularly challenging for our revisionary plans but many primary types were studied for the first time since their initial description. In contrast to most of the foregoing revisions of European/Palearctic Gelechiidae we took advantage of molecular methods, in particular DNA barcoding, combined with extensive studies of external and internal adult morphology (Huemer and Hebert 2011, Huemer et al. 2014). This resulted in the discovery of an unexpected layer of hitherto undescribed diversity in *Megacraspedus*, encompassing approximately half of the presently described fauna. However, despite all efforts, we failed to fully resolve conflicting results in molecular data and morphology for several species and more cryptic diversity cannot be excluded.

## Materials and methods

### Specimens

Our study is based on a large amount of material of *Megacraspedus* from various institutional and private collections (see below). Most of the material was traditionally pinned, set and dried or alternatively spread; few specimens are only pinned. Genitalia preparations followed standard techniques (Robinson 1976) adapted for male genitalia of Gelechiidae by the so-called “unrolling technique” (Pitkin 1986); some older slides remained traditionally mounted or were carefully remounted in exceptional cases. Wing preparation followed techniques described by Belkin and McDonald (1952) with small modifications according to Landry et al. (2017). Discoloured wings were stained in a solution of Acid Fuchsin in 30% ethanol for 48 hours. Subsequent cleaning from scales and fringes was undertaken in 30% ethanol. Measurements of genitalia structures were taken with a micrometer eyepiece. Data from holotypes are cited exactly as on the labels of the specimens, whereas other material is organised in a standardized format rather than verbatim, viz., alphabetic after country and province (region, oblast etc.); material from the same province is listed chronologically.

### DNA Barcoding

Lepidopteran DNA barcode sequences are based on a 658 base-pair long segment of the 5’ terminus of the mitochondrial COI gene (cytochrome c oxidase 1). DNA samples (dried legs) were prepared according to the prescribed standards. Legs from 418 specimens of *Megacraspedus* were processed at the Canadian Centre for DNA Barcoding (CCDB, Biodiversity Institute of Ontario, University of Guelph) to obtain DNA barcodes using the standard high-throughput protocol described in deWaard et al. (2008). The vast majority of material (368 specimens), collated from various institutional and private collections, was submitted for processing by Tiroler Landesmuseum Ferdinandeum, Innsbruck (Austria), further specimens by the University of Oulu (Finland) (44 specimens), Landesmuseum Kärnten (Klagenfurt, Austria) (5 specimens), and Zoologische Staatssammlung (Munich, Germany) (1 specimen). DNA sequencing resulted in 279 sequences, with 264 sequences > 500 bp. 249 sequences were barcode compliant, including a minimum sequence length of 500 bp, fewer than 1% ambiguous bases, the presence of two trace files, a minimum of low trace quality status, and the presence of a country specification in the record as set out by the Consortium for DNA Barcoding (CBOL). Usually only such sequences were considered for analysis, only in rare exceptions where there was a lack of high quality sequences were shorter sequences analysed. For several species successful sequencing failed, likely because of degraded DNA whereas for few species no suitable specimens were available. Sequences were submitted to GenBank; further details including complete voucher data and images can be accessed in the public dataset “Lepidoptera of the Palearctic – Gelechiidae/*Megacraspedus*” https://doi.org/10.5883/DS-LEPIMEGA in the Barcode of Life Data Systems (BOLD; Ratnasingham and Hebert 2007). Degrees of intra- and interspecific variation of DNA barcodes were calculated under Kimura 2-parameter model of nucleotide substitution (Kimura 1980) using analytical tools of BOLD systems v. 3.0 (http:// www.boldsystems.org). Neighbour-Joining analysis was conducted in MEGA7 (Kumar et al. 2016). Additional nuclear sequencing (CAD and MDH genes) organized by Marko Mutanen (University of Oulu) failed for many samples and the results for a limited number of voucher specimens will be published separately.

### Photographic documentation

Photographs of the adults were taken with an Olympus SZX 10 binocular microscope and an Olympus E-3 digital camera over a dark grey background and developed using the software Helicon Focus 4.3 and Adobe Photoshop CS4 and Lightroom 2.3. Genitalia photographs were taken with an Olympus E1 Digital Camera from an Olympus BH2 microscope. Multiple photos of different focal planes were assembled into single deep-focus images both for adult specimens and genitalia. Photographs from external sources are used for few specimens that we were unable to study ourselves. Photographs of wing slides were taken with a Canon 750D Digital camera and lens Canon MP-E 65 mm. Multiple photos of different focal planes were stacked into single deep-focus images, assembled with Helicon Focus 6 and Adobe Photoshop CC 2017. Adults, as well as male and female genitalia are usually figured to the same scale.

### Terminology

The descriptive terminology of the genitalia structures generally follows Kristensen (2003).

### Formal synonymisations

Some unpublished taxonomic changes made by Linda Pitkin and Klaus Sattler (both at BMNH) have been made available on the internet by Beccaloni et al. (2005), and from there they have been copied into numerous webpages. These new synonyms and combinations have not yet been formalised in print. Based on the advice of Thomas Pape, president of the International Commission of Zoological Nomenclature, we are taking the opportunity to do this here.

### Gender agreement

Gender agreement between specific and generic names as prescribed by article 31.2 of the ICZN (1999) is not followed in accordance with the widely accepted proposals by Sommerer (2002).

**Abbreviations of private and institutional collections**

**BFUS** Zoological collection, Faculty of Biology, Sofia University, Bulgaria

**BMNH**The Natural History Museum, London, U.K. (formerly British Museum of Natural History)

**ECKU** Collection of Ecology-Centre, Kiel University, Germany

**HNHM** Hungarian Natural History Museum, Budapest, Hungary

**LMK** Landesmuseum Kärnten, Klagenfurt, Austria

**MGAB** National Museum of National History “Grigore Antipa”, Bucharest, Romania

**RMNH** Naturalis Biodiversity Center, Leiden, the Netherlands

**MHNT** Muséum d´Histoire naturelle, Tolouse, France

**MNCN** Museo Nacional de Ciencias Naturales, Madrid, Spain

**MNHN** Muséum national d´Histoire naturelle, Paris, France

**MTD** Senckenberg Museum für Tierkunde, Dresden, Germany

**MZH** Finnish Museum of Natural History, Helsinki, Finland

**NHMB** Naturhistorisches Museum, Basel, Switzerland

**NHMW** Naturhistorisches Museum, Vienna, Austria

**NKU** Insect Collection of Nankai University, Tianjin, China

**NMPC** National Museum Prague, Czech Republic

**RCBB** Research Collection Bengt Åke Bengtsson, Färjestaden, Sweden

**RCAB** Research Collection Aleksei Bidzilya, Kiev, Ukraine

**RCAP** Research Collection Alexander Pototski, Tallinn, Estonia

**RCAW** Research Collection Andreas Werno, Nunkirchen, Germany

**RCCDL** Research Collection Carmello De Lucca, Malta

**RCEA** Research Collection Ernst Arenberger, Vienna, Austria

**RCER** Research Collection Emili Requena Miret, Gurb, Spain

**RCGB** Research Collection Giorgio Baldizzone, Asti, Italy

**RCGT** Research Collection Giovanni Timossi, Oderzo, Italy

**RCHW** Research Collection Hugo van der Wolf, Nuenen, The Netherlands

**RCJD** Research Collection Jordi Dantart, Barcelona, Spain

**RCJJ** Research Collection Jari Junnilainen, Vantaa, Finland

**RCJN** Research Collection Jacques Nel, La Ciotat, France

**RCJR** Reseach Collection Jorge Rosete, Louriçal, Portugal

**RCKO** Research Collection Sándor Kovács & Zoltán Kovács, Miercurea Ciuc, Romania

**RCKN** Research Collection Kari Nupponen, Espoo, Finland

**RCMC** Research Collection Martin Corley, Faringdon, U.K.

**RCPF** Research Collection Per Falck, Nexø, Denmark

**RCPT** Research Collection Paolo Triberti, Verona, Italy

**RCRH** Research Collection Robert J. Heckford, Plympton, Plymouth, U.K.

**RCSP** Research Collection Serge Peslier, Perpignan, France

**RCTM** Research Collection Toni Mayr, Feldkirch, Austria

**RCTV** Research Collection Thierry Varenne, Nice, France

**RCTZ** Research Collection Tomasz Rynarzewski, Poznań, Poland

**RCZT** Research Collection Zdenko Tokár, Šal’a, Slovakia

**RCWS** Research Collection Willibald Schmitz, Bonn, Germany

**SMNK** Staatliches Museum für Naturkunde, Karlsruhe, Germany

**TLMF** Tiroler Landesmuseum Ferdinandeum, Innsbruck, Austria

**ZMHU** Zoologisches Museum, Humboldt University, Berlin, Germany

**ZMUC** Zoological Museum, Natural History Museum of Denmark, Copenhagen, Denmark

**ZSM** Zoologische Staatssammlung, Munich, Germany

## Systematic part

### The systematic position of *Megacraspedus*

The systematics of the Anomologinae on tribal and generic level is still imperfectly known, and it is outside the scope of the present study to determine the exact phylogenetic placement of *Megacraspedus*. However, there is an overall agreement about the systematic position of *Megacraspedus*. Meyrick (1925) placed the genus in his second genus group (“*Aristotelia* type”), which largely coincides with present day Anomologinae, between *Pycnostola* Meyrick, 1917, and *Isophrictis* Meyrick, 1917. This was later followed in major checklists, e.g., Hodges (1983: 19), Edwards (1996: 113), Karsholt and Riedl (1996: 104), and Ponomarenko (2008: 90). This placement has recently been supported by a molecular analysis of some genera of Gelechiidae based on eight genes (Karsholt et al. 2013: 341). According to that study *Megacraspedus* is nested (with high support) within the Anomologinae between an unresolved cluster of four genera (*Aristotelia* Hübner, 1825, *Psamathocrita* Meyrick, 1925, *Xerometra* Meyrick, 1925 and *Deltophora* Janse, 1950) and *Isophrictis* and *Metzneria* Zeller, 1839. Only one species of each genus was used in that study (for *Megacraspedus, M.dolosellus* was analysed).

Edwards (1996: 108) placed *Xerometra* in the Dichomeridinae, but the species used by Karsholt et al. (2013), *X.mesophracta* (Turner, 1919), was removed from *Xerometra* by Li and Sattler (2012: 56) and placed without a genus in the Anomologini. We examined the genitalia of a pair of *X.mesophracta* used for the above-mentioned molecular analysis and found them to be close to those of *Megacraspedus*.

### Generic descriptions

***Megacraspedus* Zeller, 1839**

*Megacraspedus* Zeller, 1839: 189.

Type species: Ypsolophus (Megacraspedus) dolosellus Zeller, 1839. Designated by Walsingham (1909: 21).

*Chilopselaphus* Mann, 1867: 850.

Type species: *Chilopselaphusfallax* Mann, 1867. By monotypy.

*Chilopselaphalus* Rebel, 1901: 161 (incorrect subsequent spelling).

*Toxoceras* Chrétien, 1915: 329, fig. 5 (homonym). By monotypy.

Type species: *Toxocerasviolacellum* Chrétien, 1915.

*Toxidoceras* Chrétien, 1923: 168 (objective replacement name of *Toxoceras* Chrétien).

Type species: *Toxocerasviolacellum* Chrétien, 1915.

*Nevadia* Caradja, 1920: 117 (homonym) syn. n. By monotypy.

*Cauloecista* Dumont, 1928: 33 syn. n.

Type species: *Cauloecistachretienella* Dumont, 1928. By original designation.

*Reichardtiella* Filipjev, 1931: 167 syn. n.

Type species: *Reichardtiellagrisea* Filipjev, 1931. By monotypy.

*Vadenia* Caradja, 1933: 94 syn. n.

Type species: *Nevadiaribbeella* Caradja, 1920 (objective replacement name of *Nevadia* Caradja).

### Remarks on genus group names

*Megacraspedus* was described as a subgenus of the genus *Ypsolophus* Fabricius, 1798 by Zeller and included two species: “*striatellus* S.V.” [= *Isophrictisstriatella* ([Denis and Schiffermüller], 1775)] and “*dolosellus* FR.” [= *Megacraspedusdolosellus* (Zeller, 1839)]. The latter was subsequently designated as the type species of *Megacraspedus* (Walsingham 1909: 21).

*Chilopselaphus* was described by Mann for his new species *C.fallax*. Among the several details stated to be peculiar to *Chilopselaphus* were the long labial palps, whereas the venation of the hindwings was stated to be similar to that of *Megacraspedus* (Mann 1867: 849–850). *Chilopselaphus* was synonymised with *Megacraspedus* by Junnilainen and Nupponen (2010: 6), and we agree with that (see below under Remarks). Most species formerly placed in *Chilopselaphus* are here referred to the *M.fallax* species group.

*Toxoceras* was described by Chrétien to include a single species *T.violacellum* from North Africa. It was compared with the genera *Paltodora* Meyrick, 1894 and *Ypsolophus*, but not with *Megacraspedus*. It was listed as a synonym of the latter by Meyrick (1925: 33). Together with *M.ibericus* sp. n., *M.violacellum* forms our *M.violacellum* species group. *Toxoceras* Chrétien is a homonym of *Toxoceras* d’Orbigny, 1842 (Mollusca). The objective replacement name is *Toxidoceras* (Nye and Fletcher 1991: 311).

*Nevadia* was described by Caradja to include a single species *N.ribbeella*. It was compared with *Megacraspedus* and *Chilopselaphus*, but stated to differ in the shape of the forewing and its cell. According to its genitalia it is nested within the *M.fallax* species group. *Nevadia* Caradja is a homonym of *Nevadia* Walcott, 1910 (Trilobita). The objective replacement name is *Vadenia*Caradja (1933: 94) (Sattler 1973: 228).

*Cauloecista* was described by Dumont to include *C.chretienella* (genus type) and *C.halfella* from Tunisia. Our studies have shown that both are synonyms of *M.violacellum* (see above under *Toxoceras*). See also the section on Formal synonymisations.

*Reichardtiella* was described by Filipjev to include a single species *R.grisea*. It was compared with *Megacraspedus* and *Pycnostola*, but stated to differ in details of wing venation. Together with *M.pacificus* sp. n. and *M.armatophallus* sp. n., *M.grisea* forms our *M.grisea* species group. Meyrick (1926) described two species (*M.neurophanes* and *M.niphorrhoa*) in the genus *Trichembola* Meyrick, 1918. However, the type species of *Trichembola* has no uncus in the male genitalia (K Sattler in litt.) and is thus not a synonym of *Megacraspedus*.

*Neda* Chambers, 1874, with the type species *Nedaplutella* Chambers, 1874 from North America, is a homonym of *Neda* Mulsant, 1850 (Coleoptera) which was later replaced by *Autoneda* Busck, 1903 (Sattler 1973: 175). Material externally matching the type species, dissected and photographed by Sangmi Lee (in litt.), probably belongs to the genus *Monochroa* Heinemann, 1870. Though the correct status needs further revisionary work, we exclude *Autoneda* as congeneric with *Megacraspedus*.

### Descriptions

**Figure 1.**
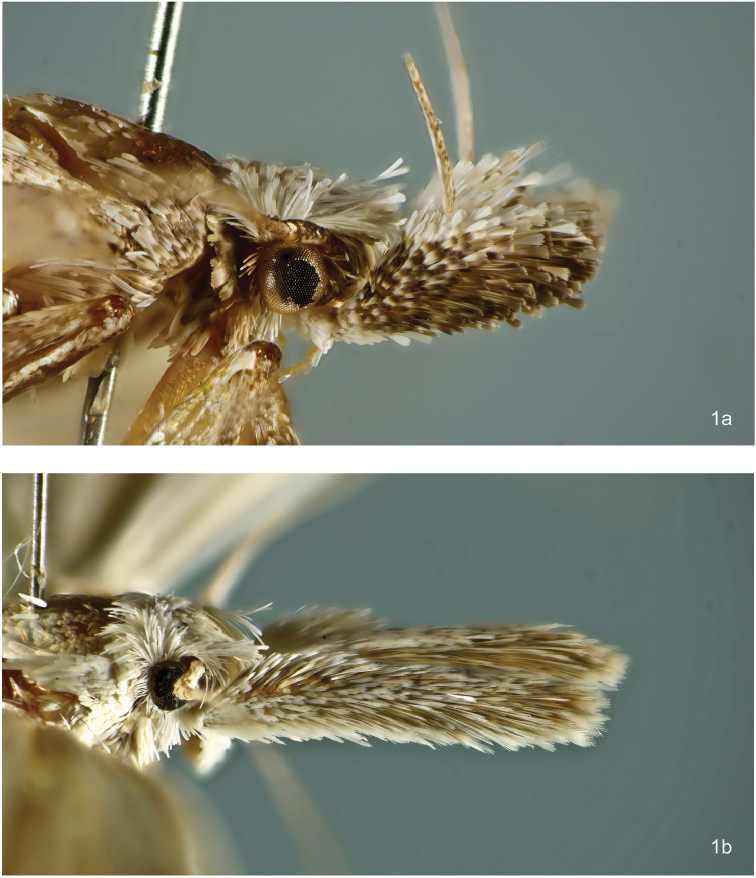
Labial palpus of *Megacraspedus*. **a***M.squalida* Meyrick, 1926 **b***M.longipalpella* Junnilainen, 2010.

**Figure 2.**
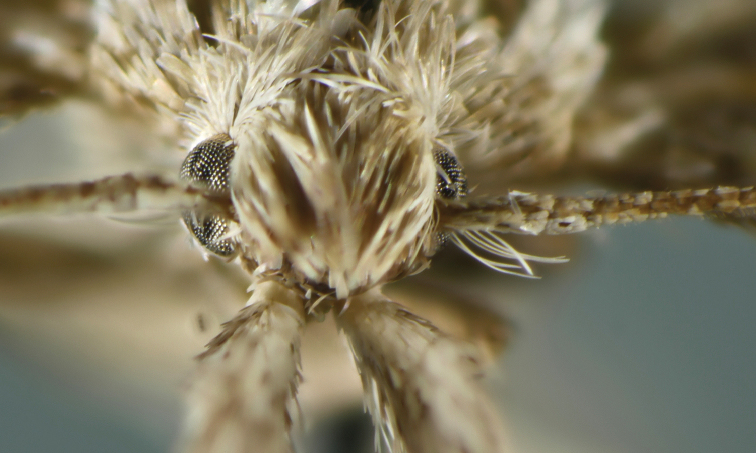
Adult head with pecten on antennal scape of *Megacraspedusniphorrhoa* (Meyrick, 1926).

*Adults.* Small to large gelechiids (wingspan 8–26 mm). Segment 2 of labial palpus with brush of long scales, these being most often light at base and becoming darker towards tip, especially on lower and outer surface; segment 3 as long as or shorter than segment 2, sometimes largely reduced, slender, either pointed upwards or porrect and more or less hidden in the scales of segment 2 (Figure 1a–b). Antennal scape in several species with pecten consisting of one to about ten deciduous hair-like scales (Figure 2); flagellum filiform or with fine short hairs, either unicolorous or more or less distinctly ringed lighter. Head often light, especially towards face; thorax and tegula normally of forewing colour, rarely darker. Forewing lanceolate with more or less pointed apex; from whitish to dark grey, often lighter along costa and in fold, with no or several black dots in fold and middle of wing, veins sometimes with white scales (rarely with dark scales), apical part of wing often darker than basal part (apart from basal costa). Hindwing of about same width as forewing or slightly broader, with clear sinuation of outer margin and more or less pointed apex, from whitish grey to dark grey. Underside greyish, showing little diagnostic characters. Female either similar to male or with different degree of wing reduction.

**Figure 3.**
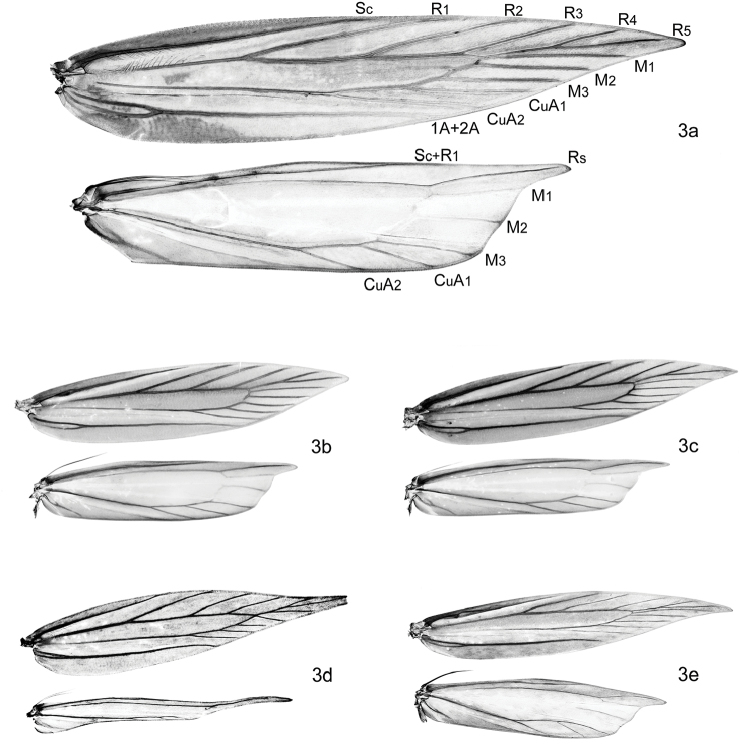
Wing venation of *Megacraspedus*. **a***M.lagopellus* Herrich-Schäffer, 1860 – male, Armenia, (NMPC), forewing 8.5 mm **b***M.peyerimhoffi* Le Cerf, 1925 – male, Spain (NMPC), forewing 9 mm **c***M.peyerimhoffi* Le Cerf, 1925 – female, Spain (NMPC), forewing 10 mm **d***M.binotella* (Duponchel, 1843) – female, Slovakia (NMPC), forewing 5.5 mm **e***M.fallax* (Mann, 1867) – male, Russia (NMPC), forewing 8.5 mm.

*Wing venation* (Figure 3a–e). Forewing narrow to broadly elongate, venation variable; costa without pterostigma between Sc and R_1_; R_3_ free; common stalk of R_4_+R_5_ shorter or as long as free R_4_+R_5_; M_1_ approximated to, connate or stalked with R_4_+R_5_; M_2_, M_3_, CuA_1_, and CuA_2_ separate. Shape of hindwing highly variable, depending on reduction of wing; termen concave beneath sharply produced apex, in brachypterous species from lanceolate to rounded, more or less without emargination below apex. R_1_+Sc close to upper margin; Rs, M_1_, M_2_ and M_3_ free or M_1_ and M_2_ reduced; M_3_ from lower angel of cell, separate from Cu_1_; border of cell between M_1_ and M_3_ ill defined. Frenulum of female composed of two acanthae.

*Male pregenital segments*. Sternite and tergite VIII without distinct modifications.

**Figure 4.**
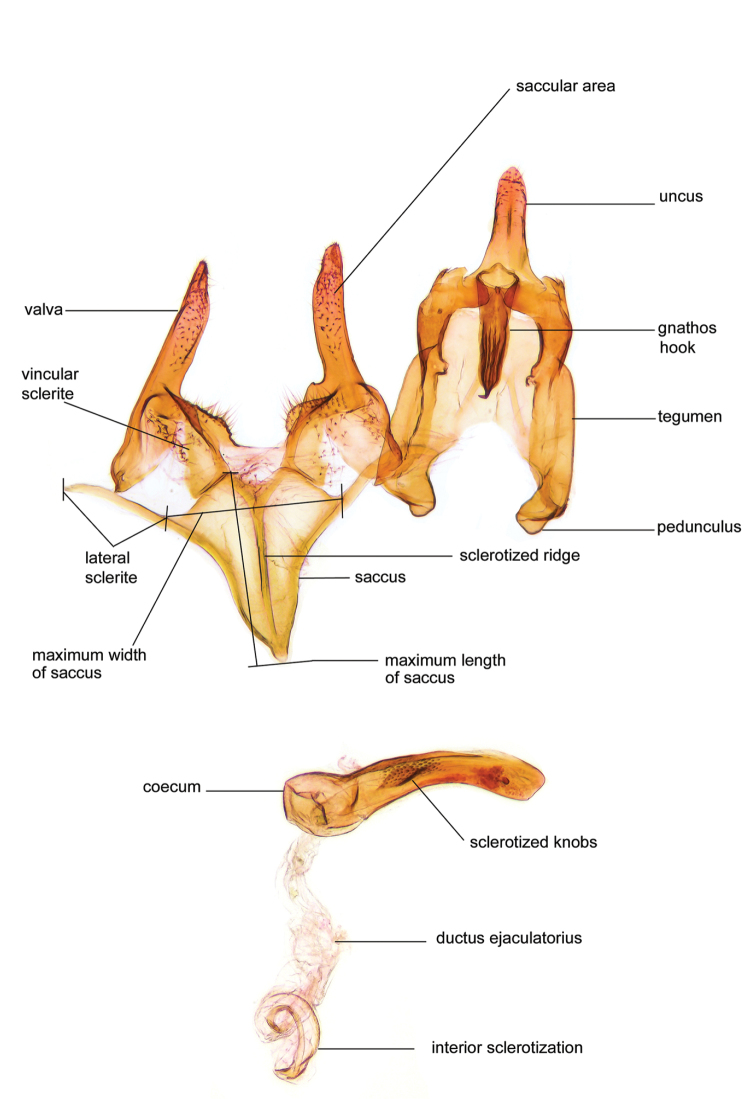
Terminology of important characters in male genitalia of *Megacraspedusgranadensis* sp. n.

*Male genitalia* (Figure 4). Uncus well developed, of variable shape, usually suboval to sub-rectangular, rarely short and slender trapezoidal (*M.lagopellu*s species group) or otherwise modified; gnathos prominent, composed of rhombic base and strongly sclerotised, straight to strongly bent or curved large gnathos hook, articulated at lateral teguminal sclerites, length of gnathos hook usually equalling length of uncus; tegumen largely without specialised structures, lateral shoulders on the level of gnathos articulation, anteriomedial part with or without sclerotised rods, anterior margin moderately to deeply emarginated, anteromedially frequently with additional excavation; pedunculi small, drop-shaped to large suboval; valva moderately slender to broadly digitate, straight to weakly curved, usually shorter than tip of uncus, exceptionally extending beyond uncus, large area of sensory setae on inner side, with or without separated sacculus; sacculus occasionally developed, short, flap-like to digitate; vinculum with largely membranous posterior part, densely covered with sensory setae, particularly on indistinct to well-developed lateral humps, medially weakly to distinctly emarginated, variably shaped vincular sclerites extending from sclerotised posterior edge of saccus to sub-basal part of valva, posteriomedial edge frequently heavily sclerotised and connected with valva; saccus large and massive, distally tapered, posterior margin arched, frequently with distinct medial emargination resulting in lateral humps, medial part of saccus smooth, with or without longitudinal sclerotised ridge, laterally with moderately short to very long rod-like sclerites; phallus usually about length of tegumen, moderately slender to broad and stout, straight to weakly curved, coecum bulbous, longer distal part norrower, frequently with sclerotised ridges, occasionally with teeth-like sclerites of various shape and size, ductus ejucalatorius with or without interior sclerotisation.

*Female pregenital segments*. Sternite and tergite VII without distinct modifications, except for *M.monolorellus* with strongly elongated sternite and tergite VII.

**Figure 5.**
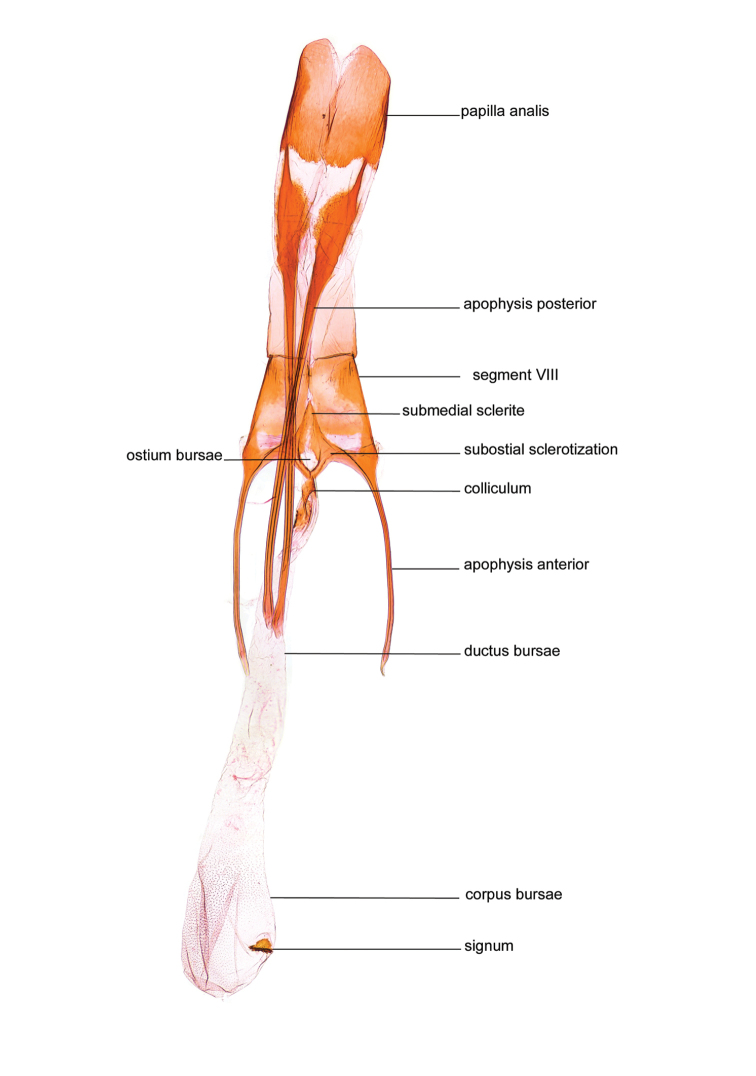
Terminology of important characters in female genitalia of *Megacraspedusniphorrhoa* (Meyrick, 1926).

*Female genitalia* (Figure 5). Papilla analis weakly to strongly sclerotised, of various size and shape, small to very large, usually elongated and apically rounded, in the *M.lagopellus* species group with dorsally pointed apex, sparsely to strongly setose; apophysis posterior usually rod-like, rarely with distinct widening, moderately short to very long, between 1.5 to 6 times length of papilla analis; segment VIII simple, largely membranous to smoothly sclerotised, particularly in posterior and lateral part, subgenital plate with sub-triangular to weakly curved subostial sclerotisation, laterally with short to long and pointed sclerites, delimiting ostium bursae, subostial sclerotisations rarely reduced, dorso-anterior edge of segment VIII sclerotised and connected with apophysis anterior; apophysis anterior slender, rod-like, about length of segment VIII, posteriorly frequently turned to rod-like venula of segment VIII, venula sometimes with widened sclerotised posterior base; short colliculum usually present, rarely sclerotised antrum developed, extending at most to anterior third of apophysis anterior; ductus bursae gradually widening to weakly separated corpus bursae; signum a small to medium-sized and usually spiny plate, occasionally much reduced in size.

### Biology

The early stages of most *Megacraspedus* species are unknown. Descriptions of larvae have only been published for three species (*M.dolosellus*, *M.peyerimhoffi*, and *M.violacellum*), although they are very detailed (and all dating back to the first part of the 20^th^ century). There are host plant records for a few further species. All confirmed host plants belong in the Poaceae. The larvae live within the rhizomes or subterranean stem of ‘soft’ grasses like *Elymusrepens* (L.) Gould, *Poaannua* L. or *Loliumperenne* L. (*M.dolosellus*), or in the lower stem of the more ‘stiff’ grass *Macrochloatenacissima* (Loefl. ex L.) (*M.peyerimhoffi* and *M.violacellum*). The larva of *M.dolosellus* has been reported as harmful to wheat (*Triticumaestivum* L.) (Joannis 1923, Trouvelot 1923).

*Megacraspedus* species occur at altitudes ranging from sea level to high mountains (2500 m in the Alps, Sierra Nevada, and Turkey, 2700 m in the Balkans, 3200 m in the Atlas Mountains, and up to 4300 m in the Pamir Mountains of Central Asia (*M.grisea*). The habitats are different kind of grassland (e.g., halophytic meadows, grassy steppes, or alpine grassland). Adults have been collected in all months from February to November, but there is no evidence that any species have more than one annual generation. Most species are active during the night and are attracted to artificial lights. Several species seem to be active also in the early morning, where females are reported to run up and down grass stems, apparently calling for males, and they can occasionally been found in copula on the stems (Huemer pers. obs., Junnilainen and Nupponen 2010).

### Distribution

The genus *Megacraspedus* is widely distributed throughout the south-western part of the Palearctic region (Supplementary material 1). The northern distribution limit for *Megacraspedus* in Europe is around the 50^th^ degree of latitude. Southwards, several species are known from North Africa but the genus is almost entirely absent from the larger Mediterranean islands. In Asia several species occur in the Middle East, whereas only a handful reach further to the east, to central Asia and north-western China, Mongolia, and Siberia. We examined a single specimen from the Himalaya mountain range which is tentatively identified as *M.dolosellus*. No species of *Megacraspedus* is known so far from East Asia or from the Oriental region.

With 25 species, Spain has the most diverse fauna of *Megacraspedus*, followed by Russia (14 species) and France (12 species). No *Megacraspedus* species were found from Switzerland, although several species occur in the adjacent parts of France and Italy, and the genus is not listed in the checklist of Swiss Lepidoptera (SwissLepTeam 2010). Of the 85 known *Megacraspedus* species, 42 species have only been found in a single country or a larger island, and only two species are widespread: *M.dolosellus* from Italy to Central Asia and *M.lanceolellus* from the Balkan Peninsula to southern Spain. This distribution pattern, and the limited distribution of many species, is most likely due to the brachyptery of females, which in numerous species are more or less unable to fly. Only a few species of *Megacraspedus*, if any at all, seem to occur outside of the Palearctic region. All these taxa need further revisionary work (see section Taxa misplaced in *Megacraspedus*).

The present revision results in many changes and new country records, e.g., the most recent checklist of the Lepidoptera of Spain (Vives Moreno 2014: 171) lists 15 species in *Megacraspedus* and one in *Vadenia*, but of these four are synonyms of other species in the list and two are misidentifications; thus, the real number of Spanish *Megacraspedus* is more than doubled. We do not go into details about new country records.

### Remarks

*Pecten*. Several species of *Megacraspedus* have a pecten at the base of the antennal scape. This may consist of one, or from two to about ten hair-like scales. The number of such scales seems to be consistent within each species. In worn specimens (obtained e.g., by sweeping or from automatic light traps) one or rarely both pectens may be lost. A pecten usually consists of a row of long erect scales. It is found in most families of the Gelechioidea and may be a groundplan character of this superfamily. It is rare within the Gelechiidae where it is known from members of the Apatetrinae and Anomologinae. In the former subfamily the pecten consists of a row of long erect scales, and is present in both the Apatetrini and Pexicopini. In the Anomologinae a pecten is present in several genera, either in all species within a genus (e.g., *Bryotropha* Heinemann, 1870 where it is a single, long and strong, narrow scale) or only in single species of, e.g.,, *Aristotelia* Hübner, 1825 or *Monochroa* Heinemann, 1870 where it is a more deciduous hair-like scale (Sattler 1979: 266). *Psamathocrita* Meyrick, 1925 species have a well-developed pecten consisting of several hair-like scales similar to that found in some species of *Megacraspedus*. Just as in the larvae of a few *Megacraspedus* species with described larval behaviour, the larva of at least one *Psamathocrita* species (*P.argentella* Pierce & Metcalfe, 1942) also feeds on grasses (Poaceae), indicating a possible relationship between these two genera. The function of the pecten is unknown, but one can imagine that it can be used for protecting or cleaning the eyes. In *Megacraspedus* a pecten is found in a number of species occurring in steppe or desert biotopes, whereas most species occurring in mountains in Europe are without an antennal pecten.

*Labial palpus*. Two different types of labial palpi are present in *Megacraspedus* (Figure 1a, b). In most species groups segment 2 has a sub-triangular forward-directed brush (which is often longer than segment 3), and segment 3 is recurved. In a few groups (the *M.fallax* species group and the *M.longivalvellus* species group) the labial palpi are porrect, viz., the short segment 3 is not turned upwards, but is pointed forward at the end of the (often very long) segment 2. In the latter case segment 3 may be very short and hidden in the scale brush of segment 2. Exceptionally (*M.glaberipalpus* sp. n.) the scale brush at segment 2 of the labial palpus is reduced. The labial palpus is a characteristic feature of gelechiid moths (hence the German name *palpenmotten* for this family), and in former days the shape of the labial palpi was considered an important character in the systematics of the Gelechiidae. Today the classification of the Gelechiidae is mainly based on structures of the genitalia and on molecular data, and it has been shown that species with different shapes of the labial palpi may still be closely related (e.g., Li and Sattler 2012: 8). *Megacraspedus* species with porrect labial palpi were placed in separate genera for a long time (*Chilopselaphus* and *Vadenia*), but according to their genitalia and DNA barcode sequences they are nested within *Megacraspedus*. Porrect labial palpi are mostly present in larger species of *Megacraspedus*, and they thereby (together with the colour and markings of their forewings) have some similarity to species of Crambinae, and with these they share grasslands as habitat. The advantage of having porrect labial palpi as opposed to more recurved ones when living in grasslands is unknown.

*Wing venation*. The overall rather uniform wing venation within Gelechiidae was in former days used as an important character for classifying the Gelechiidae (e.g., Meyrick 1925). More modern classifications of Gelechiidae are primarily based on the structures of the genitalia, and more recently on studies of DNA. Although some traits from the wing venation may be useful for studying taxonomy of this family, this is hampered because the venation cannot easily be examined. It is even more problematic that the venation is variable, within the same genus (as in *Megacraspedus*), but also between specimens of the same species and even within right and left wings of the same specimen (e.g., Sattler 1979: 271, Landry et al. 2017: 460). Wing venation representing several genera of Anomologinae was recently figured by Nel and Varenne (2013: 38), showing some diversity in the venation between genera, but such figures should only be used very carefully for identification purpose.

*Wing reduction*. Females of many *Megacraspedus* species are brachypterous. According to Sattler (1991: 245) the term is used for “species showing various degrees of wing reduction”. In female *Megacraspedus* especially the hindwings of a number of species become more slender without distinct emargination of termen and more or less distinctly shorter. One or more veins are lost, and the fringe is reduced. The forewings of such species are less reduced, but tend to become more lanceolate with a pointed apex. There is no clear distinction between fully winged and more brachypterous species, and in females of several species the wings are only slightly reduced. Sattler (1991: 268) estimated that wing reduction could be observed in about one-third of the Palearctic *Megacraspedus* then known. We could only examine the female genitalia of 36 of the 86 species dealt with here, and females of three additional species were not available for dissection. About half of these have more or less reduced wings, but it is likely that a larger number of the unknown females are brachypterous, because flightless females are much more difficult to collect than males, who are attracted to artificial lights. In Lepidoptera wing reduction is rare, being known from less than 1% of the described species (Sattler and Wojtusiak 2000: 435), and *Megacraspedus* is among the genera with most brachypterous species.

Brachyptery in Lepidoptera is not a uniform phenomenon but has various reasons which should be looked at on their own merit (Sattler 1991: 244). It is not clear which factors contribute to the development of wing reduction in *Megacraspedus* females. It is found among most species groups within the genus, and in species living both in lowlands and in the mountains. The known larvae of *Megacraspedus* species are associated with grasses, and Sattler (1991: 248) comments that a number of species with brachypterous females inhabit grassland, which constitutes a permanent, continuous habitat.

Female wing reduction in Lepidoptera is rarely polymorphic. One classical example is the aquatic moth *Acentriaephemerella* (Denis & Schiffermüller, 1775) (Crambidae), which has both winged and wingless females. Heppner (1991: 129) gives a few examples of polymorphic brachyptery e.g., in different generations of the same species or in restricted geographical areas. It is likely that such kind of polymorphism is underestimated due to a lack of series of brachypterous females. One further example is a recently described brachypterous *Ephysteris* Meyrick, 1908 from Asia which has some variation in the length and shape of the hindwing in the females (Bidzilya and Karsholt 2018). Wing shape polymorphy occurs also in the tortricid species *Apheliaviburnana* ([Denis and Schiffermüller], 1775) and *Zelothersespaleana* (Hübner, 1793) where females can have more or less reduced wings (O Karsholt pers. obs.).

*Female genitalia.* Female genitalia are only known for 34 of 85 species, and even for several species groups they are currently undescribed. Therefore the description of female genitalia characters on a generic level is tentative.

**Table 1.** COI sequences in *Megacraspedus*. Intraspecific mean K2P (Kimura 2 parameter) divergences, maximum pairwise distances, and distance to the nearest neighbour in percentage.

Species	Mean Intra-Sp	Max Intra-Sp	Nearest Neighbour	Nearest Species	Distance to NN
* M. albovenata *	0.93	1.39	LEAST584-17	* M. kazakhstanicus *	5.09
* M. andreneli *	4.45	4.45	PHLAH795-12	* M. tokari *	11.22
* M. argyroneurellus *	1.19	2.24	LEEUA729-12	* M. kirgizicus *	7.56
* M. attritellus *	2.08	4.19	LEEUA685-12	* M. leuca *	6.77
* M. balneariellus *	0	0	LEASS140-16	* M. podolicus *	6.28
* M. barcodiellus *	0	0	LEATI093-15	* M. binotella *	10.66
* M. bengtssoni *	0.15	0.15	PHLAF012-11	* M. lanceolellus *	11.99
* M. bidentatus *	N/A	0	PHLAH794-12	* M. cuencellus *	3.14
* M. bilineatella *	N/A	0	LEATJ1347-16	* M. fallax *	14.91
* M. binotella *	2.19	3.81	LEASS112-16	* M. devorator *	8.97
* M. brachypteris *	3.74	5.97	LEASS032-16	* M. dolosellus *	11.25
* M. cerussatellus *	N/A	0	PHLAI412-13	* M. attritellus *	7.55
* M. consortiella *	N/A	0	LEALT202-16	* M. leuca *	6.64
* M. cuencellus *	N/A	0	LEASS012-16	* M. bidentatus *	3.14
* M. devorator *	2.16	2.16	LEATI093-15	* M. binotella *	8.97
* M. dolosellus *	7.49	13.76	PHLAF014-11	* M. lanceolellus *	9.85
* M. eburnellus *	0.15	0.15	PHLAI458-13	* M. skulei *	8.46
* M. fallax *	0.1	0.16	LEAST573-17	* M. niphorrhoa *	5.56
* M. faunierensis *	1.04	1.57	LEASS022-16	* M. sumpichi *	11.88
* M. feminensis *	0.15	0.15	PHLAH788-12	* M. lagopellus *	7.77
* M. gallicus *	0.7	1.13	LEASS026-16	* M. ribbeella *	6.24
* M. glaberipalpus *	N/A	0	PHLAG308-12	* M. cerussatellus *	10.77
* M. golestanicus *	0.1	0.15	PHLAJ385-14	* M. uzunsyrtus *	8.78
* M. gredosensis *	N/A	0	LEASS012-16	* M. bidentatus *	10.01
* M. heckfordi *	0.49	0.77	LECRT129-16	* M. tenuiuncus *	7.74
* M. homochroa *	N/A	0	PHLAD606-11	* M. golestanicus *	9.1
* M. ibericus *	N/A	0	LEAST388-17	* M. violacellum *	7.46
* M. imparellus *	3.38	4.6	LEATJ1354-16	* M. attritellus *	9.15
* M. junnilaineni *	N/A	0	LECRT125-16	* M. similellus *	9.35
* M. kazakhstanicus *	N/A	0	LEEUA949-12	* M. albovenata *	5.09
* M. kirgizicus *	1.2	2.82	LEAST575-17	* M. argyroneurellus *	7.56
* M. korabicus *	0.2	0.31	LEASS618-17	* M. quadristictus *	10.13
* M. lagopellus *	0.15	0.15	LEAST583-17	* M. feminensis *	7.77
* M. lanceolellus *	7.61	12.51	LEASS101-16	* M. dolosellus *	9.85
* M. leuca *	0.03	0.19	LEAST594-17	* M. skulei *	6.57
* M. longipalpella *	N/A	0	LEAST572-17	* M. niphorrhoa *	4.78
* M. longivalvellus *	0.93	0.93	LEATJ1383-16	* M. skulei *	2.33
* M. monolorellus *	N/A	0	PHLAD606-11	* M. golestanicus *	9.17
* M. multispinella *	N/A	0	LEEUA846-12	* M. nupponeni *	8.13
* M. niphorrhoa *	0.7	1.4	PHLAI416-13	* M. longipalpella *	4.78
* M. numidellus *	N/A	0	LEFIJ2706-15	* M. gallicus *	8.97
* M. nupponeni *	N/A	0	LEEUA779-12	* M. multispinella *	8.13
* M. peyerimhoffi *	0.56	0.93	LEATJ1383-16	* M. skulei *	9.45
* M. podolicus *	N/A	0	LEASS136-16	* M. balneariellus *	6.28
* M. pototskii *	0	0	LEASS005-16	* M. ibericus *	9.14
* M. pusillus *	N/A	0	LEASS120-16	* M. spinophallus *	8.43
* M. quadristictus *	0.97	1.55	PHLAG675-12	* M. teriolensis *	9.69
* M. ribbeella *	0.15	0.15	LEFIJ2706-15	* M. gallicus *	6.24
* M. similellus *	0.79	1.19	PHLAJ385-14	* M. uzunsyrtus *	6.44
* M. skoui *	N/A	0	PHLSA052-11	* M. spinophallus *	7.97
* M. skulei *	0.32	0.64	LECRT099-16	* M. longivalvellus *	2.33
* M. spinophallus *	0.8	2.83	LASTS762-15	* M. skoui *	7.97
* M. squalida *	0.45	0.95	PHLAI458-13	* M. skulei *	9.3
* M. sumpichi *	1.71	2.5	PHLAG308-12	* M. cerussatellus *	11.41
* M. tabelli *	0.62	0.62	LEASS007-16	* M. numidellus *	9.82
* M. tenuiuncus *	N/A	0	PHLAH508-12	* M. heckfordi *	7.74
* M. teriolensis *	2.99	7.45	PHLAI454-13	* M. quadristictus *	9.69
* M. tokari *	N/A	0	PHLAH794-12	* M. cuencellus *	9.97
* M. trineae *	N/A	0	LEASS012-16	* M. bidentatus *	7.76
* M. tristictus *	N/A	0	LECRT129-16	* M. tenuiuncus *	11.98
* M. uzunsyrtus *	0	0	LEASS108-16	* M. similellus *	6.44
* M. violacellum *	N/A	0	LEASS005-16	* M. ibericus *	7.46

### Molecular results

**Figure 6.**
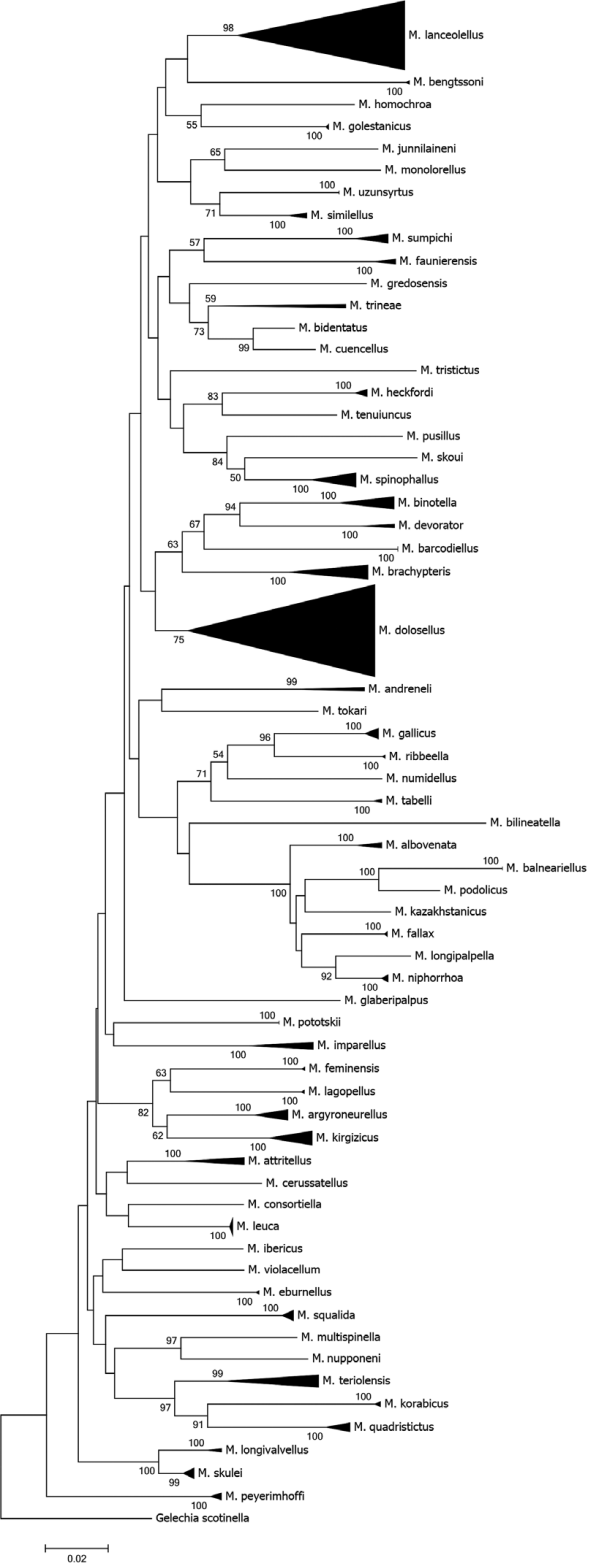
Maximum likelihood tree (built with MEGA7) of cytochrome *c* oxidase subunit I (COI) barcode fragments. Values on branches represent bootstrap values of ≥ 50 % inferred from 500 replicates, scale bar represents substitutions per site. Note: the scale bar only applies to internal branches between species. The width of the triangles represents the sample size, and the depth the relative genetic variation within the cluster (2× scale bar). Source: DNA Barcode data from BOLD (Barcode of Life Database, cf. Ratnasingham and Hebert 2007).

279 specimens of *Megacraspedus* were successfully sequenced, with 264 COI sequences longer than 500 bp. The sequenced 62 species group in different clusters (Figure 6). Maximum intraspecific distances are less than 2% in 49 (for 20 of these species only a single barcode is available) whereas it reaches 12.5% in *M.lanceolellus* and 13.8% in *M.dolosellus*. Minimum distances to the nearest neighbour range from 2.3% to 14.9% (Tab. 1). All species are separated in their BINs (Barcode Index Number) in BOLD (Ratnasingham and Hebert 2013), but ten species have more than one BIN, reflecting considerable intraspecific divergence or eventually cryptic diversity.

## Checklist of Palearctic *Megacraspedus*

*Species groups.* Closely related species are treated within informal species groups. These are mainly based on male genitalia characters (females are frequently unknown), supported by molecular data of the DNA barcode (available for the majority of species), and supplemented by external features of the adults. Several of these groups, particularly those without molecular support, may not be monophyletic and similarities could occasionally reflect convergences only. Except for a few widely distributed species, the vast majority of species groups are geographically restricted, indicating a common evolutionary scenario of species. Junnilainen and Nupponen (2010) have already dealt with some species groups of *Megacraspedus*: the *attritellus*-group: “the gnathos is 1.5× longer, the saccus is very long and evenly broad with a rounded apex”, the *fallax*- and *balneariellus*-groups with “labial palps … almost straight and very long with third segment not turned upwards”, but they did not define groups for other *Megacraspedus* species and species coverage was incomplete in their groups.

*Systematic arrangement of species.* The sequence of species groups and species largely follows a maximum likelihood analysis of DNA barcode data, but also considers genitalia morphology which is partially but not fully concordant with molecular results. Species without molecular data are arranged according to morphological similarities.

***Megacraspedus* Zeller, 1839**

*Chilopselaphus* Mann, 1867

*Chilopselaphalus* Rebel, 1901 (incorrect subsequent spelling)

*Toxoceras* Chrétien, 1915 (homonym)

*Toxidoceras* Chrétien, 1923

*Nevadia* Caradja, 1920 (homonym)

*Cauloecista* Dumont, 1928, syn. n.

*Reichardtiella* Filipjev, 1931, syn. n.

*Vadenia* Caradja, 1933, syn. n.

***Megacraspeduslanceolellus* species group**

*Megacraspeduslanceolellus* (Zeller, 1850)

= *M.hessleriellus* Rössler, 1868

= *M.subdolellus* Staudinger, 1859, syn. n.

= *M.tutti* Walsingham, 1897, syn. n.

= *M.grossisquammellus* Chrétien, 1925, syn. n.

*Megacraspedusbengtssoni* sp. n.

***Megacraspedushomochroa* species group**

*Megacraspedushomochroa* Le Cerf, 1932

= *M.culminicola* Le Cerf, 1932, syn. n.

***Megacraspedusdolosellus* species group**

*Megacraspedusmonolorellus* Rebel, 1905

*Megacraspedusjunnilaineni* sp. n.

*Megacraspedusuzunsyrtus* Bidzilya & Budashkin, 2015

*Megacraspedussimilellus* sp. n.

*Megacraspedusgolestanicus* sp. n.

*Megacraspedustokari* sp. n.

*Megacraspedusdolosellus* (Zeller, 1839)

= *M.separatellus* (Fischer von Röslerstamm, 1843), syn. n.

= *M.incertellus* Rebel, 1930, syn. n.

***Megacraspedusfaunierensis* species group**

*Megacraspedusneli* sp. n.

*Megacraspedusfaunierensis* sp. n.

***Megacraspedusgredosenis* species group**

*Megacraspedusgredosensis* sp. n.

***Megacraspeduscuencellus* species group**

*Megacraspeduscuencellus* Caradja, 1920

*Megacraspedusbidentatus* sp. n.

*Megacraspedusfuscus* sp. n.

*Megacraspedustrineae* sp. n.

***Megacraspeduspusillus* species group**

*Megacraspedustristictus* Walsingham, 1910

*Megacraspedusalfacarellus* Wehrli, 1926

*Megacraspeduspusillus* Walsingham, 1903

*Megacraspedusskoui* sp. n.

*Megacraspedusspinophallus* sp. n.

*Megacraspedusoccidentellus* sp. n.

*Megacraspedusgranadensis* sp. n.

*Megacraspedusheckfordi* sp. n.

*Megacraspedustenuiuncus* sp. n.

***Megacraspeduslativalvellus* species group**

*Megacraspeduslativalvellus* Amsel, 1954

***Megacraspedusdejectella* species group**

*Megacraspedusdejectella* (Staudinger, 1859)

***Megacraspedusbinotella* species group**

*Megacraspedusdevorator* sp. n.

*Megacraspedusbinotella* (Duponchel, 1843)

*Megacraspedusbrachypteris* sp. n.

*Megacraspedusbarcodiellus* sp. n.

***Megacraspedusbilineatella* species group**

*Megacraspedusbilineatella* Huemer & Karsholt, 1996

*Megacraspedusandreneli* Varenne & Nel, 2014

*Megacraspedussumpichi* sp. n.

***Megacraspedusfallax* species group**

*Megacraspedustabelli* sp. n.

*Megacraspedusgallicus* sp. n.

*Megacraspedusribbeella* (Caradja, 1920), comb. n.

*Megacraspeduslibycus* sp. n.

*Megacraspedusnumidellus* (Chrétien, 1915), comb. n.

= *M.mareotidellus* Turati, 1924, syn. n.

*Megacraspedusalbovenata* Junnilainen, 2010

*Megacraspeduslongipalpella* Junnilainen, 2010

*Megacraspedusniphorrhoa* (Meyrick, 1926)

*Megacraspedusalbella* (Amsel, 1935), comb. n.

*Megacraspedusfallax* (Mann, 1867)

*Megacraspedusbalneariellus* (Chrétien, 1907)

*Megacraspeduspodolicus* (Toll, 1942)

*Megacraspeduskazakhstanicus* sp. n.

*Megacraspedusknudlarseni* sp. n.

***Megacraspedusmajorella* species group**

*Megacraspedusmajorella* Caradja, 1920

*Megacraspeduslatiuncus* sp. n.

***Megacraspedustenuignathos* species group**

*Megacraspedustenuignathos* sp. n.

***Megacraspedusglaberipalpus* species group**

*Megacraspedusglaberipalpus* sp. n.

***Megacraspedusimparellus* species group**

*Megacraspedusimparellus* (Fischer von Röslerstamm, 1843)

= *M.litovalvellus* Junnilainen, 2010, syn. n.

***Megacraspedusconsortiella* species group**

*Megacraspedusmultispinella* Junnilainen & Nupponen, 2010

*Megacraspedusnupponeni* sp. n.

*Megacraspeduscerussatellus* Rebel, 1930

*Megacraspedusattritellus* Staudinger, 1871

*Megacraspedusconsortiella* Caradja, 1920

*Megacraspeduspototskii* sp. n.

*Megacraspedusleuca* (Filipjev, 1929)

= *M.kaszabianus* Povolný, 1982, syn. n.

*Megacraspedusorenburgensis* Junnilainen & Nupponen, 2010

***Megacraspeduslagopellus* species group**

*Megacraspeduslagopellus* Herrich-Schäffer, 1860

*Megacraspeduscoleophorodes* (Li & Zheng, 1995)

*Megacraspedusfeminensis* sp. n.

*Megacraspeduskirgizicus* sp. n.

*Megacraspedusargyroneurellus* Staudinger, 1871

***Megacraspedusviolacellum* species group**

*Megacraspedusibericus* sp. n.

*Megacraspedusviolacellum* (Chrétien, 1915), comb. n.

= *M.chretienella* (Dumont, 1928), syn. n.

= *M.halfella* (Dumont, 1928), syn. n.

= *M.arnaldi* (Turati & Krüger, 1936), syn. n.

***Megacraspedussqualida* species group**

*Megacraspedussqualida* Meyrick, 1926

= *M.escalerellus* Schmidt, 1941, syn. n.

***Megacraspeduspentheres* species group**

*Megacraspeduspentheres* Walsingham, 1920

*Megacraspedussteineri* sp. n.

*Megacraspedusgibeauxi* sp. n.

*Megacraspedusmultipunctellus* sp. n.

*Megacraspedusteriolensis* sp. n.

*Megacraspeduskorabicus* sp. n.

*Megacraspedusquadristictus* Lhomme, 1946

*Megacraspeduseburnellus* Huemer & Karsholt, 2001

***Megacraspeduslongivalvellus* species group**

*Megacraspedusskulei* sp. n.

*Megacraspeduslongivalvellus* sp. n.

***Megacraspeduspeyerimhoffi* species group**

*Megacraspeduspeyerimhoffi* Le Cerf, 1925

***Megacraspeduspeslieri* species group**

*Megacraspeduspeslieri* sp. n.

***Megacraspedusgrisea* species group**

*Megacraspedusgrisea* (Filipjev, 1931), comb. n.

*Megacraspeduspacificus* sp. n.

*Megacraspedusarmatophallus* sp. n.

### Taxa misplaced in *Megacraspedus*

Numerous species from various regions have been described in *Megacraspedus*, but could not be confirmed to belong unequivocally to this genus. Although a revisionary work of these problems is outside the scope of our paper we present a short overview. Several species from the Afrotropical region were described in *Megacraspedus* or *Chilopselaphus* (Janse, 1958: 136), but none of these could be confirmed to belong in *Megacraspedus*. *M.brachypogon* Meyrick, 1937 and *M.coniogramma* Meyrick, 1921 are now placed in *Ephysteris* (Janse 1958: 136, Edwards 1996: 112), *M.peracuta* Meyrick, 1920 now in *Ischnocraspedus* (Janse 1958: 136), *M.photinopa* Meyrick, 1920 now in *Photodotis* (Beccaloni et al. 2005), and *M.suffusellus* Walsingham, 1891 now in *Pyncostola* (Bidzilya and Mey 2011: 2019. *M.taphrites* Meyrick, 1937 is a junior subjective synonym of *Dichomerisorthacma* Meyrick, 1926 (Janse 1858: 136), and also *Chilopselaphusethicodes* Meyrick, 1920 probably belongs to Dichomeridinae (type examined by OK). Finally the generic classification of *M.serica* Meyrick, 1909 had already been doubted by Janse (1958: 136–137).

Edwards (1996: 113) lists 23 species from Australia in *Megacraspedus*: *M.achroa* (Lower, 1901), *M.aenictodes* Turner, 1919, *M aphileta* Meyrick, 1904, *M argonota* (Lower, 1901), *M.astemphella* Meyrick, 1904, *M.centrosema* Meyrick, 1904, *M.chalcoscia* Meyrick, 1904, *M.coniodes* Meyrick, 1904, *M.euxena* Meyrick, 1904, *M.hoplitis* Meyrick, 1904, *M inficeta* Meyrick, 1904, *M.ischnota* Meyrick, 1904, *M.isotis* Meyrick, 1904, *M.melitopis* Meyrick, 1904, *M.niphodes* (Lower, 1897), *M.oxyphanes* Meyrick, 1904, *M.pityritis* Meyrick, 1904, *M.platyleuca* Meyrick, 1904, *M.popularis* Meyrick, 1904, *M.sagittifera* (Lower, 1900), *M.sclerotricha* Meyrick, 1904, *M.sematacma* Meyrick, 1921, and *M.stratimera* (Lower, 1897). Li and Sattler (2012: 58) transferred the Australian species under a revised *Pycnobathra* Lower, 1901 genus group, without formally combining them with any of the nine genera they recognize under this group. According to Li and Sattler (op. cit.) and Sattler (in litt.) the *Pycnobathra* genus group is not closely related to *Megacraspedus*. We did not examine *M.calamogonus* Meyrick, 1886 from New Zealand, but it should probably also be removed from *Megacraspedus*. Two species, *M.exilis* Walsingham, 1909 from Mexico and *M.plutella* (Chambers, 1874) from North America are currently listed in *Megacraspedus* but most likely they do not belong to this genus (see section Remarks on genus group names). Finally a few taxa from the Palearctic region were incorrectly placed in *Megacraspedus*. *M.jablonkayi* Gozmány, 1954 is a junior synonym of *Pycnostolabohemiella* (Nickerl, 1864) (Gozmány 1958: 289), *M.exoletellus* Erschoff, 1874 is now placed in *Pleurota* (Lvovsky 1990: 639), and *M.marocanellus* Lucas, 1932 probably belongs to the Autostichidae [type examined by OK].

### Key to species groups based on male genitalic characters

**Table:** 

1	Gnathos hook straight, massive and bulky, with longitudinal grooves (Figs 183–194)	***M.pusillus*** species group
–	Gnathos hook usually curved or bent, of various shape, without longitudinal grooves	**2**
2	Uncus apically rod-like, maximum half width of valva (Figs 239–244)	***M.lagopellus*** species group
–	Uncus without rod-like apical part, usually with rounded apex, two-thirds to about twice width of valva	**3**
3	Sub-basal part of valva with long and curved thorn-like process (Fig. 247)	***M.squalida*** species group
–	Sub-basal part of valva without process	**4**
4	Valva very broad, about width of uncus, with sclerotised subapical ridge (Figs 264–266)	***M.grisea*** species group
–	Valva slender, rarely broad but without sclerotised subapical ridge	**5**
5	Valva distinctly exceeding apex of uncus; phallus long and slender, without distinct sclerotisations (Figs 258–260)	***M.longivalvellus*** species group
–	Valva at most length of uncus; phallus of different shape, partially with sclerotisations	**6**
6	Uncus basally constricted, short, suboval	**7**
–	Uncus basally not constricted, sub-rectangular to sub-triangular	**8**
7	Sacculus usually present; phallus distally narrowing with sclerotised, denticulate ridge (Figs 248–257)	***M.pentheres*** species group
–	Sacculus absent; phallus distally with broadly sinusoid dorsal margin, without denticulate ridge (Figs 261–262)	***M.peyerimhoffi*** species group
8	Phallus with long and slender thorn-like sclerite from about middle nearly to apex, parallel to phallus axis (Fig. 263)	***M.peslieri*** species group
–	Phallus of different shape, without long and slender thorn	**9**
9	Phallus bent distad of bulbous of coecum, with long and slender distal part, 1–2 strong medial or sub-apical teeth	**10**
–	Phallus of different shape, without sclerotisation or with groups of smaller teeth or differently shaped sclerotisation	**11**
10	Sacculus well developed, digitate; phallus very long (Figs 175–176)	***M.gredosensis*** species group
–	Sacculus absent, phallus shorter (Figs 177–182)	***M.cuencellus*** species group
11	Uncus sub-quadrate, large, nearly width of posterior part of tegumen; ductus ejaculatorius with double-twisted interior sclerotisation (Figs 223–224)	***M.majorella*** species group
–	Uncus smaller, oblong; ductus ejaculatorius with or without interior sclerotisation of different shape	**12**
12	Sacculus well developed, digitate	**13**
–	Sacculus absent	**14**
13	Uncus oblong 1.7 times longer than maximum width; gnathos hook weakly curved; ductus ejaculatorius with broad and short interior sclerotisation (Figs 226–227)	***M.glaberipalpus*** species group
–	Uncus rounded, about same maximum length as width; ductus ejaculatorius with very long and 4–5× contorted interior sclerotisation (Figs 228–230)	***M.imparellus*** species group
14	Saccus large, oblong, apically usually broadly rounded, rarely pointed, with longitudinal sclerotised ridge extending almost to apex	**15**
–	Saccus shorter, sub-triangular, smoothly sclerotised without or with short ridge of various shape	**16**
15	Uncus small, about width of valva; gnathos hook straight; phallus with stout and broad distal part (Figs 173–174)	***M.faunierensis*** species group
–	Uncus large, distinctly broader than valva; gnathos hook curved to strongly bent; phallus with oblong and usually slender distal part (Figs 231–238)	***M.consortiella*** species group
16	Valva short and broad; saccus broadly suboval, with rounded apex (Figs 245–246)	***M.violacellum*** species group
–	Valva long, slender to moderately broad; saccus sub-triangular, with pointed apex	**17**
17	Saccus long and slender, ratio maximum width to length 0.6; phallus with small sub-apical spine and larger sub-apical tooth (Fig. 159)	***M.homochroa*** species group
–	Saccus short to moderately long, ratio maximum width to length minimum 0.8; phallus of different shape, exceptionally subapical tooth on right side and two small spines on left side	**18**
18	Gnathos hook long and slender, extending nearly to anterior margin of tegumen (Fig. 225)	***M.tenuignathos*** species group
–	Gnathos hook shorter, not extending to anterior margin of tegumen	**19**
19	Gnathos hook short, stout, curved; valva apically distinctly inflated, phallus with sclerotised, usually dentated ridge (Figs 206–222)	***M.fallax*** species group
–	Gnathos hook moderately short to long, slender; valva apically narrowing or contorted and rounded; phallus without sclerotised, dentated ridge	**20**
20	Saccus with short, furcated posterior ridges (Figs 197–201)	***M.binotella*** species group
–	Saccus without or with short, unfurcated ridge	**21**
21	Phallus with S-curvature (Figs 160–172)	***M.dolosellus*** species group
–	Phallus without S-curvature	**22**
22	Gnathos hook long, bent at right angles at about two fifth (Fig. 195)	***M.lativalvellus*** species group
–	Gnathos hook shorter, nearly straight to weakly curved	**23**
23	Phallus with broadly lobed dorsal and rod-like ventral sclerotisation (Figs 153–158)	***M.lanceolellus*** species group
–	Phallus of different shape, without broadly lobed dorsal sclerotisation	**24**
24	Valva broad, with longitudinal ridge (Fig. 196)	***M.dejectella*** species group
–	Valva slender, without ridge (Figs 202–205)	***M.bilineatella*** species group

## Taxonomic treatments of species

### *Megacraspeduslanceolellus* species group

The *M.lanceolellus* species group includes two species: *M.lanceolellus* and *M.bengtssoni* sp. n.

*External morphology*. Segment 2 of labial palpus with long scale brush; segment 3 shorter or about length of segment 2. Antennal scape without pecten. Wingspan (males) 12–15 mm. Forewing light greyish or yellowish with indistinctly lighter veins and 1–2 black dots. Known females slightly brachypterous.

*Genitalia morphology*. Male genitalia. Uncus moderately slender, sub-triangular; gnathos hook long and stout, about length of uncus; anterior margin of tegumen deeply emarginated; valva straight, slender, with weakly contorted and rounded apical part; saccular area setose, without separated sacculus; saccus nearly V-shaped, broad, abruptly tapered distally; phallus with band-like sclerotisation of coecum, broadly lobed dorsal and rod-like ventral sclerotisation, and one to two short and broadly linear interior sclerotisation of ductus ejaculatorius. Female genitalia. Papilla analis small; apophysis posterior very long; segment VIII long and slender, largely membranous; subgenital plate with sub-triangular subostial sclerotisation, anteromedially with long sinusoid projection; apophysis anterior rod-like, from posterior margin of segment VIII; colliculum short; signum small, rounded spiny plate.

*Diagnosticremarks*. The *M.lanceolellus* species group is defined by a unique combination of several structures in the male genitalia. The moderately large and apically narrowing uncus, the nearly straight gnathos hook, the slender and apically weakly contorted valva, the nearly V-shaped, broad saccus with a long medial ridge, and particularly the shape of the phallus with a broad dorsal lobe and a slender ventral sclerotisation as well as the linear sclerotisation of the ductus ejaculatorius are characteristic. The female genitalia overall largely agree with several taxa in other species groups.

#### Megacraspedus lanceolellus

Taxon classificationAnimaliaLepidopteraGelechiidae

(Zeller, 1850)

Ypsolophus lanceolellus Zeller, 1850: 143.
Megacraspedus hessleriellus Rössler, 1868: 347.
Megacraspedus subdolellus Staudinger, 1859: 243, syn. n.
Megacraspedus tutti Walsingham, 1897: 140, syn. n.
Megacraspedus grossisquammellus Chrétien, 1925: 257, syn. n.

##### Examined material.

The examined material cannot be unequivocally attached to barcode clusters due to lack of barcode sequences from several places, or partially sympatric clusters, and is thus listed in a standardized format in the alphabetic order of countries.

**Syntypes** [?], 2 ♂, *Ypsolophuslanceolellus*, [Italy] “Livorno Mn” “Gen. Präp. Mus. Vind. 16.653 ♂” (NHMW). **Lectotype** ♂, **here designated**, [Spain] *Megacraspedussubdolellus*, “Lecto-type” “20/8” “Granada m.” “Origin.” “Lectotype ♂ *Megacraspedussubdolellus* Stdgr teste K. Sattler, 1986” “ex coll. Staudinger” “GU 15/1404 ♂ P.Huemer” (ZMHU). **Paralectotypes**, 5 ♂, *Megacraspedussubdolellus*, all labelled “Para-lecto-type” “Origin.” “ex coll. Staudinger” (ZMHU). **Holotype** ♂, *Megacraspedustutti*, [France] “Holo-type” “La Grave 5000 ft Dauphiné FRANCE VIII.1896 Tutt. 8157” “Type” “Walsingham Collection, 1910–427.” “*Megacraspedus tutti* ♂ Wlsm. Ent. Rec. IX.140. (1897) TYPE ♂ (BMNH). **Paralectotypes**, 2 ♂, *Megacraspedusgrossisquamпellus*, [Spain] “TYPE” “*grossisquamellus*” “S. Ildef. 24.6.02” “25.6.02” “GENITALIA ♂ P. VIETTE PRÉP. NO. 3568” (MNHN) [photographs examined]; 2 ♂ (on same polyporus), “S.[an] Ildef.[onso] 7.02” “GU 15/1451 ♂ P.Huemer” (MNHN). **Non-type material.** Andorra. 1 ♂, Sant Julia, Fontaneda, 1300 m, 6.viii.2003, leg Viehmann (RCWS); 3 ♂, 1 ♀, Port de Cabús, 2290 m, 16.vii.2012, leg. P. Huemer, genitalia slides GEL 1192 ♂ Huemer, GEL 1257 ♀ Huemer (TLMF). Croatia. 1 ♀, Krk, Misučaynica, 5.viii.1967, leg. G. Baldizzone (ZMUC); 1 ♀, Krk, str. Krk-Vrbnik, 20.vii.1998, leg. G. Baldizzone (TLMF); 1 ♂, Krk isl., dint. di Poljica, 10.vii.2012; 4 ♂, Krk isl., Mt. Hiam, Branušine, 20–22.vi.2013, 180 m, leg. G. Baldizzone (RCGB; ZMUC); 2 ♂, Gorne Bilišane, 6.vii.2004, leg. Z. Tokár; 1 ♂, Bilišane, 300 m, 23.vi.2006, leg. Z. Tokár (all RCZT); 2 ♂, Pag, 10.vi.2015, leg. J. Junnilainen (RCJJ). Italy. 1 ♂, prov. Alessandria, Parco NR Cappane di Marcarolo, sent. Lago Badana, 800 m, 27.vi.2002, leg. G. Baldizzone; 1 ♂, same data, but Monto Peggio, 900 m, 12.vii.2002 (all RCGB); 4 ♂, prov. Pordenone, Vivaro, Magredi di Vivaro, 110 m, 6.vi.2008, leg. P. Huemer, genitalia slide GEL 1220 Huemer; 1 ♂, same data, but 7 km SSW Vivaro, 5.vi.2005; 1 ♂, prov. Pordenone, San Quirino, Magredi di San Quirino, 100 m, 11.vi.2001, leg. P. Huemer (all TLMF); 3 ♂, Livorno, 1872, leg. J. Mann (NHMW); 1 ♂, prov. Livorno, Antignana, 10 m, 26.vi.2004, leg. J. Liška (NMPC); 1 ♂, prov. Roma, Bracciano, 20–22.vi.2001, leg. A. Cox (ZMUC); 3 ♂, prov. Roma, Monti di Tolfa, dint. Manziana, 23–28.vi.1989, leg. G. Baldizzone (TLMF, RCGB); 1 ♂, prov. Latina, Aurunci, 4 km NW Castelforte, 400 m, 1.vii.1969, leg. R. Johansson, genitalia slide 3155 Karsholt; 1 ♂, prov. Latina, Maranola, 400 m, 19.vi.1969, leg. R. Johansson; 1 ♂, prov. Torino, Torre del Colle, Villa Dora (To), 500 m, 5.vii.1991, leg. U. Parenti; 1 ♀, same data, but 9.vii.1991; 2 ♂, prov. Puglia, 5 km SW Manfredònia, 50 m, 26.v.2005, leg. P. Skou (all ZMUC); 1 ♂, prov. Albenga, Salea, 200 m, 4.vii.2012, leg. J. Skyva; 2 ♂, same data, but 8.vii.1996, leg. J. Liška (all NMPC); 4 ♂, prov. Calábria, Monte Pollino, Timpa del Demonio, 1200 m, 11.vii.1991, leg. G. Baldizzone (RCGB); 4 ♂, same data, but 3 km N Civita, 800 m, 31.v.–1.vi.2005, leg. P. Skou (TLMF, ZMUC); 1 ♂, same data, but 2 km NNW Civita, 775 m, 26.vii.2011, leg. P. Skou & B. Skule (ZMUC). France. 1 ♂, Dep. Alpes Maritimes, Caussols, Mgne du Cheiron, 1150 m, 12.v.2007, leg. J. Skyva (NMPC); 1 ♂, Dep. Alpes Maritimes, Caussols, 1100 m, 22.vi.2002, leg. J. Nel; 6 ♂, same data, but 14.viii.1971, leg. F. Dujardin; 4 ♂, Dep. Alpes Maritimes, St. Barnabé, 950 m, 14.viii.1953, leg. F. Dujardin; 1 ♂, Dep. Alpes Maritimes, La Turbie, 250 m, 30.v.–2.vi.1959, leg. K. Burmann; 1 ♂, Dep. Alpes Maritimes, Coursegoules, Nougueiret, 945 m, 6.vii.1995, genitalia slide 3988 Nel; 1 ♂, Dep. Alpes-de-Haute-Provence, Col d´Allos, W, 2300 m, 25.vii.1999, leg. J. Nel, gen. slide 9390 Nel; 1 ♂, same data, but 9.vii.2005, gen. slide 19514 Nel; 1 ♂, Dep. Alpes-de-Haute-Provence, Allos, 1500 m, 30.vii.1972, leg. F. Dujardin; 1 ♂, Dep. Alpes-de-Haute-Provence, St. Etienne, St. Sebastiene, 12.v.2001, leg. J. Nel; 1 ♂, Dep. Alpes-de-Haute-Provence, Dabisse, 3.vii.2005, leg. J. Nel; 1 ♀, Dep. Alpes-de-Haute-Provence, Digne, 600 m, 2–5.vi.1959, leg. K. Burmann, genitalia prep. 3769 Z. Tokàr (in glycerin); 2 ♂, Dep. Hautes Alpes, Les Vigneaux, 1200 m, 25.vii.1990, leg. P. Huemer & G. Tarmann (all TLMF); 1 ♀, Dep. Hautes Alpes, Les Vigneaux, 1000 m, 12.vii.2002, leg. J. Junnilainen; 1 ♂, same data, but Les Vigneaux 5 km W, 1250 m, 5–6.vi.2003; 1 ♂, 1 ♀, Dep. Hautes Alpes, Embrun, 850 m, 6–7.vii.2002, leg. J. Junnilainen, genitalia slide GU 16/1472 ♀ Huemer; 1 ♂, Dep. Hautes Alpes, Col de la Cayolle, Estene, 1800 m, 25.vi.2006, leg. J. Junnilainen (all RCJJ); 1 ♂, Dep. Hautes Alpes, La Bessèe, 1100 m, 21.vii.1961, leg. K. Burmann; 1 ♂, Dep. Hautes Alpes, Ristolas, Le Brasc1640 m, 13.vii.1990, leg. R. Robineau, genitalia slide 2078 Nel; 1 ♂, Dep. Hautes Alpes, Col de Montgenèvre, 1800 m, 1.viii.1998, leg. J. Nel, genitalia slide 8536 Nel; 1 ♂, Dep. Hautes Alpes, Pelvoux, 2300 m, 29.vii.1994, leg. J. Nel, genitalia slide 2276 Nel; 1 ♂, Dep. Hautes Alpes, Villard, St. Crepin, 4.vi.2005, leg. J. Nel (all TLMF); 3 ♂, Dep. Hautes Alpes, Céreste, 8–17.vi.2010 (RCHW, ZMUC); 1 ♂, Dep. Ardèche, Lablachere, 2.vii.2004, leg. J. Procházka (NMPC); 1 ♂, Dep. Vaucluse, Mt. Ventoux, 18–19.v.1990, leg. G. Luquet, genitalia slide 4660 Nel; 2 ♂, same data, but 30–31.v.1990, genitalia slides 4660 Nel, 4654 Nel; 1 ♂, Dep. Vaucluse, Méthamis, 1.viii.1995, leg. G. Brusseaux; 1 ♂, same data, but 30.vii.1995, genitalia slide 44165 Nel; 2 ♀, Dep. Var, Val de Purian, Rougiers, 15.vi.1994, leg. J. Nel, gen. slide 2116 Nel; 1 ♂, Dep. Var, Camillier, Montmeyan, 550 m, 13.vi.1994, leg. T. Varenne, genitalia slide 2551 Nel; 1 ♂, Dep. Var, Caramy, 15.vi.1997, leg. J. Nel, genitalia slide 6048 Nel; 1 ♂, Dep. Var, Sommet Mt. Caume, 1.vii.1995, leg. J. Nel, genitalia slide 3517 Nel; 1 ♂, Dep. Var, Pourrières, riv. de l´Arc, 27.v.2000, leg. J. Nel, genitalia slide 11372 Nel; 1 ♂, Dep. Var, Mtgne. Lachens, 1600 m, 7.vii.1994, leg. J. Nel, genitalia slide 2225 Nel; 1 ♂, same data, but 11.vii.1995; 1 ♂, Dep. Var, Plan d´Aups, Bertagne, 850 m, 24.v.1999, leg. J. Nel, genitalia slide 10708 Nel; 1 ♂, Dep. Var, Puits de Rians, 20.vi.2992, leg. J. Nel, genitalia slide GEL 1222 Huemer; 1 ♂, Dep. Var, Signes, le Camp, 24.v.1998, leg. J. Nel (all TLMF); 1 ♂, Dep. Var, Montmeyan, 10.vi.1981, leg. Hahn (RCEA); 1 ♂, Dep. Bouches-du-Rhône, La Ciotat, Roumagoua, 30.v.1995, leg. J. Nel, genitalia slide 3449 Nel (TLMF); 1 ♂, Provence, 3 km W Cereste, 3–4.vii.1989, leg. B. Å. Bengtsson, genitalia slide Bengtsson 3266 (RCBB); 1 ♂, Rhône-Alpes, Montelimar, 27.vi.1980, leg. K. Schnack; 1 ♂, Alpes Maritimes, Col de Vence, 11–12.vi.1981, leg. Hahn (ZMUC); 1 ♂, Dep. Ardèche, St. Laurent-du-Pape, 400 m, 20–21.vi.1999, leg. P. Skou; 1 ♀, Dep. Hautes-Alpes, Châteauvieux, 750 m, 26.vi.1996, leg. A. Cox; 1 ♂, same data, but 3.vii.1998; 2 ♂, Dep. Hautes-Alpes, Réotier, 1000 m, 23–30.vii.1990, genitalia slide 4516, 4517 Hendriksen; 1 ♂, Dep. Hautes-Alpes, 1.2 km SSW Prelles, 1200 m, 2.vii.2017, leg. P.Skou (all ZMUC); 1 ♂, Dep. Var, Pont l’Arbuty, 800 m, 5.vii.1994, leg. K. Larsen (ZMUC); 1 ♂, Dep. Vaucluse, Vaison la Romaine, 200 m, 17.vii.1991, leg. A. Cox, genitalia slide 4518 Hendriksen (ZMUC). Montenegro. 3 ♂, Fundina, 15.vi.2011, leg. I. Richter. genitalia slide 5315 Karsholt (RCIR, ZMUC). Spain. 1 ♂, prov. Alicante, Sierra de Crevillente, 5 km NE Albatera 450 m, 23.v.2004, leg. P. Huemer, genitalia slide GEL 1238 Huemer (TLMF); 1 ♂, prov. Alicante, 3.8 km NW Torremendo, 5.v.2008, leg. J. Tabell (ZMUC); 2 ♂, prov. Almería, Sierra de los Filabres, Calar Alto, 2130 m, 5.vii.2015, leg. J. Tabell, genitalia slide GU 16/1430 Huemer (TLMF, ZMUC); 2 ♂, same data, but 2000 m, 1–2.viii.2010, leg. J. Šumpich, genitalia slide GU 16/1436 Huemer; 1 ♂, same data, but route Purchena - Senes, 1600 m, 16.vi.2007, leg. J. Šumpich (all NMPC); 2 ♂, same data, but Calar Alto, 1900–2022 m, 17–18.vi.2007, leg. J. Šumpich (NMPC); 1 ♂, prov. Almería, Tabernas, 380 m, 6–8.vii. 2007, leg. G. Jeppesen (ZMUC); 1 ♂, prov. Almería, Maria, 1200 m, 18–25.vi.2008, leg. M. Delnoye (ZMUC); 1 ♂, prov. Avila, Sierra de Villafrance, 1 km W La Herguijuela, 1650 m, 20.vii.2003, leg. B. Skule (ZMUC); 1 ♂, prov. Avila, Sierra de Gredos, below Platforme de Gredos, 1700 m, 21.vii.2003, leg. B. Skule; 1 ♂, prov. Burgos, Espinosa de Cervera, 24.vii.1988, leg. M. Hull (ZMUC); 1 ♂, same data, but 1650 m, 25.vi.1986, leg. P. Skou (ZMUC); 2 ♂, prov. Castellón, 20 km SE Morella, 15.vi.1989, leg. B. Å. Bengtsson, genitalia slide Bengtsson 3264 (RCBB, ZMUC); 1 ♂, prov. Castellon, 5 km E Cuevas de Vinroma, 200 m, 13.vii.1992, leg. M. Fibiger; 1 ♂, prov. Castellon, 25 km NW Castellon, La Banderetta pass, 800 m, 17.vii.1992, leg. M. Fibiger (all ZMUC); 1 ♂, prov. Girona, Port Bou, 0–600 m, 18.vi.–1.vii.1963, leg. M. & W. Glaser (SMNK); 2 ♂, same data, but 0–300 m, 9–24.vi.1964 (SMNK); 3 ♂, 1 ♀, prov. Girona, Cerdanya, Colle del Moixeró, 1972 m, 18.vii.2012, leg. J. Dantart, genitalia slide GU 16/1412 ♂ Huemer (RCJD, TLMF); 4 ♂, prov. Lleida, Puente de Montanana, 15.vi.2012, leg. P. Huemer & A. Mayr (TLMF); 3 ♂, prov. Zaragoza, Gelsa 8 km NE, 240 m, 19.v.2016, leg. J. Tabell (ZMUC); 5 ♂, prov. Zaragoza, Caspe 7 km N, 18.v.2004 (RCJJ); 6 ♂, prov. Huesca, Los Monegros, Castejón de Monegros, 570 m, 10.vi.2007, leg. J. Šumpich; 3 ♂, same data, but 20.vi. 2007; 1 ♂, same data, but 26–27.iv.2003 (all NMPC); 1 ♂, prov. Huesca, 8 km S Candasnos, Barranco de Valcuerna, 175 m, 5.vii.2002, leg. B. Skule (ZMUC); 2 ♂, same data, but 250 m, 28.vi.2005, leg. D. Feierabend; 1 ♂, same data, but 10 km S, 30.v.2015, leg. J. Viehmann; 1 ♂, same data, but 3.vii.2016 (all RCWS); 1 ♂, prov. Soria, El Burgo de Osma, 895 m, 14–15.vi.1995, leg. A. Cox, genitalia slide 6104 Wolf (RCHW); 2 ♂, prov. Soria, 30 km SW Soria, El Temerosa, 1080 m, 18.vii.2012, leg. T. Nupponen, genitalia slide 5015 Tabell (ZMUC); 1 ♂, prov. Tarragona, 2 km S Miami Hospital, 0 m, 12.vii.1992, leg. M. Fibiger (ZMUC); 2 ♂, prov. Teruel, Albarracin, 1100 m, 19.vi.2007, leg. J. Šumpich; 1 ♂, same data, but 3.v.2003; 5 ♂, 1 ♀, same data, but 28.vii.2010; 1 ♂, same data, but7.viii.2010; 1 ♂, same data, but 13.vii.2012, leg. M. Dvorak; 2 ♂, same data, but 14.vii.2012, leg. M. Dvorak (all NMPC); 26 ♂, 1 ♀, prov. Teruel, Sierra de Cucalon, Baguena 5 km E, 15.vi.2004, leg. J. Junnilainen (RCJJ); 1 ♂, prov. Teruel, Cosa, 2–13.viii.1989, leg. C. Gielis (RCHW); 1 ♂, prov. Teruel, Albarracin, 26.vi.1982, leg. C. Gielis; 1 ♂, same data, but 1000–1200 m, 4–8.viii.1989, leg. C. Gielis; 2 ♂, Teruel, 1 km E Albarracin, Valdevacar, 12.vii.2001, leg. C. Gielis (all RMNH); 6 ♂, prov. Teruel, Albarracin, 4.5 km NE, 1110 m, 7.vii.2016, leg. J. Tabell (TLMF, ZMUC); 3 ♂, prov. Teruel, Albarracin, Val de Vacar, 1200 m, 16.vii.1992, leg. M. Fibiger; 3 ♂, same data, but 1250 m, 17–18.vii.1988, leg. M. Fibiger, genitalia slide 4544 van der Wolf (ZMUC); 1 ♂, same data, but 5.vi.1993, leg. J. Wolschrijn (ZMUC); 1 ♂, same data, but 1100 m, 22.v.1998, leg. P. Skou; 1 ♂, same data, but 10.vi.1999; 4 ♂, same data, but 12.vi.1999 (ZMUC); 2 ♂, same data, but 15.vi.2004, leg. Viehmann, genitalia slide 9217 van der Wolf (RCWS); 1 ♂, same data, but 1200 m, 4.viii.2007, leg. B. Skule & P. Skou (ZMUC); 1 ♀, prov. Teruel, Albarracin, 1100 m, 14.vi.1986, leg. P. Skou; 3 ♂, same data, but 1000 m, 7.viii.1988 (ZMUC); 1 ♂, same data, but, 1170 m, 8–10.vi.1994, leg. A. Cox, genitalia slide 4514 Hendriksen (ZMUC); 1 ♂, same data, but, 1170 m, 18–20.vi.1996, leg. A. Cox, genitalia slide 4513 Hendriksen (ZMUC); 1 ♂, same data, but 2–4.vi.1999, leg. A. Cox (ZMUC); 2 ♂, same data, but 1200 m, 11.vi.1999, leg. P. Skou (ZMUC); 1 ♂, same data, but 3 km W, 1200 m, 1.vii.2005, leg. D. Feierabend (RCWS); 1 ♂, same data, but 3.5 km SWW, 1200 m, 13.vi.2010, leg. J. Tabel, genitalia in tube (ZMUC); 1 ♂, same data, but 2 km NW, 1100 m, 4.vii.2010, leg. Z. Tokár (RCZT); 7 ♂, same data, but 4.5 km N, 1110 m, 7.vii.2016, leg. J. Tabel (ZMUC); 1 ♂, prov. Teruel, Pozondon, 1500 m, 21.vii.1984, leg. W. O. De Prins (ZMUC); 2 ♂, prov. Teruel, 5 km NW Montalban, 950 m, 17.vii.2003, leg. B. Skule (TLMF, ZMUC); 2 ♂, same data, but 5.viii.2007, genitalia slide 6491 Hendriksen (ZMUC);1 ♂, prov. Teruel, 1 km E Tramacastillo, 1250 m, 3.ix.2001, leg. B. Skule & BC. Hviid (ZMUC); 13 ♂, prov. Teruel, Moscardon, 1500 m, 3.vii.2016, leg. J. Viehmann (RCWS); 1 ♂, 1 ♀ prov. Granada, Sierra de Alfacar, 1500 m, 27.vi.1968, leg. K. Sattler & D.J. Carter, gen. slides 33657 ♂, 33658 ♀ (BMNH); 4 ♂, prov. Granada, Sierra Nevada, El Pardor Nat, 2500 m, 21.vii.1980, leg. E. Traugot-Olsen; 1 ♂, same data, but 23.vii.1983, genitalia slide 5777 Traugott-Olsen; 1 ♂, prov. Granada, Sierra Nevada, Camino de Valeta, 2300 m, 23.vii.1983, leg. G. Baldizzone & P. Triberti; 1 ♂, same data, but 2250 m, 2.viii.1984, leg. E. Traugott-Olsen; 1 ♂, same data, but 17.viii.1984; 1 ♂, same data, but 2300 m, 19.viii.1984; 1 ♂, same data, but 2050 m, 6.viii.1986; 3 ♂, same data, but 2250 m, 21.vii.1985, leg. G. Baldizzone & E. Traugott-Olsen, genitalia slide 6534 Hendriksen; 1 ♂, same data, but 23.vii.1985 (all ZMUC); 1 ♂, same data, but 1.viii.1984, leg. E. Traugott-Olsen, genitalia prep. 4128 Z. Tokár (in glycerin) (NHMW); 1 ♂, Granada, Puerto de la Ragua, 1800 m, 23.vii.2003, leg. P. Skou; 1 ♂, Granada, Puerto de la Ragua, 2200 m, 3.viii.1982, leg. W.O. De Prins (all ZMUC); 1 ♂, Granada, Cam. Baza-Benamaurel, 15 km de Baza, 16.vii.1987, leg. G. Baldizzone & E. Traugott-Olsen; 3 ♂, same data, but 19.vii.1987; 2 ♂, same data, but 20.vii.1987 (all TLMF); 1 ♂, Segovia, San Ildefonso 26.vii.1884, coll Staudinger (ZMHU); 2 ♂, prov. Zaragoza, Tosos, 600 m, 16.vi.1999, leg. P. Skou (ZMUC); 1 ♂, prov. Zaragoza, Nuévalos, 1200 m, 9.vi.2003, leg. H. van der Wolf (RCHW); 5 ♂, prov. Zaragoza, 6 km W Bujaraloz, 300 m, 29.v.2015, leg. J. Viehmann (RCWS, ZMUC). Without locality: 2 ♂, [San Ildefonso?] 26.vi.[year unknown], coll. Staudinger; 1 ♂, same data, but 13.vii.; 1 ♂, same data, but 26.vii. (all ZMHU).

##### Redescription.

**Figures 7–12.**
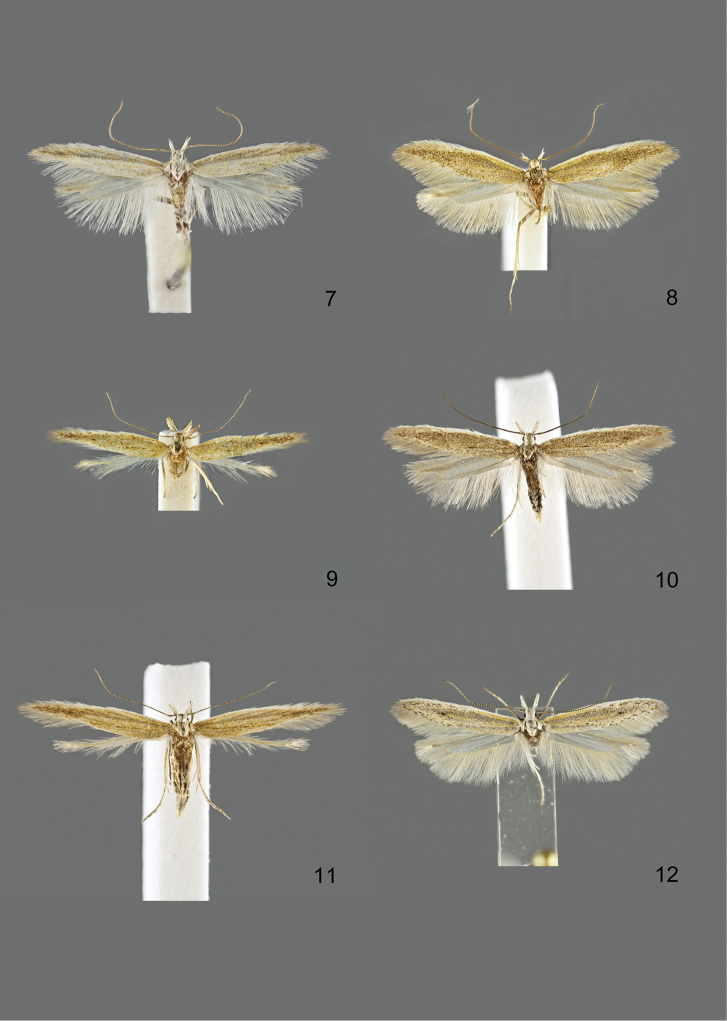
*Megacraspedus* adults in dorsal view. **7***M.lanceolellus* (Zeller, 1850) – male, Italy (NMPC) **8***M.lanceolellus* (Zeller, 1850) – male, France (TLMF) **9***M.lanceolellus* (Zeller, 1850) – female, France (TLMF) **10***M.lanceolellus* (Zeller, 1850) – male, Spain (TLMF) **11***M.lanceolellus* (Zeller, 1850) – female, Spain (TLMF) **12***M.lanceolellus* (Zeller, 1850) – male, Spain (ZMUC).

**Figures 13–18.**
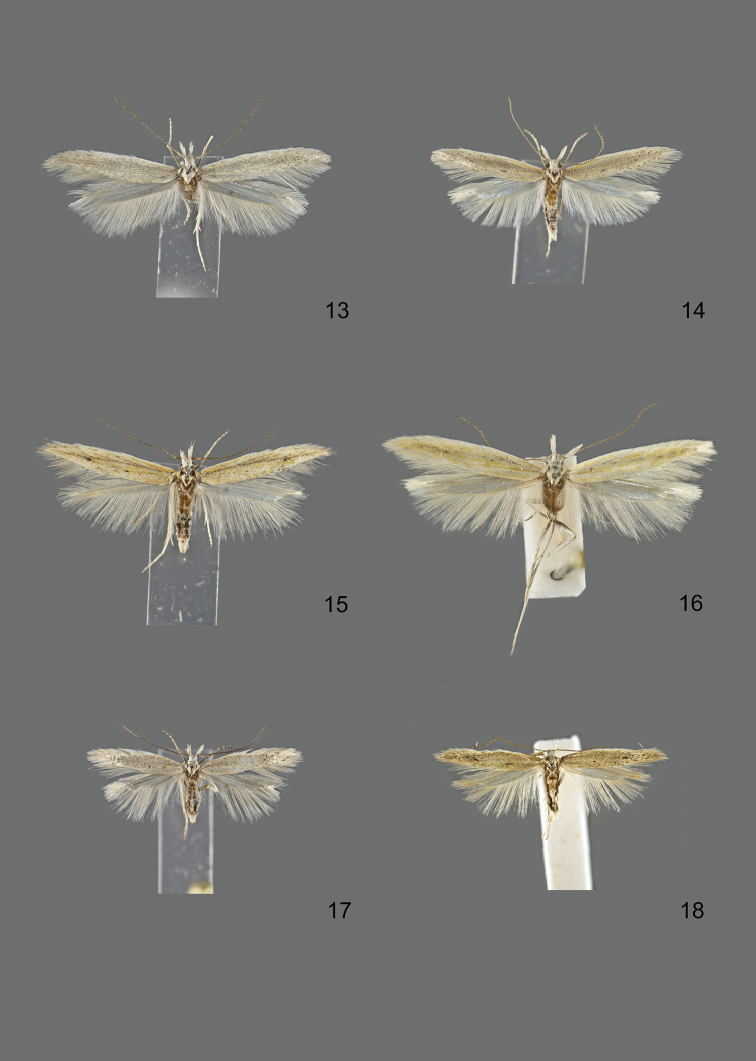
*Megacraspedus* adults in dorsal view. **13***M.lanceolellus* (Zeller, 1850) – male, Spain (TLMF) **14***M.lanceolellus* (Zeller, 1850) – male, Spain (TLMF) **15***M.lanceolellus* (Zeller, 1850) – male, Spain (ZMUC) **16***M.lanceolellus* (Zeller, 1850) – male, Spain (ZMUC) **17***M.bengtssoni* sp. n. – Holotype, male, Spain (ZMUC) **18***M.bengtssoni* sp. n. – Paratype, male, Spain (ZMUC).

Adult. *Male* (Figs 7–8, 10–16). Wingspan 12–17 mm. Segment 2 of labial palpus with long scale brush, brownish at outer and inner surface, white at lower and upper surface; segment 3 about half length of segment 2, white with black tip. Antennal scape without pecten; flagellum ringed black and whitish brown. Head, thorax and tegula whitish brown. Forewing light yellow, mottled with brown-tipped scales, forming a darker area in costal part of wing; costa white; veins indistinctly white; a small black dot at end of cell; a dark streak towards apex; fringes whitish grey. Hindwing light grey with cream-white fringes.

*Female* (Figure 9). Wingspan 12–17 mm. Forewing ellipsoid with elongate apex; same colour as male, but apex with more black scales. Hindwing distinctly reduced in width; apex produced. Otherwise similar to male.

*Variation*. *Megacraspeduslanceolellus* shows both individual and also a tendency to geographical variation. The nominotypical form has light yellowish forewings with a dark shadow or streak towards the apex, but such individuals also occur elsewhere, e.g., in Spain. Specimens from south-western France and northern and central Spain (described as *M.tutti* and *M.grossisquammellus*, respectively) have a more or less pronounced white costa and can moreover often be recognized by the light yellow forewings with darker veins. Specimens from the Sierra Nevada in southern Spain (described as *M.subdolellus*) are larger on average (wingspan 14–20 mm) and are characterised by their more clear yellow forewings that almost lack black dots, and the whitish costa. Specimens from Montenegro have a black dot at 2/5 in the fold.

*Male genitalia* (Figs 153–156). Uncus moderately slender, nearly twice as long as maximum basal width, sub-triangular, gradually tapered to weakly rounded apex; gnathos hook stout, slightly longer than uncus, with curved and pointed apex; anterior margin of tegumen with broad, medially deep V-shaped emargination; pedunculi small; valva straight, slender, extending to about base of gnathos, apex weakly contorted, rounded, saccular area densely covered with setae, without separated sacculus; posterior margin of vinculum with moderately deep medial emargination, broadly rounded lateral humps, vincular sclerites broadly sub-rectangular, tapered towards valva, with sclerotised posterior edge; saccus long and moderately broad, almost V-shaped, with abruptly tapered distal part and pointed apex, ratio maximum width to length 0.75, posterior margin strongly bulged, with rounded mediolateral projections, separated by moderately shallow incision, medial part with elongated ridge from posterior edge to middle, lateral sclerites of variable length, slightly shorter to longer than maximum width of saccus; phallus about length of tegumen, stout, straight, with bulbous coecum, sclerotised band in anterior part of coecum, distal two-thirds stout, with rod-like ventral and lobed dorsal sclerotisations, ductus ejaculatorius with two linear interior sclerotisations.

*Female genitalia* (Figs 267–268). Papilla analis small, apically rounded; apophysis posterior slender rod-like, about 4 mm long, with short, bifurcate posterior end, bordered by minute sclerotised field, apex widened and rounded; segment VIII approximately 1.5 mm long, membranous; subgenital plate with sub-triangular subostial sclerotisation, posteriorly extended into shortly pointed sclerites, delimiting trapezoidal ostium bursae, anterior margin with rod-like edge connected with apophysis anterior, medially with long sinusoid projection; apophysis anterior slender, rod-like, at most one-third length of segment VIII, posteriorly becoming rod-like venula of segment VIII, extending to posterior margin; colliculum short, sclerotised; ductus bursae short, moderately broad; corpus bursae, moderately short and broad, weakly delimited from ductus bursae, entire length of ductus and corpus bursae nearly 2 mm; signum small, rounded spiny plate.

##### Diagnosis.

Due to its variation *M.lanceolellus* is difficult to characterise. It is a medium-sized species with light yellowish forewings, mottled with brown and black-tipped scales, a lighter costa, and sometimes one or two black dots in the middle of the wing and/or a black streak towards apex. See also *M.bengtssoni* sp. n. (p 37) and *M.andreneli* (p 96). The male genitalia are characterised by several structures such as the sub-triangular shape of the uncus, the slender and apically weakly contorted valva, the shape of the saccus with a long medial ridge, and particularly the massive distal part of the phallus with a broadly lobed dorsal and a slender ventral sclerotisation, as well as the two linear sclerotisations of the ductus ejaculatorius. They are somewhat similar to *M.bengtssoni* sp. n. but differ e.g., by the longer uncus, the medially strongly emarginated anterior margin of the tegumen, the longer and more slender valva, and the broader phallus.

The female genitalia are characterised by the long sinusoid anteriomedial projection of segment VIII, but in the absence of females of closely related species such as *M.bengtssoni* sp. n., these features may not be specifically diagnostic.

##### Molecular data.

The extraordinary DNA barcode divergence is reflected by 19 BINs! The mean and maximum intraspecific divergence of the barcode region in this species is 7.6% and 12.5% respectively, largely reflecting a geographic pattern with several distinct clusters and a remarkable divergence of 4.3 to 8.1% to the nearest cluster. However, even within these clusters intraspecific variation of the barcode region is considerable with e.g., mean divergence of 2.5% in south-western alpine populations. Four specimens collected in the same microhabitat at Sierra de los Filabres (prov. Almería, Spain) form two distinct clusters with a variation of maximum 0.2% within clusters but minimum 8.6% between these clusters. Similarly diverging sympatric clusters are reported from prov. Teruel, and from Prov. Lleida.

The following 19 clusters are defined (based on sequenced material):

BIN lanc01 (Italy: Pordenone, Calabria; Croatia): BOLD:AAU2834 (n = 4).

BIN lanc02 (Italy: Calabria): BOLD:ACZ3381 (n = 1).

BIN lanc03 (Croatia): BOLD:ACZ2933 (n = 1).

BIN lanc04 (Italy: Livorno): BOLD:ACZ2657 (n = 1).

BIN lanc05 (Spain: Almeria): BOLD:ACZ9024 (n = 2).

BIN lanc06 (Andorra): BOLD:ACA9759 (n = 2)

BIN lanc07 (Spain: Barcelona): BOLD:ACZ8656 (n = 2).

BIN lanc08 (France: Alpes Maritimes): BOLD:ABA3648 (n = 1).

BIN lanc09 (Italy: Savona): BOLD:ACZ2656 (n = 1).

BIN lanc10 (France: Alpes Maritimes, Hautes Alpes, Var): BOLD:ABA3649 (n = 2).

BIN lanc11 (Spain: Almeria): BOLD:ACZ2607 (n = 2).

BIN lanc12 (Spain: Teruel, Valencia): BOLD:AAU1829 (n = 5).

BIN lanc13 (Spain: Soria): BOLD:ACZ8269 (n = 1).

BIN lanc14 (Spain: Teruel): BOLD:ADF2256 (n = 2).

BIN lanc15 (Spain: Teruel): BOLD:ACM1006 (n = 1).

BIN lanc16 (Spain: Zaragoza): BOLD:ADB7571 (n = 1).

BIN lanc17 (Spain: Zaragoza): BOLD:ADF2263 (n = 3).

BIN lanc18 (Spain: Lleida): BOLD:ADF2194 (n = 1).

BIN lanc19 (Spain: Lleida): BOLD:ADF1916 (n = 1).

The minimum distance to the nearest congeneric neighbour *M.dolosellus* is 9.9% (p-dist).

##### Distribution.

Southern parts of Europe, from the Balkan Peninsula to Italy, France, and Spain, also known from Germany (northwards to Rheinland-Pfalz (Hausenblas 2006: 10)). According to Mariani (1943: 174) also in Sicily and Corsica.

##### Biology.

Early stages are undescribed. According to Lhomme (1946: 538 as *M.tutti*) the larva feeds within the stem of *Festuca* L. The adults have been collected from late April until the middle of August, from low altitudes to 2300 m in the Alps and 2500 m in the Sierra Nevada. There is probably only one generation each year, and the flight period seems to at least partly depend on the altitude of the collecting site.

##### Remarks.

*Ypsolophuslanceolellus* was described from an unstated number of males collected by J. Mann at Ardenza and Salviano, Italy (Zeller 1850).

*Megacraspedushessleriellus* was described from two males collected at Biebrich and Mombach in Nassau, S Germany (Rössler 1866: 348). It was synonymised with *M.lanceolellus* by Heinemann (1870: 349).

*Megacraspedussubdolellus* was described from an unspecified number of both sexes collected in August at high altitudes (ca. 2700 m) in the Sierra Nevada, Spain (Staudinger 1859). Despite the slightly differing phenotypic appearance (see above) we found no sign of diagnostic characters in genitalia structures and thus consider *M.subdolellus* to be conspecific with *M.lanceolellus* (syn. n.). DNA barcodes are not yet known from the type locality. A lectotype, already labelled as such by K Sattler, is here designated in order to fix the identity of the species and conserve stability of nomenclature.

*Megacraspedustutti* was described from a single male collected in August at La Grave, SE France by JW Tutt (Walsingham 1897). Despite of the slightly differing phenotypic appearance compared to nominotypic *M.lanceolellus* (see above) we found no sign of diagnostic characters in genitalia structures and thus consider *M.tutti* to be conspecific with *M.lanceolellus* (syn. n.). *Megacraspedusgrossisquammellus* was described from an unstated number of specimens collected in Spain, Segovia, San Ildefonso, in June and July 1902. A lectotype was designated by Agenjo (1962: 161, pl. 2–3) and figured together with its genitalia. Two paralectotypes and numerous additional material from Spain examined by us show a considerable individual variation but cannot be reliably separated from *M.lanceolellus* in other areas, particularly in genitalia characters. We therefore consider *M.grossisquamпellus* to be conspecific with *M.lanceolellus* (syn. n.).

*Megacraspeduslanceolellus* shows a remarkable morphological and genetic variation. Barcode data clearly support several putative taxa, but the morphology gives a different and less straightforward picture. Though the phenotypic appearance partly depends on geography it is frequently impossible to reliably assign specimens to a barcode cluster. Male genitalia morphology is comparatively uniform and alleged diagnostic characters show intraspecific variation. Considering the intrapopulational barcode variation which exceeds 8% on one occasion we conclude that *M.lanceolellus* is a widespread, mainly western-Mediterranean species. The exceptional DNA barcode divergence may be explained by weak dispersal ability, particularly of the slightly brachypterous females, leading to several genetically isolated populations which are mainly restricted to various mountain systems. However, it seems unlikely that this is the sole cause for the observed intraspecific variation (see Discussion).

#### Megacraspedus bengtssoni sp. n.

Taxon classificationAnimaliaLepidopteraGelechiidae

http://zoobank.org/DD8A8B50-77B7-4966-B142-D90CF4BB68C4

##### Examined material.

**Holotype** ♂, “SPAIN [prov.] Aragon Gelsa 8 km NE, 240 m N41.46004 W0.37642 19.5.2016 J. Tabell leg.” “DNA Barcode TLMF Lep 21279” (ZMUC). **Paratypes**. Spain. 1 ♂, prov. Alicante, Parcent, 450 m, 27.vi.2015, leg. H. Rietz; 1 ♂, prov. Alicante, Sierra de Crevillente, 400 m, 5.vi.2016, leg. H. Roweck & N. Savenkov (all ECKU); 1 ♂, prov. Castellon, 5 km E Cuevas de Vinroma, 200 m, 13.vii.1992, leg. M. Fibiger; 1 ♂, prov. Cuenca, Uña, 1150 m, 8–9.vii.2002, leg. B. Skule; 1 ♂, prov. Huesca, 6 km S Candasnos, Barranco de Valcuerna, 250 m, 19.vi.1998, leg. P. Skou, genitalia slide 3354 Hendriksen (all ZMUC); 10 ♂, prov. Huesca, 6 km SW Candasnos, Barranco de Valcuerna, 300 m, 15.vi.1999, leg. P. Skou, genitalia slide GU 15/1393 Huemer (TLMF, ZMUC); 1 ♂, prov. Cuenca, Cuenca, 29.vi.1982, leg. C. Gielis (RMNH); 1 ♂, prov. Cuenca, Uña, 1300 m, 12.ix.2007, leg. J. Viehmann; 1 ♂, prov. Huesca, Ontiñena, 300 m, 28.v.2015, leg. J. Viehmann; 1 ♂, prov. Huesca, 10 km SW Candasnos, 31.v.2016, leg. J. Viehmann (all RCWS); 5 ♂, same data, but 30.v.2015, leg. J. Viehmann (RCWS, ZMUC); 2 ♂, prov. Huesca, 10 km S Benabarre, Esteña, 800 m, 18.vi.1999, leg. P. Skou; 1 ♂, prov. Lleida, Artensa de Segre, Rubió, 14.vi.1989, leg. B. Å. Bengtsson, genitalia slide 3265 Bengtsson (RCBB); 1 ♂, prov. Lleida, 30 km NW Fraga, Ontinema, 250 m, 11.vii.1992, leg. M. Fibiger; 1 ♂, prov. Tarragona, Falset, 600 m, 19.vii.1984, leg. W. De Prins; 1 ♂, prov. Tarragona, 5 km S St. Carles de Ràpita, 20 m, 14.vi.1999, leg. P. Skou; 1 ♂, prov. Teruel, Cosa, 10.vii.1986, leg. C. Gielis; 1 ♂, prov. Teruel, 5 km NW Montalban, 950 m, 17.vii.2003, leg. B. Skule, genitalia slide GU 15/1394 Huemer (all ZMUC); 3 ♂, prov. Teruel, Sierra de Cucalon, 5 km E Baguena, 15.vi.2004, leg. J. Junnilainen, genitalia prep. (in glycerin); 2 ♂, prov. Zaragoza, Caspe, 6.v.2005, leg. J. Junnilainen (all RCJJ); 1 ♂, prov. Zaragoza, Tosos, 400m, 28.vi.1992, leg. P. Skou & B. Skule; 1 ♂, prov. Zaragoza, 4 km N Tosos, 400m, 28.vi.1997, leg. P. Skou (ZMUC).

##### Description.

Adult. *Male* (Figs 17–18). Wingspan 9–12 mm. Segment 2 of labial palpus with scale brush of about same length as segment 3, dark brown on outer surface, white mottled with brown on inner surface, white on upper surface; segment 3 about length of segment 2, white at base, becoming black towards tip. Antennal scape without pecten; flagellum ringed black and light grey-brown. Head cream-white; thorax and tegula light brown, the latter with whitish tip. Forewing light grey-brown with scattered black scales, especially in fold and apical part; costa white from 1/3; a black dot at end of cell; some black scales along termen; fringes light grey. Hindwing light grey with concolorous fringes.

*Female*. Unknown.

*Variation*. There is slight variation in the colour of the forewing (from light greyish towards light grey-brown) and in the amount of blackish scales. Some specimens have an indistinct black dot in the fold.

*Male genitalia* (Figs 157–158). Uncus moderately slender, 1.5 times as long as maximum basal width, sub-ovate, gradually tapered to weakly rounded apex; gnathos hook stout, slightly longer than uncus, straight, with pointed apex; anterior margin of tegumen with broadly rounded emargination, short ridges from anterior edge converging towards middle; pedunculi small, with additional sclerite; valva, straight, moderately stout, extending to about middle of uncus, apex weakly contorted, rounded, saccular area densely covered with setae, without separated sacculus; posterior margin of vinculum with shallow emargination, indistinct lateral humps, vincular sclerites broadly sub-rectangular, tapered towards valva, with sclerotised posterior edge; saccus moderately short and broad, almost U-shaped, with abruptly tapered and pointed apical fifth, ratio maximum width to length about 1, posterior margin strongly bulged, with rounded mediolateral projections, separated by moderately shallow incision, medial part with sclerotised ridge from posterior edge to first third, lateral sclerites about two-thirds to full length of maximum width of saccus; phallus slightly shorter than tegumen, weakly curved medially, with bulbous coecum, sclerotised band in anterior part of coecum, distal two-thirds slender, with weakly rod-like sclerotisation ventrally, subapical area with small sclerite, with or without minute tooth, ductus ejaculatorius with linear interior sclerotisation.

*Female genitalia*. Unknown.

##### Diagnosis.

*Megacraspedusbengtssoni* sp. n. is characterised by its relatively small size and by its light greyish brown forewings with a single black dot at the end of the cell. It is similar to *M.lanceolellus*, but that species is larger, it has shorter segment 3 of the labial palps and the costa is white from base. See also *M.dejectella* (p 85). The male genitalia are somewhat similar to *M.lanceolellus* (Figs 153–156) but differ in several characters such as the shorter uncus, the medially less emarginated anterior margin of tegumen, the shorter valva, and the broader saccus with a shorter medial ridge.

##### Molecular data.

BIN: BOLD:ACM1097 (n = 2). The intraspecific divergence of the barcode region has a maximum divergence of 0.2%. The distance to the nearest congeneric neighbour *M.lanceolellus* is 12% (p-dist).

##### Distribution.

Spain.

##### Biology.

Host plant and early stages are unknown. The adults have been collected from the middle of May to the middle of July at altitudes up to 1150 m.

##### Etymology.

The species name, a noun in the genitive case, is dedicated to Bengt Å. Bengtsson, Sweden, who collected part of the type series of this species and other valuable *Megacraspedus* specimens used for our study.

### *Megacraspedushomochroa* species group

The *M.homochroa* species group includes one species: *M.homochroa*.

External morphology. See species description.

Genitalia morphology. Male genitalia. See species description.

Diagnosticremarks. The *M.homochroa* species group is defined by unique structures in the male genitalia. Particularly the long and slender saccus with a long and forked medial ridge and the sub-apical spine and larger sub-apical tooth of the phallus are diagnostic. Furthermore the broad valva is rarely present in other species groups.

#### Megacraspedus homochroa

Taxon classificationAnimaliaLepidopteraGelechiidae

Le Cerf, 1932

Megacraspedus homochroa Le Cerf, 1932: 165.
Megacraspedus culminicola Le Cerf, 1932: 165, syn. n.

##### Examined material.

Morocco. 2 ♂, Prov. Al Haouz, Oukaïmeden 1.5 km SE, 2660 m N31.19340 W7.85311, 2.vii.2016, leg. J. Tabell, DNA Barcode TLMF Lep 21271, genitalia slide GU 16/1453 Huemer (ZMUC); 7 ♂, same data, but 2400 m, 7–17.vi.1965, leg. Y. de Lajonquière (SMNK, ZMUC); 9 ♂, Middle Atlas, Ifrane, 5.–10.vii.1972, genitalia slide GU 5264 Stübner, leg. F. Hahn (SMNK, TLMF, ZSM).

##### Redscription.

**Figures 19–24.**
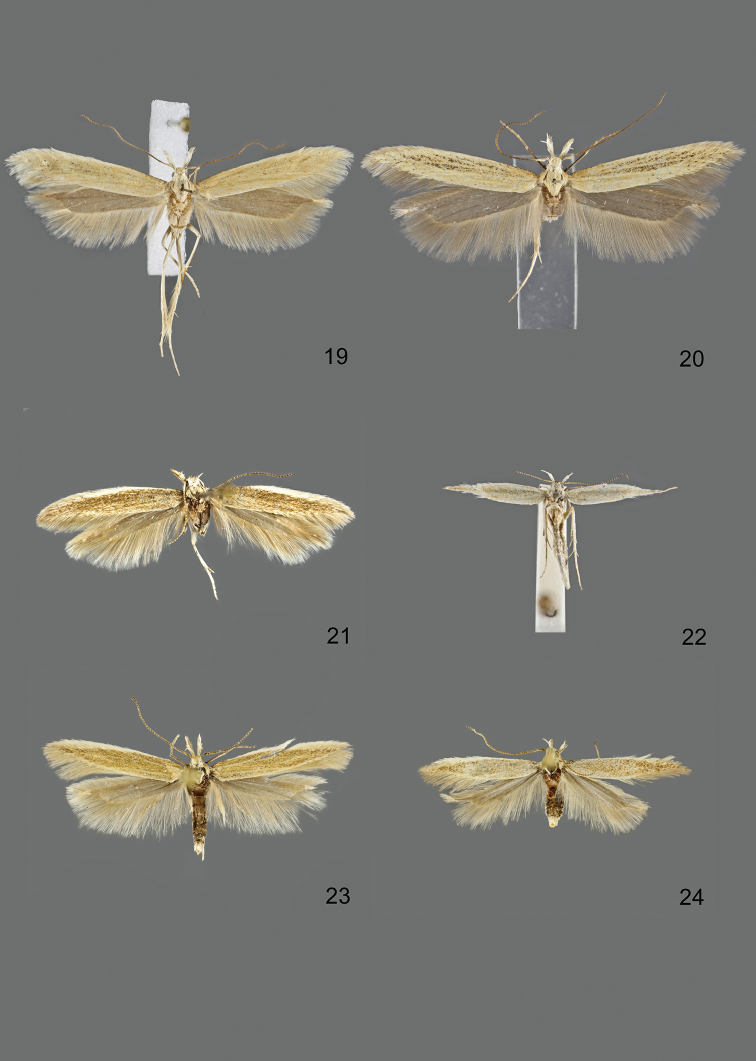
*Megacraspedus* adults in dorsal view. **19***M.homochroa* Le Cerf, 1932 – male, Morocco (ZSM) **20***M.homochroa* Le Cerf, 1932 – male, Morocco (ZMUC) **21***M.monolorellus* Rebel, 1905 – Lectotype, male (NHMW) **22***M.monolorellus* Rebel, 1905 – female, Turkey (RCEA) **23***M.junnilaineni* sp. n. – Paratype, male (TLMF) **24***M.uzunsyrtus* Bidzilya & Budashkin, 2015 – Paratype, male (ZMKU).

Adult. *Male* (Figs 19–20). Wingspan 18–22 mm. Segment 2 of labial palpus with scale brush, light brown, on outer surface mottled with black-tipped scales, white on upper surface; segment 3 thin, longer than scale brush of segment 2, whitish brown mottled with black towards tip. Antennal scape without pecten; flagellum ringed with light brown and black. Head whitish brown; thorax and tegula light brown. Forewing light yellow-brown, mottled with scattered black scales, especially at base of costa and in apical area; veins indistinctly lighter; fringes light grey. Hindwing grey with grey fringes.

*Female* (based on original description). Wingspan 11–16.5 mm. Forewing lanceolate, shorter than the body, hindwing narrow; both pairs almost entirely reddish at the base.

*Variation*. A rather variable species as regards the amount of blackish brown scales on the forewings. Specimens with many such scales look greyish yellow. Material from Middle Atlas is rather reddish-brown than yellow-brown.

*Male genitalia* (Figure 159). Uncus large, approximately 1.3 times longer than wide, elongated suboval with evenly rounded apex; gnathos hook moderately slender, slightly longer than uncus, hardly curved with pointed apex; anterior margin of tegumen with deep V-shaped emargination, sclerotised edge merged mediad in sub-anterior part of tegumen; pedunculi distinct, suboval; valva massive, stout, thumb-shaped, extending slightly beyond base of uncus, apical part weakly divergent, rounded; saccular area densely covered with setae, without separated sacculus; posterior margin of vinculum with deep U-shaped emargination, weakly rounded lateral humps, suboval vincular sclerite with strongly sclerotised posterior edge; saccus long, sub-triangular, evenly tapered to pointed apex, ratio maximum width to length approximately 0.6, posterior margin with rounded mediolateral projections, separated by moderately broad and shallow incision, medial part with strongly sclerotised longitudinal ridge, forked in medial part of saccus and extending to anteriolateral edge, lateral sclerites about length of maximum width of saccus; phallus with weakly bulbous coecum, distal two-thirds stout, straight, medial ridge with small sub-apical spine, ventral edge with larger sub-apical tooth, both pointing ventrad, apex rounded, ductus ejucalatorius with contorted linear interior sclerotisation.

*Female genitalia*. Undescribed.

##### Diagnosis.

*Megacraspedushomochroa* is characterised by its rather large size and its light yellow-brown to reddish-brown forewings without spots. See also *M.tenuignathos* sp. n. (p 126). The male genitalia are unmistakable due to the combined shape of the large uncus and saccus, and the peculiar structures of the phallus.

##### Molecular data.

BIN BOLD:ADF2257 (n = 1). The distance to the nearest neighbour *M.golestanicus* sp. n. is 9.1% (p-dist).

##### Distribution.

Morocco (Middle Atlas, High Atlas).

##### Biology.

Host plant and early stages are unknown. The type-series of *M.homochroa* was collected in the Middle Atlas in August at an altitude of 2400 m, that of *M.culminicola* at high altitude of 3200 m in the middle of July. Additional samples date from about mid-June to early July and at altitudes from ca. 2400 to 2660 m.

##### Remarks.

*Megacraspedushomochroa* was described from two males collected in Morocco, Middle Atlas (Le Cerf 1932). The type-material could not be traced at MNHN, but the description of the adults leaves no doubt as to the identity of this species.

*Megacraspedusculminicola* was described from two males and two females taken in copula in Morocco, Middle Atlas (Le Cerf 1932). Like *M.homochroa*, the type-material could not be traced at MNHN, but descriptions of the males of both species agree in almost every detail and the type-localities are nearby. We therefore consider *M.culminicola* to be a junior synonym of *M.homochroa* (syn. n.).

### *Megacraspedusdolosellus* species group

The *M.dolosellus* species group includes seven species: *M.monolorellus*, *M.junnilaineni* sp. n., *M.uzunsyrtus*, *M.similellus* sp. n., *M.golestanicus* sp. n., *M.tokari* sp. n., and *M.dolosellus*.

External morphology. Segment 2 of labial palpus with scale brush shorter or as long as segment 3; segment 3 about length of segment 2. Antennal scape without or with one or a few hairs. Wingspan (males) 10–16 mm. Forewing with white costa, and in some species with white veins, but no black dots. Known females distinctly to strongly brachypterous.

Genitalia morphology. Male genitalia. Uncus moderately slender, sub-triangular; gnathos hook long, slender, slightly longer than uncus; anterior margin of tegumen deeply emarginated; valva straight, moderately slender; saccular area setose, without separated sacculus; saccus nearly V-shaped, short, ratio maximum width to length approximately 0.8, posterior margin with distinct projection, distally abruptly tapered, without or with short, partially furcated medial ridge; phallus moderately stout, with globular coecum, distal portion weakly to distinctly S-curved, with broad dorsal and long and slender ventral lobes, apically tapered.

Female genitalia. Papilla analis small; apophysis posterior is very long; segment VIII long and slender, largely membranous; subgenital plate with sub-triangular subostial sclerotisation, anteromedially with short projection; apophysis anterior is rod-like, from posterior margin of segment VIII; colliculum short; signum small, rounded spiny plate.

Diagnosticremarks. The *M.dolosellus* species group is particularly defined by the usually distinctly S-curved phallus in combination with several other characters of the male genitalia such as the long and slender gnathos hook, the moderately slender valva, and the broadly sub-triangular saccus with or without a short medial ridge.

Taxa of the *M.dolosellus* species group are difficult to separate by phenotypic appearance, with only subtle differences in the forewing colour and pattern. Similarly diagnostic characters of male genitalia are cryptic in some species with only subtle differences.

#### Megacraspedus monolorellus

Taxon classificationAnimaliaLepidopteraGelechiidae

Rebel, 1905

Megacraspedus monolorellus Rebel, 1905: 213.

##### Examined material.

**Lectotype** ♂, **here designated**, “Asia min. Penther ’02 Ilgin.” “*Megacraspedus monolorellus* Type Rbl.” “BC TLMF Lep 06704” “NM 16.649 ♂” (NHMW). **Paralectotypes**. 1 ♂, 1 ♀, [in copula] “Ilgün ´02 Penther” “*Megacrasp Monolorellus* Rbl Type” (NHMW). Turkey. 1 ♂, Silifke, Mut, 400 m, 31.v.1996, leg. F. Schepler, genitalia slide 5338 Karsholt (ZMUC); 1 ♂, 1 ♀, 30 km NE Konya, 27.v.1969, leg. E. Arenberger (RCEA); 16 ♂, prov. Konya, 25 km E Konya, 23.v.1997, leg. K. Nupponen & J. Junnilainen (RCKN).

##### Redescription.

Adult. Male (Figure 21). Wingspan 14–16 mm. Segment 2 of labial palpus with scale brush of same length as segment 3, brown on outer surface, whitish brown on inner surface, otherwise white; segment 3 about same length as segment 2, white mottled with light brown. Antennal scape with pecten of one hair; flagellum black, indistinctly ringed with grey. Head, thorax and tegula whitish brown. Forewing brown, slightly mottled with white scales; a distinct white stripe along costa, broadest in middle; base of costa blackish; fringes light grey. Hindwing grey with light grey fringes.

Female (Figure 22). Wingspan 11 mm. Labial palpus cream-white, segment 2 finely mottled with brown. Antennal scape with pecten of one hair; flagellum blackish brown, ringed with white. Forewing broadest in middle, apical half tapering into lanceolate, blackish tip. Hindwing very short (about one-fifth length of forewing), slender with rounded apex, whitish. Abdomen as long as forewing, whitish brown, becoming white at apical segments. Ovipositor long, protruding. Otherwise similar to male.

Variation. The examined specimens show only slight variation.

Male genitalia (Figure 160). Uncus moderately broad, approximately 1.6 times longer than maximum basal width, weakly tapered from base to distal third, apically constricted with rounded apex; gnathos hook moderately stout, about length of uncus, weakly curved, narrowing towards pointed apex; anterior margin of tegumen with moderately deep and broadly V-shaped emargination, medial ridge from anterior edge to middle, anteriolateral edge with small peg-like sclerite; pedunculi distinct, irregular shape; valva evenly broad, short, stout, about maximum width of uncus, apex rounded, extending slightly beyond middle of uncus; saccular area covered with numerous setae, without separated sacculus; posterior margin of vinculum with moderately deep medial emargination, lateral humps weakly developed, vincular sclerite oblong, with broadly sclerotised posteriomedial edges; saccus nearly V-shaped, apical half strongly narrowing, with pointed apex, ratio maximum width to length approximately 0.8, posterior margin arched, with small medial incision, medial part with short, furcated ridge from posterior edge almost extended to medial part, lateral sclerites nearly length of maximum width of saccus; phallus moderately stout, with globular coecum, distal portion nearly straight, with broad dorsal and slender ventral lobes, apically tapered.

Female genitalia (Figure 269). Papilla analis small, apically rounded; apophysis posterior slender rod-like, approximately 3.6 mm long, with short, bifurcate posterior end, bordered by minute sclerotised field, apex weakly widened and rounded; segment VIII approximately 1.8 mm long, membranous; subgenital plate with sub-triangular subostial sclerotisation, posteriorly extended into moderately long, pointed sub-medial sclerites, with weak medial flaps delimiting oblong ostium bursae, anterior margin with rod-like edge connected with apophysis anterior, medially membranous, without anterior projection; apophysis anterior slender, rod-like, 2 mm length, posteriorly becoming rod-like venula of segment VIII, extending to posterior margin of segment VIII; colliculum short, sclerotised; ductus bursae and corpus bursae weakly delimited, moderately short with entire length of approximately 3.1 mm; signum moderately large, suboval spiny plate.

##### Diagnosis.

*Megacraspedusmonolorellus* is characterised by its brown forewings with white costa and without further markings. It is similar to *M.uzunsyrtus* (p 45) and *M.similellus* sp. n. (p 46). The male genitalia are very similar to *M.junnilaineni* sp. n. (Figure 161) but differ particularly by the broader uncus and gnathos hook, and the shorter and broader valva. From other related species such as *M.uzunsyrtus* (Figure 162) and *M.similellus* sp. n. (Figs 163–164) they can be separated e.g., by the gradually tapered uncus. The female genitalia differ from *M.similellus* sp. n. (Figure 270) and all other species of *Megacraspedus* with known females in particular by the extraordinarily long apophysis anterior.

##### Molecular data.

BIN BOLD:ACA8694 (n = 1). The distance to the nearest neighbour *M.golestanicus* sp. n. is 9.2% (p-dist).

##### Distribution.

Turkey.

##### Biology.

Host plant and early stages are unknown. The type series was collected in the first third of May, and also the few additional specimens examined were found in May.

##### Remarks.

*Megacraspedusmonolorellus* was described from several specimens of both sexes collected on 10.v.1902 near Ilgin (“Ilgün”), central Turkey (Rebel 1905: 213). An especially large male mentioned by Rebel is here designated as the lectotype, in order to stabilize nomenclature, and the above description is in first hand based on that well preserved specimen.

#### Megacraspedus junnilaineni sp. n.

Taxon classificationAnimaliaLepidopteraGelechiidae

http://zoobank.org/CDA564BB-C420-4643-837F-FC3BAA104E5D

##### Examined material.

**Holotype** ♂, “Turkey [prov. Nevşehir] Urgup [Ürgüp] 7 km E 18.5.2005 J. Junnilainen leg.” “DNA Barcode TLMF Lep 19975” “GU 16/1448 ♂ P. Huemer” (RCJJ). **Paratypes**. Turkey. 1 ♂, same data as holotype (TLMF); 1 ♂, Nevşehir, 10 km V Ürgüp, Göreme, ‘Love Valley’, 1300 m, 2.vii.1997, leg. M. Fibiger, genitalia slide 5325 Karsholt (ZMUC); 1 ♂, prov. Istanbul, 5 km NE Aksaray, 19.v.2005, leg. J. Junnilainen (TLMF).

##### Description.

Adult. Male (Figure 23). Wingspan 12–13 mm. Segment 2 of labial palpus with scale brush almost as long as segment 3, brown on outer surface, whitish brown on inner surface, otherwise white; segment 3 about same length as segment 2, white, darker on lower surface and towards tip. Antennal scape with pecten of one hair; flagellum black, indistinctly ringed with grey. Head, thorax and tegula whitish grey-brown. Forewing light greyish brown from brownish white and black-tipped scales; base of costa blackish brown, otherwise whitish; fringes light grey. Hindwing grey with light grey fringes.

Female. Unknown.

Variation. The examined specimens show only slight variation.

Male genitalia (Figure 161). Uncus basally very broad, slightly longer than maximum basal width, gradually and weakly tapered from base to broadly rounded apex; gnathos hook slender, about one-third longer than uncus, weakly curved, narrowing towards pointed apex; anterior margin of tegumen with moderately deep and broad emargination, medial ridge from anterior edge to middle, anteriolateral edge with small peg-like sclerite; pedunculi distinct, irregular shape; valva moderately slender, long, maximum two-thirds width of uncus, gradually tapered to slightly pointed apex, extending slightly beyond middle of uncus; saccular area covered with numerous setae, without separated sacculus; posterior margin of vinculum with shallow medial emargination, lateral humps weakly developed, vincular sclerite sub-rectangular, with broadly sclerotised posteriomedial edges; saccus nearly V-shaped, apical third strongly narrowing, with pointed apex, ratio maximum width to length nearly 1, posterior margin arched, with small medial incision, medial part with short, furcated ridge from posterior edge almost extended to medial part, lateral sclerites about three-quarters length of maximum width of saccus; phallus moderately stout, with globular coecum, distal portion S-curved, with broad dorsal and long and slender ventral lobes, apically tapered.

Female genitalia. Unknown.

##### Diagnosis.

*Megacraspedusjunnilaineni* sp. n. is characterised by its light greyish brown forewings without markings. Externally it is hardly separable from *M.golestanicus* sp. n. (p 48). The male genitalia are very similar to *M.monolorellus* (Figure 160) but differ in particular by the more slender uncus and gnathos hook, and the longer and comparatively slender valva. From other related species such as *M.uzunsyrtus* (Figure 162) and *M.similellus* sp. n. (Figs 163–164) they can be separated e.g., by the gradually tapered uncus.

##### Molecular data.

BIN BOLD:ADB7272 (n = 1). The distance to the nearest neighbour *M.similellus* sp. n. is 9.4% (p-dist).

##### Distribution.

Turkey.

##### Biology.

Host plant and early stages are unknown. The adults have been collected from the middle of May to the middle of August at altitudes of between 1200 and 1300 m.

##### Etymology.

The species name (a noun in the genitive case) is dedicated to Jari Junnilainen, Finland, who collected part of the type series of this species and numerous other valuable specimens used for our study.

#### Megacraspedus uzunsyrtus

Taxon classificationAnimaliaLepidopteraGelechiidae

Bidzilya & Budashkin, 2015

Megacraspedus uzunsyrtus Bidzilya & Budashkin, 2015: 222, figs 19–22.

##### Examined material.

**Paratypes**. Ukraine. 2 ♂, Crimea, Koktebel vic., SW slope of Uzun-Syrt, evening collecting, 17.v.2014, leg. Yu. Budashkin, genitalia slide GU 16/1463 Huemer (ZMKU).

Ukraine. 2 ♂, Crimea, Aj-Petri, 5–6.vii.2002, leg, Yu. Budashkin (ZMKU).

##### Redescription.

Adult. Male (Figure 24). Wingspan 12–14 mm. Segment 2 of labial palpus with moderately long scale brush, brown on outer surface, whitish brown on inner surface, otherwise white; segment 3 white, darker on lower surface and towards tip. Antennal scape with pecten of one hair; flagellum light grey-brown ringed with black. Head, thorax and tegula as forewing. Forewing light greyish brown from white brownish- and black-tipped scales, darkest towards apex; a distinct white stripe along costa; base of costa blackish; fringes light grey. Hindwing grey with light grey fringes.

Female. Unknown.

Variation. The examined specimens show only slight variation.

Male genitalia (Figure 162). Uncus moderately slender, slightly more than twice as long as broad, parallel-sided, apical 1/3 weakly narrowed, apex rounded; gnathos hook stout, about length of uncus, bent near middle, apex curved, pointed; anterior margin of tegumen with moderately deep and broadly rounded emargination, long medial ridge from anterior edge to posterior third, anteriolateral edge with small peg-like sclerite; pedunculi distinct, suboval; valva broad, about one-third width of uncus, straight, of equal width, apex rounded, extending to middle of uncus, sub-basally with rounded hump; saccular area covered with setae, without separated sacculus; posterior margin of vinculum with shallow medial emargination, lateral humps weakly developed, basally broad vincular sclerite tapered posteriorly, with broadly sclerotised posteriomedial edges; saccus nearly V-shaped, with pointed, rod-like apical third, ratio maximum width to length approximately 0.8, posterior margin weakly arched, with small medial incision, medial part with short furcated ridge from posterior edge, lateral sclerites approximately 0.8 times length of maximum width of saccus; phallus with globular coecum, distal portion weakly S-curved, few minute teeth on dorsal edge, tapered apically.

Female genitalia. Unknown.

##### Diagnosis.

*Megacraspedusuzunsyrtus* is characterised by its light greyish brown forewings with a white costa and without further markings. It is very similar to *M.monolorellus* (Figure 21) which has more brownish forewings. *M.similellus* sp. n. (p 46). The male genitalia are particularly similar to *M.similellus* sp. n. (Figs 163–164) but differ by the abruptly bent gnathos hook, and the broader valva without distal constriction. From other related species they differ by several subtle characters, particularly the shape of the uncus with parallel-sided outer edges.

##### Molecular data.

BIN BOLD:ACS7353 (n = 2). The intraspecific divergence of the barcode region is 0%. The distance to the nearest neighbour *M.similellus* sp. n. is 6.4% (p-dist).

##### Distribution.

Ukraine (Crimea).

##### Biology.

Host plant and early stages are unknown. Adults have been collected in the middle of May in the evening before sunset on xerothermic slopes on *Jurineastoechadifolia* (M. Bieb.) DC (Asteraceae) which is considered to be the larval host plant (Bidzilya and Budashkin 2015). Additional specimens were found in early July.

##### Remarks.

*M.uzunsyrtus* was described from four males collected in Crimea on 17.v.2014 (Bidzilya and Budashkin 2015: 222). Although these authors state that “*Jurinea stoechadifolia* … is undoubtedly the larval host plant” this needs to be confirmed by breeding as other host plant records of *Megacraspedus* species are from Poaceae. Bidzilya and Budashkin (op. cit.) stated that *M.uzunsyrtus* can be separated from *M.monolorellus* by having an antennal pecten of one hair, which should not be present in the latter species. However, examination of the lectotype of *M.monolorellus* shows that a pecten is present at the base of the left antenna.

#### Megacraspedus similellus sp. n.

Taxon classificationAnimaliaLepidopteraGelechiidae

http://zoobank.org52EBF73B-8260-4211-87BE-A42F810A2868

##### Examined material.

**Holotype** ♂, “BULGARIA [Dobrich region] Tuzlata 3.6.2014 J.Junnilainen leg.” “GU 16/1449 ♂ P. Huemer” “DNA Barcode TLMF Lep 19950” (RCJJ). **Paratypes**. Bulgaria. 1 ♀, same data as holotype, but genitalia slide GU 16/1471 Huemer; 1 ♂, same data, but 28.v.2002, leg. J. Junnilainen; 1 ♂, same data, but 5.vi.2002, leg. J. Junnilainen (all RCJJ); 1 ♂, Dobrich reg., Kaverna, Chirakman, 21.v.2010, leg. B. S. Larsen (ZMUC). Romania. 1 ♂, Dobrogea, Jurilovca Capul, Dolosman, 16.vi.2012, leg. S. & Z. Kovacs, genitalia slide GU 16/1470 Huemer (RCKO). Turkey, 2 ♂, prov. Nigde, Bolkar Dağlari, northern slope, Maden, 1700 m, 29.vii.1997, leg. K. Larsen, genitalia slide 5326, 5345 Karsholt (ZMUC); 1 ♂, prov. Isparta, 35 km SW Askehir, Çetince, 1200 m, 14.viii.1999, leg. J. Junnilainen, genitalia slide GU 16/1462 Huemer (RCJJ); 1 ♂, 25 km E Konya, 23.v.1997, leg. K. Nupponen & J. Junnilainen; 1 ♂, 5 km N Urgüp, 25.v.1997, leg. K. Nupponen & J. Junnilainen; 1 ♂, 30 km SW Kayseri, Erciyes Dagi, 24.v.1997, leg. K. Nupponen & J. Junnilainen (all RCJJ).

##### Description.

**Figures 25–30.**
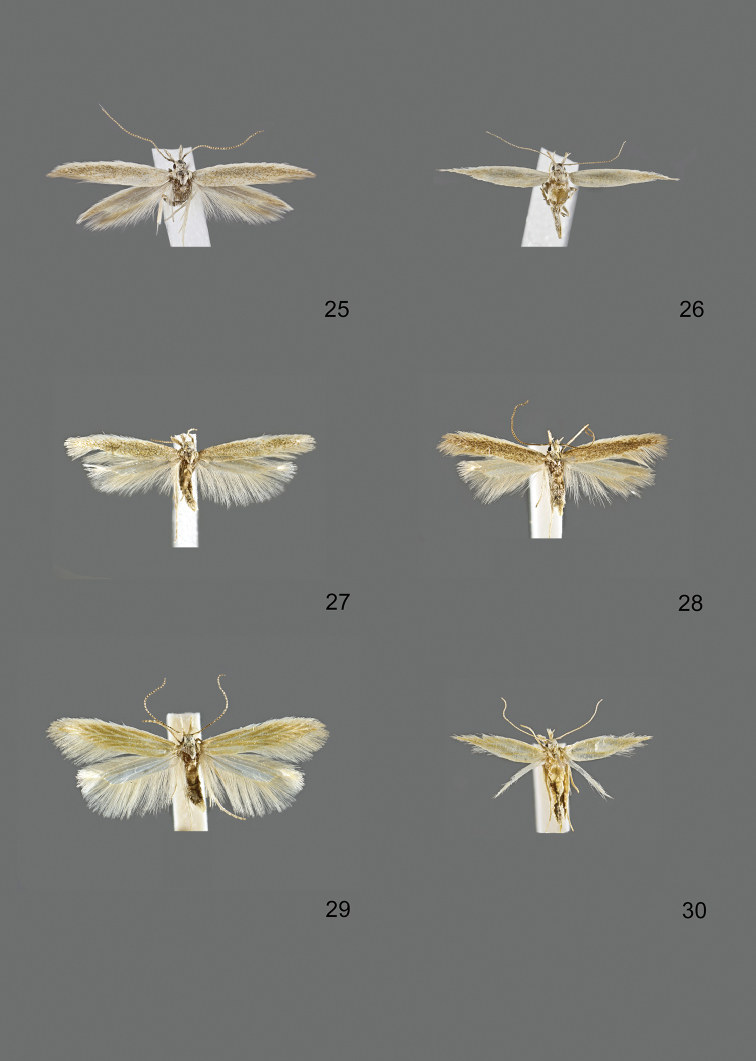
*Megacraspedus* adults in dorsal view. **25***M.similellus* sp. n. – Holotype, male (RCJJ) **26***M.similellus* sp. n. – Paratype, female (RCJJ) **27***M.golestanicus* sp. n. – Paratype, male (TLMF) **28***M.tokari* sp. n. – Holotype, male, Croatia (RCZT) **29***M.dolosellus* (Zeller, 1839) – male, Slovenia (RCZT) **30***M.dolosellus* (Zeller, 1839) – female (form *dolosellus*), Slovenia (RCZT).

Adult. *Male* (Figure 25). Wingspan 13–14 mm. Segment 2 of labial palpus with scale brush as long as segment 3, brown on outer surface, whitish brown on inner surface, otherwise white; segment 3 about same length as segment 2, dark brown. Antennal scape with pecten of one hair; flagellum black ringed with light brown. Head whitish grey; thorax as forewing; neck and tegula white. Forewing light grey brown from white brownish- and black-tipped scales, darkest towards apex; costa white; fringes light grey. Hindwing grey with light grey fringes.

*Female* (Figure 26). Wingspan 11 mm. Segment 3 of labial palpus white mottled with dark brown. Forewing broadest in middle, apical half tapering into lanceolate, blackish tip. Hindwing very short (about one-fifth length of forewing), slender with rounded apex, whitish. Otherwise similar to male.

*Variation*. There is some variation in the colour of the forewings: from lighter to darker brown.

*Male genitalia* (Figure 163–164). Uncus moderately slender, slightly more than twice as long as broad, parallel-sided, apical 1/3 weakly tapered, apex rounded; gnathos hook stout, about length of uncus, weakly and evenly curved, apex pointed; anterior margin of tegumen with moderately deep and broadly rounded emargination, long medial ridge from anterior edge to posterior third, anteriolateral edge with small peg-like sclerite; pedunculi distinct, suboval; valva moderately broad, basally slightly exceeding width of uncus, distal part narrowing, straight, apex rounded, extending to middle of uncus, sub-basally with rounded hump; saccular area covered with setae, without separated sacculus; posterior margin of vinculum with shallow medial emargination, lateral humps weakly developed, vincular sclerite broadly sub-rectangular, with broadly sclerotised posteriomedial edges; saccus nearly V-shaped, apical third evenly pointed, ratio maximum width to length approximately 0.8, posterior margin weakly arched, with small medial incision, medial part with short, furcated ridge from posterior edge, lateral sclerites approximately 0.8 times length of maximum width of saccus; phallus moderately stout, with globular coecum, distal portion weakly S-curved, tapered apically.

*Female genitalia* (Figure 270). Papilla analis small, apically rounded; apophysis posterior slender rod-like, about 4 mm long, with short, bifurcate posterior end, bordered by minute sclerotised field, apex weakly widened and rounded; segment VIII approximately 1.4 mm long, membranous; subgenital plate with sub-triangular subostial sclerotisation, posteriorly extended into long, pointed sub-medial sclerites, with medial flaps delimiting oblong ostium bursae, anterior margin with rod-like edge connected with apophysis anterior, straight, without anterior projection; apophysis anterior slender, rod-like, posteriorly becoming rod-like venula of segment VIII, extending to posterior margin of segment VIII; colliculum short, sclerotised; ductus bursae and corpus bursae weakly delimited, moderately short with entire length of approximately 2.5 mm; signum moderately large, suboval spiny plate.

##### Diagnosis.

*Megacraspedussimilellus* sp. n. is characterised by its light greyish brown forewings with white costa and without further markings. Externally it is hardly separable from *M.uzunsyrtus* (Figure 24). It is also similar to *M.monolorellus* (Figs 21–22) which has more brownish forewings. The female is hardly separable from that of *M.monolorellus*. The male genitalia are very similar to *M.uzunsyrtus* (Figure 162) but differ by the evenly curved gnathos hook, and particularly the more slender and distally narrowing valva. From other related species they differ in several subtle characters, particularly the shape of the uncus with parallel-sided outer edges. The female genitalia differ from *M.monolorellus* (Figure 269) particularly by the distinctly shorter apophysis anterior, but females of several related species are unknown.

##### Molecular data.

BIN BOLD:ADB8685 (n = 3). The intraspecific divergence of the barcode region is moderate with mean 0.8% and maximum divergence of 1.2%. The distance to the nearest neighbour *M.uzunsyrtus* is 6.4% (p-dist).

##### Distribution.

Bulgaria, Romania, Turkey.

##### Biology.

Host plant and early stages are unknown. The adults have been collected from the middle of May to late July from sea level in the Balkans to 1700 m in Turkey.

##### Etymology.

The species name refers to the similarity to related taxa and is derived from the Latin word *similis* (meaning like), and the diminutive suffix –*ellus*. The name is a compound noun.

#### Megacraspedus golestanicus sp. n.

Taxon classificationAnimaliaLepidopteraGelechiidae

http://zoobank.org/5ED8551E-F59E-45C0-B539-CFDA1732ABCF

##### Examined material.

**Holotype** ♂, “Iran, [prov.] Khorassan NP [National Park] Golestan, Almeh 1770 m 2.5.2001 37°20,75'N, 56°07,02'E leg. P. Huemer” “BC TLMF Lep 03782” “P. Huemer GEL 1241 ♂” (TLMF). **Paratypes**. Iran. 4 ♂, same data as holotype, but BC TLMF Lep 03780, 03781 (TLMF).

##### Redescription.

Adult. *Male* (Figure 27). Wingspan 10–11 mm. Segment 2 of labial palpus with moderately long scale brush, brown on outer surface, whitish brown on inner surface, otherwise white; segment 3 white mottled with light grey-brown. Antennal scape with pecten of one hair; flagellum light brown, indistinctly ringed with black. Head, thorax and tegula as forewing. Forewing light greyish brown from white brownish- and black-tipped scales; base of costa blackish brown; margin of costa whitish; fringes light grey. Hindwing grey with light grey fringes.

*Female*. Unknown.

*Variation*. The few examined specimens show only slight variation. The margin of the forewing costa can be greyish or whitish. In one specimen there is no hair at the base of the antennal scape.

*Male genitalia* (Figure 165). Uncus approximately 1.5 times longer than broad, parallel-sided, apex rounded; gnathos slightly longer than uncus, moderately slender, curved, apex pointed; tegumen weakly constricted, anterior margin deeply excavated, pedunculi small, rounded; valva short, straight, of equal width, apex slightly distorted, rounded, extending to base of uncus; posterior margin of vinculum with broad lobes, anteromedial emargination broad, shallow; saccus broad at base, with long and narrow lateral projections, anterior edge with sinus-shaped projections, incised in-between, medial part with short sclerotised ridge, distal portion abruptly tapered, rod-like; phallus about length of tegumen without uncus, globular coecum, distal two-thirds weakly S-curved, tapered apically.

*Female genitalia*. Unknown.

##### Diagnosis.

*Megacraspedusgolestanicus* sp. n. is characterised by its relatively small wingspan, and its light greyish brown forewings without markings. Externally it is hardly separable from *M.junnilaineni* sp. n. (Figure 23). The short medial ridge of the saccus and the abruptly tapered rod-like distal part are characteristic features of *M.golestanicus* sp. n.

##### Molecular data.

BIN BOLD:AAU3338 (n = 3). The intraspecific divergence of the barcode region is low with mean 0.1% and maximum divergence of 0.2%. The distance to the nearest neighbour *M.uzunsyrtus* is 8.8% (p-dist).

##### Distribution.

Iran (Golestan).

##### Biology.

Host plant and early stages are unknown. The habitat is dominated by steppe meadows. The adults have been collected in the last third of May at light at an altitude of 1770 m.

##### Etymology.

This species is named after its place of occurrence: the province of Golestan in north-eastern Iran. The name is a masculine adjective.

#### Megacraspedus tokari sp. n.

Taxon classificationAnimaliaLepidopteraGelechiidae

http://zoobank.org/D230F7C8-64C2-46C3-8DBE-DE674C91BE0D

##### Examined material.

**Holotype** ♂, “CROATIA [Dalmatia region] Konjevrate 200 m 25.06.2006 leg. Z. Tokár” “*Megacraspedus* sp. det. Zdenko Tokár” “DNA Barcode TLMF Lep 16630” (RCZT). **Paratypes**. Croatia. 2 ♂, same data as holotype; 1 ♂, same data, but 25.vi.2003; 8 ♂, same data, but 28.vi.2003; 2 ♂, same data, but 6.vi.2005; 1 ♂, Gorne Bilišane, 6.vii.2004, leg. Z. Tokár; 1 ♂, Bilišane, 23.vi.2006, leg. Z. Tokár; 1 ♂, same data, but 15.ix.2007, genitalia slide 14/1384 Huemer (all RCZT); 2 ♂, Krk isl. Str. Krk-Vrbnik, 20.vii.1988, leg. G. Baldizzone (TLMF); 1 ♂, Krk isl., Punat-Stara Bavka, Trstenova, 18.vii.2013, leg. G. Baldizzone (RCGB); 2 ♂, Krk isl., Mt. Hiam, Branušine, 22.vi.2013, 180 m, leg. G. Baldizzone (RCGB; ZMUC).

##### Description.

Adult. *Male* (Figure 28). Wingspan 10 mm. Segment 2 of labial palpus with scale brush shorter than segment 3, brown on outer surface, whitish brown on inner surface, otherwise white; segment 3 about same length as segment 2, white. Antennal scape with pecten of a few hairs; flagellum black, indistinctly ringed with light brown. Head cream-coloured; thorax and tegula cream-coloured mottled with brownish. Forewing brown mottled with some yellow-white, especially along dorsum; a distinct white stripe along costa; fold yellowish; fringes light grey. Hindwing grey with light grey fringes.

*Female*. Unknown.

*Variation*. The examined specimens show no variation, but worn specimens become light greyish.

*Male genitalia* (Figure 166). Uncus moderately broad, 1.5 times as long as maximum basal width, sub-trapezoidal, weakly tapered to rounded apex; gnathos hook slender, straight, about one-quarter longer than uncus, with curved and pointed apex; anterior margin of tegumen with moderate, broadly rounded emargination, medially with longitudinal ridge, extending from anterior edge beyond middle of tegumen; pedunculi small, rounded, with sclerotised ridge; valva straight, slender, basally wider, apical part contorted, rounded, extending to about base of gnathos, saccular area covered with setae, with hardly separated sacculus, fused with valva; posterior margin of vinculum with shallow medial emargination, weakly rounded lateral humps, vincular sclerite suboval, posteriomedial edge strongly sclerotised; saccus moderately large, broadly V-shaped, with rod-like apical fifth, ratio maximum width to length approximately 0.9, posterior margin with broadly rounded projections, separated by V-shaped emargination, medial part of saccus with short sclerotised ridge, furcated at approximately one-third length of saccus, lateral sclerites approximately 0.8 times length of maximum width of saccus; phallus about length of tegumen, moderately stout, with bulbous coecum, distal two-thirds weakly curved, tapered apically, with rod-like ventral sclerotisation.

*Female genitalia*. Unknown.

##### Diagnosis.

*Megacraspedustokari* sp. n. is characterised by cream-coloured head and its brown forewings with a white costa and without further markings. Its small size separates it from similar looking species. The male genitalia differ from other species of the *M.dolosellus* species group particularly by the furcated ridge of the saccus and the weakly curved phallus.

##### Molecular data.

BIN BOLD:ACM1095 (n = 1). The distance to the nearest congeneric neighbour *M.cuencellus* is 10%, the distance to the nearest BIN in BOLD, *Monochroascutatella* (Müller-Rutz, 1920), is 9.2% (p-dist).

##### Distribution.

Croatia.

##### Biology.

Host plant and early stages are unknown. The adults have been collected from late June to the middle of September at low altitudes.

##### Etymology.

The species name (a noun in the genitive case) is dedicated to Zdenko Tokár, Slovakia, who collected most of the type series of this species and numerous other valuable specimens used for our study.

#### Megacraspedus dolosellus

Taxon classificationAnimaliaLepidopteraGelechiidae

(Zeller, 1839)

Ypsolophus (Megacraspedus) dolosellus Zeller, 1839: 190.
Ypsolophus separatellus Fischer von Röslerstamm, 1843: 300, 302, pl. 100, figs 1a–d [on plate *as’separatella*‘], syn. n.
Megacraspedus incertellus Rebel, 1930: (14), syn. n.

##### Examined material.

**Lectotype** ♂, *Ypsolophusdolosellus*, designated by Huemer & Karsholt (2001), “*dolosella* FR Wien” “*Ypsolophus dolosellus* Zeller Isis p 190 (1839) TYPE ♂” “Zeller Coll. Wlsm. Coll. B.M. 1910-427. ” “Lectotypus ♂ *Ypsolophus dolosellus* Select. K. Sattler, 1961” “B.M. Genitalia slide No. 7102 ♂“ ´Type” “LECTO-TYPE” (BMNH). **Lectotype** ♂, *Megacraspedusincertellus*, here designated, “LECTO-TYPE” “Alibotuschgebir Al K. Drenowski“ “*Megacraspedus incertellus* Rbl Type ♂” “LECTOTYPE ♂ *Megacraspedusincertellus* Rebel det. L.M. Pitkin, 1987” “Mus.Vind. Gen.Präp. 16.518 ♂” “BC TLMF Lep 06703” (NHMW). Albania. 2 ♂, Pashtrik, 7–15.viii.1918; 2 ♂, Gjalica Ljums, 17–26.vi.1918, genitalia slide Mus. Vind. 15.348 (NHMW). Austria. 1 ♀, without locality, leg. J. Mann (NHMW). Austria. 1 ♀ [form *dolosellus*], Wien, Prater, 1856 (NHMW); 2 ♂, Wien (ZSM); 1 ♂, Niederösterreich, Hainburg, 16.v.1909, leg. Zerny (NHMW). Bosnia and Herzegovina. 1 ♂, Trebovic, 5.vii.1898, genitalia slide Mus.Vind 15.343 ♂ (NHMW); 12 ♂, Dolovi, 10.vi.2011, leg. I. Richter; 1 ♂, same data, but 21.vi.2011 (RCIR, ZMUC); 1 ♂, Dolovi, 10.vi.2011, leg. I Richter (all NMPC). Bulgaria. 1 ♂, Jakoruda, 23.vi.2000, leg. J. Junnilainen, leg. J. Junnilainen, genitalia prep. (in glycerin); 1 ♂, Belashitsa, 2000 m, 27.vii.2013, leg. J. Junnilainen, genitalia slide 16/1465 Huemer [BOLD:ADB8789] (RCJJ); 1 ♂, 1 ♀ [form *dolosellus*], Rilo, 1600 m, 28.7.1902, genitalia slides Mus.Vind 16.523 ♂, Mus.Vind. 16.527 ♀; 2 ♂, Slivno, vi.1896, leg. H. Rebel, genitalia slide Mus.Vind 16.526 ♂ (all NHMW); 1 ♂, 5 km NNE Pastra, 31.vii.2013, leg. B. Å. Bengtsson; 1 ♂, Pirin mts, Oreliak, 1850–1950 m, 9.viii.2013, leg. O. Karsholt & B. Zlatkov (all ZMUC); 5 ♂, same data, but 1800–1950 m, 24.vi.2014, leg. Z. Tokár, genitalia slide GEL 1216 Huemer (RCZT, TLMF); 1 ♂, but 2000 m, 4.vii.2014, leg. J. Junnilainen, genitalia in gylcerin (RCJJ); 3 ♂, Pirin mts, Vichren-Kazana, 2000 m, 26–30.vii.1986, leg. J. Liška (NMPC); 12 ♂, Pirin mts, Popovi, Livadi, 2000 m, 22.vi.2001, leg. J. Junnilainen, genitalia slide 16/1468 Huemer; 1 ♂, 10 km E Ilindentci, 1200 m, 10.vii.2014, leg. J. Junnilainen (all RCJJ). Croatia. 3 ♂, 1 ♀ [form *dolosellus*], Petrinjski kras, Petrinja, 3.vi.2005, leg. Z. Tokár (RCZT); 1 ♂, Pag, 10.vi.2015, leg. J. Junnilainen [BOLD:ADB8686] (RCJJ); 6 ♂, N Velebit, Pandore, 800 m, 21.vi.2006, leg. Z. Tokár; 6 ♂, same data, but 21.vi.2006, genitalia prep. (in glycerin); 6 ♂, Biokovo mts, Vošac, 1370 m, 27.vi.2006, leg. Z. Tokár, genitalia prep. (in glycerin); 1 ♀, Malovan-Gracac, 10.vii.2004, leg. Z. Tokár (all RCZT). France. 1 ♀ [form *dolosellus*], Dep. Pyrénées-Orientales, Vernet, 1.vii.1895, leg. P. Chrétien; 1 ♂, 1 ♀ [form *dolosellus*], Dep. Paris, Seine, leg. P. Chrétien; 1 ♂, Paris, leg. P. Chrétien (all ZSM); 2 ♂, Dep. Cantal, Joursac, 4.vii.1996, leg. J. Nel, genitalia slides 5005 Nel, 5363 Nel; 1 ♂, Dep. Marne, Reims, Université, 4.vii.1997, leg. J. Nel, genitalia slide 5945 Nel (all TLMF). Greece. 1 ♂, 1 ♀ [form *separatellus*], Olympos, Kataphygion, 2000 m, 27–30.vi.1962, leg. J. Klimesch; 2 ♀ [form *dolosellus*], same data, but 2–-7.vii.1962, leg. J. Klimesch (all ZSM); 2 ♂, same data, but 2100 m, 12–18.vii.1962 leg. F. Kasy (NHMW, ZSM); 1 ♂, 1 ♀ [form *separatellus*], Chelmos, 2200–2300 m, 24.vi. 1958, leg. J. Klimesch; 1 ♂, same data, but 24.vi.1968, leg. J. Klimesch (all ZSM); 1 ♂, same data, but 30.v.2009, leg. J. Junnilainen [BOLD:AAG0031] (RCJJ); 4 ♂, Epirus, above Monodendri, 1300 m, 16.vi.2010, leg. P. Skou; 3 ♂, Florina, above Germanos, 2000 m, 27.vii.2013, leg. B. Skule & C. Hviid; 1 ♂, Fokida, Mt. Parnassos, road to southern ski resort, 1650 m, 10.vi.2010, leg. P. Skou; 1 ♂, same data, but 2.6 km N Kellaria Ski Center, 1600 m, 4.vi.2013; 1 ♂, Makedonia, Olympos, 700–2100 m, 21–26.v.1990, leg. O. Karsholt; 1 ♂, Makedonia, Kavala, Pangeo, 1700 m, 24.viii.1989, leg. K. Larsen; 1 ♂, Larissa, Olympos mts, below Skolio, 11 km NE Kalvia, 1850 m, 20.vii.1998, leg. B. Skule & D. Nilsson; 1 ♂, same data, but 3.vii.2004, leg. B. Skule; 1 ♀ [form *dolosellus*], Makedonia, 1 km NE Agios, Germanus, 1060 m, 14.vi.2013, leg. P. Skou; 7 ♂, Makedonia, 2.4 km SE Pisoderi, Vigia, 1550 m, 18.vi.2013, leg. P. Skou; 1 ♂, Makedonia, Mt. Voras, 4 km N Zervi, road to Voras Ski Resort, 1850 m, 5.vii.2016, leg. P. Skou (all ZMUC); 1 ♂, Makedonia, Kavala, Ofryno, Strymon Delta, 24.v.2009, leg. W. Schmitz; 2 ♂, Askion Oros, Vlasti, 1400 m, 7.vi.2014, leg. W. Schmitz (all RCWS); 4 ♂, Makedonia, 15 km W Kozani, Xerolimni, 21–23.v.2003, leg. J. Junnilainen; 1 ♂, same data, but 11.vi.2010, leg. J. Junnilainen; 7 ♂, Makedonia, Kozani NE, 23–24.v.2003, leg. J. Junnilainen; 26 ♂, Makedonia, Askio Vellia, 1450 m, 23.v.2003, leg. J. Junnilainen; 1 ♂, 1 ♀ [form *separatellus*], Makedonia, 15 km W Olympos, Leptokaria, 750 m, 27.v.2001, leg. J. Junnilainen, genitalia slides GU 16/1446 ♂ Huemer, GU 16/1467 ♀ Huemer (all RCJJ); 1 ♂, Epirus, Parga, 0 m, 1.vi.2002, leg. W. Schmitz (ZMUC); 1 ♂, Fokida, Parnassos, Itea/Desfina, 550 m, 21.v.2009, leg. W. Schmitz (RCWS).; 15 ♂, Drama, Phalakro mts, above Volas, 1700 m, 20.vii.1987, leg. M. Fibiger; 7 ♂, same data, but 15.vii.1998, leg. B. Skule & D. Nilsson (all ZMUC); 6 ♂, Peloponnes, Arkadien, Mari env. 620 m, 17.v.2009, leg. T. Mayr (TLMF, RCTM); 1 ♂, Peloponnes, Chelmos, 1600 m, 12.vi.2008, leg. J. Skyva (NMPC); 27 ♂, 1 ♀, Ipiros, Katara Pass, 15–1700 m, 24–27.v.1994, leg. O. Karsholt, genitalia slide GU 00/888 Huemer; 4 ♂, Ioánina, 8 km above Monodendri, Mt. Timfi, 1380 m, 22.vi.2004, leg. B. Skule; 1 ♂, Mt. Phalakron, above Volas, 6.vii.1986, leg. M. Fibiger, genitalia slide 6518 H. Hendriksen; 1 ♂, Makedonia/Thessalia, Olympos mts east, 900–1500 m, 17–18.v.1994, leg. O. Karsholt; 2 ♂, same data, but 700–2100 m, 21–26.v.1994; 1 ♂, Peloponnesos, Taygetos mts, 1000 m, 11.vi.1980, leg. G. Christensen; 2 ♂, same data, but 1700 m, 28–29.vi.1982, leg. B. Skule & S. Langemark; 2 ♂, 1 ♀, same data, but, above Trapenzandi, 5.vii.1984, 1800 m, leg. B. Skule, genitalia slide 5327 Karsholt; 2 ♂, Peloponnesos, Chelmos mts, above Kalavrita, 1700 m, 17–19.vi.1982; leg. B. Skule & S. Langemark; 1 ♂, Peloponnesos, Parnon Oros, 1700 m, 8.vii.1982; leg. B. Skule & S. Langemark; 2 ♂, same data, but western slope, 1000–1500 m, 3.vi.1994, leg. O. Karsholt; 1 ♂, Peloponnesos, Mt. Kyllini, above Trikala, 1650 m, 13.vi.2010, leg. P. Skou; 1 ♂, Thessaly, 3.1 km NE Metsovo, Katara Pass, 1580 m, 19.vi.2013, leg. P. Skou; 3 ♂, Epirus, 0.9 km NNE Kapesovo, 1160 m, 21.vi.2013, leg. P. Skou, genitalia slide GU 16/1441 ♂ P. Huemer (all ZMUC); 1 ♂, Crete, Mt. Ida, Südhang, Rouwawald, 1300 m, 15–31.vii.1938, leg. Dürck, genitalia slide in vial (ZSM). Hungary. 1 ♂, Szentggotthárd, 4.vi.1910, leg. Schmidt; 5 ♂, Leanyfalu, 5.–10.vii.1997, leg. B. S. Larsen; 2 ♂, 2 km N Börgönd, 24–25.vi.1998, leg. B. S. Larsen (all ZMUC); 1 ♂, Budafok, 20.vi.1916, leg. Uhrik; 1 ♂, 2 km SW Agárd, 20.vi.1998, leg. B. S. Larsen; 2 ♂, 3 km E Öksü, 29.vi.1998, leg. B. S. Larsen (all ZMUC); 1 ♂, Hortobágy, 18.v.1912; 1 ♀ [form *dolosellus*], same data, but 2.vi.1912 (all NHMW); 1 ♂, Csákberény, Bucka-hegy, 21.v.2005, leg. Z. Tokár; 1 ♂, Bélmegyer env., salt marshes, 9.v.2014, leg. Z. Tokár [BOLD:AAG0031]. Italy. 1 ♂, prov. Cuneo, Briga Alta, Monte Tanarello, 2000 m, 20.vii.1993, leg. G. Delmastro (TLMF); 2 ♂, prov. L´Aquila, NP Gran Sasso, Campo Imperatore, E Observatorio, 2010 m, 13.vii.2010, leg. P. Huemer, genitalia slide GEL 1198, 1200 Huemer, BC TLMF Lep 01493 (TLMF); 1 ♂, prov. Rieti, Monte Terminillo, 1730–1780 m, 12.vii.2010, leg. P. Huemer, genitalia slide GEL 1239 Huemer, BC TLMF Lep 04316 (TLMF); 2 ♂, prov. Roma, Monte Lepini, 4 km SE Carpineto Romano, 700 m, 20.v.2014, leg. J. Tabell, genitalia slide 16/1443 Huemer (ZMUC). Macedonia. 1 ♂, Treska Schlucht, 4–10.v.1963, leg. J. Klimesch; 1 ♂, Ohrid, Petrina Planina, 17–26.vi.1959, leg. J. Klimesch; 1 ♂, Shar Planina, Ljuboten, 1500–2000 m, 22.vi.1955, leg. J. Klimesch (all ZSM); 5 ♂, Galicica Planina, Stara Planina, Monte Magaro N, 2240 m, 26.vi.2009, leg. G. Tarmann; 9 ♂, NP Mavrovo, Korab, eastern ridge, 2325–2400 m, 28.vii.–1.viii.2011, leg. P. Huemer & G. Tarmann; 42 ♂, 2 ♀ [form *separatellus*], NP Mavrovo, Korab, Korabska jezero, Kobilino pole, 2080–2180 m, 28.vii.–1.viii.2011, leg. P. Huemer & G. Tarmann; 7 ♂, 1 ♀ [form *separatellus*], same data, but leg. P. Huemer & G. Tarmann [BOLD:ABA2915] (all TLMF); 5 ♂, same data, but 20–21.vii.2015, 2300 m, leg. I. Richter (RCIR); 1 ♂, Galicica, 12–13.vi. 2014, leg. I. Richter; 1 ♂, same data, but NP Galicica, 12.vi.2016; 1 ♂, same data, but NP Galicica, 15–16.vi.2017; 2 ♂, Bjelovodica, Mermerno jezero, 13–14.vi.2017, leg. I. Richter (all NMPC); 2 ♂, Ohrid, Galicica, 1400 m, 28.vi.2014, leg. J. Junnilainen [BOLD:ADB8790] (RCJJ); 1 ♂, NP Pelister, Golemo Jezero, 1.viii.2015, leg. I. Richter [BOLD:ABA2915] (RCIR); 2 ♂, Galicica NP, Galicica Saddle, 1380 m, 15–16.vi.2013, leg. P. Skou (ZMUC); 1 ♂, Prilep, Treskavac, 6.vi.2014, leg. I. Richter; 1 ♂, same data, but 10.vi.2016; 1 ♂, NP Mavrovo, 10 km N. Nalicnik, 20.vi.2016, leg. I. Richter; 4 ♂, Pivska pl. Pešče, 21.vi.2012, leg. I Richter (all NMPC). Montenegro. 1 ♂, Durmitor NP, 8.7 km NW Zabljak, Mali Stouc, 1880 m, 20–21.vii.2014, leg. C. Hviid & O. Karsholt [BOLD:ACS7352]; 1 ♂, Durmitor NP, 9 km E Zabljak, 1280 m, 24.vi.2013, leg. C. Hviid & O. Karsholt [BOLD:ACS7982]; 15 ♂, Durmitor National Park, 4 km S Žabljak, Virak, 1550 m, 22–28.vi.2013, leg. C. Hviid, O. Karsholt & K. Larsen; 1 ♂, Durmitor National Park, 6.8 km NW Žabljak, 1740 m, 23.vii.2014, leg. C. Hviid & O. Karsholt; 3 ♂, Durmitor National Park, 4 km S Žabljak, Virak, 1550 m, 19–24.vii.2014, leg. C. Hviid & O. Karsholt; 4 ♂, Durmitor National Park, 13 km SW Žabljak, Sedlo Pass, 1900 m, 19–24.vii.2014, leg. C. Hviid & O. Karsholt; 1 ♂, Durmitor National Park, 8 km S Žabljak, Virak, 1600 m, 19–24.vii.2014, leg. C. Hviid & O. Karsholt; 1 ♂, Durmitor National Park, 8.7 km NW Žabljak, Mali Stouc, 1880 m, 20–21.vii.2014, leg. C. Hviid & O. Karsholt (all ZMUC). Nepal. 1 ♂, Binap-Nager, 6.x.1996, leg. V. Cikolovecs, genitalia slide 17/1494 Huemer (RCZT). Romania. 1 ♂, Carpatii orientali, Mni. Baraolt, Ariusd, 600 m, 2.vi.1996, leg. S. & Z. Kovács; 1 ♂, Carpatii orientali, Muntii Ciucului, Racu, Cseretetö, 650 m, 13.vii.2004, leg. S. & Z. Kovács; 1 ♂, same data, but 14.vii.2005 (all RCKO); 6 ♂, 1 ♀ [form *dolosellus*], Rimetea area, 600 m, 29–31.v.2009, leg. O. Karsholt (ZMUC); 1 ♀ [form *dolosellus*], same data, leg. O. Karsholt [BOLD:AAG0031]; 1 ♂, same data, but 30.v.2009, leg. J. Junnilainen [BOLD:AAG0031] (RCJJ). Russia. 1 ♂, N Caucasus, Kabardino-Balkaria, river Gundelen, 1400 m, 10–20.vii.2012, leg. L. Srnka, genitalia prep. (in glycerin) (RCZT); 6 ♂, 3 ♀ [form *separatellus*], S Ural, Cheliabinsk distr., Arkaim reserve, near Akurskii vill., 16.vi.1996, leg. K. Nupponen et al.; 1 ♂, S Ural, Orenburg distr., 20 km S Pokrovka vill., Schibendy valley, 3–7.vi.1998, leg. J. Junnilainen (all RCJJ); 1 ♀ [form *separatellus*], same data, but 22.vi.1999, leg. T. & K. Nupponnen; 2 ♂, same data, but 22.vi.1999 (all ZMUC); 1 ♂, S-Ural, Moskovo env., 500 m, 18.vi.2009, leg. J. Šumpich; 2 ♂, same data, but 575 m, 19.vi.2009, leg. J. Šumpich; 2 ♂, same data, but 15–18.vii.2011, leg. J. Šumpich; 4 ♂, S-Ural, Moskovo, village, 6–7.vii.2013, leg. L. Srnka (all NNMP); 1 ♂, S Ural, Cheliabinsk distr., Kizilskoye village, 1.vii.2017, leg. H. Roweck & N. Savenkov (ECKU); 7 ♂, SW Altai, Katun valley, 10 km W Katanda, 1200 m, 22.vi.–8.vii.1983, leg. K. Mikkola, H. Hippa & J. Jalava, genitalia slide Hendriksen 2979 (MZH); 2 ♂, Altai mts, 10 km SE Aktash, 1500 m, 13.vii.1997, leg. C. Gielis (RCHW); 4 ♂, 3 ♀, Altai mts, Kuraisky hrebet, 2300 m, 10.vii.2001, leg. K. Nupponen (ZMUC); 2 ♂, Rep. Altai, Aktash vill., 1400 m, 21.vi.2015, lg. J. Šumpich; 2 ♂, Rep. Altai, 3 km SE Aktash, 1300 m, 17.vi.2012, leg. B. Schacht, genitalia slide GEL 1246 Huemer (all TLMF). Slovakia, 1 ♂, Plešivec, 12.vi.1987, leg. B. Å Bengtsson (ZMUC); 2 ♂, Komárno, 1.vi.1991, leg. G. Pastorális; 2 ♂, 3 ♀ [form *dolosellus*], Šal’a, Váh, 1.v.2007, leg. S. Tokár (NMPC, ZMUC); 1 ♂, Plášt’ovce, 2.vi.1986, leg. J. Patočka (ZMUC); 2 ♀ [form *dolosellus*], Šal’a, okolie Váhu, 2.v.2009, leg. Z. Tokár; 1 ♂, same data, leg. Z. Tokár [BOLD:ACS9604]; 1 ♀ [form *dolosellus*], same data, leg. Z. Tokár [BOLD:AAG0031]; 1 ♂, same data, but 11.v.2009, leg. Z. Tokár (all RCZT); 1 ♂, Slovenský Kras, Zádiel, 27.v.2001 (ZMUC). Slovenia. 3 ♂, Karst, Presnica, 15.v.2002, leg. H. Deutsch, genitalia slide GEL 1190 Huemer (TLMF); 3 ♂, Senezece, 500 m, 30.v.2008, leg. J. Junnilainen (RCJJ). Turkey. 3 ♂, Prov. Mersin, Çamliyayla, 5.vi.1984, leg. G. F. Curletti, genitalia slide Hendriksen 3339 (ZMUC). Ukraine. 1 ♂, Odessa, Borodino, Tarutinskaya steppe, 7.vi.2011, leg. A. Bidzilya, genitalia prep. (in glycerin) [BOLD:ADA0140] (RCAB); 1 ♂, Crimea, Karadag, 5.vii.1987, leg. R. Puplesis (ZMUC); 1 ♂, same data but 3.vi.2004, leg. Yu. Budashkin, genitalia slide GU 16/1464 P. Huemer; 1 ♂, same data, but 6.vi.2004; 1 ♂, same data, but 10.vi.2004 (all RCAB). Locality illegible. 1 ♀ [form *separatellus*], 22.vi.1902 (ZMUC).

##### Redescription.

**Figures 31–36.**
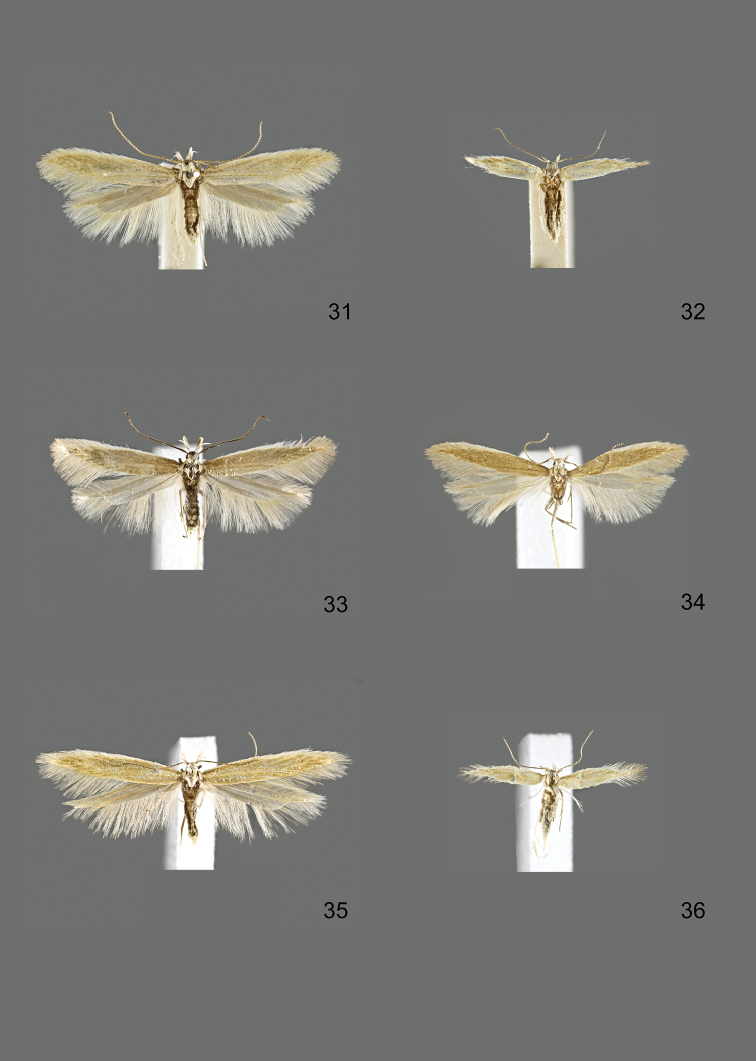
*Megacraspedus* adults in dorsal view. **31***M.dolosellus* (Zeller, 1839) – male, Greece (ZSM) **32***M.dolosellus* (Zeller, 1839) – female (form *separatellus*), Greece (ZSM) **33***M.dolosellus* (Zeller, 1839) – male, Bulgaria (RCJJ) **34***M.dolosellus* (Zeller, 1839) – male, Greece (TLMF) **35***M.dolosellus* (Zeller, 1839) – male, Russia (ZMUC) **36***M.dolosellus* (Zeller, 1839) – female, Russia (ZMUC).

**Figures 37–42.**
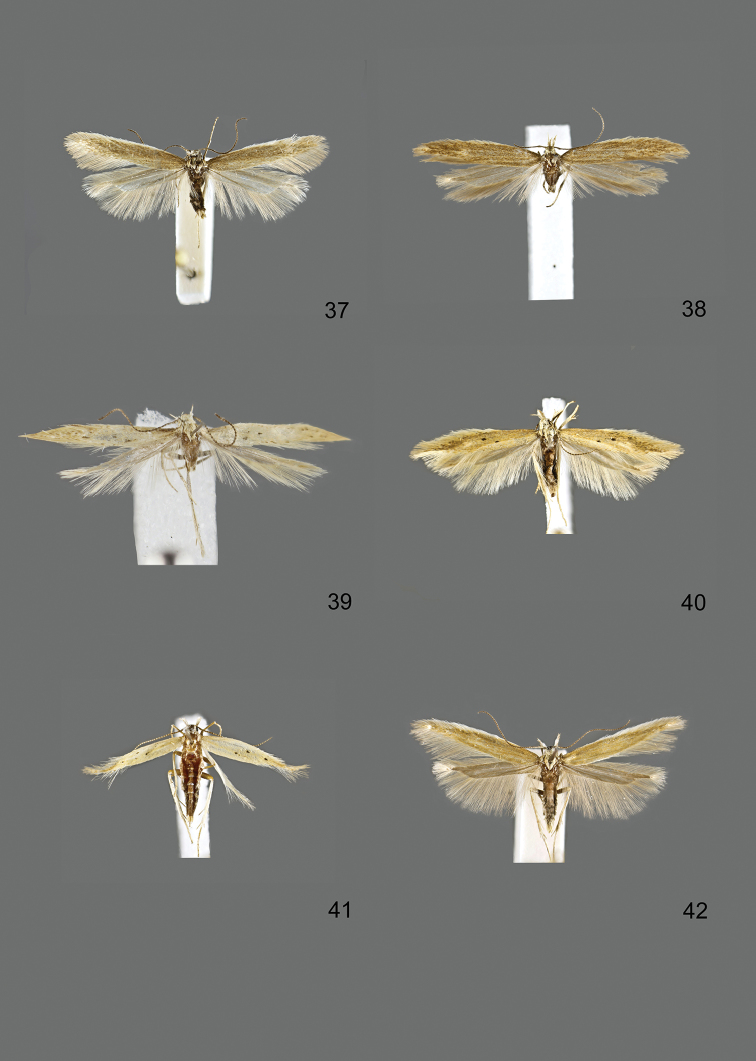
*Megacraspedus* adults in dorsal view. **37***M.dolosellus* (Zeller, 1839) – male (form *incertellus*), Bulgaria (RCZT) **38***M.dolosellus* (Zeller, 1839) – male, Greece (ZMUC) **39***M.neli* sp. n. – Holotype, male, France (TLMF) **40***M.faunierensis* sp. n. – Paratype, male, Italy (TLMF) **41***M.faunierensis* sp. n. – Paratype, female, Italy (TLMF) **42***M.gredosensis* sp. n. – Paratype, male, Spain (TLMF).

Adult. *Male* (Figs 29, 31, 33–38). Wingspan 10–15 mm. Segment 2 of labial palpus with scale brush about as long as segment 3, brown on outer and inner surface, white on lower and upper surface; segment 3 white. Antennal scape without pecten, flagellum ringed blackish and dirty white to nearly unicolorous black. Head, thorax and tegula cream coloured, mottled with brown. Forewing light brown (lightest towards dorsum) to light clay-brown; veins and costa clear white to finely dirty white; fringes grey. Hindwing grey with light grey fringes.

*Female* (Figs 30, 32). Wingspan 11–12 mm. Forewing broadest in middle, apical part pointed; fringes light grey. Hindwing lanceolate, about two-thirds length of forewing, whitish, with a few dark scales in apex; only scattered whitish fringes (form *dolosellus*) or hindwing slender with pointed tip, about one-fifth the length of forewing, whitish (form *separatellus*), hindwing about one-third length of forewings in several females from Altai mts Otherwise similar to male.

*Variation*. There is some variation in the forewing colour (more or less yellowish to greyish or brownish), and in the distinctness of the whitish veins. Worn specimens become lighter. Specimens from Turkey are larger (wingspan 14–18 mm) than European ones, but otherwise similar.

*Male genitalia* (Figs 167–172). Uncus moderately broad, 1.5 times as long as maximum basal width, sub-trapezoidal, weakly tapered to rounded apex; gnathos hook slender, straight, about length of uncus, with hardly curved and pointed apex; anterior margin of tegumen with moderate, broadly rounded emargination, sclerotised ridges extending from anterior margin and merged in medial zone; pedunculi small, rounded, with scleotized ridge; valva straight, slender, basally wider, apical part contorted, rounded, extending to about base of gnathos, saccular area covered with setae, with hardly separated sacculus, fused with valva; posterior margin of vinculum with curved medial emargination, broadly rounded lateral humps, vincular sclerite oblong, posterior edge strongly sclerotised; saccus moderately large, broadly V-shaped, with broad and short rod-like apex, ratio maximum width to length about 0.85, posterior margin with broadly rounded projections, separated by V-shaped emargination, medial smooth with at most very short sclerotised ridge, lateral sclerites broad, approximately 0.85 times length of maximum width of saccus; phallus about length of tegumen, stout, with bulbous coecum, distal two-thirds strongly S-curved, tapered apically, with rod-like ventral sclerotisation.

*Female genitalia* (Figs 271–274). Papilla analis small, apically rounded; apophysis posterior slender rod-like, approximately2.2–2.6 mm long, with short, weakly bifurcate posterior end; segment VIII 0.9–1.0 mm long, membranous; subgenital plate with sub-triangular subostial sclerotisation, posteriorly extended into shortly pointed sclerites, large medial flaps delimiting ostium bursae, anterior margin with rod-like edge connected with apophysis anterior, medially with broadly sinusoid projection; apophysis anterior 1.4–1.8 mm, slender, rod-like, posteriorly becoming rod-like venula of segment VIII, extending to posterior margin of segment VIII; ductus bursae short, moderately broad, about same length as corpus bursae; corpus bursae oblong, weakly delimited from ductus bursae, entire length of ductus and corpus bursae approximately 2.5 mm; signum small, variably shaped spiny plate.

##### Diagnosis.

Males are variable from light brown, with clear white veins and costa to light clay-brown and with veins and costa finely dirty white, ocassionally white without distinct markings. Females of form *dolosellus* have slender lanceolate hindwings, whereas females of form *separatellus* have very short ellipsoid hindwings. In a series of 5 females collected at the same place and date in Russia (Altai mts) 4 females have lanceolate hindwings but shorter than in the European form *dolosellus*, whereas a single female comes close to form *separatellus*. The male genitalia are very similar to the closely related *M.tokari* sp. n. (Figure 166) but can be separated in particular by the distinctly curved phallus. The female genitalia are somewhat similar to those of *M.lanceolellus* (Figs 267–268) but differ by the much shorter apophysis posterior and the shorter sinusoid anteriomedial projection of segment VIII. From the more closely related *M.sumpichi* sp. n. (Figure 279) they differ in the much longer apophysis anterior and the large medial flaps delimiting the ostium bursae. However, in the absence of females in other closely related species such as *M.tokari* sp. n. the diagnostic value of these characters for species delimitation remains uncertain.

##### Molecular data.

The extraordinary DNA barcode divergence is reflected by 23 BINs! The intraspecific divergence of the barcode region in this species is mean 7.5% and maximum 13.8%, largely reflecting a geographic pattern. However, on several occasions large intrapopulational divergence was detected, e.g., in specimens from Italy (Gran Sasso), Bulgaria (Pirin Mts Blagoewgrad), Montenegro (Durmitor), and Slovakia with two sympatric clusters in each region. Individual variation of specimens collected at the same time in the same microhabitat may be high in these cases as e.g., in clusters dolo03 and dolo04 with two specimens diverging 6.9% in DNA barcode.

The following 23 clusters are defined (based on sequenced material):

BIN dolo01 (Greece: Ioannina): BOLD:ACS7817 (n = 1).

BIN dolo02 (Bulgaria: Alibotusch, Pirin mts): BOLD:ACA9065 (n = 3).

BIN dolo03 (Italy: l`Aquila): BOLD:AA03318 (n = 1).

BIN dolo04 (Italy: l`Aquila): BOLD:AA03319 (n = 1).

BIN dolo05 (Italy: Rieti): BOLD:AAX3311 (n = 1).

BIN dolo06 (Italy: Lazio): BOLD:ACZ7902 (n = 1).

BIN dolo07 (Greece: Peloponnes): BOLD:ACQ6924 (n = 2).

BIN dolo08 (Bulgaria: Kyustendil): BOLD:ACZ9025 (n = 1).

BIN dolo09 (Bulgaria: Pirin mts): BOLD:ACR2396 (n = 5).

BIN dolo10 (Bulgaria: Blagoewgrad): BOLD:ADB8684 (n = 1).

BIN dolo11 (Montenegro: Durmitor): BOLD:ACS7982 (n = 1)

BIN dolo12 (Macedonia: Galicica): BOLD:ADB8790 (n = 2).

BIN dolo13 (Macedonia: Korab, Pelister): BIN BOLD:ABA2915 (n = 10).

BIN dolo14 (Montenegro: Durmitor): BIN BOLD:ACS7352 (n = 1).

BIN dolo15 (Croatia: Pag): BIN BOLD:ADB8686 (n = 1).

BIN dolo16 (Ukraine: Odessa): BOLD:ADA0140 (n = 1).

BIN dolo17 (Bulgaria: Blagoewgrad): BOLD:ADB8789 (n = 1).

BIN dolo18 (Slovenia): BOLD:AAV7561 (n = 3).

BIN dolo19 (Russia: Orenburg obl.): BOLD:ACZ3281 (n = 3).

BIN dolo20 (Russia: Altai mts): BOLD:ACB3319 (n = 1).

BIN dolo21 (Russia: Altai mts): BOLD:ACZ3530 (n = 4).

BIN dolo22 (Hungary, Romania, Slovakia): BOLD:AAG0031 (n = 4).

BIN dolo23 (Austria, Slovakia): BOLD:ACS9604 (n = 3).

The minimum distance to the nearest neighbour *M.lanceolellus* is 9.9% (p-dist).

##### Distribution.

Widely distributed from western Europe (northwards to ca. 49°N latitude) to Central Asia (Altai Mts). A specimen from the Himalaya Mountains needs verification. According to Mariani (1943: 174) also in Sicily. Absent from the Iberian Peninsula and the northern part of Central Europe as well as northern Europe.

##### Biology.

The larva was described in great detail (570 words, but no figures!) by Joannis (1923: 156–158). It lives from March to May underground in the rhizome of a wild grass (“Graminées sauvages”) (Joannis op. cit., Trouvelot 1923: 158–160). Although the larva makes long galleries within a rhizome, the plant does not seem to suffer from its presence because the roots beyond the attacked point are sufficient to ensure its nourishment. If wheat (*Triticumaestivum* L.) is sown in an abandoned field where *M.dolosellus* occurs, the larvae will attack the young wheat plants. The young larva occupies a similar situation, attacking one of the buried internodes of the roots, but this is serious because it causes the complete death of the affected plant (Trouvelot op. cit.) since wheat does not have rhizomes. The pupation takes place at the end of May, and Trouvelot obtained the first adults on 10^th^ June; they do not fly, but stay hidden or run at ground level (Trouvelot, op. cit.). Trouvelot did not state in which species of wild grass he found the larvae of *M.dolosellus*. According to Joannis (1923: 156) it was probably *Elymusrepens* (L.) Gould (“chiendent?”). Lhomme (1946: 539) referred to “chiendent” as *Cynodondactylon* Rich. [recte (L.) Pers.].

Chrétien (1924: 66–67) bred *M.dolosellus* from eggs. He placed some females in a container where he had planted *Poaannua* L., *Loliumperenne* L., and *Trifoliumrepens* L. The females placed their eggs in the leaf sheaths of the grasses or between stipules of the leaves and the stems of *T.repens*. The larvae hatched within 10–12 days, and the young larva descends to the base of the stems of the grasses. It bores into the stem and occupies a small space, depositing its frass inside the stem. Chrétien (op. cit.) did not explicitly write that larvae of *M.dolosellus* feed within stems or roots of *Trifolium*, and that plant is unlikely as a host for a *Megacraspedus* species, but he also recorded that he bred *M.dolosellus* from roots of *Poatrivialis* L.

The adults have been collected from early May to late August at altitudes from lowland localities to ca. 2400 m. The single record from Nepal dates from October. However, the identity of this specimen remains doubtful.

##### Remarks.

Ypsolophus (Megacraspedus) dolosellus was described from four males collected in June and July in the surroundings of Vienna, Austria (Zeller 1839: 190). Later it was redescribed in detail and both sexes figured in colour by Fischer von Röslerstamm (1843: 301, pl. 99, fig. 3).

*Ypsolophusseparatellus* was described from an unstated number of males and two females collected in the surroundings of Vienna (Austria) by J. Mann (Fischer von Röslerstamm 1843). The correct identification of males of *M.dolosellus* and *M.separatellus* was always considered to be an extremely difficult task as both taxa were considered as hardly separable in the male sex (Kasy 1987: 13). In contrast the two female morphotypes with longer lanceolate (form *dolosellus*) or short and pointed (form *separatellus*) hindwings were widely accepted as diagnostic for species identification by Kasy (*op. cit*.) and other authors. However, additional females from Russia (Altai mts) show intermediate characters with a tendency to individual infrasubspecific variation. We therefore cross-checked genetic divergence in both female morphotypes. Sequences of three morphologically identified females of *M.dolosellus* exhibit a considerable intraspecific divergence of mean 1.2% and maximum 1.9% with a barcode gap of 4.9% to *M.separatellus*. The intraspecific barcode divergence in two sequenced females of *M.separatellus* is even more pronounced at 2.3%. Alleged differences in forewing colour and pattern (light brown, with clear white veins and costa in *M.dolosellus*, light clay-brown and with veins and costa finely dirty white in *M.separatellus*) similarly underly infrasubspecific individual and geographic variation. Joannis (1923: 156) had already observed, based on seven specimens (apparently all males) bred from larvae, that fresh specimens were referable to *M.dolosellus*, whereas a worn specimen looked more like *M.separatellus*. He moreover noted that the colour of living specimens differed from dead and set specimens: *J’ajoute que, après étalage, les lignes blanches étaient moins visibles et la couleur semblait plus foncée. De plus l’aspect luisant que signalent les descriptions pour les ailes était insensible chez l’animal vivant, très sensible au contraire chez l’insecte étalé*. [I would add that, after mounting, the white lines were less visible and the colour looked darker. Moreover, the glossy aspect of the descriptions of the wings was indiscernible in the living animal, on the contrary very discernible in the spread insect”] (Joannis op. cit.). Finally, both the male and female genitalia of *M.dolosellus* are inseparable from those of *M.separatellus*, and alleged specific differences figured by Elsner et al. (1999: pl. 2, figs 7–8) proved infrasubspecific. We therefore consider *M.separatellus* to be a junior synonym of *M.dolosellus* (syn. n.).

*Megacraspedusincertellus* was described from an unstated number of males collected by AK Drenowski in the Alibotusch Mountains, S Bulgaria from 22–23.vii.1929 (Rebel 1930). A lectotype is here designated in order to fix the identity of the species and conserve stability of nomenclature. Specimens from South Bulgaria and North Greece are highly variable in DNA barcode with at least five strongly divergent clusters, either separated geographically or occurring sympatrically, e.g., in Pirin Mts. They differ from nominotypical *M.dolosellus* in the darker antennae, and the light grey-brown forewings with a white costa and reduced markings. However, such specimens are inseparable from nominotypical *M.dolosellus* in the male genitalia (female unknown). We therefore consider *M.incertellus* as a local form at most and a synonym of *M.dolosellus* (syn. n.), the latter of which shows an exceptional genetic and to some extent morphological variation. Barcode data clearly support several putative taxa, but the morphology is less straightforward. Phenotypic appearance is only partially congruent with geography and it is frequently impossible to reliably assign specimens to a barcode cluster. Male genitalia morphology is uniform and alleged subtle diagnostic characters underlie intraspecific variation. Considering the observed intrapopulational barcode variation of nearly 8% we conclude that *M.dolosellus* is as a widespread species, ranging from Eastern Europe to Central Asia. The exceptional DNA barcode divergence may be explained by weak dispersal ability, particularly of the strongly brachypterous females, leading to several genetically isolated populations. However, it seems unlikely that this is the sole cause for the observed intraspecific variation (see Discussion).

### *Megacraspedusfaunierensis* species group

The *M.faunierensis* species group includes two species: *M.neli* sp. n. and *M.faunierensis* sp. n.

External morphology. Segment 2 of labial palpus with scale brush shorter than segment 3; segment 3 as long as or longer than segment 2. Antennal scape without or with one fine hair. Wingspan (males) 11–14 mm. Forewing with three black dots, but without white costa and veins. Known females are brachypterous.

Genitalia morphology. Male genitalia. Uncus moderately small, about width of valva, sub-rectangular; gnathos hook moderately short, slender, straight, slightly longer than uncus; anterior margin of tegumen moderately emarginated; valva straight, stout; saccular area setose, with longitudinal ridge, without separated sacculus; saccus V-shaped, oblong, posterior margin with distinct projection, distally strongly tapered, with long medial ridge almost extended from posterior margin to apex; phallus weakly curved, with weakly inflated coecum and stout distal part, sclerotised dorsal ridge, ventrally wrinkled; ductus ejaculatorius with linear internal sclerotisation.

Female genitalia. Papilla analis small; apophysis posterior very long; segment VIII long and slender, largely membranous; subgenital plate with sub-triangular subostial sclerotisation, anteromedially with almost tubular projection; apophysis anterior rod-like, from posterior margin of segment VIII; colliculum short; signum small, rounded spiny plate.

Diagnosticremarks. The *M.faunierensis* species group is characterised by a combination of several diagnostic structures, particularly the small uncus, the straight and long gnathos hook, the longitudinal ridge of the saccular area, the oblong saccus with a longitudinal ridge and the shape of the phallus with wrinkles.

#### Megacraspedus neli sp. n.

Taxon classificationAnimaliaLepidopteraGelechiidae

http://zoobank.org/FDC671DF-8B9D-4F35-A9F0-2C07AA1AFF65

##### Examined material.

**Holotype** ♂, [France] “NEL Jacques [leg.] [Dep. Vaucluse, Montange de] Lure. Ou. 1700 m. 12.VI.1999” “P. Huemer GEL 1218 ♂” (TLMF). **Paratypes**. Italy. 1 ♂, prov. Torino, Valsusa, Mompantero, Monte Rocciamelone, 2200 m, 3.vii.1993, leg. G. Bassi, genitalia prep. (in glycerin) (ZMUC); 1 ♂, same data, but loc. Riposa, 2200 m, 16.vii.1993, leg. G. B. Delmastro (TLMF).

##### Description.

Adult. *Male* (Figure 39). Wingspan 11 mm. Segment 2 of labial palpus with scale brush shorter than segment 3, blackish brown on outer surface, white mottled with brown on inner surface, white on lower and upper surface; segment 3 longer than segment 2, cream-white with darker tip. Antennal scape with a single fine pecten; flagellum blackish brown ringed with grey. Head, thorax and tegula cream-white. Forewing cream coloured mottled with light brown and some black-tipped scales; base of costa dark grey; indistinct black dots in fold and at 3/5 in middle of wing and at end of cell; some black-tipped scales along termen; fringes light grey. Hindwing grey with light grey fringes.

*Female*. Unknown.

*Variation*. Unknown.

*Male genitalia* (Figure 173). Uncus moderately small, sub-rectangular, apical corners rounded, apical edge nearly straight; gnathos hook moderately slender, straight, apically pointed, 1.3 times length of uncus; anterior margin of tegumen with suboval emargination, sclerotised ridges from anterior edge converged in medial part of tegumen; pedunculi small, suboval, with irregular ridge; valva about width of uncus, stout, extending to about base of uncus, digitate distal part, apex rounded; saccular area densely covered with setae, with longitudinal ridge, without separated sacculus; posterior margin of vinculum distinctly emarginated, with shallow lateral humps, vincular sclerite elongated sub-ovate, with sclerotised posterior edge; saccus broadly V-shaped, ratio maximum width to length approximately 0.85, posterior margin with distinct projections, separated by broad incision, medial part with long sclerotised ridge from posterior margin to apical third, lateral sclerites 0.7 times length of maximum width of saccus; phallus weakly curved at about one-quarter, with weakly inflated coecum, 1.5 times wider than distal part, distal part 3 times length of coecum, sclerotised dorsal ridge, ventrally wrinkled, apex broadly rounded; ductus ejaculatorius with linear internal sclerotisation.

*Female genitalia*. Unknown.

##### Diagnosis.

*Megacraspedusneli* sp. n. is a rather uncharacteristic species of *Megacraspedus*, which may be recognized by its small size with a single pecten on the antennal scape. The holotype is somewhat worn and specimens in better condition may reveal additional diagnostic characters. It resembles *M.pentheres* (Figure 130), but that species has more distinct black dots in the forewing.

The male genitalia are similar overall to *M.faunierensis* sp. n. (Figure 174) but differ in several characters such as the larger uncus, the more slender valva, and in particular the much broader and shorter saccus.

##### Molecular data.

Not available, barcoding failed.

##### Distribution.

France (Dep. Vaucluse), Italy (prov. Torino).

##### Biology.

Host plant and early stages are unknown. The species was collected from the middle of June to mid-July at altitudes from ca. 1700 to 2200 m.

##### Etymology.

The species name (a noun in the genitive case) is dedicated to Jacques Nel, France, who collected the holotype of this species and numerous other *Megacraspedus* specimens used for our study.

##### Remarks.

The holotype was collected in the western part of Montagne de Lure, at the edge of the road at 1700 m altitude, between “the Refuge the Lure” and the “Signal de Lure” (J Nel in litt.).

#### Megacraspedus faunierensis sp. n.

Taxon classificationAnimaliaLepidopteraGelechiidae

http://zoobank.org/6AE2F46A-F21B-4FE9-B61D-28A6E4533378

##### Examined material.

**Holotype** ♂, “Italien, Prov. Cuneo Alpi Cozie, Demonte NW Colle Valcavera NE, 2420 m 7°6'23"E, 44°23'04"N 2.8.2010, leg. Huemer TLMF 2011-010” (TLMF). **Paratypes.** Italy. 26 ♂, 9 ♀, same data as holotype, but genitalia slides GEL 1219 ♂ Huemer, GEL 1235 ♀ Huemer (TLMF, ZMUC); 12 ♂, Prov. Cuneo, Demonte NW, Colle Fauniera Umgebung, 2480–2500 m, 3.viii.2008, leg. P. Huemer; 3 ♂, same data, but Colle Valcavera Umgebung, 2400–2500 m, 5.viii.2008, leg. P. Huemer; 1 ♂, same data, but Colle Valcavera NE, 2420 m, 4.viii.2008, leg. P. Huemer; 2 ♂, same data, but 23.vii.2009, leg. P. Huemer; 21 ♂, 3 ♀, same data, but 28.vii.2009, leg. P. Huemer (all TLMF); 11 ♂, 1 ♀, same data, but 2420 m, 27.vii.2009, leg. T. Mayr; 1 ♂, same data, but 17.vii.2012, leg. T. Mayr; 2 ♂, same data, but 18.vii.2012, leg. T. Mayr; 1 ♀, same data, but 16.viii.2013, leg. T. Mayr; 10 ♂, same data, but 17.viii.2013, leg. T. Mayr; 4 ♂, same data, but 2450 m, 22.vii.2018, leg. T. Mayr; 1 ♂, Prov. Cuneo, Gias Valcavera, 2050 m, 23.vii.2009, leg. T. Mayr; 14 ♂, 1 ♀, same data, but 22.vii.2018, leg. T. Mayr (all RCTM); 2 ♂, Prov. Cuneo, Valdieri N, RN Juniperus phoenicea, 900–1000 m, 29.vi.2008. leg. P. Huemer; 1 ♂, same data, but 8.vi.2009 (all TLMF).

##### Description.

Adult. *Male* (Figure 40). Wingspan 13–14 mm. Segment 2 of labial palpus with moderately long scale brush, brown on outer surface, white mottled with brown on inner surface, white on lower and upper surface; segment 3 cream-white. Antennal scape without pecten; flagellum dark brown ringed with white. Head, thorax and tegula cream-white. Forewing cream coloured mottled with some brown-tipped scales, especially in apical part; base of costa dark grey; a black dot in fold at 2/5 and two black dots at 3/5 in middle of wing and at end of cell; some black-tipped scales along termen; fringes light grey. Hindwing light grey with concolorous fringes. Female (Figure 41). Wingspan 11 mm. Forewing with apical part pointed, light yellowish brown, darker towards apex, fringes whitish. Hindwing lanceolate, whitish grey. Otherwise similar to male.

*Variation*. The amount of black scales on the forewings is variable. Rarely the black dot before the apex is absent. There is a slight variation in the colour of the forewings; worn specimens look more greyish.

*Male genitalia* (Figure 174). Uncus small, nearly sub-rectangular, basally weakly widened, apical corners rounded, apical edge straight with weak medial emargination; gnathos hook moderately slender, straight, apically pointed, approximately 1.2 times length of uncus; anterior margin of tegumen with suboval emargination, sclerotised ridges from anterior edge converge in medial part of tegumen; pedunculi small, suboval, with small transverse ridge; valva slightly broader than uncus, stout, extending slightly beyond base of uncus, digitate distal part, apex broadly rounded; saccular area densely covered with setae, with longitudinal ridge, without separated sacculus; posterior margin of vinculum weakly emarginated, without distinct lateral humps, vincular sclerite elongated sub-ovate, with sclerotised posterior edge; saccus prominent, slightly longer than valva, slender V-shaped, ratio maximum width to length 0.6, posterior margin with distinct projections, separated by broad incision, medial part with long sclerotised ridge from posterior margin to apex, lateral sclerites approximately 0.8 times length of maximum width of saccus; phallus weakly curved at about one-quarter, with weakly inflated coecum, 1.5 times wider than distal part, distal part 3 times length of coecum, sclerotised dorsal ridge, ventrally wrinkled, apex broadly rounded; ductus ejaculatorius with linear internal sclerotisation.

*Female genitalia* (Figure 275). Papilla analis small, apically rounded; apophysis posterior slender rod-like, 2.5 mm long, with short, bifurcate posterior end, bordered by minute sclerotised field; segment VIII 0.8 mm long, membranous; subgenital plate with sub-triangular subostial sclerotisation, posteriorly extended into long and pointed sclerites delimiting small ostium bursae, anterior margin with rod-like edge connected with apophysis anterior, medially with moderately short nearly tubular projection; apophysis anterior slender, rod-like, about half length of segment VIII, posteriorly becoming rod-like venula of segment VIII, extending to posterior margin of segment VIII; ductus bursae short, moderately broad; corpus bursae, moderately short and broad, weakly delimited from ductus bursae, entire length of ductus and corpus bursae nearly 2 mm; signum small, rounded spiny plate.

##### Diagnosis.

*Megacraspedusfaunierensis* sp. n. is characterised by its cream coloured forewings with three distinct black spots. It resembles *M.tristictus* (Figure 48) which has darker forewings, but *M.faunierensis* sp. n. has the head white and the antennae darker. It is also similar to *M.pentheres* (Figure 130), which shares the yellowish brown colour of the forewings with *M.tristictus*, but has the dot at the end of the cell largely reduced, and there are several small dark spots in the terminal area. The male genitalia differ from the probably closest species *M.neli* sp. n. (Figure 173) particularly in the smaller uncus, broader valva, and the shape of the saccus. They somewhat resemble those of *M.leuca* (Figure 237) in this character, although the saccus is V-shaped and not U-shaped, but differ in many other structures. The female genitalia are similar to several species such as *M.sumpichi* sp. n. (Figure 279) and differ in particular by the tubular rather than sinusoid projection of the anterior edge of segment VIII. However, this character is unknown for related species and maybe of limited diagnostic value in the delimitation of species.

##### Molecular data.

BIN BOLD:AAJ3164 (n = 3). The intraspecific divergence of the barcode region is considerable with mean 1% and maximum divergence of 1.6%. However, this divergence is exclusively based on a divergent specimen from a lowland locality (Prov. Cuneo, Valdieri N, RN Juniperus phoenicea, 900–1000 m, 29.vi.2008. leg. P. Huemer). The distance to the nearest congeneric neighbour *M.sumpichi* sp. n. is 11.9%, the distance to the nearest BIN in BOLD, an unnamed Limacodidae, is 9.1% (p-dist).

##### Distribution.

North-western Italy.

##### Biology.

Host plant and early stages are unknown. The adults have been collected from early June to mid-August, depending on the altitude. In alpine habitats they were found flying freely in the early morning about sunrise. At this time several specimens could be collected in copula, mainly sitting on low vegetation, particularly on blades of grass. The altitudes range from ca. 900 to 2500 m.

##### Etymology.

This species is named after the place of occurrence of part of the type series: the Colle di Fauniera pass in northern Italy. The name is a noun in apposition.

### *Megacraspedusgredosensis* species group

The *M.gredosensis* species group includes one species, *M.gredosensis* sp. n.

External morphology. See species description.

Genitalia morphology. Male genitalia. See species description.

Female genitalia unknown.

Diagnosticremarks. The *M.gredosensis* species group is defined by unique structures in the male genitalia. In particular the distinctly digitate sacculus and the phallus with a small coecum, and a long and slender distal part with subapical tooth are characteristic structures.

#### Megacraspedus gredosensis sp. n.

Taxon classificationAnimaliaLepidopteraGelechiidae

http://zoobank.org/0DA336DC-65B5-4E51-9208-3EED1FB4045B

##### Examined material.

**Holotype** ♂, “Hispania, [prov. Avila] 19.7.1980 Sierra de Gredos Navacepeda, 1500 m M. u. E. Arenberger” “GU 16/1416 ♂ P. Huemer“ (RCEA). **Paratypes.** Spain. 1 ♂, same data as holotype (TLMF); 1 ♂, prov. Avila, Sierra de Villafranca, 1 km W La Herguijulea, 1650 m, 20.vii.2003, leg. B. Skule, genitalia slide 5314 Karsholt (ZMUC); 1 ♂, same data, but 15.vi.2012, leg. T. Nupponen, genitalia slide 17/1489 Huemer (RCKN).

##### Description.

Adult. *Male* (Figure 42). Wingspan 15 mm. Segment 2 of labial palpus with scale brush about as long as segment 3, dark brown on outer surface, whitish brown on inner surface, white on lower and upper surface; segment 3 white mottled with some black. Antennal scape without pecten; flagellum blackish brown, indistinctly ringed with dirty white. Head, thorax and tegula cream coloured, mottled with brown. Forewing yellow-brown, mottled with grey towards costa; costa basally grey-brown, otherwise pure white; veins indistinctly white; fringes light grey. Hindwing dark grey with grey fringes.

*Female*. Unknown.

*Variation*. Unknown.

*Male genitalia* (Figure 175–176). Uncus moderately slender, approximately 2.5 times as long as maximum basal width, with parallel outer margin, distal part with weakly rounded apex; gnathos hook stout, about length of uncus, bent near base and slightly curved at apex, distal part club-shaped, apically pointed; anterior margin of tegumen with broadly rounded emargination; pedunculi moderately small, with additional sclerotisation; valva moderately slender, basal part distinctly wider than distal part, nearly straight with weakly curved and inflated apical part, extending to about base of uncus; sacculus long, slender digitate; posterior margin of vinculum with distinct medial emargination, broadly rounded lateral humps, vincular sclerites slender, with sclerotised proximal edge; saccus irregularly V-shaped, medially bulged, apical fifth abruptly tapered, ratio maximum width to length approximately 0.65, posterior margin with sub-triangular mediolateral projections, separated by deep incision, media part with long ridge, forked in middle of saccus, lateral sclerites stout, about length of maximum width of saccus; phallus with bulbous coecum, distal three-quarters slender, basally curved, a short sclerotised ridge with a single tooth subapically on ventral surface.

*Female genitalia*. Unknown.

##### Diagnosis.

*Megacraspedusgredosensis* sp. n. is characterised by its yellow-brown forewings with a clear white costa and no black dots, and the dark grey hindwigs. It may resemble species of the *M.dolosellus*-complex, but these have more pure whitish veins, but not a pure white costa and lighter hindwings. The male genitalia are somewhat similar to *M.glaberipalpus* sp. n. (Figs 226–227) but are unmistakable due to the characteristic shape of the phallus.

##### Molecular data.

BIN BOLD:ADI8272 (n = 1). The distance to the nearest neighbour *M.bidentatus* sp. n. is 10.01% (p-dist).

##### Distribution.

Central Spain (prov. Avila).

##### Biology.

Host plant and early stages are unknown. The few adults known to date have been collected in the middle of July at altitudes between 1500 and 1650 m.

##### Etymology.

This species is named after its place of occurrence: the mountain range of Sierra de Gredos in Central Spain. The name is an adjective.

### *Megacraspeduscuencellus* species group

The *M.cuencellus* species group includes four species: *M.cuencellus*, *M.bidentatus* sp. n., *M.fuscus* sp. n., and *M.trineae* sp. n.

External morphology. Segment 2of labial palpus with scale brush shorter to longer than segment 3; segment 3 as long as or longer than segment 2. Antennal scape without pecten. Wingspan (males) 10–14 mm. Forewing in some species with 1–2 indistinct black dots, but without white costa and veins. Females unknown.

Genitalia morphology. Male genitalia. Uncus small; gnathos hook stout, distally narrowing, curved and apically pointed, about length of uncus; anterior margin of tegumen with broadly rounded emargination; valva straight, stout, basal part distinctly wider than distal part, distal part weakly contorted; saccular area setose, without separated sacculus; saccus massive, larger than tegumen, semi-oval, with weakly pointed apex, posterior margin arched, with pointed mediolateral projections, medial part with strongly sclerotised longitudinal ridge, lateral sclerites short; phallus moderately slender, medially bent, orbicular coecum, distal two-thirds slender, sclerotised ridges with 1–2 strong teeth; ductus ejucalatorius with contorted linear interior sclerotisation.

Female genitalia unknown.

Diagnosticremarks. The *M.cuencellus* species group is unmistakable due to several characters of the male genitalia such as the small uncus compared to the tegumen, the massive saccus, and the dentation of the phallus.

#### Megacraspedus cuencellus

Taxon classificationAnimaliaLepidopteraGelechiidae

Caradja, 1920

Megacraspedus cuencellus Caradja, 1920: 117.

##### Examined material.

**Holotype** ♂, “Cuenca V Korb” “HOLOTYPE *Megacraspedus cuencellus* Car. ♂ ROMANIA” “CIS-Korea Microlep. 4216 *Megacraspedus cuencellus* Car. – Cuena [sic], Spain K. T. Park” (MGAB) [photographs examined]. **Non type-material.** France. 1 ♂, Alpes Maritimes, Caussols, 1100 m, 22.v.2002, leg. J. Nel, genitalia slide 14295 Nel (TLMF). Spain. 1 ♂, prov. Teruel, Carretera Moscardón, 1600 m, 5.vii.2010, leg. Z. Tokár, genitalia slide GU 15/1401 Huemer (RCZT).

##### Redescription.

**Figures 43–48.**
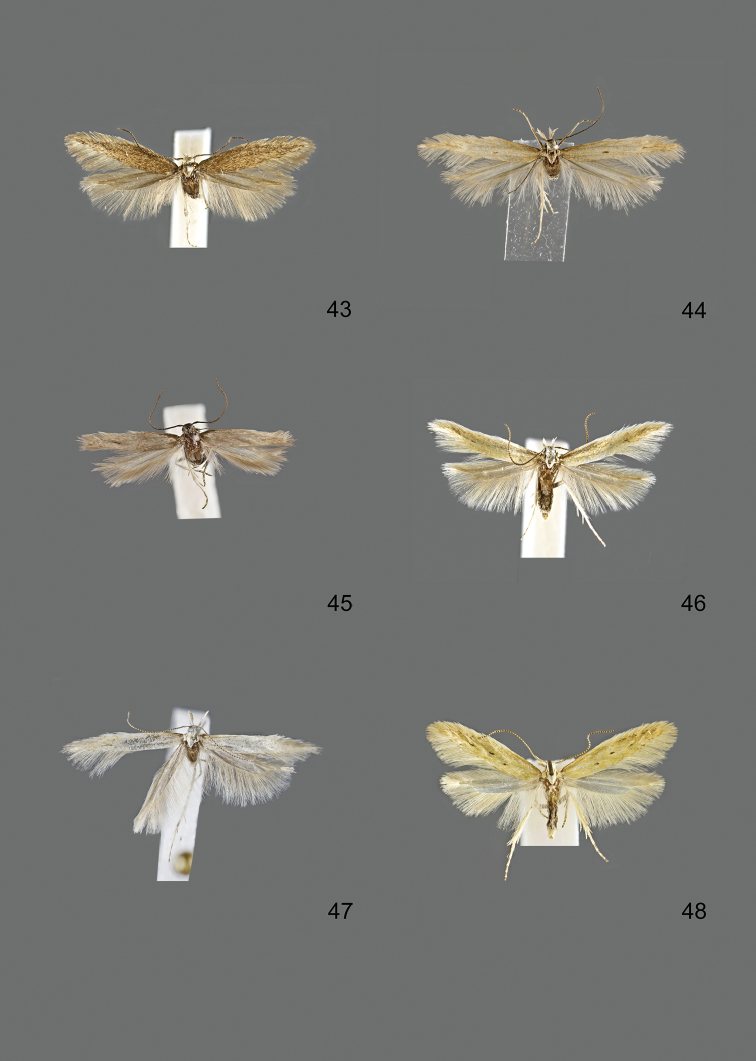
*Megacraspedus* adults in dorsal view. **43***M.cuencellus* Caradja, 1920 – male, Spain (RCZT) **44***M.bidentatus* sp. n. – Holotype male, Spain (ZMUC) **45***M.fuscus* sp. n. – Holotype, male, Spain (ZMUC) **46***M.trineae* sp. n. – Paratype, male, Portugal (ZMUC) **47***M.cf.trineae* sp. n. – male, Spain (ZMUC) **48***M.tristictus* Walsingham, 1910 – male, France (RCEA).

Adult. *Male* (Figure 43). Wingspan 10–14 mm. Segment 2 of labial palpus with large scale brush, dark brown on outer surface, white mottled with brown on inner and lower surface, white upper surface; segment 3 white with black tip. Antennal scape without pecten; flagellum black, indistinctly lighter ringed. Head, thorax, and tegula light grey. Forewing dark grey from blackish, light grey-based scales; fringes grey. Hindwing dark grey with grey fringes.

*Female*. Unknown.

*Variation*. The forewing colour varies slightly from brownish grey to darker grey. The holotype (examined from a photograph) differs in having black streaks in the fold and towards apex.

*Male genitalia* (Figure 177). Uncus moderately small and slender, about two times longer than wide, sub-rectangular with rounded apex; gnathos hook strong, about length of uncus, distally moderately curved with pointed apex; anterior margin of tegumen with deep, U-shaped emargination, pedunculi distinct, suboval; valva nearly straight, stout, extending almost to apex of uncus, basal part distinctly wider than distal part, distal part weakly curved dorsad, apically rounded; saccular area densely covered with setae, without separated sacculus; posterior margin of vinculum with moderate medial emargination, weakly rounded lateral humps, broadly sub-rectangular vincular sclerites extending from sclerotised posterior edge of saccus to sub-basal part of valva; saccus massive, semi-oval, with weakly pointed apex, ratio maximum width to length about 1, posterior margin arched, with pointed mediolateral projections, separated by shallow medial emargination, medial part with strongly sclerotised longitudinal ridge extending almost to middle of saccus, lateral sclerites short, nearly about half length of maximum width of saccus; phallus moderately slender, medially bent, orbicular coecum, distal two-thirds slender, sclerotised medial ridge with strong tooth, ventral edge with strongly sclerotised subapical tooth, ductus ejucalatorius with contorted linear interior sclerotisation.

*Female genitalia*. Unknown.

##### Diagnosis.

*Megacraspeduscuencellus* is characterised by its almost plain dark grey forewings and dark hindwings but largely agrees with *M.bidentatus* sp. n. (p 65), *M.fuscus* sp. n. (p 67) and *M.skoui* sp. n. (p 74). The male genitalia are unmistakable due to the unique structures of the phallus. *M.cuencellus* differs from other species particularly in the thorns of the phallus having a different position.

##### Molecular data.

BIN BOLD:ACC5029 (n = 1). The distance to the nearest neighbour *M.bidentatus* sp. n. is 3.1% (p-dist).

##### Distribution.

Southern France (Nel and Varenne 1997), Spain.

##### Biology.

Host plant and early stages are unknown. The few adults have been collected from late May to early July at altitudes of between 1500 and 1650 m.

##### Remarks.

*Megacraspeduscuencellus* was described from one male in good condition from the province of Cuenca in Central Spain (Caradja 1920: 117). The genitalia of the holotype were figured by Park (1996: 75, figs 50–52). A male specimen from France corresponds with *M.cuencellus* from Spain in the genitalia and in the short COI sequence of 407 bp.

#### Megacraspedus bidentatus sp. n.

Taxon classificationAnimaliaLepidopteraGelechiidae

http://zoobank.org/D621CAD0-B1DA-4F12-9009-C14BEDDD5F28

##### Examined material.

**Holotype** ♂, “SPAIN [prov. Barcelona] Gironella 680 m N42.03975 E2.02987 9.5.2013 J. Tabell leg.” “GU 16/1433 ♂ P. Huemer” “DNA Barcode TLMF Lep 19855” (ZMUC).

**Paratype.** Spain. 1 ♂, same data as holotype (ZMUC).

##### Description.

Adult. *Male* (Figure 44). Wingspan 13–14 mm. Segment 2 of labial palpus with scale brush of about length of segment 3, dark brown on outer surface, dark brown mottled with white on inner surface, white on lower and upper surface; segment 3 cream-white with brown tip. Antennal scape without pecten; flagellum blackish brown. Head cream-white mottled with light brown; neck white; thorax as forewing; tegula whitish brown. Forewing greyish brown from light black- and brown-tipped scales, costal third more blackish; a narrow yellow line in fold ending in a small black dot; a small black dot at end of cell; termen with some black scales; fringes light grey. Hindwing grey with light grey fringes.

*Female*. Unknown.

*Variation*. One of the two examined specimens has the rather denuded forewings light greyish.

*Male genitalia* (Figure 178). Uncus moderately small, 1.7 times longer than maximum basal width, sub-triangular, evenly tapered to rounded apex; gnathos hook strong, about length of uncus, distally curved with pointed apex; anterior margin of tegumen with deep, U-shaped emargination, pedunculi distinct, suboval; valva nearly straight, stout, extending almost to apex of uncus, basal part distinctly wider than distal part, distal part weakly curved dorsad, apically rounded; saccular area covered with setae, without separated sacculus; posterior margin of vinculum with shallow medial emargination, weakly rounded lateral humps, broad vincular sclerites extending from sclerotised posterior edge of saccus to sub-basal part of valva; saccus massive, semi-oval, with pointed rod-like apex, ratio maximum width to length approaching 1, posterior margin arched, with pointed mediolateral projections, separated by shallow medial emargination, medial part with strongly sclerotised longitudinal ridge extending almost to middle of saccus, lateral sclerites short, about half length of maximum width of saccus; phallus moderately slender, medially bent, orbicular coecum, distal two-thirds slender, sclerotised medial ridge with subapical tooth, ventral edge with subapical tooth, ductus ejucalatorius with contorted linear interior sclerotisation.

*Female genitalia*. Unknown.

##### Diagnosis.

**Figures 49–54.**
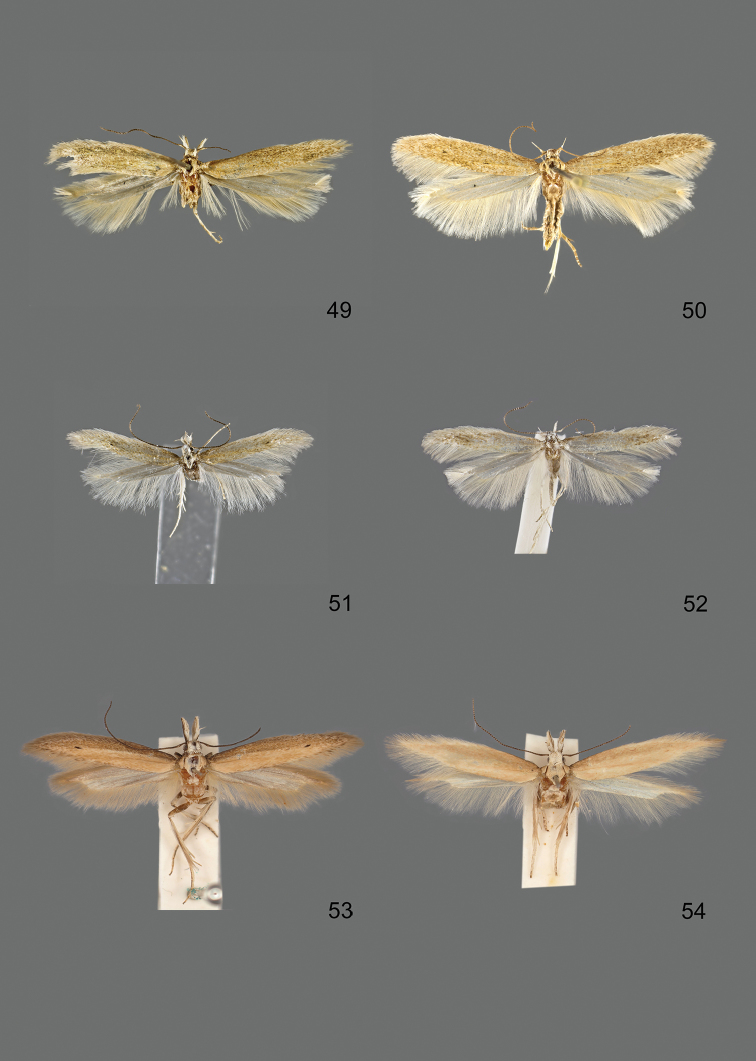
*Megacraspedus* adults in dorsal view. **49***M.alfacarellus* Wehrli, 1926 – Holotype, male, Spain (NHMB) **50***M.alfacarellus* Wehrli, 1926 – male, Spain (ZMHU) **51***M.pusillus* Walsingham, 1903 – male, Spain (ZMUC) **52***M.skoui* sp. n. – Paratype, male, Spain (RCZT) **53***M.spinophallus* sp. n. – Paratype, male, Spain (BMNH) **54***M.spinophallus* sp. n. – Paratype, female, Spain (BMNH).

*Megacraspedusbidentatus* sp. n. is characterised by its greyish brown forewings, which are darker in the costal third and have a distinct black dot at end of the fold and at the cell. It is similar to *M.alfacarellus* (Figs 49–50) which is sligtly larger and has more yellow in the fold and more indistinct black dots on the forewings. It is also similar *M.pusillus* (Figure 51), but in that species the forewings are not darker in the costal third. *M.fuscus* sp. n. (Figure 45) differs in being almost unicolorous blackish grey. See also *M.cuencellus* (p 64). The male genitalia of *M.bidentatus* sp. n. are almost unmistakable due to unique structures, particularly the phallus, somewhat similar to *M.fuscus* sp. n. (Figure 179) from which it differs in several characters such the shape of the uncus, valva and the saccus. From the similar *M.cuencellus* (Figure 177) it differs in the shape of the uncus and the larger thorns of the phallus having a different position.

##### Molecular data.

BIN BOLD: ADA0203 (n = 1). The distance to the nearest neighbour *M.cuencellus* is 3.1% (p-dist).

##### Distribution.

North-eastern Spain (prov. Barcelona).

##### Biology.

Host plant and early stages are unknown. The two type specimens were collected in early May at an altitude of 680 m.

##### Etymology.

The species name refers to the sclerotisations of the phallus and is derived from the Latin word *bidentatus* (having two teeth). The name is a masculine adjective.

#### Megacraspedus fuscus sp. n.

Taxon classificationAnimaliaLepidopteraGelechiidae

http://zoobank.org/74345AA8-E895-41AE-A77B-D0831E875F6B

##### Examined material.

**Holotype** ♂, “Spain, Extremadura [prov. Cáceres] 39°34'11"N, 06°06'34"W 11 km SE Monroy 4. iv. 2009, 375 m leg. B. Skule” “DNA Barcode TLMF Lep 21299” “GU 17/1478 ♂ P. Huemer” (ZMUC). **Paratype.** Spain. 1 ♂, same data as holotype (ZMUC); 1 ♂, prov. Salamanca, Belena, 8.v.1979, leg. C. Gielis, genitalia slide 5344 Karsholt (RMNH).

##### Description.

Adult. *Male* (Figure 45). Wingspan 13 mm. Segment 2 of labial palpus with scale brush shorter than segment 3, blackish brown, mottled with white on inner surface; segment 3 longer than segment 2, black mottled with white on inner surface. Antennal scape without pecten; flagellum black. Head, thorax and tegula blackish grey. Forewing blackish grey; fold slightly lighter; an indistinct black dot in fold and at end of cell; fringes dark grey. Hindwing dark grey with grey fringes. Abdomen black.

*Female*. Unknown.

*Variation*. Unknown.

*Male genitalia* (Figure 179). Uncus moderately small, approximately 1.5 times longer than maximum basal width, sub-rectangular, apical edge weakly rounded; gnathos hook strong, about one-third longer than uncus, distally weakly curved with pointed apex; anterior margin of tegumen with broad and deep U-shaped emargination, pedunculi distinct, suboval; valva nearly straight, stout, extending almost to apex of uncus, basal part distinctly wider than distal part, distal part weakly curved dorsad, apically rounded, sub-basal edge with long and sword-shaped sclerite, pointed disto-posteriad; saccular area covered with setae, without separated sacculus; posterior margin of vinculum with shallow medial emargination, weakly rounded lateral humps, broadly suboval vinucular sclerites extending from sclerotised posterior edge of saccus to sub-basal part of valva; saccus nearly V-shaped, with rounded apex, ratio maximum width to length approximately 0.6, posteriolateral margin medially projected, without incision, medial part with broad and strongly sclerotised longitudinal ridge extending almost to apex of saccus, lateral sclerites slightly longer than maximum width of saccus; phallus moderately slender, nearly straight, orbicular coecum, distal two-thirds slender, sclerotised medial ridge with medial and ventral tooth, ductus ejucalatorius with contorted linear interior sclerotisation.

*Female genitalia*. Unknown.

##### Diagnosis.

*Megacraspedusfuscus* sp. n. is characterised by its almost plain blackish grey forewings and dark hindwings. Externally it mostly resembles *M.grisea* from Asia, but that species has ringed antennae and some lighter scales along the termen. It is also similar to, but darker than, *M.cuencellus* (Figure 43), *M.pusillus* (Figure 51) and *M.skoui* sp. n. (Figure 52). The male genitalia are unmistakable, due to unique structures, such as the sword-shaped sclerites of the valva, the peculiar saccus with a broad longitudinal ridge, and the dentation of the phallus situated mediad compared to *M.bidentatus* (Figure 178).

##### Molecular data.

Not available, barcoding failed.

##### Distribution.

Western Spain (prov. Cáceres, Salamanca).

##### Biology.

Host plant and early stages are unknown. The type specimens were collected in early April and early May at altitudes from 375 m to ca. 900 m.

##### Etymology.

The name of this species is derived from the Latin word *fuscus* (meaning dark) after the colour of the forewings. The name is a masculine adjective.

##### Remarks.

*Megacraspedusfuscus* sp. n. is tentatively attributed to the *M.cuencellus* species group due to the overall similarity in the male genitalia. Molecular data will be necessary to confirm this classification.

#### Megacraspedus trineae sp. n.

Taxon classificationAnimaliaLepidopteraGelechiidae

http://zoobank.org/34CDDD41-3E72-4E7B-9B68-6617B3CDF087

##### Examined material.

**Holotype** ♂, “Portugal BB [= prov. Beira Baixa] Serra de Estréla Torre, 1600–1950 m 10.-11.vii.1986 O. Karsholt” “Gen. Præparat N^o^ 3355 ♂ H. Hendriksen” (ZMUC). **Paratypes.** Portugal. 12 ♂, same data as holotype, but genitalia slide GEL 1206 Huemer (TLMF, ZMUC); 1 ♂, Braçais, Castello de Vide, Alto Alentejo, 9.v.1999, leg. M. Corley, genitalia slide 2003 Corley (RCMC); 3 ♂, Poco do Inferno, 1100 m, 6.vii.2014, leg. M.F.V. Corley, genitalia prep. (in glycerin) (RCMC). Spain. 1 ♂, Castilia, viii.1886, leg. O. Staudinger, genitalia slide Mus. Vind. 16.655 (NHMW); 1 ♂, prov. Segovia, San Ildefonso, 23.vi. [without year], leg. Staudinger, genitalia prep. (in glycerin); 1 ♂, same data, but 21.vi., leg. O. Staudinger; 1 ♂, same data, but 10.vii, leg. O. Staudinger (ZMHU). No collecting data. 1 ♂, 25.vi., coll. O. Staudinger (ZMHU). **Excluded from the type-series** (see Remarks): Spain. 1 ♂, prov. Avila, 5 km NW Guisando, 1150–1400 m, 21.vi.2012, leg. T. Nupponen, genitalia slide 5014 Tabell (ZMUC).

##### Description.

Adult. *Male* (Figure 46). Wingspan 12–13 mm. Segment 2 of labial palpus with moderately long scale brush, brownish on outer surface, white mottled with brown on inner surface, otherwise white; segment 3 white, mottled with black on outer surface and with black tip. Antennal scape without pecten; flagellum blackish brown ringed with light grey. Head cream-white, thorax, and tegula as forewing. Forewing bone white, mottled with light brown in costal half; a weak black dot at end of cell; costa blackish brown at base, otherwise white; fringes light grey. Hindwing greyish with concolorous fringes.

*Female*. Unknown.

*Variation*. A rather variable species. The colour of the forewing varies from almost plain bone white to light brownish with a white costa. A rather indistinct black dot is sometimes present at the end of the cell. Sometimes there are a few black scales at the end of the cell and/or near apex.

*Male genitalia* (Figs 180–181). Uncus moderately slender, about two times longer than wide, sub-rectangular with broadly rounded apex; gnathos hook strong, about length of uncus, weakly curved with pointed apex; anterior margin of tegumen with deep V-shaped emargination, pedunculi small, suboval; valva straight, moderately slender, basal part wider than distal part, extending to about apex of uncus, apically rounded; short, digitate sacculus at medial part of valva, weakly separated; posterior margin of vinculum with moderate medial emargination, weakly rounded lateral humps, broad semi-oval sclerites extending from sclerotised posterior edge of saccus to sub-basal part of valva; saccus massive, suboval, with abruptly tapered apex, ratio maximum width to length approaching 1, posterior margin arched, with shallow medial emargination, medial part with strongly sclerotised longitudinal ridge extending to anterior third of saccus, lateral sclerites short, about half length of maximum width of saccus; phallus slender, medially bent, orbicular coecum, distal three-quarters slender, rod-like, distal half with strongly sclerotised ridge and a single postmedial tooth, subapical area with few small thorns, ductus ejucalatorius with contorted linear interior sclerotisation.

*Female genitalia*. Unknown.

##### Diagnosis.

*Megacraspedustrineae* sp. n. is a rather uncharacteristic *Megacraspedus* which can be recognized by lacking black dots (apart from an indistinct one at the end of the cell) and the whitish coloured veins on the forewings. It is very similar to *M.occidentellus* sp. n. (p 79). The male genitalia differ from other species of the *M.cuencellus* species group in the characteristic postmedial tooth and the presence of a short sacculus.

##### Molecular data.

BIN BOLD:ADF0469 (n = 1). The distance to the nearest neighbour *Megacraspedusbidentatus* sp. n. is 7.8% (p-dist).

##### Distribution.

Portugal and Spain.

##### Biology.

Host plant and early stages are unknown. The adults have been collected in early May and in the first half of July at altitudes from 1100 to ca. 1900 m.

##### Etymology.

The species name (a noun in the genitive case) is dedicated to Trine Karsholt, Denmark, who assisted OK when collecting the holotype and a part of the type series of this species.

##### Remarks.

A single male from Central Spain (Figure 47) differs in the broader phallus and sclerotised ridge (Figure 182), whereas other characters, e.g., the presence of a short sacculus, cannot be determined with certainty due to the traditional preparation technique employed. As well as the obvious differences in the genitalia morphology the considerable DNA barcode distance (BIN BOLD:ACZ8654) of 7.2% to *M.trineae* sp. n. indicates a, presumably, different species. However, in the absence of sufficient material we postpone a description.

### *Megacraspeduspusillus* species group

The *M.pusillus* species group includes nine species: *M.tristictus*, *M.alfacarellus*, *M.pusillus*, *M.skoui* sp. n., *M.spinophallus* sp. n., *M.occidentellus* sp. n., *M.granadensis* sp. n., *M.heckfordi* sp. n., and *M.tenuiuncus* sp. n.

External morphology. Segment 2 of labial palpus with scale brush about same length as segment 3; segment 3 shorter or as long as segment 2. Antennal scape without pecten. Wingspan (males) 9–20 mm. Forewing with 0–3 black spots, but without white costa (except in *M.tenuiuncus* sp. n.), in some species with lighter dorsum. Female (only known for *M.spinophallus* sp. n.) moderately brachypterous.

Genitalia morphology. Male genitalia. Uncus long and moderately to very slender; gnathos hook bulky, strongly sclerotised, particularly at apex, with longitudinal grooves, straight;

anterior margin of tegumen with strongly sclerotised edge, reversed V-shaped; valva straight, apically distorted, clubbed; saccular area densely covered with setae, without or with short sacculus; saccus broadly sub-triangular, posterior margin projected with medial indentation, sclerotised medial ridge of various length; phallus stout, almost straight, with moderately inflated coecum, distal two-thirds gradually tapered, predominantly with spiny dorsal zone, exceptionally with large spine, ductus ejucalatorius twirled, with contorted linear interior sclerotisation.

Female genitalia. Papilla analis small; apophysis posterior very long; segment VIII long and slender, largely membranous; subgenital plate with strongly sclerotised sub-triangular subostial sclerotisation, anteromedially with broadly convex projection; apophysis anterior rod-like, from posterior margin of segment VIII; colliculum strongly sclerotised; signum small, rounded spiny plate.

Diagnosticremarks. The *M.pusillus* species group is characterised by unique structures, in particular the massive and bulky gnathos hook with longitudinal grooves is highly diagnostic. Furthermore the combination of characters such as the distorted and clubbed apex of the valva, the strongly setose saccular area with a tendency for separation of the sacculus and the medial ridge of the saccus are important characters. Female genitalia differ from other species groups having an overall similarity by the absence of posteriorly pointed sclerites of the subgenital plate. However, females are unknown for the vast majority of species and the diagnostic value of this character is therefore unknown.

#### Megacraspedus tristictus

Taxon classificationAnimaliaLepidopteraGelechiidae

Walsingham, 1910

Megacraspedus tristictus Walsingham, 1910: 231.

##### Examined material.

**Holotype** ♂, [France] “Holo-type” “Cannes S FRANCE 22.V.1892 Wlsm. 81169” “Type” “Walsingham Collection, 1910–427.” “Megacraspedustristictus ♂ Wlsm. Ent. Mo. Mag. 46.231-2 sp. 3009.1 (1910) TYPE ♂ (f) deser 81169” (BMNH). **Non-type material.** France. 2 ♂, Dep. Alpes Maritimes, S of Tende, Saorge, 482 m, 3–4.vii.2008, leg. O. Karsholt (ZMUC); 1 ♂, same data, but 6.vi.2009, leg. P. Huemer; 1 ♂, same data, but 7.vi.2009, leg. P. Huemer, genitalia prep. (in glycerin); 1 ♂, Alpes Maritimes, Les Ferres, 3.vii.1971, leg. F. Dujardin; 1 ♂, Dep. Alpes Maritimes, St. Barnabé, 7.vi.1970, leg. F. Dujardin; 1 ♂, Dep. Alpes Maritimes, Èze, Fort de la Revere, 700 m, 23.vi.1995, leg. J. Nel, genitalia slide 4105 Nel (all TLMF); 1 ♂, Dep. Alpes Maritimes, Col de Vence 11–12.vi.1981, leg. Hahn (RCEA); 1 ♂, Dep. Alpes Maritimes, Gourdon, late vi.1968, leg. F. Zürnbauer, genital slide GEL 1185 Huemer (TLMF); 1 ♂, same data, but 13.v.1994, leg. Petru (NMPC); 1 ♂, Dep. Drôme, Col de l’Homme Mort, 1212 m, 17.vi.1982, leg. C. Gibeaux & Lovenu (ZMUC); 2 ♂, Dep. Var, Puits de Rians, 27.v.2000, leg. J. Nel, genitalia slide 11295 Nel; 2 ♂, Dep. Var, Puits de Rians, la Gardiole, 3.vi.2000, leg. J. Nel; 1 ♂, same data, but 19.vi.2001, leg. J. Nel; 1 ♂, same data, but 4.vi.1995, leg. J. Nel, genitalia slide 3819 Nel; 1 ♂, same data, but 30.v.1999, leg. J. Nel; 1 ♂, Dep. Var, Puits de Rians, la Plarée, 31.v.2004, leg. J. Nel; 1 ♂, Dep. Var, Mt. Caume, l´ubac, 750 m, 5.vi.2000, leg. J. Nel; 1 ♂, Dep. Var, Mt. Caume, ouest, 740 m, 29.v.1994, leg. J. Nel, genitalia slide 2064 Nel; 1 ♂, Dep. Var, Draguignan, 6.vi.1993, leg. J. Nel, genitalia slide 1170 Nel; 1 ♂, same data, but 31.v.1992, leg. J. Nel, genitalia slide 2543 Nel (all TLMF); 1 ♂, Dep. Var, Frejus, 20.v.1998, leg. H. K. Jensen (ZMUC). Italy. 1 ♂, prov. Savona, Conna, 300 m, 28.vi.1976, leg. G. Baldizzone (ZMUC).

##### Redescription.

Adult. *Male* (Figure 48). Wingspan 10–13 mm. Segment 2 of labial palpus with moderately long scale brush, light brown on outer surface, white mottled with brown on inner surface, white on lower and upper surface; segment 3 white with black tip. Antennal scape without pecten; flagellum light brown ringed with black. Head very light brown; thorax and tegula yellowish brown. Forewing light brown, from dorsum to fold yellowish brown, costa whitish brown apart from dark base; a black dot in fold at 2/5 and two black dots at 3/5 in middle of wing and at end of cell; a few blackish scales along termen; fringes light grey. Hindwing light grey with concolorous fringes.

*Female*. Unknown.

*Variation*. There is some variation in size, including among specimens from the same locality.

*Male genitalia* (Figure 183). Uncus moderately slender, about one-third times longer than maximum basal width, weakly tapered, apical edge evenly rounded; gnathos hook bulky, strongly sclerotised, particularly at apex, with longitudinal grooves, straight, approximately 1.5 times length of uncus, apically pointed; anterior margin of tegumen with deep V-shaped emargination, suboval pedunculi small; valva straight, stout, extending to about middle of uncus, distorted apical part rounded; saccular area densely covered with setae, without separated sacculus; posterior margin of vinculum with deep U-shaped medial emargination, broadly rounded lateral humps, suboval vincular sclerites with sclerotised posterior edge; saccus broadly sub-triangular, abruptly tapered at about two-thirds, ratio maximum width to length about 1, posterior margin with pointed mediolateral projections, separated by moderately deep incision, medial part with strongly sclerotised longitudinal ridge extending nearly to apex of saccus, lateral sclerites slightly shorter than maximum width of saccus; phallus slightly shorter than tegumen, almost straight, with moderately inflated coecum, distal two-thirds gradually tapered, ventrally with sclerotised ridge, dorsomedial area broadly sclerotised, ductus ejucalatorius twirled, with contorted linear interior sclerotisation.

*Female genitalia*. Unknown.

##### Diagnosis.

*Megacraspedustristictus* is characterised by its light yellowish brown forewings with three distinct black dots. It resembles *M.pentheres* (Figure 130). *M.faunierensis* sp. n. (Figure 40) differs in having the head white and the antennae darker. The male genitalia are similar overall to other species of the *M.pusillus* species group but differ e.g., by the distinctly shorter uncus.

##### Molecular data.

BIN BOLD:ADA0606 (n = 1). The distance to the nearest congeneric neighbour *M.tenuiuncus* sp. n. is 12% , the distance to the nearest BIN in BOLD, *Emargineacombusta* (Noctuidae), is 10.3% (p-dist).

##### Distribution.

South-eastern France, north-western Italy.

##### Biology.

Host plant and early stages are unknown. The adults have been collected from the middle of May to early July at altitudes from 300 to 1200 m.

##### Remarks.

*Megacraspedustristictus* was described from one male collected by Walsingham at Cannes, SE France, 22.v.1892 (Walsingham 1910).

#### Megacraspedus alfacarellus

Taxon classificationAnimaliaLepidopteraGelechiidae

Wehrli, 1926

Megacraspedus alfacarellus Wehrli, 1926: 163.

##### Examined material.

**Holotype** ♂, “Type” [Spain, prov. Granada] “Sierra Alfacar 18/19 VI 25 Dr. E. Wehrli, Basel” “*alfacarellus* Sa. Alfacar ♂ + 18.VI.25. W. ” “GU 16/1414 ♂ P. Huemer” (NHMB). **Non-type material.** Spain. 1 ♂, prov. Granada, Sierra de Alfacar, 24.vi.1880, coll. Staudinger; 1 ♂, Granada, coll. Staudinger (all ZMHU).

##### Redescription.

Adult. *Male* (Figs 49–50). Wingspan 16 mm. Segment 2 of labial palpus with long scale brush, dark brown on outer surface, white mottled with brown on inner surface, white on lower and upper surface; segment 3 whitish brown. Antennal scape without pecten; flagellum black. Head whitish brown with white neck; thorax and tegula as forewing. Forewing dark brown; a yellow line in fold ending in an elongate black dot; a small black dot in fold and at end of cell; fringes grey. Hindwing grey with light grey fringes.

*Female*. Unknown.

*Variation*. The limited material available so far shows no variation.

*Male genitalia* (Figs 184–185). Uncus slender, about two times longer than maximum basal width, evenly tapered towards weakly pointed apex; gnathos hook bulky, with longitudinal grooves, straight, slightly longer than uncus, apically strongly sclerotised, pointed; anterior margin of tegumen with deep V-shaped emargination, suboval pedunculi distinct; valva straight, stout, extending to about middle of uncus, distorted apical part rounded; saccular area densely covered with setae, without separated sacculus; posterior margin of vinculum with deep U-shaped medial emargination, broadly rounded lateral humps, suboval vincular sclerites with sclerotised posterior edge; saccus sub-triangular, apically abruptly tapered, rod-like, ratio maximum width to length approximately 0.6, posterior margin with pointed mediolateral projections, separated by moderately deep incision, medial part with strongly sclerotised longitudinal ridge extending to anterior part of saccus, with or without forked anterior end, lateral sclerites about length of maximum width of saccus; phallus slightly shorter than tegumen, almost straight, with moderately inflated coecum, distal two-thirds gradually tapered, ventral margin with sclerotised ridge, dorsomedial area with long row of spines, ductus ejucalatorius twirled, with contorted linear interior sclerotisation.

*Female genitalia*. Unknown.

##### Diagnosis.

*Megacraspedusalfacarellus* is characterised by its black antennae, and by the dark brown forewings with two weak black dots. It is similar to some forms of *M.spinophallus* sp. n. (Figs 53–54) and *M.bidentatus* sp. n. (Figure 44). The male genitalia are very similar to other species of the *M.pusillus* species group and mainly differ in the distally tapered phallus and the row of dorsomedial spines; they are only weakly separated from *M.skoui* sp. n. (Figure 52) by the larger uncus and the less pointed apex of the saccus.

##### Molecular data.

Not available, no suitable specimen was available for barcoding.

##### Distribution.

Southern Spain (prov. Granada).

##### Biology.

Host plant and early stages are unknown. The few adults known to date have been collected in the second half of June at unreported altitudes.

##### Remarks.

*Megacraspedusalfacarellus* was described from one male collected between 18–19.vi.1925 in Sierra Alfacar, Andalusia, Spain (Wehrli 1926).

#### Megacraspedus pusillus

Taxon classificationAnimaliaLepidopteraGelechiidae

Walsingham, 1903

Megacraspedus pusillus Walsingham, 1903: 266.

##### Examined material.

**Paratype** ♂, “Sierra Nevada Granada ; SPAIN 3 VI 1901 Wlsm 86728” “Paratype” “*Megacraspedus* ♂ *pusillus* WLSM 2/3” “Walsingham Collection, 1910-427.”, genitalia slide 33661 (BMNH). **Non-type material.** Spain. 1 ♂, prov. Granada, Sierra Nevada, El Parador NP, 2500 m, 21.vii.1980, leg. E. Traugott-Olsen, genitalia slide 5621 Traugott-Olsen; 1 ♂, prov. Granada, Sierra Nevada, Camino de Valeta, 1600 m, 13.vi.1986, leg. E. Traugott-Olsen; 1 ♂, prov. Granada, Sierra Nevada, 3.4 km SSE Güejar Sierra, 1830 m, 4.vii.2015, leg. J. Tabell, genitalia slide 16/1432 Huemer; 1 ♂, prov. Almería, Sierra de los Filabres, Calar Alto, 2130 m, 5.vii.2015, leg. J. Tabell (all ZMUC).

##### Redescription.

Adult. *Male* (Figure 51). Wingspan 12–13 mm. Segment 2 of labial palpus with scale brush of about length of segment 3, dark brown on outer and inner surface, white on lower and upper surface; segment 3 cream-white with brown tip. Antennal scape without pecten; flagellum dark brown. Head whitish brown with white neck; thorax and tegula as forewing. Forewing light grey-brown, darkest at base of costa; a narrow yellow line in fold with an indistinct elongate black dot; a distinct black dot at end of cell; fringes light grey. Hindwing grey with light grey fringes.

*Female*. Unknown.

*Variation*. The black dot in the fold is absent in some specimens.

*Male genitalia* (Figs 186–187). Uncus moderately slender, approximately 1.7 times longer than maximum basal width, evenly tapered towards rounded apex; gnathos hook bulky, with longitudinal grooves, straight, slightly longer than uncus, apically strongly sclerotised, pointed; anterior margin of tegumen with deep V-shaped emargination, rounded pedunculi distinct; valva straight, moderately broad, extending slightly beyond base of gnathos, distorted apical part rounded; saccular area densely covered with setae, without separated sacculus; posterior margin of vinculum with deep medial emargination, broadly rounded lateral humps, suboval vincular sclerites with sclerotised posterior edge; saccus sub-triangular, with rod-like apex, ratio maximum width to length approximately 0.6, posterior margin with pointed mediolateral projections, separated by shallow incision, medial part with strongly sclerotised longitudinal ridge extending to middle of saccus, with weakly forked anterior end, lateral sclerites slightly longer than maximum width of saccus; phallus about length of tegumen, weakly curved, with weakly inflated coecum, distal two-thirds stout, with rod-like ventral sclerite extending to apex, broader and shorter dorsal sclerotisation with few minute teeth, ductus ejaculatorius with long and slender band-like interior sclerotisation.

*Female genitalia*. Unknown.

##### Diagnosis.

*Megacraspeduspusillus* is characterised by its light grey-brown forewings with a distinct black dot at end of the cell. It is particularly similar to *M.skoui* sp. n. (Figure 52), but also to *M.bidentatus* sp. n. (Figure 44) and *M.fuscus* sp. n. (Figure 45). The male genitalia differ from those of *M.skoui* sp. n. (Figure 188) and *M.spinophallus* sp. n. (Figs 189–190) in the shallow medial incision of the posterior margin of the saccus and the shorter and less forked medial ridge, and the absence of a distinct row of dorsal and ventral teeth of the phallus.

##### Molecular data.

BIN BOLD:ACZ8007 (n = 1). The distance to the nearest neighbour *M.spinophallus* sp. n. is 8.4% (p-dist).

##### Distribution.

Southern Spain (prov. Granada and Almería).

##### Biology.

Host plant and early stages are unknown. The few adults known to date have been collected from the middle of June to the second half of July, at altitudes from between 1600 and 2500 m.

##### Remarks.

*Megacraspeduspusillus* was described from four males collected in the Sierra Nevada on 2.vi.1901 (Walsingham 1903: 266).

#### Megacraspedus skoui sp. n.

Taxon classificationAnimaliaLepidopteraGelechiidae

http://zoobank.org/EE8D21DA-7245-451B-9FB3-579CB3C8B609

##### Examined material.

**Holotype** ♂, “Spain, [prov.] Granada, Sierra Nevada, Camino del Veleta 1650 m, 25.VI.1986, leg. Peder Skou” “Gen. prep. 5318 ♂ O. Karsholt” (ZMUC). **Paratypes.** Spain. 3 ♂, prov. Granada, Puerto de la Mora, 1300 m, 6.vi.1974, leg. W. Glaser, genitalia slide 5319 Karsholt (SMNK, ZMUC); 1 ♂, prov. Granada, 2 km W Diezma, 1400 m, 9–10.vii.2010, leg. Z. Tokár, genitalia slide GU 16/1405 ♂ Huemer (RCZT).

##### Description.

Adult. *Male* (Figure 52). Wingspan 11–13 mm. Segment 2 of labial palpus with scale brush almost as long as segment 3, brown on outer surface, brown mottle with white on inner surface, white on upper and lower surface; segment 3 longer than segment 2, black with white tip. Antennal scape without pecten; flagellum black, indistinctly ringed with light brown. Head, thorax and tegula cream-white mottled with light grey-brown. Forewing light grey-brown; base of costa and apex darker; fringes grey. Hindwing grey with grey fringes.

*Female*. Unknown.

*Variation*. One specimen has an indistinct black dot in the fold of the forewings.

*Male genitalia* (Figure 188). Uncus moderately slender, 1.7 times longer than maximum basal width, evenly tapered towards rounded apex; gnathos hook bulky, with longitudinal grooves, straight, slightly longer than uncus, apically strongly sclerotised, pointed; anterior margin of tegumen with deep V-shaped emargination, rounded pedunculi distinct; valva straight, moderately broad, extending slightly beyond base of gnathos, distorted apical part rounded; saccular area densely covered with setae, without separated sacculus; posterior margin of vinculum with deep medial emargination, broadly rounded lateral humps, suboval vincular sclerites with sclerotised posterior edge; saccus sub-triangular, with rod-like apex, ratio maximum width to length 0.7, posterior margin with pointed mediolateral projections, separated by moderately deep V-shaped incision, medial part with strongly sclerotised ridge strongly forked in medial part of saccus, lateral sclerites slightly longer than maximum width of saccus; phallus about length of tegumen, weakly curved, with weakly inflated coecum, distal two-thirds stout, ventral margin with sclerotised ridge extending to apex, weakly dentated near base, broader and shorter dorsal sclerotisation with row of minute teeth extending over entire length, ductus ejaculatorius with long and slender band-like interior sclerotisation.

*Female genitalia*. Unknown.

##### Diagnosis.

*Megacraspedusskoui* sp. n. is characterised by its almost unicolorous light grey forewings. It resembles several smaller, unicolorous *Megacraspedus* species, especially *M.pusillus*, which has a distinct black dot at end of the cell (Figs 186–187). The male genitalia are very similar overall to other species of the *M.pusillus* species group and differ in particular in the characteristic dentations of the phallus. In this character the species resembles *M.alfacarellus* (Figs 184–185) but differs in the smaller uncus and some minute details such as the longer rod-like apex of the saccus.

##### Molecular data.

BIN BOLD:ACT1624 (n = 1). The distance to the nearest neighbour *M.spinophallus* sp. n. is 7.97% (p-dist).

##### Distribution.

Spain (prov. Granada).

##### Biology.

Host plant and early stages are unknown. Adults have been collected in June and July at altitudes between 1300 and 1650 m.

##### Etymology.

The species name (a noun in the genitive case) is dedicated to Peder Skou, Denmark, who collected the holotype of this species and numerous other *Megacraspedus* specimens used for our study.

#### Megacraspedus spinophallus sp. n.

Taxon classificationAnimaliaLepidopteraGelechiidae

http://zoobank.org/C8F2ACC1-6BD5-4D86-8A3B-3734FC6C6712

##### Examined material.

**Holotype** ♂, “Spanien, [prov.] Alicante Sierra de Crevillente 5 km NE Albatera, 450 m, 38°15,22'N, 00°54,86'W 26.5.2004 leg. Huemer TLMF 2005-04” “BC TLMF Lep 03227” “P. Huemer GEL 1208 ♂” (TLMF). **Paratypes.** Spain. 8 ♂, same data as holotype (TLMF); 2 ♂, prov. Alicante, Rebate, 26.vi.1989, leg. B. Å Bengtsson, genitalia slide Bengtsson 3268 (RCBB, ZMUC); 2 ♂, prov. Alicante, 4 km E Aspe, Rio Vialopo, 300 m, 24.v.1998, leg. P. Skou (ZMUC); 2 ♂, prov. Alicante, 8 km N Albatera, 300 m, leg. J. Šumpich (NMPC); 1 ♂, prov. Alicante, Sierra Alta, Aitana, 1200 m, 18.vi.2011, leg. H. Rietz; 1 ♂, prov. Alicante, Parcent, 8.vi.2014, leg. H. Rietz; 1 ♂, prov. Alicante, Beniarbeig, 16.vi.2015, leg. H. Rietz; 1 ♂, prov. Alicante, Pto de la Carresqueta, 1050 m, 24.vi.2015, leg. H. Rietz (all ECKU); 1 ♂, prov. Alicante, Alcoj, Font Roja, 27.vi.2010, leg. A. Stübner (TLMF); 1 ♂, prov. Almería, 5 km SW Tabernas, Rambla de Tabernas, 200 m, 28.v.1998, leg. P. Skou; 1 ♂, prov. Almería, 10 km E Bedar, El Pinar, 325 m, 19–27.iv.2001, leg. P. Skou & B. Skule; 3 ♂, prov. Almería, El Pozo del Esparto, 20 m, 22–26.iv.2001, leg. C. Hviid, P. Skou & B. Skule (all ZMUC); 2 ♂, 3 km SW Pulpi, 200 m, 2.vi.2002, leg. W. Schmitz (RCWS); 1 ♂, prov. Almería, Tabernas, 380 m, 6–8.vii.2007, leg. G. Jeppesen, genitalia slide 6490 Hendriksen) (ZMUC); 1 ♂, prov. Almería, Tabernas env., Aghuilla Salada, 550 m, 7.vii.2010, leg. Z. Tokár; 1 ♂, prov. Almería, Sierra de Alhamilla, Huebro, 700–800 m, 29.iv.2008, leg. Z. Tokár (all RCZT); 6 ♂, same data, but leg. J. Šumpich; Sierra de Alhamilla, vicinity of Huebro, 700–800 m, 29.iv.2008, leg. J. Šumpich; 1 ♂, prov. Almería, Sierra Cabrera, Mojácar, 50 m, 4.v.2008, leg. J. Sumpich; 25 ♂, prov. Almería, Sierra de Alhamilla, Turrillas env., route Colativi, 1000 m, 15–19.vi.2007, leg. J. Šumpich; 8 ♂, Sierra de Alhamilla, vicinity of Níjar, 560 m, 29.iv.2008, leg. J. Šumpich (all NMPC); 1 ♂, prov. Almeria, Nijar, Huebro, 10.v.2014, leg. A. Stübner (ZSM); 1 ♂, prov. Castellon, 5 km E Cuevas de Vinroma, 200 m, 13.vii.1992, leg. M. Fibiger; 16 ♂, prov. Castellon, 25 km NW Castellon, La Banderetta pass, 800 m, 17.vii.1992, leg. M. Fibiger, genitalia slide 6521 Hendriksen, 15/1403 Huemer (ZMUC); 1 ♂, 1 ♀, prov. Granada, valley of Rio Guadelfo, South side, E of Orgiva, 1000 m, 5.vii.1969, leg. K. Sattler & D.J. Carter, gen. slides 33659 ♂, 33660 ♀ (BMNH); 1 ♂, prov. Huesca, 6 km SW Candasnos, Barranco de Valcuerna, 300 m, 15.vi.1999, leg. P. Skou; 1 ♂, same data, but 8 km S Candasnos, 175 m, 5.vi.2002, leg. B. Skule (all ZMUC); 2 ♂, prov. Huesca, 6 km W Ontiñena, 7.vi.2002, leg. W. Schmitz; 1 ♂, prov. Huesca, 10 km S Candasnos, 300 m, 13.vi.2004, leg. W. Schmitz; 4 ♂, prov. Huesca, Ontiñena, 300 m, 28.v.2015, leg. J. Viehmann (all RCWS); 1 ♂, prov. Huesca, 10 km SW Candasnos, 30.v.2015, leg. J. Viehmann (ZMUC); 5 ♂, prov. Huesca, Los Monegros, La Zaida, 19.v.2004, leg. J. Junnilainen; 2 ♂, same data, but 10 km NW Gelsa, 21.v.2004, leg. J. Junnilainen (all RCJJ); 2 ♂, prov. Lleida, 30 km NW Fraga, Ontinema, 250 m, 11.vii.1992, leg. M. Fibiger; 1 ♂, prov. Málaga, Camino de Ojen, 150 m, 21.vi.1980, leg. E. Traugott-Olsen; 1 ♂, same data, but 8.vi.1986; 1 ♂, prov. Málaga, Sierra de Marbella, 14.vii.1980, leg. E. Traugott-Olsen; 1 ♂, prov. Valencia, Porta Coeli, 13.vii.1988, leg. J. Baixeras & M. Domingues (all ZMUC); 2 ♂, prov. Valencia, Villargordo del Cabriel, 20.vi.2010, leg. A. Stübner; 3 ♂, same data, but 19.vi.2010; 1 ♂, same data, but 21.vi.2010 (all ZSM); 1 ♂, 1 ♀, prov. Zaragoza, Belchite, 350 m, 3.vii.2004, leg. J. Procházka (NMPC).

##### Description.

Adult. Male (Figure 53). Wingspan 11–20 mm. Segment 2 of labial palpus with long scale brush, dark brown on outer surface, white mottled with brown on inner surface, white on lower and upper surface; segment 3 white with some black towards tip. Antennal scape without pecten; flagellum black. Head whitish brown with white neck; thorax and tegula as forewing. Costal half and apical part of forewing dark brown; dorsal part light brown, darker towards dorsum; elongate black dots in fold at 1/3 and at end of cell; a few black scales at tornus; fringes light brownish grey. Hindwing grey with light grey fringes.

Female (Figure 54). Wingspan 19 mm. Forewing light yellow-brown, darker towards costa and especially at base of costa. An indistinct black dot at end of cell. Hindwing more slender than in male. Otherwise similar to male.

Variation. Highly variable in size, with specimens from southern Spain being generally larger. The colour of the head varies from almost cream coloured to grey-brown, and thorax and tegula vary accordingly. In some specimens the forewing (apart from the fold) is almost plain brown. One specimen has an indistinct black streak in the middle of the forewing. One of the two female specimens has no black dot in the fold.

Male genitalia (Figs 189–190). Uncus slender, about two times longer than maximum basal width, evenly tapered towards rounded apex; gnathos hook bulky, with longitudinal grooves, straight, slightly longer than uncus, apically strongly sclerotised, pointed; anterior margin of tegumen with deep V-shaped emargination, suboval pedunculi distinct; valva straight, stout, extending to about middle of uncus, distorted apical part rounded; saccular area densely covered with setae, without separated sacculus; posterior margin of vinculum with deep U-shaped medial emargination, broadly rounded lateral humps, suboval vincular sclerites with sclerotised posterior edge; saccus sub-triangular, apically abruptly tapered, rod-like, ratio maximum width to length 0.6, posterior margin with pointed mediolateral projections, separated by moderately deep incision, medial part with strongly sclerotised longitudinal ridge extending to anterior part of saccus, with distinctly forked anterior end, lateral sclerites about length of maximum width of saccus; phallus slightly shorter than tegumen, almost straight, with moderately inflated coecum, distal two-thirds gradually tapered, ventral margin with sclerotised ridge, dorsomedial area with large group of spines, ductus ejucalatorius twirled, with contorted linear interior sclerotisation.

Female genitalia (Figure 276). Papilla analis small, apically rounded; apophysis posterior slender rod-like, long, with short, bifurcate posterior end, bordered by sclerotised field; segment VIII approximately 1.5 mm long, membranous; subgenital plate with sub-triangular subostial sclerotisation, without pointed sclerites posteriorly, posteromedially broadly merged with medial flaps delimiting rounded ostium bursae, anterior margin with rod-like edge connected with apophysis anterior, medially with rounded projection; apophysis anterior slender, rod-like, about length of segment VIII, posteriorly becoming rod-like venula of segment VIII, extending to posterior margin; colliculum short, strongly sclerotised; ductus bursae short, broad; corpus bursae moderately short and broad, weakly delimited from ductus bursae; signum small, rounded spiny plate.

##### Diagnosis.

*Megacraspedusspinophallus* sp. n. is characterised by its black antennae, and by the dark brown forewings having two elongate black dots and being yellowish in dorsal half. It is very similar to *M.alfacarellus* (Figs 49–50), but that species has more plain dark brown forewings (apart from the yellow fold) with two small black dots. The male genitalia are very similar to other species of the *M.pusillus* species group but differ in the characteristic long and furcated medial ridge of the saccus and particularly the large field of spines of the phallus. The female genitalia are similar overall to several taxa in other species groups but differ in the subgenital plate without pointed sclerites. However, it remains unknown if this character is shared with other species in the *M.pusillus* species group.

##### Molecular data.

BIN BOLD:AAU1828 (n = 7), BIN BOLD:ACT2894 (n = 1). Genetically variable species. The intraspecific divergence of the barcode region is large and reflected by 2 BINs with 2.8% maximum divergence, based on a single specimen compared with a larger cluster. Within the latter average divergence is only 0.2% and maximum divergence is 0.6% (n = 2). The minimum distance to the nearest neighbour *M.skoui* sp. n. is 8% (p-dist).

##### Distribution.

Spain.

##### Biology.

Host plant and early stages are unknown. The adults have been collected from late April to the middle of July at altitudes from sea level to 1200 m.

##### Etymology.

The species name is a compound word derived from the Latin adjective *spinosus* (meaning spiny) and noun *phallus*, referring to the spiny phallus. The name is a noun in apposition.

#### Megacraspedus occidentellus sp. n.

Taxon classificationAnimaliaLepidopteraGelechiidae

http://zoobank.org/7F60E509-29B6-4D15-9792-E6D739B6B712

##### Examined material.

**Holotype** ♂, “Portugal, Ext. [=prov. Estremadura] Ericeira, 30 m 4.-8.vii.1986 O. Karsholt” “GU 13/1352 ♂ Huemer” (ZMUC). **Paratypes.** Portugal. 3 ♂, same data as holotype, but genitalia slide 6520 Hendriksen (TLMF, ZMUC); 1 ♂, Estremadura, 3 km E Capo da Roca, 200 m, 8.vii.1986, leg. O. Karsholt (ZMUC).

##### Description.

**Figures 55–60.**
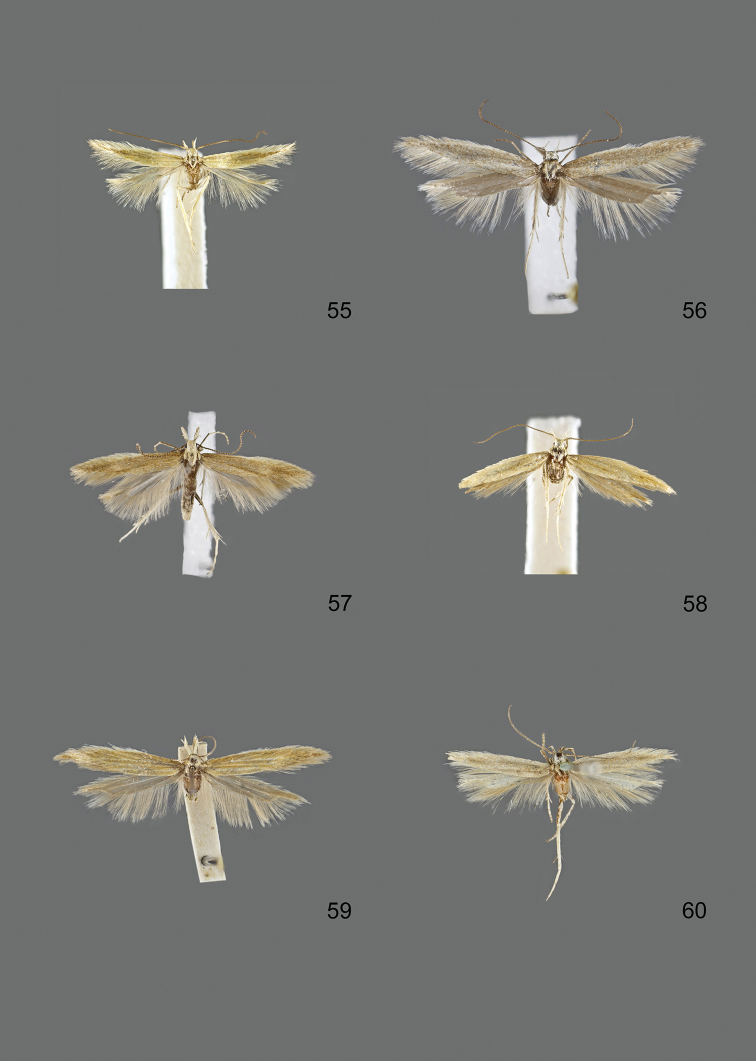
*Megacraspedus* adults in dorsal view. **55***M.occidentellus* sp. n. – Holotype, male, Portugal (ZMUC) **56***M.granadensis* sp. n. – Holotype, male, Spain (ZMUC) **57***M.heckfordi* sp. n. – Paratype, male, Spain (TLMF) **58***M.tenuiuncus* sp. n. – Paratype, male, Spain (ZMUC) **59***M.lativalvellus* Amsel, 1954 – Paratype, male, Malta (LNK) **60***M.dejectella* (Staudinger, 1859) – Lectotype, male, Spain (ZMHU).

Adult. Male (Figure 55). Wingspan 9–12 mm. Segment 2 of labial palpus with moderately long scale brush, brownish on outer surface and on apical half of inner surface, otherwise white; segment 3 white mottled with blackish brown on lower and outer surface and with black tip. Antennal scape without pecten; flagellum dark brown, indistinctly ringed with black. Head, thorax and tegula as forewing. Dorsal half of forewing light yellow, costal half light brown, extending into a dark brown apical streak; costa whitish; fringes light grey. Hindwing grey with light grey fringes. Female. Unknown.

Variation. The few examined specimens show a slight variation in the forewing colour which, however, may be due to a more or less fresh condition.

Male genitalia (Figure 191). Uncus slender digitate, about 3 times longer than maximum basal width, apex rounded; gnathos hook bulky, with longitudinal grooves, slightly longer than uncus, straight, apically strongly sclerotised, pointed; anterior margin of tegumen with deep V-shaped emargination, pedunculi small, drop-shaped; valva straight, stout, extending slightly beyond middle of uncus, distorted apical part rounded; saccular area densely covered with setae, with weakly separated sacculus; posterior margin of vinculum with U-shaped emargination, broadly rounded lateral humps, suboval vincular sclerites with sclerotised posterior edge; saccus semi-oval, apically tapered, about same length and width, posterior edge weakly arched, with shallow medial incision, medial part with strongly sclerotised furcated longitudinal ridge, lateral sclerites shorter than maximum width of saccus; phallus stout, inflated coecum about twice width and almost length of distal part, sclerotised ridge in distal part without group of spines medially, ductus ejucalatorius with contorted linear interior sclerotisation.

Female genitalia. Unknown.

##### Diagnosis.

*Megacraspedusoccidentellus* sp. n. is a rather uncharacteristic *Megacraspedus* which can be recognized by lacking black dots and whitish coloured veins on the forewing. It is similar, e.g., to the slightly larger *M.trineae* sp. n. (Figure 46), but can be recognized by its more uniformly coloured dark antennae, and by having the forewing colour separated by a lighter dorsal part and a darker costal part, the latter extending into a dark brown apical streak. The male genitalia differ from other species of the *M.pusillus* species group in having a slender uncus and in particular the posteriorly deeply incised saccus and the stout phallus with a comparatively large coecum.

##### Molecular data.

Not available, barcoding failed.

##### Distribution.

Western Portugal.

##### Biology.

Host plant and early stages are unknown. The type series was collected in early July at low altitudes.

##### Etymology.

This species is named after its occurrence at the most western part of continental Europe. The species name is derived from a combination of the Latin *occidentalis* (meaning western) and the diminutive suffix –*ellus*. The name is a compound noun.

#### Megacraspedus granadensis sp. n.

Taxon classificationAnimaliaLepidopteraGelechiidae

http://zoobank.org/FD13E3A1-F8F4-4859-8D64-1571610ED899

##### Examined material.

**Holotype** ♂, “Hispania. [prov. Granada] Andalucia. Sierra Nevada. Cam.d.Valeta 1600 m 14.6.1986 E. Traugott-Olsen” “GU 16/1458 ♂ P. Huemer” (ZMUC). **Paratypes.** Spain. 1 ♂, prov. Granada, Sierra Nevada, Camino de Valeta, 1600 m, 12.vi.1986, leg. E. Traugott-Olsen; 2 ♂, same data, but 13.vi.1986; 4 ♂, same data, but 14.vi.1986, genitalia slide 6535 Hendriksen (ZMUC).

##### Description.

Adult. Male (Figure 56). Wingspan 13–16 mm. Segment 2 of labial palpus with scale brush about length of segment 3, brown on outer surface, brown mottled with white on inner surface, white on lower and upper surface; segment 3 about same length as segment 2, white mottled with black especially on lower surface and at tip. Antennal scape without pecten; flagellum indistinctly ringed black and brownish. Head light brown mottled with white; thorax cream coloured slightly mottled with brown; tegula brown at base, whitish towards apex. Forewing light grey-brown from whitish brown- and black-tipped scales, lighter in dorsal part below fold; costa blackish brown at base, otherwise greyish white; fold indistinctly light yellowish; some black scales along termen; fringes light grey. Hindwing dark grey with concolorous fringes.

Female. Unknown.

Variation. The yellowish streak in the fold can be more or less distinct.

Male genitalia (Figure 192). Uncus evenly slender, digitate, about four times longer than broad, basally slightly widened, apically converged; gnathos hook massive, with longitudinal grooves, slightly longer than uncus, basally about two-third width, evenly tapered with pointed apex; anterior edge of tegumen with deep suboval emargination; pedunculi suboval; valva extending to about base of uncus, broad, apical sixth abruptly tapered, distorted, with rounded apex; saccular area densely covered with setae, without clearly separated sacculus; posterior margin of vinculum medially emarginated, laterally with hump, suboval vincular sclerites with sclerotised posterior and proximal edge; saccus sub-triangular, approximately 1.4 times longer than broad, posterior edge weakly sinusoid, medial part with strongly sclerotised ridge extending from posterior edge to apex, lateral sclerites slightly shorter than maximum width of saccus; phallus with weakly inflated coecum, about one-quarter length of broadly digiate and weakly curved distal part, medial part with large group of small sclerotised knobs, apex rounded, ductus ejucalatorius with two contorted linear interior sclerotisations.

Female genitalia. Unknown.

##### Diagnosis.

*Megacraspedusgranadensis* sp. n. is characterised in having the forewings covered with whitish brown- and black-tipped scales, in having the costa blackish brown at base and whitish grey in middle, and having no black dots. Also the dark grey hindwings are characteristic. The male genitalia differ from the particularly similar *M.spinophallus* sp. n. (Figs 189–190) in the knob-like sclerites of the phallus which is furthermore of a different shape.

##### Molecular data.

Not available, barcoding failed.

##### Distribution.

Spain (prov. Granada).

##### Biology.

Host plant and early stages are unknown. The adults have been collected in the middle of June at an altitude of 1600 m.

##### Etymology.

This species is named after its place of occurrence: the province of Granada in southern Spain. The name is a masculine adjective.

#### Megacraspedus heckfordi sp. n.

Taxon classificationAnimaliaLepidopteraGelechiidae

http://zoobank.org/137B14A8-5568-4407-A5F7-1111B44A3F5E

##### Examined material.

**Holotype** ♂, “ESPANA, Prov. Cantabria PN Picos de Europa Fuente De, El Cable Bergst. 4°48,53'W, 43°09,55'N 1870 m, 11.7.2012 leg. Huemer TLMF 2012-011” “BC TLMF Lep 08326” (TLMF). **Paratypes.** Spain. 1 ♂, same data as holotype; 7 ♂, same data, but TLMF 2013-011 (all TLMF); 30 ♂, same data, but leg. T. Mayr (RCTM); 1 ♂, Prov. Cantabria, PN Picos de Europa, above Fuente Dé, 1780 m, 9.vii.1999, leg. R. J. Heckford, genitalia slide 949 Heckford (RCRH); 21 ♂, prov. Cantabria, PN Picos de Europa, Espinama, Portillas des Poqueion, 1340 m, 11.vii.2012, leg. P. Huemer; 2 ♂, prov. Leon, Picos de Europa, Portilla de la Reina env., 1230 m, 9.vii.2012, leg. P. Huemer, genitalia slide GEL 1205 Huemer (RCTM, TLMF, ZMUC).

##### Description.

Adult. Male (Figure 57). Wingspan 12–14 mm. Segment 2 of labial palpus with scale brush as long as segment 3, grey-brown on outer surface, brown mottled with white on inner surface, white on lower and upper surface; segment 3 as long as segment 2, white with black tip. Antennal scape without pecten; flagellum black, indistinctly ringed with grey-brown. Head, thorax and tegula cream-white. Forewing light greyish brown from light brown dark-tipped scales; costa blackish brown at base, otherwise whitish; fringes light grey. Hindwing grey with grey fringes.

Female. Unknown.

Variation. The examined specimens show only slight variation.

Male genitalia (Figure 193). Uncus evenly slender, digitate, about four times longer than broad, apex rounded; gnathos hook massive, with longitudinal grooves, slightly longer than uncus, basally same width, evenly tapered to pointed apex; anterior edge of tegumen with deep suboval emargination; pedunculi sub-triangular, posteriorly with sclerotised ridge; valva extending to about middle of uncus, broad, apical sixth abruptly tapered, distorted, with rounded apex; saccular area densely covered with setae, without clearly separated sacculus; posterior margin of vinculum medially emarginated, laterally with hump, suboval vincular sclerites with sclerotised posterior edge; saccus sub-triangular, approximately 1.5 times longer than broad, posterior edge weakly sinusoid, medial part with strongly sclerotised ridge extending from posterior edge to apex, lateral sclerites slightly shorter than maximum width of saccus; phallus with inflated coecum, about twice width and one-third length of distal part, strongly sclerotised spine of about ¾ length of coecum emerging from distodorsal part, directed towards apex of phallus in moderate curvation, distal part of phallus slightly curved, apex rounded.

Female genitalia. Unknown.

##### Diagnosis.

*Megacraspedusheckfordi* sp. n. is characterised by its plain light greyish brown forewings without markings. It can be separated from other species without markings on the forewings by its cream-white head. The male genitalia are unmistakable within *Megacraspedus* and can be easily identified by the characteristic large spine of the phallus.

##### Molecular data.

BIN BOLD:ACA9758 (n = 4). The intraspecific divergence of the barcode region is low with mean 0.5% and maximum divergence of 0.8%. The distance to the nearest neighbour *M.tenuiuncus* sp. n. is 7.8% (p-dist).

##### Distribution.

Spain (prov. Cantabria).

##### Biology.

Host plant and early stages are unknown. The adults have been collected in the middle of July at altitudes from 1230 to 1870 m.

##### Etymology.

The species name (a noun in the genitive case) is dedicated to Robert (Bob) Heckford, U.K., who collected the first specimen and provided his valuable material available to our study.

#### Megacraspedus tenuiuncus sp. n.

Taxon classificationAnimaliaLepidopteraGelechiidae

http://zoobank.org/538B86BC-5840-477D-976C-2F03267781F8

##### Examined material.

**Holotype** ♂, “Spain [prov.] Lerida Roni near Sort 7.vii.1993, 1000 m P. Skou” “GU 15/1402 ♂ P. Huemer” (ZMUC). **Paratypes.** France. 1 ♂, Dep. Pyrénées-Orientales, Les Queres, Angoustrine, 1410 m, 19.v.2004, leg. T. Varenne, genitalia slide 17979 Nel; 3 ♂, same data, but Les Fontêtes, 1500 m, 13.vii.2005, genitalia slide 19284 Nel (all RCTV). Spain. 1 ♂, same data as holotype, but 8.vii.1993, leg. P. Skou (ZMUC); 2 ♂, prov. Barcelona, Castellfollit de Riubregós, Bosc de Roters, 560 m, 14.vi.2012, leg. F. Vallhonrat & C. Coll, genitalia slide 2152 Requena and 26231 Nel (RCCR, RCJN); 1 ♂, prov. Barcelona, Castellfollit de Riubregós, Torrent del Magrà, 716 m, 15.vi.2012, leg. F. Vallhonrat & C. Coll (RCCR); 1 ♂, prov. Girona, La Molina, 1800 m, 23–26.vi.2004, leg. A. Blumberg, genitalia slide 5343 Karsholt (RCWS).

##### Description.

Adult. Male (Figure 58). Wingspan 13 mm. Segment 2 of labial palpus with scale brush about same length as segment 3, brown on outer surface, white mottled with brown on inner surface, white on upper surface; segment 3 rather short, white. Antennal scape without pecten; flagellum black. Head cream-white; thorax and tegula light brown, the latter with whitish tip. Forewing light greyish; costa white from base; fold and veins in apical part of wing whitish; an indistinct black dot in fold and a black dot at end of cell; termen with some blackish scales; fringes grey. Hindwing grey with concolourus fringes.

Female. Unknown.

Variation. The examined specimens show only slight variation.

Male genitalia (Figure 194). Uncus extraordinarily long and slender, about five times longer than broad, basally weakly widened, apex slighty pointed; gnathos hook bulky, with longitudinal grooves, straight, slightly longer and broader than uncus, apically strongly sclerotised, curved tip; anterior margin of tegumen with deep, U-shaped emargination, teguminal wall with two longitudinal ridges anteriorly, converging mediad; suboval pedunculi with small supplementing hump; valva straight, stout, extending to about middle of uncus, distorted apical part rounded; saccular area densely covered with setae, distally with short digitate sacculus; posterior margin of vinculum with deep U-shaped medial emargination, broadly rounded lateral humps, vincular sclerites elongated, posteriomedial edge strongly sclerotised; saccus sub-triangular, apically abruptly tapered, short, ratio maximum width to length approximately 1.1, posterior margin with pointed mediolateral projections, separated by moderately deep V-shaped incision, medial part with strongly sclerotised longitudinal ridge extending to anterior part of saccus, with weakly forked anterior end, lateral sclerites about three-quarters length of maximum width of saccus; phallus with weakly inflated coecum, about twice width of digitate distal four-fifths, distomedial part curved, with small spines ventrally, ductus ejucalatorius with contorted linear interior sclerotisation.

Female genitalia. Unknown.

##### Diagnosis.

*Megacraspedustenuiuncus* sp. n. is characterised by its black antennae, and by its light greyish forewings with two black dots. It is similar to *M.lanceolellus* (Figs 7–16), but that species has ringed antennae, and less distinct black dots on the forewing. The male genitalia of *M.tenuiuncus* sp. n. are unmistakable and in particular the extremely long and slender uncus is unique in *Megacraspedus*. It furthermore differs from most other species of the *M.pusillus* species group by the presence of a short sacculus.

##### Molecular data.

BIN BOLD:ADF1915 (n = 1). The distance to the nearest neighbour *M.heckfordi* sp. n. is 7.7% (p-dist).

##### Distribution.

France (Pyrénées), Northern Spain.

##### Biology.

Host plant and early stages are unknown. The adults have been collected from the middle of June to early July at altitudes from 560 m to 1800 m.

##### Etymology.

The species name is a compound word derived from the Latin words *tenuis* (meaning small) and *uncus*, referring to the slender uncus. The name is a noun in apposition.

### *Megacraspeduslativalvellus* species group

The *M.lativalvellus* species group includes one species: *M.lativalvellus*.

External morphology. See species description.

Genitalia morphology. Male genitalia. See species description.

Diagnosticremarks. The *M.lativalvellus* species group is defined by structures of the male genitalia, particularly the long gnathos hook, submedially bent at right angles, the short and smoothly sclerotised saccus without ridge, and the sclerotisation of the phallus with postmedial thorn are characteristic.

The systematic position of the *M.lativalvellus* species group is uncertain and tentative due to the preparation-related lack of a ductus ejaculatorius, and in the lack of females and supportive molecular data.

#### Megacraspedus lativalvellus

Taxon classificationAnimaliaLepidopteraGelechiidae

Amsel, 1954

Megacraspedus lativalvellus Amsel, 1954: 54, fig. 4.

##### Examined material.

**Holotype** ♂, “MALTA SALINA 16.III.52. Coll. Delucca” “Type” “Gen. prep. Nr. 5092 ♂ O. Karsholt” (RCCDL). **Paratype.** 1 ♂, same data as holotype, but 31.iii.1952, genitalia slide 1850 Amsel (SMNK). **Non-type material.** Spain. 1 ♂, prov. Cadiz, 10 km NW Tarifa, Punta Paloma, 30.iii.-4.iv.1994, leg. H. van der Wolf, genitalia slide 5347 Karsholt (RCHW).

##### Redescription.

Adult. Male (Figure 59). Wingspan 14 mm. Segment 2 of labial palpus with scale brush about same length as segment 3, brown on outer and inner surface, white on lower and upper surface; segment 3 cream-white mottled with some brown. Antennal scape with pecten of 1–2 hairs, flagellum finely ciliate, brownish, indistinctly ringed lighter. Head, thorax and tegula cream coloured, mottled with light brown. Forewing light yellowish brown mottled with some greyish, especially in costal third and towards apex; veins and costa white; fringes grey. Hindwing grey with light grey fringes.

Female. Unknown.

Variation. Unknown.

Male genitalia (Figure 195). Uncus approximately 1.5 times longer than broad, with parallel lateral margins, apex shallow sinusoid; gnathos hook evenly slender, more than twice length of uncus, bent at right angles at about 2/5; tegumen with broad and shallow U-shaped anterior margin, anteromedially small additional emargination; pedunculi small, sub-triangular, posteriorly divided by sclerotised ridge; valva extending to middle of uncus, basally weakly inflated, digitate distal part weakly tapered to slightly pointed apex; sacculus absent; posterior margin of vinculum with distinct lateral hump, vincular sclerite broadly sub-triangular, without strongly sclerotised posterior margin; saccus sub-triangular, with pointed apex, short, ratio maximum width to length approximately 1.4, posterior margin with weakly sinusoid mediolateral humps, medial part smooth, without sclerotised ridge, lateral sclerites slightly shorter than maximum width of saccus; phallus with strongly inflated coecum, about three times wider than distal part, oblong sclerotisation in distal part with a short spine.

Female genitalia. Unknown.

##### Diagnosis.

*Megacraspeduslativalvellus* is similar overall to *M.dolosellus* and related species. It can be separated from these by the longer segment 3 of the labial palpus, and by having a pecten of one or a few hairs on the antennal scape. The male genitalia are easily recognized by the characteristic gnathos hook, only shared by a few other species such as *M.leuca* (Figure 237) from which they differ in several characters. Furthermore, the short and broad, smoothly sclerotised saccus, and the spine of the phallus are characteristic of *M.lativalvellus*.

##### Molecular data.

Not available, no suitable specimen was available for barcoding.

##### Distribution.

Malta and South Spain.

##### Biology.

Host plant and early stages are unknown. The few examined specimens were collected in March to early April at unreported altitudes.

##### Remarks.

*Megacraspeduslativalvellus* was described from three males collected in Malta in March 1952 by C. De Lucca (Amsel, 1954). The holotype and one paratype were kept in the collection of Carmello De Lucca. After his death in 1971 the collection stayed with the family and was not curated, with the result that it suffered much from attack by pest insects (P Sammut in litt.). With the help from Paul Sammut we were able to borrow these two type specimens, but only the pins and labels were left – apart from the tip of the abdomen of the holotype, from which an almost complete genitalia slide could be prepared, however, leaving the apex of the phallus broken. The second paratype in SMNK is in relatively good condition.

The type locality, Salina in northern Malter is now a nature reserve, and there is thus a possibility that *M.lativalvellus* is still present there.

### *Megacraspedusdejectella* species group

The *M.dejectella* species group includes one species: *M.dejectella*.

External morphology. See species description.

Genitalia morphology. Male genitalia. See species description.

Diagnosticremarks. The *M.dejectella* species group is defined by structures of the male genitalia, particularly the small uncus, and the stout and apically curved gnathos hook, the distinct longitudinal ridge of the entire saccular area, and the posteriorly arched and smoothly sclerotised saccus are characteristic.

The systematic position of the *M.dejectella* species group is uncertain and tentative in the absence of females and supportive molecular data.

#### Megacraspedus dejectella

Taxon classificationAnimaliaLepidopteraGelechiidae

(Staudinger, 1859)

Gelechia dejectella Staudinger, 1859: 242.

##### Examined material.

**Lectotype** ♂, designated by Huemer and Tokár (2000: 2), “LECTO-TYPE” ”27/4” “Granada m. “Origin.” “Lectotype ♂ *Gelechia dejectella* Stgr teste. K. Sattler, 1986” “GU 01/968 ♂ P. Huemer” “LECTOTYPE ♂ *Gelechia dejectella* Staudinger des. Huemer, 2002” (ZMHU). **Paralectotype** ♂, “PARA-LECTO-TYPE” “Origin.” “Paralectotype ♂ *Gelechia dejectella* Stgr teste. K. Sattler, 1986” “Ex coll. 2/2 Staudinger” “*dejectella* Stdgr.” “Gen. pr. Z. Tokár ♂ No 4456 [in tube]” “*Megacraspedus dejectellus* Z. Tokár, 1999” (ZMHU). **Non-type material.** No collecting data. 1 ♂, coll. Staudinger (ZMHU).

##### Redescription.

Adult. *Male* (Figure 60). Wingspan 11 mm. Segment 2 of same length as segment 3 [labial palpi of all three type spcimens completely denuded]. Antennal scape without pecten; flagellum blackish brown, indistinctly lighter ringed. Head whitish; thorax and tegula concolorous with forewing. Forewing light grey-brown mottled with whitish scales, apex darker grey brown; costa and fold white; an indistinct black streak in termen; fringes whitish grey. Hindwing light grey with whitish grey fringes.

*Female*. Unknown.

*Variation*. The above description is based on one rather worn specimen, having the labial palps totally denuded.

*Male genitalia* (Figure 196). Uncus small, maximum width about one-third of posterior teguminal edge, about two times longer than broad, with parallel lateral margins, apex evenly rounded; gnathos hook stout, slightly longer than uncus, distal half strongly curved with pointed apex; tegumen with broad and moderately shallow emargination anteriorly; pedunculi small, divided by sclerotised ridge; valva extending beyond middle of uncus, broadly digitate, distal part weakly contorted with rounded apex; saccular area with longitudinal ridge, covered with setae, without separated sacculus; posterior margin of vinculum with shallow emargination, distinct lateral hump, vincular sclerite broadly semi-oval, without strongly sclerotised posterior margin; saccus sub-triangular, with weakly concave outer edge, distal part more abruptly tapered to pointed apex, ratio maximum width to length approximately 0.7, posterior margin evenly arched, medially flattened, without incision, medial part smooth, without sclerotised ridge, lateral sclerites about two-thirds maximum width of saccus; phallus with bulbous coecum, distal three-fifths straight, evenly tapered to nearly pointed apex, with sclerotised longitudinal ridge medially.

*Female genitalia*. Undescribed.

##### Diagnosis.

*Megacraspedusdejectella* is characterised by its relatively small size, and by the light grey-brown forewings with a white costa and fold and being without black spots. It is similar to *M.sumpichi* sp. n. (Figs 70–71), but that species has scattered black scales on the forewing, and its fold is not white. It also resembles *M.bengtssoni* sp. n. (Figs 17–18), whose fold is also not white, and which has a black spot at the end of the forewing cell. The male genitalia are somewhat similar to species of the *M.bilineatella* species group but differ in several characters such as the shape of the uncus and gnathos hook, the broader valva with a distinct ridge and the phallus.

##### Molecular data.

Not available, no suitable specimen was available for barcoding.

##### Distribution.

Southern Spain (prov. Granada).

##### Biology.

Host plant and early stages are unknown. The type series was collected at the end of April at unreported altitudes.

##### Remarks.

*Megacraspedusdejectella* was described from three specimens of both sexes collected in the surroundings of Granada, southern Spain (Staudinger 1859). We examined two males from the type-series, but were unable to trace the female. Staudinger did not state the female to be brachypterous, and one may wonder if he had a female – or if the unlabelled male in ZMHU represents the third type specimen.

### *Megacraspedusbinotella* species group

The *M.binotella* species group includes four species: *M.devorator* sp. n., *M.binotella*, *M.brachypteris* sp. n., and *M.barcodiellus* sp. n.

External morphology. Segment 2 of labial palpus with scale brush as long as or longer than segment 3; segment 3 as long as segment 2. Antennal scape without pecten. Wingspan (males) 13–19 mm. Forewing with two black dots, but without white costa and white veins. Known females brachypterous.

Genitalia morphology. Male genitalia. Uncus large, suboval to mitre-shaped; gnathos hook moderately slender, weakly curved; valva stout, digitate, basally with small hump, without separate sacculus; saccus moderately small, broadly V-shaped, with usually well developed forked sclerotised ridge from posterior margin; phallus with globular coecum, stout, distal part weakly to distinctly S-curved, without dentation.

Female genitalia. Papilla analis small; apophysis posterior very long; segment VIII moderately long and slender, membranous; subgenital plate with sub-triangular subostial sclerotisation, posteriorly with pointed sclerites of variable length, anterior edge medially with distinct sub-triangular projection; apophysis anterior rod-like, from posterior margin of segment VIII, posteriorly with sclerotised widening; colliculum short; signum small, rounded spiny plate.

Diagnosticremarks. The *M.binotella* species group is defined by the combined structures of the male genitalia, particularly the nearly mitre-shaped uncus, the slender gnathos hook, the short and comparatively small saccus with long lateral sclerites, and particularly the S-curved phallus that is only shared wirth few other species of the *M.dolosellus* species group. The female genitalia largely agree overall with several taxa in other species groups.

#### Megacraspedus devorator sp. n.

Taxon classificationAnimaliaLepidopteraGelechiidae

http://zoobank.org/40A57FA9-21B5-47EB-A5EF-D60AFEA54752

##### Examined material.

**Holotype** ♂, “BULGARIA [Dobrich region] Nos Sabla [Shabla] 27.5.2002 J. Junnilainen leg.” “DNA Barcode TLMF Lep 19954” genitalia prep. (in glycerin) (RCJJ). **Paratypes.** Bulgaria. 2 ♂, Tuzlata, 26.v.2006, leg. J. Junnilainen (RCJJ); 1 ♂, East Rhodopes mts, near Madzharovo, Nature Conservation Center “E Rhodopes”, 29.iv.2006, leg. B. Zlatkov & S. Beshkov (BFUS). Romania. 1 ♂, N Dobrogea, 5 km NE Ciucurova, 23.v.2009, leg. J. Junnilainen, genitalia slide GU 16/1461 Huemer (RCJJ).

##### Description.

**Figures 61–66.**
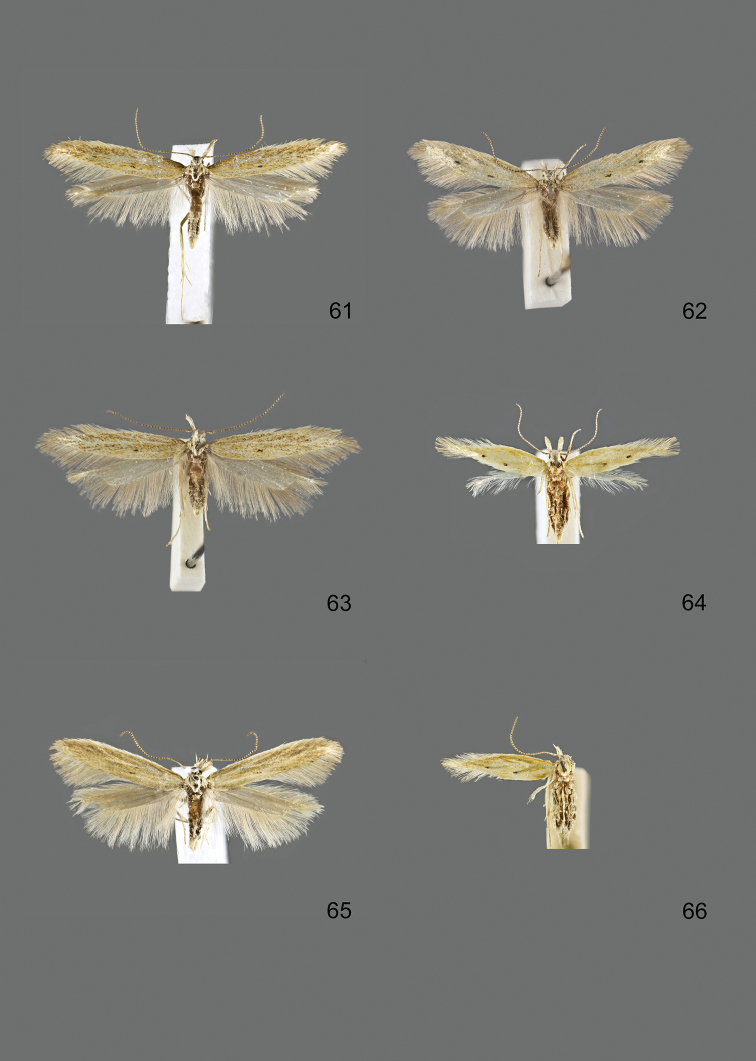
*Megacraspedus* adults in dorsal view. **61***M.devorator* sp. n. – Paratype, male, Romania (RCJJ) **62***M.binotella* (Duponchel, 1843) – male, Austria (RCEA) **63***M.binotella* (Duponchel, 1843) – male, Austria (RCEA) **64***M.binotella* (Duponchel, 1843) – female, Italy (TLMF) **65***M.brachypteris* sp. n. – Paratype, male, Albania (NMPC) **66***M.brachypteris* sp. n. – Paratype, female, Macedonia (NHMW).

Adult. *Male* (Figure 61). Wingspan 12–17 mm. Segment 2 of labial palpus with long scale brush, light brown on outer surface, white mottled with brown on inner surface, white on lower and upper surface; segment 3 white. Antennal scape without pecten; flagellum ringed blackish brown and light brown. Head white; thorax and tegula as forewing. Forewing light brown mottled with some black, especially towards apex; edge of costa white; an elongate black spot in fold and a shorter one at end of cell; veins in apical part indistinctly lighter; fringes light grey. Hindwing grey with light grey fringes.

*Female*. Unknown.

*Variation*. There is considerable variation in wingspan.

*Male genitalia* (Figure 197). Uncus large, approximately 1.8 times as long as maximum width, parallel outer margins, apically rounded; gnathos hook moderately slender, apically pointed, about length of uncus, weakly curved; anterior margin of tegumen with broad and shallow U-shaped emargination, teguminal wall with short longitudinal ridge anteriorly; pedunculi of moderate size, suboval, with small additional sclerite; valva stout, basally with small hump, sub-basally with large bulge, distal part slender digitate, about half width of uncus, extending slightly to about middle of uncus, apically constricted, weakly rounded; saccular area densely covered with setae, without separated sacculus; posterior margin of vinculum with shallow medial emargination, without distinct lateral humps, vincular sclerite elongated, suboval, with strongly sclerotised posterior edge; saccus moderately large, broadly V-shaped, short, ratio maximum width to length about 1, posterior margin with broadly rounded projections, separated by shallow incision, medial part with furcated sclerotised ridge from posterior margin to anterior third of saccus, lateral sclerites approximately 0.9 times length of maximum width of saccus; phallus with strongly inflated globular coecum, with transverse sclerotised band, approximately 2.5 times wider than distal part, distal part moderately stout, about 3 times length of coecum, straight, with dorsal and ventral sclerotised zones, apex weakly pointed, ductus ejaculatorius with slender interior sclerotisation.

*Female genitalia*. Unknown.

##### Diagnosis.

*Megacraspedusdevorator* sp. n. is characterised by its light brown forewings with a white costal edge and two elongate black spots. It is very similar to *M.binotella* (Figs 62–64), which differs by its lighter, cream-coloured ground colour of the forewings. The male genitalia differ from the similar *M.binotella* (Figure 198) particularly in the more slender and straight distal part of the phallus and the shape of the uncus with parallel outer margin, from other species of the *M.binotella* species group e.g., by the slender valva.

##### Molecular data.

BIN BOLD:ADB7270 (n = 2). Genetically variable species. The intraspecific divergence of the barcode region is considerable with 2.2%. The distance to the nearest neighbour *M.binotella* is 9% (p-dist).

##### Distribution.

Bulgaria, Romania.

##### Biology.

Host plant and early stages are unknown. The adults have been collected from late April to late May from sea level to unreported altitudes in Dobrogea mountains.

##### Etymology.

The species name refers to the Latin noun *devorator* (=food), and refers to eating (too) much after hunger during an expedition to Altai mts in search for *Megacraspedus* together with Christoph Wieser.

#### Megacraspedus binotella

Taxon classificationAnimaliaLepidopteraGelechiidae

(Duponchel, 1843)

Palpula binotella Duponchel, 1843: 256, pl. 72, fig. 7.
Ypsolophus binotellus Fischer von Röslerstamm, 1843: 300, 301, pl. 99, figs 2a, 2b; homonym and synonym of Palpulabinotella Duponchel, 1843.

##### Examined material.

**Syntype** ♂, “*binotella* FR” “TYPE” “Duponchel” “2169” [without abdomen] (MNHN) [photographs examined]. **Non-type material.** Austria. 1 ♂, without locality, leg. Mann (NHMW); 1 ♂, 1 ♀, Niederösterreich, südl. Wienerwald, Sooss, 28.iv.1968, leg. Arenberger (RCEA); 2 ♂, Niederösterreich, Gramatneusiedl, Fürbachwiesen, 22.v.1964, leg. E. Arenberger; 2 ♂, same data, but 17.v.1964, leg. E. Arenberger; 1 ♂, same data, but 21.v.1968, leg. F. Kasy (NHMW, RCEA, ZMUC); 1 ♂, Niederösterreich, Wolkersdorf, Hochleiten, 14.v.1925; 1 ♂, Niederösterreich, Mödling, 18.vi.1916; 1 ♀, Wien, Bisamberg, 27.v.1902, leg. Preissecker; 2 ♀, Wien, Prater, 1857, genitalia slide GU 17/1481 Huemer (all NHMW); 1 ♂, Steiermark, U. Rein, Enzenbach-Horgar Pauli, 500 m, 16.v.1978, leg. K. Rath, genitalia slide GEL 187 Huemer (TLMF). Croatia. Platak, 1000 m, 1.vi.2008, leg. J. Junnilainen (RCJJ). Czech Republic. 1 ♂, Brvany, Pisecny, 300 m, 7.vi.2013, leg. J. Šumpich (NMPC). Germany. 2 ♂, Bayern, Eching, late v.1949, leg. H. Pfister (TLMF). Hungary. 1 ♂, Budaörs, 2.v.1953, leg. J. Szöcs (ZMUC). Italy. 1 ♂, 1 ♀, Mte. Baldo, Costabella, 1800 m, late vi.1965, leg. K. Burmann (TLMF); 1 ♂, Verona, Monte, 420 m, 11.v.2012, leg. J. Skyva (NMPC). Poland. 1 ♂, prov. Lublin, Dobużek, 30.v.1996, leg. T. Rynarzewski (RCTZ). Romania. 1 ♂, Carpatii orientali, Muntii Gurghiului, Brădeşti, 600 m, 14.v.2003, leg. S. & Z. Kovács; 1 ♂, Carpatii orientali, Muntii Perşani, Cheile Vărghişului, 700 m, 27.v.2009, leg. S. & Z. Kovács (all RCKO). Slovakia. 1 ♂, Hrabušice, v.1977, leg. J. Patočka; 1 ♂, NR Viniansky Hradný vrch, 8.v.1988, leg. Z. Tokár; 1 ♂, same data, but 14.v.1988 (ZMUC); 1 ♀, same data, but 19.v.2009 (RCTZ); 1 ♂, Staré, 8.v.1988, leg. Z. Tokár (ZMUC); 1 ♂, Zemplinske vrchy, 2.v.2003, leg. Z. Tokár; 1 ♂, 1 ♀, NR Viniansky, hradny vrch, 10.v.2009, leg. Z. Tokár; 1 ♀, Mikulásow, 30.iv.-1.v.2005, leg. Z. Tokár (all RCZT). Slovenia. 2 ♂, Nanos mts, Strmec, 700 m, 25.v.2001, leg. J. Liška; 1 ♂, Nanos mts, Hribac, 1000 m, 28.v.2000, leg. J. Liška (all NMPC); 1 ♂, Nova Gorica, Sabotin, 450 m, 3.vi.2008, leg. J. Skyva (NMPC). Without locality. 1 ♂ (ZMUC).

##### Redescription.

Adult. *Male* (Figs 62–63). Wingspan 13–17 mm. Segment 2 of labial palpus with long scale brush, light brown on outer surface, white mottled with brown on inner surface, white on lower and upper surface; segment 3 white. Antennal scape without pecten; flagellum ringed black and white. Head cream-white; thorax and tegula as forewing. Forewing cream coloured, more or less mottled with yellow and brown-tipped scales; basal part of costa dark; a black dot in fold at ½ and one at end of cell; fringes light grey. Hindwing light grey with concolorous fringes.

*Female* (Figure 64). Wingspan 12–14 mm. Forewing ellipsoidal with two distinct black spots and some black scales in apex. Hindwing about one-third as broad as in male, with lanceolate apex; whitish grey. Otherwise similar to male.

*Variation*. The forewing can be more or less mottled with brown-tipped scales, and rarely the veins are whitish. Some specimens have darker scales along the termen. Also the head can be light brown. The tip of segment 3 of the labial palps is sometimes black. *M.binotella* furthermore shows some variation in size (see also *M.brachypteris* sp.n.) with e.g., two specimens collected simultaneously ranging between 14 and 17 mm wingspan.

*Male genitalia* (Figure 198). Uncus large, mitre-shaped, apex strongly constricted; gnathos hook moderately slender, apically pointed, about length of uncus, weakly curved; anterior margin of tegumen with broad and shallow U-shaped emargination, teguminal wall with short longitudinal ridge anteriorly; pedunculi of moderate size, suboval; valva stout, basally with small hump, sub-basally with large bulge, distal part slender digitate, about half width of uncus, extending slightly beyond base of uncus, apex rounded; saccular area densely covered with setae, without separated sacculus; posterior margin of vinculum with shallow medial emargination, without distinct lateral humps, vincular sclerite elongated, suboval, with strongly sclerotised posterior edge; saccus moderately large, broadly V-shaped, short, ratio maximum width to length about 1, posterior margin with broadly rounded projections, separated by shallow incision, medial part with furcated sclerotised ridge from posterior margin anterior third of saccus, lateral sclerites approximately 0.8 times length of maximum width of saccus; phallus with strongly inflated globular coecum, with transverse sclerotised band, approximately 2.5 times wider than distal part, distal part moderately stout, approximately 2.5 times length of coecum, sclerotised dorsal ridge weakly S-curved, apex constricted, ductus ejaculatorius with slender interior sclerotisation.

*Female genitalia* (Figure 277). Papilla analis small, apically rounded; apophysis posterior slender rod-like, 2.2 mm long, with short, bifurcate posterior end and minute sclerotised zone; segment VIII 0.9 mm long, membranous; sub-genital plate with sub-triangular subostial sclerotisation, posteriorly with long, pointed sclerites, extended beyond middle of segment VIII, anteromedially delimiting sub-ovate ostium bursae, anterior margin with rod-like edge connected with apophysis anterior, medially with long and slender sub-triangular projection; apophysis anterior 1.3 mm, slender, rod-like, about length of segment VIII, posteriorly becoming rod-like venula of segment VIII with widened end; colliculum short, sclerotised; ductus bursae gradually widened into sub-ovate and weakly delimited corpus bursae, entire length of ductus and corpus bursae 1.8 mm; signum medium-sized, longitudinal, suboval spiny plate.

##### Diagnosis.

*Megacraspedusbinotella* is characterised by the creamy forewings with two distinct black dots. It resembles well-marked specimens of *M.brachypteris* sp. n., a species which usually differs in the whitish veins and the short-winged female. See also *M.devorator* sp. n. (p 86). The male genitalia are very similar to *M.barcodiellus* sp. n. (see also Figure 201) and *M.brachypteris* sp. n. (Figs 199–200) from which they differ in the more slender valva and the shape of the phallus which is less curved than in *M.brachypteris* sp. n. and not straight as in *M.barcodiellus* sp. n. The female genitalia are very similar to *M.brachypteris* sp. n. (Figure 278) and mainly differ in the distinctly longer pointed sclerites of the sub-genital plate.

##### Molecular data.

BIN BOLD:ACM09062 (n = 5), BIN BOLD:ACW5732 (n = 1), BOLD:ADI7972 (n = 1). Genetically variable species. The intraspecific divergence of the barcode region is large and reflected by 3 BINs with an average distance of 1% and a maximum divergence of 2% in BIN BOLD:ACM09062. The mean intraspecific divergence is 2.2%, with a maximum divergence of 3.8% indicating possible cryptic diversity. The minimum distance to the nearest neighbour *M.devorator* sp. n. is 9% (p-dist).

##### Distribution.

Austria, Czech Republic, Germany (northwards to Rheinland-Pfalz and Hessen) (Hausenblass 2006: 10), Hungary, northern Italy, south-eastern Poland, Romania, Slovakia, Slovenia. *Megacraspedusbinotella* is also listed from Portugal and Spain by Corley (2015: 86) and Vives (2014: 171), respectively. However, it seems very unlikely to us that the eastern European *M.binotella* occurs on the Iberian Peninsula. Material from Spain and Portugal is probably based on misidentifications and may refer to the externally similar *M.peslieri* sp. n. and/or other similar species.

##### Biology.

Early stages are unknown. Piskunov (1981: 989) gives *Poa* (Poaceae) as a host plant. The adults have been collected from late April to late June at altitudes up to 1800 m.

##### Remarks.

*Palpulabinotella* was described from an unstated number of specimens which Duponchel had received through the insect dealer Parreyss under the name “*binotella* Fischer de Röslerstamm” (Duponchel 1843). *Megacraspedusbinotella* was previously attributed to Fischer von Röslerstamm. However, Joannis (1915: 116) stated that the description by Duponchel was published in January 1843, and that of Fischer von Röslerstamm in March 1843. In the legends to the plate in the original description the combination was given as “*Lita binotella*”. The type locality of *Palpulabinotella* was not stated by Duponchel, but it may well have been Vienna, Austria, from where the type-series of *Ypsolophusbinotellus* Fischer von Röslerstamm originated.

#### Megacraspedus brachypteris sp. n.

Taxon classificationAnimaliaLepidopteraGelechiidae

http://zoobank.org/CC9E6A71-F4C4-492E-9712-AF4015BA9A1D

##### Examined material.

**Holotype** ♂, “Macedonia NP [National Park] Mavrovo Korab [mountain], eastern ridge ca. 2325–2400 m 20°34'46"E, 41°47'08"N 28.7.-1.8.2011 leg. Huemer & Tarmann” “BC TLMF Lep 05189” “P. Huemer GEL 1199 ♂” (TLMF). **Paratypes.** Albania. 2 ♂, Qafa e Theothores, 14.vii.2013, leg. I. Richter, genitalia slide GU 17/1482 Huemer (NMPC); 1 ♂, Pashtrik, 4–14.vii.1918; 2 ♂, same data, but 29.v–4.vi.1918; 3 ♂, Kula Ljums, 18–28.v.1918, genitalia slide Mus. Vind 16.660 (NHMW); Kula Ljums, 18–28.v.1918, leg. Alban. Exped.; 1 ♂, Pashtrik, 19.v.–4.vi.1918, leg. Alban. Exped., genitalia in vial (all NHMW). Bosnia and Herzegovina. 1 ♂, Trescavica, 16.vii.1903, leg. H. Rebel (NHMW). Greece. 1 ♂, Makedonia, 2.4 km SE Pisoderi, Vigla, 1550 m, 18.vi.2013, leg. P. Skou genitalia slide GU 16/1460 Huemer (ZMUC). Macedonia. 3 ♂, same data as holotype; 1 ♂, 1 ♀, Sar Planina, Kara Bunar, 2300 m, 31.vii.–1.viii.2014 (all TLMF); 2 ♂, Gornja Matka, 22–23.v.2013, leg. I. Richter (NMPC); 1 ♂, 1 ♀, Peristeri mts, Golemo ezero, 2200 m, 15–25.vii.1959, leg. F. Kasy (NHMW). Montenegro. 2 ♂, Kučka Krajina, Rikavačo jezero, 28.vi.2012 (NMCP).

##### Description.

Adult. *Male* (Figure 65). Wingspan 14–19 mm. Segment 2 of labial palpus with long scale brush, dark to light brown on outer and inner surface, white on lower and upper surface; segment 3 dirty white. Antennal scape without pecten; flagellum ringed blackish brown and white. Head white mottled with light brown; thorax and tegula as forewing. Forewing light brown from white brown-tipped scales; basal part of costa darker; a black dot in fold at ½ and one at end of cell well developed to reduced; fold yellow-brown; veins and medial edge of costa faintly whitish; fringes light grey. Hindwing light grey with concolorous fringes.

*Female* (Figure 66). Wingspan 11 mm. Labial palpus cream coloured. Forewing ellipsoidal, yellowish grey-brown, especially at base, along costa and around apex; two distinct black spots; veins indistinctly lighter; fringe light yellowish. Hindwing 2.5 times shorter than forewing, lanceolate, with lanceolate apex; whitish grey. Hindwing whitish grey, almost without fringes. Otherwise similar to male.

*Variation*. The variation in the forewing colour and labial palpi is probably due to faded compared with fresh specimens. Similarly the extent of the the two black dots of the forewing shows some variation, particularly in worn specimens they are largely reduced or denuded. Specimens from Albania (Pashtrik, Kula Ljums) have distinct black spots and are larger on average (17–19 mm) than those from other populations.

*Male genitalia* (Figs 199–200). Uncus large, sub-ovate, apex strongly constricted; gnathos hook moderately slender, apically pointed, about length of uncus, weakly curved; anterior margin of tegumen with broad and shallow U-shaped emargination, teguminal wall with short longitudinal ridge anteriorly; pedunculi of moderate size, suboval; valva stout, basally with small and digitate lateral projection, sub-basally with broad hump, distal part broadly digitate, about width of uncus, extending at most slightly beyond base of uncus, apex weakly contorted, rounded; saccular area densely covered with setae, without separated sacculus; posterior margin of vinculum with distinct medial emargination, weakly developed lateral humps, vincular sclerite elongated, suboval, with broadly sclerotised posterior edge; saccus moderately large, broadly V-shaped, short, ratio maximum width to length about 1, posterior margin with broadly rounded, shallow projections, weakly separated by small incision, medial part with shortly furcated sclerotised ridge, lateral sclerites approximately 0.9 times length of maximum width of saccus; phallus with strongly inflated globular coecum, about two times wider than distal part, with transverse sclerotised band, distal part moderately stout, about two times length of coecum, sclerotised dorsal and ventral ridge distinctly S-curved, apex pointed ventrad, ductus ejaculatorius with slender interior sclerotisation.

*Female genitalia* (Figure 278). Papilla analis small, apically rounded; apophysis posterior slender rod-like, 2.2 mm long, with short, bifurcate posterior end and minute sclerotised zone; segment VIII approximately 0.9 mm long, membranous; subgenital plate with broadly sub-triangular subostial sclerotisation, posteriorly with short, pointed sclerites, extending across first third of segment VIII, anteromedially delimiting sub-ovate ostium bursae, anterior margin with rod-like edge connected with apophysis anterior, medially with long and broad sub-triangular projection; apophysis anterior approximately 1.6 mm, slender, rod-like, about length of segment VIII, posteriorly becoming rod-like venula of segment VIII, extending to posterior margin of segment VIII, with widened posterior end; colliculum short, sclerotised; ductus bursae gradually widened into sub-ovate and weakly delimited corpus bursae, entire length of ductus and corpus bursae 1.8 mm; signum medium-sized, longitudinal, suboval spiny plate.

##### Diagnosis.

*Megacraspedusbrachypteris* sp. n. is characterised by its light brown forewings with faintly whitish veins and with two black spots present or reduced. It is sometimes hardly separable from certain forms of *M.binotella* (Figs 62–64). Females differ from the latter by the largely reduced hindwings. The male genitalia differ from other species of the *M.binotellus* species group by the broad valva and the strongly curved distal part of the phallus. These characters are only shared with *M.barcodiellus* sp. n. (Figure 201) but the sub-basally bulged valva and the scleritized ridge of the saccus are distinctive. The female genitalia are very similar to *M.binotella* (Figure 278) and differ in particular in the distinctly shorter, pointed sclerites of the subgenital plate.

##### Molecular data.

BIN BOLD:ABA3165 (n = 3), BIN BOLD:ACZ2904 (n = 1), BIN BOLD:ACZ3665 (n = 1), BIN BOLD:ADJ3544 (n = 2). Genetically variable species. The intraspecific divergence of the barcode region is large and reflected by 4 BINs with no divergence in BIN BOLD:ABA3165 but 0.9% variation in two specimens collected together and clustered in BIN BOLD:ADJ3544. The mean intraspecific divergence is 3.7%, with a maximum divergence of 6% indicating possible cryptic diversity. The minimum distance to the nearest neighbour *M.dolosellus* is 11.3% (p-dist).

##### Distribution.

Albania, Greece, Macedonia, Montenegro.

##### Biology.

Host plant and early stages are unknown. The adults have been collected from late May to early August at altitudes between 1550 and 2400 m.

##### Etymology.

The species name refers to the brachypterous female sex and is an adjective.

##### Remarks.

*Megacraspedusbrachypteris* sp. n. shows considerable phenotypic variation both individually and to some extent also geographically. However, the large intraspecific barcode divergence neither shows a clear geographic structure nor a concordant pattern to phenotypic appearance. Similarly the genitalia do not support cryptic diversity and therefore we accept only one species.

#### Megacraspedus barcodiellus sp. n.

Taxon classificationAnimaliaLepidopteraGelechiidae

http://zoobank.org/CA2E1223-08FD-4C1A-835D-63515ABB9720

##### Examined material.

**Holotype** ♂, “Macedonia NP [National Park] Mavrovo Korab [mountain], Korabska jezero, Kobilino pole, 2080–2180 m 20°34'55"E, 41°46'42"N 28.7.-1.8.2011 leg. Huemer & Tarmann” “BC TLMF Lep 05178” “P. Huemer GEL 1184 ♂” (TLMF). **Paratypes.** Macedonia. 7 ♂, NP Mavrovo Korab, eastern ridge, 2325–2400 m, 28.vii.–1.viii.2011, leg. P. Huemer & G. Tarmann; 1 ♂ Sar Planina, Titov Vrv SE, 2680–2748 m, 2.viii.2014, leg. G. Tarmann (TLMF, ZMUC).

##### Description.

**Figures 67–72.**
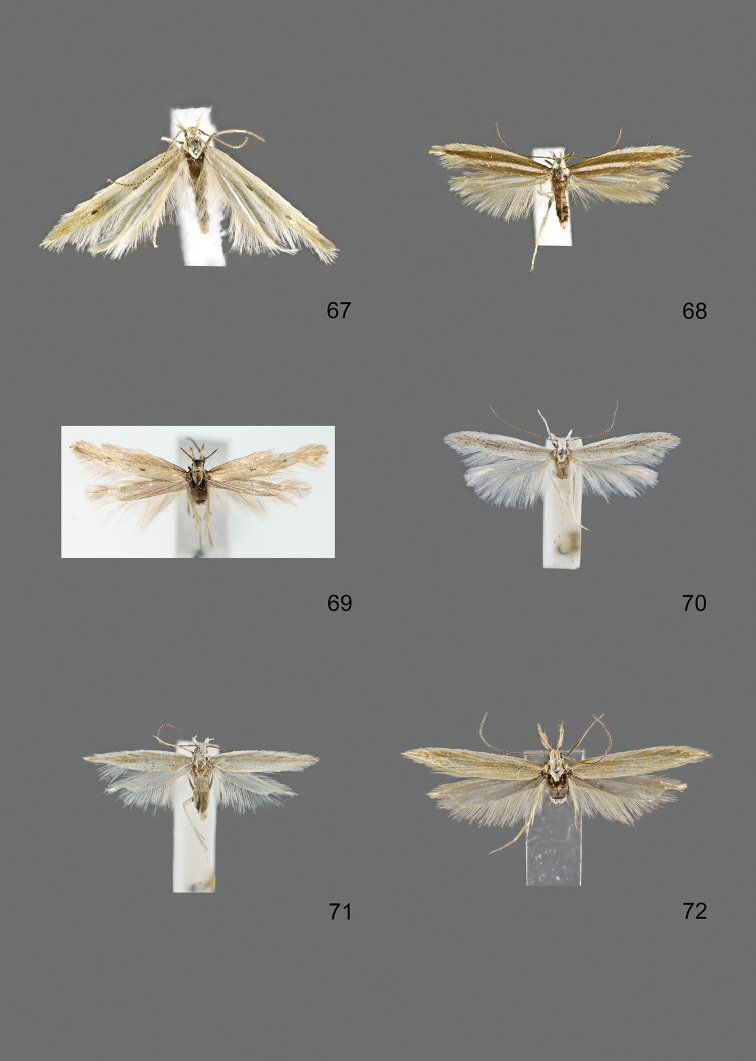
*Megacraspedus* adults in dorsal view. **67***M.barcodiellus* sp. n. – Paratype, male, Macedonia (TLMF) **68***M.bilineatella* Huemer & Karsholt, 1996 – Paratype, male, Italy (TLMF) **69***M.andreneli* Varenne & Nel, 2014 – Holotype, male, France (RCTV) **70***M.sumpichi* sp. n. – Holotype, male, Spain (TLMF) **71***M.sumpichi* sp. n. – Paratype, female, Spain (NMPC) **72***M.tabelli* sp. n. – Holotype, male, Morocco (ZMUC).

Adult. *Male* (Figure 67). Wingspan 14–15 mm. Segment 2 of labial palpus with long scale brush, white, slightly mottled with blackish brown, especially on outer surface; segment 3 white. Antennal scape without pecten; flagellum distinctly ringed black and white. Head white; thorax and tegula as forewing. Forewing cream-white, slightly darker towards apex; a distinct black spot in fold and one at end of cell; fringes whitish grey. Hindwing whitish grey with concolorous fringes.

*Female*. Unknown.

*Variation*. The spots of the forewing are sometimes reduced, but this may be due to worn specimens which furthermore become lighter and almost whitish.

*Male genitalia* (Figure 201). Uncus large, sub-ovate, apex strongly constricted; gnathos hook moderately slender, apically pointed, slightly longer than uncus, weakly curved; anterior margin of tegumen with broad and shallow U-shaped emargination, teguminal wall with short longitudinal ridge anteriorly; pedunculi broadly suboval; valva stout, basally with small lateral hump, distal part broadly digitate, slightly narrower than uncus, extending slightly beyond base of uncus, sub-apically a tiny tooth-like sclerite, apex weakly contorted, rounded; saccular area densely covered with setae, without separated sacculus; posterior margin of vinculum with distinct medial emargination, weakly developed lateral humps, vincular sclerite elongated, suboval, with sclerotised posterior edge; saccus moderately large, broadly V-shaped, short, ratio maximum width to length about 1, posterior margin with broadly rounded, shallow projections, weakly separated by small incision, medial part smooth, lateral sclerites approximately 0.8 times length of maximum width of saccus; phallus with strongly inflated globular coecum, approximately 2.5 times wider than distal part, with transverse sclerotised band, distal part moderately slender, nearly 3 times length of coecum, sclerotised dorsal and ventral ridge distinctly S-curved, apex pointed ventrad, ductus ejaculatorius with slender interior sclerotisation.

*Female genitalia*. Unknown.

##### Diagnosis.

*Megacraspedusbarcodiellus* sp. n. is characterised by its plain cream-white forewings with two distinct black spots. It is very similar to *M.andreneli* (p 93). The male genitalia are similar to those of other species of the *M.binotella* species group but differ in particular in the strongly curved distal part of the phallus and the broad valva. These characters are similar to *M.brachypteris* sp. n. (Figs 199–200) but the sub-basal part of the valva is not bulging and the saccus wall is smooth.

##### Molecular data

. BIN BOLD:ABA2916 (n = 3). The intraspecific divergence of the barcode region is 0%. The distance to the nearest neighbour *M.binotella* is 10.7% (p-dist).

##### Distribution.

Macedonia (Korab, Sar Planina).

##### Biology.

Host plant and early stages are unknown. The few adults known to date have been collected in late July and early August at altitudes between 2400 and ca. 2700 m.

##### Etymology.

The name refers to the valuable method of DNA barcoding in identification of this and several other species of *Megacraspedus*. It is derived from the latinized vernacular barcode, and the diminutive suffix -*ellus*. The name is a compound noun in apposition.

### *Megacraspedusbilineatella* species group

The *M.bilineatella* species group includes three species: *M.bilineatella, M.andreneli*, and *M.sumpichi* sp. n.

External morphology. Segment 2 of labial palpus with scale brush shorter to longer than segment 3; segment 3 shorter than segment 2. Antennal scape with a single pecten. Wingspan (males) 12–13 mm. Forewing light with two black dots or brown with three white streaks. Females unknown.

Genitalia morphology. Male genitalia. Uncus moderately small, gradually tapered with rounded apical edge; gnathos hook massive, strongly sclerotised, weakly curved at apex; tegumen with short and slightly converging sclerotised ridges in anteriomedial third; valva moderately long and slender, straight, digitate, apex rounded; saccular area moderately setose, without separated sacculus; saccus elongated sub-triangular, posterior margin shallowly arched, with weak medial emargination, medial part smooth, without sclerotised ridge, lateral sclerites short; phallus straight, with bulbous coecum, distal two-thirds slender, rod-like, with sclerotised longitudinal ridge.

Diagnosticremarks. The *M.bilineatella* species group is defined by several structures of the male genitalia, particularly the small and distally narrowing uncus, the comparatively massive gnathos hook, the slender and digitate valva without sacculus, the smoothly sclerotised saccus, and the phallus without distinct sclerotisations are characteristic in this combination.

The three species in this species group are overall similar in the male genitalia. However, they strongly differ in phenotypic appearance and in DNA barcode divergence and thus the assignment to one species group is tentative. Studies of the female as well as molecular analysis of additional markers will be necessary to resolve this problem satisfactorily.

#### Megacraspedus bilineatella

Taxon classificationAnimaliaLepidopteraGelechiidae

Huemer & Karsholt, 1996

Megacraspedus bilineatella Huemer & Karsholt, 1996: 251, figs 1–10.

##### Examined material.

**Holotype** ♂, “ITALIA sept. Prov. Bergamo Alpi Orobie W Ca. San Marco, 1800–1900 m, 22.7.1992 leg. Huemer & Tarmann” “Holotype *Megacraspedus bilineatella* Huemer & Karsholt, 1995” (TLMF). **Paratypes.** Italy. 7 ♂, same data as holotype, but genitalia slides GEL 355 Huemer, GEL 561 Huemer, GEL 562 Huemer (TLMF, ZMUC).

##### Redescription.

Adult. *Male* (Figure 68). Wingspan 12–13 mm. Segment 2 of labial palpus with comparatively short scale brush, brown on outer and inner surface, white on upper and lower surface; segment 3 shorter than segment 2, white with some black towards tip. Antennal scape with a single white pecten, flagellum blackish brown. Head whitish brown; thorax and tegula as forewing. Forewing brown; a broad white streak from base through fold to termen; costa white almost from base to apex; an indistinct white line along dorsum; fringes whitish grey. Hindwing light grey with concolorous fringes.

*Female*. Unknown.

*Variation*. The few examined specimens show only slight variation.

*Male genitalia* (Figure 202). Uncus moderately small, approximately 1.25 times length of maximum basal width, sub-ovate, gradually tapered from base to rounded apical edge; gnathos hook massive, slightly longer than uncus, strongly sclerotised with weakly curved apex; anterior margin of tegumen with shallow excavation, medially with additional weak emargination, with short and slightly converging sclerotised ridges in anteriomedial third; pedunculi small with peg-like sclerotisation; valva moderately long and slender, straight, digitate, apex rounded, extending to about middle of uncus, almost entire length of valva weakly lobed ventrally; saccular area covered with some microtrichia, without separated sacculus; posterior margin of vinculum with weak medial emargination, indistinct lateral humps, vincular sclerites broadly sub-ovate, posterior edge strongly sclerotised; saccus elongated sub-triangular, apically pointed, ratio maximum width to length approximately 0.75, posterior margin shallowly arched, with indistinct medial emargination, medial part smooth, without sclerotised ridge, lateral sclerites short, approximately 0.7 times length of maximum width of saccus; phallus about two-thirds length of genitalia capsule, straight, with bulbous coecum, distal two-thirds slender, rod-like, with sclerotised longitudinal ridge.

*Female genitalia*. Unknown.

##### Diagnosis.

*Megacraspedusbilineatella* is unique within the genus in having the forewings brown with two distinct, longitudinal white streaks along costa and through the middle of the wing and no other markings. The male genitalia are somewhat similar to several other congeners and differ from the closest, *M.andreneli* (Figure 203), in the massive and strongly sclerotised gnathos hook without subapical widening, the small pedunculi and the elongated sub-triangular saccus. From the nearest neighbour in DNA barcode *M.albovenata* (Figure 213), it differs furthermore in the shape of the uncus.

##### Molecular data.

BIN BOLD:ABU7227 (n = 1). The distance to the nearest congeneric neighbour *M.fallax* is extraordinarily large with 14.9% (p-dist), whereas several other Gelechiidae in BOLD have smaller distances.

##### Distribution.

Northern Italy (Prov. Bergamo).

##### Biology.

Host plant and early stages are unknown. The adults have been collected in the last third of July, flying freely around low vegetation in the early morning, just after sunrise. The habitat includes mainly grazed, south-exposed slopes with rich vegetation on siliceous soil at altitudes between about 1800 to 1900 m (Huemer and Karsholt 1996: 252).

##### Remarks.

*Megacraspedusbilineatella* was described from nine males collected in the Orobian Alps in northern Italy (Huemer and Karsholt 1996: 251).

#### Megacraspedus andreneli

Taxon classificationAnimaliaLepidopteraGelechiidae

Varenne & Nel, 2014

Megacraspedus andreneli Varenne & Nel, 2014: 61, figs 7–9.

##### Examined material.

**Holotype** ♂, France, Alpes-Maritimes, Saint-Sauveur-sur-Tinée, Lac Nègre, Parc national du Mercantour, 10 août 2013, 2450 m Th. Varenne leg., genitalia slide JN n°27182 (RCTV) [photographs examined]. **Paratype.** France. 1 ♂, Alpes-Maritimes, Valdeblore, Col de Veillos, 2250 m, 20.vii.2010, leg. A. Nel, genitalia slide 24592 J. Nel (TLMF).

##### Redescription.

Adult. *Male* (Figure 69). Wingspan 12–12.5 mm. Segment 2 of labial palpus with long scale brush, brownish mottled with white on inner and upper surface; segment 3 shorter than segment 2, white with black tip. Antenna dark brown, indistinctly ringed lighter. Head cream-white; thorax and tegula as forewing. Forewing cream coloured, slightly mottled with yellow- or brown-tipped scales; basal part of costa blackish brown; a black dot in fold at ½ and one at end of cell; fringes light grey with some black-tipped scales. Hindwing grey with light grey fringes.

*Female*. Unknown.

*Variation*. The forewings of the paratype are darker than those of the holotype, probably because the latter is more worn.

*Male genitalia* (Figure 203). Uncus moderately small, 1.25 times length of maximum basal width, sub-ovate, gradually tapered from base to rounded apical edge; gnathos hook massive, stout, slightly longer than uncus, strongly sclerotised, subapically widened, with abruptly tapered and weakly curved apex; anterior margin of tegumen with moderately shallow excavation, with short and medially converged sclerotised ridges in anteriomedial third; pedunculi small, rounded; valva moderately long and slender, weakly curved, digitate, apex rounded, extending to about middle of uncus; saccular area covered with some microtrichia, without separated sacculus; posterior margin of vinculum emarginated, indistinct lateral humps, vincular sclerites sub-ovate; saccus sub-triangular, apically pointed, ratio maximum width to length approximately 0.85, posterior margin arched, with indistinct medial emargination, medial part smooth, without sclerotised ridge, lateral sclerites short, approximately 0.7 times length of maximum width of saccus; phallus with bulbous coecum, distal two-thirds about half width of coecum, weakly curved, digitate, apically rounded, with sclerotised longitudinal ridge.

*Female genitalia*. Unknown.

##### Diagnosis.

*Megacraspedusandreneli* is a rather uncharacteristic species. It can be confused with several other species having two black dots in the forewing, e.g., *M.binotella* and *M.barcodiellus* sp. n. (flagellum distinctly ringed black and white), and *M.lanceolellus* (costa white). The male genitalia are similar to those of *M.bilineatella* (Figure 202) but differ in the subapically widened gnathos hook, the more slender and curved valva, the shorter saccus and several subtle characters.

##### Molecular data.

BIN BOLD:ADG6163 (n = 1), BIN BOLD:ACS0692 (n = 1). Genetically variable species. The intraspecific divergence of the barcode region is large and reflected by 2 BINs with 4.3% divergence, based on a single specimen of each cluster. The minimum distance to the nearest congeneric neighbour *Megacraspedustokari* sp. n. is 11.2% (p-dist).

##### Distribution.

France (Dep. Alpes Maritimes).

##### Biology.

Host plant and early stages are unknown. The few type specimens were collected in July and August at altitudes between 2250 and 2450 m.

##### Remarks.

*Megacraspedusandreneli* was described from two males collected at two localities in the Alpes Maritimes (Varenne and Nel 2014). The observed intraspecific barcode divergence is not supported by obvious diagnostic characters in morphology from the original figures, except for the forewing colour of the holotype and paratype. We therefore accept for the time being that both specimens are conspecific. The description of male genitalia is based on a re-mounted slide. Some characters such as the posterior margin of the vinculum or the vincular sclerites were damaged before re-mounting and thus it was not possible to describe them accurately.

#### Megacraspedus sumpichi sp. n.

Taxon classificationAnimaliaLepidopteraGelechiidae

http://zoobank.org/01A74FDC-8957-4B8D-ADB0-4005F3BE6B2C

##### Examined material.

**Holotype** ♂, “HISP.Prov.d.Granada Sierra de Baza 1300 m 18.7.1987 leg.G. Baldizzone E.Traugott-Olsen” “P.Huemer GEL 1244 ♂” (TLMF). **Paratypes.** Spain. 2 ♂ prov. Alicante, 8 km N Albatera, 300 m, 28.iv.2008; 1 ♂, same data, but 5.v.2008; 5 ♂, 1 ♀, same data, but 4.viii.2010; 4 ♂, prov. Alicante, Santa Pola env., S of Balsares, salt marshes, 12.vi.2007, lg. J. Šumpich (all NMPC); 2 ♂, prov. Alicante, Rebate, 26.vi.1989, leg. B. Å Bengtsson, genitalia slide Bengtsson 3267 (RCBB, ZMUC); 1 ♂, prov. Alicante, 5 km NE El Campello, Buscot, 50 m, 2.v.1997, leg. P. Skou (ZMUC); 1 ♂, prov. Alicante, La Marina, 9–12.vi.2007, leg. A. Cox (ZMUC); 1 ♂, Almería, Capo de Gata, 23.vi.1989, leg. B. Å. Bengtsson (ZMUC); 1 ♂, prov. Almería, 10 km E Bedar, El Pinar, 325 m, 19–27.iv.2001, leg. P. Skou & B. Skule (ZMUC); 1 ♂, prov. Almería, El Pozo del Esparto, 10 m, 27.v.2006, leg. P. Skou (ZMUC); 1 ♂, prov. Almería, 1 km SW Tabernas, 370 m, 8.viii.2014, leg. J. Tabell (ZMUC); 1 ♂, prov. Almería, Sierra de Alhamilla, Huebro, 800–900 m, 29.iv.2008, leg. J. Sumpich; 2 ♂, prov. Almería, Sierra Cabrera, Mojácar env., El Agua del Medio, 50–150 m, 4.v.2008, leg. J. Sumpich; 1 ♂, prov. Almería, Tabernas env., Rambla de Tabernas 400 m, 3.viii.2010, leg. J. Šumpich (all NMPC); 1 ♂, prov. Almería, Sierra de Alhamilla, Nijar env., 560 m, 30.iv.2008, leg. Z. Tokár, genitalia slide GU 14/1382 Huemer (ZMUC); 16 ♂, 1 ♀, same data, but 13–14.vi.2007, leg. J. Šumpich (NMPC); 5 ♂, 3 ♀, prov. Almería, Sierra de Alhamilla, Turrillas env., route Colativi, 1000 m, 15–19.vi.2007, leg. J. Šumpich (NMPC);1 ♂, prov. Almería, Sierra de Alhamilla, 10 km N Nijar, 10.v.2014, leg. A. Stübner (ZSM); 3 ♂, 4 ♀, prov. Granada, Sierra del Chaparral, 900 m, 8–12.vii.2007, leg. G. Jeppesen, genitalia slides 6494 ♂, 6496 ♀ Hendriksen (TLMF, ZMUC); 2 ♂, prov. Granada, Moscaril, 500 m, 8–12.vii.2014, leg. G. Jeppesen, genitalia slide GU 15/1397 Huemer (TLMF, ZMUC); 3 ♂, prov. Granada, Benamaurel, 3–4.vi.2015, leg. H. Roweck & N. Savenkov; 1 ♂, prov. Alicante, Sierra de Crevillente, 400 m, 5.vi.2016, leg. H. Roweck & N. Savenkov (all ECKU); 1 ♂, 1 ♀, prov. Málaga, 3 km W Sedella, La Rahige, 650 m, 25.vii.2003, leg. P. Skou (ZMUC); 3 ♂, prov. Murcia, Sierra Espuña, 25.vi.1989, leg. B. Å. Bengtsson, genitalia slide Bengtsson 3269 (RCBB, ZMUC); 1 ♂, prov. Murcia, Mazzaron, Bolnuevo, 10 m, 25.v.1998, leg. P. Skou (ZMUC); 1 ♀, prov. Zaragoza, Mequinenza env., 80 m, 14.vii.2010, leg. Z. Tokár (RCZT).

##### Description.

Adult. *Male* (Figure 70). Wingspan 10–12 mm. Segment 2 of labial palpus with long scale brush, light brown on outer, lower and inner surface, white on upper surface; segment 3 white. Antennal scape without pecten; flagellum blackish brown, indistinctly ringed lighter. Head thorax and tegula whitish. Forewing light grey-brown with scattered black scales, especially along apical margins; costa white; fold and veins indistinctly white; a few black scales along termen; fringes grey. Hindwing light grey with whitish fringes.

*Female* (Figure 71). Similar to male.

*Variation*. There is some variation in the amount of grey-brown scales in the forewing. In specimens with many such scales the white veins look more distinct. Worn specimens become whitish.

*Male genitalia* (Figs 204–205). Uncus moderately slender, 1.5 times as long as maximum basal width, sub-ovate, gradually tapered to evenly rounded apex; gnathos hook slender, approximately 1.3 times length of uncus, weakly curved, with pointed apex; anterior margin of tegumen with deep and broadly rounded emargination, short ridges from anterior edge converging towards middle; pedunculi small, with additional sclerite; valva, straight, stout, extending beyond middle of uncus, apex rounded, saccular area densely covered with setae, without separated sacculus; posterior margin of vinculum with shallow emargination, indistinct lateral humps, vincular sclerites broadly sub-ovate, with weakly sclerotised posterior edge; saccus moderately short and broad, V-shaped, ratio maximum width to length nearly 1, posterior margin weakly bulged, with straight mediolateral projections, separated by shallow incision, medial part smooth, without sclerotised ridge, lateral sclerites approximately 0.7 times length of maximum width of saccus; phallus slightly shorter than tegumen, weakly curved medially, with bulbous coecum, distal three-fifths slender, with weakly rod-like sclerotisation ventrally, subapical area slightly contorted, ductus ejaculatorius with interior sclerotisation.

*Female genitalia* (Figure 279). Papilla analis small, apically rounded; apophysis posterior slender rod-like, approximately 2.1 mm long, with short, bifurcate posterior end; segment VIII approximately 0.7 mm long, membranous; subgenital plate with sub-triangular subostial sclerotisation, posteriorly extended into pointed sclerites delimiting sub-ovate ostium bursae, anterior margin with rod-like edge connected with apophysis anterior, medially with broadly sinusoid projection; apophysis anterior slender, rod-like, slightly shorter than segment VIII, posteriorly becoming rod-like venula of segment VIII, extending to posterior margin; colliculum short, sclerotised; ductus bursae very short, gradually widened into oblong and weakly delimited corpus bursae, entire length of ductus and corpus bursae approximately 1.5 mm; signum small, spiny, transverse plate with strong lateral thorns.

##### Diagnosis.

*Megacraspedussumpichi* sp. n. is characterised by its relatively small size, by the white head and the light grey-brown forewings without black dots and with white costa. It is very similar to *M.dejectella* (p 85). The male genitalia differ from related species particularly in the V-shaped and smooth saccus. The female genitalia are similar to those of *M.lanceolellus* (Figs 267–268) but differ in the much shorter apophysis posterior and the shorter sinusoid anteriomedial projection of segment VIII. From the more closely related *M.dolosellus* (Figs 271–274) they differ in the shorter apophysis anterior and posterior and the absence of large medial flaps, delimiting the ostium bursae.

##### Molecular data.

BIN BOLD:ACM1096 (n = 5). The intraspecific divergence of the barcode region is considerable with mean 1.7% and maximum divergence of 2.5%. The distance to the nearest neighbour *M.cerussatellus* is 11.4% (p-dist).

##### Distribution.

Southern Spain.

##### Biology.

Host plant and early stages are unknown. The adults have been collected from late April to early August at altitudes from sea level to 1300 m, indicating possible bivoltinism. Some specimens were collected in salt marshes.

##### Etymology.

The species name (a noun in the genitive case) is dedicated to Jan Šumpich, Czech Republic, who collected part of the type series and numerous other *Megacraspedus* specimens used for our study.

### *Megacraspedusfallax* species group

The *M.fallax* species group includes 14 species: *M.tabelli* sp. n., *M.gallicus* sp. n., *M.ribbeella*, *M.libycus* sp. n., *M.numidellus*, *M.albovenata*, *M.longipalpella*, *M.niphorrhoa*, *M.albella*, *M.fallax*, *M.balneariellus*, *M.podolicus*, *M.kazakhstanicus* sp. n., and *M.knudlarseni* sp. n.

External morphology. Segment 2 of labial palpus long to very long, porrect; segment 3 reduced. Antennal scape with pecten consisting of one to several hairs. Wingspan (males) 13–26 mm. Forewing with longitudinal white or grey streaks, and in some species with white costa, but without black spots. Known females (wingspan 10–30 mm) vary from fully winged to brachypterous.

Genitalia morphology. Male genitalia. Uncus longer than broad, with parallel outer margin, apex rounded; gnathos hook stout, massive, rarely moderately slender, curved, apically distinctly pointed; tegumen with broadly emarginated anterior margin, anteromedially with sclerotised ridge; valva extending to base of uncus, digitate, basally inflated, distal part tapered, apically weakly inflated, rounded, exceptionally narrowing; sacculus absent; saccus sub-triangular, longer than broad, posterior margin sinusoid, medial part smooth, without sclerotised ridge; phallus with inflated coecum, distal part with long sclerotised distodorsal ridge, with variable extent of dentation, ductus ejaculatorius without specialised sclerotisations.

Female genitalia. Papilla analis moderately small to large, apically rounded, rarely pointed; apophysis posterior of modest length, stout to moderately slender, sometimes with widening; segment VIII frequently with sclerotised posteriolateral part; subgenital plate with sub-triangular subostial sclerotisation, posteriorly with pointed sclerites of variable length, anterior edge medially with rounded to sub-triangular projection; apophysis anterior rod-like, extending from sclerotised widening at posterior margin of segment VIII, or reduced to free apical part; short colliculum or longer antrum present; signum of variable size, suboval spiny plate.

Diagnosticremarks. The *M.fallax* species group differs from the vast majority of *Megcraspedus* by the long and porrect labial palpus with reduced segment 3. It is furthermore defined by combined structures of the male genitalia, particularly the shape of the uncus with a parallel outer edge, the stout gnathos hook, the digitate valva with usually weakly inflated apex and the comparatively small, sub-triangular saccus without sclerotised ridges. The female genitalia vary in several characters and agree overall with some taxa in other species groups, but females are unknown for the majority of *Megacraspedus*.

The majority of species of the *M.fallax* species group are very similar in the male and, as far as known, female genitalia, usually differing at most by subtle characters. However, all these species clearly differ in phenotypic appearance, supported by DNA barcode divergence in several taxa.

#### Megacraspedus tabelli sp. n.

Taxon classificationAnimaliaLepidopteraGelechiidae

http://zoobank.org/3BEDAA5A-F0A5-44CE-9258-0A35EA8AE846

##### Examined material.

**Holotype** ♂, “MOROCCO Settat Prov. Sidi Said Machou 2.5 km E 1125 m, N33.14137 W8.09181 18.5.2016 J. Tabell leg.” “DNA Barcode TLMF Lep 21269” “GU 16/1452 ♂ P. Huemer” (ZMUC). **Paratype.** Morocco. 1 ♂, same data as holotype, but genitalia slide GEL 1261 Huemer (TLMF).

##### Description.

Adult. *Male* (Figure 72). Wingspan 14–15 mm. Labial palpus long, porrect, dark brown mottled with white, especially on inner and upper surface; segment 3 reduced. Antennal scape with pecten of one soft hair; flagellum dark brown, indistinctly lighter ringed. Head, thorax and tegula as forewing. Forewing light yellow-brown with white veins; fringes light grey, blackish grey at apex. Hindwing grey with grey fringes.

*Female*. Unknown.

*Variation*. The two examined specimens are similar except for being in different conditions.

*Male genitalia* (Figure 206). Uncus moderately broad, about two times longer than broad, with parallel outer margin apex sinusoid; gnathos hook stout, massive, about length of uncus, weakly curved; tegumen with broad and shallowly emarginated anterior margin, anteromedially additional emargination, anteromedially with sclerotised ridge; pedunculi small, suboval, posteriorly with sclerotised ridge; valva extending to base of uncus, basally inflated, digitate distal part strongly tapered, apically rounded; sacculus absent; posterior margin of vinculum shallowly emarginated, laterally with small hump; saccus sub-triangular, strongly tapered towards apex, slightly longer than broad, posterior margin sinusoid, medial part without sclerotised ridge, lateral sclerites slightly shorter than maximum width of saccus; phallus with inflated coecum, about twice width of distal part, long sclerotised distodorsal ridge, dentated.

*Female genitalia*. Unknown.

##### Diagnosis.

*Megacraspedustabelli* sp. n. is characterised by its light yellow-brown forewings with white veins. It may resemble *M.albovenata* (Figs 81–82) or *M.longipalpella* (Figs 83–84), but these species are larger and have black scales scattered on the forewings. The male genitalia largely agree with those of *M.numidellus* (Figs 210–212) and other members of the *M.fallax* species group but differ from *M.numidellus* by the more slender uncus and gnathos hook.

##### Molecular data.

BIN BOLD:ADF1917 (n = 2). The intraspecific divergence of the barcode region is low with maximum divergence of 0.6%. The distance to the nearest congeneric neighbour *M.numidellus* sp. n. is 9.8% (p-dist).

##### Distribution.

North-western Morocco.

##### Biology.

Host plant and early stages are unknown. The small type series was collected in middle of May at an altitude of 1125 m.

##### Etymology.

The species name (a noun in the genitive case) is dedicated to Jukka Tabell, Finland, who collected the type series of this species and significantly contributed to our work with most valuable material.

#### Megacraspedus gallicus sp. n.

Taxon classificationAnimaliaLepidopteraGelechiidae

http://zoobank.org/FA42AADE-F536-4C23-818F-1A2797A8EDDF

##### Examined material.

**Holotype** ♂, “SPAIN [prov.] Almería Sierra de Alhamilla 800–900 m route Huebro - Colotivi 1.5.2008 Zdenko Tokár leg.” “Gen. pr. Z. Tokár ♂ No 12180 [in glycerin]” “*Megacraspedus fallax* det. Zdenko Tokár” “DNA Barcode TLMF Lep 16638” (TLMF). **Paratypes.** France. 2 ♂, 1 ♀, Dep. Var, Esterel, Agay-Radier, 25.iv.1998, leg. J. Nel, genitalia slides 535 ♂ Nel, GEL 1201 ♂ Huemer; 1 ♀, Dep. Var, Correns, 400 m, 12–18.v.2007, leg. R. Seliger (TLMF); 1 ♂, Dep. Var, Puits de Rians, La Gardiole, 6.vi.1995, leg. J. Nel, genitalia slide GEL 1203 Huemer; 1 ♀, same data, but 13.v.2001; 1 ♂, Dep. Var, Mt. Caume, adret, 500 m, 26.iv.1998, leg. J. Nel; 1 ♂, Dep. Var, Mt. Faron, ligne de Crete Est, 25.v.1996, leg. J. Nel, genitalia slide 4999 Nel; 1 ♀, Dep. Var, Draguignan, 27.v.1993, leg. J. Nel, genitalia slide 0993; 1 ♀, Dep. Var/Bouches du Rhône, Puits de Rians, 13.v.2001, leg. J. Nel; 1 ♂, Dep. Vaucluse, Mt. Ventoux, Combe du Grande Barbeirol, 1.v.1992, leg. J. Nel, genitalia slide 1739 Nel; 1 ♀, 1 ♂, same data, but station CGB, 27.v.1995, genitalia slide 3493 ♀ Nel; 1 ♀, Dep. Vaucluse, Barroux, La Rabirette, 250 m, 28.iv.2001; 1 ♂, Dep. Vaucluse, Villes/Auzon, l´Ermitage, 6.v.2000, leg. J. Nel (all TLMF). Spain. 1 ♀, prov. Almería, 10 km E Bedar, El Pinar, 325 m, 19–27.iv.2001, leg. P. Skou & B. Skule (ZMUC); 1 ♂, 2 ♀, prov. Almería, Maria, 1200 m, 18–25.vi.2006, leg. M. Delnoye, genitalia slide 6513 Hendriksen (ZMUC); 1 ♂, prov. Almería, Sierra de Alhamilla, 700–800 m, 29.iv.2008, leg. Z. Tokár (RCZT); 1 ♂, 1 ♀, prov. Almeria, Sierra de Alhamilla, road Huebro – Colotivi, 800–900 m, 29.iv.2008, leg. J. Šumpich (NMPC); 1 ♀, prov. Almeria, Sierra de Alhamilla 10 km N Nijar, 7.v.2014, leg. A. Stübner (ZSM); 5 ♂, prov. Zaragoza, 8 km NE Gelsa, 240 m, 19.v.2016, leg. J. Tabell (ZMUC); 1 ♂, prov. Cuenca, Paracuelos, 5.vi.1986, leg. M. Hull (ZMUC); 1 ♂, 3 ♀, prov. Castellón, Benicàssim, 250 m, 5.v.2003, leg. J. Šumpich (MNCN, NMPC); 2 ♂, 1 ♀, prov. Cuenca, Monteagudo de las Salinas, 1000 m, 6.v.2008, leg. J. Šumpich (NMPC); 1 ♂, prov. Granada, Benamaurel, 9.vi.2011, leg. H. Rietz (ECKU); 1 ♀, prov. Murcia, 3 km E San Miguel de Salinas, 26.iv.2009, leg. J. Tabell; 1 ♂, 1 ♀, prov. Teruel, Sierra de San Just, Aliega, 1350 m, 27.v.2003, leg. J. Procházka (NMPC); 1 ♀, prov. Teruel, 7 km SE Albarracin, 1400 m, 27.vi.1992, leg. P. Skou; 1 ♀, prov. Teruel, Albarracin, 1170 m, 8–10.vi.1994, leg. A. Cox (all ZMUC); 1 ♂, same data, but 1100 m, 3.v.2003, leg. J. Šumpich (NMPC); 1 ♀, prov. Teruel, Sierra de Albarracin, Sierra Alta, 1750 m, 25.vi.2016, leg. J. Viehmann (RCWS); 1 ♂, 2 ♀, prov. Zaragoza, 6 km W Bujaraloz, 300 m, 29.v.2015, leg. J. Viehmann (RCWS, ZMUC).

##### Description.

**Figures 73–78.**
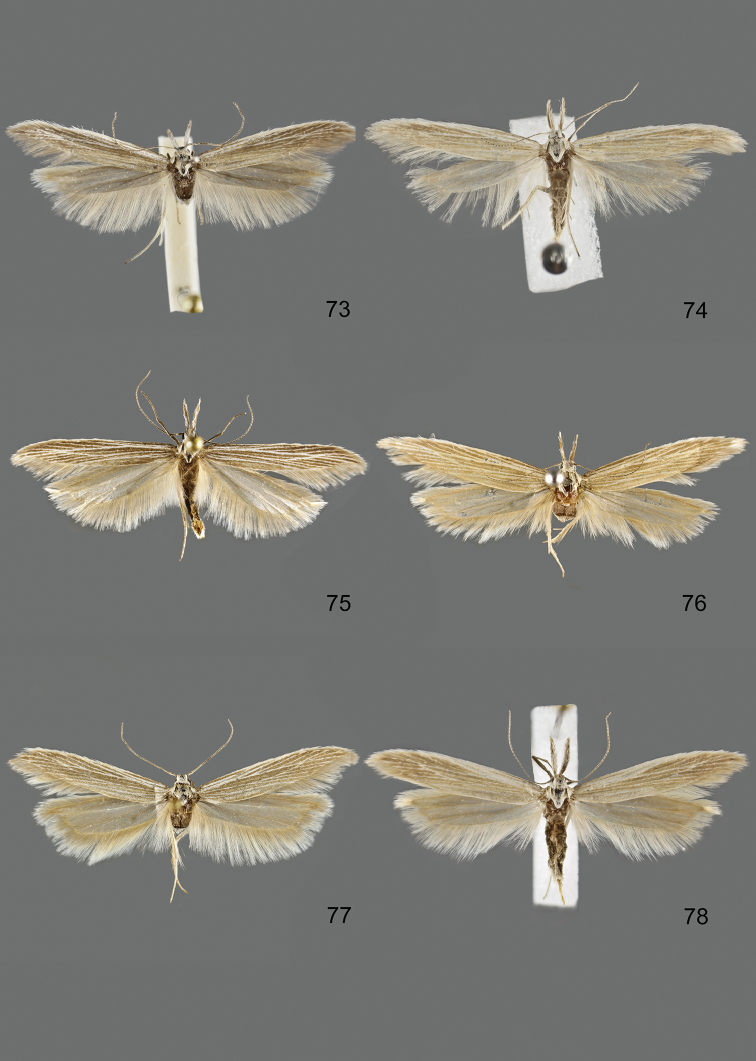
*Megacraspedus* adults in dorsal view. **73***M.gallicus* sp. n. – Holotype, male, Spain (TLMF) **74***M.gallicus* sp. n. – Paratype, female, France (TLMF) **75***M.ribbeella* (Caradja, 1920) – male, Spain (ZMUC) **76***M.ribbeella* (Caradja, 1920) – female, Spain (NHMW) **77***M.libycus* sp. n. – Holotype, male, Libya (ZMUC) **78***M.libycus* sp. n. – Paratype, female, Morocco (ZSM).

Adult. *Male* (Figure 73). Wingspan 17–20 mm. Labial palpus long, porrecting, brown mottled with white, especially on upper and lower surface; segment 3 reduced. Antennal scape with pecten of 1–2 soft hairs; flagellum light brown, indistinctly darker ringed. Head and thorax light brown; tegula cream. Forewing light grey-brown; costa white with blackish brown at base; veins in costal half white; a small white spot at base of termen; fringes grey. Hindwing grey with grey fringes.

*Female* (Figure 74). Wingspan 18–22 mm. Forewing whitish brown, resulting in less contrasting white veins in costal half of the wing. Otherwise similar to male.

*Variation*. The forewing colour of both males and females can be lighter or darker brownish.

*Male genitalia* (Figure 207). Uncus oblong, approximately 1.9 times longer than broad, with parallel outer margin, apex curved and tapered to medio-apical tip; gnathos hook stout, about length of uncus, curved distal half, with pointed apex; anterior margin of tegumen with moderately broad and deep V-shaped emargination, anteromedial part of tegumen with short longitudinal sclerotised ridge; pedunculi large, suboval, with additional rounded sclerite; valva moderately slender, almost same width throughout, extending to posterior third of uncus, apically rounded, setose; saccular area covered with setae, without separated sacculus; posterior margin of vinculum shallowly emarginated, laterally with indistinct hump, vincular sclerite basally sub-rectangular, distal part oblong, with sclerotised posteriomedial edge; saccus sub-triangular, moderately long, abruptly tapered from about middle to pointed apex, ratio maximum width to length 0.75, posterior margin arched, with weak medial emargination, medial part smooth, without sclerotised ridge, lateral sclerites about maximum width of saccus; phallus with inflated coecum, about twice width of distal part, long sclerotised distodorsal ridge with few short spines in apical part.

*Female genitalia* (Figure 280). Papilla analis medium-sized, apically rounded; apophysis posterior slender rod-like, approximately 2 mm long, bordered by large sclerotised field posteriorly, weak curvation at about one-third; segment VIII approximately 0.6 mm long, membranous; subgenital plate with band-like subostial sclerotisation, with broadly V-shaped projection anteriorly, posteriorly extended into very long, pointed sub-medial sclerites, delimiting oblong ostium bursae, anterior margin with rod-like edge connected with apophysis anterior; apophysis anterior slender, rod-like, almost length of segment VIII, posteriorly becoming rod-like venula of segment VIII, distinctly widening to oblong sclerotised zone, extending to posterior margin of segment VIII; antrum well sclerotised, approximately 0.6 mm length; ductus bursae gradually widening to weakly delimited suboval corpus bursae, entire length of ductus and corpus bursae approximately 3.5 mm; signum moderately small, suboval spiny plate.

##### Diagnosis.

*Megacraspedusgallicus* sp. n. is characterised by its relatively large size, its long labial palps and the light brownish forewings with white veins in costal half. It can be confused with *M.fallax* (Figure 89), but that species has a distinct, silvery white sub-costal streak on the forewings. *M.ribbeella* (Figs 75–76) is larger and has contrasting brownish and white forewings. The male genitalia are similar overall to other species of the *M.fallax* species group but differ from most taxa in the posterior margin of the saccus without medial emargination. This character is only shared with a few species, particularly the very similar *M.longipalpella* (Figure 214), from which *M.gallicus* sp. n. differs e.g., by the stout gnathos hook and the more slender saccus. The female genitalia are very similar to *M.libycus* sp. n. (Figure 282), differing in particular in the apically rounded papilla analis and subtle characters such as the shorter apophyis posterior and the more strongly sclerotised antrum. However, the diagnostic value of these character stages is uncertain and may be due to individual variation. The species furthermore differs from the related *M.ribbeella* (Figure 281) e.g., by the smaller papilla analis, the rod-like anterior margin of segment VIII, and the smaller signum.

##### Molecular data.

BIN BOLD:ACF7111 (n = 5). The intraspecific divergence of the barcode region is moderate with mean 0.7% and maximum divergence of 1.1%. The distance to the nearest neighbour *M.ribbeella* is 6.2% (p-dist).

##### Distribution.

Southern France, Spain.

##### Biology.

Host plant and early stages are unknown. The adults have been collected from late April to late June at light at altitudes up to 1400 m.

##### Etymology.

This species is named after one of its places of occurrence: the Latin *Gallia* which included present-day France and adjacent territories. The name is a masculine adjective.

#### Megacraspedus ribbeella

Taxon classificationAnimaliaLepidopteraGelechiidae

(Caradja, 1920) comb. n.

Nevadia ribbeella Caradja, 1920: 118.

##### Examined material.

**Holotype** ♀, [Spain] “*Nevadia ribbeella*”, “HOLOTYPE *Vadenia* (=*Nevadia*) *ribbeella* Car. ♀ ROMÄNIA” “S^ra^ Nevada” “Genit. praep. ♀ N 187797 Euparal 2003 Ponomarenko” (MGAB) [photographs examined]. **Non-type material.**Spain. 1 ♀, prov. Granada, Sierra Nevada, Ruta del Valeta, 1650 m, 25.vi.1986, leg. P. Skou (ZMUC); 1 ♀, prov. Sierra de Los Guájares, 1160 m, 26.vi.–6.vii.2007, leg. G. Jeppesen (ZMUC); 1 ♂, 1 ♀, prov. Murcia, Sierra de Espuña, 1909, leg. Korb, genitalia slide Mus. Vind. 16.650 ♂, NM 16.651 ♀ (NHMW); 1 ♀, prov. Murcia, Sierra de Espuña, 8 km W Alhama, 1000 m, 26.v.1998, leg. P. Skou (ZMUC); 1 ♀, prov. Murcia, 3 km E San Miguel de Salinas, 12.iv.2009, leg. J. Tabell (TLMF); 1 ♀, same data, but 3 km SW San Miguel de Salinas, 16.iv.2009 (ZMUC); 1 ♂, prov. Murcia, 7 km NW Sucina, Sierra de Alteona, 360 m, 27.iv.2009, leg. J. Tabell (ZMUC).

##### Redescription.

Adult. *Male* (Figure 75). Wingspan 22–26 mm. Labial palpus long, porrect, dark brown mottled with white, especially on upper and lower surface; segment 3 reduced. Antennal pecten consisting of 1–3 hairs; flagellum greyish brown, indistinctly ringed with white. Head, thorax and tegula light greyish brown, thorax with dark streak in middle. Forewing brown; veins and narrow streak along costa and dorsum white; fringes grey. Hindwing grey with grey fringes.

*Female* (Figure 76). Wingspan 19–30 mm. Similar to male.

*Variation*. Very variable in size. The dark parts of the forewings can be lighter or darker brown. One specimen has a broader white streak along the costa. The dark streak on the thorax is often obsolete because specimens tend to become greasy. Worn specimens become lighter.

*Male genitalia* (Figure 208). Uncus broad, approximately 1.3 times longer than broad, with parallel outer margin, apex evenly curved; gnathos hook stout, slightly longer than uncus, curved, with pointed apex; anterior margin of tegumen with moderately broad and deep V-shaped emargination, anteromedial part of tegumen with short longitudinal sclerotised ridge; pedunculi large, suboval, with additional rounded sclerite; valva moderately slender, extending to posterior third of uncus, basally broader, medially slightly constricted, apically weakly inflated, setose; saccular area covered with setae, without separated sacculus; posterior margin of vinculum shallowly emarginated, laterally with indistinct hump, sub-rectangular vincular sclerite with sclerotised posteriomedial edge; saccus sub-triangular, moderately long, evenly tapered to pointed apex, ratio maximum width to length approximately 0.75, posterior margin arched, without medial emargination, medial part smooth, without sclerotised ridge, lateral sclerites shorter than maximum width of saccus; phallus with weakly inflated coecum, about twice width of distal part, long sclerotised distodorsal ridge with few short spines in apical part.

*Female genitalia* (Figure 281). Papilla analis moderately large, apically broadly rounded; apophysis posterior slender rod-like, approximately 1.6 mm long, bordered by sclerotised field posteriorly, weak curvation and widening at about one-third; segment VIII approximately 0.5 mm long, membranous; subgenital plate with band-like subostial sclerotisation, with slender and moderately long V-shaped projection anteriorly, posteriorly extended into very long, pointed sub-medial sclerites, delimiting oblong ostium bursae, anterior margin with band-like edge connected with apophysis anterior; apophysis anterior slender, rod-like, about length of segment VIII, posteriorly becoming band-like venula of segment VIII, distinctly widening to oblong sclerotised zone, extending to posterior margin of segment VIII; antrum slender, sclerotised, approximately 0.3 mm length; ductus bursae gradually widening to weakly delimited suboval corpus bursae, entire length of ductus and corpus bursae about 4 mm; signum moderately large, suboval spiny plate.

##### Diagnosis.

*Megacraspedusribbeella* is characterised by its large size, and by its contrasting brownish and white forewings. Small specimens resemble *M.albovenata* (Figs 81–82) or *M.longipalpella* (Figs 83–84), but these species have the dark parts of the forewings yellowish, dusted with brown scales. It is furthermore similar to *M.gallicus* sp. n. (p 101). The male genitalia are similar overall to other species of the *M.fallax* species group but differ from most taxa in the posterior margin of the saccus without medial emargination. This character is only shared with a few species, particularly the very similar *M.longipalpella* (Figure 284), from which *M.ribbeella* differs e.g., by the stout gnathos hook and the more slender saccus. The female genitalia are similar to *M.gallicus* sp. n. (Figure 280) differing in several characters such as the distinctly larger papilla analis, the nearly band-like anterior margin of segment VIII, the more slender and less sclerotised antrum, and the larger signum.

##### Molecular data.

BIN BOLD:ACZ9288 (n = 2). The intraspecific divergence of the barcode region is low with maximum divergence of 0.2%. The distance to the nearest neighbour *Megacraspedusgallicus* sp. n. is 6.2% (p-dist) (p-dist).

##### Distribution.

Southern Spain.

##### Biology.

Host plant and early stages are unknown. The adults have been collected from late May to late June at light at altitudes up to 1650 m.

##### Remarks.

*Nevadiaribbeella* was described from one specimen from Sierra Nevada, Spain (Caradja 1920). In the original description it was stated to be a male, but according to Popescu-Gorj (1992) and photographs of the holotype kindly supplied by M. Stanescu (MGAB) it is a female.

#### Megacraspedus libycus sp. n.

Taxon classificationAnimaliaLepidopteraGelechiidae

http://zoobank.org/B69F11E7-9A82-4537-B9A4-9B357F16A43F

##### Examined material.

**Holotype** ♂, “[northern] LIBYA, Gharian Wadi el Hira, 7.iv.1983 Uffe Seneca” “GU 16/1435 ♂ P. Huemer” (ZMUC). **Paratypes.** Libya. 1 ♀, Gharian, Wadi el Hira, 25.iii.1983, leg. U. Seneca; 2 ♂, 1 ♀, same data, but 7.iv.1983, genitalia slides 6514 Hendriksen; 1 ♂, 2 ♀, same data, but 12.iv.1983, GU 17/1477 ♀ Huemer; 1 ♀, same data, but 15.iv.1984; 2 ♀, same data, but 22.iv.1983; 1 ♂, 1 ♀, same data, but 30.iv.1983 (all ZMUC). Morocco. 1 ♂, 2 ♀, Middle Atlas, Ifrane, 25.vi.1972, leg. F. Hahn, GU 18/1504 ♀ Huemer (SMNK); 2 ♀, same data, but 30.vi.1972, genitalia slide GEL 1266 ♀ Huemer (TLMF, ZSM).

##### Description.

Adult. *Male* (Figure 77). Wingspan 23–26 mm. Labial palpus long, porrect, dark brown mottled with white, especially on upper and lower surface; segment 3 reduced. Antennal pecten consisting of 1–3 hairs; flagellum greyish brown, indistinctly ringed with white. Head, thorax and tegula light greyish brown, thorax with dark streak in middle. Forewing brown; veins and narrow streak along costa and dorsum white; fringes grey. Hindwing grey with grey fringes.

*Female* (Figure 78). Wingspan 19–25 mm. Similar to male.

*Variation*. The examined specimens exhibit only slight variation. The dark streak on the thorax is often obsolete because specimens tend to be greasy. Worn specimens become lighter. Specimens from Morocco are generally lighter both on the forewings and hindwings.

*Male genitalia* (Figure 209). Uncus moderately broad, sub-rectangular, about two times longer than wide, apical corners rounded, apicomedially nearly pointed; gnathos hook stout, slightly shorter than uncus, evenly curved with pointed apex; anterior margin of tegumen with moderately broad and deep V-shaped excavation, medially with longitudinal sclerotised ridge from anterior edge to about middle; pedunculi large, suboval, with additional rounded sclerite; valva moderately stout, extending to posterior third of uncus, apex slightly swollen, weakly rounded, setose; saccular area covered with setae, without separated sacculus; posterior margin of vinculum with shallow medial emargination, weak lateral humps, elongated suboval vincular sclerite with strongly sclerotised posteriomedial edge; saccus long, irregularly V-shaped, basally broad, abruptly tapered to pointed apex at one-third, ratio maximum width to length approximately 0.6, posterior margin arched, without medial emargination, medial part smooth, without sclerotised ridge, lateral sclerites long and slender, about maximum width of saccus; phallus straight, with bulbous coecum, distal two-thirds slender, rod-like, sclerotised ridge with few minute subapical thorns.

*Female genitalia* (Figure 282). Papilla analis medium-sized, apically weakly pointed; apophysis posterior slender rod-like, approximately 2 mm long, bordered by large sclerotised field posteriorly, weak curvation at about one-third; segment VIII approximately 0.7 mm long, membranous; subgenital plate with band-like subostial sclerotisation, with broadly V-shaped projection anteriorly, posteriorly extended into very long, pointed sub-medial sclerites, delimiting oblong ostium bursae, anterior margin with rod-like edge connected with apophysis anterior; apophysis anterior slender, rod-like, almost length of segment VIII, posteriorly becoming rod-like venula of segment VIII, distinctly widening to oblong sclerotised zone, extending to posterior margin of segment VIII; antrum partially sclerotised, approximately 0.6 mm length; ductus bursae gradually widening to weakly delimited suboval corpus bursae, entire length of ductus and corpus bursae about 3 mm; signum moderately small, suboval spiny plate.

##### Diagnosis.

*Megacraspeduslibycus* sp. n. is characterised by its large size, and by its contrasting brownish and white forewings. Externally it is hardly separable from *M.ribbeella* (Figs 75–76). The male genitalia are overall similar to other species of the *M.fallax* species group, particularly *M.ribbeella* (Figure 208), but differ in particular in the elongated saccus and the peculiar shape of the uncus. The female genitalia largely resemble those of *M.gallicus* sp. n. (Figure 280) but differ e.g., by the apically weakly pointed papilla analis. They are separable from *M.ribbeella* (Figure 281) by several characters such as the rod-like medial part of the apophysis anterior and the rod-like anterior edge of segment VIII as well as the distinctly smaller signum.

##### Molecular data.

Not available, barcoding failed.

##### Distribution.

Libya, Morocco (Middle Atlas).

##### Biology.

Host plant and early stages are unknown. The adults have been collected from late March to the end of April at light, at higher altitudes in late June.

##### Etymology.

This species is named after its place of occurrence, Libya. The name is an adjective.

##### Remarks.

Females from Morocco fully agree with material from Libya in the genitalia, whereas one male is slightly different in having a shorter saccus.

#### Megacraspedus numidellus

Taxon classificationAnimaliaLepidopteraGelechiidae

(Chrétien, 1915) comb. n.

Chilopselaphus numidellus Chrétien, 1915: 333.
Megacraspedus mareotidellus Turati, 1924: 169, pl. 6, fig. 15, syn. n.

##### Examined material.

**Lectotype** ♂, *Chilopselaphusnumidellus*, **here designated**, “TYPE” “*Chilopselaphus numidellus*” “Dj. Gafsa 28.4.09” genitalia slide PGCGN 8017 (MNHN) [photographs examined]. **Syntype** ♂, *Megacraspedus mareotidellus*, “PARA-LECTO-TYPE” “Paralectotype ♂ *Megacraspedus mareotidellus* Tur. teste. K. Sattler, 1980” “Cyrenaika Bengasi 15.iii.22. Geo.C.Krüger” “Mus. TURATI coll. HARTIG” “E. Turati Coll. F. Hartig Coll. B.M. 1979–141” “BMNH ♂ Genitalia slide No. 33664” (BMNH). **Non-type material.** Libya. 1 ♂, Cyrenaica, R. U. Agrario, 20.iii.1925, leg. G. C. Krüger (ZMUC); 1 ♂, Gharian, Wadi El Hira, 25.iii.1983, leg. U. Seneca; 4 ♂, same data, but 7.iv.1983, genitalia slide 5335 Karsholt (ZMUC). Morocco. 1 ♂, Anti Atlas, prov. Tiznit, 3.2 km SSW Mirleft, 10 m, 12.iv.2015, leg. J. Tabell, genitalia slide 5336 Karsholt (ZMUC); 1 ♂, Anti Atlas, prov. Tiznit, 24 km SW Tafraout, 1125 m, 13.iv.2015, leg. J. Tabell (ZMUC). Spain. 3 ♂, Fuerteventura, Jandia, Bco. Esquinzo, 15.ii.-26.iv.2004, leg. Paas, genitalia slides GU 16/1424 ♂ Huemer, 5334 Karsholt; 1 ♂, same data, but 17.ii.-8.iii.2002, leg. Paas; 1 ♂, same data, but 9.iii.-10.iv.2002; 1 ♂, same data, but 12–28.ii.2003; 2 ♂, same data, but 30.iii.-20.iv.2003; 2 ♂, same data, but 20.iii.-19.iv.2005; 1 ♂, 20.iv.-1.v.2005 (RCWS, TLMF, ZMUC); 1 ♂, Fuerteventura, Caldereta, 120 m, 27.ii.-19.iii.2018 leg. P. Falck (RCPF).

##### Redescription.

**Figures 79–84.**
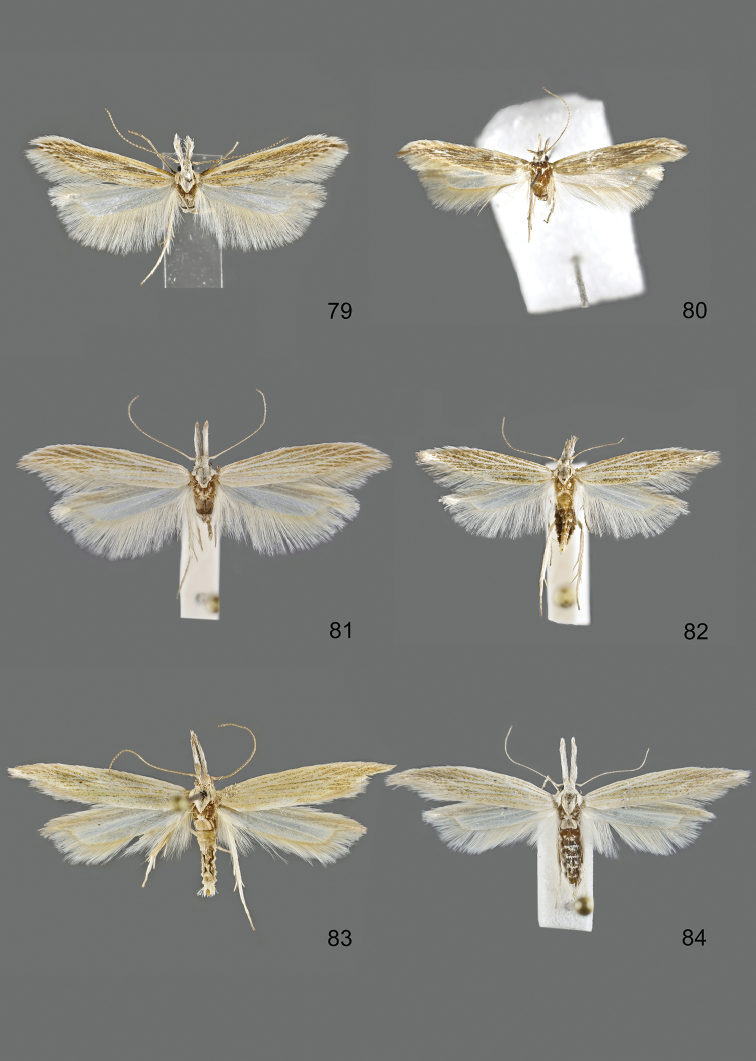
*Megacraspedus* adults in dorsal view. **79***M.numidellus* (Chrétien, 1915) – male, Morocco (ZMUC) **80***M.numidellus* (Chrétien, 1915) – male, Spain (ZMUC) **81***M.albovenata* Junnilainen, 2010 – male, Czech Republic (TLMF) **82***M.albovenata* Junnilainen, 2010 – female, Czech Republic (TLMF) **83***M.longipalpella* Junnilainen, 2010 – male, Ukraine (ZMKU) **84***M.longipalpella* Junnilainen, 2010 – female, Russia (ZMUC).

Adult. *Male* (Figs 79–80). Wingspan 12–17 mm. Labial palpus long (about one-quarter to one-fifth the length of antenna), porrecting, brown mottled with white on outer surface, white mottled with brown on inner surface, white on upper and lower surface; segment 3 reduced. Antennal scape with pecten consisting of several hairs; flagellum indistinctly ringed white and dark, lightest near base. Head light brown in middle, laterally white; thorax brown mottled with white; tegula yellowish brown. Forewing yellowish brown, partly mottled with blackish brown scales; veins white; a thin white sub-costal stripe followed by a yellow stripe from base to apex; a yellow stripe in fold; a white patch between 3/5 and 4/5 in middle of wing; termen black interrupted by white; fringes light grey, blackish grey at apex. Hindwing whitish grey with concolorous fringes.

*Female*. Unknown.

*Variation*. The few examined specimens from Northern Africa are rather worn with a tendency of white rather than yellow stripes. These stripes also show some variation in extension and the amount of blackish brown scales on the forewing is also variable. The patch between 3/5 and 4/5 in middle of the forewing wing is sometimes yellowish.

*Male genitalia* (Figs 210–212). Uncus approximately 1.5 to 2 times longer than broad, weakly widened posteriad, apex projected, sinusoid; gnathos hook stout, slightly shorter than uncus, distal part curved with pointed apex; tegumen with broad and shallowly emarginated anterior margin, anteromedially small additional emargination; pedunculi sub-triangular, posteriorly with sclerotised ridge; valva extending to slightly beyond middle of uncus, basally broader, digitate distal part tapered, apically weakly inflated; sacculus absent; posterior margin of vinculum shallowly emarginated, laterally with indistinct humps; saccus sub-triangular, moderately long, abruptly tapered towards pointed apex, posterior edge sinusoid, medial part without sclerotised ridge, lateral sclerites slightly shorter than maximum width of saccus; phallus with weakly inflated coecum, about twice width of distal part, distal two-thirds moderately broad, straight, subapically with long sclerotised distodorsal ridge on right-hand side with or without short dentation.

*Female genitalia*. Unknown.

##### Diagnosis.

*Megacraspedusnumidellus* is characterised by its moderately long labial palps with reduced segment 3, by the antennal scape with the pecten consisting of several hairs and by the forewings having three white streaks, but no black dots. The male genitalia are similar to *M.albovenata* (Figure 213) and differ only slightly e.g., by the stouter valva and the sclerotisations of the phallus.

##### Molecular data.

BIN BOLD:ADA0605 (n = 1). The distance to the nearest neighbour *M.gallicus* sp. n. is 9% (p-dist).

##### Distribution.

Libya, Morocco, Spain (Canary Islands: Fuerteventura), Tunisia.

##### Biology.

Host plant and early stages are unknown. The few specimens known to date were collected from middle of February to early May April at altitudes from 10 to 1125 m.

##### Remarks.

*Chilopselaphusnumidellus* was described from an unspecified number of specimens (probably more than one as the wingspan is stated to be “15–16 mm”) collected at Gafsa in Tunisia in May. It was compared with *C.fallax* Mann (Chrétien 1915). We have been able to examine photographs of a syntype from MNHN. This specimen is here designated as the lectotype in order to fix the identity of the species and conserve stability of nomenclature. *Megacraspedusmareotidellus* was described from eleven specimens collected at El Berca, Benghazi, Libya (Turati 1924: 169). It was compared with “*oranellus* B.H.”, but we have been unable to trace this name in the literature. A male labelled as a paralectotype by K Sattler and considered to be a syntype fully corresponds with figures of a syntype of *M.numidellus* and we thus consider *M.mareotidellus* to be a junior synonym of the former (syn. n.).

#### Megacraspedus albovenata

Taxon classificationAnimaliaLepidopteraGelechiidae

Junnilainen, 2010

Megacraspedus albovenata Junnilainen, 2010, *in* Junnilainen & Nupponen, 2010: 1, figs 22, 28.

##### Examined material.

**Paratypes.** Russia. 3 ♂, S Ural, Cheliabinsk district, Arkaim reserve near Amurskii village, 16.vi.1996, leg. K. Nupponen, J.-P. Kaitila, J. Junnilainen & M. Ahola (RCKN, ZMUC); 1 ♂, same data, but 17.vi.1996 (ZMUC); 1 ♂, same data, but 18.vi.1996 (ZMUC); 1 ♂, same data, but 19.vi.1996 (RCKN); 3 ♂, same data, but, Troizkii reserve near Berlin village, 30.vi.1997, leg. K. Nupponen & J. Junnilainen, genitalia in vial (RCKN, ZMUC); 1 ♂, same data, but 2.vii.1997 (RCKN). **Non-type material.** Czech Republic. 5 ♂, 1 ♀, Moravia, Znojmo distr., Ječmeništĕ, 250 m, 11.vi.2011, leg. J. Šumpich, genitalia slides GEL 1210 ♂ Huemer, GU 17/1474 ♀ Huemer (NMPC, TLMF, ZMUC). Russia. 1 ♂, S Ural, Cheliabinsk distr., Ustinovo village, 25.vi.2016, leg. H. Roweck & N. Savenkov; 1 ♂, S Ural, Cheliabinsk distr., Kizilskoye village, 1.vii.2017, leg. H. Roweck & N. Savenkov; 9 ♂, S Ural, Cheliabinsk distr., Uvelsk reg., Mihiri village, Zhemeryak river, 1–2.vii.2016, leg. H. Roweck & N. Savenkov; 14 ♂, S Ural, Cheliabinsk distr., Oktyabrskoya, Kocherdyk reserve, 3–4.vii.2016, leg. H. Roweck & N. Savenkov (all ECKU).

##### Redescription.

Adult. *Male* (Figure 81). Wingspan 15–18 mm. Labial palpus long, about one-third length of antenna, porrect, white with medial part of inner and outer surface dark brown; segment 3 reduced. Antennal scape with pecten consisting of a few hairs; flagellum greyish brown, indistinctly ringed with white. Head, thorax, and tegula white. Forewing light yellow mottled with brown and black scales especially in costal and apical part; veins pure white; fringes light grey. Hindwing white with white fringes.

*Female* (Figure 82). Wingspan 15 mm. Similar to male apart from slightly more slender and pointed wings.

*Variation*. The examined specimens show only slight variation, and specimens from central Europe are similar to those from the southern Urals.

*Male genitalia* (Figure 213). Uncus moderately slender, sub-rectangular, approximately 1.5 times longer than wide, distally rounded; gnathos hook massive, slightly longer than uncus, distally curved with pointed apex; anterior margin of tegumen with broad and moderately shallow excavation, medially with additional small emargination, longitudinal sclerotised ridge from anterior edge to posterior third; pedunculi small, rounded; valva moderately slender, exceeding base of uncus, apex weakly rounded, setose; saccular area covered with setae, without separated sacculus; posterior margin of vinculum with shallow medial emargination, without lateral humps, suboval vincular sclerite with strongly sclerotised posterior edge; saccus sub-triangular, basally broad, distally tapered to pointed apex, ratio maximum width to length about 1, posterior margin with weakly sinusoid mediolateral projections, separated by shallow emargination, medial part smooth, without sclerotised ridge, lateral sclerites long and slender, slightly shorter than maximum width of saccus; phallus straight, with bulbous coecum, distal two-thirds slender, rod-like, with weakly curved lateral sclerotisation, few minute subapical thorns.

*Female genitalia* (Figure 283). Papilla analis strongly sclerotised, large, apically constricted, rounded; apophysis posterior slender rod-like, moderately short, approximately 1.5 mm long, basal fifth weakly curved and widened anteriad; segment VIII approximately 0.6 mm long, about same width, large sclerotised dorso- and ventrolateral zone, medially largely membranous; subgenital plate with band-like subostial sclerotisation, with broadly sinusoid projection anteriorly, posteriorly extended into moderately long, basally widened, distally pointed sub-medial sclerites, delimiting oblong ostium bursae, anterior margin with rod-like edge connected with apophysis anterior; apophysis anterior moderately stout, rod-like, about length of segment VIII, posteriorly becoming band-like venula of segment VIII, distinctly widening to large sclerotised zone, extending to posterior margin; colliculum short, sclerotised; ductus bursae gradually widening to weakly delimited, slender corpus bursae, entire length of ductus and corpus bursae approximately 2.7 mm; signum moderately large, transverse, sub-triangular spiny plate.

##### Diagnosis.

*Megacraspedusalbovenata* is characterised by its light yellow, brownish dusted forewings with clear white veins, giving it a striped look. It is similar to *M.longipalpella* (Figs 83–84) and *M.kazakhstanicus* sp. n. (Figure 93) and furthermore to *M.ribbeella* (p 103) and *M.tabelli* sp. n. (p 100). The male genitalia are characterised by the shape of the uncus in combination with the sub-triangular saccus. *M.albovenata* is very similar to *M.numidellus* (Figs 210–212) in these and several other characters but differs from the latter by the more stout valva. It furthermore largely resembles *M.longipalpella* (Figure 214) only differing in subtle characters such as the apex of the valva. The female genitalia are very similar to *M.longipalpella* (Figure 284) and mainly differ in subtle characters such as the longer apophysis posterior and anterior, the sinusoid anteriomedial projection of the segment VIII and the smaller colliculum.

##### Molecular data.

BIN BOLD:ACE2688 (n = 3). The intraspecific divergence of the barcode region is moderate with mean 0.9% and maximum divergence of 1.4%. The distance to the nearest neighbour *M.kazakhstanicus* is 5.1% (p-dist).

##### Distribution.

Czech Republic, Russia (S Ural).

##### Biology.

Host plant and early stages are unknown. The type series was collected in June. The adults fly at night and come to artificial light. The habitat is grassy steppe (Junnilainen and Nupponen 2010).

##### Remarks.

*Megacraspedusalbovenata* was described from numerous males collected in the southern Ural Mountains, Russia (Junnilainen and Nupponen 2010).

#### Megacraspedus longipalpella

Taxon classificationAnimaliaLepidopteraGelechiidae

Junnilainen, 2010

Megacraspedus longipalpella Junnilainen, 2010, *in*Junnilainen and Nupponen 2010: 16, figs 23, 29.

##### Examined material.

**Paratypes.** Russia. 5 ♂, S Ural, Cheliabinsk district, Troizkii reserve near Berlin village, 30.vi.1997, leg. K. Nupponen & J. Junnilainen, genitalia slides no. 1/7.IV.2008 K. Nupponen, 02022402 J. Junnilainen (RCKN, ZMUC); 1 ♂, same data, but 1.vii.1997; 1 ♂, same data, but 2.vii.1997; 1 ♂, S-Ural, Orenburg oblast, 20 km S Pokrovka village, Schibendy valley, 2.vii.2003, leg. K. Nupponen (all ZMUC); 3 ♂, Orenburg oblast, near Burannoe village, 3.vii.2003, leg. K. Nupponen, genitalia slide GU 15/1398 Huemer (RCKN, ZMUC). **Non-type material.** Russia. 1 ♂, S-Ural, Kidriasovo env., 21.vi.2009, leg. J. Šumpich (NMPC); 10 ♂, same data, but 1.vii.2017, leg. H. Roweck & N. Savenkov (ECKU, ZMUC); 1 ♂, S Ural, Cheliabinsk distr., Ustinovo village, 25.vi.2016, leg. H. Roweck & N. Savenkov (ECKU); 20 ♂, 1 ♀, S Ural, Cheliabinsk distr., Uvelsk reg., Mihiri village, Zhemeryak river, 1–4.vii.2016, leg. H. Roweck & N. Savenkov (ECKU, ZMUC); 10 ♂, S Ural, Cheliabinsk distr., Oktyabrskoje, Selitkul reserve, 5–6.vii.2016, leg. H. Roweck & N. Savenkov (ECKU, ZMUC). Ukraine. 1 ♂, Lugansk region, Melovoi distr., Streltsovskaya steppe Nat.Res., 5.vii.2002, leg. A. Bidzilya; 1 ♂, same data, but 6.vii.2002; 1 ♀, same data, but 10.vii.2002, genitalia slide GU 17/1475 Huemer (RCAB).

##### Redescription.

Adult. *Male* (Figure 83). Wingspan 18–20 mm. Labial palpus very long, almost half length of antenna, porrect, white with medial part of inner and outer surface brown; segment 3 reduced. Antennal scape with up to 5 long hairs; flagellum light brown, indistinctly ringed with blackish brown. Head, thorax and tegula light yellowish white. Forewing light yellow mottled with brown scales especially in apical part; veins pure white; fringes light grey. Hindwing whitish grey, darker towards costa, with white fringes.

*Female* (Figure 84). Wingspan 19–22 mm. Similar to male.

*Variation*. The examined specimens show only slight variation.

*Male genitalia* (Figure 214). Uncus moderately slender, sub-rectangular, approximately 1.5 times longer than wide, distally evenly rounded; gnathos hook moderately slender, slightly longer than uncus, distally curved with pointed apex; anterior margin of tegumen with broad and moderately shallow excavation, medially with additional small emargination, longitudinal sclerotised ridge from anterior edge to posterior third; pedunculi large, rounded; valva moderately slender, extending to middle of uncus, apex slightly swollen, weakly rounded, setose; saccular area covered with setae, without separated sacculus; posterior margin of vinculum with shallow medial emargination, without lateral humps, suboval vincular sclerite with strongly sclerotised posterior edge; saccus sub-triangular, basally broad, distally tapered to pointed apex, ratio maximum width to length approximately 0.9, posterior margin arched, without medial emargination, medial part smooth, without sclerotised ridge, lateral sclerites long and slender, slightly shorter than maximum width of saccus; phallus straight, with bulbous coecum, distal two-thirds slender, rod-like, with weakly curved lateral sclerotisation, few minute subapical thorns.

*Female genitalia* (Figure 284). Papilla analis strongly sclerotised, large, apically constricted, rounded; apophysis posterior slender rod-like, moderately short, approximately 2.2 mm long, basal sixth weakly curved and widened anteriad; segment VIII approximately 0.8 mm long, about same width, large sclerotised dorso- and ventrolateral zone, medially largely membranous; subgenital plate with band-like subostial sclerotisation, with small sub-triangular projection anteriorly, posteriorly extended into moderately long and basally widened, distally pointed sub-medial sclerites delimiting oblong ostium bursae, anterior margin a sclerotised edge connected with apophysis anterior; apophysis anterior moderately stout, rod-like, longer than segment VIII, approximately 1.1 mm, posteriorly becoming band-like venula of segment VIII, distinctly widening to large sclerotised zone, extending to posterior margin; colliculum moderately short, sclerotised; ductus bursae gradually widening to weakly delimited, slender corpus bursae, entire length of ductus and corpus bursae approximately 3.5 mm; signum moderately large, transverse, sub-triangular spiny plate.

##### Diagnosis.

*Megacraspeduslongipalpella* is characterised by its light yellow, brownish dusted forewings with clear white veins. It is similar to *M.albovenata* (Figs 81–82), but is larger and has longer labial palps (almost half length of antennae, whereas about one-third length of antennae in *M.albovenata*). See also *M.ribbeella* (p 103) and *M.tabelli* sp. n. (p 100). The male genitalia are very similar to other species of the *M.fallax* species group, particularly *M.albovenata* (Figure 213) and *M.niphorrhoa* (Figure 215) and differ only in subtle characters such as the slightly swollen apex of the valva, the stouter gnathos hook and the shorter and more rounded uncus. The female genitalia are very similar to *M.albovenata* (Figure 283) and differ only by subtle characters such as the longer apopohysis posterioris and anterioris, the small anteriomedial projection of segment VIII and the larger colliculum.

##### Molecular data.

BIN BOLD:ACM1349 (n = 1). The distance to the nearest neighbour *M.niphorrhoa* is 4.8% (p-dist).

##### Distribution.

Russia (S Ural), Ukraine (Bidzilya et al. 2011: 65).

##### Biology.

Host plant and early stages are unknown. The type series were collected by artificial light from early June to early August at grassy lowland steppes (Junnilainen and Nupponen 2010).

##### Remarks.

*Megacraspeduslongipalpella* was described from numerous males collected in the southern Urals, Russia (Junnilainen and Nupponen 2010).

#### Megacraspedus niphorrhoa

Taxon classificationAnimaliaLepidopteraGelechiidae

(Meyrick, 1926)

Trichembola niphorrhoa Meyrick, 1926: 272.

##### Examined material.

Kazakhstan. 2 ♂, 20 km E Chelkar settl., Bolshoe Barsuk sands, 185 m, 16.v.2012, leg. K. Nupponen; 1 ♂, near Zhababulak vill., Kumzhargan sands by Emba river, 17.v.2012, leg. K. Nupponen, genitalia slide 17/1490 ♂ Huemer; 8 ♂, 17 km NE Emba vill., 300 m, 18.v.2012, leg. K. Nupponen; 1 ♂, same data, but 20.v.2012; 4 ♂, 45 km NE Zhana-Uzen town, Bostankum sands, 160 m, 26.v.2011, leg. K. Nupponen; 1 ♂, Sengirkum sands, Terekurpa well, 70 m, 27.v.2011, leg. K. Nupponen (RCKN, TLMF); 1 ♂, Ryn-Kum sandy steppe, 15 km E Bisen village, 28.v.2000, leg. V. Karalius & J. Miatleuski (RCHW). Russia. 7 ♂, 1 ♀, S-Ural, Bashkiria, Sakmara River, Jantyshevo village, 20.vi.1996, leg. K. Nupponen, J.-P. Kaitila, J. Junnilainen & M. Ahola, genitalia slides 15/1399 ♂ Huemer, 17/1493 ♀ Huemer, no. 2/21X.2008 K. Nupponen, 02022602 J. Junnilainen (MZH, RCKN, ZMUC); 1 ♂ (paratype *M.litovalvellus*), Orenburg oblast, 20 km S Pokrovka village, Schibendy valley, 6.vi.1998, leg. T. & K. Nupponen; 1 ♂, same data, but 11.vi.2001, leg. K. Nupponen (all RCKN); 1 ♂, S Ural, Cheliabinsk distr., Kizilskoye village, 24.vi.2017, leg. H. Roweck & N. Savenkov; 4 ♂, same data, but 1.vii.2017 (all ECKU).

##### Redescription.

**Figures 85–90.**
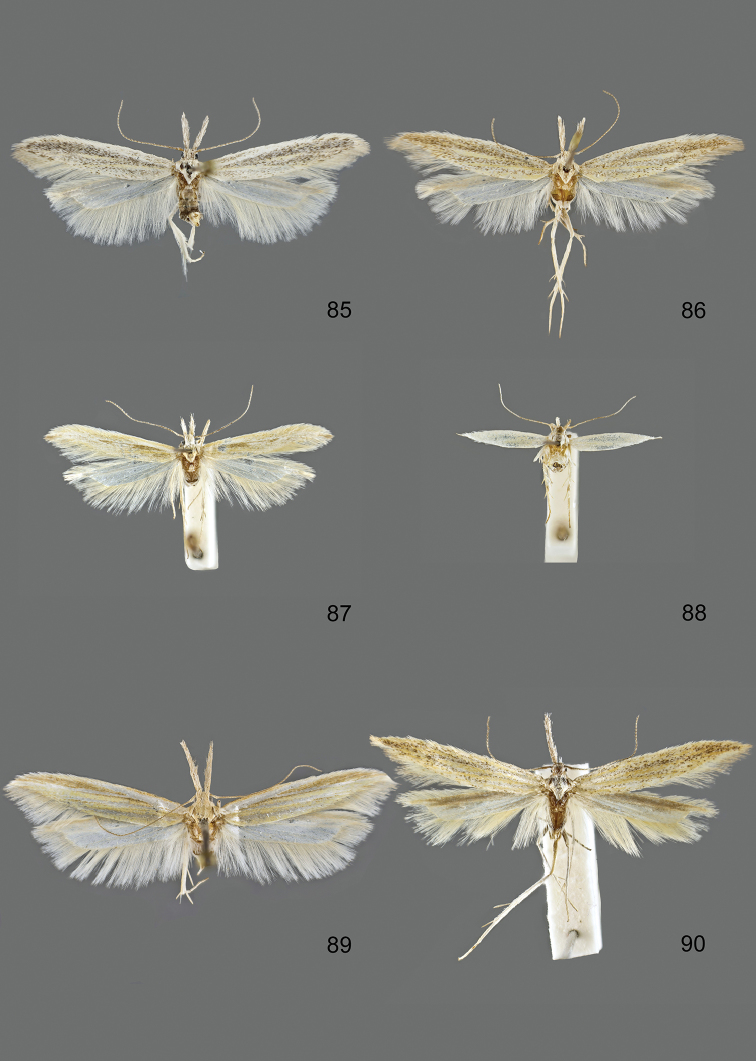
*Megacraspedus* adults in dorsal view. **85***M.niphorrhoa* (Meyrick, 1926) – male, Kazakhstan (RCKN) **86***M.niphorrhoa* (Meyrick, 1926) – female, Russia (MZH) **87***M.albella* (Amsel, 1935) – male, Iran (NHMW) **88***M.albella* (Amsel, 1935) – female, Iran (NHMW) **89***M.fallax* (Mann, 1867) – Holotype, male, Hungary (NHMW) **90***M.balneariellus* (Chrétien, 1907) – male, France (ZMUC).

Adult. *Male* (Figure 85). Wingspan 15–19 mm. Labial palpus very long, porrect, light greyish white with medial part of outer surface dark brown, inner surface greyish brown; segment 3 reduced. Antennal scape with pecten consisting of a few hairs; flagellum light greyish brown, indistinctly ringed with black. Head, thorax and tegula as forewing. Forewing whitish grey, densely mottled with dark brown, mostly in costal two-thirds (apart from costa itself); a yellow streak along dorsum, a yellow streak from base through fold to tornus, and a similar streak in middle of wing from one-fifth from base toward apex; fringes light grey. Hindwing whitish grey, darker towards costa, with white fringes.

*Female* (Figure 86). Wingspan 18 mm. Similar to male.

*Variation*. The examined specimens show only slight variation.

*Male genitalia* (Figure 215). Uncus moderately slender, sub-rectangular, approximately 1.4 times longer than wide, distally evenly rounded; gnathos hook stout, slightly longer than uncus, evenly curved to pointed apex; anterior margin of tegumen with broad and moderately shallow excavation, medially with additional small emargination, longitudinal sclerotised ridge from anterior edge to posterior third; pedunculi small, rounded; valva moderately slender, extending to about base of uncus, apex slightly swollen, weakly rounded, setose; saccular area covered with setae, without separated sacculus; posterior margin of vinculum with shallow medial emargination, with indistinct lateral humps, suboval vincular sclerite with strongly sclerotised posterior edge; saccus sub-triangular, basally broad, distally tapered to pointed apex, ratio maximum width to length about 1, posterior margin with weakly sinusoid mediolateral projections, separated by shallow emargination, medial part smooth, without sclerotised ridge, lateral sclerites long and slender, approximately 0.8 times shorter than maximum width of saccus; phallus straight, with bulbous coecum, distal two-thirds slender, rod-like sclerotisation with few minute subapical thorns.

*Female genitalia* (Figure 285). Papilla analis strongly sclerotised, moderately large, apically weakly constricted, rounded; apophysis posterior rod-like, with exteremly widened sub-posterior sixth, moderately short, approximately 2.8 mm long; segment VIII approximately 0.7 mm long, about same width, large sclerotised dorso- and ventrolateral zone, medially largely membranous; subgenital plate with sub-triangular subostial sclerotisation, distinct suboval projection anteromedially, posteriorly extended into moderately long, basally widened, distally pointed sub-medial sclerites delimiting oblong ostium bursae, anterior margin a sclerotised edge connected with apophysis anterior; apophysis anterior rod-like, almost twice length of segment VIII, posteriorly becoming band-like venula of segment VIII, distinctly widening to large sclerotised zone, extending to posterior margin; colliculum moderately long, irregularly sclerotised; ductus bursae gradually widening to weakly delimited, slender corpus bursae, entire length of ductus and corpus bursae about 3 mm; signum moderately small, sub-triangular spiny plate.

##### Diagnosis.

*Megacraspedusniphorrhoa* is characterised by its light greyish forewings mottled with dark brown scales especially in the middle and apical part of the wing, and with three longitudinal yellow streaks. It is very similar to *M.podolicus* (Figure 92). Males of these two species can be separated by brushing scales off the tip of the abdomen in order see the uncus, which is shorter and broader in *M.niphorrhoa*. The male genitalia are very similar to other species of the *M.fallax* species group, particularly *M.albovenata* (Figure 213) and *M.longipalpella* (Figure 214), and differ only in subtle characters such as the anterior edge of the saccus and the stout gnathos hook. The female genitalia are characterised by the posteriorly extremely widened apopophysis posterior. However, females of several related species remain unknown and thus the diagnostic value of this character is tentative.

##### Molecular data.

BIN BOLD:ACB3210 (n = 4). The intraspecific divergence of the barcode region is moderate with mean 0.7% and maximum divergence of 1.4%. The distance to the nearest neighbour *M.longipalpella* is 4.8% (p-dist).

##### Distribution.

Kazakhstan, Russia (S Ural).

##### Biology.

Host plant and early stages are unknown. The adults have been collected in June at low altitudes.

##### Remarks.

*Trichembolaniphorrhoa* was described from one male collected by Bartel at Lake Indersky, W. Kazakhstan (Meyrick 1926: 272).

#### Megacraspedus albella

Taxon classificationAnimaliaLepidopteraGelechiidae

(Amsel, 1935) comb. n.

Chilopselaphus albella Amsel, 1935: 302, pl. 10, fig. 57

##### Examined material.

**Lectotype** ♂, **here designated**, [Israel/Palaestine, West Bank]“Georgsklost. Wad. Kelt Lichtfang 1.4.1930“ “Palästina Expedition 18.2.-4.6.30 H. Amsel” “GU 699” “Typus leg. H. Amsel % *Chilopselaphus albella*” “coll. SMNK” “ex coll. H. G. Amsel” (SMNK). **Non-type material.** Iran. 1 ♂, 1 ♀, 40 km N of Bandar-Abbas, 7.iv.1972, genitalia slides Mus. Vind. 16.662 ♀, Mus. Vind. 16.663 ♂ (NHMW).

##### Redescription.

Adult. *Male* (Figure 87). Wingspan 15 mm. Labial palpus moderately long, porrect, cream-white, slightly mottled brown on outer surface; segment 3 reduced. Antennal scape with pecten consisting of several hairs; flagellum indistinctly ringed white and dark. Head light yellow in middle, laterally white; thorax light yellow; tegula yellowish white. Forewing yellowish, darker yellow along costa, lighter yellow in dorsal third; veins white; a white sub-costal streak from base to 2/5, a brown streak in basal third and brown patches at end of cell and in apex; fringes yellowish grey. Hindwing whitish grey with concolorous fringes.

*Female* (Figure 88). Wingspan 10 mm. Forewing apically lanceolate; costal and dorsal third whitish, medially yellow. Hindwing very short, sub-triangular, white. Otherwise similar to male.

*Variation*. Not observed because of very limited material.

*Male genit*alia (Figure 216). Uncus moderately slender, approximately 1.8 times longer than minimum width, weakly obovate with outer edges slightly diverging towards evenly rounded distal part, posterioventral part covered with strong microtrichia; gnathos hook stout, about length of uncus, evenly curved to pointed apex; anterior margin of tegumen with broad and shallow excavation, medially with additional small emargination, tegumen medially without distinct longitudinal sclerotised ridge; pedunculi small, rounded, with transverse sclerotised ridge; valva moderately slender, extending to about middle of uncus, apex weakly rounded, setose; saccular area with few short setae, without separated sacculus; posterior margin of vinculum with shallow medial emargination, indistinct lateral humps, suboval vincular sclerite with strongly sclerotised medio-posterior edges; saccus sub-triangular, basally broad, with weakly convex outer edge, distally tapered to pointed apex, short, ratio maximum width to length about 1, posterior margin with weakly sinusoid mediolateral projections, separated by shallow emargination, medial part smooth, without sclerotised ridge, lateral sclerites moderately short, approximately 0.7 times length of maximum width of saccus; phallus straight, with strongly bulbous coecum, distal two-thirds moderately slender, with dorsal and ventral sclerotised lobes, 3 minute subapical thorns, apex broadly rounded.

*Female genitalia* (Figure 286). Papilla analis long and moderately slender, apically weakly narrowing, rounded; apophysis posterior slender rod-like, about 2 mm long, with small sclerotised field posteriorly; segment VIII approximately 0.6 mm long, membranous; subgenital plate with narrow subostial sclerotisation, with shallowly rounded projection anteriorly, posteriorly extended into very long, pointed sub-medial sclerites, delimiting oblong ostium bursae, anterior margin with rod-like edge connected with apophysis anterior; apophysis anterior slender, rod-like, almost length of segment VIII, posteriorly becoming rod-like venula of segment VIII, posterior fifth distinctly widening to oblong sclerotised zone, extending to posterior margin; small colliculum present; ductus bursae gradually widening to weakly delimited suboval corpus bursae, entire length of ductus and corpus bursae approximately 2.6 mm; signum small, spiny plate.

##### Diagnosis.

*Megacraspedusalbella* is characterised by its long labial palps with reduced segment 3, by the antennal scape with pecten consisting of several hairs, and by the light yellowish forewings with brown streaks/patches in medial part and by having white veins. It resembles *M.numidellus* (Figs 79–80) but that species has many dark brown scales on the forewing. The male genitalia are very similar to *M.fallax* and differ only in subtle characters such as the shorter gnathos hook, the absence of a sclerotised teguminal ridge, and the apically less swollen valva. The female genitalia are somewhat similar to *M.gallicus* sp. n. (Figure 280) but differ in particular in the absence of a sclerotised antrum.

##### Molecular data.

Not available, no suitable specimen was available for barcoding.

##### Distribution.

Iran, Israel/Palaestine.

##### Biology.

Host plant and early stages are unknown. The few adults known to date have been collected in early April and early June at unreported altitudes.

##### Remarks.

*Chilopsephalusalbella* Amsel, 1935 was described from few specimens (“selten”) collected by HG Amsel at “Georgskloster”, now Israel, 1 April 1930. A lectotype is here designated in order to fix the identity of the species and conserve stability of nomenclature. The lectotype is rather worn and bleached. The original male genitalia slide (without phallus) has been remounted. It fully agrees with specimens from Iran and we thus consider these samples conspecific.

#### Megacraspedus fallax

Taxon classificationAnimaliaLepidopteraGelechiidae

(Mann, 1867)

Chilopselaphus fallax Mann, 1867: 850.
Trichembola neurophanes Meyrick, 1926: 271.

##### Examined material.

**Holotype** ♂, *Chilopselaphusfallax*, “Ofen 1866” “Mann 1867 Type“ “*Chilopselaphus fallax* Mn 1867 Ofen.” “BC TLMF Lep 06708” “Gen. Präp. Mus. Vind. 16.647 ♂” (NHMW). **Non-type material.** Kazakhstan. 2 ♂, Dzhambulskaya obl., 70 km NNE Frunze, 950 m, 19.vii.1990, leg. Kaila & Mikkola (MZH); 1 ♂, Kungey Alatau, Kegen Pass, N-slope, 1580 m, 11.vii.2002, leg. A. Pototski (RCAP); 5 ♂, North Kegen, vii.2002, leg. U. Jürivete (ZMUC). Russia. 1 ♂, S-Ural, Guberli, 23.vii.1892, leg. Christoph; 1 ♂, Guberli, leg. Duske, 1 ♂, S- Ural, 28.vi.1897, leg. Duske; 1 ♂, [leg. Duske] (all MZH); 1 ♂, Volgograd, 25–31.v.1967, leg. V. Zouhar (ZMUC); 1 ♂, S-Ural, Orenburg oblast, 20 km S Pokrovka village, Schibendy valley, 22.vi.1999, leg. K. & T. Nupponen (ZMUC); 7 ♂, same data, but 1.vii.2003, leg. K. Nupponen (RCKN, ZMUC); 1 ♂, S-Ural, Orenburg oblast, 40 km W Orsk, near Guberlja vill., 26.vi.2003, leg. K. Nupponen (RCKN); 1 ♂, same data, but 12–13.vii.2015, leg. H. Roweck & N. Savenkov; 1 ♂, same data, but 25–26.vi.2017 (all ECKU);7 ♂, S-Ural, Orenburg oblast, Orenburgskij zap., Burtinskil step, 12–17.vi.2007, leg. J. Kullberg & M. Zalewski (MZH); 2 ♂, S-Ural, Kidriasovo env., 300 m, 21.vi.2009, leg. J. Šumpich (TLMF, ZMUC); 4 ♂, same data, but, 6 km W Donskoje village, mount Verbljushka 24–28.vi.2009, leg. J. Šumpich (NMPC, TLMF, ZMUC); 1 ♂, same data, but, 3 km 7–8.vii.2015, leg. H. Roweck & N. Savenkov (ECKU); 1 ♂, Baskortostan, Moskovo, 6–7.vii.2013, leg. L. Srnka (RCZT); 1 ♂, S Ural, Cheliabinsk distr., Kizilskoye village, 6.vii.2015, leg. H. Roweck & N. Savenkov; 1 ♂, same data, but 1.vii.2017 (all ECKU). Turkey. 2 ♂, prov. Erzincan, 40 km SW Erzincan, Kemah, 1100 m, 2.viii.1997, leg. K. Larsen, genitalia slide 5316 Karsholt (ZMUC).

##### Redescription.

Adult. *Male* (Figure 89). Wingspan 17–22 mm. Labial palpus long, porrect, white on upper and lower surface, dark brown on inner and outer surface; segment 3 reduced. Antennal scape with pecten of 1–3 hairs; flagellum light brown, ringed with black. Head light brown; thorax and tegula yellowish brown, the latter with white tip. Forewing yellowish, overlaid with dark brown in costal third; a distinct silvery white sub-costal streak dividing into two half way to apex; two such, but more slender streaks in dorsal half of the wing edged by dark brown scales; veins in apical area silvery white; fringes light grey. Hindwing white, darker towards costa, fringes whitish grey.

*Female*. Unknown.

*Variation*. The amount of dark brown scales on the forewings vary, and in some specimens the apical area (between veins) is almost brown. Specimens tend to become greasy and the whitish parts of the head and body thereby become brown.

*Male genitalia* (Figure 217). Uncus moderately slender, approximately 1.6 times longer than wide, with parallel outer edges, evenly rounded distal part, posterioventral part covered with strong microtrichia; gnathos hook stout, approximately 1.3 times length of uncus, evenly curved to pointed apex; anterior margin of tegumen with broad and moderately shallow excavation, medially with additional small emargination, with longitudinal sclerotised ridge extending to about middle of tegumen; pedunculi small, rounded, with transverse sclerotised ridge; valva moderately slender, extending to about middle of uncus, apex weakly rounded, swollen, setose; saccular area setose, without separated sacculus; posterior margin of vinculum with shallow medial emargination, indistinct lateral humps, suboval vincular sclerite with strongly sclerotised posterior edge; saccus sub-triangular, basally broad, with weakly convex outer edge, distally tapered to pointed apex, short, ratio maximum width to length approximately 0.9, posterior margin with weakly sinusoid mediolateral projections, separated by shallow emargination, medial part smooth, without sclerotised ridge, lateral sclerites moderately long, approximately 0.8 times length of maximum width of saccus; phallus straight, with strongly bulbous coecum, distal two-thirds moderately slender, with dorsal and ventral sclerotised lobes, few minute subapical thorns, apex broadly rounded.

*Female genitalia*. Unknown.

##### Diagnosis.

*Megacraspedusfallax* is characterised by the distinct, silvery white sub-costal streak on the forewings. *M.gallicus* sp. n. (p 101). The male genitalia are very similar to *M.albella* (Figure 216) and differ only in subtle characters such as the longer gnathos hook, the presence of a sclerotised teguminal ridge, and the distinctly swollen valva apically.

##### Molecular data.

BIN BOLD:ACB0437 (n = 3). The intraspecific divergence of the barcode region is low with mean 0.1% and maximum divergence of 0.2%. The distance to the nearest neighbour *M.niphorrhoa* is 5.6% (p-dist).

##### Distribution.

Hungary, Kazahkstan, Russia (S Ural, Volgograd oblast), Turkey. According to Bidzilya (2002: 64) in Siberia (Krasnojarsk Krai). Also recorded from China (Xinjiang, Kuldja) (Rebel 1914: 277), but we have not been able to check if the material in question is correctly identified.

##### Biology.

Early stages are unknown. The larva is stated to feed in stems of *Stipa* (Piskunov 1981: 987). The adults have been collected from late May to early August at altitudes up to 1580 m.

##### Remarks.

*Chilopselaphusfallax* was described from one specimen collected by Rudolf Anker at Ofen (now part of Budapest), Hungary (Mann 1867). *Trichembolaneurophanes* was described from one male collected at Uralsk, W Kazakhstan by Bartel (Meyrick 1926). The two specimens from Turkey differ in being larger (wingspan 24 mm). Their male genitalia are similar to typical *M.fallax*, apart from having a few additional small thorns laterally on the phallus.

#### Megacraspedus balneariellus

Taxon classificationAnimaliaLepidopteraGelechiidae

(Chrétien, 1907)

Chilopselaphus balneariellus Chrétien, 1907: 179.

##### Examined material.

Croatia. 1 ♂, Konjevrate, 6.vi.2005, leg. Z. Tokár; 1 ♂, 5 km SE Pirovac, 24.vi.2006, leg. Z. Tokár (all RCZT); 3 ♂, 2 ♀, Pag, 10.vi.2015, leg. J. Junnilainen, genitalia slide GU 16/1457 Huemer (RCJJ); 1 ♂, 5 km SE Pirovac, 24.vi.2006, leg. Z. Tokár (RCZT). France. 1 ♂, Dep. Gard, Aigues-Mortes, 3.vii.1988, leg. K. Larsen (ZMUC); 1 ♂, Dep. Hérault, Séte, 4.vi.2003, leg. J. Procházka (NMPC); 10 ♂, Dep. Hérault, Marselian Plage, 13.vi.2001, leg. K. Larsen (ZMUC); 4 ♂, 2 ♀, Dep. Hérault, Frontignan, sea level, 26–29.v.2004, leg. O. Karsholt, genitalia slides 6516 Hendriksen, GU 14/1388 ♂ Huemer (ZMUC); same data, but 1 ♂, 23.vi.1999, leg. A. Cox (ZMUC); 2 ♂, same data, but 10–15.vi.2004 (ZMUC); 1 ♂, Dep. Pyrénées-Orientales, St. Cyprien, Plage, 15.vi.1992, leg. J. Nel, genitalia slide 1833 NEL (TLMF). Italy. 1 ♂, prov. Venezia, Chioggia, Bosco Nordi, 11.v.2016, leg. G. Timossi (RCGT); 3 ♀, prov. Grosseto, Marina di Albarese, Maremma, 19.vi.1981, leg. M. & E. Arenberger, genitalia slide GU 17/1476 Huemer (RCEA; ZMUC). Spain. 1 ♂, prov. Girona, Port Bou, 0–300 m, 9–24.vi.1964, leg. M. & W. Glaser (SMNK); 4 ♂, prov. Girona, Rosas, saltmarsh, 20 m, 20.vi.1964, leg. M. & W. Glaser (SMNK, ZMUC); 1 ♂, prov. Tarragona, Sierra de Roquerole, Coll de la Teixeta, 1000 m, 3.vi.2003, leg. J. Procházka, genitalia prep. (in glycerin) (NMPC).

##### Redescription.

Adult. *Male* (Figure 90). Wingspan 17–19 mm. Labial palpus very long, porrect, white mottled with brown on inner and outer surface, white on upper and lower surface; segment 3 reduced. Antennal scape with pecten consisting of 1–3 hairs; flagellum ringed light and black. Head, thorax and tegula light grey, the latter with white tips. Forewing yellow, veins looking greyish by being covered with white blackish brown tipped scales; fringes light grey. Hindwing whitish, grey towards costa, with white fringes.

**Figures 91–96.**
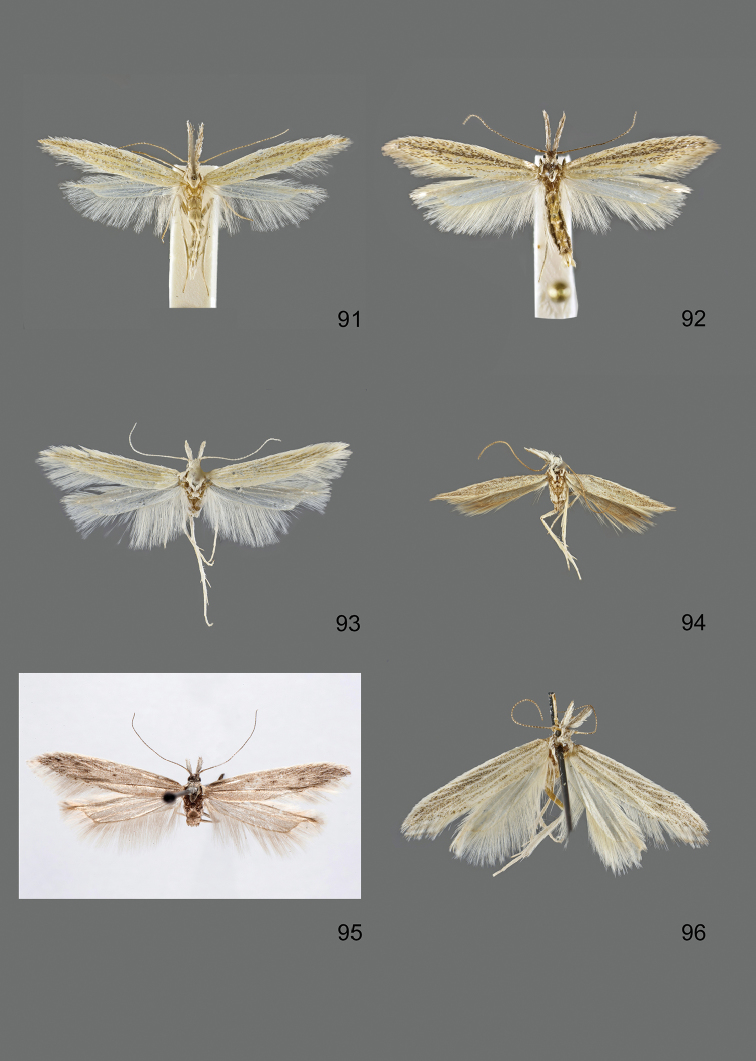
*Megacraspedus* adults in dorsal view. **91***M.balneariellus* (Chrétien, 1907) – female, Italy (RCEA) **92***M.podolicus* (Toll, 1942) – male, Hungary (RCZT) **93***M.kazakhstanicus* sp. n. – Holotype, male, Kazakhstan (RCKN) **94***M.knudlarseni* sp. n. – Holotype, male, Spain (ZMUC) **95***M.majorella* Caradja, 1920 – Paralectotype, male, Kyrgyzstan (MGAB) **96***M.latiuncus* sp. n. – Holotype, male, Kazakhstan (MZH).

*Female* (Figure 91). Wingspan 19 mm. Similar to male apart from slightly more slender and pointed wings.

*Variation*. The examined specimens exhibit only minor variation. One specimen has a wingspan of only 15 mm.

*Male genitalia* (Figs 218–219). Uncus slender, three times longer than minimum width, broadest at base, distally narrowing with outer edges almost parallel, apical edge evenly convex; gnathos hook massive, stout, slightly longer than uncus, evenly curved, distal half produced to pointed apex; anterior margin of tegumen with broad and moderately shallow excavation, medially with additional small emargination, longitudinal sclerotised ridge from anterior edge to middle of tegumen; pedunculi small, rounded, transverse sclerite; valva slender, extending slightly beyond base of uncus, basally widened, with longitudinal ridge, distal part digitate with apex slightly swollen and weakly rounded, setose; saccular area covered with setae, without separated sacculus; posterior margin of vinculum with shallow medial emargination, with weakly developed lateral humps, sub-ovate vincular sclerite with strongly sclerotised sub-posterior edge; saccus almost sub-triangular, with distinctly concave outer edge, basally broad, distally strongly tapered to pointed apex, ratio maximum width to length approximately 0.7, posterior margin arched, with weakly sinusoid mediolateral projections, separated by shallow emargination, medial part smooth, without sclerotised ridge, lateral sclerites long and slender, about length of maximum width of saccus; phallus straight, with bulbous coecum, distal two-thirds slender, sclerotised dorsal and ventral zones, subapical-ridge with few small teeth, apex rounded.

*Female genitalia* (Figure 287). Papilla analis large, approximately 0.8 mm long, 0.5 mm broad, sub-rectangular, posteriorly slightly elongated, with weakly pointed apex; apophysis posterior rod-like, short, approximately 1.7 mm long, with distinctly widened sub-posterior sixth, posterior end curved, slender; segment VIII approximately 0.6 mm long, posterior and lateral part smoothly sclerotised; subgenital plate with band-like subostial sclerotisation, with broad and shallow projection anteriorly, posteriorly extended into moderately short, pointed sub-medial sclerites, extending to about middle of segment VIII and delimiting suboval ostium bursae, anterior margin with band-like edge connected with apophysis anterior; apophysis anterior rod-like, about length of segment VIII; colliculum small; ductus bursae gradually widening to weakly delimited corpus bursae, entire length of ductus and corpus bursae approximately 3.2 mm; signum moderately large, cleft, sub-triangular plate.

##### Diagnosis.

*Megacraspedusbalneariellus* is characterised by its yellow forewings with greyish veins. It is similar to *M.podolicus* (p 119). The male genitalia are similar overall to other species of the *M.fallax* species group, but differ from all species particularly by the slender uncus. From the nearest species *M.podolicus* (Figure 220) they can furthermore be separated by the longer phallus with dentated sub-apical ridge. The female genitalia differ from other species of the *M.fallax* species group with known females in several characters, particularly the peculiar shape of the papilla analis and the apophysis posterior.

##### Molecular data.

BIN BOLD:ADB9039 (n = 2). The intraspecific divergence of the barcode region is 0%. The distance to the nearest neighbour *M.podolicus* is 6.3% (p-dist).

##### Distribution.

Croatia, France, Italy, Spain.

##### Biology.

Host plant and early stages are unknown. The adults have been collected from late May to late June in halophytic habitats (with a single specimen stated to have been collected at an altitude of 1000 m).

##### Remarks.

*Chilopselaphusbalneariellus* was described from an unstated number of specimens from France, Languedoc (Chrétien 1907: 179).

#### Megacraspedus podolicus

Taxon classificationAnimaliaLepidopteraGelechiidae

(Toll, 1942)

Chilopselaphus podolicus Toll, 1942: 170, pl. 13, figs 7–8, pl. 16, fig. 26.

##### Examined material.

Austria. 2 ♂, Burgenland, Weiden am See S, Zitzmannsdorfer Wiesen, 24.vi.1961, leg. F. Kasy (NHMW, TLMF); 1 ♂, same data, but 23.vi.1962, genitalia slide GEL 1202 Huemer. Hungary. 1 ♂, Csákberény, Bucka-hegy, 19.vi.2004, leg. Z. Tokár (RCZT); 5 ♂, Leanufalu, 5–10.vii.1997, leg. B. S. Larsen, genitalia slide 6517 Hendriksen (ZMUC); 4 ♂, 2 km N Börgönd, 24–25.vi.1998, leg. B. S. Larsen (ZMUC). Romania. 1 ♂, Dobrogea, Grindul Chituc, Vadu, 17.vi.2012, leg. S. & Z. Kovacs, genitalia slide 5323 Karsholt (RCKO). Russia. 2 ♂, S-Ural, Orenburg oblast, 8 km E Novoiletzk, 8.vi.1998, leg. T. & K. Nupponen (ZMUC); 1 ♂, Altai Republic, Aktash village, 1400 m, 11.vii.2014, leg. J. Šumpich, genitalia slide GU 16/1459 Huemer (TLMF); 5 ♂, S Ural, Cheliabinsk distr., Oktyabrskoje Selitkul reserve, 5–6.vii.2016, leg. H. Roweck & N. Savenkov (ECKU). Ukraine. 1 ♂, Borszczów district, Ścianka Hłody, 27.vi.1938, leg. S. Toll (HNHM); 1 ♂, same data, but 29.vi.1938 (ZMUC); 1 ♂, Crimea, Karadag, 26.vi.1993, leg. A. Bidzilya (ZMUC); 3 ♂, same data, but 3–9.vi.1996, leg. Yu. Budashkin (MZH).

##### Redescription.

Adult. *Male* (Figure 92). Wingspan 15–18 mm. Labial palpus very long, porrect, brown mottled with some white on inner and outer surface, greyish white on upper and lower surface; segment 3 reduced. Antennal scape with up to 5 hairs; flagellum light brown, indistinctly darker ringed. Head, thorax and tegula light grey-brown, the latter with white tips. Forewing whitish grey, densely mottled with dark brown, especially in middle of wing; a yellow streak along dorsum, a yellow streak from base through fold to tornus, a similar streak in middle of wing from one-quarter from base toward apex and a yellow sub-costal streak to middle of wing; fringes light grey. Hindwing whitish grey, darker towards costa, with white fringes.

*Female*. Unknown.

*Variation*. The colour of the forewings varies from lighter to darker, depending on the amount of dark brown scales. Light specimens have more yellow between the veins. Worn specimens are generally lighter than fresh specimens. The hairs on the antennal scape are easily lost.

*Male genitalia* (Figure 220). Uncus slender, nearly two times longer than wide, outer edges almost parallel, apical edge strongly convex; gnathos hook massive, stout, slightly longer than uncus, evenly curved, distal half projected to pointed apex; anterior margin of tegumen with broad and moderately shallow excavation, medially with additional small emargination, longitudinal sclerotised ridge from anterior edge to middle of tegumen; pedunculi small, rounded, transverse sclerite; valva slender, extending to about middle of uncus, basally widened, with longitudinal ridge, distal part digitate with apex slightly swollen and weakly rounded, setose; saccular area covered with setae, without separated sacculus; posterior margin of vinculum with shallow medial emargination, without demarcated lateral humps, sub-ovate vincular sclerite with strongly sclerotised sub-posterior edge; saccus almost sub-triangular, with distinctly concave outer edge, basally broad, distally strongly tapered to pointed apex, ratio maximum width to length approximately 0.8, posterior margin arched, with weakly sinusoid mediolateral projections, separated by shallow emargination, medial part smooth, without sclerotised ridge, lateral sclerites long and slender, slightly shorter than maximum width of saccus; phallus straight, with bulbous coecum, distal two-thirds slender, dorsomedially with two minute teeth, elongated sclerotised zones dorsally and ventrally, apex rounded.

*Female genitalia*. Unknown.

##### Diagnosis.

*Megacraspeduspodolicus* is characterised by its long labial palps, slender wings and by the dark brown, longitudinal streak in the middle of the wing, which is absent in *M.balneariellus* (Figs 90–91). Also very similar to *M.niphorrhoa* (p 112) from which it is hardly separable externally. The male genitalia are similar overall to other species of the *M.fallax* species group, but differ from most species by the comparatively slender uncus and valva. From the nearest species *M.balneariellus* (Figs 218–219) they can be separated be the shape of the uncus and the stouter phallus without dentated sub-apical ridge.

##### Molecular data.

BIN BOLD:ADB8683 (n = 1). The distance to the nearest neighbour *M.balneariellus* is 6.3% (p-dist).

##### Distribution.

Austria, Hungary, Romania, Russia (S Ural, Altai mts), Ukraine.

##### Biology.

Host plant and early stages are unknown. The adults have been collected from early June to early July at low altitudes.

##### Remarks.

*Chilopselaphuspodolicus* was described from western Ukraine, Borszczow, Hlody (Toll 1942). Kasy (1962) compared *M.podolicus* with *M.balneariellus* and figured the male genitalia, showing clear differences between these taxa, but according to the taxonomic concept at that time he concluded that the former should be regarded as a subspecies of the latter.

#### Megacraspedus kazakhstanicus sp. n.

Taxon classificationAnimaliaLepidopteraGelechiidae

http://zoobank.org/C6A7EFE4-553D-4CAA-B859-2CAE5946B50A

##### Examined material.

**Holotype** ♂, “SW-KAZAKHSTAN 43°48'45"N 53°31'29"E Sengirkum sand, 70 m Terekurpa well 27.5.2011, K. Nupponen leg.” “DNA Barcode TLMF Lep 23657” “GU 17/1496 ♂ P. Huemer” (RCKN).

##### Description.

Adult. *Male* (Figure 93). Wingspan 16 mm. Labial palpus long, about one-third length of antenna, porrect, white with medial part of outer surface dark brown; segment 3 reduced. Antennal scape with pecten consisting of 5 hairs; flagellum white. Head, thorax and tegula cream-white. Forewing light yellow mottled with brownish and black scales especially in costal and apical part; veins pure white; fringes whitish grey. Hindwing white with white fringes.

*Female*. Unknown.

*Variation*. Unknown.

*Male genitalia* (Figure 221). Uncus moderately broad, sub-rectangular, approximately 1.3 times longer than wide, apical corners distinctly rounded, apical edge weakly convex; gnathos hook massive, stout, slightly longer than uncus, medially curved at right angles, distal half projected to pointed apex; anterior margin of tegumen with broad and moderately shallow excavation, medially with additional small emargination, longitudinal sclerotised ridge from anterior edge to posterior third; pedunculi small, rounded, transverse sclerite; valva moderately slender, extending to about middle of uncus, apex slightly swollen, weakly rounded, setose; saccular area covered with setae, without separated sacculus; posterior margin of vinculum with shallow medial emargination, without demarcated lateral humps, sub-rectangular vincular sclerite with strongly sclerotised sub-posterior edge; saccus sub-triangular, with concave outer edge, basally broad, distally strongly tapered to pointed apex, ratio maximum width to length approximately 0.7, posterior margin arched, with weakly sinusoid mediolateral projections, separated by shallow emargination, medial part smooth, without sclerotised ridge, lateral sclerites long and slender, nearly same length as maximum width of saccus; phallus straight, with bulbous coecum, distal three-quarters slender, medially with group of about 8–9 small spinules, distally with rod-like sclerotisation with few minute subapical thorns, apex broadly rounded.

*Female genitalia*. Unknown.

##### Diagnosis.

*Megacraspeduskazakhstanicus* sp. n. is characterised by its light yellow, brownish, and blackish dusted forewings with pure white veins, giving it a striped look. It is very similar to *M.albovenata* (Figs 81–82), but can probably be separated by the white antenna having a pecten of five hairs on the scape. The male genitalia are similar to other species of the *M.fallax* species group, but differ in characters such as the distinctly broader and shorter uncus.

##### Molecular data.

BIN BOLD:ADJ1190 (n = 1). The distance to the nearest neighbour *M.albovenata* is 5.1% (p-dist).

##### Distribution.

South-western Kazakhstan.

##### Biology.

Host plant and early stages are unknown. The holotype was collected in late May in a sandy area at a low altitude of 70 m.

##### Etymology.

This species is named after its place of occurrence: the republic of Kazakhstan in Central Asia. The name is a masculine adjective.

#### Megacraspedus knudlarseni sp. n.

Taxon classificationAnimaliaLepidopteraGelechiidae

http://zoobank.org/C9A4B05E-07F9-4916-B046-5E88A99BDA4C

##### Examined material.

**Holotype** ♂, “SPAIN.Islas Canarias Gran Canaria. Mogán, El Barranquillo Andres, 2 km. S. 24.vii.1995 K. Larsen” “GU 16/1454 ♂ P. Huemer” (ZMUC). **Paratype.** Spain. 1 ♂, Gran Canaria, Barranquillo Andrés, 700 m, 11–24.vi.2018, leg. P. Falck, genitalia slide 2721 Falck (RCPF).

##### Description.

Adult. *Male* (Figure 94). Wingspan 13 mm. Labial palpus long, porrect, black, white on lower and upper surface; segment 3 reduced. Antennal scape with pecten of several hairs; flagellum brown, indistinctly ringed with black. Head, thorax and tegula whitish. Forewing grey-brown from brown- and black-tipped scales; costa and veins lighter; fringes light grey, whitish at base. Hindwing grey with white fringes.

*Female*. Unknown.

*Variation*. Unknown.

*Male genitalia* (Figure 222). Uncus broad, about same length as width, maximum width at base, outer margins slightly converging, apical third strongly converged to weakly pointed apex; gnathos hook stout, about one-third longer than uncus, weakly curved, with pointed apex; anterior margin of tegumen with moderately broad and deep emargination, anteromedially sclerotised ridges merged in middle of tegumen; pedunculi small, sub-triangular; valva long, moderately slender, extending to posterior third of uncus, basally broader, distally slightly constricted, apically weakly inflated, setose; saccular area covered with few setae, without separated sacculus; posterior margin of vinculum shallowly emarginated, without lateral hump, elongated sub-rectangular vincular sclerite without sclerotised posteriomedial edge; saccus about three-quarters length of valva, nearly V-shaped, apical quarter more abruptly tapered to hardly rounded apex, ratio maximum width to length approximately 0.9, posterior margin medially emarginated, without lateromedial curvature, medial part smooth, without sclerotised ridge, length of lateral sclerites approximately 0.6 times maximum width of saccus; phallus moderately slender, inflated coecum about twice width of digitate distal two-thirds, long and curved sclerotised ridge, apex rounded.

*Female genitalia*. Unknown.

##### Diagnosis.

*Megacraspedusknudlarseni* sp. n. is amongst the smallest species in the genus, with a reduced segment 3 of the labial palps, and only comparable with *M.skulei* sp. n. (Figs 142–143), from which it differs in having an antennal pecten, and by the grey-brown forewings with lighter costa and veins. The male genitalia are somewhat similar to other species of the *M.fallax* species group but differ in several characters such as the shape of the uncus, the elongated valva-vinculum complex, the vincular sclerites, the anterior margin of the saccus and other details.

##### Molecular data.

Not available, barcoding failed.

##### Distribution.

Spain (Canary Islands: Gran Canaria).

##### Biology.

Host plant and early stages are unknown. The holotype was collected in the second half of July at an unreported altitude, and the paratype was collected in June in an altitude of 700 m.

##### Etymology.

The name (a noun in the genitive case) is dedicated to Knud Larsen, Denmark, who collected the holotype of this species and other valuable material for our study.

##### Remarks.

The systematic position of *M.knudlarseni* sp. n. in the *M.fallax* species group is based only on the labial palps and the overall similarity of the male genitalia, whereas molecular data are unavailable for this species.

### *Megacraspedusmajorella* species group

The *M.majorella* species group includes two species: *M.majorella*, and *M.latiuncus* sp. n.

External morphology. Segment 2 of labial palpus with scale brush longer than segment 3; segment 3 shorter than segment 2. Antennal scape with several long hairs. Wingspan (males) 17–21 mm. Forewing grey-brown with three black spots, or yellowish grey with white veins and no black spots. Females unknown.

Genitalia morphology. Male genitalia. Uncus broadly sub-rectangular; gnathos hook stout, longer than uncus, bent at about one-quarter, apex pointed; anterior margin of tegumen curved, sclerotised ridges joined medially in anterior part; valva digitate, weakly curved, distally slightly narrowing, apex rounded; saccular area without separated sacculus; saccus sub-triangular, basally broad, distal half tapered, with pointed apex, posterior margin arched, sinusoid, without medial emargination, medial part smooth, without sclerotised ridge; phallus straight, coecum inflated, distal two-thirds slender, with sclerotised dorsal and ventral zones, medially with small sclerotised patch, ductus ejaculatorius with double-twisted interior sclerotisation.

Diagnosticremarks. The *M.majorella* species group is defined by unique structures of the male genitalia, particularly the extraordinary large, sub-square uncus, the distinct gnathos hook, the short phallus with small sclerotised patch and the double-twisted interior sclerotisation of the ductus ejaculatorius. The two species in this species group are similar in the male genitalia. However, they differ considerably in phenotypic appearance. The systematic position of the *M.majorella* species group is uncertain and tentative due to the absence of females and supportive molecular data.

#### Megacraspedus majorella

Taxon classificationAnimaliaLepidopteraGelechiidae

Caradja, 1920

Megacraspedus imparellus (? var.) majorella Caradja, 1920: 117.

##### Examined material.

**Lectotype** ♂, “Alai” “LECTOTYPE *Megacraspedus imparellus* v. *majorellus* Car. ♂ des. Dr A. Popescu – Gori” “176434” “CIS-Korea Microlep. 4217 *Megacraspedus majorella* Car. – Alai, Himalaya K. T. Park ’96” (MNGA) [photographs examined]. **Paralectotype** ♂, same data, but without genitalia slide (MNGA) [photographs examined].

##### Redescription.

Adult. *Male* (Figure 95). Wingspan 17–21 mm. Segment 2 of labial palpus with scale brush longer than segment 3, blackish brown on outer surface, whitish grey on upper surface; segment 3 shorter than segment 2, whitish. Antenna indistinctly ringed black and light grey-brown. Head, thorax and tegula as forewing. Forewing light grey-brown mottled with black, especially in apical third; black dots in fold at 2/5, at 3/5 in middle of wing and at end of cell; scattered black scales forming an interrupted line along termen; fringes whitish grey around apex, light grey towards tornus. Hindwing grey with light grey fringes.

*Female*. Unknown.

*Variation*. None from the few examined specimens.

*Male genitalia* (Figure 223). Uncus broadly sub-rectangular, slightly longer than broad, almost width of posterior edge of tegumen, apical corners weakly rounded; gnathos hook moderately stout, approximately 1.5 times length of uncus, bent at about one-quarter, distal part nearly straight, apex pointed; anterior margin of tegumen with broad and moderately shallow excavation, medially with additional small emargination, curved sclerotised ridges joined medially in anterior part; pedunculi moderately small, rounded; valva digitate, weakly curved, extending to about middle of uncus, distally slightly narrowing, apex weakly rounded; saccular area without separated sacculus; posterior margin of vinculum with shallow medial emargination; saccus sub-triangular, basally broad, distal half tapered to weakly pointed apex, posterior margin arched, sinusoid, without medial emargination, medial part smooth, without sclerotised ridge, lateral sclerites well developed; phallus straight, bulbous coecum, distal two-thirds slender, with sclerotised dorsal and ventral zones, medially with small sclerotised patch, ductus ejaculatorius with slender, double-twisted interior sclerotisation.

*Female genitalia*. Unknown.

##### Diagnosis.

*Megacraspedusmajorella* is characterised by its light greyish brown forewings with three black dots and an interrupted black line along the termen. It is similar to *M.imparellus* (Figs 228–230), which has lighter forewings and labial palps, and *M.pacificus* sp. n. (Figure 149), which has the margin of the costa white. It also resembles *M.latiuncus* sp. n. (Figure 96), which has longer labial palps and lighter forewings with white veins, but without distinct black dots. The male genitalia are similar to *M.latiuncus* sp. n. (Figure 224) but differ in the more slender uncus and gnathos hook whereas other potential diagnostic characters cannot be compared due to different preparation techniques.

##### Molecular data.

Not available, no specimen was available for barcoding.

##### Distribution.

Kyrgyzstan (Alai Mountains).

##### Biology.

Host plant and early stages are unknown. No specific collecting data of the type-series were published.

##### Remarks.

*Megacraspedusimparellus* (? var.) *majorella* was described from two males from “Alai” (=Alai Mountains in Kyrgyzstan and Tajikistan), which were stated to be larger and having the two dots in the disc more separated compared with European *M.imparellus* (Caradja 1920). A lectotype (without abdomen) was published by Popescu-Gorj (1992). Park (1996) figured the genitalia (presumably of the paralectotype), compared it with those of *M.lanceolellus* and stated that *M.majorella* represents a distinct species. The description of *M.majorella* is based on photographs of the lectotype and a paralectotype. Due to the traditionally mounted slide of the lectotype and the picture quality several potentially diagnostic characters cannot be determined with certainty.

#### Megacraspedus latiuncus sp. n.

Taxon classificationAnimaliaLepidopteraGelechiidae

http://zoobank.org/968B2B50-78C5-4EC5-86F3-E5BDFA81CD47

##### Examined material.

**Holotype**, ♂, [Kazakhstan] “USSR 43°05'N, 77°15'E Kazakhstan, Zailiskiy Alatau, Alma-Atinskij Nat. P.” “1750 m, steppe slope/Carex creek, 14.vii.1990 ad luc., L Kaila & K Mikkola leg.” “Gen. prep. nr. 5324 ♂ O. Karsholt” (MZH).

##### Description.

Adult. *Male* (Figure 96). Wingspan 18 mm. Labial palpus long, porrect, white mottled with dark brown especially on outer surface; segment 3 reduced. Antennal scape with several long hairs; flagellum blackish brown ringed with dirty white. Head, thorax and tegula light yellowish white. Forewing light yellow mottled with brown and black scales especially in costal part; veins white; fringes light grey with a whitish fringe line. Hindwing whitish grey with whitish grey fringes.

*Female*. Unknown.

*Variation*. Unknown.

*Male genitalia* (Figure 224). Uncus broadly sub-quadrate, nearly width of posterior edge of tegumen, apical corners weakly rounded; gnathos hook massive, stout, slightly longer than uncus, abruptly curved at about one-quarter, distal part nearly straight, medially weakly widened, apically pointed; anterior margin of tegumen with broad and moderately shallow excavation, medially with additional small emargination, curved sclerotised ridges joined medially in anterior part; pedunculi moderately small, rounded; valva broadly digitate, weakly curved, extending to about middle of uncus, distal sixth narrowing, apex weakly rounded, setose; saccular area covered with setae, without separated sacculus; posterior margin of vinculum with shallow medial emargination, with lateral humps, vincular sclerite oblong; saccus sub-triangular, basally broad, distally almost evenly tapered to weakly pointed apex, ratio maximum width to length approximately 0.8, posterior margin strongly arched, sinusoid, without medial emargination, medial part smooth, without sclerotised ridge, lateral sclerites approximately 0.7 times of maximum width of saccus; phallus straight, bulbous coecum, distal two-thirds slender, with sclerotised dorsal and ventral zones, medially with small sclerotised patch of differently sized minute spines, ductus ejaculatorius with slender, double-twisted interior sclerotisation.

*Female gen*italia. Unknown.

##### Diagnosis.

*Megacraspeduslatiuncus* sp. n. is characterised by its light yellow, brownish and blackish dusted forewings with white veins. It is similar to *M.longipalpella* (Figs 83–84), but has shorter labial palps and darker forewings with less contrasting light veins. It is also similar to *M.albovenata* (Figs 81–82), which has clear white veins on light brown forewings which are not darker towards the costa. See also *M.majorella* (p 124). The male genitalia are similar to *M.majorella* (Figure 223) but characterised by the large and sub-quadrate uncus, the massive gnathos hook, and by the small field of minute spines of the phallus.

##### Molecular data.

Not available, barcoding failed.

##### Distribution.

South-eastern Kazahkstan.

##### Biology.

Host plant and early stages are unknown. The holotype was collected at light in July on a steppe slope with *Carex* at an altitude of 1750 m.

##### Etymology.

The species name is a compound word derived from the Latin words *latus* (meaning wide) and *uncus*, referring to the broad uncus. The name is a noun in apposition.

### *Megacraspedustenuignathos* species group

The *M.tenuignathos* species group includes one species: *M.tenuignathos* sp. n.

External morphology. See species description.

Genitalia morphology. Male genitalia. See species description.

Diagnosticremarks. The *M.tenuignathos* species group is defined by unique structures in the male genitalia. In particular the long and slender gnathos hook, almost extending to the anterior margin of the tegumen, and the strongly arched and evenly rounded posterior margin of the saccus as well as the distinct sub-apical tooth and supplementary small spines of the phallus are diagnostic.

The systematic position of the *M.tenuignathos* species group is uncertain and tentative due to the absence of females and supporting molecular data.

#### Megacraspedus tenuignathos sp. n.

Taxon classificationAnimaliaLepidopteraGelechiidae

http://zoobank.org/80721A58-2996-43C2-B4C0-D2C61753E56D

##### Examined material.

**Holotype** ♂, “MAROKKO Imilchil Hoher Atlas 2000 m Seeufer [lake shore] TF 11.08.1998 leg. Rolf Bläsius” “DNA Barcode TLMF Lep 19881” “GU 16/1423 ♂ P. Huemer” (RCAW).

##### Description.

**Figures 97–102.**
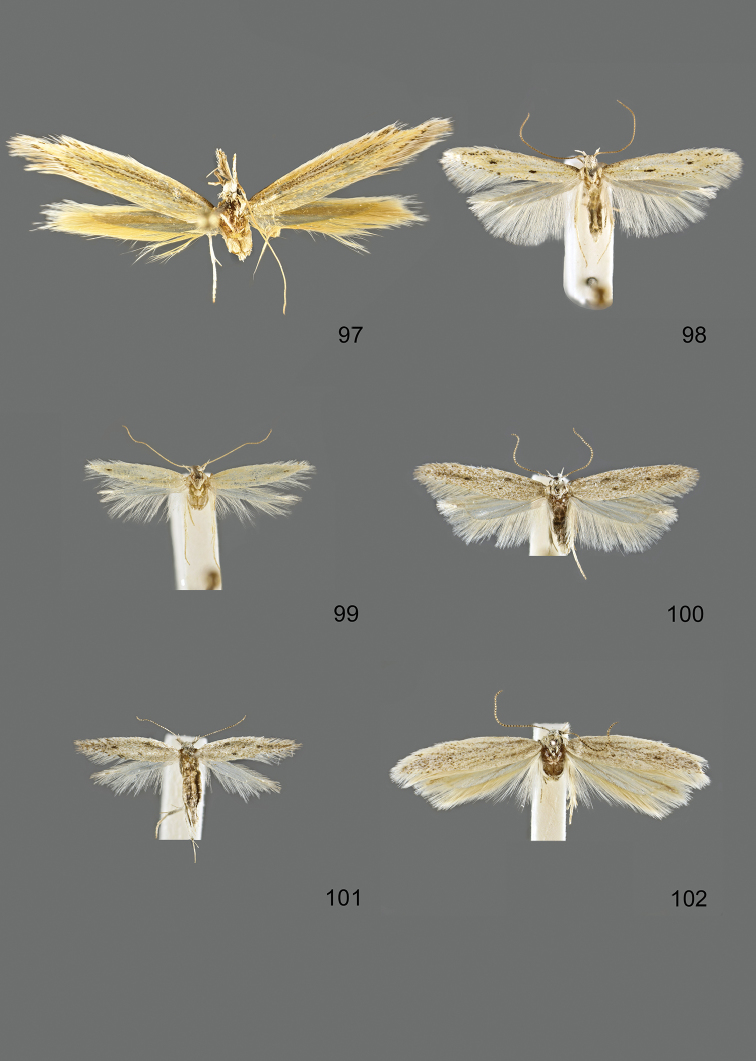
*Megacraspedus* adults in dorsal view. **97***M.tenuignathos* sp. n. – Holotype, male, Morocco (RCAW) **98***M.glaberipalpus* sp. n. – Paratype, male, Morocco (NHMW) **99***M.glaberipalpus* sp. n. – Paratype, female, Morocco (NHMW) **100***M.imparellus* (Fischer von Röslerstamm, 1843) – male, Hungary (RCZT) **101***M.imparellus* (Fischer von Röslerstamm, 1843) – female, Hungary (RCZT) **102***M.imparellus* (Fischer von Röslerstamm, 1843) – male, Greece (ZMUC).

Adult. *Male* (Figure 97). Wingspan 22 mm. Segment 2 of labial palpus with long scale brush, dark brown mottled with white; upper surface white; segment 3 long, white. Antennal scape without pecten; flagellum light brown. Head whitish; thorax and tegula as forewing. Forewing light yellow; veins white, more or less edged with black scales; costal streak white, dark yellow at base; black scales along termen; fringes alternatively light and dark. Hindwing light grey, with yellowish grey fringes.

*Female*. Unknown.

*Variation*. Unknown.

*Male genitalia* (Figure 225). Uncus broadly oblong, approximately 1.5 times longer than broad, outer margin parallel, apex strongly arched; gnathos hook long and slender, slightly longer than uncus, evenly curved, apically pointed; anterior margin of tegumen with broad and deep emargination, pedunculi moderately large with additional sclerite; valva moderately slender, distal part slightly tapered, evenly curved, extending to about base of uncus; saccular area covered with strong setae, without separated sacculus; posterior margin of vinculum with shallow medial emargination, without lateral hump, elongated suboval vincular sclerite with strongly sclerotised posteriomedial edge, laterally fused with base of valva; saccus nearly V-shaped, long, ratio maximum width to length approximately 0.6, rod-like apical quarter abruptly tapered, posterior margin broadly arched, without medial emargination, medial part with short sclerotised ridge, lateral sclerites slightly longer than maximum width of saccus; phallus with bulbous coecum, distal two-thirds stout, straight, with two long sclerotisations, distinct subapical tooth on right side and two small spines on left side.

*Female genitalia*. Unknown.

##### Diagnosis.

*Megacraspedustenuignathos* sp. n. is among the largest species in the genus without a reduced segment 3 of the labial palpus. It is moreover characterised by its light yellow forewings with white veins edged with black without black dots. The male genitalia are unmistakable due to the long and slender gnathos hook, the strongly arched posterior margion of the saccus without medial emargination, and the characteristic spines of the phallus.

##### Molecular data.

Not available, barcoding failed.

##### Distribution.

Morocco (High Atlas).

##### Biology.

Host plant and early stages are unknown. The holotype was collected in mid August at an altitude of ca. 2000 m.

##### Etymology.

The species name is a compound word derived from the Latin words *tenuis* (meaning small) and *gnathos*, referring to the slender uncus. The name is a noun in apposition.

### *Megacraspedusglaberipalpus* species group

The *M.glaberipalpus* species group includes one species: *M.glaberipalpus* sp. n.

External morphology. See species description.

Genitalia morphology. Male genitalia. See species description.

Female genitalia. See species description.

Diagnosticremarks. The *M.glaberipalpus* species group is defined by the unique structure of the labial palpus without a scale brush on segment 2. Furthermore the male genitalia exhibit some diagnostic features such as the presence of a short sacculus, the long saccus with a distinctly forked ridge and the broad and oblong sclerotisation of the ductus ejaculatiorius.

#### Megacraspedus glaberipalpus sp. n.

Taxon classificationAnimaliaLepidopteraGelechiidae

http://zoobank.org/10588381-606F-4083-8BAF-B715022D5D7C

##### Examined material.

**Holotype** ♂, “Maroc, Haut Atlas, Toubkal Massif Tamatert near Imlil above Tamatert 27.vii.2008, leg. A. Steiner, 2400 m, 31°9'N, 7°53'W A. Steiner leg.” (ZMUC). **Paratypes.** Morocco. 3 ♂, same data as holotype, but genitalia slide GEL 1245 P. Huemer (TLMF, ZMUC); 1 ♂, High Atlas, Toubkal Massif, Oukaïmeden area, piste Oukaïmeden, Tizi-n-Eddi, 2815 m, 28.vii.2008, leg. A. Steiner (ZMUC); 1 ♂, High Atlas, Oukaïmeden, 2400 m, 7–17.vi.1965, leg. Y. de Lajonquière (SMNK); 2 ♂, 1 ♀, High Atlas, 4 km E Oukaïmeden, 2200m, 10.vii.1975, leg. F. Kasy, genitalia slides Mus. Vind. 16.665 ♂, Mus. Vind. 16.666 ♀ (NHMW); 2 ♂, High Atlas, prov. Al Haouz, Imlil, 1680 m, 30.vi.2016, leg. J. Tabell, genitalia slide GEL 1252 P. Huemer (TLMF, ZMUC); 1 ♂, Middle Atlas, Ifrane, 5–10.vii.1972, leg. F. Hahn (ZSM).

##### Description.

Adult. *Male* (Figs 98). Wingspan 13–17 mm. Segment 2 of labial palpus without scale brush, cream-white, mottled with blackish brown on lower, outer and inner surface; segment 3 about same length as segment 2, white. Antennal scape without pecten; flagellum blackish brown, indistinctly lighter ringed. Head cream white; thorax and tegula as forewing. Forewing light yellowish mottled with blackish brown scales, especially at base of costa and in apical area; fold yellow with a black spot; a black spot at end of cell; fringes grey with darker fringe line. Hindwing grey with yellowish grey fringes.

*Female* (Figure 99). Wingspan 11 mm. Segment 2 of labial palpus cream-yellow, mottled with blackish brown on outer surface. Forewing yellowish with only a few black scales. Hindwing more slender than forewing. Otherwise similar to male.

*Variation*. The amount blackish brown scales on the forewing varies, giving the wing either a yellowish or light brownish appearance. There is quite a large variation in the wingspan.

*Male genitalia* (Figs 226–227). Uncus large, broad, approximately 1.7 times as long as maximum basal width, distal part slightly tapered to evenly rounded apex; gnathos hook slender, about length of uncus, slightly curved, apically pointed; anterior margin of tegumen with deep emargination; pedunculi large, suboval, with additonal ridge; valva slender, nearly straight, basally about two-thirds width of uncus base, gradually narrowing to contorted, rounded apical part, extending to about middle of uncus; sacculus short to moderately long, digitate; posterior margin of vinculum with distinct medial emargination, weakly rounded lateral humps, vincular sclerites broadly sub-rectangular, with strongly sclerotised posterior edge; saccus V-shaped, medially weakly bulged, apical third strongly tapered, ratio maximum width to length approximately 0.8, posterior margin with sinusoid mediolateral projections, separated by deep incision, medial part with short, anteriorly forked ridge, lateral sclerites slender, about length of maximum width of saccus; phallus with bulbous coecum, distal two-thirds gradually tapered, weakly S-curved, dorsal and ventral zones sclerotised, a short sclerotised ridge with longitudinal row of about four small teeth subapically on ventral surface, ductus ejaculatorius with broad and elongated sclerotisation.

*Female genitalia* (Figure 288). Papilla analis medium-sized, apically rounded; apophysis posterior slender rod-like, approximately 3.5 mm long, with short, bifurcate posterior end; segment VIII approximately 1.3 mm long, membranous; subgenital plate with sub-triangular subostial sclerotisation, posteriorly extended into long pointed sclerites, medial flaps delimiting ostium bursae, anterior margin with rod-like edge connected with apophysis anterior, medially with broad and long sinusoid projection; apophysis anterior 1.9 mm, slender, rod-like, distinctly shorter than segment VIII, posteriorly becoming rod-like venula of segment VIII, extending to posterior margin, posterior end with sclerotised broader zone; colliculum moderately long, sclerotised; ductus bursae short, moderately broad; corpus bursae suboval, weakly delimited from ductus bursae, entire length of ductus and corpus bursae approximately 2.5 mm; signum large, sub-triangular plate with strong spines.

##### Diagnosis.

*Megacraspedusglaberipalpus* sp. n. is characterised by its light yellow to brownish forewings with two black spots. It is unique in *Megacraspedus* by the absence of a scale brush at segment 2 of the labial palpi. It otherwise resembles *M.steineri* sp. n. (Figure 131), but that species has a long scale brush on segment 2 of the labial palpi and three black spots on the forewing. The male genitalia are unmistakable due to the sacculus, the shape of the saccus and the phallus. From the somewhat similar *M.gredosensis* sp. n. (Figs 175–176) they are e.g., separable by the broader uncus and the very different shape of the phallus. The female genitalia are in particular characterised by the broad posterior end of the apophysis anterior and the strong spines of the signum.

##### Molecular data.

BIN BOLD:ADF1285 (n = 1). The distance to the nearest neighbour, an unidentified Spilomelinae in BOLD, is 9.3%, the distance to the nearest congeneric species *M.cerussatellus* is 10.8% (p-dist).

##### Distribution.

Morocco (High Atlas).

##### Biology.

Host plant and early stages are unknown. The adults have been collected from the first half of June until late July at altitudes from ca. 1700 m to ca. 2800 m.

##### Etymology.

The species name is derived from combining of the Latin words *glaber* (= hairless or smooth) and *palpus*, referring to segment 2 of the labial palpi being without a scale brush. The name is a noun in apposition.

### *Megacraspedusimparellus* species group

The *M.imparellus* species group includes one species: *M.imparellus*.

External morphology. See species description.

Genitalia morphology. Male genitalia. See species description.

Female genitalia. See species description.

Diagnosticremarks. The *M.imparellus* species group is defined by diagnostic structures of the male genitalia such as the rounded shape of the uncus, the long and evenly curved gnathos hook, the presence of a short, digitate sacculus and in particular the very long and multiple contorted interior sclerotisation of the ductus ejaculatiorius. The female genitalia are characterised by the granulate microsculpture of segment VIII, and the ductus bursae and ductus seminalis, but females are unknown in several species groups and therefore this character may not be of diagnostic value.

#### Megacraspedus imparellus

Taxon classificationAnimaliaLepidopteraGelechiidae

(Fischer von Röslerstamm, 1843)

Ypsolophus imparellus Fischer von Röslerstamm, 1843: 300, 303, pl. 100, figs 2a–d [on plate as ‘*imparella*’].
Megacraspedus litovalvellus Junnilainen, *in*Junnilainen and Nupponen 2010: 10, figs. 16–17, 24, 30–31, syn. n.

##### Examined material.

**Paratypes** of *M.litovalvellus*: Russia. 2 ♂, S-Ural, Orenburg oblast, 20 km S Pokrovka village, Schibendy valley, 6–7.vi.1998, leg. K. & T. Nupponen (ZMUC); 4 ♂, same data, but 28.v.2004, leg. K. Nupponen (ZMUC); 1 ♂, same data, but 22.vi.1999, leg. T. & K. Nupponen; 1 ♂, same data, but 10.vi.2001, leg. K. Nupponen; 2 ♂, same data, but 28.v.2004 (RCKN). **Non-type material.** Austria. 1 ♂, Kärnten, Fraunstein, 9.v.1960, leg. K. Burmann (ZMUC); 7 ♂, Niederösterreich, Gramatneusiedl, Fürbachwiesen, 30.v.1979, leg. F. Kasy, genitalia slide GEL 1195 (NHMW, TLMF, ZMUC); 1 ♂, same data, but 27.v.1981 (ZMUC); 1 ♂, 1 ♀, Niederösterreich, Gramatneusiedl, 11.vi.1982, leg. E. Arenberger, genitalia slide GU 17/1479 ♀ Huemer (RCEA). Bulgaria. 1 ♂, 5 km N Sandanski, 270 m, 30.iv.2011, leg. Z. Tokár; 1 ♂, same data, but 5.v.2011, leg. Z. Tokár, genitalia prep. 12097 Z. Tokár (in glycerin) (all RCZT). Greece. 1 ♂, Thessalia, Olympos S, 30 km NW Karia, 1700 m, 10.vii.1990, leg. M. Fibiger; 1 ♂, same data, but 1850 m, 30.vii.1990 (all ZMUC); 1 ♂, 15 km W Kozani, Xerolimni, 21–23.v.2003, leg. J. Junnilainen, genitalia slide GU 16/1456 Huemer (RCJJ). Hungary. 1 ♂, Csákberény, Bucka-Hegy, 6.vi.1997, leg. Z. Tokár (ZMUC); 1 ♂, same data, but 21.v.2005, leg. Z. Tokár (RCZT); 1 ♀, same data, but 26.v.2011, leg. Z. Tokár (RCZT); 1 ♂, Mór-Gánt, 7.v.1995, leg. J. Liška (NMPC). Romania. 1 ♂, Muntii Apuseni, Cheile Turzii, 11.v.1991, leg. S. & Z. Kovács (RCKO). Slovakia. 1 ♀, Devínska Kobyla, 8.v.1992, leg. G. Pastoralis (ZMUC). Russia. 1 ♂, S Ural, Orenburg distr., Orsk, Guberlia village, 12–13.vii.2015, leg. H. Roweck & N. Savenkov;1 ♂, S Ural, Orenburg distr., Akbulak, Pokrovka village, 28–30.vi.2017, leg. H. Roweck & N. Savenkov (all ECKU); 1 ♂, Caucasus, Kabardino-Balkaria, Bezengi vill., 3.vi.1997, leg. I. & O. Kostjuk, genitalia prep. (in glycerin) (ZMKU).

##### Redescription.

Adult. *Male* (Figs 100, 102). Wingspan 12–16 mm. Segment 2 of labial palpus with long scale brush, light brown on outer, white mottled with brown on inner surface, white on lower and upper surface; segment 3 white with black tip. Antennal scape with pecten of 1–3 hairs; flagellum ringed black and light grey-brown. Head light grey-brown; thorax and tegula as forewing. Forewing bone white mottled with lighter and darker brown, especially in outer part of wing; black dots in fold at 2/5, and at 3/5 in middle of wing and at end of cell; an indistinct dark sub-costal dot at 1/3; scattered black scales forming an interrupted line along termen; fringes light grey. Hindwing light grey with concolorous fringes.

*Female* (Figure 101). Wingspan 10–12 mm. Antenna ringed black and white. Forewing slightly ellipsoid. Hindwing reduced in width and with an extended apex. Otherwise similar to male.

*Variation*.There is some variation in the amount of dark brown scales on the forewings. In some specimens they form a dark apical streak. Freshly emerged specimens are more greyish compared with specimens in collections. Specimens from Greece (Olympos) are slightly larger (wingspan 14–16 mm), slightly more broad-winged and with fewer dark scales on the forewing (thereby resembling *M.leuca*) but the reduction of dark scales may be due to worn specimens.

*Male genitalia* (Figs 228–230). Uncus almost evenly rounded, about same length as width; gnathos hook moderately slender, gradually narrowing to apical point, approximately 1.5 times length of uncus, evenly curved from base to apex; tegumen with broad and shallow excavation of anterior margin, anteromedially small additional emargination; pedunculi small, suboval; valva extending slightly beyond tip of uncus, basally weakly inflated, digitate distal part weakly curved, tapered to slightly pointed apex; saccular area distinctly digitate, basally fused with valva, distal part separated; posterior margin of vinculum with shallow emargination, with weakly curved lateral hump, vincular sclerite sub-triangular; saccus broadly V-shaped, slightly shorter than valva, posterior margin weakly emarginated, with indistinctly sinusoid mediolateral humps, medial part with sclerotised ridge from posterior margin to middle of saccus, lateral sclerites about half length of maximum width of saccus; phallus with bulbous coecum, distal two-thirds moderately slender, with long dorsal sclerotisation, ductus ejaculatorius with long, 4–5× contorted interior sclerotisation.

*Female genitalia* (Figure 289). Papilla analis medium-sized, apically rounded; apophysis posterior slender rod-like, approximately 2.3 mm long, weakly bent and widened at posterior third, with short, bifurcate posterior end; segment VIII approximately 0.5 mm long, lateroposterior part smoothly sclerotised, membranous ventromedial part with granulate microsculpture; subgenital plate with sub-triangular subostial sclerotisation, posteriorly extended into moderately short pointed sclerites, delimiting ostium bursae, anterior margin with rod-like edge connected with apophysis anterior, medially with nearly tubular projection; apophysis anterior approximately 0.9 mm, slender, rod-like, longer than segment VIII, posteriorly becoming small sclerotised zone weakly extended into segment VIII; colliculum short; ductus bursae short, broad, posterior part with granulate microsculpture, ductus seminalis originating near colliculum, broad, with granulate microsculpture; corpus bursae elongated suboval, weakly delimited from ductus bursae, entire length of ductus and corpus bursae approximately 2.2 mm; signum modertately small, transverse, spiny plate.

##### Diagnosis.

*Megacraspedusimparellus* is characterised by its light greyish brown forewings with three distinct black dots and an interrupted black line along termen. It normally has only a single pecten on the antennal scape, whereas the similar looking *M.leuca* (Figs 111–112) has several hairs on the antennal scape. It is furthermore similar to *M.pacificus* sp. n. (p 187) and *M.majorella* (p 124). The male genitalia are unmistakable due to the shape of the uncus, the gnathos hook, the saccular area and in particular the interior sclerotisation of the ductus ejaculatorius. The female genitalia differ from other species of *Megacraspedus* in particular in the granulate microsculpture of segment VIII, ductus bursae and ductus seminalis.

##### Molecular data.

BIN BOLD:ABW9450 (n = 2), BOLD:ADB7271 (n = 1), BOLD:ACB3182 (n = 1). Genetically variable species. The intraspecific divergence of the barcode region is large and reflected by 3 BINs with maximum divergence of 4.6% within all clusters. The minimum distance to the nearest neighbour *M.attritellus* is 9.2% (p-dist).

##### Distribution.

South-eastern Europe, with confirmed records from Austria, Bulgaria, Greece, Hungary, Russia (S. Ural, Caucasus), Romania, and Slovakia. A record from France (Varenne and Nel 2014: 61) refers to *M.quadristictus*. For records from Germany see under Remarks. Records from Mongolia (Piskunov 1979: 401–402) refer to *M.leuca*. Recorded from Italy (Toscana) by Mariani (1943: 174).

##### Biology.

Host plant and early stages are unknown. The type series of *M.litovalvellus* was collected at artificial light at night and by sweeping before sunset, from late May to early August on chalk steppe (Junnilainen and Nupponen 2010). Other material was collected from late April to the first half of July, mostly in lowland localities, but in Greece at altitudes up to 1850 m.

##### Remarks.

*Ypsolophusimparellus* was described from two males and two females collected in June at Baden near Vienna, Austria (Fischer von Röslerstamm 1843). The excellent figures in the original description leave no doubt to the identity of this species. *Megacraspeduslitovalvellus* was described from numerous specimens of both sexes collected in thesouthern Urals, Russia (Junnilainen and Nupponen 2010). We examined several specimens and could not confirm the alleged diagnostic differences from *M.imparellus* stated in the original description. Thus *M . litovalvellus* is formally synonymised with *M.imparellus* (syn. n.). The intraspecific barcode divergence observed in this species is not supported by morphology.

### *Megacraspedusconsortiella* species group

The *M.consortiella* species group includes eight species: *M.multispinella*, *M.nupponeni* sp. n., *M.cerussatellus*, *M.attritellus*, *M.consortiella*, *M.pototskii* sp. n., *M.leuca*, and *M.orenburgensis*.

External morphology. Segment 2 of labial palpus with scale brush longer than segment 3; segment 3 half as long to same length as segment 2. Antennal scape with pecten consisting of one to 10 hairs. Wingspan (males) 10–18 mm. Forewing with 2–3 black spots, in some species with longitudinal white or grey stripes. Known females vary from fully winged to brachypterous.

Genitalia morphology. Male genitalia. Uncus moderately large, sub-square to sub-rectangular; gnathos hook slender, curved to strongly bent in basal half; valva stout, digitate, without separate sacculus; saccus massive, U- to V-shaped, with longitudinal sclerotised ridge from posterior margin almost to apex, lateral sclerites of saccus apically often strongly bulged; phallus with distinctly inflated coecum, distal part long and slender, variably dentated, ductus ejaculatorius without specialised sclerotisation.

Female genitalia. Papilla analis medium-sized to large, apically rounded, predominantly membranous; apophysis posterior slender rod-like, short to moderate length, with small sclerotised posterior zone; segment VIII short, large sclerotised dorso- and ventrolateral zone, medially largely membranous, with or without microtrichia; subgenital plate with sub-triangular subostial sclerotisation, posteriorly extended into shortly pointed sub-medial sclerites, or subostial region membranous; apophysis anterior moderately stout, about length of segment VIII, posteriorly broadly connected with segment VIII by sclerotised band; colliculum short, sclerotised; signum small, weak spiny plate, or reduced.

Diagnosticremarks. The *M.consortiella* species group is defined by diagnostic structures of the male genitalia, in particular the moderately large uncus, the long and slender, postbasally bent gnathos hook, and the large, oblong saccus with a long medial ridge. The female genitalia are similar overall to other species of *Megacraspedus* and mainly characterised by combined structures such the short apophysis posterior and anterior, the short, pointed sub-medial sclerites or membranous subostial area, and the broadly band-like connection of apophysis anterior with segment VIII. However, females are unknown in several species groups and therefore these characters may not be of diagnostic value.

#### Megacraspedus multispinella

Taxon classificationAnimaliaLepidopteraGelechiidae

Junnilainen & Nupponen, 2010

Megacraspedus multispinella Junnilainen& Nupponen, 2010: 11, figs 19, 26.

##### Examined material.

**Holotype** ♂, “RUSSIA S-Ural Bashkiria Sakmara river Jantyshevo village 20.-21.6.1996 K. Nupponen, J.-P. Kaitila, J. Junnilainn & M. Ahola” “Prep. no. 03022406 ♂ Det. J. Junnilainen” “*Megacraspedus aholai* sp. n. ♂ Det. J. Junnilainen” “HOLOTYPE *Megacraspedus aholai* Junnilainen” “DNA sample KN00022 Lepid. Phyl.” (RCKN). **Paratype.** Russia. ♂, S-Ural, Cheliabinsk distr., near Moskovo vill., 18.vi.1998, leg. T. & K. Nupponen (RCKN). **Non-type material.** Russia. 19 ♂, 6 ♀, Tuva rep., 52°04'N, 94°22'E, Ust-Uljuk, steppe hills, 670 m, 3–5.vi.1995, leg. J. Jalava & J. Kullberg, genitalia slides GU 16/1418 ♂ Huemer, GU 16/1419 ♀ Huemer, genitalia prep. (in glycerin) (MZH); 1 ♀, Tuva rep., 51°43'N, 94°27'E, Kyzyl, Nanophyton steppe, 700 m, 5–6.vi.1995, leg. J. Jalava & J. Kullberg (MZH).

##### Redescription.

**Figures 103–108.**
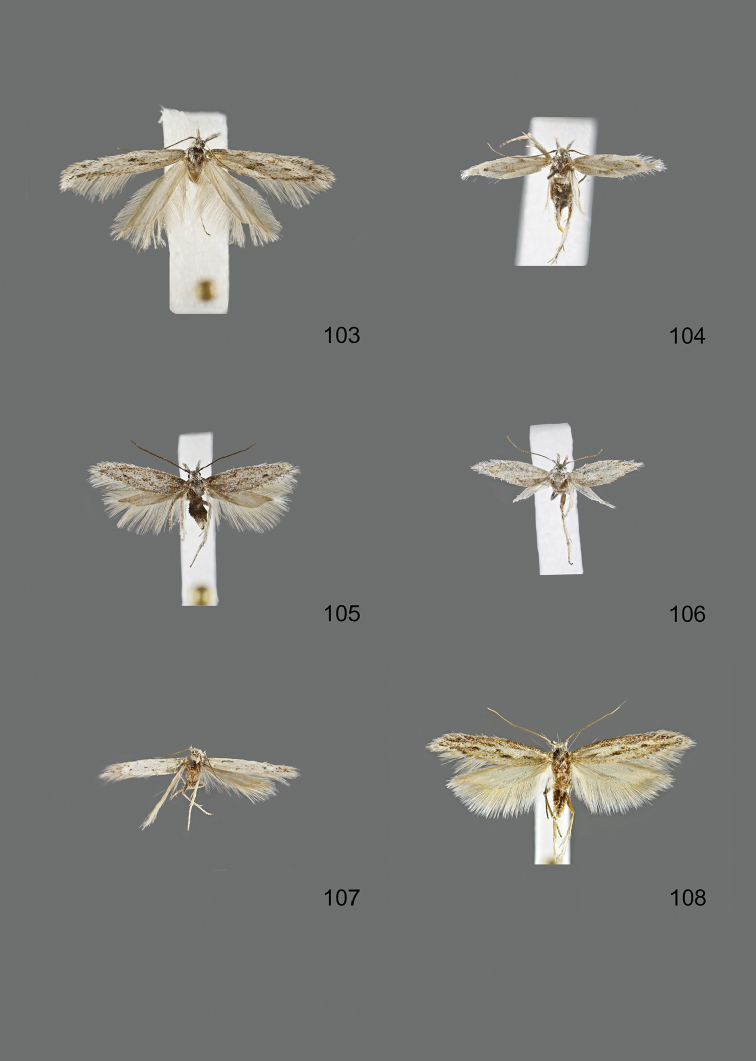
*Megacraspedus* adults in dorsal view. **103***M.multispinella* Junnilainen & Nupponen, 2010 – male, Russia (MZH) **104***M.multispinella* Junnilainen & Nupponen, 2010 – female, Russia (MZH) **105***M.nupponeni* sp. n. – Paratype, male, Russia (RCKN) **106***M.nupponeni* sp. n. – Paratype, female, Russia (RCKN) **107***M.cerussatellus* Rebel, 1930 – Lectotype, male, Bulgaria (NHMW) **108***M.attritellus* Staudinger, 1871 – male, Russia (NMPC).

Adult. *Male* (Figure 103). Wingspan 14–15 mm. Segment 2 of labial palpus with sub-rectangular scale brush longer than segment 3, blackish, white on upper surface; segment 3 half as long as segment 2, white mottled with black. Antennal scape with pecten of several hairs; flagellum light brown, indistinctly ringed with black. Head, thorax and tegula whitish grey mottled with blackish brown. Forewing whitish grey mottled with brown- or blackish-tipped scales, especially along apical margins; black spots in fold and at 2/3 and 3/4 in middle of wing; fringes whitish grey with two grey fringe lines. Hindwing light grey with concolorous fringes.

*Female* (Figure 104). Wingspan 10–11 mm. Labial palpus whitish mottled with some blackish brown on lower, outer and inner surfaces. Antenna with flagellum ringed white and light brown. Head, thorax, and tegula cream-white. Forewing ellipsoid with pointed apex, whitish mottled with some darker scales especially along apical margins; fold light yellowish; a black spot at end of cell. Hindwing nearly lanceolate. Fringes on both wings reduced.

*Variation*. There is a slight variation in the intensity of dark scales on the forewing.

*Male genitalia* (Figure 231). Uncus large, sub-square, apical edge evenly rounded; gnathos hook evenly slender, apically pointed, almost twice length of uncus, bent at right angles at about one-quarter; anterior margin of tegumen with broad and shallow U-shaped emargination, anteromedially small additional emargination; pedunculi moderately small, suboval; valva stout, extending slightly beyond base of uncus, basally weakly inflated, broadly digitate distal part weakly tapered to slightly pointed apex; saccular area densely covered with setae, without separated sacculus; posterior margin of vinculum with U-shaped medial emargination, without distinct lateral humps, vincular sclerite elongated, digitate, with sclerotised posterior edge; saccus large, slightly shorter than valva, basally broad, distally evenly tapered with broadly rounded apex, ratio maximum width to length about 1, posterior margin with rounded shallow projections, separated by small incision, medial part with long sclerotised ridge from posterior margin to subapical area of saccus, lateral sclerites almost length of maximum width of saccus, with massively bulged apex; phallus with distinctly inflated coecum, about two times wider than distal part, distal three-quarters digitate, slightly swollen subapically, with tapered apex, medial part with numerous small teeth.

*Female genitalia* (Figure 290). Papilla analis weakly sclerotised, large, apically rounded; apophysis posterior slender rod-like, short, approximately 1.1 mm long, posterior end with small sclerotised zone; segment VIII short, approximately 0.4 mm long, large sclerotised dorso- and ventrolateral zone, medially largely membranous, covered with microtrichia; subgenital plate with sub-triangular subostial sclerotisation, posteriorly extended into very short, distally pointed sub-medial sclerites, anteriorly with rounded sclerotised and projected ring delimiting ostium bursae, anterior margin with broadly rod-like edge connected with apophysis anterior; apophysis anterior moderately stout, rod-like, about length of segment VIII, posteriorly broadly connected with segment VIII by sclerotised band; colliculum short, sclerotised; ductus bursae gradually widening to weakly delimited, slender corpus bursae, entire length of ductus and corpus bursae approximately 2.5 mm; signum moderately small, oblong and weakly spiny plate.

##### Diagnosis.

*Megacraspedusmultispinella* is characterised by the short segment 3 of the labial palpus, by the antennal scape with pecten having several hairs, and by the whitish grey forewings mottled with brownish- or blackish-tipped scales along apical margins. It resembles *M.leuca* (Figs 111–112), but differs in the more greyish forewings, and by the distinct blackish scaling along the margins in the apical part of the wing. The male genitalia differ from related species of the *M.consortiella* species group in particular in the numerous teeth-like sclerites of the phallus. The female genitalia are characterised in particular by the short segment VIII as well as the short apophysis posterior and anterior. The latter character is the major diagnostic feature for separating *M.multispinella* from *M.nupponeni* sp. n. (Figure 232).

##### Molecular data.

BIN BOLD:ACM0852 (n = 1) The distance to the nearest neighbour *M.nupponeni* sp. n. is 8.1% (p-dist).

##### Distribution.

Russia (S. Ural, Tuva rep.).

##### Biology.

Host plant and early stages are unknown. The adults have been collected in early June at altitudes of ca. 700 m.

##### Remarks.

*Megacraspedusmultispinella* was described from two males collected in the southern Ural Mountains, Russia (Junnilainen and Nupponen 2010). We have been able to examine several females, all of which are more or less worn. The holotype of *M.multispinella* has a label “HOLOTYPE *Megacraspedus aholai* Junnilainen”, which is a nomen nudum.

#### Megacraspedus nupponeni sp. n.

Taxon classificationAnimaliaLepidopteraGelechiidae

http://zoobank.org/DF1BF2E9-45FA-4094-9825-68530D0B741A

##### Examined material.

**Holotype** ♂, “RUSSIA S-Buryatia 51°11–13'N 106°10–12'E 700 m, Hamar Daban mnts Murtoy river, Gusinoe Ozero vill. 6 km NW, forest steppe 27.5.2006 K. Nupponen leg.” “prep. No. 8/8.X.2006 K. Nupponen” “DNA sample KN00089 Lepid. Phyl.” (RCKN). **Paratypes.** Russia. 1 ♀, same data as holotype but genitalia slide 4/16.7.2017 K. Nupponen; 1 ♂, Buryatia rep., Chikoy valley, 10 km Novoselengnisk vill., 550–600 m, sand dunes/sandy steppe, 2.vi.2006, leg. K. Nupponen (RCKN).

##### Description.

Adult. *Male* (Figure 105). Wingspan 10.5 mm. Segment 2 of labial palpus with scale brush as long as segment 3, black mottled with white, especially on inner and upper surface; segment 3 almost as long as segment 2, white. Antennal scape with pecten of several hairs; flagellum blackish brown. Head, thorax and tegula as forewing. Forewing rather broad, white, mottled with black-tipped scales especially along margins; base black; a black dot at end of fold, one sub-costal at one-quarter, one in middle of wing and one before apex; black-tipped scales along termen; fringes white at base, light grey beyond black fringe line. Hindwing grey, with light grey fringes.

*Female* (Figure 106). Wingspan 8 mm. Antenna ringed blackish brown and whitish grey. Forewing short and broad (only 3.5 times as long as broad). Hindwing about one-third as broad as forewing, apex tapered. Otherwise similar to male.

*Variation*. Unknown.

*Male genitalia* (Figure 232). Uncus large, suboval, basal width about entire length, apical edge rounded with weak medial emargination; gnathos hook stout, evenly curved, apically pointed, slightly longer than uncus; anterior margin of tegumen with broad and shallow U-shaped emargination, anteromedially small additional emargination; pedunculi moderately small, suboval; valva stout, extending to about middle of uncus, basally weakly inflated, broadly digitate distal part weakly tapered to slightly pointed apex; saccular area densely covered with setae, longitudinal ridge present, without separated sacculus; posterior margin of vinculum with U-shaped medial emargination, without distinct lateral humps, vincular sclerite broadly elongated, with sclerotised proximo-posterior edge; saccus large, slightly shorter than valva, almost V-shaped, apex rounded, ratio maximum width to length approximately 0.7, posterior margin strongly bulged with rounded shallow projections, separated by small incision, medial part with long sclerotised ridge from posterior margin to subapical area of saccus, lateral sclerites slightly shorter than maximum width of saccus, with massively bulged apex; phallus with distinctly inflated coecum, about two times wider than distal part, distal two-thirds digitate, medial part with two groups of 2–3 small spinules.

*Female genitalia* (Figure 291). Papilla analis weakly sclerotised, large, apically rounded; apophysis posterior slender rod-like, short, approximately 1.3 mm long, posterior end with small sclerotised zone; segment VIII short, approximately 0.4 mm long, large sclerotised dorso- and ventrolateral zone, medially largely membranous, covered with microtrichia; subgenital plate with sub-triangular subostial sclerotisation, posteriorly extended into very short, distally pointed sub-medial sclerites, anteriorly with rounded sclerotised and projected ring delimiting ostium bursae, anterior margin with broadly rod-like edge connected with apophysis anterior; apophysis anterior moderately stout, rod-like, almost twice length of segment VIII, posteriorly broadly connected with segment VIII by sclerotised band; colliculum short, weakly sclerotised; ductus bursae slender, widening to weakly delimited, suboval corpus bursae, entire length of ductus and corpus bursae approximately 1.8 mm; signum moderately small, oblong and weakly spiny plate.

##### Diagnosis.

*Megacraspedusnupponeni* sp. n. is characterised by its small size, its relatively short and broad forewings, the antennal scape with several hairs, and by its clear white forewings with scattered black scales and dots. It resembles some forms of *M.attritellus* (Figure 108), but that species has more slender forewings with a whitish longitudinal streak and a shorter segment 3 of the labial palps. *Megacraspedusnupponeni* sp. n. diffes from other species of the *M.consortiella*-group in several characters of the male genitalia, particularly the short and stout gnathos hook, the almost V-shaped saccus, and the characteristic spinules of the phallus. The female genitalia are similar in particular to *M.multispinella* sp. n. (Figure 290) from which they differ e.g., in the much longer apophysis anterior.

##### Molecular data.

BIN BOLD:ACB0748 (n = 1). The distance to the nearest neighbour *M.multispinella* sp. n. is 8.1% (p-dist).

##### Distribution.

Russia (Buryatia rep.).

##### Biology.

Host plant and early stages are unknown. The adults have been collected from late May to early June in forest steppe and sandy habitats at altitudes from ca. 550 to 700 m.

##### Etymology.

The species name (a noun in the genitive case) is dedicated to Kari Nupponen, Finland, who collected the type series of this species and significantly contributed to our work with extremely valuable material from Russia.

#### Megacraspedus cerussatellus

Taxon classificationAnimaliaLepidopteraGelechiidae

Rebel, 1930

Megacraspedus cerussatellus Rebel, 1930: (14).

##### Examined material.

**Lectotype** ♂, **here designated**, [Bulgaria] “Alibotusch [mountains] 1700 m 14.VII.29” “*Megacraspedus cerussatellus* Rbl Type ♂” “BC TLMF Lep 06702” “Mus.Vind Gen.Präp. 16.648 ♂” “BC TLMF Lep 06703” (NHMW).

##### Redescription.

*Adult*. Male (Figure 107). Wingspan 11 mm. Segment 2 of labial palpus with moderately long scale brush, brown on outer and lower surface, white mottled with brown on inner surface, white on upper surface; segment 3 about half as long as segment 2, whitish brown. Antennal scape white, with pecten of several hairs; flagellum brown. Head and thorax white mottled with light brown in middle; tegula white. Forewing clear white, mottled with scattered black-tipped scales, especially in apical part; a blackish brown dot at end of fold; one sub-costal at 1/4, one in middle of wing and one before apex; black-tipped scales along termen; fringes white. Hindwing light grey, with white fringes.

*Female*. Unknown.

*Variation*. Only one specimen was examined.

*Male genitalia* (Figure 233). Uncus large, sub-square, apically weakly convex; gnathos hook slender, distinctly longer than uncus, strongly bent at about one-quarter, distal part straight and weakly diverged, apically pointed; anterior margin of tegumen with shallow, rounded emargination; pedunculi of moderate size; valva moderately stout, distally weakly curved, extending to about middle of uncus; saccular area densely covered with setae, without separated sacculus; posterior margin of vinculum with shallow medial emargination, without distinct lateral humps, vincular sclerite elongated sub-triangular, with sclerotised edges; saccus prominent, weakly U-shaped, with rounded apex, ratio maximum width to length approximately 0.75, posterior margin with broadly rounded mediolateral projections, separated by shallow incision, medial part with long sclerotised ridge from posterior margin almost to apex of saccus, lateral sclerites nearly length of maximum width of saccus, with distinctly bulged apex; phallus with large globular coecum, distal three-fifths slender, straight, dorsal part with weakly bulged sclerotisation sub-apically.

*Female genitalia*. Unknown.

##### Diagnosis.

*Megacraspeduscerussatellus* is characterised by its short segment 3 of the labial palpus, the antennal scape with several hairs, and by its pure white forewings with scattered black scales and dots. It is most similar to *M.multipunctellus* sp. n. (p 167). The male genitalia are very similar to *M.attritellus* (Figure 234) from which they differ in the slightly longer and distally broader gnathos hook and in particular the large globular coecum of the phallus, with a shorter and more slender distal part lacking distinct sclerotisation. They differ from *M.consortiella* (Figure 235) e.g., by the sub-square shape of the uncus and are easily distinguished from *M.leuca* by the distinctly smaller and V-shaped saccus.

##### Molecular data.

BIN BOLD:ACA8764 (n = 1). The distance to the nearest neighbour *M.attritellus* is 7.6% (p-dist).

##### Distribution.

Southern Bulgaria.

##### Biology.

Host plant and early stages are unknown. The type-series was collected in late July at an altitude between 1600 m and 1700 m.

##### Remarks.

*Megacraspeduscerussatellus* was described from several, mostly defective, males in poor condition, collected by A. K. Drenowski in the Alibotusch Mountains, S Bulgaria on 24.vii.1929 (Rebel 1930). A lectotype is here designated in order to fix the identity of the species and conserve stability of nomenclature.

#### Megacraspedus attritellus

Taxon classificationAnimaliaLepidopteraGelechiidae

Staudinger, 1871

Megacraspedus attritellus Staudinger, 1871: 316.

##### Examined material.

**Lectotype** ♂, **here designated**, [Russia, Volgograd oblast] “Lecto-type” “Sarepta [Krasnoarmeysk] Chr.[istoph]” “Origin.” “Lectotype ♂ *Megacraspedus attritellus* Stdgr teste K. Sattler, 1986” “ex coll. Staudinger” “GU 16/1426 ♂ P.Huemer” (ZMHU). **Paralectotypes**, 3 ♂, “Para-lecto-type” “Origin.” “ex coll. Staudinger”, genitalia slide 03022304 J. Junnilainen (ZMHU). **Non-type material.** Russia. 1 ♂, Volgograd oblast, Sarepta, 1872, coll. Staudinger, genitalia slide 3203 Mus. Vind. (NHMW); 1 ♂, Volgograd oblast, Volgograd, 25–31.v.1967, leg. V. Zouhar (ZMUC); 2 ♂, Orenburg oblast, near Burannoe, 18.viii.2006, leg. K. Nupponen (ZMUC); 1 ♂, same data, but 7.viii.2005, genitalia slide no. 2/11.X.2005 K. Nupponen (RCKN); 1 ♂, Orenburg oblast, 20 km S Pokrovka village, Schibendy valley, 21.viii.2006, leg. K. Nupponen, genitalia prep. (in glycerin) (RCKN); 2 ♂, S Ural, Orenburg distr., 40 km W Orsk, Guberlia village, 12–13.vii.2015, leg. H. Roweck & N. Savenkov (ECKU); 1 ♂, Dagestan oblast, Derbent, leg. Christoph (ZMHU). No collecting data. 2 ♂ (MZH); 4 ♂, coll. Staudinger (ZMHU).

##### Redescription.

Adult. *Male* (Figure 108). Wingspan 11–13 mm. Segment 2 of labial palpus short with sub-triangular scale brush of about same length as segment 3, brown on outer and lower surface, white on inner and upper surface; segment 3 about half as long as segment 2, white mottled with brown. Antennal scape with pecten of several hairs; flagellum ringed black and light brown. Head and thorax cream-white mottled with light brown; tegula whitish. Forewing white, more or less densely mottled blackish brown, especially along costa, in fold and in apical area; an irregular whitish streak from base almost to apex; two elongate black dots in fold; a black dot at end of cell; termen with black scales; fringes light grey with darker fringe line. Hindwing light grey with concolorous fringes.

*Female*. Unknown.

*Variation*. There is variation in the amount of blackish brown scales on the forewing. Worn specimens become whitish.

*Male genitalia* (Figure 234). Uncus large, sub-square, latero-apically convex; gnathos hook slender, slightly longer than uncus, strongly bent at about one-fifth, distal part straight, apically pointed; anterior margin of tegumen with shallow, rounded emargination and small additional emargination medially; pedunculi of moderate size; valva moderately stout, distally weakly curved, extending to about middle of uncus; saccular area densely covered with setae, without separated sacculus; posterior margin of vinculum with shallow medial emargination, without distinct lateral humps, vincular sclerite elongated sub-triangular, with strongly sclerotised posterior edge; saccus prominent, V-shaped, evenly tapered to rounded apex, ratio maximum width to length approximately 0.85, posterior margin with broadly rounded mediolateral projections, separated by shallow incision, medial part with long sclerotised ridge from posterior margin almost to apex of saccus, lateral sclerites nearly length of maximum width of saccus, with distinctly bulged apex; phallus with bulbous coecum, distal two-thirds slender, straight, dorsal part with distinct sub-triangular sclerotisation sub-apically.

*Female genitalia*. Unknown.

##### Diagnosis.

*Megacraspedusattritellus* is characterised by segment 3 of the labial palpus being short, by the pecten of the antennal scape, and by its blackish brown forewings with a whitish longitudinal streak and three elongate black spots. Among species with a scale brush at segment 2 and short segment 3 of the labial palpus it resembles *M.leuca* most, but that species is larger, and has lighter forewings with indistinct black dots. It is furthermore similar to *M.nupponeni* sp. n. (p 135) and *M.orenburgensis* (p 144). The male genitalia are very similar to *M.cerussatellus* (Figure 233) from which they differ in characters such as the slightly shorter and more slender gnathos hook, the more slender saccus and in particular the comparatively shorter and less bulbous basal part of the phallus, with a characteristic sub-apical sclerotisation. They differ from *M.consortiella* (Figure 235) e.g., in the sub-square shape of the uncus and are easily distinguished from *M.leuca* by the distinctly smaller and V-shaped saccus.

##### Molecular data.

BIN BOLD:ACE2700 (n = 3), BOLD:ACB0770 (n = 1). Genetically variable species. The intraspecific divergence of the barcode region is low in one cluster, with mean 0.1% and maximum divergence of 0.2% and unknown in the second cluster. The maximum distance between both clusters is 4.2%, indicating possible cryptic diversity, whereas the distance to the nearest neighbour *M.leuca* is 6.8% (p-dist).

##### Distribution.

South-western Russia.

##### Biology.

Host plant and early stages are unknown. The adults have been collected during July and August in steppe grassland at unreported altitudes (Junnilainen and Nupponen 2010: 12), with a single specimen found in May.

##### Remarks.

*Megacraspedusattritellus* was described from four males collected by H Christoph in Sarepta (now Krasnoarmeysk), southern Russia (Staudinger 1871). A lectotype, already labelled as such by K Sattler, is here designated in order to fix the identity of the species and conserve stability of nomenclature. Anikin and Zolotuhin (2017: 469, pl. 35, fig. 7) figured the lectotype but did not designate it following ICZN (1999) Art. 74.7.3.

#### Megacraspedus consortiella

Taxon classificationAnimaliaLepidopteraGelechiidae

Caradja, 1920

Megacraspedus consortiella Caradja, 1920: 117.

##### Examined material.

**Holotype** ♂, [Kyrgyzstan] “Alai [mountains]” “HOLOTYPE *Megacraspedus consortiella* ♂ Car. DES. Dr. A. POPESCU-GORJ Romania” “176436” “CIS-Korea Microlep. 4218 *Megacraspedus consortiella* Car. – Alai, Himalaya K. T. Park ‘96” (MGAB) [photographs examined].

**Non-type material.** Kyrgyzstan. 1 ♂, Alai mts, Tengiz-Bai Gate, 2800–2900 m, 12.vii.2011, leg. A. Pototski (RCAP); 3 ♂, Alai mts, Ak-Bosogo, 2725 m, 31.vii.2010, leg. A. Pototski (RCAP, ZMUC); 2 ♂, Alai mts, Pamirsky trakt, near Ak-Bosogo village, 2725 m, 20.vii.2010, leg. K. Nupponen & R. Haverinen, genitalia slide GU 17/1498 ♂ Huemer (RCKN).

##### Redescription.

**Figures 109–114.**
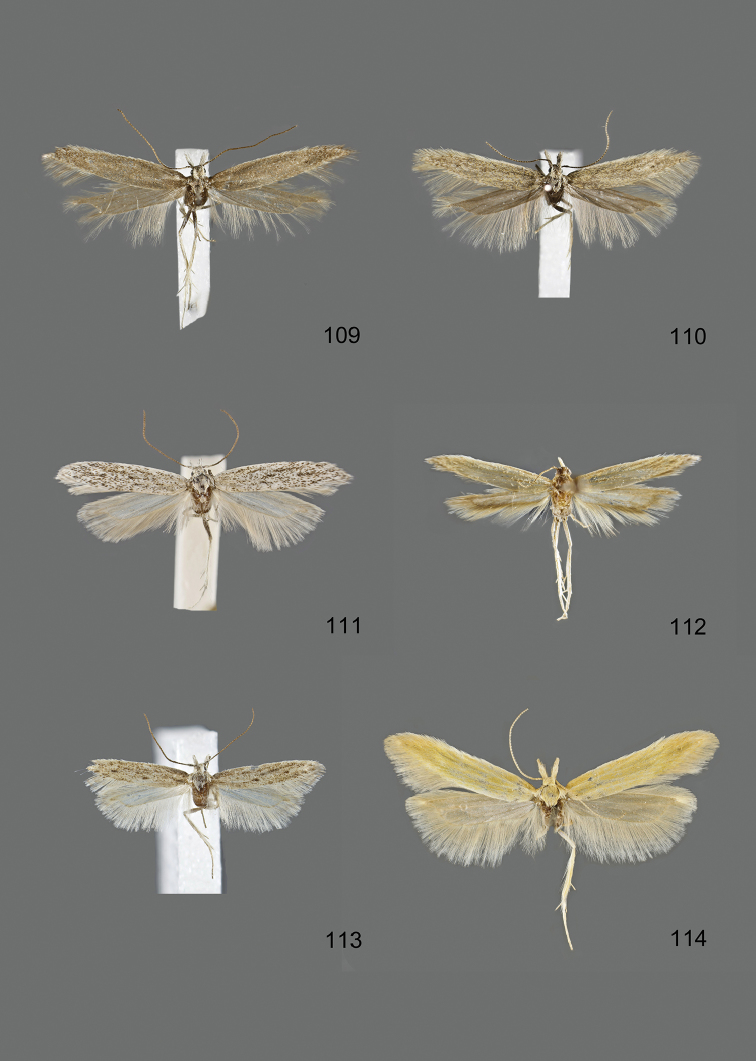
*Megacraspedus* adults in dorsal view. **109***M.consortiella* Caradja, 1920 – male, Kyrgyzstan (RCKN) **110***M.pototskii* sp. n. – Holotype, male, Kyrgyzstan (RCKN) **111***M.leuca* (Filipjev, 1929) – male, Russia (NMPC) **112***M.leuca* (Filipjev, 1929) – Paratype (*kaszabianus*), female, Mongolia (HNHM) **113***M.orenburgensis* Junnilainen & Nupponen, 2010 – Holotype, male, Russia (RCKN) **114***M.lagopellus* Herrich-Schäffer, 1860 – male, Hungary (NHMW).

Adult. *Male* (Figure 109). Wingspan 13–15 mm. Segment 2 of labial palpus with scale brush longer than segment 3, blackish on outer and inner surface, white on upper surface; segment 3 white with some black towards tip. Antennal scape with pecten of a single hair; flagellum blackish grey, indistinctly ringed lighter. Head grey; thorax and tegula as forewing. Forewing grey with slightly lighter fold; costa white from 1/5; an indistinct black dot in fold, and a black spot at end of cell; a few black scales along termen; fringes light grey with darker fringe line. Hindwing grey with grey fringes.

*Female*. Unknown.

*Variation*. In four of the examined specimens the black dot at end of the fold (which is present in the holotype) is almost obsolete and the hair at the base of the antennal scape is missing, but these specimens are rather worn.

*Male genitalia* (Figure 235). Uncus large, sub-rectangular, approximately 1.5 times longer than broad, latero-apically convex, apex weakly excavated; gnathos hook slender, about one-third longer than uncus, strongly bent at about one-quarter, distal part straight, apically pointed; anterior margin of tegumen with shallow, rounded emargination and small additional emargination medially; pedunculi of moderate size; valva moderately stout, distally weakly curved, extending to basal third of uncus; saccular area densely covered with setae, without separated sacculus; posterior margin of vinculum with shallow medial emargination, with lateral humps, knob-like sclerite, vincular sclerite elongated sub-triangular, with weakly sclerotised edges; saccus prominent, nearly V-shaped, evenly tapered to broadly rounded apex, ratio maximum width to length approximately 0.65, posterior margin with broadly rounded mediolateral projections, separated by shallow incision, medial part with long sclerotised ridge from posterior margin almost to apex of saccus, lateral sclerites nearly length of maximum width of saccus, with distinctly bulged apex; phallus with weakly bulbous coecum, distal two-thirds slender, straight, medial part with group of minute spinules.

*Female genitalia.* Unknown.

##### Diagnosis.

*Megacraspedusconsortiella* is characterised by the grey forewings with a black dot at the end of the cell, and by having most of the margin of the costa white. It is very similar to and hardly separable from *M.pototskii* sp. n. (p 141). The male genitalia are similar to *M.cerussatellus* (Figure 233) and *M.attritellus* (Figure 234) from which they differ e.g., in the sub-rectangular shape of the uncus. They are easily distinguished from *M.leuca* (Figure 237) by the distinctly smaller saccus.

##### Molecular data.

BIN BOLD:ADG5879 (n = 1). The distance to the nearest neighbour *M.leuca* is 6.6% (p-dist).

##### Distribution.

Kyrgyzstan (Alai mountains).

##### Biology.

Host plant and early stages are unknown. The few adults known to date were collected from the middle of July to the end of July at altitudes from ca. 2700 to 2900 m.

##### Remarks.

*Megacraspedusconsortiella* was described from one male in good condition from “Alai” (= Alai Mountains in Kyrgyzstan and Tajikistan) (Caradja 1920: 117). Park (1996) figured the genitalia of the holotype.

#### Megacraspedus pototskii sp. n.

Taxon classificationAnimaliaLepidopteraGelechiidae

http://zoobank.org/85DE6271-8E32-44C3-B48C-794EC3B52DD1

##### Examined material.

**Holotype** ♂, “KYRGYZSTAN Alai mts 3650 m 39°38'51.1"N 72°14'00.1"E Tengiz-Bai pass 23.7.2010 K. Nupponen & R. Haverinen leg.” “DNA Barcode TLMF Lep 23650”, genitalia prep. (in glycerin) (RCKN). **Paratypes.** Kyrgyzstan. 3 ♂, same data as holotype, but genitalia slide GU 17/1492 Huemer (RCKN, TLMF); 1 ♂, Alai mts, Ters-Agar Pass, 3627 m, 28.vii.2011 (abdomen missing) leg. A. Pototski (RCAP).

##### Description.

Adult. *Male* (Figure 110). Wingspan 12–16 mm. Segment 2 of labial palpus with scale brush longer than segment 3, light grey-brown, darker on outer surface; segment 3 with whitish base and black tip. Antennal scape with pecten of 1–3 hairs; flagellum blackish grey, ringed with light grey. Head, thorax and tegula as forewing. Forewing light yellowish grey; margin of costa white from 1/5; a black dot at end of cell; veins indistinctly dusted with grey scales; some black-tipped scales before apex; fringes light grey with darker fringe line. Hindwing grey with light grey fringes.

*Female*. Unknown.

*Variation*. There is a slight variation in the amount of light yellowish in the forewing, and there can be indistinct black dots fold, and in the middle of wing at 3/5. The few examined specimens are also variable in size.

*Male genitalia* (Figure 236). Uncus sub-rectangular, approximately 1.5 times longer than broad, apical margin laterally rounded, medially weaky indented; gnathos hook moderately slender, distal two-thirds widened, apically pointed, slightly longer than uncus, abruptly bent at right angles at one-third; tegumen with broad and moderately deep anterior emargination, additional weak emargination medially, with short sclerotised sublateral ridges merged near middle of tegumen; pedunculi small, double lobed; valva moderately broad, extending slightly beyond base of uncus, basally widened, distal part broad, with longitudinal sclerotised ridge, apical fifth converged and distorted, rounded, setose; saccular area without separated sacculus; posterior margin of vinculum with shallow emargination, curved lateral hump, vincular sclerites suboval with sclerotised ridge; saccus massive, stout, broadly V-shaped with abruptly tapered distal sixth and rounded apex, approximately 0.8 times length of valva, ratio maximum width to length approximately 0.8, posterior margin distinctly emarginated, with sinusoid mediolateral humps, medial part with long sclerotised ridge from posterior margin to subapical area, lateral sclerites approximately 0.7 times maximum width of saccus; phallus moderately slender, inflated coecum, about two times width of digitate two-thirds, medially with area of about 30 minute spinules, apically rounded.

*Female genitalia*. Unknown.

##### Diagnosis.

*Megacraspeduspototskii* sp. n. is characterised by the light yellowish grey forewings with a black dot at the end of the cell, and by having most of the margin of the costa white. It is very similar to and hardly separable from *M.consortiella* (Figure 109), although the latter seems to have more black-tipped scales along the termen. *Megacraspeduspototskii* sp. n. differs from the externally similar *M.consortiella* (Figure 235) in several characters of the male genitalia such as the broader uncus and gnathos hook, the absence of a knob-like sclerite of the vinculum and the field of minute spinules of the phallus.

##### Molecular data.

BIN BOLD:ADI8386 (n = 2). The intraspecific divergence of the barcode region is 0%. The distance to the nearest neighbour *M.ibericus* sp. n. is 9.1% (p-dist).

##### Distribution.

Kyrgyzstan (Alai mts).

##### Biology.

Host plant and early stages are unknown. The type-series was collected in the last third of July at high altitudes of ca. 3600 to nearly 3700 m.

##### Etymology.

The species (a noun in the genitive case) name is dedicated to Alexander Pototski, Estonia, who made valuable material of *Megacraspedus* from Asia available for our study.

#### Megacraspedus leuca

Taxon classificationAnimaliaLepidopteraGelechiidae

(Filipjev, 1929)

Nothris leuca Filipjev, 1929: 9, pl. 2a, fig. 3.
Megacraspedus kaszabianus Povolný, 1982: 193, figs 1–2, syn. n.

##### Examined material.

**Paratypes** of *M.kaszabianus*. Mongolia. 1 ♂, Gobi Altaj aimak, Chasat chajrchan ul mts, cca. 20 km S Somon Zargalan, 2400 m, 15.vii.1966, leg. Z. Kaszab; 1 ♂, Südgobi aimak, Gurban Sajchan ul, 15 km S Dalanzadgad, 1750 m, 13.vi.1967, leg. Z. Kaszab; 1 ♂, 1 ♀, Südgobi aimak, Somon Bulgan Talyn bulag, 1350 m, 5.vii.1967, genitalia slides GU 16/1446 ♂ Huemer, GU 16/1447 ♀ Huemer, leg. Z. Kaszab (HNHM). **Non-type material.** Russia. 1 ♂ Alai mts, Kuraisk hrebet, 2300 m, 13.vii.2001, leg. K. Nupponen, genitalia slide no. 3/21.X.2008 K. Nupponen (RCKN); 2 ♂, Altai rep., 15 km S Kosh-Agach, Thuja steppe, 1800 m, 10.viii.2000, leg. A. Bidzilya (ZMUC); 4 ♂, Altai rep., 6.5 km SW Kosh-Agach, Kurai env., 1550 m, 9–10.vii.2014, leg. J. Šumpich, genitalia slide GU 16/1408 ♂ Huemer (NMPC, TLMF); 19 ♂, Altai rep., 10 km NE Kosh-Agach, Kurai mts, Range valley of Tabozhok river, 2100 m, 2–4.viii.2016, leg. P. Huemer & B. Wiesmair; 1 ♂, Altai rep., 17 km NNE Kokorya village, Chikhacheva mts range, Talduair Mt., valley of Sajlyugem river, 30.vii–2.viii.2016, leg. P. Huemer & B. Wiesmair; 2 ♂, Altai rep., Aktasch env., 17.vi–2.vii.2009, leg. B. Schacht (all TLMF); 4 ♂, Tuva rep., 52°04'N, 94°22'E, Ust-Uljuk, steppe hills, 670 m, 3–5.vi.1995, leg. J. Jalava & J. Kullberg (MZH); 26 ♂, Tuva rep., 50°01'N, 95°03'E, Lake Tere Khol, sand dunes, 1150 m, 9–12.vi.1995, leg. J. Jalava & J. Kullberg (MZH, ZMUC); 8 ♂, 1 ♀, Tuva rep., 50°16'N, 94°54'E, ca. 25 km W Erzin, steppe/stony slopes, 1250 m, 7–11.vi.1995, leg. J. Jalava & J. Kullberg (MZH); 18 ♂, Tuva rep., 50°44'N, 93°08'E, E Tannu-Ola mts, Irbitei r., stony steppe slopes, 1000 m, 13–16.vi.1995, leg. J. Jalava & J. Kullberg (MZH); 1 ♂, Tuva rep., 50°45'N, 94°29'E, E Tannu-Ola mts, 5 km ENE Khol-Oozha, steppe slopes, 1000 m, 13–16.vi.1995, leg. J. Jalava & J. Kullberg (MZH).

##### Redescription.

Adult. *Male* (Figure 111). Wingspan 15–18 mm. Segment 2 of labial palpus with sub-rectangular scale brush longer than segment 3, blackish brown mottled with white, especially on upper surface; segment 3 about two-thirds length of segment 2, white mottled with black. Antennal scape with pecten of several hairs; flagellum light brown, indistinctly ringed with black. Head, thorax and tegula whitish mottled with light brown. Forewing cream whitish, mottled with brownish- or blackish-tipped scales; indistinct blackish blown spots in yellow-white fold and at 2/3 and 3/4 in middle of wing; fringes whitish grey with two grey fringe lines. Hindwing light grey with concolorous fringes.

*Female* (Figure 112). Wingspan 15 mm. Otherwise similar to male.

*Variation*. The amount of brownish- or blackish-tipped scales of the forewing varies; they are normally scattered over the wing but can also form lines along the veins. The blackish brown dots on the forewing can be more or less distinct. One specimen (in good condition) is almost without dark scales.

*Male genitalia* (Figure 237). Uncus large, sub-square, apical corners rounded, apical edge straight to weakly emarginated; gnathos hook evenly slender, apically pointed, almost twice length of uncus, bent at right angles at about one-quarter; anterior margin of tegumen with broad and shallow U-shaped emargination, anteromedially small additional emargination; pedunculi small, suboval; valva stout, extending slightly beyond base of uncus, basally weakly inflated, digitate distal part weakly tapered to slightly pointed apex; saccular area densely covered with setae, longitudinal ridge present, without separated sacculus; posterior margin of vinculum shallow medial emargination, without distinct lateral humps, vincular sclerite elongated sub-triangular, with sclerotised posterior edge; saccus prominent, longer than valva, U-shaped, ratio maximum width to length approximately 0.7, posterior margin with broadly rounded shallow projections, separated by small incision, medial part with long sclerotised ridge from posterior margin to apex of saccus, lateral sclerites approximately 0.8 times length of maximum width of saccus, with distinctly bulged apex; phallus with weakly inflated coecum, about two times wider than distal part, distal part more than twice length of coecum, sclerotised dorsal ridge with one to five small teeth.

*Female genitalia* (Figure 292). Papilla analis weakly sclerotised, moderately small, apically rounded; apophysis posterior slender rod-like, short, slightly longer than apophysis anterior, posterior end with small transverse sclerotised zone; segment VIII very short, laterally strongly sclerotised, medially largely membranous; anterior edge of segment VIII laterally rod-like, connected with apophysis anterior, subgenital plate reduced, ostium bursae without bordering sclerotisations; apophysis anterior rod-like, almost five times length of segment VIII, posteriorly broadly connected with segment VIII by sclerotised band; colliculum short, ring-shaped; ductus bursae slender, weakly widening to hardly delimited, oblong corpus bursae; signum reduced.

##### Diagnosis.

*Megacraspedusleuca* is characterised by having the antennal scape with a pecten of several hairs, and by the having the cream whitish grey forewings mottled with brownish- or blackish-tipped scales. It resembles *M.multispinella* (Figs 103–104), which is smaller and has greyish white forewings with black scales along the apical margins. It is furthermore similar to *M.imparellus* (p 130), *M.orenburgensis* (p 144), and *M.attritellus* (p 138). The male genitalia are unmistakable and in particular the prominent, long and slender U-shaped saccus is unique in *Megacraspedus*. The female genitalia are characterised by several unique structures such as the short segment VIII with a membranous medial part, the comparatively very long apophysis anterior, and the absence of a signum.

##### Molecular data.

BIN BOLD:ACB3260 (n = 10). The intraspecific divergence of the barcode region is low with mean 0.03% and maximum divergence of 0.2%. The distance to the nearest neighbour *M.skulei* is 6.6% (p-dist).

##### Distribution.

Asian part of Russia, Mongolia.

##### Biology.

Host plant and early stages are unknown. Bidzilya (2002: 64) collected adults in numbers at artificial light placed among tussocks of *Achnatherumsplendens* (Trin.) Nevski (= *Stipasplendens* Trin.). *M.leuca* is by far the most common species of the genus in Central Asia, inhabiting a wide range of different steppe habitats. According to Povolný (1982) it was collected from June to August in grass-, sand- and salt steppes at altitudes of between 1000 and 2400 m. Adults are easily attracted to articificial light (PH, pers. obs.).

##### Remarks.

*Nothrisleuca* was described from one male collected at Munku-Sardyk (=Mönkh-Saridak) in the Sajan Mountains at the border between Russia and Mongolia (Filipjev 1929). The holotype in ZISP was studied and figured by Junnilainen and Nupponen (2010: 11).

*Megacraspeduskaszabianus* was described from several hundred males collected in Mongolia by Z. Kaszab (Povolný 1982). We have been able to examine paratypes, which fully agree with *M.leuca* in every detail. We therefore formally synonymise *M.kaszabianus* with *M.leuca* (syn. n.).

#### Megacraspedus orenburgensis

Taxon classificationAnimaliaLepidopteraGelechiidae

Junnilainen & Nupponen, 2010

Megacraspedus orenburgensis Junnilainen & Nupponen, 2010: 13, figs 20–21, 27

##### Examined material.

**Holotype** ♂, “RUSSIA S-Ural Orenburg district Pokrovka village 20 km S Schibendy valley 21.6.1999 T. & K. Nupponen leg.” “Prep. no:03022405 Det J. Junnilainen” “*Megacraspedus orenburgensis* sp. n. ♂ Det J. Junnilainen” “HOLOTYPE *Megacraspedus orenburgensis* Junnilainen & K. Nupponen” (RCKN). **Paratype.** 1 ♂, same data as holotype, but 2.viii.2005, leg. K. Nupponen, genitalia slide no. 2/17.IV2006 K. (RCKN).

##### Redescription.

Adult. *Male* (Figure 113). Wingspan 12 mm. Segment 2 of labial palpus with scale brush exceeding segment 3, white mottled with blackish brown, with upper surface white; segment 3 about half as long as segment 2, white with blackish brown tip. Antennal scape with pecten of about ten hairs; flagellum ringed black and brown. Head and thorax cream-white mottled with light brown; tegula whitish. Forewing white, mottled with black- and brown-tipped scales; base of costa grey-brown; a black dot at base of fold and one at 2/5 of wing; an indistinct black spot at 3/5 in middle of the wing and a distinct black spot at end of cell; termen with black scales; fringes whitish grey with darker fringe line. Hindwing whitish grey with a dark line along margin in apical part; fringes light grey.

*Female*. Unknown.

*Variation*. Unknown.

*Male genitalia* (Figure 238). Uncus large, sub-rectangular, slightly longer than wide, apical corners rounded; gnathos hook stout, at about one-fifth, apically pointed, slightly longer than uncus; anterior margin of tegumen with broad emargination, anteromedially small additional emargination; pedunculi moderately small, sub-triangular; valva stout, extending to about middle of uncus, basally inflated, broadly digitate distal part weakly tapered to blunt apex; saccular area warty, without separated sacculus; posterior margin of vinculum with shallow medial emargination, without distinct lateral humps, vincular sclerite broadly elongated, with sclerotised proximo-posterior edge; saccus large, slightly shorter than valva, broad, weakly converged, distally abruptly tapered to rounded apex, ratio maximum width to length approximately 0.7, posterior margin bulged with rounded shallow projections, separated by small incision, medial part with long, weakly sclerotised ridge from posterior margin to subapical area of saccus, lateral sclerites slightly shorter than maximum width of saccus, with strongly bulged apex; phallus with weakly inflated coecum, distal two-thirds stout, straight, with sclerotised folds, posteriomedial part widened, longitudinal row of 6–7 teeth dorsodistally.

*Female genitalia*. Unknown.

##### Diagnosis.

*Megacraspedusorenburgensis* is characterised by the short segment 3 of the labial palpus and by the pecten of the antennal scape having many hairs. Among species with a scale brush on segment 2 and a short segment 3 of the labial palps it mostly resembles *M.leuca* (Figs 111–112), which is larger and has indistinct black dots at the forewings, and *M.attritellus* (Figure 108), which has the costa of the forewings blackish brown. The male genitalia are unmistakable and in particular the teeth-like sclerotisations of the phallus are unique in *Megacraspedus*.

##### Molecular data.

Not available, no specimen was available for barcoding.

##### Distribution.

Russia (S. Ural).

##### Biology.

Host plant and early stages are unknown. The adults have been collected from June to early August by sweeping just before sunset on chalk steppe at low altitudes from 170 to 230 m (Junnilainen and Nupponen 2010).

##### Remarks.

*Megacraspedusorenburgensis* was described from two males collected in the southern Urals, Russia (Junnilainen and Nupponen 2010).

### *Megacraspeduslagopellus* species group

The *M.lagopellus* species group includes five species: *M.lagopellus*, *M.coleophorodes*, *M.feminensis* sp. n., *M.kirgizicus* sp. n., and *M.argyroneurellus*.

External morphology. Labial palpus long, segment 2 with long scale brush; segment 3 short and narrow. Antennal scape without or with a single, soft hair. Wingspan (males) 17–26 mm. Forewing with whitish veins, and in some species with white costa, but without black spots. Known females (wingspan 21–25 mm) with slightly narrower wings, otherwise similar to males.

Genitalia morphology. Male genitalia. Uncus sub-triangular, short, apical part slender, partially rod-like, sub-basally with or without lateral flaps; gnathos hook straight, large to medium sized, strongly sclerotised; anterior margin of tegumen broadly emarginated with small medial excavation; valva broad, without separate sacculus, apex with distinct spine; saccus sub-triangular, posterior margin strongly arched, medial part without sclerotised ridge; phallus straight, with weakly bulbous coecum, distal three-quarters slender, rod-like, with small dorsoapical tooth.

Female genitalia. Papilla analis laterally compressed, extruding from tip of abdomen, strongly sclerotised, with or without fine longitudinal lines, apically pointed; apophysis posterior and anterior moderately short; segment VIII strongly sclerotised, subgenital plate without specialised structures, except for lateral ride delimiting ostium bursae; signum small, rounded plate.

Diagnosticremarks. The *M.lagopellus* species group is mainly defined by the characteristic labial palpus and diagnostic structures of the male genitalia. The small and pointed uncus, the comparatively massive gnathos hook, the strongly arched posterior edge of the saccus, and in particular the broad valva with an apical spine are diagnostic. The highly adapted ovipositor in the female genitalia is only shared with *M.kazakhstanicus* sp. n., but females are unknown for the majority of *Megacraspedus*. This structure seems suitable for a specialised form of oviposition inside the host plant.

#### Megacraspedus lagopellus

Taxon classificationAnimaliaLepidopteraGelechiidae

Herrich-Schäffer, 1860

Megacraspedus lagopellus Herrich-Schäffer, 1860: 13, pl. [14], fig. 81.

##### Examined material.

Hungary. 1 ♂, Ofen [Budapest], 1868, leg. Anker, genitalia slide Mus. Vind. 16.656; 1 ♀, same data, but 1874, genitalia slide NM 16657 (all NHMW). Kazakhstan. 1 ♂, 6 ♀, Kapchagai, 600 m, 18.v.2004, leg. A. Pototski & U. Jürivete (RCAP, ZMUC). Russia. 4 ♂, 7 ♀, S-Ural, Orenburg oblast, 20 km S Pokrovka village, Schibendy valley, 3–7.vi.1998, leg. J. Junnilainen, K. & T. Nupponen (RCKN, ZMUC); 2 ♂, same data, but 10.vi.2001, leg. K. Nupponen (RCKN, ZMUC); 1 ♀, same data, but 2.vii.2003, leg. K. Nupponen; 2 ♂, same data, but 30.v.2004, leg. K. Nupponen (all ZMUC); 1 ♂, 7 ♀, S-Ural, Orenburg oblast, Orenburgskij zap., Burtinskil step, 12–17.vi.2007, leg. J. Kullberg & M. Zalewski (MZH, ZMUC); 1 ♂, S-Ural, Orenburg oblast, 40 km W Orsk, near Guberlja vill., 26.vi.2003, leg. K. Nupponen (ECKU).

##### Redescription.

Adult. *Male* (Figure 114). Wingspan 18–20 mm. Segment 2 of labial palpus with long scale brush, brown in middle of outer and inner surface, whitish on lower and upper surface; segment 3 thin, white. Antennal scape without pecten; flagellum brown, ringed with white. Head whitish brown, thorax and tegula as forewing. Forewing light yellowish, darker in costal part; veins indistinctly white; costa white; fringes whitish grey. Hindwing light grey with concolorous fringes.

**Figures 115–120.**
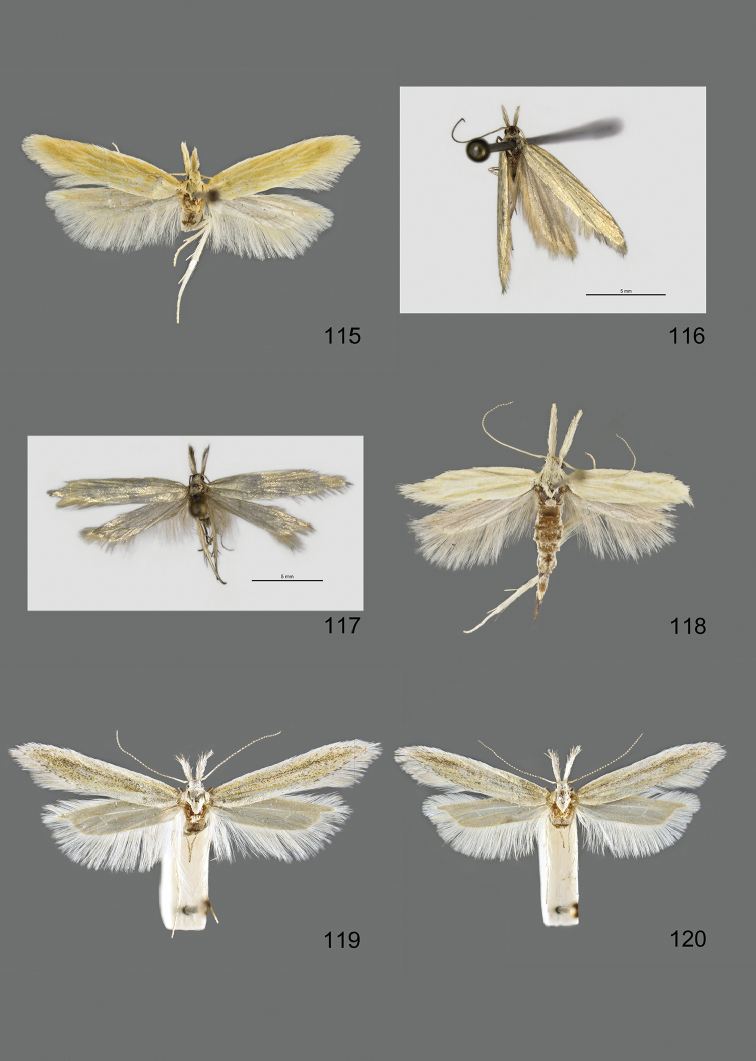
*Megacraspedus* adults in dorsal view. **115***M.lagopellus* Herrich-Schäffer, 1860 – female, Hungary (NHMW) **116***M.coleophorodes* (Li & Zheng, 1995) – Holotype, male, China (NKU) **117***M.coleophorodes* (Li & Zheng, 1995) – Paratype, female, China, (NKU) **118***M.feminensis* sp. n. – Paratype, female, Kazakhstan (RCKN) **119***M.kirgizicus* sp. n. – Paratype, male, Afghanistan (NHMW) **120***M.kirgizicus* sp. n. – Paratype, female, Afghanistan (NHMW).

*Female* (Figure 115). Wingspan 17–19 mm. Antennal scape without pecten; flagellum white, indistinctly ringed darker; colour of wings slightly lighter than male; otherwise similar to male.

*Variation*. The brownish part of the labial palpus can be more or less distinct, and the forewing colour can vary from whitish yellow to light yellow. Specimens tend to become greasy and the whitish parts of the head and body thereby turn brown.

*Male genitalia* (Figure 239). Uncus slender, short; gnathos hook strong, slightly longer than uncus, weakly curved with pointed apex; tegumen with medially converging sclerotised anterior ridges, anterior margin of tegumen with moderately shallow emargination, medially additional U-shaped excavation; pedunculi large, weakly demarcated, suboval; valva straight, massive, broad, extending almost to apex of uncus, apically rounded, with small spine, distal area covered with setae, without separated sacculus; posterior margin of vinculum with weakly developed oblique lateral humps, vincular sclerites elongated, sub-triangular; saccus sub-triangular, evenly tapered, broadly rounded apex, ratio maximum width to length approximately 0.8, posterior margin arched, without medial emargination, medial part without sclerotised ridge, lateral sclerites slightly shorter than maximum width of saccus; phallus straight, with weakly bulbous coecum, distal three-quarters slender, rod-like, with small dorsoapical tooth.

*Female genitalia* (Figure 293). Papilla analis laterally compressed, extruding from tip of abdomen, strongly sclerotised, large, approximately 1.4 mm long, posteriorly narrowing, weakly curved, ventral edge convex, dorsal edge concave, apex distinctly pointed; apophysis posterior rod-like, approximately 2.6 mm long, posterior end pointed, anterior end rounded; segment VIII about 1 mm long, strongly sclerotised except for less sclerotised ventromedial zone; subgenital plate almost without modified sclerotisations, anterior edge of segment straight, small elongated ostium bursae delimited by sclerotised lateral ridge; apophysis anterior rod-like, about length of segment VIII; colliculum short, sclerotised; ductus bursae gradually widening to weakly delimited corpus bursae, posterior part with granulate microsculpture, corpus bursae suboval, entire length of ductus and corpus bursae approximately 2.2 mm; signum moderately small, rounded, spiny plate.

##### Diagnosis.

**Figures 121–126.**
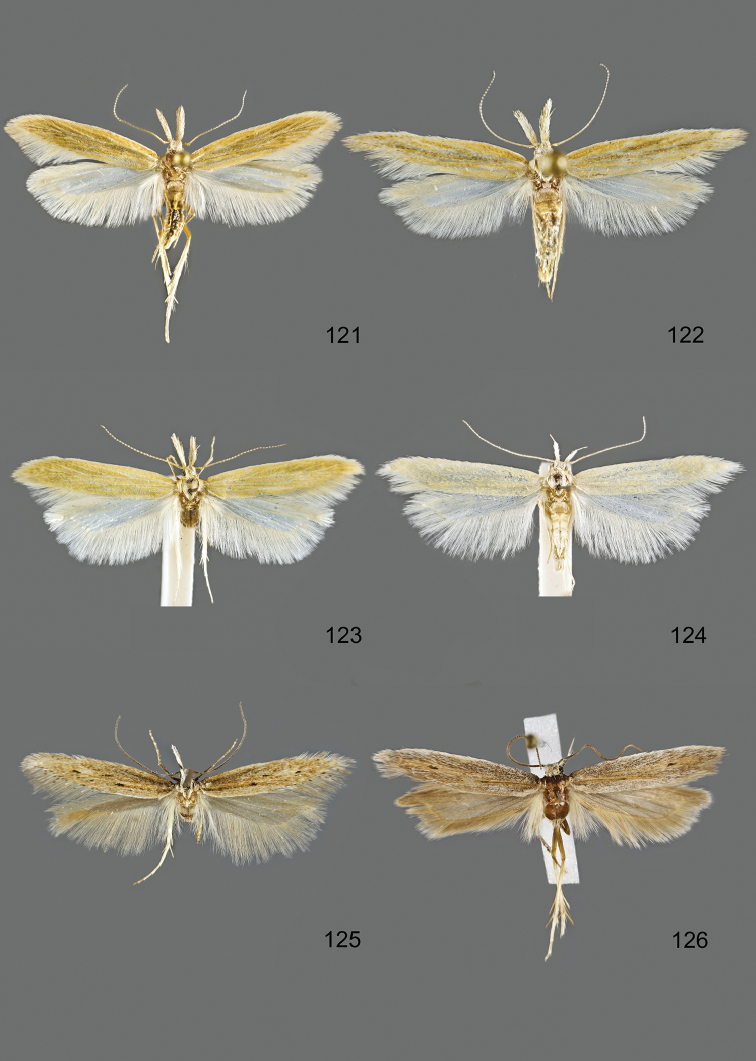
*Megacraspedus* adults in dorsal view. **121***M.argyroneurellus* Staudinger, 1871 – male, Russia (NMPC) **122***M.argyroneurellus* Staudinger, 1871 – female, Russia (NMPC) **123***M.argyroneurellus* Staudinger, 1871 – male, Turkey (RCEA) **124***M.argyroneurellus* Staudinger, 1871 – male, Turkey (RCEA) **125***M.ibericus* sp. n. – Paratype, male, Spain (TLMF) **126***M.violacellum* (Chrétien, 1915) – male, Tunisia (TLMF).

*Megacraspeduslagopellus* is characterised by the long scale brush on segment 2 of the labial palps and by its light yellowish forewings with indistinct white on the veins. It resembles the larger *M.argyroneurellus* (Figs 121–124), but that species has more distinct white veins. It is furthermore similar to *M.coleophorodes*, (see below). The male genitalia are similar to *M.coleophorodes* (Figure 240) but differ in the unique shape of the uncus and the broader valva. The female genitalia are similar to *M.coleophorodes* (Figure 294) but differ in the less pointed papilla analis. The species can be separated from *M.feminensis* sp. n. (Figure 295) e.g., by the much smaller and pointed papilla analis.

##### Molecular data.

BIN BOLD:ACB0458 (n = 2). The intraspecific divergence of the barcode region is low with maximum divergence of 0.2%. The distance to the nearest neighbour *M.feminensis* sp. n. is 7.8% (p-dist).

##### Distribution.

Hungary, Kazakhstan, Russia (S. Ural and Volgograd oblast).

##### Biology.

Host plant and early stages are unknown. The adults have been collected from the middle of May to the middle of July at altitudes up to 600 m.

##### Remarks.

*Megacraspeduslagopellus* was described from an unstated number of specimens from Sarepta (now Krasnoarmeysk), southern Russia (Herrich-Schäffer 1860). We have not been able to trace any syntypes, but the identity of this species is fixed by Herrich-Schäffer’s figure (op. cit.: fig. 81). *M.lagopellus* is unusual among *Megacraspedus* because more females than males were available for study.

#### Megacraspedus coleophorodes

Taxon classificationAnimaliaLepidopteraGelechiidae

(Li & Zheng, 1995)

Chilopselaphus coleophorodes Li & Zheng, 1995: 1, 10, figs 1–4.

##### Examined material.

**Holotype** ♂, China, Liancheng, Yongdeng County, Gansu Province, 6-July-1985, Coll. Houhun Li, genitalia slide no L94136 (NKU) [labels translated from Chinese, photographs examined]. **Paratype.** 1 ♀, Yanchi County, Ningxia Hui Autonomous Region, June-1986, Coll. Ren, genitalia slide no L92040 [labels translated from Chinese, photographs examined].

##### Redescription.

Adult. *Male* (Figure 116). Wingspan 21 mm. Segment 2 of labial palp with long scale brush, brown on upper and outer surface, whitish on lower and inner surface; segment 3 short. Antenna brown, indistinctly ringed with white. Head, thorax and tegula yellowish. Forewing yellowish, darker in costal part, lighter towards dorsum; veins white; fringe grey. Hindwing greyish.

*Female* (Figure 117). Wingspan 21–22 mm. Otherwise similar to male.

*Variation*. According to the original description the colour of the forewings varies from grey-brown to grey-white.

*Male genitalia* (Figure 240). Uncus sub-triangular, moderately small, apex pointed; gnathos hook about length of uncus, nearly straight with pointed apex; tegumen with medially converging sclerotised anterior ridges, anterior margin of tegumen with moderately shallow emargination, medially additional U-shaped excavation; pedunculi large, weakly demarcated, suboval; valva straight, broad, extending to about apex of uncus, apically rounded, with small spine, distal area covered with setae, without separated sacculus; posterior margin of vinculum with weakly developed oblique lateral humps, vincular sclerites elongated, sub-triangular; saccus sub-triangular, evenly tapered, medially weakly convex, rounded apex, ratio maximum width to length approximately 0.8, posterior margin arched, without medial emargination, medial part without sclerotised ridge, lateral sclerites shorter than maximum width of saccus; phallus straight, with weakly bulbous coecum, distal three-quarters slender, rod-like, with group of small spines dorsomedially and with distinct dorsoapical tooth.

*Female genitalia* (Figure 294). Papilla analis laterally compressed, extruding from tip of abdomen, strongly sclerotised, large, approximately 1.5 mm long, posteriorly narrowing abruptly, ventral edge weakly angled, dorsal edge predominantly straight, concave towards distinctly pointed, weakly curved apex; apophysis posterior rod-like, approximately 2.5 mm long, posterior end pointed, anterior end rounded; segment VIII about 1 mm long, strongly sclerotised except for less sclerotised ventromedial zone; subgenital plate almost without modified sclerotisations, anterior edge of segment straight, small elongated ostium bursae delimited by sclerotised lateral ridge; apophysis anterior rod-like, about length of segment VIII; colliculum short, sclerotised; ductus bursae widening to weakly delimited corpus bursae, corpus bursae suboval, entire length of ductus and corpus bursae 2.8 mm; signum small, rounded, spiny plate.

##### Diagnosis.

*Megacraspeduscoleophorodes* is characterised by its rather large size, the long scale brush on segment 2 of the labial palps, and by its yellowish forewings with white on the veins, but not on the costa. It resembles *M.argyroneurellus* (Figs 121–124) and *M.lagopellus* (Figs 114–115), but these species are white along the costa. It is furthermore similar to *M.feminensis* sp. n. (see below). The male genitalia are similar to *M.lagopellus* (Figure 239) but differ in the shape of the uncus, the more slender valva and the phallus. The female genitalia are similar to *M.lagopellus* (Figure 293) and *M.feminensis* sp. n. (Figure 295) but differ in the different shape of the papilla analis with a strongly pointed apex.

##### Molecular data.

Not available, no specimen was available for barcoding.

##### Distribution.

Northern China. Recently recorded from Korea (Koo et al. 2018).

##### Biology.

Host plant and early stages are unknown. The adults have been collected from May to August at unreported elevation.

##### Remarks.

*Chilopselaphuscoleophorodes* was described from one male (holotype) from the Gansu province and 11 females from Ningxia Hui Autonomous Region, China (Li and Zheng 1995). The above descriptions are based on photographs of the holotype and a paratype, as well as the original description.

#### Megacraspedus feminensis sp. n.

Taxon classificationAnimaliaLepidopteraGelechiidae

http://zoobank.org/2A0C5D7F-958D-4C61-B6D1-EB9F6E96AABB

##### Examined material.

**Holotype** ♀, “Kazakhstan 43°47'55"N 79°54'51"E Rahat Kuduk by Ili River shore tugai forest, 515 m 2.VI.2014 K. Nupponen & R. Haverinen leg.” “DNA Barcode TLMF Lep 23656” “GU 17/1495 ♀ P. Huemer” (RCKN). **Paratypes.** Kazakhstan. 6 ♀, same data as holotype (RCKN).

##### Description.

Adult. *Male*. Unknown.

*Female* (Figure 118). Wingspan 22 mm. Labial palpus long, about two-fifths length of antenna, porrect, light yellow to white on upper and lower surface, white mottled with brown on inner and outer surface, white on upper and lower surface; segment 3 reduced. Antennal scape without pecten, scape white, indistinctly ringed with light brown. Head, thorax and tegula whitish yellow. Forewing light yellow; veins white; a narrow white line along costa; fringes light yellowish grey. Hindwing grey, with light grey fringes.

*Variation*. The colour of the upper and lower surface of the labial palpus varies from predominantly light yellow to white. Worn specimens become lighter.

*Male genitalia*. Unknown.

*Female genitalia* (Figure 295). Papilla analis laterally compressed, extruding from tip of abdomen, strongly sclerotised, with fine longitudinal lines, large, approximately 1.6 mm long, posteriorly evenly tapered to pointed apex, ventral edge convex, dorsal margin nearly straight; apophysis posterior rod-like, about 3 mm long, posterior end pointed, anterior end rounded; segment VIII about 1 mm long, strongly sclerotised except for transverse suboval ventromedial zone; subgenital plate almost without modified sclerotisations, anterior edge of segment straight, small elongated ostium bursae delimited by sclerotised lateral ridge; apophysis anterior rod-like, about length of segment VIII, with slightly inflated apex; colliculum short, weakly sclerotised; ductus bursae widening to weakly delimited corpus bursae, posterior part with granulate microsculpture; corpus bursae suboval, entire length of ductus and corpus bursae approximately 2.6 mm; signum small, rounded, spiny plate.

##### Diagnosis.

*Megacraspedusfeminensis* sp. n. is characterised by its rather large size, by the long, porrecting labial palps, and by its yellowish forewings with white veins. It resembles *M.coleophorodes* (Figure 117), but diffiers in having porrecting labial palps and a thin white costa. *M.feminenis* sp. n. is also very similar to *M.argyroneurellus* (Figure 122) (especially matching lighter coloured specimens occurring in Turkey, Iran and Turkmenistan), and *M.lagopellus* (Figure 115), but these species have segment 3 of the labial palps well developed, and *M.argyroneurellus* has white hindwings. The female genitalia are similar to those of *M.lagopellus* (Figure 293) and *M.coleophorodes* (Figure 294) but differ e.g., in the large and less pointed papilla analis with longitudinal lines, and the shape of the membranous zone of segment VIII.

##### Molecular data.

BIN BOLD:ADJ1364 (n = 2). The intraspecific divergence of the barcode region is low with mean and maximum divergence of 0.2%. The distance to the nearest neighbour *M.lagopellus* is 7.8% (p-dist).

##### Distribution.

Kazakhstan.

##### Biology.

Host plant and early stages are unknown. The adults have been collected at the beginning of June at an altitude of ca. 500 m.

##### Etymology.

This species, being known only from females, is named in honour of the feminine gender. The name is a noun in apposition.

##### Remarks.

*Megacraspedusfeminensis* sp. n. is one of the few species in the genus with predominant numbers of collected females, resembling other closely related species of the *M.lagopellus* species group.

#### Megacraspedus kirgizicus sp. n.

Taxon classificationAnimaliaLepidopteraGelechiidae

http://zoobank.org/D109CAD5-462C-4A14-8530-ED5FD246DC5C

##### Examined material.

**Holotype** ♂, “Kirgisien/ Prov. Batken, Turkestan Geb., S Zardaly, Stat. Korgon HP 19 1750 m 8.6.2010 GPS 39°57'52"N;70°58'36"E leg. Dr. C. Wieser Kärntner Landesmuseum” “KLM Lep 00111” “GU 16/1406 ♂ P. Huemer” (LMK). **Paratypes.** Afghanistan. 1 ♂, 1 ♀, 10 km NW Kabul, 1900 m, 1.vi.1965, leg. F. Kasy & E. Vartian, genitalia slides in gylcerin (NHMW). Armenia. 136 ♂, 10 ♀, Goravan vill., S of Vedi, near Goravan Sands Reserve, sandy steppe, 956 m, 31.v.2017, leg. J. Šumpich (NMPC). Kazakhstan. 1 ♂, Charyn River, 1220 m, 1.vi.2014, leg. K. Nupponen & R. Haverinen; 3 ♂, Karatau mts, 50 km N of Turkestan town, 540 m, 8.v.2011, leg. K. Nupponen; 1 ♂, same data, but 11.v.2011; 1 ♂, 160 km E Bozoi settl., Ustyurt plateau, N slope, 205 m, 29.v.2011, leg. K. Nupponen; 1 ♂, Ustyurt Nat. Res., Kendyrli, 115 m, 20.v.2011, leg. K. Nupponen, genitalia slide GU 17/1491 ♂ Huemer; 1 ♂, 1 ♀, Ustyurt Nat. Res., Mametkazgan, 80 m, 22.v.2011, leg. K. Nupponen; 1 ♂, 40 km N Suzak settl., Muyunkum sands, 195 m, 10.v.2010, leg. K. Nupponen; 1 ♂, 1 ♀, Sengirkum sands, Terekurpa well, 70 m, 27.v.2011, leg. K. Nupponen (all RCKN). Kyrgyzstan. 1 ♀, same data as holotype, but 9.vi.2010; 1 ♀, Prov. Batken, Turkestan mts, valley Kalay Makhmud between Or-mazan-Suu and Alai Maidan HP 24 1800–2000 m, 10.6.2010, leg. Wieser, genitalia slide GU 16/1407 ♀ Huemer; 1 ♀, Prov. Osch, Distr. Kara Suu, Alai mts, S Tarylga, valley Ak Buura, 1550–1750 m, 5.vi.2010, leg. C. Wieser (all LMK); 4 ♂, 1 ♀, Alai mts, Tengiz-Bai Gate, 2800–2900 m, 12.vii.2011, leg. A. Pototski (RCAP, ZMUC); 8 ♂, 4 ♀, Alai mts, road Daroot-Korgon–Tengiz-Bai, 2820 m, 24.vii.2012, leg. A. Pototski (RCAP, ZMUC); 7 ♂, 2 ♀, Alai mts, Tengiz-Bai pass gate, 2820 m, 24.vii.2010, leg. K. Nupponen & R. Haverinen (RCKN, TLMF).

##### Description.

Adult. *Male* (Figure 119). Wingspan 19–26 mm. Labial palpus very long, porrect, dark greyish from white black-tipped scales, upper surface white; segment 3 less than one-third length of segment 2, white mottled with black. Antennal scape with single, soft hair; flagellum ringed black and white. Head, thorax and tegula whitish mottled with light grey-brown. Forewing light yellow mottled with black-tipped scales, especially along veins; a broad light grey streak from base to apex; margin of costa white; scattered black scales along termen; fringes light grey. Hindwing grey, with light grey fringes.

*Female* (Figure 120). Wingspan 23–25 mm. Similar to male apart from having a protruding ovipositor.

*Variation*. There is a slight variation in the colour of the head, thorax, and tegulae: from almost white to light grey-brown. The single, soft hair at the base of the antennal scape is missing in several of the examined specimens (especially the males), but that may be because the specimens have been damaged.

*Male genitalia* (Figs 241–242). Uncus sub-triangular, short, with sub-basal lateral flaps, rod-like distal part; gnathos hook massive, much longer and broader than uncus, distal part curved with shovel-shaped apex, pointed in lateral view; anterior margin of tegumen with moderately shallow emargination, medially U-shaped excavation; pedunculi weakly demarcated, suboval; valva straight, basal part wider than distal part, extending to about apex of uncus, apically pointed; distal area covered with setae, without separated sacculus; posterior margin of vinculum with weakly developed oblique lateral humps, vincular sclerites elongated, sub-triangular; saccus sub-triangular, with evenly tapered apex, ratio maximum width to length approximately 0.8, posterior margin arched, with shallow medial emargination, medial part without sclerotised ridge, lateral sclerites long, nearly length of maximum width of saccus; phallus slender, almost straight, suboval coecum about twice width of slender distal two-thirds, distal part with strongly sclerotised ridge and indistinct subapical sclerite.

*Female genitalia* (Figure 296). Papilla analis laterally compressed, extruding from tip of abdomen, strongly sclerotised, with fine longitudinal lines, large, approximately 1.8 mm long, subapically with emarginated dorsal and ventral edge resulting in acutely pointed apex, ventral edge convex, dorsal margin nearly straight; apophysis posterior stout, rod-like, about 3 mm long, posterior third widened to longitudinal sclerotised zone, anterior end rounded; segment VIII approximately 0.8 mm long, uniformly sclerotised; subgenital plate weakly modified, anterior edge of segment medially slightly convex, small suboval ostium bursae delimited by sclerotised lateral ridge; apophysis anterior rod-like, almost two times length of segment VIII; colliculum small, sclerotised; ductus bursae gradually widening to weakly delimited suboval corpus bursae; entire length of ductus and corpus bursae approximately 3.3 mm; signum moderately small, rounded, spiny plate.

**Diagnosis.***Megacraspeduskirgizicus* sp. n. is characterised by its large wingspan, the very long labial palps, and by the light yellow forewings with a broad light grey streak from base to apex.

It resembles *M.podolicus* (Figure 92) and *M.niphorrhoa* (Figs 85–86) but these two species are smaller and have shorter labial palps. The male genitalia are similar to *M.argyroneurellus* (Figs 243–244) but differ e.g., in the more slender apical part of the uncus and the slender phallus. The female genitalia are unmistakable in the *M.lagopellus* species group in the shape of the papilla analis and the apophysis posterior, and the very long apophysis anterior.

##### Molecular data.

BIN BOLD:AAW5664 (n = 6), BOLD:ACB3361 (n = 2). Genetically variable species. The intraspecific divergence of the barcode region is low in both clusters, with mean 0.25% and maximum divergence 0.5 in BIN BOLD:AAW5664 and maximum divergence 0.31% in the second BIN. The maximum distance between both BINs is 2.8%, indicating possible cryptic diversity. The minimum distance to the nearest neighbour *M.argyroneurellus* is 7.6% (p-dist).

##### Distribution.

Afghanistan, Armenia, Kazakhstan, Kyrgyzstan.

##### Biology.

Host plant and early stages are unknown. The adults have been collected from early June to the second half of July at altitudes from ca. 500 to 2900 m. In Armenia the biotope is sandy steppe.

##### Etymology.

This species is named after one of the countries of occurrence: the Kyrgyz Republic (also known as Kirgizia or Kyrgyzstan). The name is a masculine adjective.

#### Megacraspedus argyroneurellus

Taxon classificationAnimaliaLepidopteraGelechiidae

Staudinger, 1871

Megacraspedus argyroneurellus Staudinger, 1871: 316.

##### Examined material.

**Lectotype** ♂, **here designated**, [Russia, Volgograd oblast] “Lecto-type” “Sarepta [Krasnoamreysk] Chr.[istoph]” “Origin.” “Lectotype ♂ *Megacraspedus argyroneurellus* Stdgr teste K. Sattler, 1986” “ex coll. Staudinger” (ZMHU) [photographs examined]. **Paralectotype**, 1 ♀, “Para-lecto-type” “Origin.” “ex coll. Staudinger” (ZMHU) [photographs examined]. **Non-type material.** Armenia. 1 ♂, 40 km E Eriwan [Yerevan], Geghard, 1700 m, 24–25.vii.1976, leg. F. Kasy & E. Vartian; 1 ♂, same data, but 30–31.vii.1976 (NHMW). Iran. 1 ♀, Alborz mts, Nesa [Nissa], 25.vii.[no year given], leg. F. Brandt (ZMUC); 1 ♂, Elburs mts, Pelur, 2000 m, 27–28.vii.1936, leg. L. Schwingenschuss, genitalia slide Mus. Vind. 16.658; 1 ♀, same data, but 18–19.vii, coll. Wagner, genitalia slide Mus. Vind. 16.659 (NHMW); 4 ♂, 1 ♀, Elburs mts, southern part, 50 km N Tehran, Shemshak, 2300 m, 1–22.vii.1970, leg. E. Vartian (SMNK). Kyrgyzstan. 7 ♂, Zhetim-Bel Range, Eki-Naryn, 2800 m, 4.viii.2010, leg. A. Pototski (RZAP, ZMUC); 1 ♂, Tien-Shan, Moldo-Too mts, Min-Kush, circuitus, 2350 m, 1.viii.2000, leg. E. Rutjan, genitalia prep. (in glycerin) (RCAB); 1 ♂, Tien-Shan mts, Eki-Naryn, 2500 m, 4.viii.2010, leg. K. Nupponen & R. Haverinen (RCKN). Romania. 1 ♂, Dobrogea, Podişul Casimcei, Cheiele Dobrogei, 16.vii.2010, leg. S. & Z. Kovács (RCKO). Russia. 1 ♂, Volgograd oblast, Butkul Salt Lake, 18.vi.1999, leg. V. Karalius & J. Miatleuski (ECKU); 1 ♂, S-Ural, Orenburg oblast, Orenburgskij zap., Burtinskil step, 12–17.vi.2007, leg. J. Kullberg & M. Zalewski (MZH); 2 ♂, S-Ural, Orenburg oblast, 20 km S Pokrovka village, Schibendy valley, 17.vii.1998, leg. K. Nupponen (RCKN); 3 ♂, same data, but 1–2.vii.2003, leg. K. Nupponen (RCKN, ZMUC); 1 ♂, same data, but 7.vi.1998; 1 ♂, Orenburg oblast, near Burannoe vill., 20.vi.1999, leg. K. Nupponen (RCKN); 2 ♂, Orenburg oblast, near Burannoe, 3.vii.2003, leg. K. Nupponen; 1 ♂, Orenburg oblast, 40 km W Orsk, near Guberlja village, 26.vi.2003, leg. K. Nupponen (all ZMUC); 4 ♂, same data, but 12–13.vii.2015, leg. H. Roweck & N. Savenkov; 4 ♂, same data, but 25–26.vi.2017 (all ECKU); 4 ♂, Orenburg oblast, 6 km W Donskoje village, mount Verbljushka 24–28.vi.2009, leg. J. Šumpich (NMPC, ZMUC); 1 ♂, 2 ♀, same data, but 22–24.vii.2011 (NMPC, TLMF); 1 ♂, Orenburg oblast, 6 km W Donskoje village, mount Verbljushka, 19.vi.1999, leg. K. Nupponen (RCKN); 2 ♂, same data, but 3 km W, 7–8.vii.2015, leg. H. Roweck & N. Savenkov (ECKU); 4 ♂, Orenburg distr., Akbulak, Pokrovka village, 9–11.vii.2015, leg. H. Roweck & N. Savenkov; 5 ♂, same data, but 28–30.vi.2017 (all ECKU). Turkey. 4 ♂, prov. Adana, 18 km N Saimbeyli, 1700 m, 6.vii.1997, leg. K. Larsen ; 2 ♂, prov. Agri, Tahir Geçidi, 2550 m, 29.vii.1984, leg. G. Derra; 3 ♂, prov. Antalya, Beydağlari, 17 km E Göltaria, 1800 m, 8.viii.1997, leg. K. Larsen; 1 ♂, prov. Çorum, Çekerek, 1300 m, 17.vii.1989, leg. N. Esser & M. Fibiger; 2 ♂, prov. Erzincan, 40 km SW Erzincan, Kemah, 1100 m, 2.viii.1997, leg. Larsen, genitalia in tube; 1 ♀, prov. Erzurum, Kizilirmak, Köprüköy, 750 m, 20–22.vi.1969, leg. F. Hahn; 1 ♂, prov. Kayseri, 25 km S Kayseri, Erciyes Daği, 2200 m, 29.vii.1989, leg. N. Esser & M. Fibiger; 2 ♂, prov. Kayseri, İncesu, 1100 m, 28.vii.1996, leg. K. E. Stovgaard; 2 ♂, 2 ♀, prov. Kayseri, Ala Dağlar, Ulupinar, 1600 m, 30.vii.1998, leg. Larsen, genitalia in tube; 2 ♂, prov. Malatya, 20 km E. Elbistan, Nurhak Daği, 1900 m, 7.vii.1987, leg. M. Fibiger; 6 ♂, prov. Malatya, Gündüzbey, 1300 m, 26.vii.1998, leg. K. Larsen; 2 ♂, prov. Niğde, Aladağlar, Demirkazik, 2500 m, 24.vii.1994, leg. K. Larsen; 4 ♂, same data, but, 1600 m, 24–25.vii.1994; 1 ♂, same data, but 1700 m, 30.vii.1997; 2 ♂, prov. Niğde, Çiftlik, 1650 m, 24.vii.1996, leg. K. E. Stovgaard; 8 ♂, prov. Sivas, 5 km W Gürün, 1700 m, 5.vii.1987, leg. M. Fibiger, genitalia slide 6515 Hendriksen; 1 ♂, prov. Sivas, 10 km W Gürün, 1650 m, 27.vii.1989, leg. N. Esser & M. Fibiger; 2 ♂, prov. Sivas, 10 km S Gürün, Gökpinar, 1500 m, 1.vii.1997, leg. Larsen; 4 ♂, same data, but 12 km S Gürün, 25.vii.1998, genitalia in tube; 1 ♂, same data, but 11.vii.2000 (all ZMUC) 1 ♂, W Gürün, 1300 m, 22.vi.1969, leg. E. Arenberger, genitalia slide GU/16/1415 Huemer; 1 ♂, Tuz Gölü, N beach, 3.vii.1968, leg. E. Arenberger (all RCEA). Ukraine. Crimea, genitalia slide GU 16/1427 Huemer (RCAB).

##### Redescription.

Adult. *Male* (Figs 121, 123–124). Wingspan 18–25 mm. Labial palpus long, segment 2 with long scale brush, light brown, whitish on lower and upper surface; segment 3 narrow, rather short, cream coloured. Antennal scape with one soft hair pecten; flagellum whitish brown at base, becoming ringed white and brown towards apex. Head yellowish white; thorax and tegula as forewing. Costal part of forewing yellow-brown, middle part brown, dorsal part light yellow; indistinct dark brown dots along termen; veins silvery white; fringes light grey. Hindwing white with cream-white fringes.

*Female* (Figure 122). Wingspan 19–22 mm. Similar to male.

*Variation*. The size of the brownish area in the middle of the forewing varies and can extend into the apical area or alternatively be largely reduced. Specimens from the south-eastern part of the distribution range have no brown area in the middle of the forewing (see remarks below). The hair at the base of the antennal scape is easily broken.

*Male genitalia* (Figs 243–244). Uncus sub-triangular, short, with sub-basal lateral flaps, moderately broad rod-like apex; gnathos hook massive, much longer and broader than uncus, distal part curved with shovel-shaped apex, pointed in lateral view; tegumen with medially converging sclerotised anterior ridges, anterior margin with moderately shallow emargination, medially additional U-shaped excavation; pedunculi weakly demarcated, large suboval; valva straight, basal part wider than distal part, extending slightly beyond apex of uncus, apically pointed, distal area covered with setae, without separated sacculus; posterior margin of vinculum with weakly developed oblique lateral humps, vincular sclerites broadly suboval; saccus with some variation, sub-triangular, with evenly tapered to moderately broad apex, ratio maximum width to length approximately 0.8, posterior margin arched, with shallow medial emargination, medial part without sclerotised ridge, lateral sclerites long, approximately 0.8 times length of maximum width of saccus; phallus moderately slender, almost straight, suboval coecum large, about three times width and half length of slender distal part, with weakly sclerotised ridge distally.

*Female genitalia* (Figure 297). Papilla analis laterally compressed, extruding from tip of abdomen, strongly sclerotised, with fine longitudinal lines, large, approximately 1.6 mm long, ventral edge convex, dorsal margin straight, apically narrowing and pointed; apophysis posterior stout, rod-like, approximately 2.6 mm long, posterior quarter weakly widened, anterior end rounded; segment VIII approximately 0.8 mm long, strongly sclerotised except for ventromedial zone, posterioventral part with fine longitudinal lines; subgenital plate with broad sub-triangular sub-ostial sclerotisation, posteriorly extended into short, pointed sclerites, medial flaps delimiting ostium bursae, anterior margin with rod-like edge connected with apophysis anterioris, medially with broad and shallow sinusoid projection; apophysis anterioris rod-like, nearly length of segment VIII; colliculum small, sclerotised; ductus bursae gradually widening to weakly delimited suboval corpus bursae; entire length of ductus and corpus bursae approximately 3.1 mm; signum almost completely reduced, spiny plate.

##### Diagnosis.

*Megacraspedusargyroneurellus* is characterised by its large size, its long scale brush on segment 2 of the labial palps and by its yellowish forewings with silvery white veins and its white hindwings. It is similar to *M.lagopellus* (p 146), *M.coleophorodes* (p 148), and *M.feminensis* sp. n. (p 149). The male genitalia are similar to *M.kirgizicus* sp. n. (Figs 241–242) but differ in several characters such as the broader apex of the uncus and the comparatively stout phallus. The female genitalia differ from other species of the *M.lagopellus* species group in several characters such as the distinct sub-ostial sclerotisations.

##### Molecular data.

BIN BOLD:ACB3166 (n = 5). The intraspecific divergence of the barcode region is moderate with mean 1.2% and maximum divergence 2.2%. The distance to the nearest neighbour *M.kirgizicus* sp. n. is 7.6% (p-dist).

##### Distribution.

Armenia, Iran, Kyrgyzstan, Romania, Russia (S. Ural), Turkey. Also recorded from China (Xinjiang: Kuldja) (Rebel 1914: 277), but we have not been able to check if the record refers to this or the following species or more likely belongs to the similar *M.coleophorodes* described from China. According to Piskunov (1990: 988) also in the Caucasus.

##### Biology.

Host plant and early stages are unknown. The adults have been collected from the second half of June to early August at altitudes ranging from lowland in Europe to 2550 m in Turkey.

##### Remarks.

*Megacraspedusargyroneurellus* was described from an unspecified number of specimens (probably more than one as the wingspan is given as “19–21 mm”) collected at Sarepta, now Krasnaormeysk, in south-western Russia by H. Christoph (Staudinger 1871). A lectotype, already labelled as such by K. Sattler, is here designated in order to fix the identity of the species and conserve stability of nomenclature. Anikin and Zolotuhin (2017: 469, pl. 35, fig. 6) figured the lectotype but did not designate it following ICZN (1999) Art. 74.7.3.

This species has a distinct geographical variation: Specimens from southern areas (Armenia, Turkey and Iran) are larger on average and have lighter, slightly broader forewings with no brown area in the middle of the forewing compared with specimens from the north (Romania, Russia and Kyrgyzstan). The male genitalia from Iran are similar to those of a specimen from Ukraine (apart from a differently shaped saccus). Unfortunately we have no DNA barcode data of specimens from the south-eastern part of the distribution area, and therefore we leave it open for future studies as to whether one or two species or subspecies are involved.

### *Megacraspedusviolacellum* species group

The *M.violacellum* species group includes two species: *M.ibericus* sp. n. and *M.violacellum*.

External morphology. Segment 2 of labial palpus with short or long scale brush; segment 3 as long as or longer than segment 2. Antennal scape without or with a single, soft hair. Wingspan (males) 12–23 mm. Forewing with or without white costa and whitish veins and 2–3 black spots. Known female brachypterous.

Genitalia morphology. Male genitalia. Uncus large, sub-quadrate, apical corners rounded; gnathos hook moderately slender, about length of uncus, bent at right angles at one-quarter, apicaly pointed; anterior margin of tegumen with moderately shallow semi-oval emargination; pedunculi weakly demarcated, suboval; valva straight, stout, broadly digitate, extending to base of uncus, distal area covered with setae, without separated sacculus; posterior margin of vinculum with rounded lateral humps; saccus broadly suboval, with rounded apex, ratio maximum width to length nearly 1, posterior margin arched, with shallow medial emargination, medial part without sclerotised ridge, lateral sclerites moderately short; phallus moderately slender, stout, almost straight, suboval coecum weakly defined, digitate distal part with small dorsomedial tooth.

Female genitalia. Undescribed (see below).

Diagnosticremarks. The *M.violacellum* species group is defined by a combination of structures of the male genitalia, e.g., the large, nearly sub-square uncus, the abruptly bent gnathos hook, the broad valva, and particularly the suboval saccus and the rounded lateral hamps of the vinculum are characteristic.

The two species are externally very different and also differ in several characters in the male genitalia. Therefore, and in absence of comparative females, the common assignment to a species group is tentative, though supported by DNA barcodes.

#### Megacraspedus ibericus sp. n.

Taxon classificationAnimaliaLepidopteraGelechiidae

http://zoobank.org/047265FD-972B-4344-812B-11DABDDE1764

##### Examined material.

**Holotype** ♂, “SPAIN [prov.] Málaga 2 km NE Mijas, 560 m, 24.ix.2012 J. Tabell leg.” (ZMUC). **Paratypes.** Spain. 2 ♂, same data as holotype, but genitalia slide Tabell 5016, GU 16/1431 Huemer (TLMF, ZMUC). Portugal. 5 ♂, Serra do Larouco, Montalegre, Trás-on-Montes, 1350 m, 23.ix.2003, leg. M. F. V. Corley, genitalia slides Corley 1753, 1996 (RCMC); 1 ♂, Serra da Estréla, Vale Glaciar do Zêzere, Manteigas, Guarda, 1100 m, 10.ix.2017, J. Rosete (RCJR).

##### Description.

Adult. *Male* (Figure 125). Wingspan 12–18 mm. Segment 2 of labial palpus with long scale brush, blackish brown on outer surface, lighter brown on inner surface, white on upper surface; segment 3 longer than segment 2, narrow, white, black at tip. Antennal scape without pecten; flagellum blackish brown. Head white; thorax and tegula as forewing. Forewing light brown mottled with black especially between veins; fold yellow, dorsally edged black; a black spot in fold and two black spots at 1/2 and 3/4 in middle of wing; veins indistinctly whitish; fringes grey. Hindwing grey with lighter grey fringes.

*Female*. Unknown.

*Variation*. The species is variable in size. Worn specimens look light brownish.

*Male genitalia* (Figure 245). Uncus large, sub-quadrate, apical corners rounded; gnathos hook moderately slender, about length of uncus, subbasally bent; anterior margin of tegumen with moderately shallow emargination; valva straight, stout, broader basal part abruptly tapered to digitate distal three-fifths, extending to base of uncus, distal area covered with setae, without separated sacculus; posterior margin of vinculum with rounded lateral humps, vincular sclerites broadly suboval; saccus massive, stout, broadly suboval, tapered distal part, with rounded apex, short, ratio maximum width to length nearly 1, posterior margin arched, with shallow medial emargination, medial part without sclerotised ridge, lateral sclerites moderately short, approximately 0.8 times length of maximum width of saccus; phallus moderately slender, stout, almost straight, suboval coecum weakly defined, digitate distal part about twice length of coecum, with small dorsomedial tooth.

*Female genitalia*. Unknown.

##### Diagnosis.

*Megacraspedusibericus* sp. n. is characterised by the long segment 2 of the labial palps, the blackish brown antennae, and by the brownish forewings with three black spots and a yellow fold dorsally edged black. It resembles *M.spinophallus* sp. n. (Figure 53) and *M.alfacarellus* (Figs 49–50), but these have only two black spots in the middle of the forewings. The male genitalia are unmistakable in the shape of the uncus and the small dorsomedial tooth of the phallus.

##### Molecular data.

BIN BOLD:ACZ7298 (n = 1). The distance to the nearest neighbour *M.violacellum* is 7.5% (p-dist).

##### Distribution.

North Portugal and South Spain.

##### Biology.

Host plant and early stages are unknown. The adults have been collected in the last third of September at altitudes between 560 and 1350 m.

##### Etymology.

This species is named after its place of occurrence: the Iberian Peninsula, including Portugal and Spain. The name is a masculine adjective.

#### Megacraspedus violacellum

Taxon classificationAnimaliaLepidopteraGelechiidae

(Chrétien, 1915)

Toxoceras violacellum Chrétien, 1915: 330.
Cauloecista chretienella Dumont, 1928: 34, figs 5A, B, syn. n.
Cauloecista halfella Dumont, 1928: 36, figs 5C, D, syn. n.
Mesophleps arnaldi Turati & Krüger, 1936: 76, pl. 11, fig. 27, syn. n.

##### Examined material.

**Lectotype** ♂, *Toxocerasviolacellum*, **here designated**, “*Toxoceras violacellum*” “Gafsa 29.II.08” “TYPE” “Genitalia ♂ P. Viette Prèp. No. 2673” (MNHN) [photographs examined]. **Lectotype** ♂, *Cauloecistachretienella*, designated by Viette (1951: 57), “Holo-TYPE” “*Cauloecista chretienella*” “MUSEUM PARIS TUNISIE METLAOUI C. DUMONT 1921” “Imago 1.X.21” “type” “*Cauloecista halfella* Dum. Lepidoptera, vol. 3 1920, p. 34” genitalia slide PGCGN 8015 (MNHN) [photographs examined]. **Lectotype** ♀, *Cauloecistahalfella*, designated by Viette (1951: 57), “TYPE” “*Cauloecista halfella* Type” “MUSEUM PARIS TUNISIE METLAOUI C. DUMONT 1921” “18.9.21 ex halfa” “*Cauloecista halfella* Dum. Lepidoptera, vol. 3 1920, p. 36” (MNHN) [photographs examined].

**Lectotype** ♂, *Mesophlepsarnaldi* (without abdomen), designated by Li and Sattler (2012: 56), [Libya], “Zuetina” “20.ix.1934” “Krüger” (BMNH). **Non-type material.** Tunisia. 7 ♂, prov. Beja, 7 km W Nefza, W lake Sidi el Barrak, 30 m, 4.x.2007, leg. B. Schacht (TLMF, ZMUC, ZSM); 1 ♂, prov. Kebili, 10–20 km W Zaafrae, 10 m, 30.ix.–1.x.2007, leg. B. Schacht, genitalia slide GEL 1269 Huemer (TLMF).

##### Redescription.

Adult. *Male* (Figure 126). Wingspan 21–23 mm. Segment 2 of labial palpus with scale brush about half the length of segment 3, blackish brown on outer surface, white with black-tipped scales on inner surface, whitish on upper and lower surface; segment 3 same length as segment 2, creamcoloured. Antennal scape without pecten; flagellum dark brown, indistinctly ringed lighter. Head light grey-brown; thorax and tegula as forewing. Forewing light grey-brown; brown streaks through fold and in middle of wing until before apex; a narrow white line in middle of costa; a black dot in fold at 2/5 and one at end of cell; veins in costal and apical parts of wing finely dusted with whitish scales; fringes grey. Hindwing grey with concolorous fringes.

**Figures 127–132.**
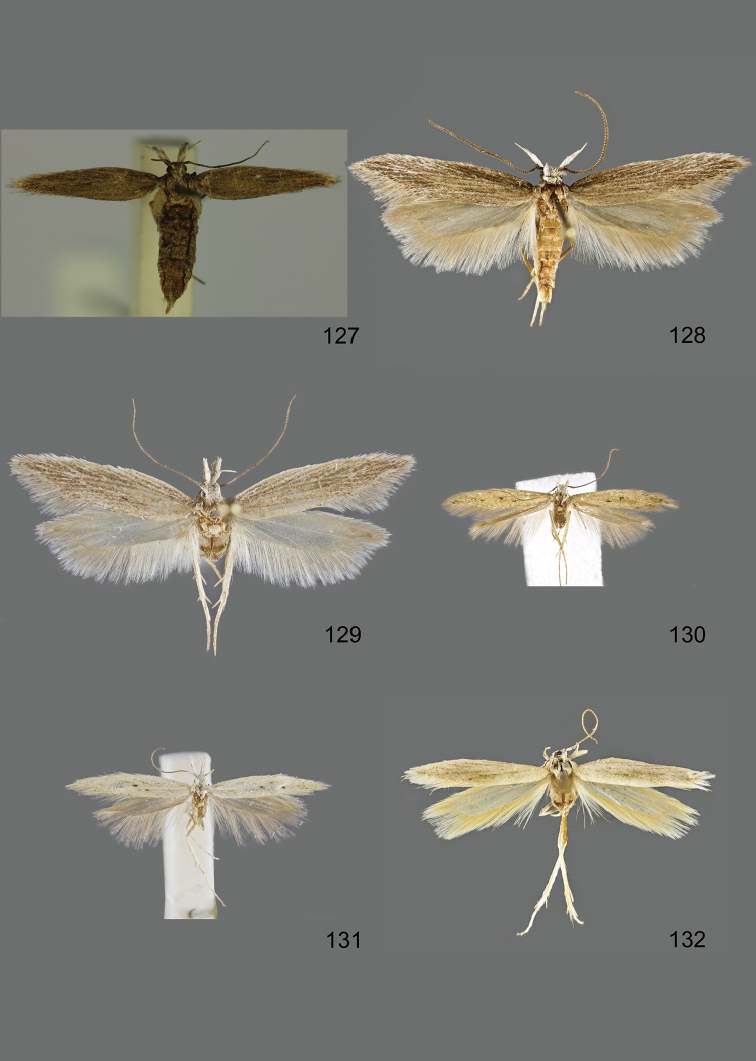
*Megacraspedus* adults in dorsal view. **127***M.violacellum* (Chrétien, 1915) – Lectotype, female (*halfella*), Tunisia (MNHN) **128***M.squalida* Meyrick, 1926 – male, Spain (NMPC) **129***M.squalida* Meyrick, 1926 – female, Spain (NMPC) **130***M.pentheres* Walsingham, 1920 – male, France (TLMF) **131***M.steineri* sp. n. – Holotype, male, Morocco (ZMUC) **132***M.gibeauxi* sp. n. – Holotype, male, Tunisia (ZMUC).

*Female* [based on original description and figure of lectotype] (Figure 127). Wingspan 18–20 mm. Forewing well developed, broadest at one-third, apical third tapered, more mottled with dark brown and black than in the male. Hindwing reduced to a short stump, transparent, grey, without scales. Otherwise similar to male.

*Variation*. The forewings vary from lighter to darker greyish brown, and there can be one or two additional small dots in the middle of the forewings. The specimen from Sbeidla is slightly more broad-winged and has the dorsal part of the forewing yellowish brown.

*Male genitalia* (Figure 246). Uncus sub-rectangular, slightly longer than broad, apical margin evenly rounded; gnathos hook moderately slender, apically pointed, slightly longer than uncus, weakly curved at about two-fifths; tegumen with broad and moderately deep anterior emargination, additional weak emargination medially, with short sclerotised sublateral ridges merged near middle of tegumen; pedunculi small, sub-triangular; valva massive, extending nearly to apex of uncus, basally widened, distal part broad, with longitudinal sclerotised ridge, apex converged, rounded; saccular area densely covered with setae, without separated sacculus; posterior margin of vinculum with deep emargination, weakly curved lateral hump, vincular sclerites broadly sub-triangular with posteriomedial ridge; saccus massive, stout, broadly suboval, tapered distal part, apically rounded, ratio maximum width to length about 1, posterior margin distinctly emarginated, with sinusoid mediolateral humps, medial part smooth, without sclerotised ridge, lateral sclerites approximately 0.6 times maximum width of saccus; phallus massive, with weakly inflated coecum, distal three-quarters stout, medially with area of numerous spinules, dorsomedially convex with broad tooth-like projection, apically rounded.

*Female genitalia*. Undescribed. Unfortunately we did not obtain permission to examine the genitalia of the only available female.

##### Diagnosis.

*Megacraspedusviolacellum* is characterised by its large size and its light grey-brown forewings with two brown streaks and two black dots. It resembles *M.squalida* (Figs 128–129) and *M.peyerimhoffi* (Figs 145–146), but these species are without brown streaks in the forewings, having only obsolete black dots. The male genitalia are similar overall to *M.armatophallus* sp. n. (Figure 266) but unmistakable in *Megacraspedus* in the unique shape and sclerotisations of the phallus.

##### Molecular data.

BIN BOLD:ADI7610 (n = 1). The distance to the nearest neighbour *M.ibericus* sp. n. is 7.45% (p-dist).

##### Distribution.

Libya, Tunisia.

##### Biology.

The larva and pupa are described in details by Dumont (1928: 35–36). The larva feeds within a stem of *Macrochloatenacissima* (Loefl. ex L.) (Poaceae) until March. Then it builds a semi-rigid chamber, composed of extremely fine, pure white silk, in which it lives a and grows slowly until the end of August/beginning of September when it pupates. Larvae collected in Tunisia were reared in Paris during the second half of September. The adults have been collected from the middle of September to early October. The label data “II”, referring to February, on a type specimen is probably the date the larva was collected.

##### Remarks.

*Toxocerasviolacellum* was described from an unstated number of specimens collected at Gafsa, Tunisia in November (Chrétien 1915: 330). We have been able to examine photographs of a syntype, labelled as type, from MNHN including the genitalia slide. This specimen is here designated as the lectotype in order to fix the identity of the species and conserve stability of nomenclature.

*Cauloecistachretienella* was described from an unstated number of specimens (“♂♂, ♀♀”) bred from larvae collected by Al-Mitlawi [“Metlaoui”], central Tunisia (Dumont 1928). A lectotype was designated by Viette (1951: 57). We have been able to examine photographs of this male specimen, incorrectly labelled as holotype, from MNHN. It fully corresponds with *M.violacellum* both externally and in genitalia and we therefore consider *M.chretienella* to be a junior synonym of *M.violacellum* (syn. n.). However, the female labelled allotype belongs to *M.squalida*.

*Cauloecistahalfella* was described from two females bred from larvae collected by Al-Mitlawi [“Metlaoui”], in central Tunisia (Dumont 1928). A lectotype was designated by Viette (1951: 57). We have been able to examine photographs of it which correspond with males of *M.violacellum* in wing markings and colour. Furthermore type-material of *Cauloecistachretienella* (=*M.violacellum*) and *C.halfella* was described from the same locality and bred from the same host plants without any remarks as to differences in the biology. We therefore consider *M.halfella* to be a further junior synonym of *M.violacellum* (syn. n.).

*Mesophlepsarnaldi* was described from two specimens collected by G. Krüger at Az Zuwaytīnah (‘Zuetina’), Libya (Turati and Krüger 1936: 76). A lectotype was published by Li and Sattler (2012: 56), who also transferred *arnaldi* from *Mesophleps* to *Megacraspedus*. These authors also suggested the synonymy of *M.arnaldi* with *M.violacellum* without, however, formally synonymising them. We examined the lectotype of *M.arnaldi* and consider it to be a junior synonym of *M.violacellum* (syn. n.).

A single male specimen from Sbeidla, Tunisia, coll. C. S. Larsen (ZMUC) was dissected by D Povolný. However, whereas the adult matches a faded *M.violacellum* the (remounted) genitalia correspond with *M.glaberipalpus* sp. n. which belongs to a different species group. We believe that a mix up of slides has probably happened during preparation or labelling.

### *Megacraspedussqualida* species group

The *M.squalida* species group includes one species: *M.squalida*.

External morphology. See species description.

Genitalia morphology. Male genitalia. See species description.

Female genitalia. See species description.

Diagnosticremarks. The *M.squalida* species group is defined by several unique and highly diagnostic structures in the male genitalia, in particular the shape of the valva with strong saccular bristles and the curved thorn-like process near base of valva. The female genitalia are characterised by the membranous ostium bursae, without delimiting sclerotisations, but females are unknown in several species groups.

#### Megacraspedus squalida

Taxon classificationAnimaliaLepidopteraGelechiidae

Meyrick, 1926

Megacraspedus squalida Meyrick, 1926: 272.
Megacraspedus escalerellus Schmidt, 1941: 38, pl. 2, fig. 5, syn. n.

##### Examined material.

**Holotype** ♂, *Megacraspedusescalerellus*, [Spain] “Spain Montarco Madrid ix.1920 F. Escalera” “Genitalia slide Mus. Madrid 53788” (MNCN). **Non-type material.** Spain. 1 ♂, 1 ♀, prov. Alicante, Alcoj, Font Roja, W El Menejador, 1300 m, 4.ix.2005, leg. P. Huemer, genitalia slide GEL 1193 ♂ Huemer (TLMF); 1 ♂, prov. Alicante, Sierra de Orcheta, Finestrat env., 28.ix.2005, leg. J. Šumpich; 2 ♂, 1 ♀, prov. Almería, Tabernas env., Rambla de Tabernas, 18–19.x.2009, leg. J. Šumpich, genitalia slide GU 16/1428 ♀ Huemer (all NMPC); 1 ♂, prov. Almería, Tabernas, ’Mini Hollywood’, 230 m, 14–15.x.1992, leg. M. Fibiger; 1 ♂, 2 ♀, prov. Almería, 5 km N Carboneras, 10 m, 16.x.1992, leg. M. Fibiger, genitalia slide 6488 Hendriksen; 1 ♀, Almería, 7 km of Cabo de Gata, 0 m, 22.x.2003, leg. J. Viehmann; 8 ♂, prov. Almería, El Pozo del Esparto, 20 m, 1.xi.2005, leg. B. Skule & P. Skou; 5 ♂, prov. Almería, Sierra Alhamilla, 9 km above Turrillas, Colativi, 1000–1350 m, 1.ix.2001, leg. C. Hviid & B. Skule; 1 ♂, prov. Almería 6 km S Serón, at A 1178, 1300 m, 16.ix.2009, leg. P. Skou; 1 ♀, prov. Almería, 3 km SW Pulpi, 300 m, 5.x.2015, leg. J. Viehmann (all ZMUC); 1 ♂, prov. Almería, Rambla Tabernas, 100 m, 8.ix.2007, leg. J. Viehmann; 1 ♀, same data, but 6.x.2016; 1 ♀, same data, but 24.x.2016; 1 ♀, prov. Almería, Sierra de los Filabres, Gergal, 1800 m, 9.ix.2007, leg. J. Viehmann (all RCWS); 1 ♂, prov. Granada, Sierra Nevada, Camino de Valeta, 2250 m, 8.ix.1985, leg. E. Traugott-Olsen; 1 ♂, prov. Granada, Camino Baza-Benamaurel, 13.x.1988, leg. E. Traugott-Olsen; 2 ♂, prov. Granada, Sierra del Chaparral, 10 km W Lenteji, 900 m, 26.viii–15.ix.2005, leg. G. Jeppesen; 1 ♂, 1 ♀, same data, but 30.viii.–4.ix.2009; 10 ♂, 1 ♀, prov. Granada, Sierra de Los Guájares, 1160–1220 m, 26.viii–15.ix.2005, leg. G. Jeppesen; 2 ♂, prov. Granada, Moscaril, 500 m, 28.viii–9.ix.2004, leg. G. Jeppesen; 1 ♂, same data, but 26.viii–15.ix.2005, leg. G. Jeppesen; 1 ♂, prov. Granada, 22 km above Otivar, Granja Escula, Huerto Alegre, 1250 m, 30.viii.2001, leg. B. Skule (all ZMUC); 1 ♂, prov. Lleida, Castelldans, Les Garrigues, 353 m, 29.x.2010, leg. P. De-Gregorio & M. Bravo (RCER); 1 ♂, prov. Madrid, Camporeal, 640 m, 19.ix.1979, leg. A. Vives, genitalia slide 1226 Vives; 1 ♂, prov. Madrid, Perales de Tarjuna, 2.x.1995, leg. J. Wolschrijn; 1 ♂, prov. Málaga, Marbella, Casa y Campo, 100 m, 11.ix.1976, leg. E. Traugott-Olsen, genitalia slide 5437 Traugott-Olsen; 1 ♂, prov. Málaga, Sierra de Marbella, El Mirador, 700 m, 4.ix.1977, leg. E. Traugott-Olsen; 1 ♂, prov. Málaga, Camino de Rhonda, Urb. Madronal, Loma del Colmenas, 500 m, 3.ix.1988, leg. E. Traugott-Olsen; 3 ♂, prov. Málaga, 2 km W Sedella, La Rahige, 650 m, 18.ix.2009, leg. P. Skou (all ZMUC); 1 ♀, prov. Valencia, Santa Pola, Playa del Pinet, 5 m, 5.ix.2005, leg. P. Huemer (TLMF). Tunisia. 1 ♂, prov. Kasserine, 20 km SW Kasserine, S NP Jebel Chambi, 900 m, 2.-3.x.2007, leg. B. Schacht, genitalia slide 5373 Stübner (ZSM); 5 ♂, 2 ♀, prov. Beja, 7 km W Nefza, W lake Sidi el Barrak, 30 m, 4.x.2007, leg. B. Schacht (TLMF, ZSM); 2 ♂, prov. Nabeul, Umg. Soliman, Boj-Cedria, 0–20 m, 5.x.2007, leg. B. Schacht, genitalia slide 5371 Stübner (ZSM).

##### Redescription.

Adult. *Male* (Figure 128). Wingspan 16–21 mm. Labial palpus comparatively strong; segment 2 with broad scale brush, dark brown on outer surface, white mottled with brown on inner and lower surface, white on upper surface; segment 3 white, slightly mottled with dark brown. Antennae thickened, black. Head light grey, dark brown towards eye; thorax and tegula as forewing. Forewing grey mottled with black, especially near base; veins and middle of costa indistinctly whitish; indistinct black dots in fold and at end of cell; fringes grey. Hindwing grey with light grey fringes.

*Female* (Figure 129). Wingspan 17–22 mm. Antennae thickened, ringed lighter and darker brown. Forewings slightly broader than in male, with more distinct lighter veins. Otherwise similar to male.

*Variation*. There is only slight variation. The black dots in fold and at end of the cell are not always visible.

*Male genitalia* (Figure 247). Uncus broad, distally weakly expanded, about same length as width, maximum width at about three-quarters length, apex strongly arched; gnathos hook about length of uncus, moderately slender, evenly curved, with apical point; anterior margin of tegumen with broadly convex excavation, pedunculi moderately large, rounded; valva broad and stout, exceeding base of gnathos, distal part tapered, apex rounded, saccular area hump like, with several long and stiff setae; flap-like sclerite with long, strongly sclerotised, curved thorn-like process near proximal base of valva; posterior margin of vinculum without distinct medial emargination; saccus broadly U-shaped, shorter than valva, posterior margin nearly straight, without medial emargination, medial part with short sclerotised ridge in posterior quarter, lateral sclerites about two-thirds length of maximum width of saccus; phallus with small coecum, distal three-quarter slender, rod-like, with distinct sclerotised tooth in middle of phallus on dorsal surface.

*Female genitalia* (Figure 298). Papilla analis moderately sclerotised, large, approximately 1.4 mm long, narrowing towards rounded apex, ventral edge convex, dorsal margin nearly straight; apophysis posterior rod-like, approximately 1.8 mm long, psoteriorly weakly widened at about one-fifth, anterior end weakly inflated; segment VIII approximately 0.6 mm long, smoothly sclerotised except from membranous anterioventral zone, oblique anteriordorsal edge strongly sclerotised ridge; subgenital plate reduced to lateral sclerotised zones, ostium bursae membranous, without delimiting sclerotisations; apophysis anterior rod-like, slightly longer than segment VIII; colliculum small, sclerotised; ductus bursae gradually widening to weakly delimited suboval corpus bursae; entire length of ductus and corpus bursae approximately 3.1 mm; signum moderately small, suboval, spiny plate.

##### Diagnosis.

*Megacraspedussqualida* is characterised by its relatively large size and its blackish grey forewings with indistinctly lighter veins. The males, moreover, are recognizable by the thickened, black antennae. The species is somewhat similar to *M.peyerimhoffi* (p 181) and *M.violacellum* (p 157). The flap-like sclerite with thorn-like process in the male genitalia is unique in *Megacraspedus*. The female genitalia are unique in *Megacraspedus* with several diagnostic features such as the membranous area of the ostium bursae.

##### Molecular data.

BIN BOLD:ACM0982 (n = 6). The intraspecific divergence of the barcode region is low with mean 0.5% and maximum divergence of 1%. The distance to the nearest neighbour *M.skulei* sp. n. is 9.3% (p-dist).

##### Distribution.

Spain, Tunisia.

##### Biology.

The larva feeds within a stem of *Macrochloatenacissima* (Loefl. ex L.) (Poaceae) (Dumont 1928), see Remarks. The adults have been collected from late August to the middle of October at altitudes from sea level to 2250 m.

##### Remarks.

*Megacraspedussqualida* was described from one female collected by Ribbe in the Sierra Nevada, Spain (Meyrick 1926). The holotype and its genitalia were figured by Clarke (1969: 229, fig 3a, b). *Megacraspedusescalerellus* was described from one specimen collected in Montarco, province of Madrid, Spain on 20^th^ September 1920 (Schmidt 1941). The holotype fully corresponds with *M.squalida* and we thus consider *M.escalerellus* to be a junior synonym of the former (syn. n.).

The ‘allotype’ of *Cauloecistachretienella* Dumont, 1928 has proved to belong to *M.squalida* (see remarks on *M.violacellum*). It was bred from *Macrochloatenacissima* (Loefl. ex L.) (Dumont 1928).

### *Megacraspeduspentheres* species group

The *M.pentheres* species group includes eight species: *M.pentheres*, *M.steineri* sp. n., *M.gibeauxi* sp. n., *M.multipunctellus* sp. n., *M.teriolensis* sp. n., *M.korabicus* sp. n., *M.quadristictus*, and *M.eburnellus*.

External morphology. Segment 2 of labial palpus with short or long scale brush; segment 3 as long as or longer than segment 2. Antennal scape without or with one to several hairs. Wingspan (males) 10–19 mm. Forewing with 0–4 black spots, in a few species with whitish or blackish veins, but without white costa. Known female slightly to moderately brachypterous.

Genitalia morphology. Male genitalia. Uncus moderately small, basally constricted, suboval; gnathos hook stout, short, medially widened, with ventromedial ridge, weakly curved; anterior margin with broad and shallow emargination, additional small emargination medially; valva long, nearly extending to apex of uncus; sacculus usually well developed, short and digitate, rarely reduced; saccus broadly V- to U-shaped, with or without short medial ridge; phallus stout, weakly separated coecum, distal part short and tapered, with sclerotised dorsal ridge, ductus ejaculatorius with long interior sclerotisation.

Female genitalia. Papilla analis large, apex broadly rounded; apophysis posterior rod-like, short, approximately 1.5 times length of papilla analis; segment VIII short, posteriolaterally sclerotised, medially membranous; subgenital plate with sub-triangular, subostial sclerotisation, broad and shallow projection anteriorly, sub-medial sclerites shortly pointed; apophysis anterior rod-like, about length of segment VIII; colliculum short, weakly sclerotised; signum small to medium-sized, spiny plate.

Diagnosticremarks. The *M.pentheres* species group is defined by structures of the male genitalia, in particular the short and suboval, basally constricted uncus, the short and medially widened gnathos hook, the valva with usually separated sacculus, and the distally narrowing phallus with a sclerotised, denticulate ridge. The female genitalia are characterised by the comparatively large papilla analis, and the short apophysis posterior and anterior as well as segment VIII, but females are unknown in several other species groups.

#### Megacraspedus pentheres

Taxon classificationAnimaliaLepidopteraGelechiidae

Walsingham, 1920

Megacraspedus pentheres Walsingham, 1920: 10.

##### Examined material.

**Paratype** ♂, “Basses Alpes S FRANCE 22.viii.1913 Wlsm. 1913-390 95935” “Paratype” “*Megacraspedus pentheres* Wlsm. PARATYPE”, genitalia slide 33662 (BMNH). **Non-type material.** France. 1 ♂, Dep. Alpes-Maritimes, Saint-Sauveur-sur-Tinée, 510 m, 4.viii.2000, leg. J. Nel, genitalia slide 11757 Nel (TLMF).

##### Description.

Adult. *Male* (Figure 130). Wingspan 11.5 mm. Segment 2 of labial palpus with scale brush longer than segment 3, creamy on inner and dorsal surface, outer surface creamy brown, covered with mid-brown tipped creamy scales; segment 3 shorter than segment 2, cream white with darker mottling. Antennal scape with pecten of about five hairs; flagellum light brown ringed with dark grey. Head, thorax and tegula cream-white mottled with light brown scales; thorax and tegula as forewing. Forewing light yellow-brown mottled with brown- and black-tipped scales, especially in apical part; costa blackish near base; a black dot in fold and two in middle of wing and at end of cell; some black scales along termen; fringes greyish. Hindwing grey with light grey fringes.

*Female*. Unknown.

*Variation*. The examined specimens show only slight variation. One specimen has a fourth black dot in the forewing. The pecten is easily lost as demonstrated from a specimen with 3 hairs on one and 5 hairs on the other side.

*Male genitalia* (Figure 248). Uncus moderately small, basally contricted, suboval, slightly shorter than maximum sub-basal width, apex evenly rounded; gnathos hook stout, about length of uncus, weakly curved, medially widened, with ventromedial ridge, apically pointed; tegumen with medially confluent sclerotised anterior ridges, anterior margin with broad and moderately shallow emargination, additional small shallow excavation medially; pedunculi small; valva long, extending to about apex of uncus, slender digitate, distally weakly curved with rounded apex; saccular area with few microtrichia, without separate sacculus; posterior margin of vinculum with shallow medial emargination, weakly rounded lateral humps, vincular sclerites oblonge, moderately slender; saccus broadly sub-triangular, with broadly V-shaped outer edge, evenly tapered to pointed apex, moderately short, ratio maximum width to length about 1, posterior margin shallowly arched, with indistinct medial emargination, medial part smooth, without sclerotised ridge, lateral sclerites short, approximately 0.6 times length of maximum width of saccus; phallus with bulbous coecum, distal three-fifths stout, straight, sclerotised sub-apical ridge with about five small teeth.

*Female genitalia*. Unknown.

##### Diagnosis.

*Megacraspeduspentheres* is characterised by light yellow-brown forewings with three black dots and black scales along termen. It closely resembles *M.tristictus* (Figure 48) (with which it shares the light yellowish colour of the forewings), but the dot at the end of the cell is largely reduced and there are several small dark spots in the terminal area, which are absent in *M.tristictus*. It is furthermore similar to *M.neli* sp. n. (p 59). The male genitalia differ from all other species of the *M.pentheres* species group in the absence of a sacculus.

##### Molecular data.

Not available, barcoding failed.

##### Distribution.

France (Dep. Alpes-de-Haute-Provence).

##### Biology.

Host plant and early stages are unknown. The type-series was collected in the middle of August at an altitude of ca. 900 m; a further specimen dates from early August.

##### Remarks.

*Megacraspeduspentheres* was described from seven males collected by Walsingham at Annot, Alpes-de-Haute-Provence, France (Walsingham 1920). The label dates of the examined paratype disagree with the dates published in the original decription, as ’9–12.VIII.1913’.

#### Megacraspedus steineri sp. n.

Taxon classificationAnimaliaLepidopteraGelechiidae

http://zoobank.org/4F730A8A-D63F-4CB8-93B8-53EE677A4FAF

##### Examined material.

**Holotype** ♂, “Maroc, Haut Atlas Toubkal Massif Oukaïmeden area piste Oukaïmeden-Tizi-n-Eddi 31°10'N, 7°50'W 3.viii.2008, 2815 m A. Steiner leg.” “GU 16/1421 ♂ Huemer” “DNA Barcode TLMF Lep 19846” (ZMUC).

##### Description.

Adult. *Male* (Figure 131). Wingspan 13 mm. Segment 2 of labial palpus with long scale brush, blackish brown, white on upper surface; segment 3 shorter than scale brush of segment 2, white with black tip. Antennal scape with pecten of a few hairs; flagellum indistinctly ringed with lighter and darker brown. Head cream-white; thorax and tegula as forewing. Forewing light yellow mottled with some blackish brown scales; black spots in fold and at 1/2 and 3/4 in middle of wing; fringes yellowish grey. Hindwing grey with grey fringes.

*Female*. Unknown.

*Variation*. Unknown.

*Male genitalia* (Figure 249). Uncus moderately small, basally constricted, suboval, about same length as width, apex broadly rounded; gnathos hook stout, about length of uncus, evenly curved, lateromedially widened, apically pointed; tegumen with medially confluent sclerotised anterior ridges, anterior margin with broad and moderately shallow emargination, additional small shallow excavation medially; pedunculi small; valva long, extending to about tip of uncus, broader at base, distal part slender, apically weakly curved with rounded apex; sacculus well developed, short, slender digitate; posterior margin of vinculum with shallow medial emargination, weakly rounded lateral humps, vincular sclerites elongated, slender; saccus broadly sub-triangular, with broadly V-shaped outer edge, evenly tapered to pointed apex, moderately short, ratio maximum width to length about 1, posterior margin broadly arched, with broad and shallow medial emargination, medial part with sclerotised ridge, extended beyond middle of saccus, lateral sclerites short, approximately 0.6 times length of maximum width of saccus; phallus with bulbous coecum, distal two-thirds stout, weakly curved, with sclerotised dorsal and ventral ridge.

*Female genitalia*. Unknown.

##### Diagnosis.

*Megacraspedussteineri* sp. n. is characterised by its light yellow forewing with three black spots. It has moreover a long scale brush at segment 2 of the labial palpi. The species is most similar to *M.glaberipalpus* sp. n. (p 128). The male genitalia are very similar to some other species of the *M.pentheres* species group with only small differences e.g., such as the medial ridge of the saccus or the absence of spines on the phallus.

##### Molecular data.

Not available, barcoding failed.

##### Distribution.

Morocco (High Atlas).

##### Biology.

Host plant and early stages are unknown. The holotype was collected in early August at an altitude of ca. 2800 m.

##### Etymology.

The species name (a noun in the genitive case) is dedicated to Axel Steiner, Germany, who collected the holotype.

##### Remarks

. Despite the limited diagnostic genitalia characters and the absence of molecular data, the separate species status is supported by the colour and pattern of the forewing.

#### Megacraspedus gibeauxi sp. n.

Taxon classificationAnimaliaLepidopteraGelechiidae

http://zoobank.org/FB931B7D-1363-4743-9B6D-15D5567BA339

##### Examined material.

**Holotype** ♂, “Tozeur [south-western] TUNESIA 15.–29.x.1988 P. Sunesen col. K. Larsen” “GU 16/1442 ♂ P. Huemer” (ZMUC). **Paratypes.** 2 ♂, Algeria, Biskra, 13.x.1911, genitalia slide 8046 Gibeaux (MNHN).

##### Description.

Adult. *Male* (Figure 132). Wingspan 15 mm. Segment 2 of labial palpus with scale brush of about same length as segment 3, white mottled with light yellow brown on outer and inner surface; segment 3 white with black tip. Antennal scape with pecten of several rather long hairs; flagellum whitish, ringed with light brown especially in apical half. Head cream-white; thorax and tegula as forewing. Forewing whitish, mottled with grey-brown especially in fold, along veins and in apical part; fringes whitish grey. Hindwing whitish grey with light yellowish fringes.

*Female*. Unknown.

*Variation*. The intensity of the grey-brown scales is variable.

*Male genitalia* (Figure 250). Uncus moderately small, basally constricted, suboval, about same length as width, sub-basally widend, apex broadly rounded; gnathos hook stout, about length of uncus, evenly curved, lateromedially widened, apically pointed; tegumen smooth, with weakly sclerotised medially confluent anterior ridges, anterior margin with broad and moderately shallow emargination, additional small and shallow excavation medially; pedunculi small; valva long, extending to about tip of uncus, broader at base, distal part slender, apically weakly curved with rounded apex; sacculus well developed, short, slender digitate; posterior margin of vinculum with shallow medial emargination, distinctly rounded lateral humps, vincular sclerites moderately broad; saccus broadly sub-triangular, with broadly V-shaped outer edge, evenly tapered to pointed apex, moderately short, ratio maximum width to length about 1, posterior margin broadly arched, with broad and shallow medial emargination, medial part smooth, without sclerotised ridge, lateral sclerites short, approximately 0.6 times length of maximum width of saccus; phallus with bulbous coecum, distal two-thirds stout, weakly curved, with sclerotised dorsal and ventral ridge and few microspines in distal part.

*Female genitalia*. Unknown.

##### Diagnosis.

*Megacraspedusgibeauxi* sp. n. is characterised by its whitish forewing with grey-brown scales along the veins and by the absence of black dots on the forewing, and by the antennal scape having a pecten of several rather long hairs. The male genitalia are very similar to other species of the *M.pentheres* species group and differ only in subtle characters, in particular the distinct humps of the vinculum and the comparatively slender phallus.

##### Molecular data.

Not available, barcoding failed.

##### Distribution.

Algeria, Tunisia.

##### Biology.

Host plant and early stages are unknown. The few specimens known were collected in October. The locality of the holotype is an oasis at the northern border of the Sahara.

##### Etymology.

The species (a noun in the genitive case) name is dedicated to Christian Gibeaux, France, in recognition of his invaluable help with dissection and photographs of type material from MNHN.

##### Remarks.

The two paratypes from Algeria were found in the collection of P. Chrétien in the MHNP. They are mounted on the same polyporus strip and bear additional labels “?*Paltodora pectinatella* 4923′” (in Chrétien’s handwriting) and “TYPE”. Chrétien apparently considered the specimens to belong to an undescribed species, but a description was not published.

#### Megacraspedus multipunctellus sp. n.

Taxon classificationAnimaliaLepidopteraGelechiidae

http://zoobank.org/893A0DCF-CF3E-48C0-88FE-8C0DF3486147

##### Examined material.

**Holotype** ♂, “TURKEI Prov. Konya, 36°57'N 33°17'E Setravul Geçidi 1550 m 28 km S Karaman, 3.09.1983 LF leg. Werner Wolf” “GU 16/1455 ♂ Huemer” (ECKU). **Paratype.** Turkey. 1 ♂, same data as holotype, but without genitalia slide (ZMUC).

##### Description.

**Figures 133–138.**
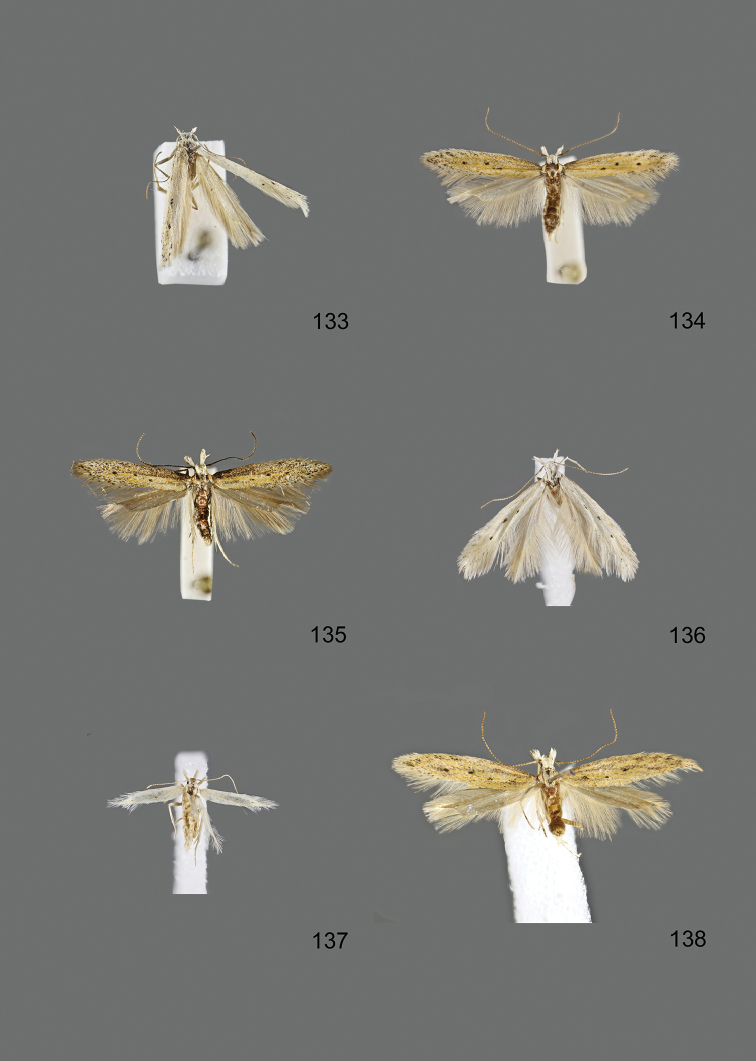
*Megacraspedus* adults in dorsal view. **133***M.multipunctellus* sp. n. – Holotype, male, Turkey (ECKU) **134***M.teriolensis* sp. n. – Paratype, male, Croatia (RCZT) **135***M.teriolensis* sp. n. – Paratype, male, Slovenia (NMPC) **136***M.korabicus* sp. n. – Holotype, male, Macedonia (TLMF) **137***M.korabicus* sp. n. – Paratype, female, Macedonia (TLMF) **138***M.quadristictus* Lhomme, 1946 – male, France (TLMF).

Adult. *Male* (Figure 133). Wingspan 14 mm. Segment 2 of labial palpus with moderately long scale brush, brown on outer and lower surface, white mottled with brown on inner surface, white on upper surface; segment 3 shorter than segment 2, whitish brown. Antennal scape white, with pecten of several hairs; flagellum brown, indistinctly ringed lighter. Head, thorax and tegula white. Forewing cream-white, mottled with scattered black-tipped scales, especially in apical part; two black dots in fold and one at end of cell; black-tipped scales along termen; fringes white. Hindwing whitish grey, with white fringes.

*Female*. Unknown.

*Variation*. None.

*Male genitalia* (Figure 251). Uncus moderately small, basally constricted, suboval, slightly shorter than broad, sub-basally widened, distinctly tapered to weakly rounded apex; gnathos hook stout, about length of uncus, evenly curved, lateromedially widened, apically pointed; tegumen smooth, with longitudinal anteriomedial ridge, anterior margin with broad and moderately shallow emargination, additional small and shallow excavation medially; pedunculi moderately small; valva long, extending to about tip of uncus, broader at base, distal part slender, apically weakly curved with rounded apex; sacculus well developed, short, slender digitate; posterior margin of vinculum with shallow medial emargination, weakly developed lateral humps, vincular sclerites irregularly oblong; saccus broadly sub-triangular, with broadly V-shaped outer edge, evenly tapered to pointed apex, moderately short, ratio maximum width to length about 1, posterior margin broadly arched, nearly sinusoid, with broad and shallow medial emargination, medial part smooth, without sclerotised ridge, lateral sclerites short, about half length of maximum width of saccus; phallus with bulbous coecum, distal two-thirds stout, medially weakly curved, with sclerotised dorsal and ventral ridge, no additional sclerites.

*Female genitalia*. Unknown.

##### Diagnosis.

*Megacraspedusmultipunctellus* sp. n. is characterised by its short segment 3 of the labial palpus, the antennal scape with several hairs, and by its cream-white forewings with scattered black scales and dots. It is most similar to *M.cerussatellus* (Figure 107), but that species is smaller and has pure white forewings. The male genitalia are very similar to *M.gibeauxi* sp. n. (Figure 250) and other species of the *M.pentheres* species group and differ only in subtle characters such as the shape of the uncus and the shape and sclerotisations of the phallus.

##### Molecular data.

Not available, barcoding failed.

##### Distribution.

Central Turkey.

##### Biology.

Host plant and early stages are unknown. The small type-series was collected in early September at an altitude of 1550 m.

##### Etymology.

The species name indicates the characteristic wing pattern and is derived from a combination of the Latin words *multus* (meaning many) and *punctus* (meaning dot), and the diminutive suffix –*ellus*. The name is a noun in apposition.

#### Megacraspedus teriolensis sp. n.

Taxon classificationAnimaliaLepidopteraGelechiidae

http://zoobank.org/D59DCC27-04A8-4729-BC8F-0CF8EAA0A2D4

##### Examined material.

**Holotype** ♂, [Italy, Provinz Bozen-Südtirol] “ITALIA sept., Südtirol Montiggl, Kl. Priol 600 m, 30.8.1995 leg. Huemer” “BC TLMF Lep 05198” “P. Huemer GEL 1191 ♂” (TLMF). **Paratypes.** Croatia. 1 ♂, Krk, Misučaynica, 13.viii.1977, leg. G. Baldizzone (ZMUC); 1 ♂, Zaostrog, 6.ix.2002, leg. Z. Tokár, genitalia slide 7957 Z. Tokár; 4 ♂, Gospič, 15.ix.2007, leg. Z. Tokár, genitalia slide 12182 Z. Tokár; 2 ♂, Bilišane, 13.ix.2007, leg. Z. Tokár; 1 ♂, 5 km SE Pirovac, 24.vi.2006, leg. Z. Tokár (all RCZT); 5 ♂, South Velebit, 26.viii.2011, leg. I. Richter; 1 ♂, same data, but 2.ix.2011 (NMPC, RCIR); 2 ♂, Kekezi, 1.ix.2011, leg. I. Richter (RCIR, ZMUC); 1 ♂, Pag, Novalja-Potočnia, 26–30.viii.2001, leg. J. Šumpich; 1 ♂, same data, but 2.ix.2009 (NMPC). Greece. 1 ♂, Parnassos, 1900 m, 4.viii.2005, leg. Viehmann (RCWS); 3 ♂, Epirus, Parga, 0 m, 12.viii.2005, leg. Viehmann, genitalia slide 5330 Karsholt (RCWS, ZMUC); 4 ♂, Ioánina, above Monodendri, Vicos Gorge, 1300 m, 25.vii.2008, leg. P. Skou, genitalia slide 16/1469 P. Huemer (ZMUC); 1 ♂, Peloponnesos, 15 km NW Kalavrita, 900 m, 26.ix.2005, leg. W. Schmitz, genitalia slide 5331 Karsholt (RCWS). Italy. 13 ♂, Südtirol, Vintschgau, Laas, 900 m, 18.viii.2011, leg. J. Skyva; 8 ♂, same data, but 31.viii.2008; 4 ♂, same data, but 600 m, 3.viii.2003, leg. J. Šumpich; 1 ♂, same data, but 10.viii.2004 (all NMPC); 1 ♂, Südtirol, Vintschgau, Naturns, 500 m, 23.vii.1983, leg. B. Skule & P. Skou (ZMUC); 3 ♂, same data, but 1–5.x.1983 (ZMUC); 1 ♂, same data, but 17.viii.1996, leg. P. Skou, genitalia slide 2437 Hendriksen (ZMUC); 1 ♂, prov. Pordenone, 7 km SSW Vivaro, Magredi S, 110 m, 11.ix.2009, leg. P. Huemer, genitalia slide GEL 1221 Huemer (TLMF) 1 ♂, prov. Verona, M. Lessini, Marano, San Rocco, 500 m, 31.viii.1974, leg. P. Triberti; 1 ♂, prov. Verona, M. Lessini, Trezzolano, 400 m, 1.ix.1978, leg. P. Triberti; 1 ♂, prov. Verona, M. Lessini, Monte, 400 m, 9.viii.1985, leg. P. Triberti; 1 ♂, prov. Verona, M. Lessini, M. Tosato, 500 m, 30.viii.1990, leg. P. Triberti (RCPT, ZMUC). Slovenia. 1 ♂, Petrinjski kras, Crnotice-Praproce, 420 m, 2.ix.2011, leg. H. Habeler; 2 ♂, Karst, Presnica, 400 m, 9.ix.2002, leg. H. Deutsch (all TLMF); 2 ♂, Nova Gorica, Sabotin, 450 m, 26.viii.2006, leg. J. Skyva; 2 ♂, Podgorje, Debeli Hrib, 24.viii.2006, leg. J. Skyva; 10 ♂, same data, but 19.viii.2007, leg. J. Skyva; 11 ♂, same data, but 22.viii.2008, leg. J. Skyva; 6 ♂, same data, but 25.viii.2011, leg. J. Skyva (all NMPC); 9 ♂, Podgorje, Crnotice, 450 m, 22.viii.2008, leg. J. Liška (NMPC).

##### Description.

Adult. *Male* (Figs 134–135). Wingspan 10–13 mm. Segment 2 of labial palpus with scale brush as long as segment 3, blackish brown on outer surface, white mottled with brown on inner surface, white on lower and upper surface; segment 3 white. Antennal scape with pecten of 1–2 fine hairs; flagellum light brown ringed with black. Head cream-white, thorax and tegula light yellow, the latter with brown base. Forewing light grey-brown from white light brown-tipped scales; a yellow streak in fold and some yellow along dorsum; two elongate black dots in fold and two black dots in middle of wing and at end of cell; some black scales along termen; fringes grey. Hindwing grey with concolorous fringes.

*Female*. Unknown.

*Variation*. The forewing colour varies from yellowish (especially in the dorsal half) to light grey- brown with yellow only in the fold and along dorsum. Two examined specimens from Croatia have the forewing blackish brown with clear yellow longitudial streaks.

*Male genitalia* (Figs 252–253). Uncus moderately small, basally constricted, irregularly rounded, slightly shorter than broad, sub-basally widened with weak lateral bulge, distally tapered to broadly rounded apex; gnathos hook massive, stout, slightly exceeding length of uncus, weakly curved, lateromedially widened, apically pointed; tegumen smooth, with weakly developed merged ridges anteromedially, anterior margin with broad and moderately shallow emargination, additional small and shallow excavation medially; pedunculi moderately small with transverse ridge; valva long, extending slightly beyond tip of uncus, broader at base, distal part slender, apically weakly curved with rounded apex; sacculus well developed, short, slender digitate; posterior margin of vinculum with shallow medial emargination, weakly developed lateral humps, vincular sclerites irregularly oblong with broad base; saccus broad, with weakly convex outer edge, distally tapered to pointed apex, moderately short, ratio maximum width to length about 1, posterior margin broadly arched, nearly sinusoid, with broad and shallow medial emargination, medial part smooth, with indistinct short sclerotised ridge, lateral sclerites short, approximately 0.6 times length of maximum width of saccus; phallus gradually tapered, with weakly defined bulbous coecum, distal two-thirds stout, straight, with broadly sclerotised zone dorsally and slender sclerotised ventral ridge, subapically with indistinct tooth.

*Female genitalia*. Unknown.

##### Diagnosis.

*Megacraspedusteriolensis* sp. n. is characterised by its light grey-brown forewings with four black dots, and a yellow streak in the fold. It is very similar to *M.quadristictus* (p 172). The male genitalia are very similar to several other species of the *M.pentheres* species group but differ in the particularly stout and well sclerotised uncus and other subtle characters. From the most similar *M.quadristictus* (Figs 255–256) they are separated by the more slender shape of the saccus. See also *M.korabicus* sp. n. (p 170).

##### Molecular data.

BIN BOLD:ABW5890 (n = 6), BOLD:ADF1918 (n = 1). Genetically variable species. The intraspecific divergence of the barcode region is large and reflected by 2 BINs with 5.4% divergence, based on a single specimen from Greece compared with a larger cluster from Croatia to Italy. Within the latter cluster average divergence is considerable with mean 1.6% and maximum divergence of 2.4%. The minimum distance to the nearest neighbour *M.quadristictus* is 9.7% (p-dist).

##### Distribution.

Croatia, Greece, Italy, Slovenia.

##### Biology.

Host plant and early stages are unknown. The adults have been collected from early August to early September at altitudes from sea level to ca. 1300 m.

##### Etymology.

This species is named after the most northern place of its occurrence: present day South Tyrol (Italy). The name is an adjective.

##### Remarks.

The considerable barcode split in two clusters is not reflected by the morphology. In particular the length of the sacculus shows some variation, e.g., dissected specimens of both clusters have a long sacculus, whereas it is short in one specimen from Greece. The separation of *M.teriolensis* sp. n., *M.korabicus* sp. n., and *M.quadristictus* is almost entirely based on differences in the barcode, because morphological differences are minute.

#### Megacraspedus korabicus sp. n.

Taxon classificationAnimaliaLepidopteraGelechiidae

http://zoobank.org/114E82C7-B8F7-49A4-927D-FBDF4BFCDB13

##### Examined material.

**Holotype** ♂, “Macedonia NP [National Park] nMavrovo Korab [mountain], Korabska jezero, Kobilino pole, 2080–2180 m 20°34'55"E, 41°46'42"N 28.7.–1.8.2011 leg. Huemer & Tarmann” “*Megacraspedusbinotella* det. P. Huemer, 2011” “P. Huemer GEL 1183 ♂” “BC TLMF Lep 05179” (TLMF). **Paratypes.** Macedonia. 1 ♀, same data as holotype (TLMF), but genitalia slide GEL 1273 ♀ P. Huemer; 2 ♂, Galičia, 3.viii.2015, leg. I. Richter (NMPC).

##### Description.

Adult. *Male* (Figure 136). Wingspan 13–15 mm. Segment 2 of labial palpus with scale brush about as long as segment 3, black on outer surface, white mottled with black on inner surface, white on lower and upper surface; segment 3 whitish. Antennal scape with pecten of one fine hair; flagellum grey-brown, indistinctly ringed lighter. Head, thorax and tegula cream-white. Forewing cream coloured mottled with yellow and some dark brown, darkest at costal third; two elongate black dots in fold and two black dots in middle of wing and at end of cell; some black scales along termen; fringes grey. Hindwing dark grey with light grey fringes.

*Female* (Figure 137). Wingspan 8 mm. Overall lighter than male. Forewing with apical half tapered, cream-white: a small black dot in fold, one in middle of wing, and one more distinct black dot at end of cell; fringe concolorous with forewing. Hindwing slightly reduced, about three-quarters length of the forewing, but more slender, emarginated before apex and tapered towards tip, whitish.

*Variation*. The yellowish dorsal part of the forewing can be more or less mottled with greyish scales. The hairs on the antennal scape are easily broken. Worn specimens become lighter.

*Male genitalia* (Figure 254). Uncus moderately small, basally constricted, suboval, slightly shorter than broad, sub-basally widened, distinctly tapered to weakly rounded apex; gnathos hook moderately slender, slightly exceeding length of uncus, weakly curved, apically pointed; tegumen smooth, with weakly developed merged ridges anteromedially, anterior margin with broad and moderately shallow emargination, additional small and shallow excavation medially; pedunculi moderately small; valva long, extending to about tip of uncus, broader at base, distal part slender, with rounded apex; sacculus developed, short, slender digitate; posterior margin of vinculum with shallow medial emargination, weakly developed lateral humps, vincular sclerites irregularly oblong with broad base; saccus broad, with weakly convex outer edge, distally tapered to pointed apex, moderately short, ratio maximum width to length about 1, posterior margin broadly arched, nearly sinusoid, with broad and shallow medial emargination, medial part smooth, without sclerotised ridge, lateral sclerites short, approximately 0.6 times length of maximum width of saccus; phallus gradually tapered, with weakly defined bulbous coecum, distal two-thirds stout, straight, with broadly sclerotised zone dorsally and slender sclerotised ventral ridge, subapically with indistinct tooth.

*Female genitalia* (Figure 299). Papilla analis large, approximately 0.7 mm long, apex broadly rounded; apophysis posterior rod-like, about 1 mm long, posteriorly bordered by large, longitudinal sclerotised field, posteriorly weakly widened and bent at about one-fifth, apex weakly inflated and rounded; segment VIII approximately 0.4 mm long, posteriolaterally sclerotised, medially membranous; subgenital plate with sub-triangular, anteriorly band-like, curved subostial sclerotisation, with broad and shallow projection anteriorly, posteriorly extended into short, pointed sub-medial sclerites, delimiting oblong ostium bursae; apophysis anterior rod-like, slightly longer than segment VIII; colliculum short, weakly sclerotised; ductus bursae slender, approximately 1.4 mm long; corpus bursae approximately 1.1 mm long, broadly suboval, clearly delimited from ductus bursae; signum small, irregularly shaped, weakly spinous plate in posterior part of corpus bursae.

##### Diagnosis.

**Figures 139–144.**
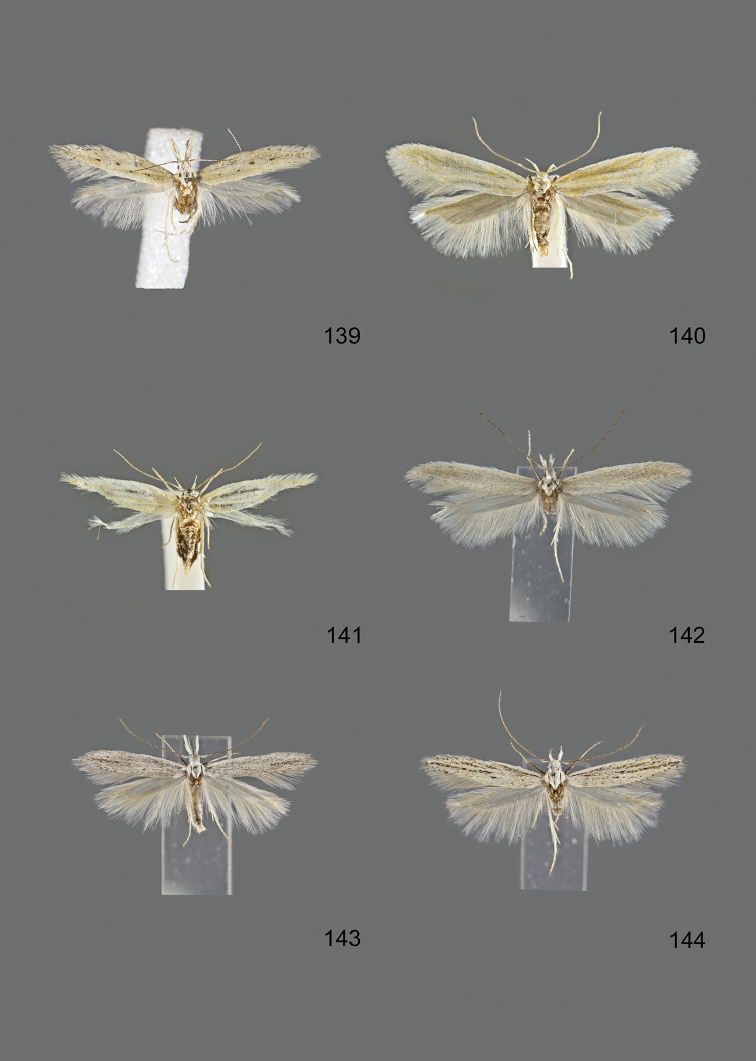
*Megacraspedus* adults in dorsal view. **139***M.quadristictus* Lhomme, 1946 – female, France (TLMF) **140***M.eburnellus* Huemer & Karsholt, 2001 – Holotype, male, Italy (TLMF) **141***M.eburnellus* Huemer & Karsholt, 2001 – Paratype, female, Italy (TLMF) **142***M.skulei* sp. n. – Holotype, male, Spain (ZMUC) **143***M.skulei* sp. n. – Paratype, female, Spain (TLMF) **144***M.longivalvellus* sp. n. – Paratype, male, Morocco (TLMF).

*Megacraspeduskorabicus* sp. n. is characterised by cream coloured forewing with four black dots. It is very similar to *M.quadristictus* (Figs 138–139) and *M.teriolensis* sp. n. (Figs 134–135), from which it can be separated by the lighter forewings, also in the costal part. The single known female is very small, having cream-white forewings with indistinct black dots and hindwings similar in shape to those of *M.quadristictus*. The male genitalia are very similar to several other species of the *M.pentheres* species group, particularly *M.teriolensis* sp. n. (Figs 252–253) and *M.quadristictus* (Figs 255–256), having only subtle differences such as the shape of the uncus and the moderately slender gnathos hook. The female genitalia are particularly similar to *M.quadristictus* (Figure 300) but differ e.g., by the more slender sub-medial sclerites of segment VIII and the much smaller and less spiny signum.

##### Molecular data.

BIN BOLD:ABA2917 (n = 3). The intraspecific divergence of the barcode region is low with mean 0.2% and maximum divergence of 0.3%. The distance to the nearest neighbour *M.quadristictus* is 10.1% (p-dist).

##### Distribution.

Macedonia.

##### Biology.

Host plant and early stages are unknown. The few adults known to date were collected from the end of July to early August in alpine grassland at an altitude from 1500 to 2100 m.

##### Etymology.

This species is named after its place of occurrence: the Korab mountain range on the border between Albania and Macedonia. The name is a masculine adjective.

#### Megacraspedus quadristictus

Taxon classificationAnimaliaLepidopteraGelechiidae

Lhomme, 1946

Megacraspedus quadristictus Lhomme, 1946: 537.

##### Examined material.

**Lectotype** ♂, **here designated**, “*quadristictus*” “Cousson 11.8.03” genitalia slide PGCG 8018 (MNHN) [photographs examined]. **Paralectotype** ♂, on polyporus with lectotype, same data, but without genitalia slide (MNHN) [photographs examined]. **Non-type material.** France. 1 ♂, Dep. Alpes-Maritimes, Cipières, 9.viii.1995, genitalia slide 5630 Nel (TLMF); 2 ♂, Dep. Drôme, St. Paul, St. Restitut, Trois Chateaux, 250 m, viii.1984, leg. M. Fibiger & A. Moberg (ZMUC); 1 ♂, Dep. Drôme, La Penne-sur-l’Ouvèze, 1–7.viii.1986, leg, H. van der Wolf (RCHW); 1 ♂, Dep. Var, Callas, 5.ix.1997, leg. H. Queis (ZMUC); 1 ♂, Dep. Var, Tourves, grand Tour rout, 30.viii.2002, genitalia slide GEL 1254 Huemer; 1 ♂, Dep. Vaucluse, Méthamis, 2.viii.1995, leg. G. Brusseaux, genitalia slide 4163 Nel; 1 ♀, Dep. Vaucluse, Méthamis, le Plafournier, leg. G. Brusseaux, 27.viii.2000, genitalia slide GEL 1265 Huemer; 1 ♀, same data, but 21.viii.2001, leg. G. Brusseaux, genitalia slide 13982 Nel; 1 ♂, same data, but 24.viii.2001, leg. G. Brusseaux; 1 ♂, same data, but 13.viii.2001, leg. G. Brusseaux, genitalia slide GEL 1207 Huemer; 1 ♀, same data, but 2.viii.1995, [second label (!)]: Dep. Vaucluse, Caseneuove, D 35, 380 m, 30.viii.1993, leg. J. Nel [abdomen missing] (all TLMF); 1 ♂, Dep. Vaucluse, Fouix, St. Martin Castillon, 23.viii.1994, leg. J. Nel; 1 ♂, Dep. Vaucluse, Viens, le Calavon, 16.viii.1993, leg. J. Nel, genitalia slide 1156 Nel; 1 ♂, Dep. Vaucluse, Fontaine de Vaucluse, 4–14.viii.1961, leg. H. Malicky, genitalia slide GEL 1204 Huemer; 1 ♂, Dep. Pyrénées-Orientales, Alenya, étang de Canet 4.ix.1994, leg. R. Mazel (all TLMF). Portugal. 3 ♂, Algarve, Bensafrim, Colinas Verdes, 26.ix.1995, leg. M. F. V. Corley, genitalia slide Corley 773 (RCMC); 1 ♂, Algarve, Estremadura, Sesimbra, 1.ix.2002, leg. M. F. V. Corley, genitalia slide Corley 1879 (RCMC). Spain. 1 ♂, prov. Barcelona, Avinyonet, 27.ix.1973, leg. P. L. Holst (ZMUC); 1 ♂, prov. Barcelona, Anoia, Òdena, salze 13.viii.2004, leg. E. Requena; 1 ♂, prov. Barcelona, Anoia, Mirales, 11.viii.2004, leg. E. Requena; 1 ♂, prov. Barcelona, Bages, Castellfullit del Boix bassa 2.viii.2003, leg. E. Requena (all RCER); 2 ♂, prov. Barcelona, 2 km W Gurb, Vic, 550 m, 15.viii.2001, leg. P. Skou; 1 ♂, prov. Castellon, 5 km E Cuevas de Vinroma, 200 m, 13.vii.1992, leg. M. Fibiger (all ZMUC); 2 ♂, prov. Cuenca, Valdemeca, 31.viii.2002, leg. H. van der Wolf (RCHW); 1 ♀, prov. Cuenca, Paracuellos, 22.ix.1983, leg. C. Gielis; 1 ♂, prov. Cuenca, 6 km S Salvacañete, 22.ix.2001, leg. C. Gielis (all RMNH); 1 ♂, prov. Cuenca, Uña, 1150 m, 28.viii.2001, leg. B. Skule; 1 ♂, prov. Cuenca, Valdecabras, 23–30.ix.1995, leg. J. Wolschrijn (all ZMUC); 1 ♂, prov. Cuenca, Uña, Castilla la Mancha, 1300 m, 12.ix.2007, leg. J. Viehmann (RCWS); 10 ♂, prov. Girona, near Sargas at B 432, 650 m, 13.viii.2001, leg. P. Skou (ZMUC); 2 ♂, Granada, 10 km NE Baza, 800 m, 27.ix.1994, leg. H. van der Wolf (RCHW); 1 ♂, prov. Huesca, Biescas, 1.viii,1989, leg. C. Gielis (RMNH); 2 ♂, prov. Huesca, 10 km S Benabarre, Esteña, 700 m, 18–19.viii.2001, leg. B. Skule & P. Skou; 2 ♂, same data, but 8.ix.2001, leg. B. Skule & C. Hviid; 3 ♂, same data, but 2.viii.2007, leg. B. Skule & P. Skou, genitalia slide GU 14/1380 Huemer; 3 ♂, prov. Huesca, 10 km sw Bielsa, Revilla, 1150 m, leg. B. Skule; 1 ♂, prov. Lleida, 30 km NW Fraga, Ontinema, 250 m, 11.vii.1992, leg. M. Fibiger; 1 ♂, prov. Teruel, Cosa, 2–13.viii.1989, leg. C. Gielis (all ZMUC); 2 ♂, prov. Teruel, 4 km E Cosa, 28.viii. 2000, leg. H. van der Wolf (RCHW); 1 ♂, prov. Teruel, Albarracin, Valdevecar, 1200 m, 21.viii.2001, leg. B. Skule & P. Skou; 3 ♂, 2 ♀, same data, but 1150 m, 1.viii.2003, leg. P. Skou; 5 ♂, same data, but 4.viii.2007, leg. B. Skule & P. Skou; 3 ♂, 5 km NW Montalban, 950 m, 17.vii.2003, leg. B. Skule; 2 ♂, same data, but 5.viii.2007, leg. B. Skule, genitalia slide 6492 Hendriksen; 1 ♂, same data, but 3 km WSW Montalban, 1450 m, 6.viii.2007, leg. B. Skule & P. Skou; 1 ♂, prov. Zaragoza, Rio Huerva, Tosos, 550 m, 20.viii.2001, leg. B. Skule & P. Skou (all ZMUC); 3 ♂, prov. Castellon, Penyagolosa E slope, 1450 m, 1.ix.2005, leg. P. Huemer, genitalia slide GEL 1223 Huemer (TLMF); 1 ♂, prov. Valencia, El Saler, Albufera, 5 m, 8.ix.2005, leg. P. Huemer (TLMF).

##### Redescription.

Adult. *Male* (Figure 138). Wingspan 12–17 mm. Segment 2 of labial palpus with scale brush longer than segment 3, dark brown on outer surface, white mottled with brown on inner surface, white on lower and upper surface; segment 3 white with black tip. Antennal scape with pecten of fine hairs; flagellum light brown ringed with dark grey. Head cream-white; thorax and tegulae as forewing. Forewing light yellow mottled with brown- and black-tipped scales, especially along veins, at base, along costa and near apex; costa blackish near base; fold and sub-costal stripe yellow, area between them greyish; two black dots in fold and two in middle of wing and at end of cell; some black scales along termen; fringes greyish. Hindwing grey with light grey fringes.

*Female* (Figure 139). Wingspan 13–16 mm. Segment 2 of labial palpus with white inner surface. Antenna ringed black and cream-white. Forewing with apical half lanceolate, white mottled with light yellow, especially along veins; only a few black-tipped scales, especially as an indistinct sub-costal patch near base, and along termen; the four black dots distinct; fringes whitish grey. Hindwing more slender than in males, white with whitish grey fringes.

*Variation*. The forewing colour varies from yellowish to greyish. A few specimens have an indistinct, dark, sub-costal patch near base. Worn specimens become lighter, and the black dots can become obsolete, and in such specimens the antennal pecten is often lost.

*Male genitalia* (Figs 255–256). Uncus moderately small, basally constricted, irregularly rounded, slightly shorter than broad, sub-basally widened with weak lateral bulge, distally tapered to broadly rounded apex; gnathos hook stout, nearly 1.5 times length of uncus, weakly curved, lateromedially widened, apically pointed; tegumen smooth, with weakly developed ridge anteromedially, anterior margin with broad and moderately shallow emargination, additional small and shallow excavation medially; pedunculi moderately small with transverse ridge; valva long, extending slightly beyond tip of uncus, broader at base, distal part slender, apically weakly curved with broadly rounded apex; sacculus well developed, short, slender digitate; posterior margin of vinculum with shallow medial emargination, distinctly developed lateral humps, vincular sclerites irregularly oblong with broad base; saccus variable, broad, nearly U-shaped to broadly V-shaped, distal third curved to rounded apex or with weakly pointed apex, moderately short, ratio maximum width to length approximately 1.2, posterior margin broadly arched, nearly sinusoid, with broad and shallow medial emargination, medial part smooth, with indistinct short sclerotised ridge, lateral sclerites short, approximately 0.6 times length of maximum width of saccus; phallus gradually tapered, with weakly defined bulbous coecum, distal two-thirds stout, straight, with broadly sclerotised zone dorsally and slender sclerotised ventral ridge, subapically with indistinct tooth.

*Female genitalia* (Figure 300). Papilla analis large, apically rounded, slightly longer than segment VIII; apophysis posterior rod-like, approximately 1.2 mm long, posteriorly bordered by large, longitudinal sclerotised field, posteriorly weakly widened and bent at about one-fifth, apex widened and rounded; segment VIII approximately 0.5 mm long, posteriolaterally sclerotised, medially membranous with microsculpture; subgenital plate with sub-triangular, anteriorly pointed subostial sclerotisation with sclerotised oblique rods posteriorly, broad and shallow projection anteriorly, posteriorly extended into short, pointed and flap-like sub-medial sclerites, delimiting rounded ostium bursae; apophysis anterior rod-like, about length of segment VIII; colliculum short and weakly sclerotised; ductus bursae slender, about 2 mm long; corpus bursae approximately 1.3 mm long, slender, weakly delimited from ductus bursae; signum small, laterally oblong spiny plate, with particularly strong lateral spines.

##### Diagnosis.

*Megacraspedusquadristictus* is characterised by its light grey-brown forewings with yellow veins and dorsum and four black dots, and the antennal scape with a pecten of fine hairs. It is very similar to *M.teriolensis* sp. n., but is on average larger and has more yellow on the forewing, especially along the veins. See also *M.korabicus* sp. n. (p 170). The male genitalia are very similar to several other species in the *M.pentheres* species group but differ by the particularly stout and well sclerotised uncus and other more subtle characters. From the most similar *M.teriolensis* sp. n. (Figs 252–253) they are separable by the broader saccus. The female genitalia are most similar to *M.korabicus* sp. n. (Figure 299) but differ in the subostial sclerotisation and the larger signum with strong spines.

##### Molecular data.

BIN BOLD:AAU1830 (n = 5). The intraspecific divergence of the barcode region is moderate with mean 1% and maximum divergence of 1.6% (n = 5). The distance to the nearest neighbour *M.teriolensis* sp. n. is 9.7% (p-dist).

##### Distribution.

France, Spain.

##### Biology.

Host plant and early stages are unknown. The adults have been collected from early August (rarely from the middle of July) to the end of September from the coast to altitudes of ca. 1450 m.

##### Remarks.

*Megacraspedusquadristrictus* was described from an unstated number of specimens (“quelques exemplars”) from France, Digne-Le Cousson, in the collection of P. Chrétien (Lhomme 1946: 537). We have been able to examine photographs of syntypes from MNHN. A male specimen is here designated as the lectotype in order to fix the identity of the species and conserve stability of nomenclature.

#### Megacraspedus eburnellus

Taxon classificationAnimaliaLepidopteraGelechiidae

Huemer & Karsholt, 2001

Megacraspedus eburnellus Huemer & Karsholt, 2001: 238, figs 3, 8, 10, 13–14.

##### Examined material.

**Holotype** ♂, “Italia [prov. Verona] Mte Baldo Noveza 1300–1600 m M.6.66. leg. Burmann” “TLMF Innsbruck 1988-12” (TLMF). **Paratypes.** Italy. 1 ♂, same data as holotype (SMNK); 2 ♂, 1 ♀, same data, but medio vii.1966 (SMNK, TLMF); 1 ♂, same data, but 19.vi.1993, leg. P. Huemer (TLMF); 5 ♂, same data, but Costabella, 1800 m, ultimo vi.1965, leg. K. Burmann (TLMF); 1 ♂, same data, but Bocca di Navene, 1400–1500 m, medio vi.1969, 1 ♂, same data, but medio vi.1970, 1 ♂, same data, but ultimo vi.1971, leg. K. Burmann (SMNK); 1 ♂, Monte Baldo, Naole, 1500–1600 m, 21.vii.1989, leg. O. Karsholt (ZMUC); 3 ♂, 1 ♀, prov. Verona, Monte Baldo, Dintorni Refugio, Novezzina, 1250 m, 24.vii.1984, leg. U. Parenti, genitalia slide GU00/891 Huemer (ZMUC); 1 ♂, prov. Verona, Monte Baldo, Naole, 1500–1600 m, 21.vii.1989, leg. O. Karsholt (ZMUC); 3 ♂, prov. Verona, Monte Baldo, 1700 m, medio vii.1959 (SMNK); 1 ♂, Garda, medio v.1970, leg. K. Burmann (SMNK); 1 ♂, prov. Brescia, Anfo, Cima Valcai S-Hang, 1200 m, 14.vi.1987, leg. P. Huemer (TLMF); 1 ♂, 3 ♀, prov. Bergamo, Alpi Orobie, Val d´Arera, 2000 m, 14–15.viii.1992, leg. P. Huemer & G. Tarmann (TLMF); 35 ♂, 1 ♀, same data, but 2100 m, 23–24.viii.1992 (TLMF); 6 ♂, same data, but Pizzo Arera, 2000 m, 2–3.viii.1999, leg. Nuss (MTD). **Non-type material.** Italy. 1 ♂, prov. Trento, Brentonico, Rif. Graziani, 1600 m, 22.vii.1977, leg. U. Parenti (ZMUC); 12 ♂, 2 ♀, prov. Verona, Monte Baldo, Dintorni Rifugio Novezzina, 1250–1300 m, 23–24.vii.1984, leg. U. Parenti; 1 ♂, same data, but 19.vii.1985; 2 ♂, same data, but 22.vii.1986; 1 ♂, same data, but 29.vii.1986; 2 ♂, same data, but 24.vii.1992 (all ZMUC).

##### Redescription.

Adult. *Male* (Figure 140). Wingspan 14–19 mm. Segment 2 of labial palpus with short scale brush, light brown on outer and lower surface, white on inner and upper surface; segment 3 of about same length as segment 2, white. Antennal scape without pecten, flagellum indistinctly ringed whitish and mid-brown. Head whitish; thorax and tegula as forewing. Forewing whitish cream, more or less mottled with light brown; basal part of costa whitish; fringes concolorous with forewing. Hindwing light grey with whitish cream fringes.

*Female* (Figure 141). Wingspan 9–13 mm. Same colour as male, but fore- and hindwing more lanceolate and in particular hindwings distinctly reduced in width. Otherwise similar to male.

*Variation*. The forewing colour varies from whitish cream to light brown. Worn specimens look whitish. The tip of segment 3 of the labial palps is sometimes black.

*Male genitalia* (Figure 257). Uncus moderately moderately large, slightly shorter than broad, basally constricted, sub-basally widened, distally tapered to broadly rounded apex; gnathos hook stout, approximately 1.3 times length of uncus, weakly curved, lateromedially widened, apically pointed; tegumen smooth, with weakly rounded anterior and short anteriomedial ridge, anterior margin with broad and shallow emargination; pedunculi of moderate size, with transverse ridge; valva long, extending almost to tip of uncus, broader at base, distal part slender, apically weakly curved and pointed; sacculus well developed, digitate; posterior margin of vinculum with shallow medial emargination, distinctly developed lateral humps, vincular sclerites irregularly oblong with broad base; saccus large, nearly V-shaped, with broadly rounded apex, moderately long, ratio maximum width to length about 1, posterior margin broadly arched, nearly sinusoid, with broad and shallow medial emargination, medial part smooth, with indistinct short and furcated ridge, lateral sclerites short, approximately 0.6 times length of maximum width of saccus; phallus gradually tapered, with weakly defined bulbous coecum, distal two-thirds stout, straight, with broadly sclerotised zone dorsally and slender sclerotised ventral ridge, elongated plate-like cornutus with several short teeth.

*Female genitalia* (Figure 301). Papilla analis very large, apically rounded, almost two times length of segment VIII; apophysis posterior rod-like, approximately 1.4 mm long, posteriorly bordered by sclerotised zone of papilla analis,; segment VIII approximately 0.5 mm long, posteriolaterally sclerotised, medially membranous with microsculpture; subgenital plate with subostial sclerotisation, sclerotised oblique rods posteriorly, broad and shallow projection anteriorly, posteriorly extended into short, pointed and flap-like sub-medial sclerites, delimiting rounded ostium bursae, anterior edge rounded; apophysis anterior rod-like, slightly longer than segment VIII; colliculum irregularly sclerotised; ductus bursae slender, approximately 3.3 mm long; corpus bursae approximately 2.2 mm long, slender, clearly delimited from ductus bursae; signum a small, suboval, strongly dentate plate.

##### Diagnosis.

*Megacraspeduseburnellus* differs from most other European *Megacraspedus* species in lacking blackish brown or black scales, dots, or streaks on the forewings. It is externally similar to *M.dolosellus* (Figs 29–38), differing in being slightly larger and with slightly broader forewings. *M.dolosellus* moreover differs in having the veins on the forewings lighter than the ground colour of the wings. The females of this species have distinctly more reduced hindwings than the female of *M.eburnellus*. The male genitalia are overall similar to other species in the *M.pentheres* species group but differ by the apically pointed valva, the large saccus, and the characteristic dentation of the phallus. The female genitalia differ from other species in the subostial sclerotisation and the suboval signum.

##### Molecular data.

BIN BOLD:AAJ3176 (n = 2). The intraspecific divergence of the barcode region is low with mean and maximum divergence of 0.2%. The distance to the nearest neighbour *M.skulei* sp. n. is 8.5% (p-dist).

##### Distribution.

Italy (Orobian Alps to Monte Baldo).

##### Biology.

Host plant and early stages are unknown. Adults have been collected during the daytime by sweeping the vegetation with a net. Males are also attracted to light. The flight period ranges from the middle of May to the middle of August, depending on altitude. The habitat is characterised by scree and rock formations with alpine grassland vegetation at altitudes of between 1200 and 2100 m. A single specimen from the lowland locality Garda (Burmann and Huemer 1997) is probably mislabelled.

### *Megacraspeduslongivalvellus* species group

The *M.longivalvellus* species group includes two species: *M.skulei* sp. n. and *M.longivalvellus*.

External morphology. Labial palpus long, porrect; segment 3 reduced. Antennal scape without pecten. Wingspan (males) 10–17 mm. Forewing white with grey or blackish scales forming longitudinal streaks especially in apical part and with 1–2 black dots; base of costa white. Female unknown.

Genitalia morphology. Male genitalia. Uncus moderately large, apically rounded; gnathos hook strongly sclerotised, stout, weakly curved; valva digitate, extraordinarily long, extending far beyond apex of uncus, apical quarter weakly curved, separated saccular area weakly bulged but without clearly separated sacculus; saccus sub-triangular, with pointed apex, moderately long posterior margin weakly arched, medial part smooth; phallus slender, straight, coecum weakly defined, distal three-quarters digitate, without specialised structures.

Female genitalia. Papilla analis large, apex broadly rounded; apophysis posterior short, about length of papilla analis; segment VIII short, posteriolaterally sclerotised, medially membranous; subgenital plate reduced to proximally convex subostial sclerotisation, laterally connected with base of apophysis anterior by sclerotised edge, sub-medial sclerites short, anteromedial edge of segment VIII membranous; apophysis anterior short, about length of segment VIII; colliculum short, sclerotised; signum small, irregularly shaped plate with two strong lateral teeth.

Diagnosticremarks. The *M.longivalvellus* species group is defined by the long and porrecti labial palpus with reduced segment 3, and in particular by several structures of the male genitalia such as the extraordinarily long and apically curved valva, the saccular bulge, the oblong saccus, and the long and slender phallus. The female genitalia are characterised by the comparatively large papilla analis, and the short apophysis posterior and anterior as well as segment VIII, and the membranous anteromedial edge of segment VIII. They are similar overall to e.g., *M.peyerimhoffi* and a few other species but females are unknown in several other species groups.

#### Megacraspedus skulei sp. n.

Taxon classificationAnimaliaLepidopteraGelechiidae

http://zoobank.org/AB505783-C561-4EC4-8827-44B5057F3ECD

##### Examined material.

**Holotype** ♂, “SPAIN [prov.] Almeria Sierra de los Filabres Calar Alto, 2130 m N37.221523 W2.545495 5.7.2015 J. Tabell leg.” “DNA Barcode TLMF Lep 19849” genitalia prep. (in glycerin) (ZMUC). **Paratypes.** Spain. 5 ♂, prov. Almería, Sierra de los Filabres, Calar Alto, 2130 m, 5.vii.2015, leg. J. Tabell (TLMF, ZMUC); 1 ♀, prov. Cádiz, Chiclana, 25.iv., coll. Staudinger, *Anarsiadisjectella* in litt. (ZMHU); 8 ♂, prov. Cuenca, 7 km ESE Fuentes, 970–1100 m, 15.vii.2010, leg. T. Nupponen (RCKN); 4 ♂, prov. Girona, Port Bou, 0–600 m, 18.vi.–1.vii.1963, leg. M. & W. Glaser (SMNK); 1 ♂, prov. Guadalajara, 4 km E Embid, 1075 m, 8.vii.2007, leg. B. Skule & P. Skou (ZMUC); 2 ♂, prov. Huesca, 12 km S Benabarre Esteña, 700 m, 14.vii.2003, leg. B. Skule, genitalia slide GU 15/1392 Huemer (ZMUC); 3 ♂, same data, but 800 m, 17.vii.2003, leg. P. Skou, genitalia slide 4817 Hendriksen (ZMUC); 5 ♂, 1 ♀, prov. Teruel, Albarracin env., 1100 m, 28.vii.2010, leg. J. Šumpich, genitalia slides GU 16/1410 ♂ Huemer, GU 16/1411 ♀ Huemer; 5 ♂, same data, but 13.vii.2012, leg. M. Dvorak; 2 ♂, same data, but 14.vii.2012, leg. M. Dvorak; 13 ♂, same data, but 14.vii.2012, leg. M. Dvorak (all NMPC); 1 ♂, prov. Teruel, 5 km SE Albarracin, 1400 m, 18.vii.1995, leg. P Skou (ZMUC); 3 ♂, prov. Teruel, 4.5 km NE Albarracin, 1110 m, 7.vii.2016, leg. J. Tabell (TLMF, ZMUC); 1 ♂, prov. Teruel, 5 km NW Montalban, 950 m, 17.vii.2003, leg. B. Skule (ZMUC); 1 ♂, prov. Teruel, Sierra de Albarracin, Sierra Alta, 1750 m, 25.vi.2016, leg. J. Viehmann (RCWS); 5 ♂, prov. Teruel, 6 km N Pozodon, San Gines, 1500 m, 2.vii.2016, leg. J. Viehmann (RCWS, ZMUC); 1 ♂, prov. Zaragoza, 3 km E Cerveuela at Rio de Huerva, 800 m, 7.vii.2002, leg. B. Skule (ZMUC); 1 ♂, prov. Zaragoza, 5 km W Aguar´n, 750 m, 3.viii.2007, leg. B. Skule & P. Skou (TLMF).

##### Description.

Adult. *Male* (Figure 142). Wingspan 10–15 mm. Labial palpus long, porrect, blackish brown, white on upper surface; segment 3 reduced. Antennal scape without pecten; flagellum whitish brown ringed with black. Head white mottled with light brown; thorax and tegula as forewing. Forewing white, mottled with scattered black-tipped scales, especially in apical part; an indistinct black dot in fold; an elongate black dot in middle of wing before apex; basal three-quarters of costa white; fringes light grey. Hindwing light grey, with light grey fringes.

*Female* (Figure 143). Wingspan 10 mm. Hindwing slightly more slender than in male with a more produced apex. Otherwise similar to male.

*Variation*. There is some variation in size, even among specimens collected at the same date. The black dots on the forewing before apex can be absent. Strongly marked specimens have a black streak before the apex. Worn specimens become more greyish.

*Male genitalia* (Figure 258). Uncus moderately large, semicircular, evenly rounded; gnathos hook strongly sclerotised, stout, slightly longer than uncus, weakly curved, medially widened, apically pointed; tegumen with medially confluent sclerotised anterior ridges, anterior margin with moderately shallow emargination, medially additional shallow excavation; pedunculi weakly demarcated, suboval; valva digitate, extraordinarily long, extending far beyond apex of uncus, broader basal part straight, tapered apical quarter weakly curved, apex pointed, distal area covered with setae medially and apically, saccular area weakly bulged but not clearly separated from valva; posterior margin of vinculum emarginated, with weakly rounded lateral humps, vincular sclerites broadly sub-rectangular; saccus sub-triangular, with pointed apex, moderately long, ratio maximum width to length approximately 0.8, posterior margin weakly arched, with shallow medial emargination, medial part without sclerotised ridge, lateral sclerites moderately short, approximately 0.8 times length of maximum width of saccus; phallus slender, straight, coecum weakly defined, distal three-quarters digitate, without specialised structures.

*Female genitalia* (Figure 302). Papilla analis large, approximately 0.7 mm long, apex broadly rounded; apophysis posterior rod-like, short, approximately 0.7 mm long, posteriorly bordered by small sclerotised field, weakly widened at about one-fifth, apex weakly inflated and rounded; segment VIII short, approximately 0.4 mm long, posteriolaterally sclerotised, medially membranous with microsculpture; subgenital plate reduced to proximally convex subostial sclerotisation, laterally connected with base of apophysis anterior by sclerotised edge, posteriorly extended into short sub-medial sclerites, delimiting oblong ostium bursae, anteromedial edge of segment VIII membranous; apophysis anterior rod-like, about length of segment VIII; colliculum short, sclerotised; ductus bursae slender, gradually widened to weakly defined corpus bursae, entire length of ductus and corpus bursae approximately 1.9 mm; signum small, irregularly shaped plate, posteriorly with two strong lateral teeth.

##### Diagnosis.

*Megacraspedusskulei* sp. n. is, together with *M.knudlarseni* sp. n. (p 122), the smallest species amongst *Megacraspedus* with a reduced segment 3 of the labial palps. It is similar to *M.longivalvellus* sp. n. (p 179). The male genitalia are almost unique within *Megacraspedus* and can be easily recognized by the extremely long and distally curved valva. They differ from the closely related *M.longivalvellus* sp. n. (Figs 259–260) in the more rounded shape of the uncus, the smaller gnathos hook, and the shorter and gradually tapered saccus. The female genitalia differ from other *Megacraspedus* in particular in the short apophysis posterior and the medially membranous anterior margin of segment VIII. However, females of the closest species *M.longivalvellus* sp. n. remain unknown for the time being.

##### Molecular data.

BIN BOLD:ACM0982 (n = 5). The intraspecific divergence of the barcode region is low with mean 0.3% and maximum divergence of 0.6%. The distance to the nearest neighbour *M.longivalvellus* sp. n. is 2.3% (p-dist).

##### Distribution.

Spain.

##### Biology.

Host plant and early stages are unknown. The adults have been collected from the second half of June to early August at altitudes from lowland to 2130 m.

##### Etymology.

The species name (a noun in the genitive case) is dedicated to Bjarne Skule, Denmark, who collected part of the type-series of this species and numerous other *Megacraspedus* specimens used for our study.

##### Remarks.

The species status of the closely allied and allopatric *M.skulei* sp. n. and *M.longivalvellus* sp. n. is supported by morphology and DNA barcodes.

#### Megacraspedus longivalvellus sp. n.

Taxon classificationAnimaliaLepidopteraGelechiidae

http://zoobank.org/80303CDE-A234-45C5-8BE1-F67AB2EFDA9A

##### Examined material.

**Holotype** ♂, “Morocco [Middle Atlas] 1400–2000 m Azrou/Ifrane area 17–19.iv.1989 Zool. Mus. Copenh. Exp.” “GU 16/1422 ♂ P. Huemer” (ZMUC). **Paratypes.** Morocco. 1 ♂, High Atlas, Oukaïmeden, 2400 m, 7–17.vi.1965, leg. Y. de Lajonquière (SMNK); 3 ♂, prov. Al Haouz, Imlil, 1680 m, 30.vi.2016, leg. J. Tabell, genitalia slide GEL 1249 Huemer (TLMF, ZMUC); 5 ♂, prov. Ouarzazate, 1 km ESE Aguelmouss, 2150 m, 3.vii.2016, leg. J. Tabell (TLMF, ZMUC); 1 ♂, Ifrane, 23.–24.vi.1972, leg. F. Hahn (ZSM).

##### Description.

Adult. *Male* (Figure 144). Wingspan 13–17 mm. Labial palpus long, porrect, blackish brown, white on upper surface; segment 3 reduced. Antennal scape without pecten; flagellum whitish brown ringed with black. Head, thorax and tegula white. Forewing white with a yellowish white tinge and mottled with white black-tipped scales between veins; sub-costal and apical streaks black; small black dots at end of fold and at end of cell; fringes whitish grey with scattered black at base. Hindwing light grey, with light grey fringes.

*Female*. Unknown.

*Variation*. The amount of black scales and the distinctness of the black spots on the forewing are variable. The yellowish white tinge on the forewing is only present in fresh specimens.

*Male genitalia* (Figs 259–260). Uncus moderately large, sub-rectangular, apically evenly rounded; gnathos hook strongly sclerotised, stout, nearly 2 times length of uncus, weakly curved, medially widened, apically pointed; tegumen with medially confluent sclerotised anterior ridges, anterior margin with moderately shallow emargination, medially additional shallow excavation; pedunculi weakly demarcated, suboval; valva digitate, extraordinarily long, extending far beyond apex of uncus, broader basal part straight, tapered apical quarter weakly curved, apex pointed, distal area covered with setae medially and apically, saccular area weakly bulged but not clearly separated from valva; posterior margin of vinculum emarginated, with weakly rounded lateral humps, vincular sclerites broadly sub-rectangular; saccus sub-triangular, with long and pointed rod-like apex, ratio maximum width to length approximately 0.65, posterior margin weakly arched, with shallow medial emargination, medial part without sclerotised ridge, lateral sclerites slightly shorter than maximum width of saccus; phallus slender, straight, coecum weakly defined, distal three-quarters digitate, without specialised structures.

*Female genitalia*. Unknown.

##### Diagnosis.

*Megacraspeduslongivalvellus* sp. n. is similar to *M.skulei* sp. n. (Figure 142), but differs in being larger, by the white head, thorax and tegulae, and by its more black-striped forewings. *Megacraspedusknudlarseni* sp. n. (Figure 94) also has a reduced segment 3 of the labial palps, but lacks the black stripes on the forewing. The male genitalia are almost unique within *Megacraspedu*s and can be easily recognized by the extremely long and distally curved valva. They differ from the closely related *M.skulei* sp. n. (Figure 258) in the less rounded shape of the uncus, the larger gnathos hook, and the longer and apically rod-like saccus.

##### Molecular data.

BIN BOLD:ADF1825 (n = 2). The intraspecific divergence of the barcode region is moderate with mean 0.9%. The distance to the nearest neighbour *M.skulei* sp. n. is 2.3% (p-dist).

##### Distribution.

Morocco (Middle Atlas and High Atlas).

##### Biology.

Host plant and early stages are unknown. The type material was collected in the middle of April and from June to early July at altitudes ranging from ca. 1400 to 2400 m.

##### Etymology.

The species name is derived from a combination of the Latin words *longus* and *valva*, indicating the characteristically elongated valva, and the diminutive suffix –*ellus*. The name is a noun in apposition.

##### Remarks.

DNA barcode divergence and diagnostic characters of adults including the male genitalia support a separate specific status of *M.skulei* sp. n. and *M.longivalvellus* sp. n.

### *Megacraspeduspeyerimhoffi* species group

The *M.peyerimhoffi* species group includes one species: *M.peyerimhoffi*.

External morphology. See species description.

Genitalia morphology. Male genitalia. See species description.

Female genitalia. See species description.

Diagnosticremarks. The *M.peyerimhoffi* species group is defined by structures of the male genitalia, in particular the characteristic shape of the uncus and gnathos hook, the long valva without separated sacculus, and the sclerotised ridges of the saccus. The female genitalia are characterised by combined characters such as the short apophysis posterior and anterior and the long sub-medial sclerites of segment VIII, but females are unknown in several species groups and the diagnostic value of these structures is uncertain.

#### Megacraspedus peyerimhoffi

Taxon classificationAnimaliaLepidopteraGelechiidae

Le Cerf, 1925

Megacraspedus peyerimhoffi Le Cerf, 1925: 12, figs 1–19.

##### Examined material.

**Lectotype** ♂, *Megacraspeduspeyerimhoffi*, **here designated**, “TYPE” “*Megacraspedus Peyerimhoffi* ♂ Le Cerf” “MUSÉUM PARIS F. LE CERF” “chenille dans alfa Boaira-Sahari V-VI-24 P. de Peyerimhoff áclos Paris 12/13-VI-1925” genitalia slide PGCG 7964 (MNHN) [photographs examined]. **Non-type material.** Algeria. 1 ♂, Guelt-es-Stel, 27–30.ix.1929, leg. Zerny; 1 ♂, same data, but 2–10.x.1929, genitalia slide Mus. Vind. 16.654 (NHMW). Tunisia. 1 ♀, 40 km S Tuni, 29.ix.2007, leg. B. Schacht (ZSM); 12 ♂, 7 ♀, prov. Beja, 7 km W Nefza, W lake Sidi el Barrak, 30 m, 4.x.2007, leg. B. Schacht, genitalia slides 5262 ♂, 5267 ♂ Stübner, GEL 1267 ♂, GEL 1268 ♀ Huemer (TLMF, ZSM); 1 ♂, 2 ♀, prov. Nabeul, Umg. Soliman, Boj-Cedria, 0–20 m, 5.x.2007, leg. B. Schacht (ZSM). Spain. 12 ♂, 5 ♀, prov. Almería, Tabernas, ’Mini Hollywood’, 230 m, 14–15.x.1992, leg. M. Fibiger; 1 ♂, same data, but leg. F. Schepler; 1 ♂, prov. Almería, Rambla de Tabernas, 24–25.x.2003, leg. J. Viehmann; 1 ♂, prov. Almería, El Pozo del Esparto, 20 m, 1.xi.2005, leg. B. Skule & P. Skou (all ZMUC); 2 ♂, 2 ♀, prov. Almería, Desierto de Tabernas, 471 m, 14.x.2006, leg. M. Rondós (TLMF); 3 ♂, Almería, 6 km S Serón, at A1178, 1300 m, 16.ix.2009, leg. P. Skou, genitalia slide GU 13/1351 Huemer (ZMUC); 3 ♂, 1 ♀, prov. Almería, Tabernas, env., Rambla de Tabernas, 18–19.x.2009, leg. J. Šumpich, genitalia slide GU 16/1409 ♀ Huemer (NMPC, TLMF); 1 ♀, same data, but 100 m, 24.x.2016, leg. J. Viehmann; 1 ♂, 2 ♀, prov. Castellon, Cati-Morella, 700 m, leg. J. Viehmann (all RCWS); 1 ♂, prov. Granada, Almunecar, 150 m, 22–27.x.2000, leg. G. Jeppesen; 1 ♀, prov. Granada, Sierra de Los Guájares, 1100 m, 20–25.ix.2004, leg. A. Cox (all ZMUC); 5 ♂, 2 ♀, same data, but 1160 m, 26.viii.–15.ix.2005, leg. G. Jeppesen, genitalia slide 6489 Hendriksen (TLMF, ZMUC); 1 ♂, prov. Granada, 10 km NE Baza, 700 m, 20.ix.2012, leg. J. Tabell, genitalia slide Tabell 5017; 1 ♂, prov. Lleida, Castelldans, Les Garrigues, 353 m, 29.x.2010, leg. J. J. Péres De-Gregorio, genitalia slide 1950 Requena (RCER); 1 ♂, prov. Madrid, Perales de Tarjuna, 2.x.1995, leg. J. Wolschrijn; 1 ♀, prov. Málaga, Marbella, El Mirador, 700 m, 29.ix.1982, leg. E. Traugott-Olsen, genitalia slide 5836 Traugott-Olsen; 1 ♂, 8 ♀, same data, but 100 m, 15.x.1984, leg. E. Traugott-Olsen, genitalia slide 5834 Traugott-Olsen; 1 ♀, same data, but Camino de Ojen, 150 m, 15.x.1984; 1 ♂, 1 ♀, same data, but Casa y Campo, 100 m, 17.x.1984 (all ZMUC); 1 ♀, prov. Málaga, 4 km W El Burgo, 800 m, 4.x.1993, leg. H. van der Wolf (RCHW).

##### Redescription.

**Figures 145–150.**
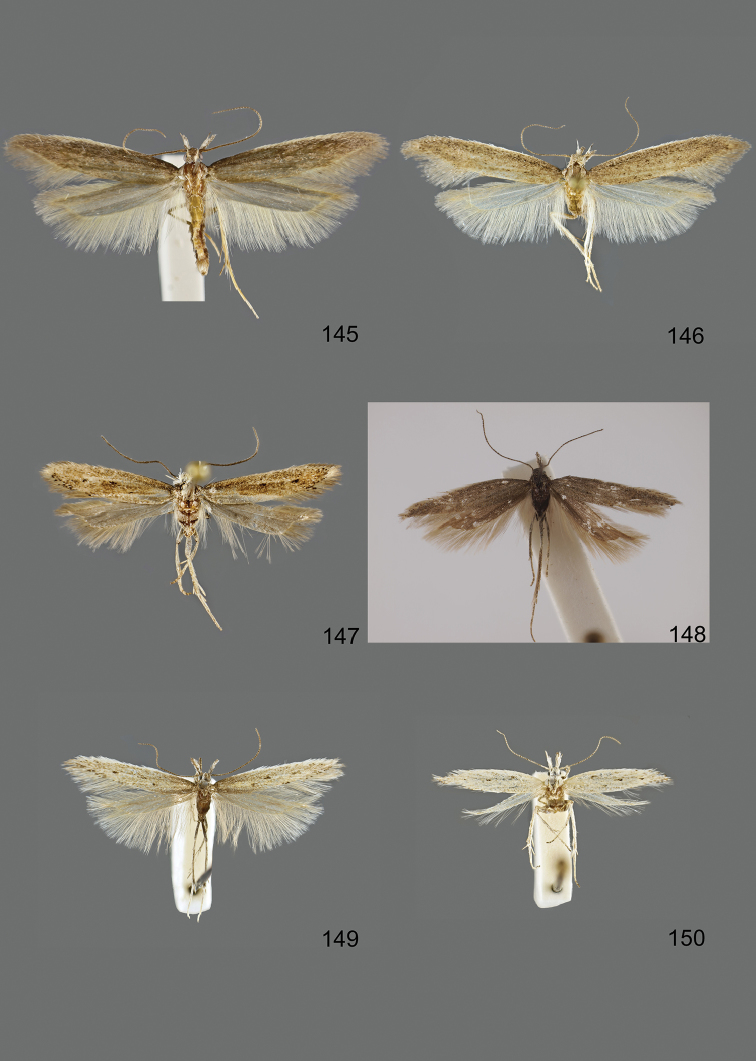
*Megacraspedus* adults in dorsal view. **145***M.peyerimhoffi* Le Cerf, 1925 – male, Spain (NMPC) **146***M.peyerimhoffi* Le Cerf, 1925 – female, Spain (NMPC) **147***M.peslieri* sp. n. – Holotype, male, France (TLMF) **148***M.grisea* (Filipjev, 1931) – Syntype, male, China (BMNH) **149***M.pacificus* sp. n. – Holotype, male, Afghanistan (NHMW) **150***M.pacificus* sp. n. – Paratype, female, Afghanistan (NHMW).

Adult. *Male* (Figure 145). Wingspan 18–25 mm. Labial palpus comparatively small; segment 2 with long scale brush, dark brown on outer surface, white mottled with brown on inner and lower surface, white on upper surface; segment 3 dark brown mottled with some white especially at base. Antenna brown, indistinctly ringed lighter. Head, thorax and tegula light grey-brown, the latter with blackish base. Forewing elongate, grey-brown, blackish near base, lighter towards dorsum, with scattered black scales; costa white in middle; fringes grey. Hindwing grey with light grey fringes.

*Female* (Figure 146). Wingspan 20–24 mm. Similar to male.

*Variation*. There is only slight variation, apart from size. One female has a slender sub-costal line near base. Worn specimens become lighter.

*Male genitalia* (Figs 261–262). Uncus semi-oval, hardly demarcated from tegumen, about one-third shorter than maximum width, basally broad, evenly tapered to broadly rounded apical margin; gnathos hook moderately stout, apically pointed, about length of uncus, evenly curved; tegumen with broad and moderately shallow anterior emargination, additional emargination medially, with sclerotised edge; pedunculi small, suboval; valva long and moderately slender, extending slightly beyond apex of uncus, basally widened, distal part tapered, apical quarter weakly curved and contorted, setose; saccular area densely covered with setae, without separated sacculus; posterior margin of vinculum with small emargination, distinct lateral hump, vincular sclerites largely reduced, oblique sclerotised ridge; saccus massive, stout, broadly V-shaped with irregularly tapered distal part, slightly shorter than valva, ratio maximum width to length approximately 0.7, posterior margin shallow incised medially, with weakly sinusoid mediolateral humps, medial part with sclerotised ridges parallel to outer edge, lateral sclerites approximately 0.7 times maximum width of saccus; phallus massive, without special sclerotisations, largely inflated coecum, medial part constricted, sub-apical third with broadly sinusoid dorsal margin.

*Female genitalia* (Figure 303). Papilla analis large, apically rounded, slightly longer than segment VIII; apophysis posterior rod-like, posteriorly bordered by large sclerotised field, approximately 1.2 mm long, posteriorly weakly widened at about one-quarter, apex rounded; segment VIII approximately 0.6 mm long, posteriolaterally sclerotised, medially membranous; subgenital plate with sclerotised zone, from apophysis anterior extended sub-medially at anterior part, abruptly tapered to oblong and pointed sub-medial sclerite, almost extended to posterior edge of segment VIII, demarcating elongated suboval ostium bursae, surrounded by smoothly sclerotised zone with suboval anterior margin; apophysis anterior rod-like, maximum length of segment VIII; colliculum short, sclerotised; ductus bursae slender, approximately 2.5 mm long; corpus bursae approximately 1.8 mm long, broadly suboval, clearly delimited from ductus bursae; signum a small irregularly shaped spiny plate, in posterior part of corpus bursae.

##### Diagnosis.

*Megacraspeduspeyerimhoffi* is characterised by its large wingspan and its elongate, greyish brown forewing without stripes or markings, apart from the slender white streak on the costa. It is often collected together with the somewhat similar to *M.squalida* (Figs 128–129), which has segment 2 of the labial palp broader and the veins on the forewing lighter. The male of the latter can also be recognized by the thickened, black antennae. It is furthermore similar to *M.violacellum* (p 157). The male genitalia are unmistakable and easily recognized by characters such as the shape of the uncus and the long valva without a sacculus, the latter character separating the species from members of the *M.quadristictus* species group. The female genitalia are similar overall to several species of *Megacraspedus*, but a combination of strutures such as the large and rounded papilla analis, the very long sub-medial sclerite of segment VIII and the short apophysis posterior and anterior are diagnostic.

##### Molecular data.

BIN BOLD:ACC5030 (n = 4). The intraspecific divergence of the barcode region is moderate with mean 0.6% and maximum divergence of 0.9%. The distance to the nearest congeneric neighbour *M.skulei* sp. n. is 9.5% (p-dist).

##### Distribution.

Algeria, Spain.

##### Biology.

The larva feeds within the lower part (above the earth surface) of a stem of *Macrochloatenacissima* (L) Kunth (Poaceae), eating the pith and thereby hollowing the stem. It spins 4–7 opercula of silk within its mine. When fullfed it lines a chamber in the bottom of the mine with silk, gnaw an exit hole and pupates in the mine. The pupa is standing head upwards on excrements which are pressed together in the bottom of the mine. The exit hole is placed so that it is covered by a leaf sheat, which the hatching adult has to slide between to reach the outside (Le Cerf 1925: 21–24).

Although Le Cerf (op. cit.: 21) stated that his observations on the behavior of the larvae of *M.peyerimhoffi* were incomplete, they are the most detailed given for any *Megacraspedus* species. The adults have been collected from early September to the beginning of November at altitudes from sea level to 1300 m.

##### Remarks.

*Megacraspeduspeyerimhoffi* was described from one male and four females bred from larvae and pupae collected by M. de Peyerimhoff in the region of Bouira-Sahari (=Had sahari), northern Algeria (Le Cerf 1925). We have been able to examine photographs of the male syntype from MNHN. This specimen is here designated as the lectotype in order to fix the identity of the species and conserve stability of nomenclature.

### *Megacraspeduspeslieri* species group

The *M.peslieri* species group includes one species: *M.peslieri* sp. n.

External morphology. See species description.

Genitalia morphology. Male genitalia. See species description.

Diagnosticremarks. The *M.peslieri* species group is defined by a combination of characters such as the short and almost knife-shaped valva, the massive and smoothly sclerotised saccus without a medial ridge, and in particular the long thorn of the phallus extending from the middle to the apex which is a unique structure.

The systematic position of the *M.peslieri* species group is uncertain and tentative due to the absence of females and supportive molecular data.

#### Megacraspedus peslieri sp. n.

Taxon classificationAnimaliaLepidopteraGelechiidae

http://zoobank.org/2037689C-F174-4E2A-90FB-511074215159

##### Examined material.

**Holotype** ♂, “France Pyr.[enees]-Or.[ientales] Conat (Bel Loc) 13-IX-2017 880 m Peslier leg.” “P. Huemer GEL 1274 ♂” (TLMF). **Paratypes.** France. 2 ♂, same data as holotype, but genitalia slide 32197 Nel (MHNT, RCSP). Spain. 1 ♂, prov. Huesca, Peñalba, 250 m, 17.x.1984, leg P. Stadel Nielsen, genitalia slide 5304 O. Karsholt (ZMUC).

##### Description.

Adult. *Male* (Figure 147). Wingspan 13 mm. Segment 2 of labial palpus with long scale brush, dark brown on outer surface, white mottled with brown on inner surface, white on lower and upper surface; segment 3 cream-white, blackish brown at base and apical half. Antennal scape without pecten; flagellum ringed black and brown. Head and thorax whitish brown; tegula as forewing. Forewing cream coloured, darker on costa, lighter on dorsum, more or less mottled with yellow and brown- and black-tipped scales; basal part of costa black; a black dot at end of fold; black dots at 3/5 in middle of wing and at end of cell; termen with black scales; fringes grey, yellowish at apex. Hindwing grey with grey fringes.

*Female*. Unknown.

*Variation*. The specimen from Spain has a black streak in the fold.

*Male genitalia* (Figure 263). Uncus broadly sub-rectangular, slightly longer than broad, apical margin evenly rounded; gnathos hook moderately slender, apically pointed, approximately 1.4 times length of uncus, bent at right angles at about one-third; tegumen with broad and moderately deep anterior emargination, additional weak emargination medially; pedunculi suboval; valva with short digitate part, extending to about middle of uncus, at most half width of uncus, basally widened, ventral edge straight, dorsal edge weakly curved, distal part weakly tapered, with rounded apex; saccular area covered with some setae, without separated sacculus; posterior margin of vinculum with shallow emargination, weakly curved lateral hump, vincular sclerites oblong, with posteriomedial ridge; saccus large, stout, broadly U-shaped, distal abruptly tapered to weakly pointed apex, ratio maximum width to length approximately 0.9, posterior margin weakly produced, with medial emargination, medial part smooth, without sclerotised ridge, lateral sclerites approximately 0.6 times maximum width of saccus, with dilated distal end; phallus long, coecum weakly inflated, distal four-fifths, slender, straight, digitate, with ventral sclerotised ridge, dorsal part with long and slender thorn-like sclerite from about middle nearly to apex, parallel to phallus axis, apex weakly excavated.

*Female genitalia*. Unknown.

##### Diagnosis.

*Megacraspeduspeslieri* sp. n. is characterised by the cream-brown forewings with a black streak through the fold and a black dot at end of cell. It is similar to *M.binotella* (Figs 62–63), but that species has more distinctly ringed antennae and no black streak in the fold. The species shows some similarities to other species with a sub-rectangular uncus in the male genitalia but is unmistakable due to the unique phallus, the short valva, and the large saccus.

##### Molecular data.

Not available, barcoding failed.

##### Distribution.

Southern France (Pyrénées Orientales), northern Spain.

##### Biology.

Host plant and early stages are unknown. The small type-series was collected from the middle of September to mid-October at altitudes of between 250 and 880 m.

##### Etymology.

The species name (a noun in the genitive case) is dedicated to Serge Peslier, France, who collected most specimens.

### *Megacraspedusgrisea* species group

The *M.grisea* species group includes three species: *M.grisea*, *M.pacificus* sp. n., and *M.armatophallus* sp. n.

The *M.grisea* species group is defined by following characters:

External morphology. Segment 2 of labial palpus with scale brush as long as or longer than segment 3; segment 3 as long as or shorter than segment 2. Antennal scape with a single hair. Wingspan (males) 15–16 mm. Forewing with 2–3 black spots, but without whitish veins and costa. Known female brachypterous.

Genitalia morphology. Male genitalia. Uncus moderately sub-rectangular, with broadly curved or indented apex; gnathos hook stout, sub-basally bent, with pointed apex; valva massive, short and broad, with proximal hump basally, distally tapered, with distinct flap-shaped ridge in distal half; saccular area densely covered with setae, without separated sacculus; saccus massive, V-shaped; phallus stout, straight, with weakly inflated coecum, distal two-thirds moderately broad, with two rod-like, weakly dentated sclerotisations or with prominent, weakly dentated thorn sub-basally.

Female genitalia. Papilla analis small, apically rounded, largely membranous; apophysis posterior long and slender; segment VIII weakly sclerotised to membranous; subgenital plate with large subostial sclerotisation, sub-triangular process from base of apophysis anterior pointed anteromedially, and with posteromedially pointed process, demarcating lateral part of ostium bursae by deeply sclerotised wall; apophysis anterior rod-like, posterior part with longitudinal sclerotised zone along entire segment VIII; colliculum short; signum a small, transverse, spiny plate.

Diagnosticremarks. The *M.grisea* species group is defined by several structures in the male genitalia, in particular the broad valva with a transverse scleorized ridge or flap, the large V-shaped saccus with a weakly developed longitudinal ridge and in particular the phallus with a prominent, weakly dentated sub-basal thorn or two rod-like, weakly dentated sclerotisations. The female genitalia are in particular characterised by the shape of the subostial sclerotisation of segment VIII and the longitudinal sclerotised zone venula of segment VIII. However, it has to be acknowledged that females are unknown in several other species groups.

#### Megacraspedus grisea

Taxon classificationAnimaliaLepidopteraGelechiidae

(Filipjev, 1931) comb. n.

Reichardtiella grisea Filipjev, 1931: 168, fig. 16, pl. 10, figs 5–7.

##### Examined material.

**Syntype** ♂, [China] “*Reichardia grisea* Flj. N. Filipjev det. [partially illegible]” “Para-lecto-type” “Paralectotype *Reichardtiella grisea* Filipjev teste. K. Sattler, 1978” “Alai valley, loc. Sary-tash Reihardt 27.VI.928 [transcribed from Cyrillic]” “Pres. by Zool. Inst. Leningrad B.M. 1978-417” “BMNH ♂ genitalia slide No. 33663” (BMNH).

##### Redescription.

Adult. *Male* (Figure 148). Wingspan 15 mm. Segment 2 of labial palpus with long ventral brush of scales extending to about same level as tip of third segment, dark grey-brown with some whitish mottling, particularly on inner surface of brush and on dorsal part of segment; segment 3 about length of second segment, creamy white with some darker mottling. Antennae ringed dark grey-brown and whitish. Head, thorax and tegulae dark grey-brown with a few whitish scales. Forewing dark grey, black at basal half of costa; an indistinct black dot in fold and at end of cell; termen with black and lighter scales; fringes grey. Hindwing dark grey with grey fringes.

*Female*. Unknown.

*Variation*. Unknown.

*Male genitalia* (Figure 264). Uncus moderately broad, approximately 1.5 times longer than wide, sub-rectangular, with broadly curved apex; gnathos hook stout, about length of uncus, weakly curved with pointed apex; anterior margin of tegumen with small excavation, pedunculi moderate, rounded; valva massive, short and broad, with proximal hump basally, distally tapered, extending to base of gnathos, outer margin weakly curved, inner margin almost straight, apex rounded, valva with distinct flap-shaped ridge in distal half; saccular area densely covered with setae, without separated sacculus; posterior margin of vinculum with moderate medial emargination, lateral humps merged with base of valva; saccus massive, V-shaped with moderately broad apical part; phallus stout, straight, with weakly inflated coecum, distal two-thirds moderately broad, with two rod-like, weakly dentated sclerotisations.

*Female genitalia*. Unknown.

##### Diagnosis.

*Megacraspedusgrisea* is characterised by the almost plain dark grey forewings. Externally it mostly resembles *M.fuscus* sp. n. (p 67). The male genitalia are unmistakable in *Megacraspedus*, in particular due to the characteristic shape of the valva and the dentation of the phallus.

##### Molecular data.

Not available, available specimens too old.

##### Distribution.

Western China (Pamir Mountains).

##### Biology.

Host plant and early stages are unknown. The typeseries was collected on 8^th^ July at an altitude of 4300 m (Filipjev 1931).

##### Remarks.

*Reichardtiellagrisea* was described from several males in variable condition collected at Sary-Kol in the Chinese part of the Pamir Mountains at an altitude of 4300 m (Filipjev 1931).

#### Megacraspedus pacificus sp. n.

Taxon classificationAnimaliaLepidopteraGelechiidae

http://zoobank.org/7A5742E8-6E2F-4268-83C6-4BF9D2BE5A6F

##### Examined material.

**Holotype** ♂, “29.VII.1963 Afghan.[istan] centr., O v [East of] Band-i-Amir, 3600 m Kasy & Vartian leg.” “Gen. Präp. Mus. Vind. 16.661 ♂” (NHMW). **Paratype.** Afghanistan. 1 ♀, prov. Bamyan, Band-i-Amir, 3000 m, 30.vii.1963, leg. Kasy & Vartian, genitalia slide Mus. Vind. 16.662 (NHMW).

##### Description.

Adult. *Male* (Figure 149). Wingspan 15 mm. Segment 2 of labial palpus with long scale brush, blackish brown on outer surface, white mottled with brown on inner surface, white on lower and upper surface; segment 3 white with black tip. Antennal scape with a single pecten; flagellum indistinctly ringed black and grey. Head, thorax and tegula light grey-brown. Forewing bone white mottled with grey and brown, especially at base and along veins and wing margins; margin of costa white; black dots in fold at 2/5, and at 3/5 in middle of wing and at end of cell; scattered black scales forming an interrupted line along termen; fringes light grey. Hindwing grey with light grey fringes.

*Female* (Figure 150). Wingspan 12 mm. Antenna ringed black and white. Head, thorax and tegula white. Forewing slightly ellipsoid, white, slightly mottled with brownish and black scales, especially along veins; a black subcostal spot at one-quarter. Hindwing reduced in width and with extended apex. Otherwise similar to male.

*Variation*. None from the few examined specimens.

*Male genitalia* (Figure 265). Uncus sub-rectangular, slightly longer than broad, distally weakly produced with evenly rounded apex; gnathos hook moderately slender, apically pointed, approximately 1.5 times length of uncus, bent at right angles at about one-third; tegumen with broad and shallow anterior emargination, anteriolaterally with short sclerotised ridges merged near middle of tegumen; pedunculi small, suboval; valva stout, extending slightly beyond base of uncus, basally broad, distal part with sclerotised setose ridge, apex shovel-shaped, distorted; saccular area densely covered with setae, without separated sacculus; posterior margin of vinculum shallow, with curved lateral hump; saccus stout, V-shaped, about length of valva, posterior margin hardly emarginated, with indistinctly sinusoid mediolateral humps, medial part with sclerotised ridge from posterior margin to middle of saccus, lateral sclerites slightly exceeding maximum width of saccus; phallus with weakly inflated coecum, distal three quarters slender, sub-basally with a prominent, weakly dentated thorn, medially covered with about a dozen short spinules.

*Female gen*italia (Figure 304). Papilla analis small, apically rounded, largely membranous; apophysis posterior slender rod-like, about 3 mm long, posterior end bordered by small sclerotised field; segment VIII approximately 0.7 mm long, weakly sclerotised to membranous; subgenital plate with large subostial sclerotisation delimiting ostium bursae, sub-triangular process from base of apophysis anterior pointed anteromedially and delimiting anterior margin of segment VIII, and posteromedially pointed process, demarcating lateral part of ostium bursae by deeply sclerotised wall; apophysis anterior rod-like, anterior part of 1 mm length, venula a longitudinal sclerotised zone along entire segment VIII; colliculum short, weakly sclerotised; ductus bursae slender, widened to weakly delimited corpus bursae; entire length of ductus and corpus bursae approximately 2.6 mm; signum a small, transverse, spiny plate.

##### Diagnosis.

*Megacraspeduspacificus* sp. n. is characterised by its light greyish brown forewings with three weak black dots and an interrupted black line along the termen. It is similar to *M.imparellus* (Figs 100–102) which has slightly broader and lighter forewings with more distinct black dots. This species and *M.pacificus* sp. n. have a single pecten on the antennal scape, whereas the somewhat similar *M.leuca* (Figs 111–112) has several hairs on the antennal scape. See also *M.armatophallus* sp. n. (see below) and *M.majorella* (p 124). The shape of the valva and the characteristic thorn of the phallus are unmistakable diagnostic characters in the male genitalia. The female genitalia are similar to *M.armatophallus* sp. n. but differ in particular in the much smaller and weakly sclerotised colliculum.

##### Molecular data.

Not available, available specimens too old.

##### Distribution.

Central Afghanistan (prov. Bamyan).

##### Biology.

Host plant and early stages are unknown. The adults have been collected at the end of July at high altitudes from 3000 to 3600 m.

##### Etymology.

The species name is derived from the Latin word *pacificus*, in the sense of bringing peace to the type locality of Afghanistan.

#### Megacraspedus armatophallus sp. n.

Taxon classificationAnimaliaLepidopteraGelechiidae

http://zoobank.org/B7608C4F-5DDD-4BC5-BE3F-941158896229

##### Examined material.

**Holotype** ♂, “SO-AFGHANISTAN Safed Koh, S-Seite Shahidan, 2700 m 21.6.1966 H. G. Amsel leg.” “SMNK” “GU 17/1497 ♂ P. Huemer” (SMNK). **Paratypes.** Afghanistan. 1 ♂, 1 ♀, same data as holotype (SMNK).

##### Description.

**Figures 151–152.**
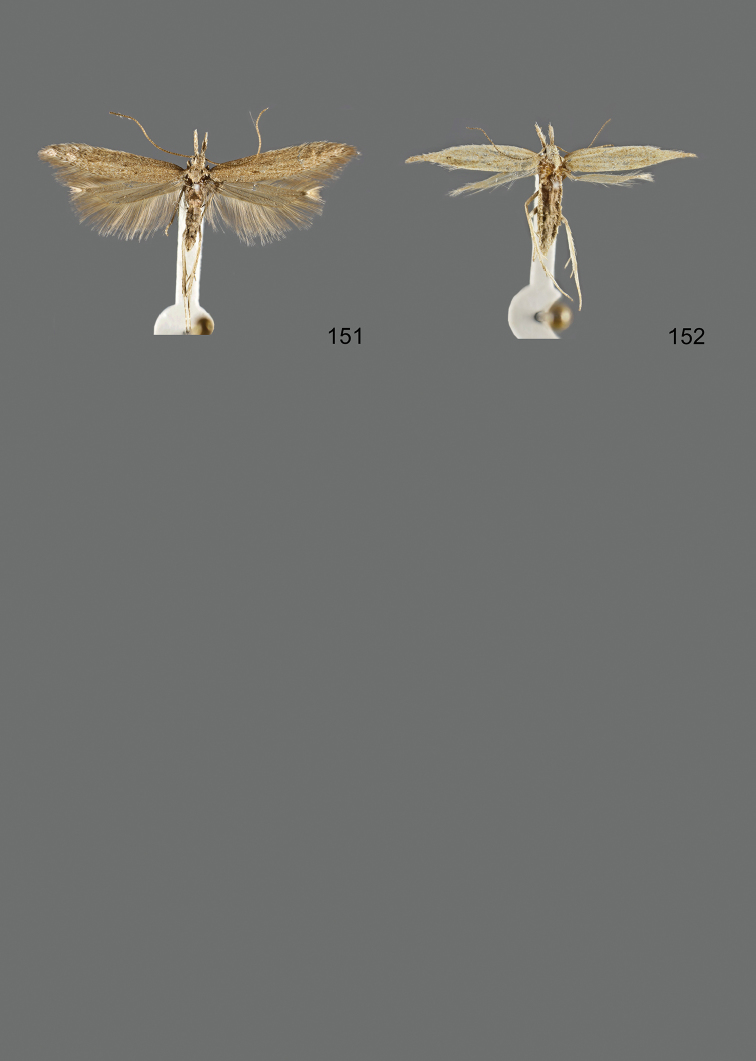
*Megacraspedus* adults in dorsal view. **151***M.armatophallus* sp. n. – Paratype, male, Afghanistan (LNK) **152***M.armatophallus* sp. n. – Paratype, female, Afghanistan (LNK).

Adult. *Male* (Figure 151). Wingspan 16 mm. Segment 2 of labial palpus with scale brush longer than segment 3, brown on outer and inner surface, white mottled with brown on upper and lower surface; segment 3 shorter than segment 2, whitish at base, black towards tip. Antennal scape with a single pecten; flagellum brown, indistinctly ringed with light grey. Head thorax and tegula as forewing. Forewing greyish brown, dark grey at base of costa; weak black dots in fold at 2/5, and at 3/5 in middle of wing and at end of cell; scattered black scales forming an interrupted line along termen; fringes grey. Hindwing grey with grey fringes.

*Female* (Figure 152). Wingspan 15 mm. Antenna ringed dark brown and white. Head, thorax and tegula cream coloured, mottled with some brown. Forewing with apical third tapered to pointed apex, light brown. Hindwing about one-third width of forewing with lanceolate apex. Otherwise similar to male.

*Variation*. The female has no pecten on the antennal scape, but as it is rather worn such may have been lost.

*Male genitalia* (Figure 266). Uncus sub-rectangular, slightly longer than broad, apical margin with distinct medial emargination; gnathos hook moderately stout, apically pointed, slightly longer than uncus, weakly curved at about one-third; tegumen with broad and moderately shallow anterior emargination, additional weak emargination medially, with short sclerotised ridges merged near middle of tegumen; pedunculi small, suboval; valva massive, extending nearly to apex of uncus, basally widened, broad distal part with sclerotised ridge, apex rounded, setose; saccular area densely covered with setae, without separated sacculus; posterior margin of vinculum with U-shaped emargination, weakly curved lateral hump; saccus stout, V-shaped, about length of valva, posterior margin hardly emarginated, with indistinctly sinusoid mediolateral humps, medial part with sclerotised ridge from posterior margin nearly to tip of saccus, lateral sclerites slightly shorter that maximum width of saccus; phallus with weakly inflated coecum, distal three-quarters slender, dorsobasally with prominent, smooth thorn, medially short ridge with small spinules, distal part strongly broadened, apically rounded, distoventrally convex with broad tooth-like projection.

Female genitalia (Figure 305). Papilla analis small, apically rounded, largely membranous; apophysis posterior slender rod-like, approximately 3.6 mm long, posterior end bordered by small sclerotised field; segment VIII approximately 0.7 mm long, weakly sclerotised to membranous; subgenital plate with large subostial sclerotisation delimiting ostium bursae, sub-triangular process from base of apophysis anterior pointed anteromedially and delimiting anterior margin of segment VIII, and posteromedially pointed process, demarcating lateral part of ostium bursae by elongated, moderately deep sclerotised wall; apophysis anterior rod-like, approximately 1.2 mm long, sclerotised zone of venula along entire segment VIII; colliculum medium-sized, strongly sclerotised; ductus bursae slender, abruptly widened to moderately delimited corpus bursae; entire length of ductus and corpus bursae approximately 3.2 mm; signum a small, transverse, spiny plate.

##### Diagnosis.

*Megacraspedusarmatophallus* sp. n. is characterised by the greyish brown forewings with three weak black dots and an interrupted black line along the termen. It is similar *M.pacificus* sp. n. (Figs 149–150) which has lighter forewings with the costal margin white and more distinct black dots. The shape of the uncus, the broad valva, and the phallus with a prominent thorn and a unique distal part are unmistakable diagnostic characters in the male genitalia. The female genitalia largely resemble those of *M.pacificus* sp. n. (Figure 304) but differ in characters such as the distinctly larger and strongly sclerotised colliculum.

##### Molecular data.

Not available, available specimens too old.

##### Distribution.

South-eastern Afghanistan.

##### Biology.

Host plant and early stages are unknown. The small type series was collected in the last third of June at altitudes of ca. 2700 m.

##### Etymology.

The species name is derived from a combination of the Latin words *armatus* (meaning armed warrior) and *phallus*, indicating the weapon-like shape of the phallus.

## Discussion

### Generic definition

The phylogeny of numerous Gelechiidae genera is still far from resolved and this also applies to *Megacraspedus*. The first comprehensive molecular study of the family by Karsholt et al. (2013) provides strong evidence for a well-supported suprageneric order. However, the generic delimitation is much less straightforward, particularly due the lack of a larger taxon coverage. Like other genera, only a single species of *Megacraspedus*, *M.dolosellus*, was considered in the aforementioned study, reflecting the general lack of molecular data and analysis at the generic level. The implementation of DNA barcoding has helped to reduce such shortcomings in the Gelechiidae for a few genera so far (Huemer and Hebert 2011, Huemer et al. 2014) but restriction to the mitochondrial COI gene limits the value for phylogenetic analysis. Molecular studies at a generic level, based on a larger number of nuclear genes, are completely lacking, not least because of the limitation and quality of available voucher material and the general lack of resources. The generic definition of *Megacraspedus* is thus based only on morphological features and DNA barcodes, but monophyly of the genus needs evidence from further in-depth studies based on several markers. With more than 50% of undescribed species, even from Europe, *Megacraspedus* is a poorly explored genus of Lepidoptera in the Palearctic. We are therefore convinced that the need for an updated alpha taxonomy justifies our revisionary work despite the general problems on a generic scale.

### Species delimitation

The correct identification of Gelechiidae to species level is a difficult task, even in some already revised genera. Characters traditionally used to delineate taxa are mainly found in the genitalia and external morphology, but the degree of diagnostic features varies from group to group. In several genera even the genitalia morphology is only of limited value for species delimitation, as shown in a monographic review of Palearctic Gnorimoschemini (Povolný 2002). Similar problems have been observed in *Megacraspedus*. Widespread external variation in adults and the general lack of fresh material is a critical obstacle in identification of many species of *Megacraspedus* on phenotypical characters. In contrast, male genitalia structures usually provide the most reliable diagnostic characters for species delimitation, whereas female genitalia are known for only about two-fifths of the species and thus are of limited value for identification. Furthermore, diagnostic morphological characters are subtle in several species and moreover individual variation is insufficiently known. Fortunately, the supplementary use of DNA barcoding for species delimitation proved extremely helpful. The intraspecific divergence in the majority of species is low with mean 0.9%, whereas interspecific divergence to the nearest neighbour is much higher with mean 7.9% and minimum 2.3% (Figure 6) and therefore supporting species delimitation. However, as exemplified in a few species, these molecular data also have to be considered with due care. In some taxa, DNA barcodes can give a strongly misleading impression of the number of putative species. Particularly in *M.lanceolellus* and in *M.dolosellus* an extraordinary level of intraspecific barcode divergence was observed with 19 and 23 BINs, or 12.5% and 13.8% maximum divergence respectively. However, even this extraordinary level of DNA barcode variation seems of limited taxonomic relevance, as it is not unambiguously supported by morphology. Genitalia morphology is very similar overall in the species involved, with some individual variation and phenotypic characters, such as wing pattern and colour, also sometimes show intrapopulational and geographic variation. In *M.dolosellus* even the reduction of the female hindwings is variable. Different stages of brachyptery occasionally occur within populations (Altai Mountains), or in two morphologically separated morphs in sympatry but inseparable in DNA barcodes. It will be necessary to resolve these conflicting problems by multi-loci analysis in future (Dupuis et al. 2012).

Large intraspecific barcode divergence as observed in *Megacraspedus* is generally rare in Lepidoptera (Mutanen et al. 2016), but remarkable examples are found, for example, in the genus *Grammia* Rambur (Schmidt 2009). Species of this genus have received special attention from many entomologists, not least because of their attractive external appearance, and thus it was possible to implement an integrative approach combining morphology, wing pattern, ecology, biogeography, and cytochrome oxidase I (*cox1*) sequence data. Accordingly, *Grammiaquenselii* (Paykull, 1793) (Erebidae) shows about 11% maximum intraspecific and 0% minimum interspecific divergence, represented by five haplotypes, which partially overlap with other species. The North American *Grammiawilliamsi* (Dodge, 1871) is even known from 23 haplotypes in the same study. Studies in Gelechiidae genera using DNA barcodes are exceptional and give a somewhat different picture. An analysis of 44 species of *Caryocolum* (Huemer et al. 2014) resulted in a maximum intraspecific divergence from 0% in several species to 6.3%, and in six species the mean divergence was greater than 3%. However, most of these cases represent allopatric cryptic diversity (Huemer pers. obs.) and even low divergences have proven to support delimitation of closely related species of Gelechiidae (Huemer and Mutanen 2012).

Similar to other Gelechiidae genera with brachypterous females (Huemer and Hebert 2011, Huemer et al. 2013), much of the observed DNA barcode pattern in *Megacraspedus* may be explained by allopatric evolutionary scenarios, but in several cases it rather reflects the dimension of intrapopulational barcode divergence with deep sympatric splits, which are in conflict with other characters (Mutanen et al. 2016). The unique dimension of intraspecific barcode divergence in Lepidoptera was probably driven by climatic oscillations during the Quarternary with population isolation and genetic differentiation, boosted by a widespread restriction of the group to mountain habitats, and the limited dispersal due to female flightlessness. The occasional co-occurrence of strikingly different haplotypes but identical morphology may be due to several independent re-colonization events. However, the extraordinary intraspecific barcode divergence in some species may also be due to a much faster evolution rate of the mtDNA than normally observed and expected in Lepidoptera. Otherwise populations with e.g., 10% divergence should have evolved to different morphospecies (Mutanen in litt.). This hypothesis is also supported by the most similar species of some *Megacraspedus* which are completely unrelated taxa, e.g., in the families Limacodidiae or Crambidae. The influence of pseudogenes to barcode variation is likely at most limited, as typical characters such as stop codons and frameshift mutations are not observed. Similarly the possible role of *Wolbachia* infection cannot explain the high barcode diversity as it typically causes haplotype sweeps, leading to a reduction of mtDNA variability. Beside of testing the potential influence of pseudogenes and *Wolbachia* on the DNA barcode diversity in *Megacraspedus* it will be desirable to have further molecular markers, particularly nuclar genes, studied in future. However, the shortcomings of suitable material for this purpose will require strong efforts in recollecting most of the species.

## Conclusions

Lepidoptera is one of the best known insect orders, and in Europe even the smaller so-called Microlepidoptera is considered relatively well known. Even though several new species of Lepidoptera from Europe are found and described every year it is very unusual to have 22 new European species in a single genus. When compared with tropical areas Europe is normally not considered to be a hotspot in biodiversity, but, contrary to general belief, developed and heavily-studied parts of the world are important reservoirs of unknown species, making Europe an unexpected frontier for biodiversity exploration (Fontaine et al. 2010, 2012).

We have examined a vast number of *Megacraspedus* specimens collected during our own fieldwork, and from private and institutional collections. The result is the first comprehensive revision of the genus. A considerable number of the species of *Megacraspedus* dealt with here, both new species and some described long ago, are only know from a few specimens or from one or two localities. It is therefore likely that further species remained unrecognized. It is our hope that the present revision will stimulate lepidopterists to give more priority to *Megacraspedus* and search for both unknown and already known species in new places, as well as for some of the many unknown females, and perhaps investigate the early stages and biology. This may also result in a better understanding of the reasons for the high genetic variation in some species.

## Plates

### Figures of *Megacraspedus* adults in dorsal view

### Figures of *Megacraspedus* male genitalia

**Figures 153–155.**
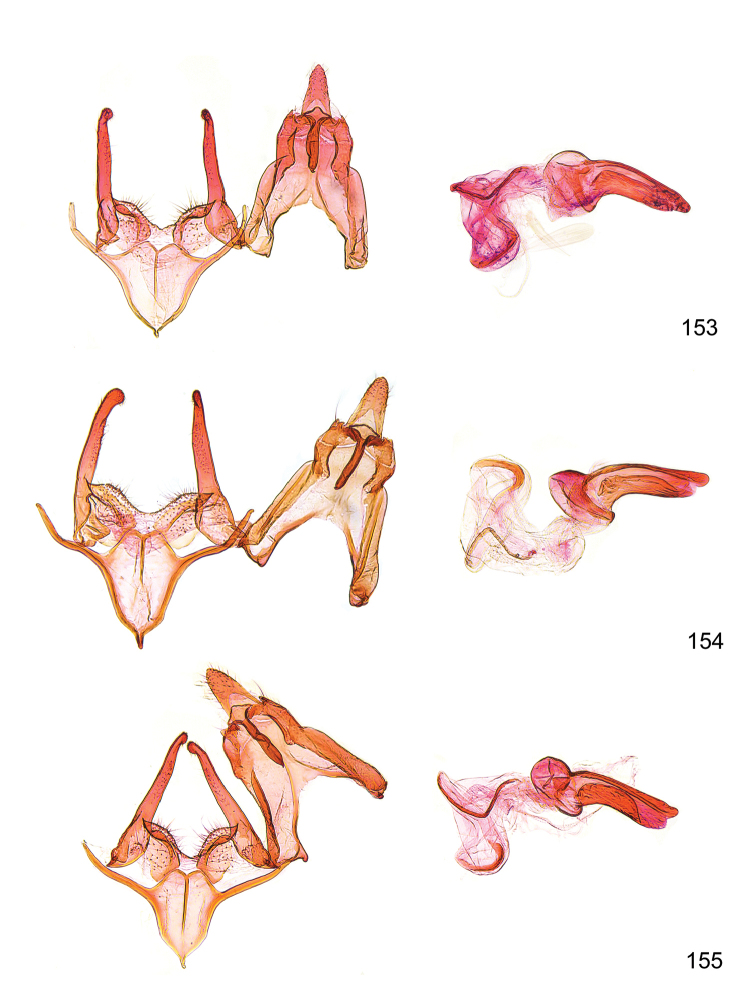
*Megacraspedus* male genitalia. **153***M.lanceolellus* (Zeller, 1850) – Italy, gen. slide 16.653 (NHMW) **154***M.lanceolellus* (Zeller, 1850) – France, GEL 1222 P.H. (TLMF) **155***M.lanceolellus* (Zeller, 1850) – Spain, GEL 1222 P.H. (TLMF).

**Figures 156–158.**
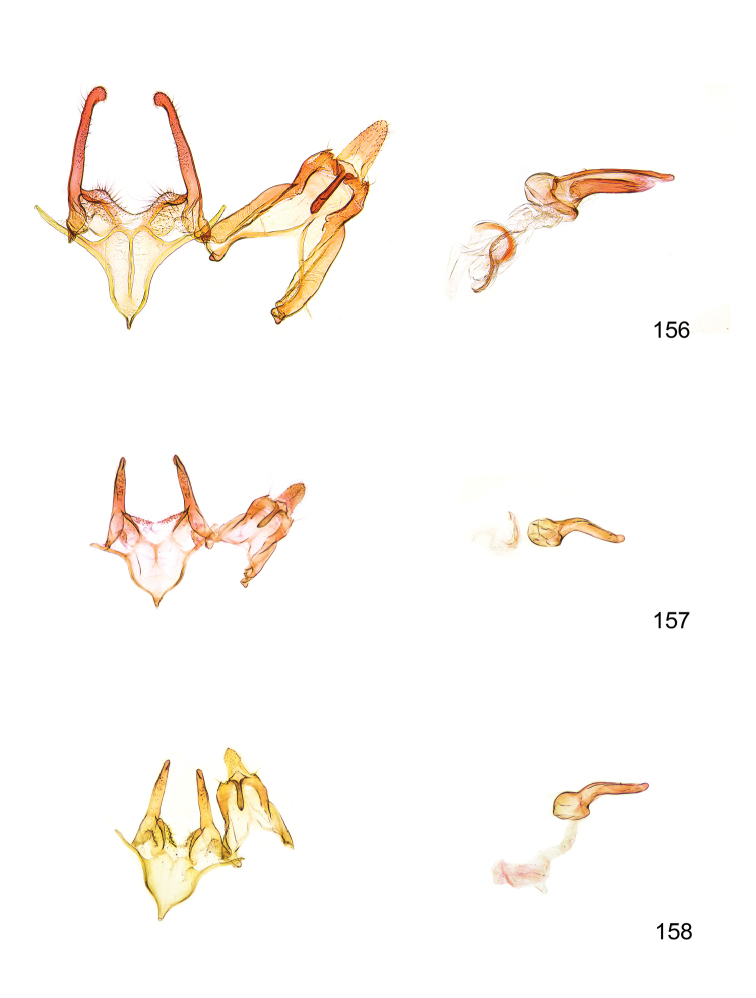
*Megacraspedus* male genitalia. **156***M.lanceolellus* (Zeller, 1850) – Lectotype (*subdolellus*), Spain, GU 15/1404 P.H. (ZMHU) **157***M.bengtssoni* sp. n. – Paratype, Spain, GU 15/1393 P.H. (ZMUC) **158***M.bengtssoni* sp. n. – Paratype, Spain, GU 15/1394 P.H. (ZMUC).

**Figures 159–161.**
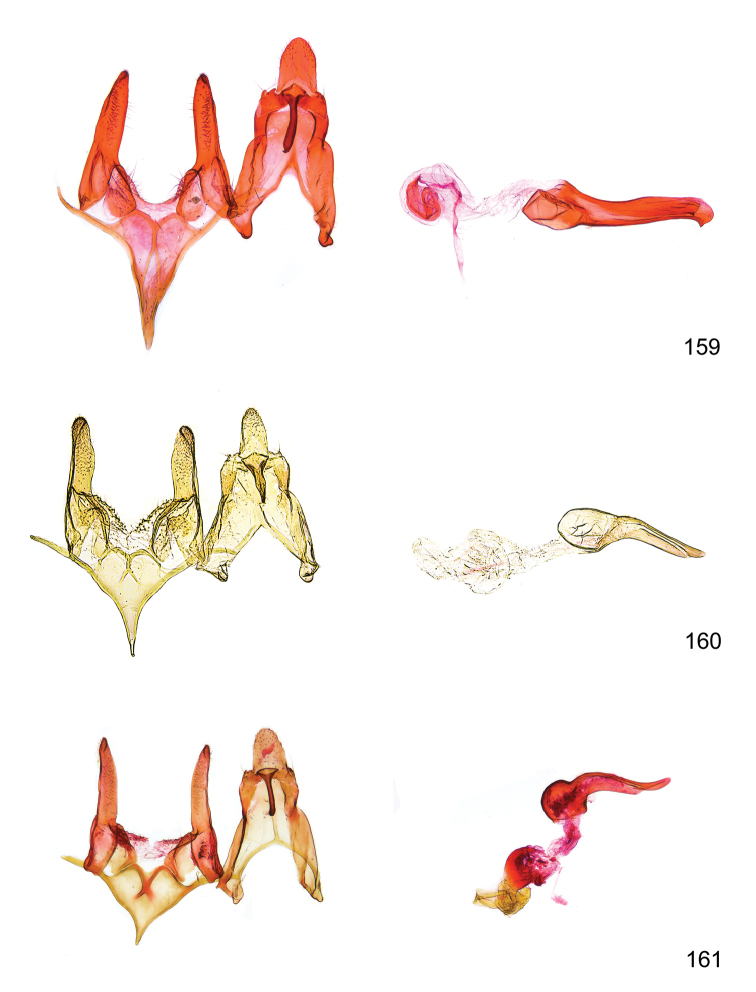
*Megacraspedus* male genitalia. **159***M.homochroa* Le Cerf, 1932 – Morocco, GU 16/1453 P.H. (ZMUC) **160***M.monolorellus* Rebel, 1905 – Lectotype, Turkey, gen. slide 16.649 (NHMW) **161***M.junnilaineni* sp. n. – Holotype, Turkey, GU 16/1448 P.H. (RCJJ).

**Figures 162–164.**
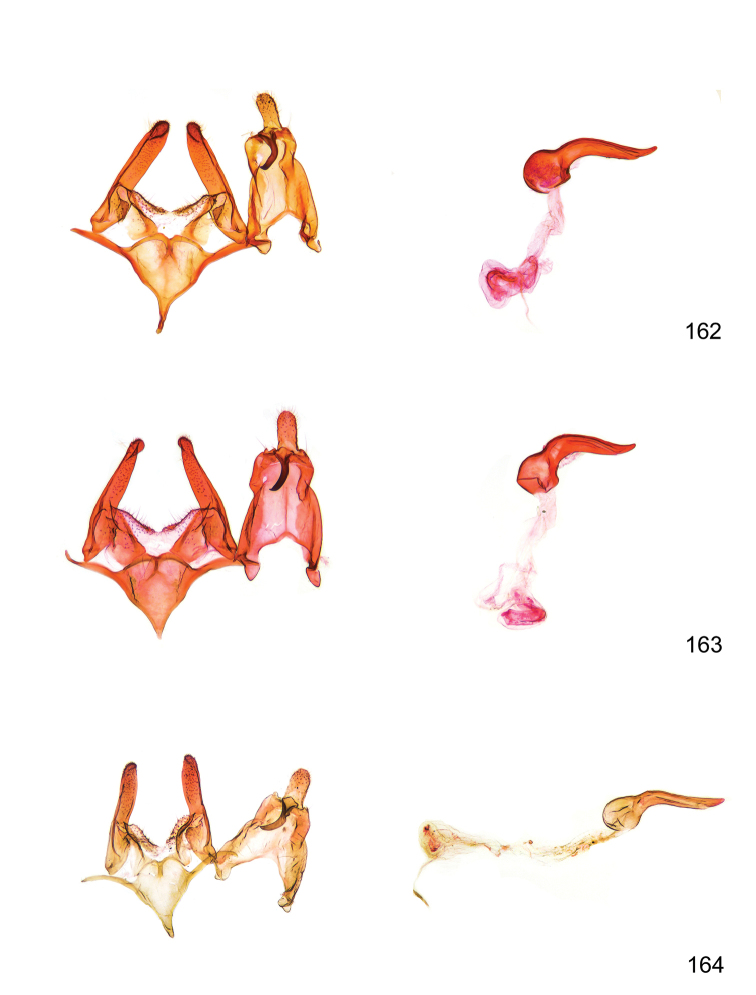
*Megacraspedus* male genitalia. **162***M.uzunsyrtus* Bidzilya & Budashkin, 2015 – Paratype, Ukraine, GU 16/1463 P.H. (ZMKU) **163***M.similellus* sp. n. – Holotype, Bulgaria, GU 16/1449 P.H. (RCJJ) **164***M.similellus* sp. n. – Paratype, Turkey, GU 16/1462 P.H. (RCJJ).

**Figures 165–167.**
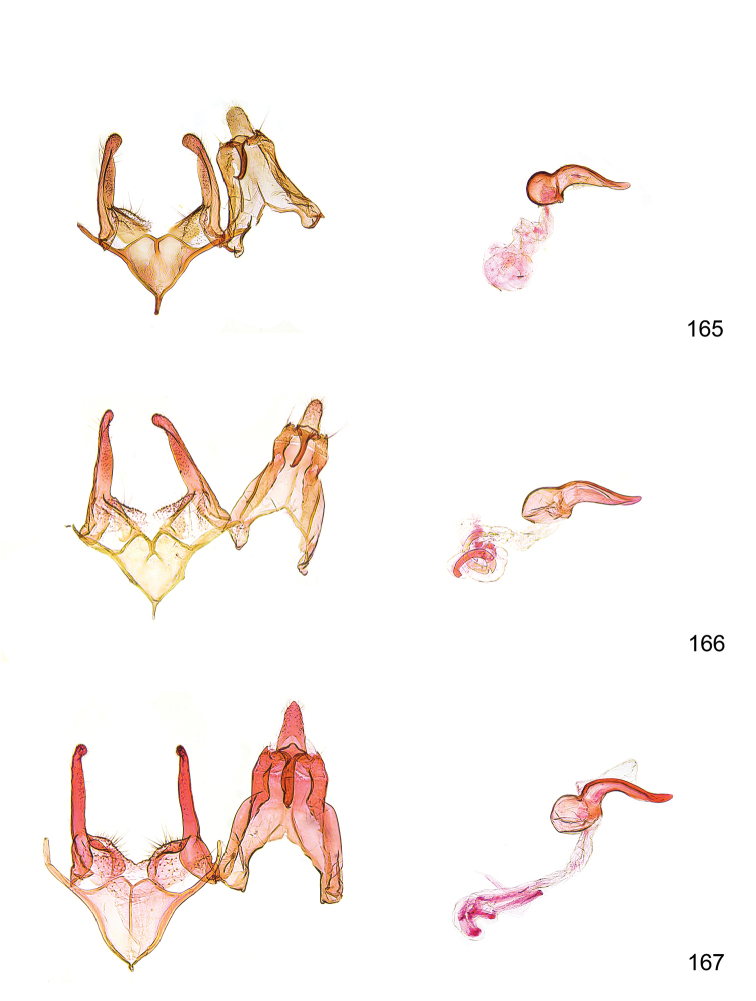
*Megacraspedus* male genitalia. **165***M.golestanicus* sp. n. – Holotype, Iran, GEL 1241 P.H. (TLMF) **166***M.tokari* sp. n. – Paratype, Croatia, GU 14/1384 P.H. (RCZT) **167***M.dolosellus* (Zeller, 1839) – Italy, GU 16/1443 P.H. (ZMUC).

**Figures 168–170.**
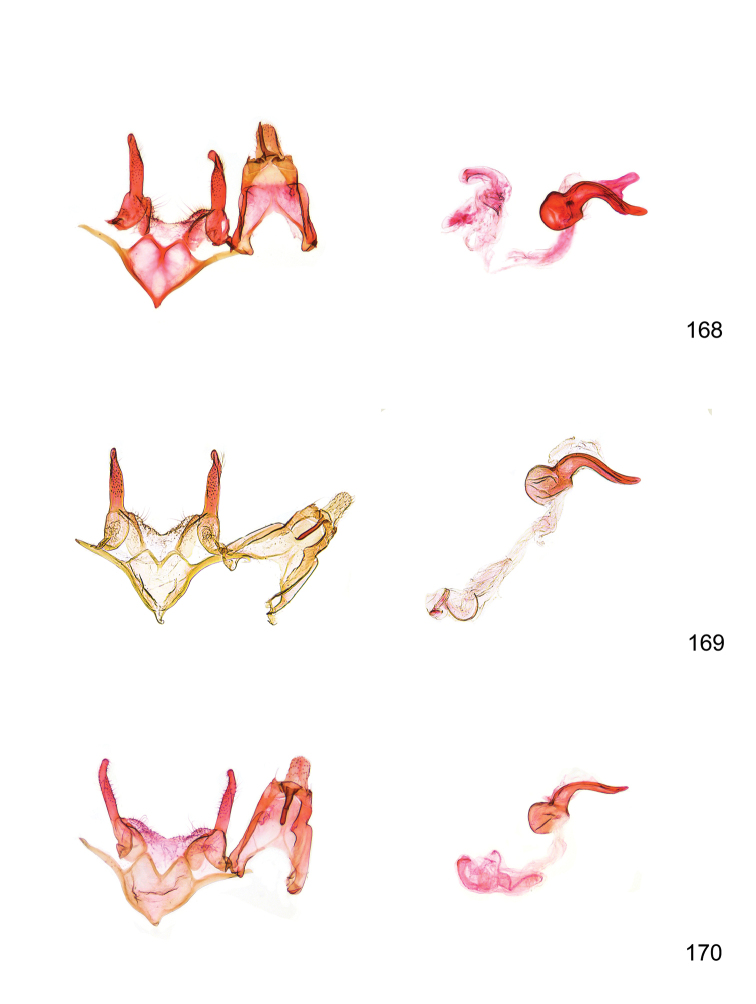
*Megacraspedus* male genitalia. **168***M.dolosellus* (Zeller, 1839) – Bulgaria, GEL 1216 P.H. (TLMF) **169***M.dolosellus* (Zeller, 1839) – Greece, GEL 1242 P.H. (TLMF) **170***M.dolosellus* (Zeller, 1839) – Russia, GEL 1246 P.H. (TLMF).

**Figures 171–173.**
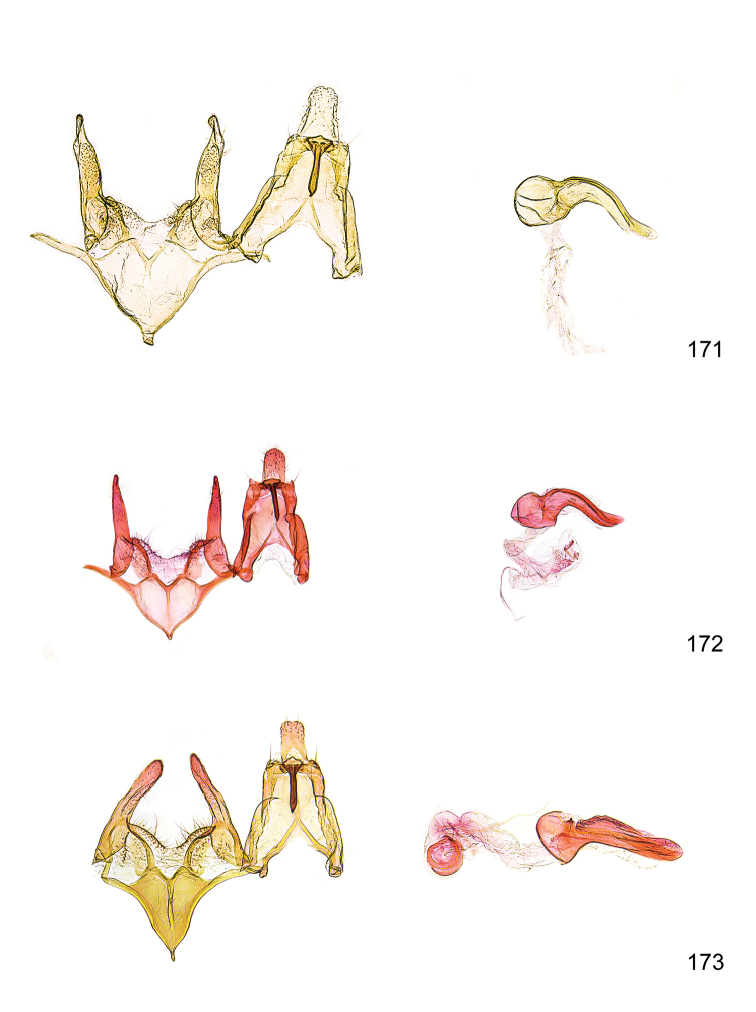
*Megacraspedus* male genitalia. **171***M.dolosellus* (Zeller, 1839) – Lectotype (*incertellus*), Bulgaria, gen. slide 16.518 (NHMW) **172***M.dolosellus* (Zeller, 1839) – Greece, GU 16/1441 P.H. (ZMUC) **173***M.neli* sp. n. – Holotype, France, GEL 1218 P.H. (TLMF).

**Figures 174–176.**
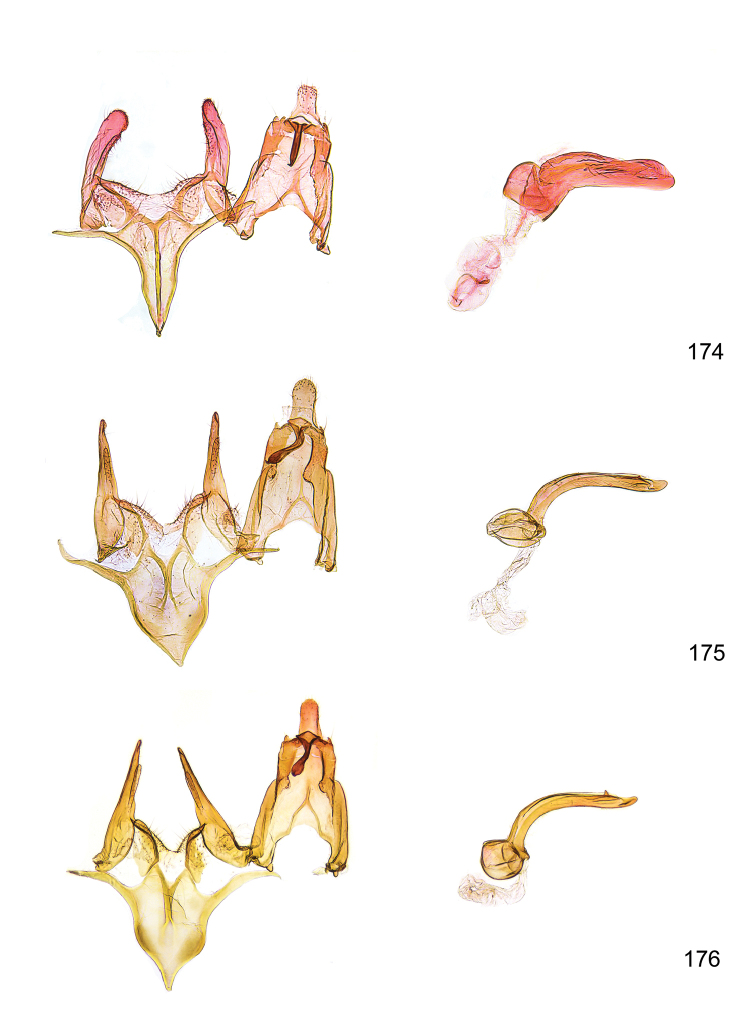
*Megacraspedus* male genitalia. **174***M.faunierensis* sp. n. – Paratype, France, GEL 1219 P.H. (TLMF) **175***M.gredosensis* sp. n. – Holotype, Spain, GU 16/1416 P.H. (RCEA) **176***M.gredosensis* sp. n. – Paratype, Spain, GU 17/1489 P.H.

**Figures 177–179.**
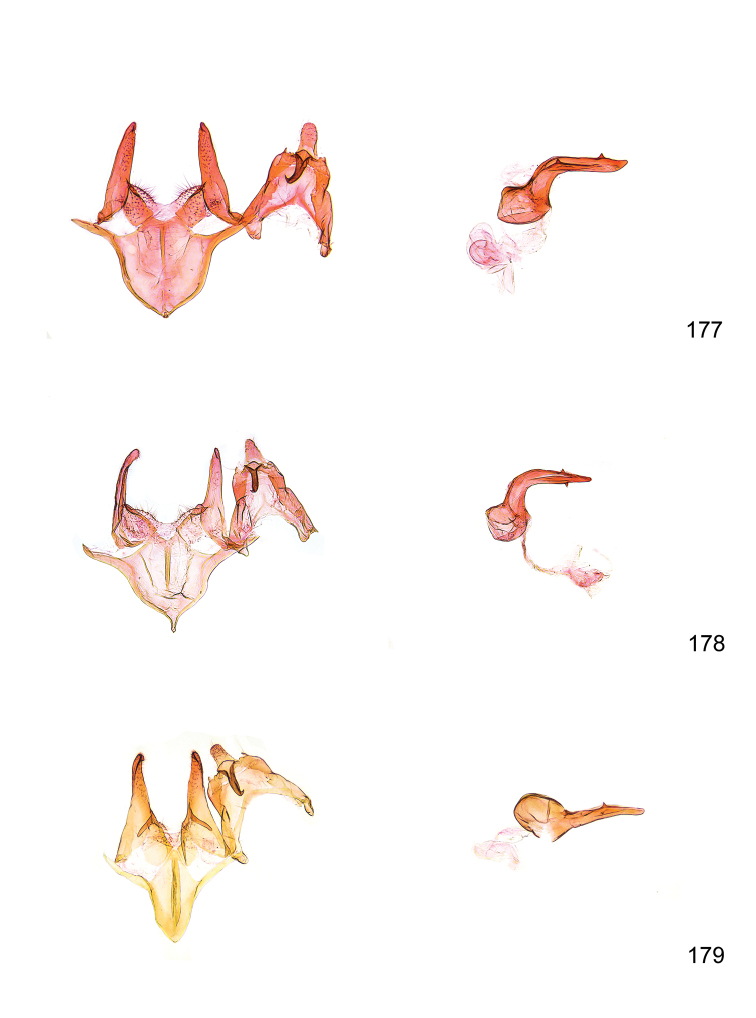
*Megacraspedus* male genitalia. **177***M.cuencellus* Caradja, 1920 – Spain, GU 15/1401 P.H. (ZMUC) **178***M.bidentatus* sp. n. – Holotype, Spain, GU 16/1433 P.H. (ZMUC) **179***M.fuscus* sp. n. – Holotype, Spain, GU 17/1478 P.H. (ZMUC).

**Figures 180–182.**
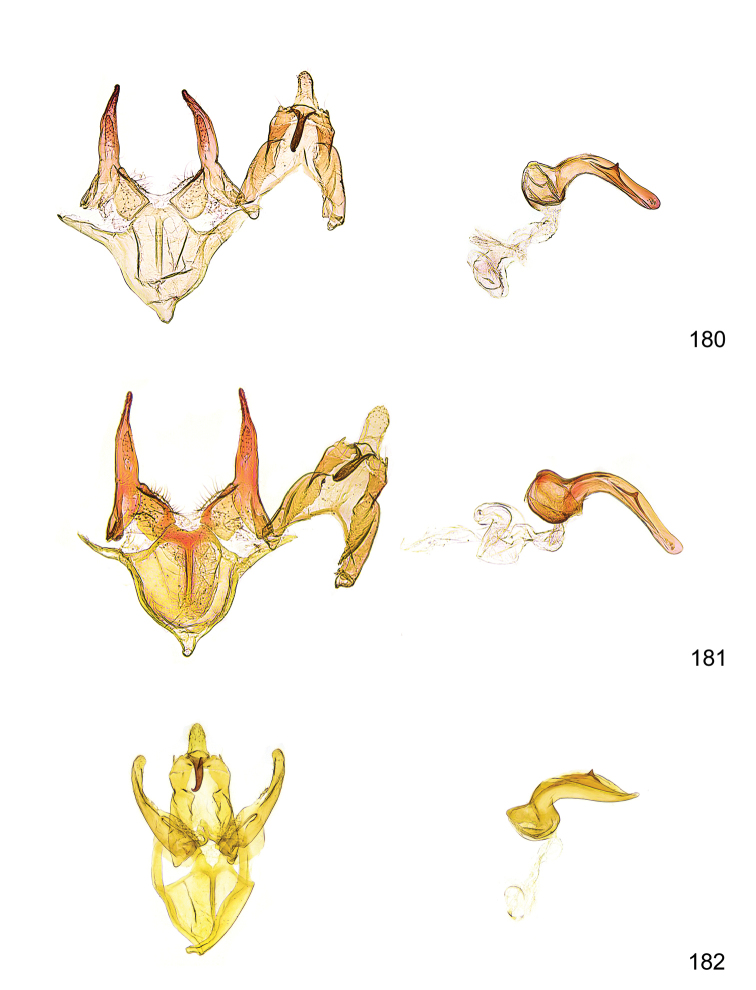
*Megacraspedus* male genitalia. **180***M.trineae* sp. n. – Paratype, Portugal, GEL 1206 P.H. (TLMF) **181***M.trineae* sp. n. – Paratype, Spain, gen. slide 16.655 (NHMW) **182***M.cf.trineae* – Spain, gen. slide 5014 J. Tabell (ZMUC).

**Figures 183–185.**
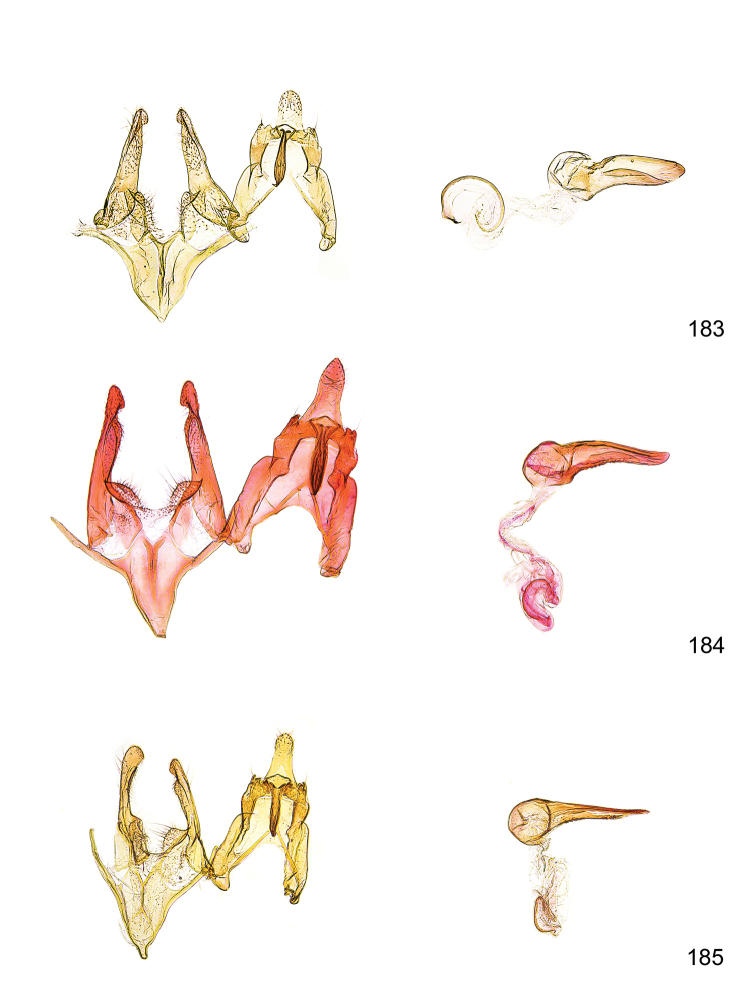
*Megacraspedus* male genitalia. **183***M.tristictus* Walsingham, 1910 – France, GEL 1185 P.H. (TLMF) **184***M.alfacarellus* Wehrli, 1926 – Holotype, Spain, GU 16/1414 P.H. (NHMB) **185***M.alfacarellus* Wehrli, 1926 – Spain, GU 15/1403 P.H. (ZMUC).

**Figures 186–188.**
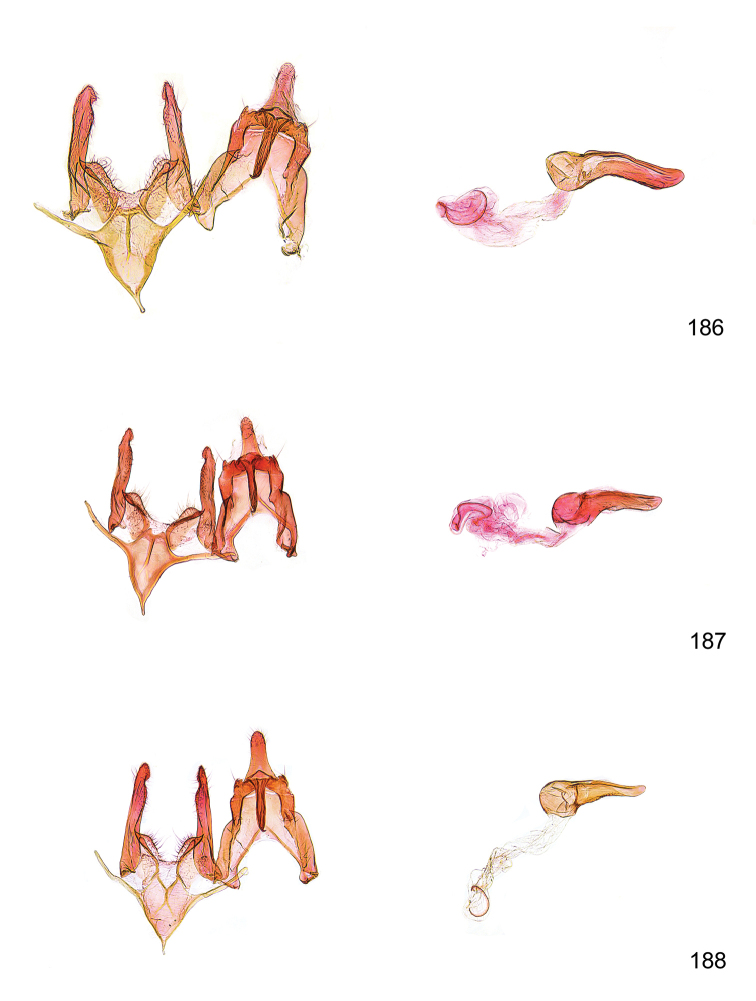
*Megacraspedus* male genitalia. **186***M.pusillus* Walsingham, 1903 – Paratype, Spain, gen. slide 33661 (BMNH) **187***M.pusillus* Walsingham, 1903 – Spain, GU 16/1432 P.H. (ZMUC) **188***M.skoui* sp. n. – Paratype, Spain, GU 16/1405 P.H. (RCZT).

**Figures 189–191.**
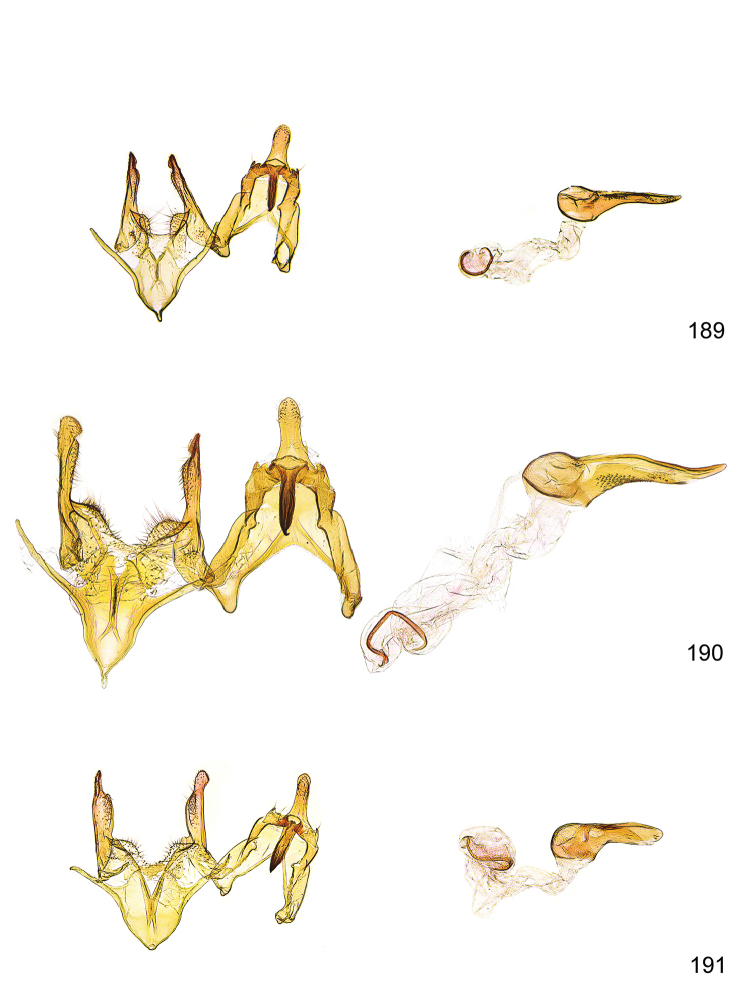
*Megacraspedus* male genitalia. **189***M.spinophallus* sp. n. – Holotype, Spain, GEL 1208 P.H. (TLMF) **190***M.spinophallus* sp. n. – Paratype, Spain, gen. slide 33659 (BMNH) **191***M.occidentellus* sp. n. – Holotype, Portugal, GU 13/1352 P.H. (ZMUC).

**Figures 192–194.**
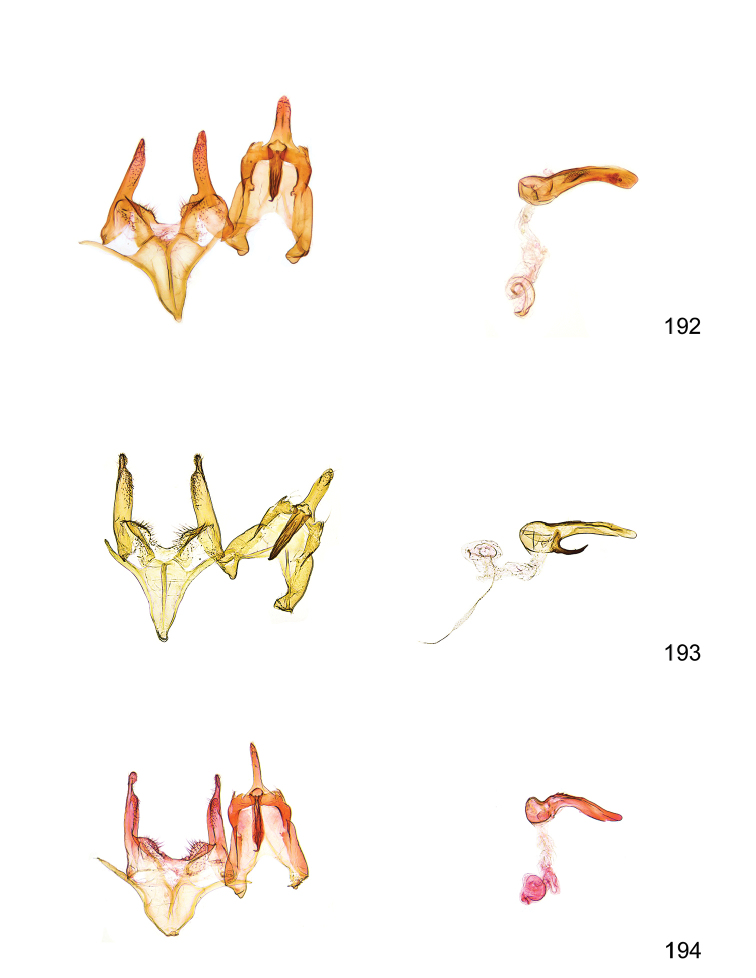
*Megacraspedus* male genitalia. **192***M.granadensis* sp. n. – Holotype, Spain, GU 16/1458 P.H. (ZMUC) **193***M.heckfordi* sp. n. – Paratype, Spain, GEL 1205 P.H. (TLMF) **194***M.tenuiuncus* sp. n. – Paratype, Spain, GU 15/1402 P.H. (TLMF).

**Figures 195–197.**
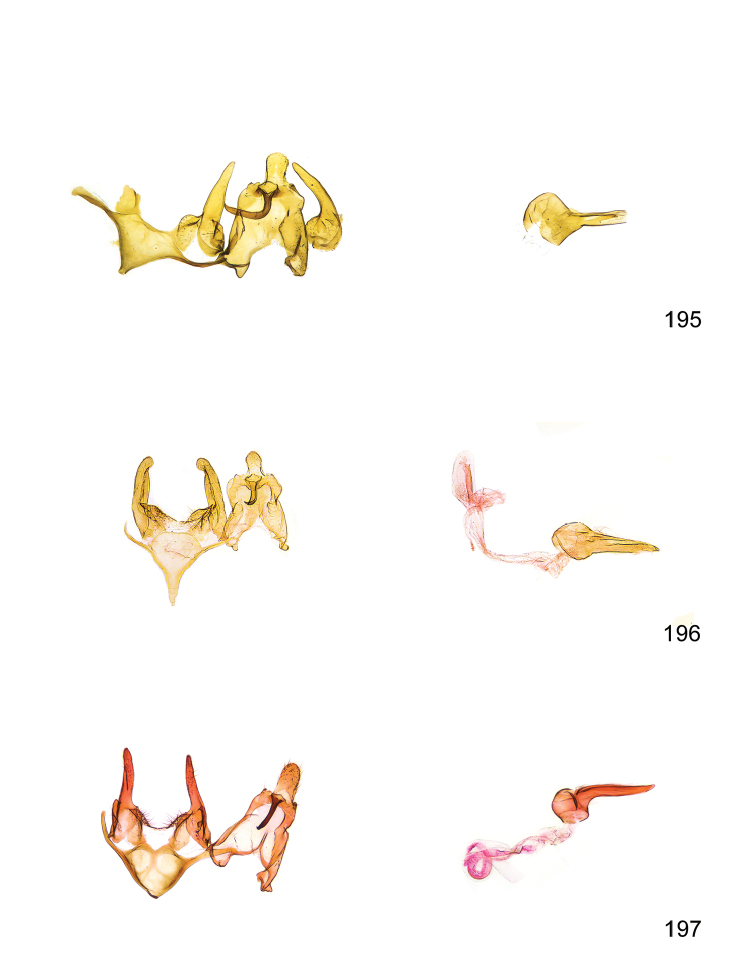
*Megacraspedus* male genitalia. **195***M.lativalvellus* Amsel, 1954 – Holotype, Malta, GU 5092 O.K. (RCCDL) **196***M.dejectella* (Staudinger, 1859) – Lectotype, Spain, GU 01/968 P.H. (ZMHU) **197***M.devorator* sp. n. – Holotype, Romania, GU 16/1461 P.H. (RCJJ).

**Figures 198–200.**
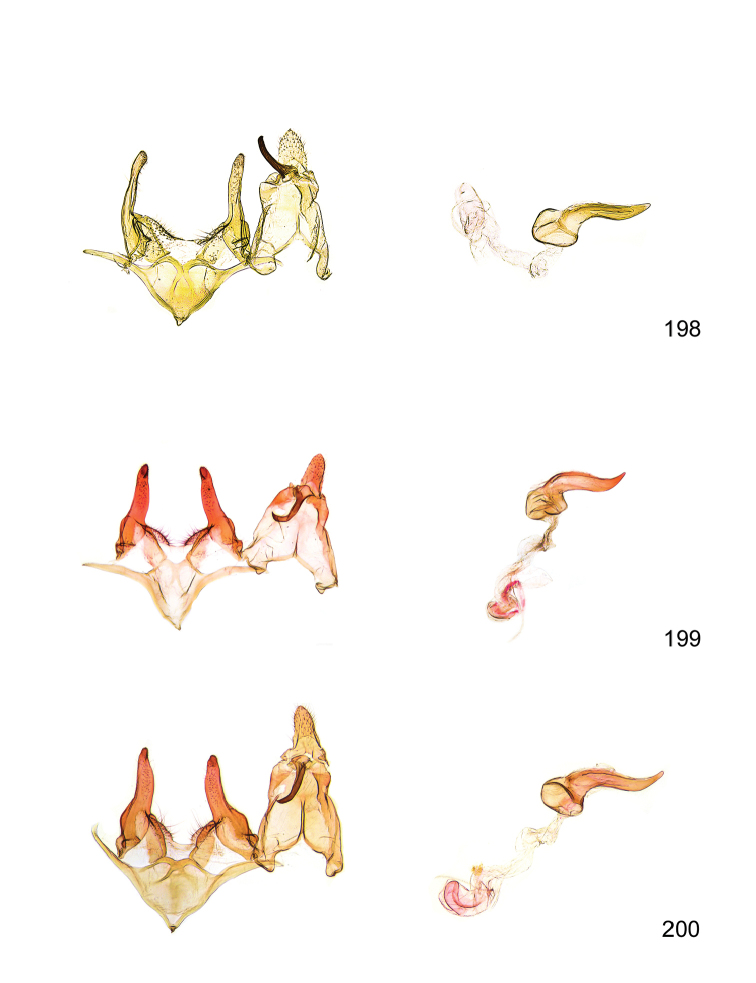
*Megacraspedus* male genitalia. **198***M.binotella* (Duponchel, 1843) – Austria, GEL 1187 P.H. (TLMF) **199***M.brachypteris* sp. n. – Paratype, Greece, GU 16/1460 P.H. (ZMUC) **200***M.brachypteris* sp. n. – Paratype, Albania, gen. slide 16.660 (NHMW).

**Figures 201–203.**
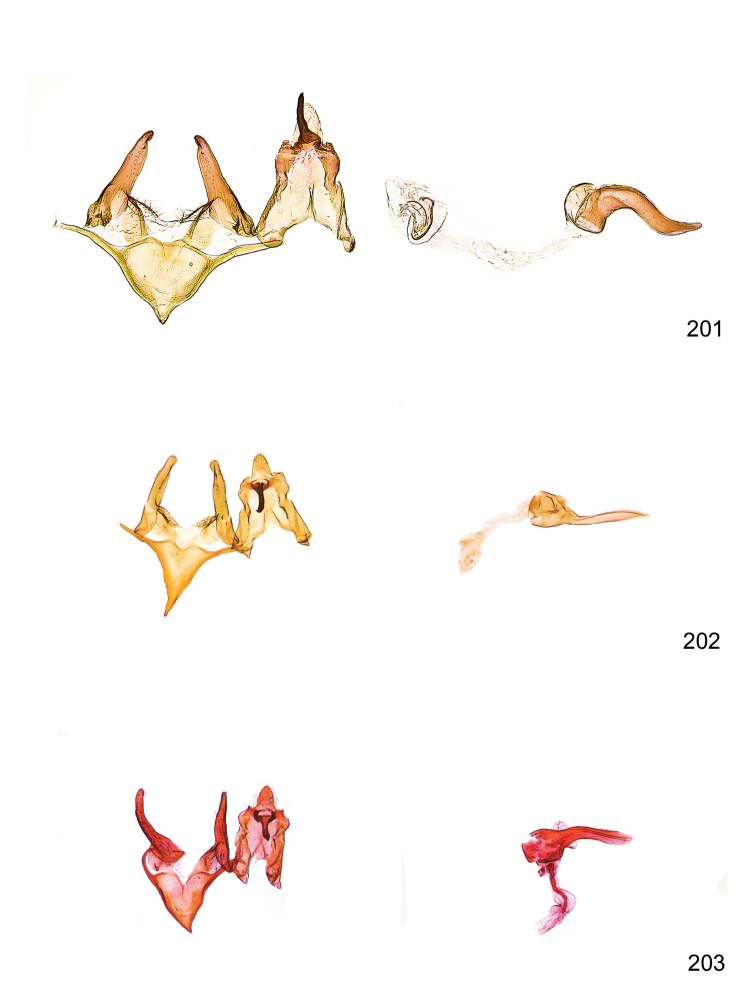
*Megacraspedus* male genitalia. **201***M.barcodiellus* sp. n. – Holotype, Macedonia, GEL 1184 P.H. (TLMF) **202***M.bilineatella* Huemer & Karsholt, 1996 – Paratype, Italy, GEL 561 P.H. (TLMF) **203***M.andreneli* Varenne & Nel, 2014 – Paratype, France, gen. slide 24592 J. Nel (TLMF).

**Figures 204–206.**
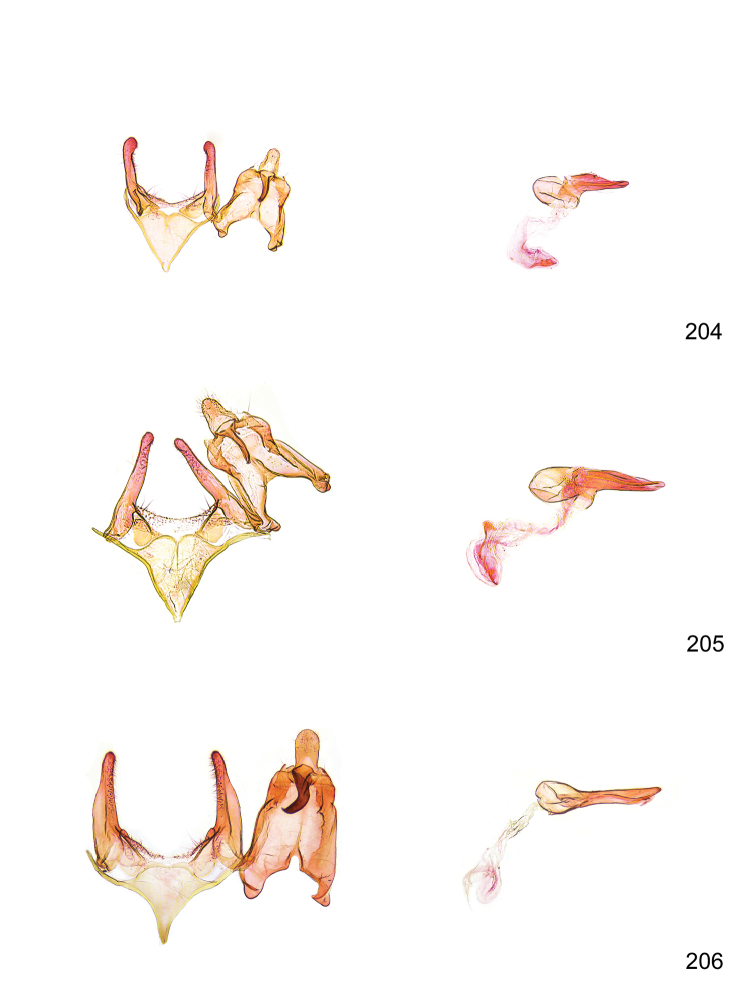
*Megacraspedus* male genitalia. **204***M.sumpichi* sp. n. – Paratype, Spain, GU 15/1397 P.H. (ZMUC) **205***M.sumpichi* sp. n. – Paratype, Spain, GU 15/1382 P.H. (ZMUC) **206***M.tabelli* sp. n. – Holotype, Morocco, GU 16/1452 P.H. (ZMUC).

**Figures 207–209.**
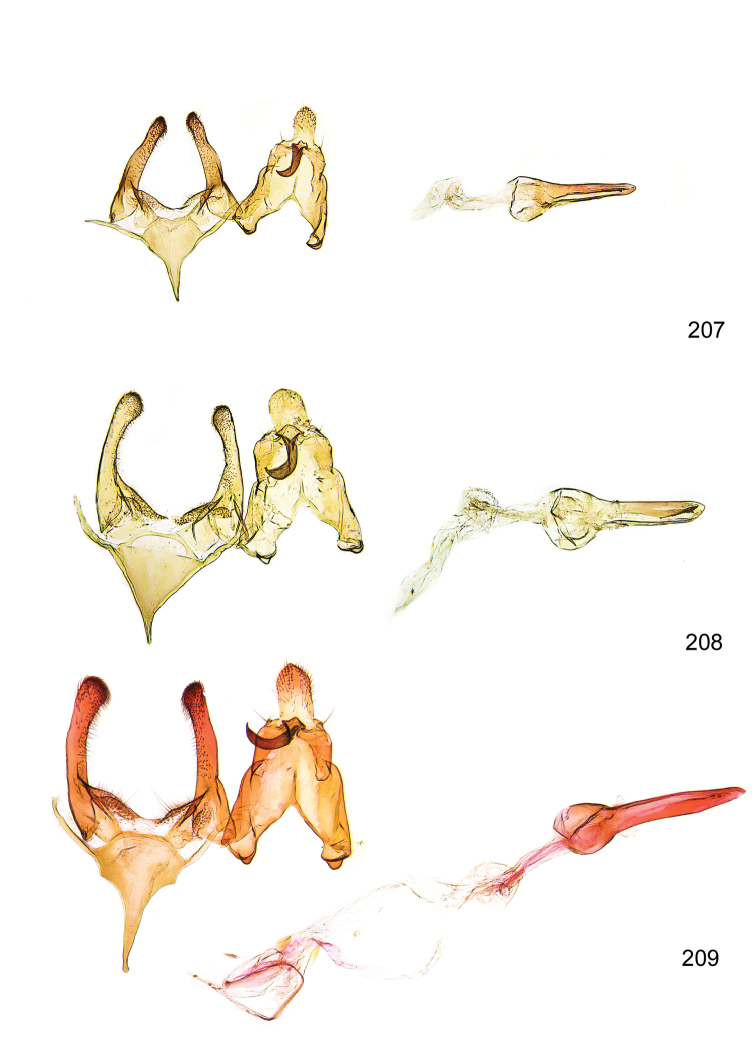
*Megacraspedus* male genitalia. **207***M.gallicus* sp. n. – Paratype, France, GEL 1203 P.H. (TLMF) **208***M.ribbeella* (Caradja, 1920) – Spain, gen. slide 16.650 (NHMW) **209***M.libycus* sp. n. – Holotype, Libya, GU 16/1435 P.H. (ZMUC).

**Figures 210–212.**
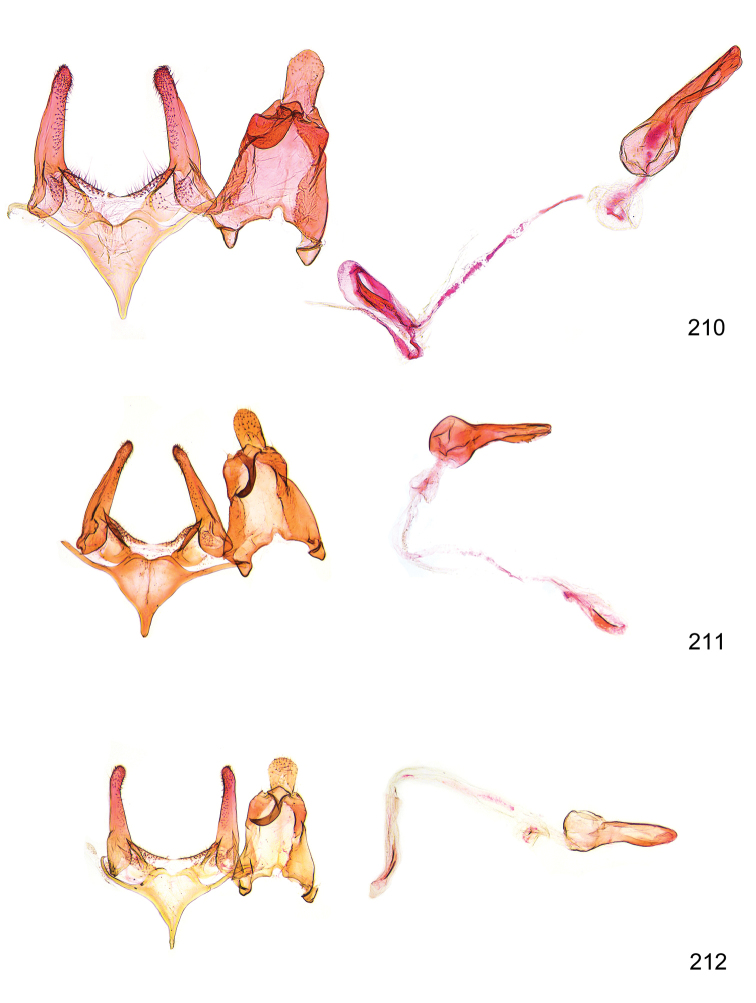
*Megacraspedus* male genitalia. **210***M.numidellus* (Chrétien, 1915) – Syntype (*mareotidellus*), Libya, gen. slide 33664 (BMNH) **211***M.numidellus* (Chrétien, 1915) – Morocco, GU 16/1417 P.H. (ZMUC) **212***M.numidellus* (Chrétien, 1915) – Spain (Fuerteventura), GU 16/1424 P.H. (ZMUC).

**Figures 213–215.**
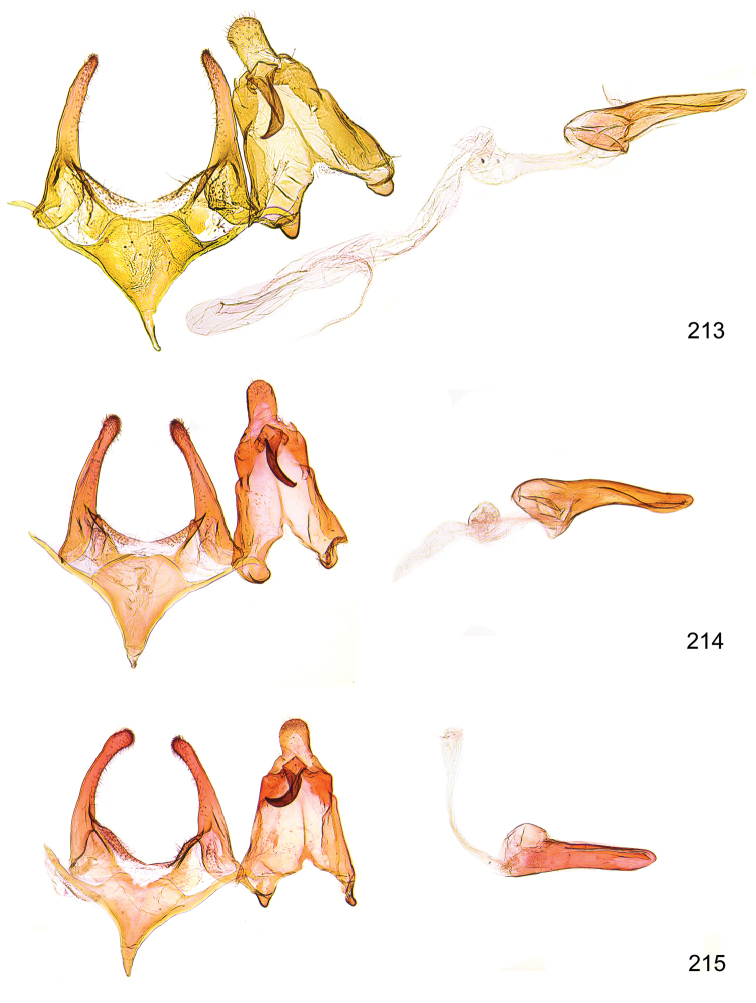
*Megacraspedus* male genitalia. **213***M.albovenata* Junnilainen, 2010 – Czech Republic, GEL 1210 P.H. (TLMF) **214***M.longipalpella* Junnilainen, 2010 – Paratype, Russia, GU 15/1398 P.H. (ZMUC) **215***M.niphorrhoa* (Meyrick, 1926) – Russia, GU 15/1399 P.H. (ZMUC).

**Figures 216–218.**
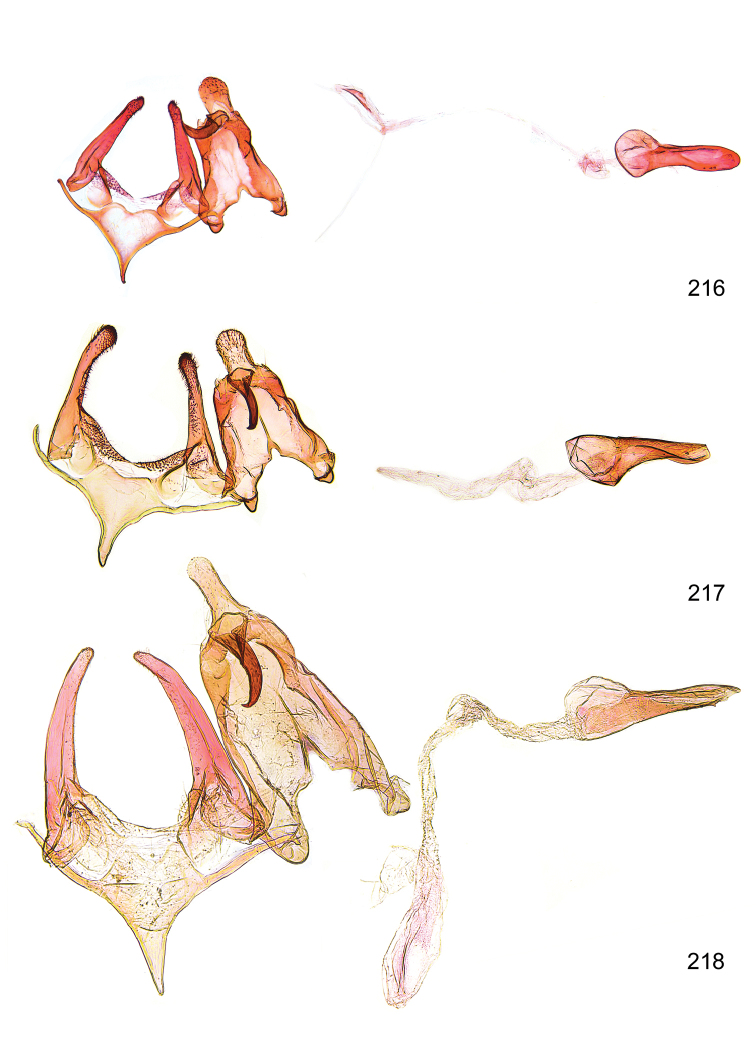
*Megacraspedus* male genitalia. **216***M.albella* – Iran, gen. slide 16.663 (NHMW) **217***M.fallax* (Mann, 1867) – Holotype, Hungary, gen. slide 16.647 (NHMW) **218***M.balneariellus* (Chrétien, 1907) – France, GU 14/1388 P.H. (ZMUC).

**Figures 219–221.**
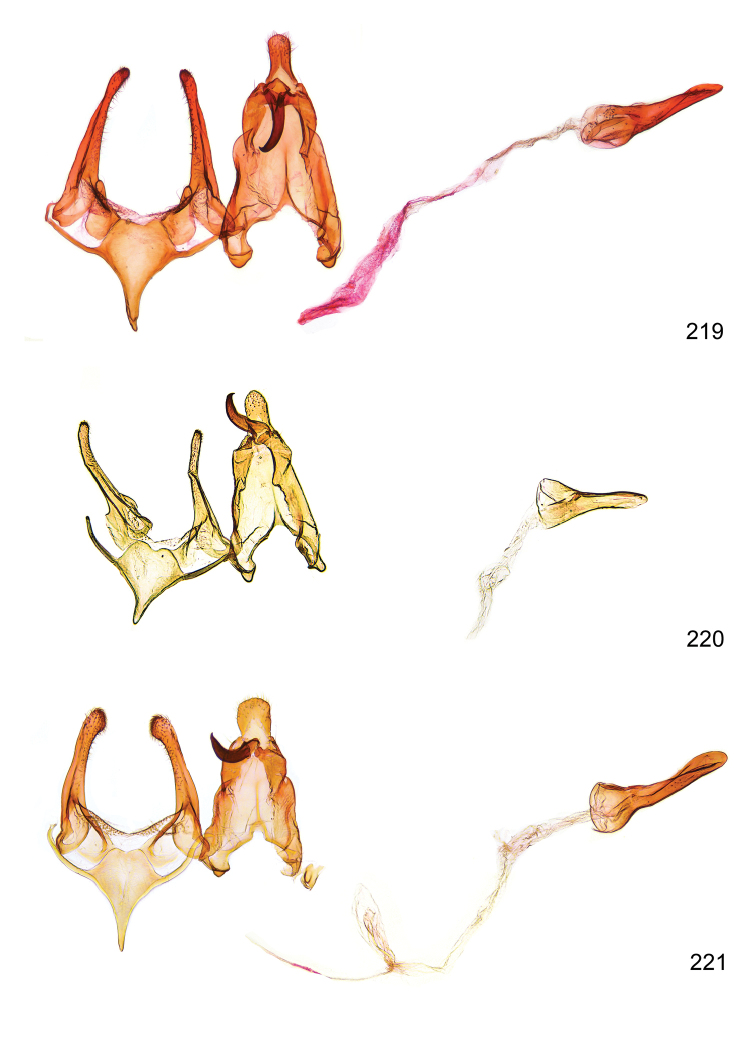
*Megacraspedus* male genitalia. **219***M.balneariellus* (Chrétien, 1907) – Croatia, GU 16/1457 P.H. (RCJJ) **220***M.podolicus* (Toll, 1942) – Austria, GEL 1202 P.H. (TLMF) **221***M.kazakhstanicus* sp. n. – Holotype, Kazakhstan, GU 17/1496 P.H. (RCKN).

**Figures 222–224.**
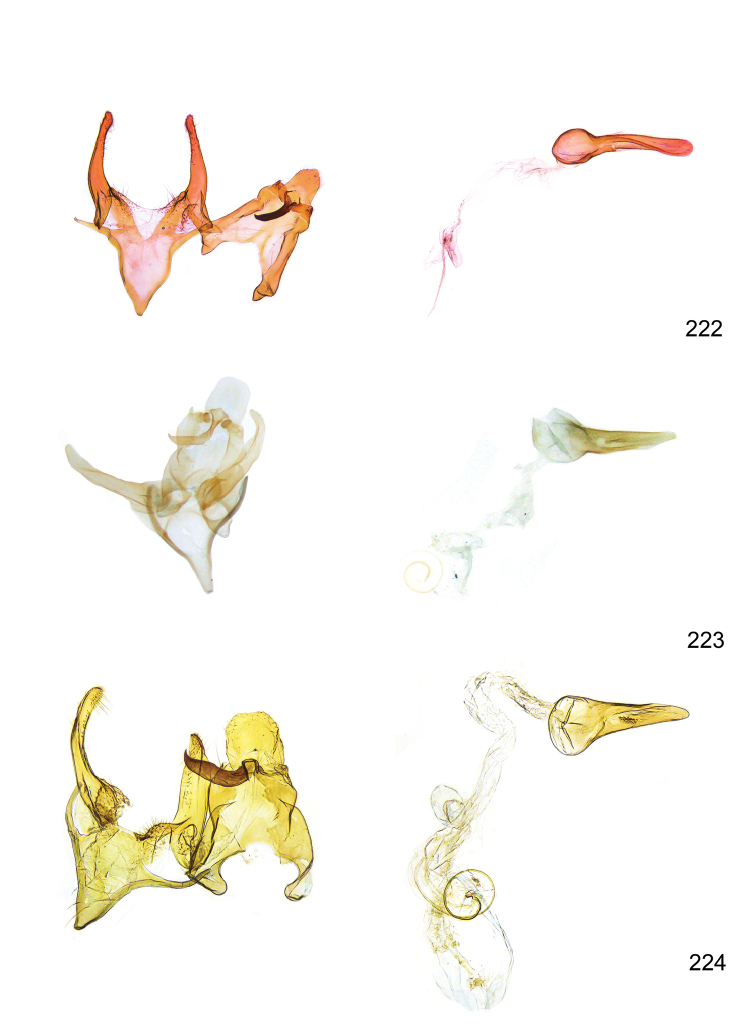
*Megacraspedus* male genitalia. **222***M.knudlarseni* sp. n. – Holotype, Spain (Gran Canaria), GU 16/1454 P.H. (ZMUC) **223***M.majorella* Caradj, 1920 – Lectotype, Kyrgyzstan, Alai mts (MGAB) **224***M.latiuncus* sp. n. – Holotype, Kazakhstan, genitalia slide 5324 O.K. (ZMUC).

**Figures 225–227.**
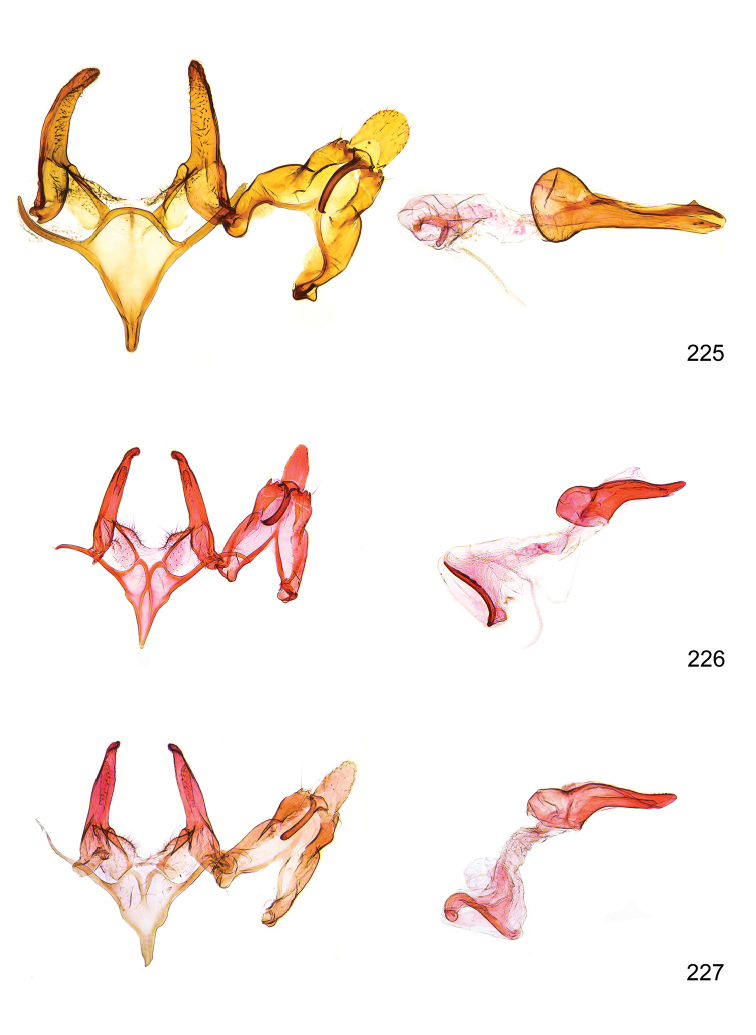
*Megacraspedus* male genitalia. **225***M.tenuignathos* sp. n. – Holotype, Morocco, GU 16/1423 P.H. (RCAW) **226***M.glaberipalpus* sp. n. – Paratype, Morocco, GEL 1245 P.H. (TLMF) **227***M.glaberipalpus* sp. n. – Paratype, Morocco, gen. slide 16.665 (NHMW).

**Figures 228–230.**
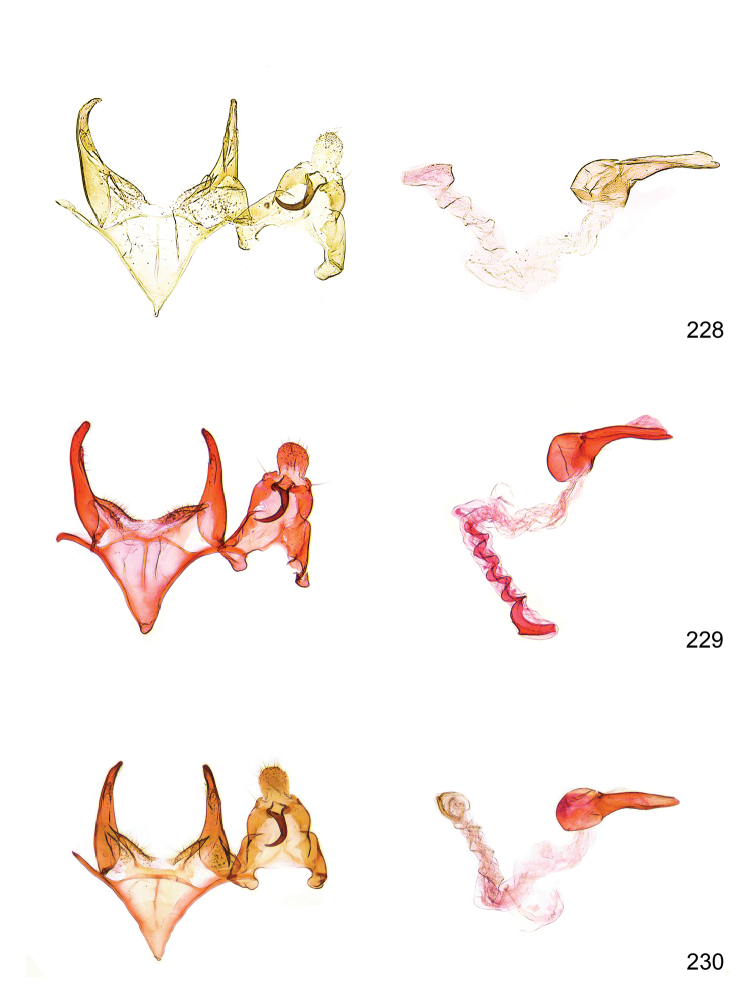
*Megacraspedus* male genitalia. **228***M.imparellus* (Fischer von Röslerstamm, 1843) – Austria, GEL 1195 P.H. (TLMF) **229***M.imparellus* (Fischer von Röslerstamm, 1843) – Paratype (*litovalvellus*), Russia, GU 15/1400 P.H. (ZMUC) **230***M.imparellus* (Fischer von Röslerstamm, 1843) – Greece, GU 16/1456 P.H. (RCJJ).

**Figures 231–233.**
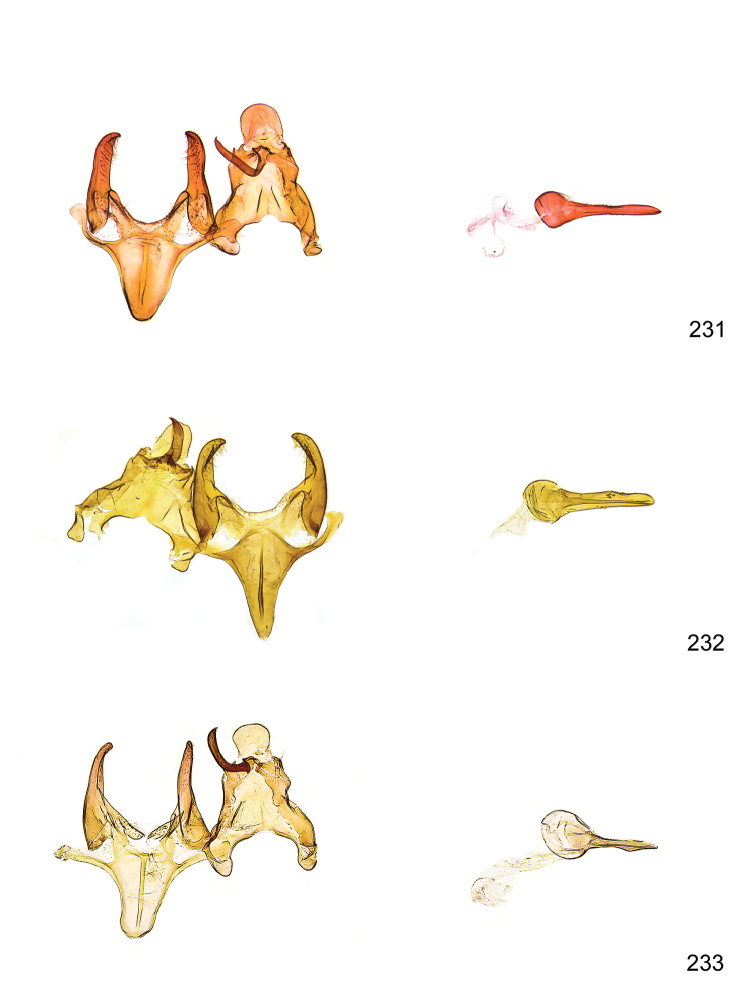
*Megacraspedus* male genitalia. **231***M.multispinella* Junnilainen & Nupponen, 2010 – Paratype, Russia, GU 16/1418 P.H. (MZH) **232***M.nupponeni* sp. n. – Holotype, Russia, genitalia slide 8/8.x.2006 K. Nupponen. (RCKN) **233***M.cerussatellus* Rebel, 1930 – Lectotype, Bulgaria, gen. slide 16.648 (NHMW).

**Figures 234–236.**
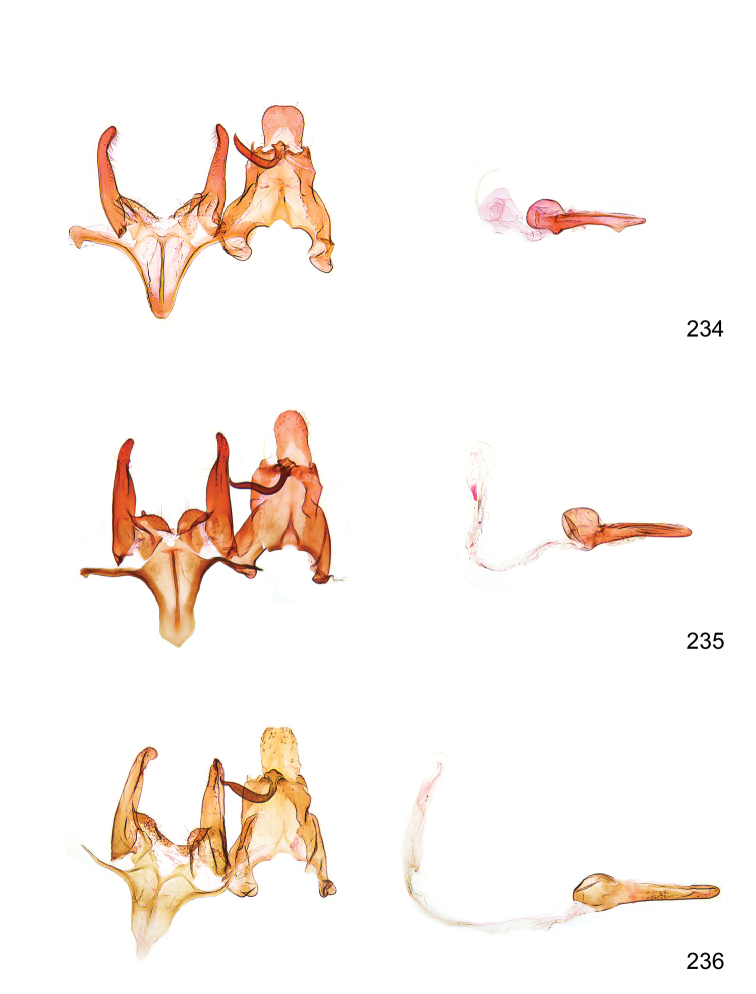
*Megacraspedus* male genitalia. **234***M.attritellus* Staudinger, 1871 – Lectotype, Russia, GU 16/1426 P.H. (ZMHU) **235***M.consortiella* Caradja, 1920 – Kyrgyzstan, GU 17/1498 P.H. (RCKN) **236***M.pototskii* sp. n. – Holotype, Kyrgyzstan, GU 17/1492 P.H. (RCKN).

**Figures 237–239.**
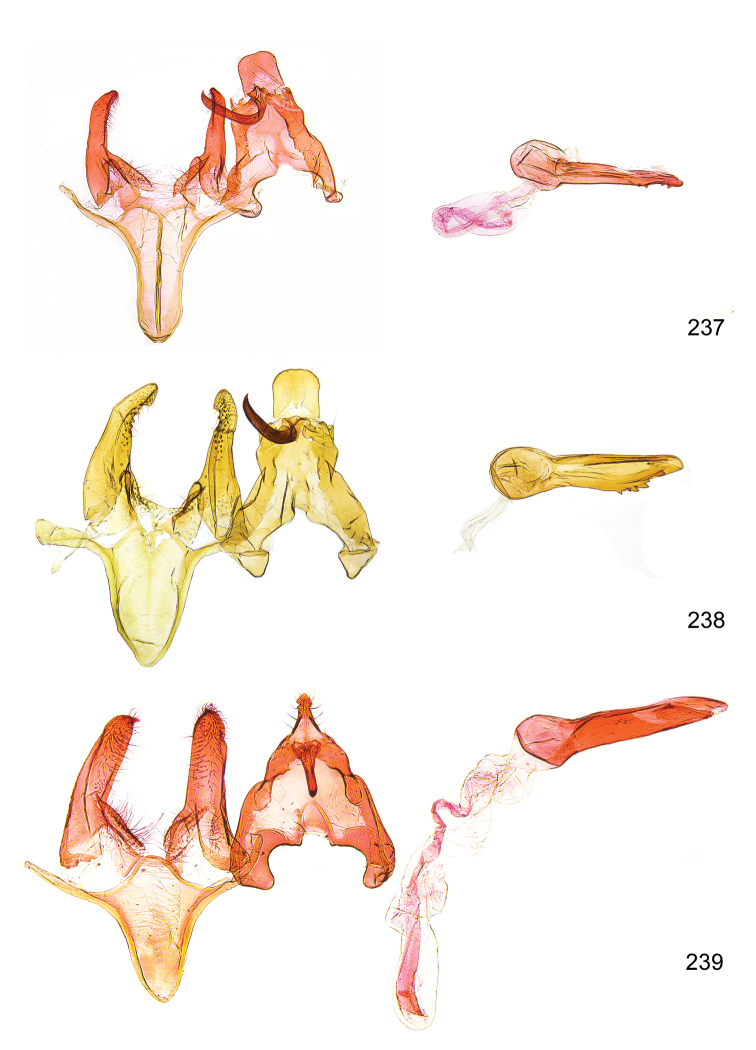
*Megacraspedus* male genitalia. **237***M.leuca* (Filipjev, 1929) – Russia, GEL 1240 P.H. (TLMF) **238***M.orenburgensis* Junnilainen & Nupponen, 2010 – Paratype, Russia, genitalia slide 17.iv.2006 K. Nupponen. (RCKN) **239***M.lagopellus* Herrich-Schäffer, 1860 – Hungary, gen. slide 16.656 (NHMW).

**Figures 240–242.**
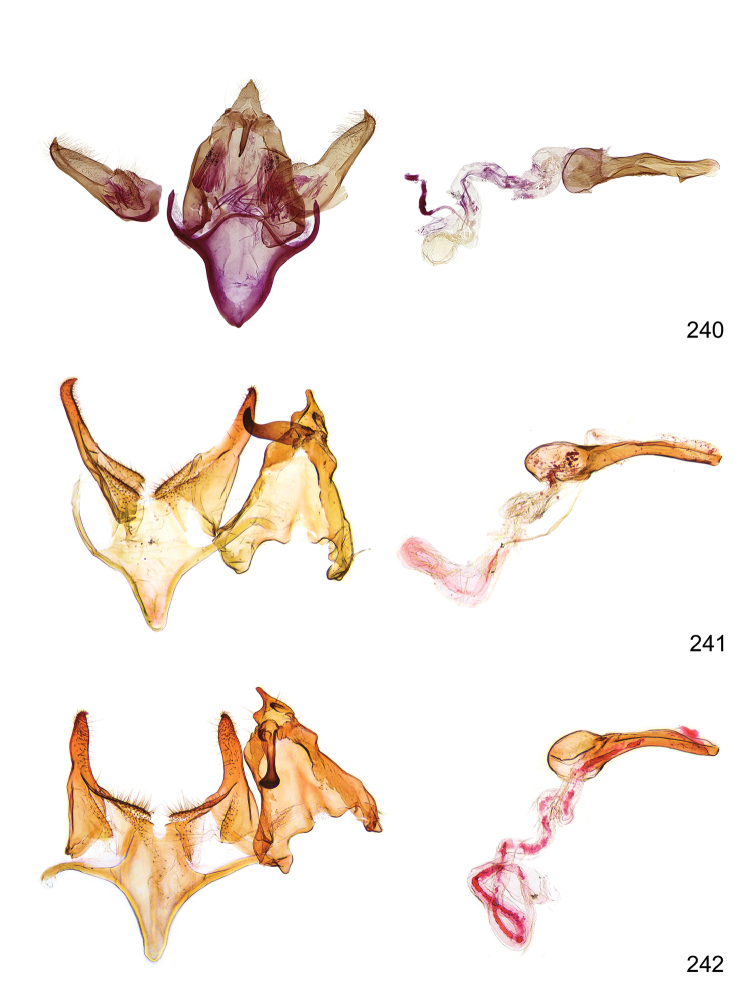
*Megacraspedus* male genitalia. **240***M.coleophorodes* (Li & Zheng, 1995) – Paratype, gen. slide L94178 (NKU) **241***M.kirgizicus* sp. n. – Holotype, Kyrgyzstan, GU 16/1406 P.H. (LMK) **242***M.kirgizicus* sp. n. – Paratype, Kyrgyzstan, GU 17/1491 P.H. (RCKN).

**Figures 243–245.**
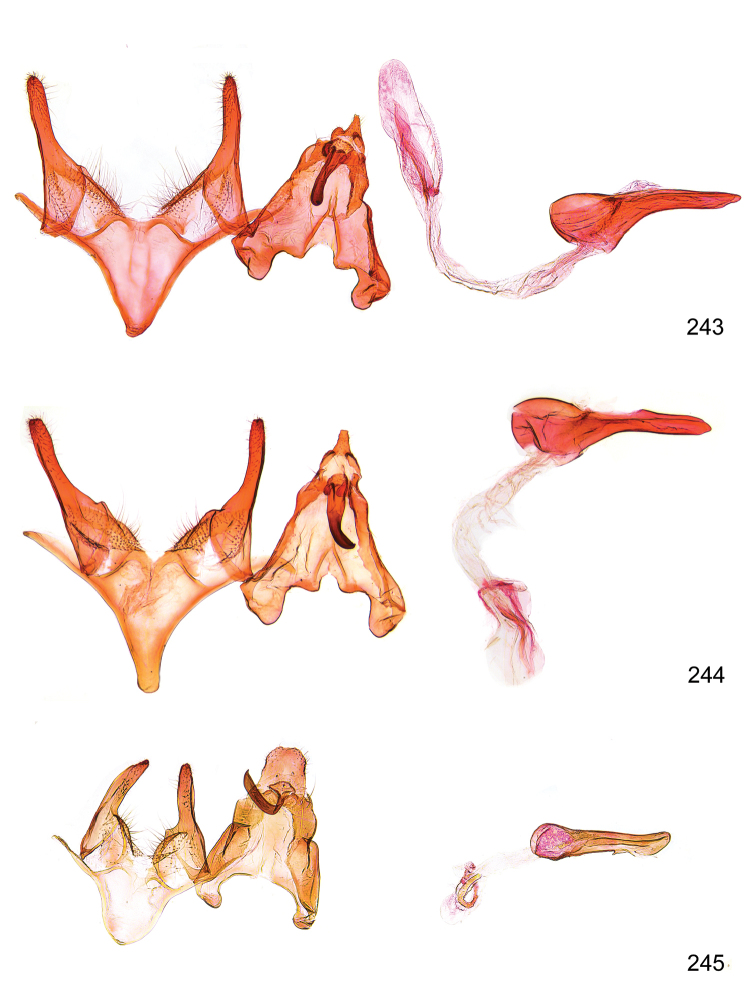
*Megacraspedus* male genitalia. **243***M.argyroneurellus* Staudinger, 1871 – Ukraine, GU 16/1427 P.H. (RCAB) **244***M.argyroneurellus* Staudinger, 1871 – Iran, gen. slide 16.658 (NHMW) **245***M.ibericus* sp. n. – Paratype, Spain, GU 16/1431 P.H. (TLMF).

**Figures 246–248.**
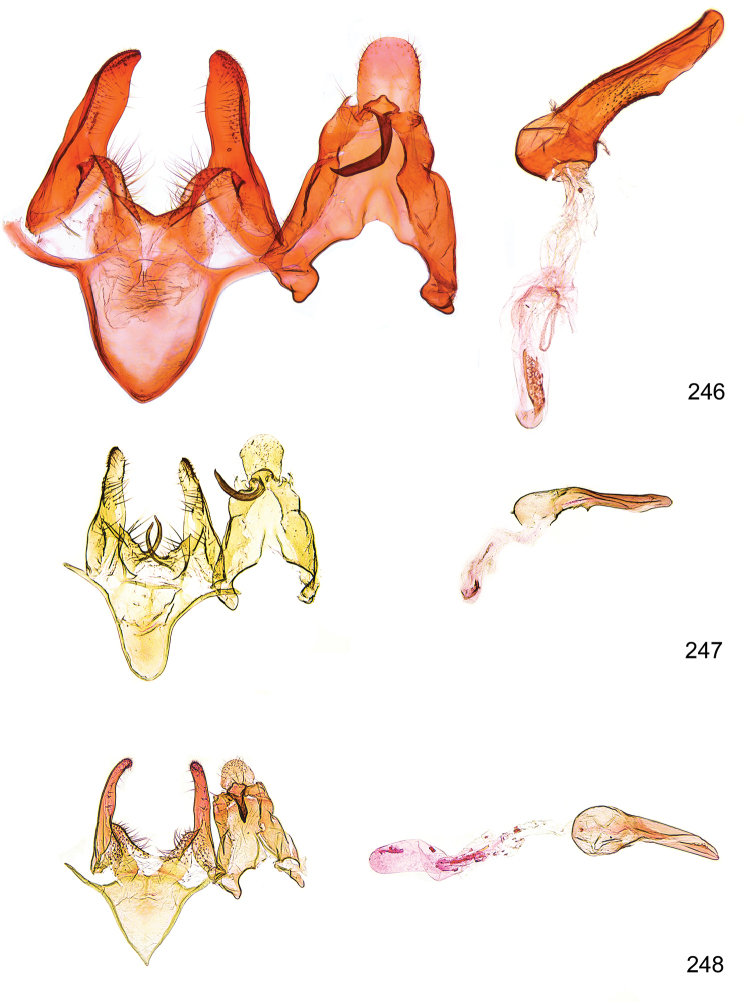
*Megacraspedus* male genitalia. **246***M.violacellum* (Chrétien, 1915) – Tunisia, GEL 1269 P.H. (TLMF) **247***M.squalida* Meyrick, 1926 – Spain, GEL 1193 P.H. (TLMF) **248***M.pentheres* Walsingham, 1920 – Paratype, France, gen. slide 33662 (BMNH).

**Figures 249–251.**
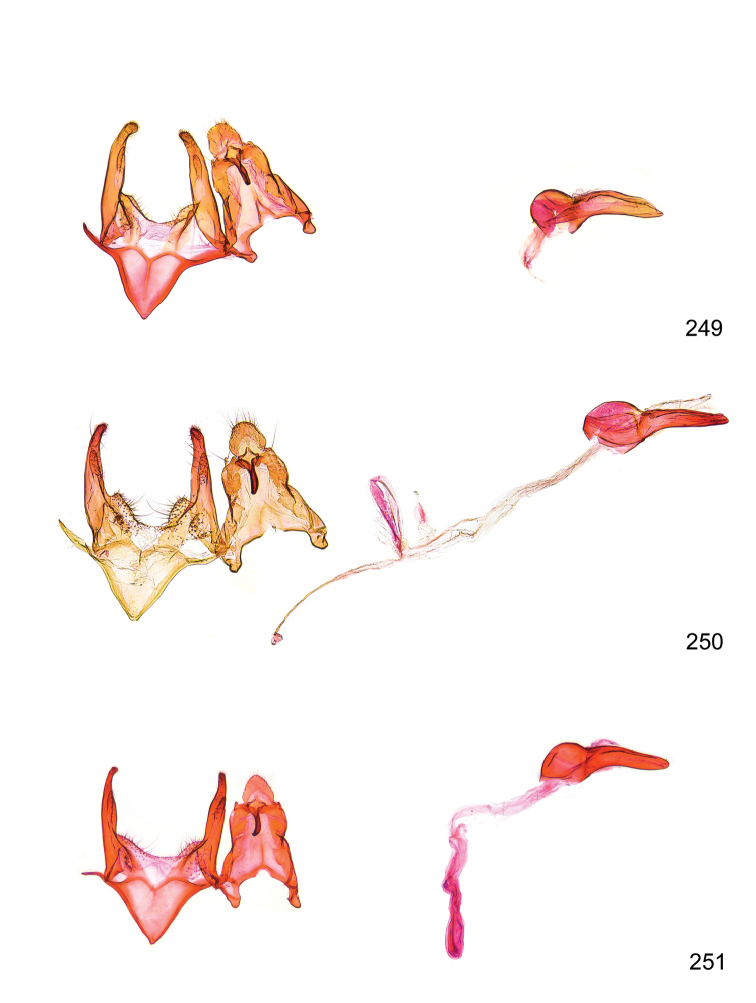
*Megacraspedus* male genitalia. **249***M.steineri* sp. n. – Holotype, Morocco, GU 16/1421 P.H. (ZMUC) **250***M.gibeauxi* sp. n. – Holotype, Tunesia, GU 16/1442 P.H. (ZMUC) **251***M.multipunctellus* sp. n. – Holotype, Turkey, GU 16/1455 P.H. (ECKU).

**Figures 252–254.**
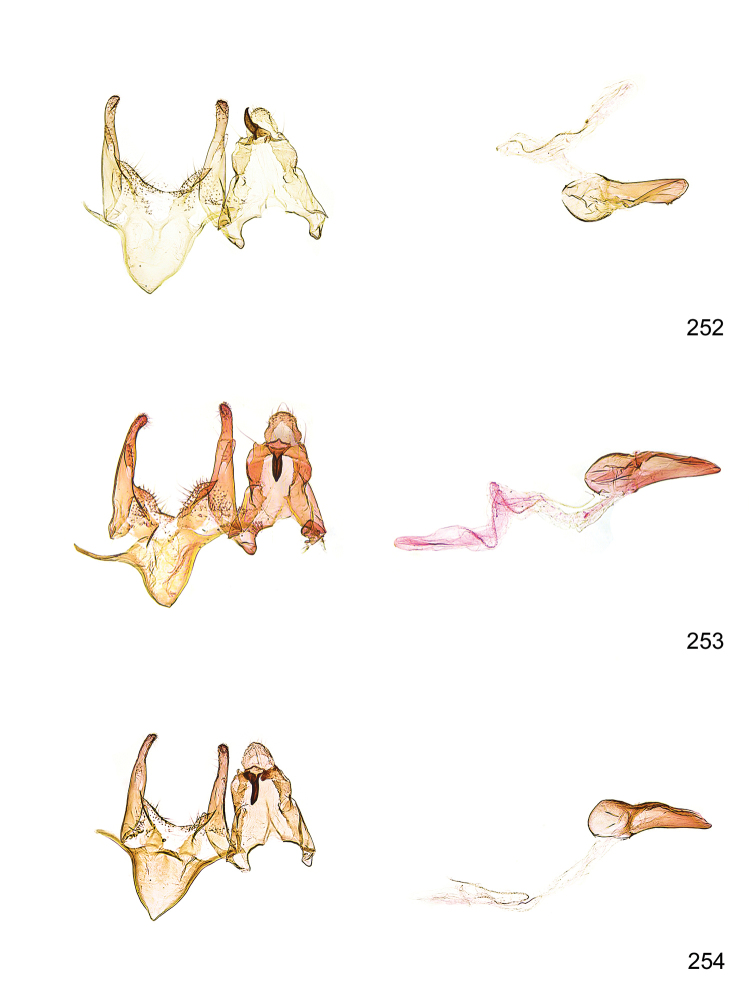
*Megacraspedus* male genitalia. **252***M.teriolensis* sp. n. – Paratype, Italy, GEL 1191 P.H. (TLMF) **253***M.teriolensis* sp. n. – Paratype, Italy, GEL 1221 P.H. (TLMF) **254***M.korabicus* sp. n. – Holotype, Macedonia, GEL 1183 P.H. (TLMF).

**Figures 255–257.**
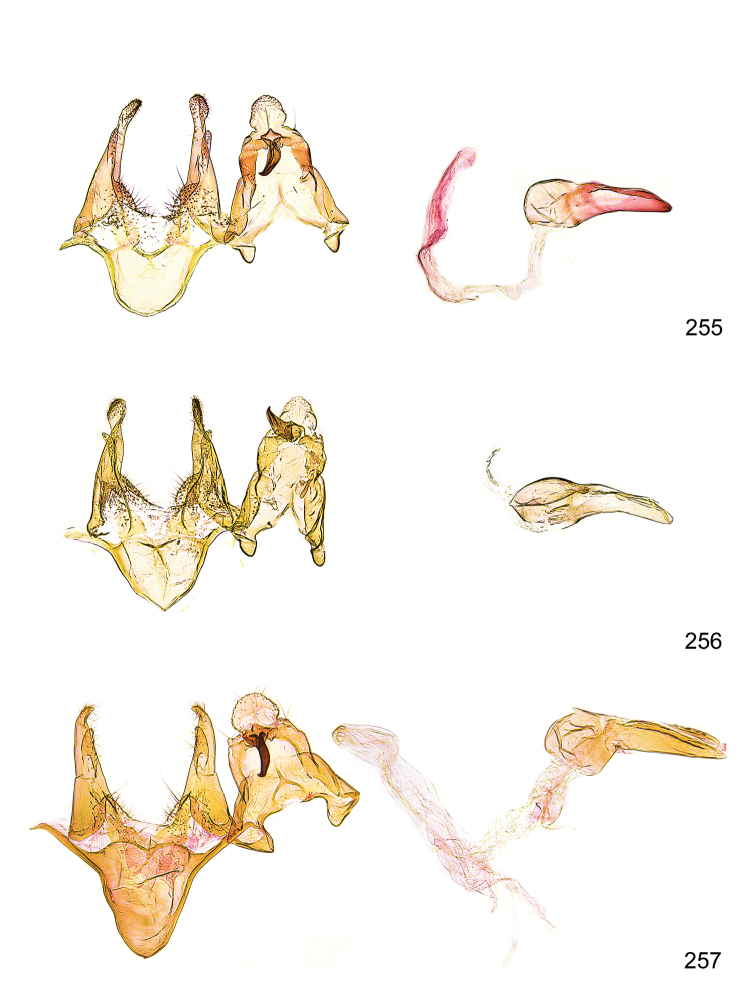
*Megacraspedus* male genitalia. **255***M.quadristictus* Lhomme, 1946 – France, GEL 1204 P.H. (TLMF) **256***M.quadristictus* Lhomme, 1946 – France, GEL 1207 P.H. (TLMF) **257***M.eburnellus* Huemer & Karsholt, 2001 – Paratype, Italy, GEL 1007 P.H. (TLMF).

**Figures 258–260.**
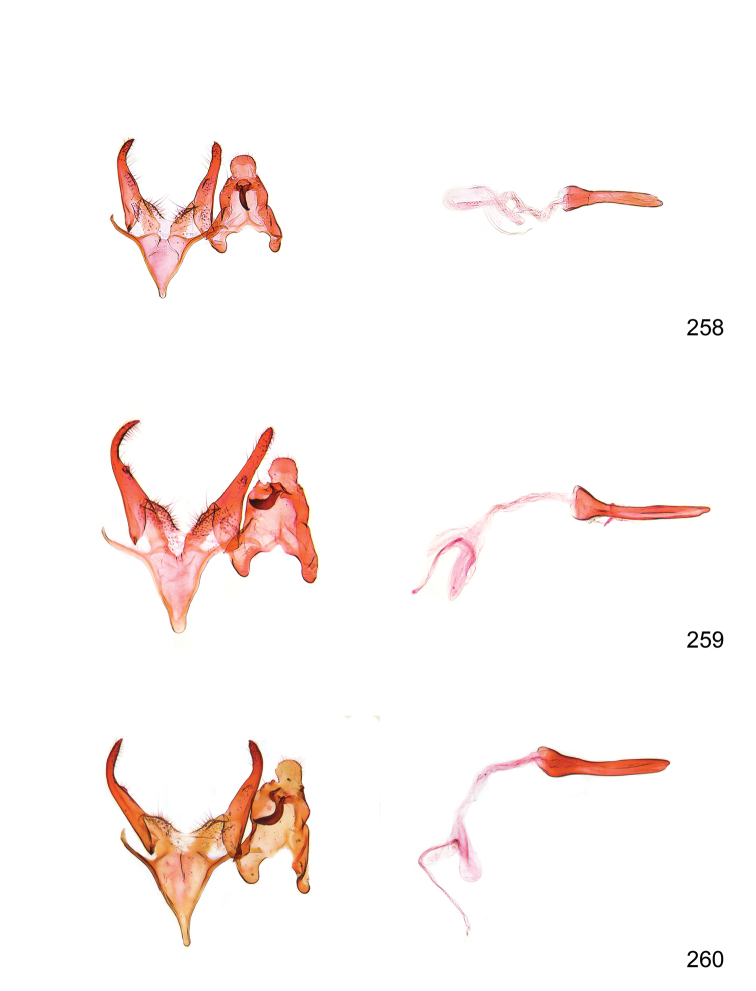
*Megacraspedus* male genitalia. **258***M.skulei* sp. n. – Paratype, Spain, GU 16/1410 P.H. (NMPC) **259***M.longivalvellus* sp. n. – Holotype, Morocco, GU 16/1422 P.H. (ZMUC) **260***M.longivalvellus* sp. n. – Paratype, Morocco, GEL 1249 P.H. (TLMF).

**Figures 261–263.**
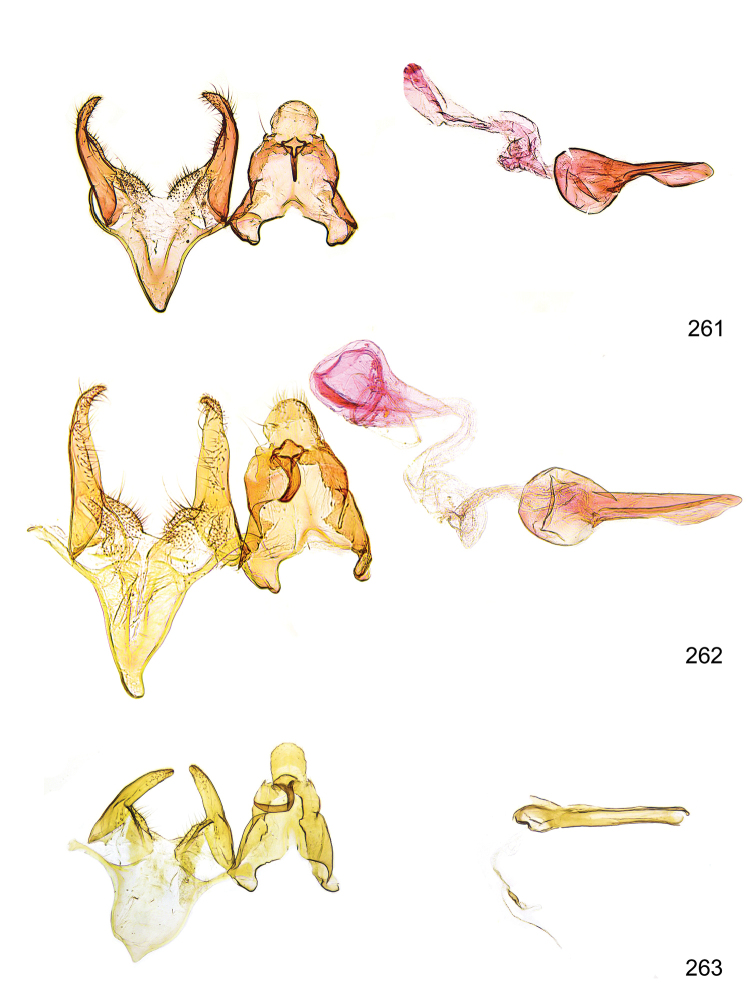
*Megacraspedus* male genitalia. **261***M.peyerimhoffi* Le Cerf, 1925 – Spain, GU 13/1351 P.H. (ZMUC) **262***M.peyerimhoffi* Le Cerf, 1925 – Algeria, gen. slide 16.654 (NHMW) **263***M.peslieri* sp. n. – Holotype, France, Holotype, GEL 1274 P.H. (TLMF).

**Figures 264–266.**
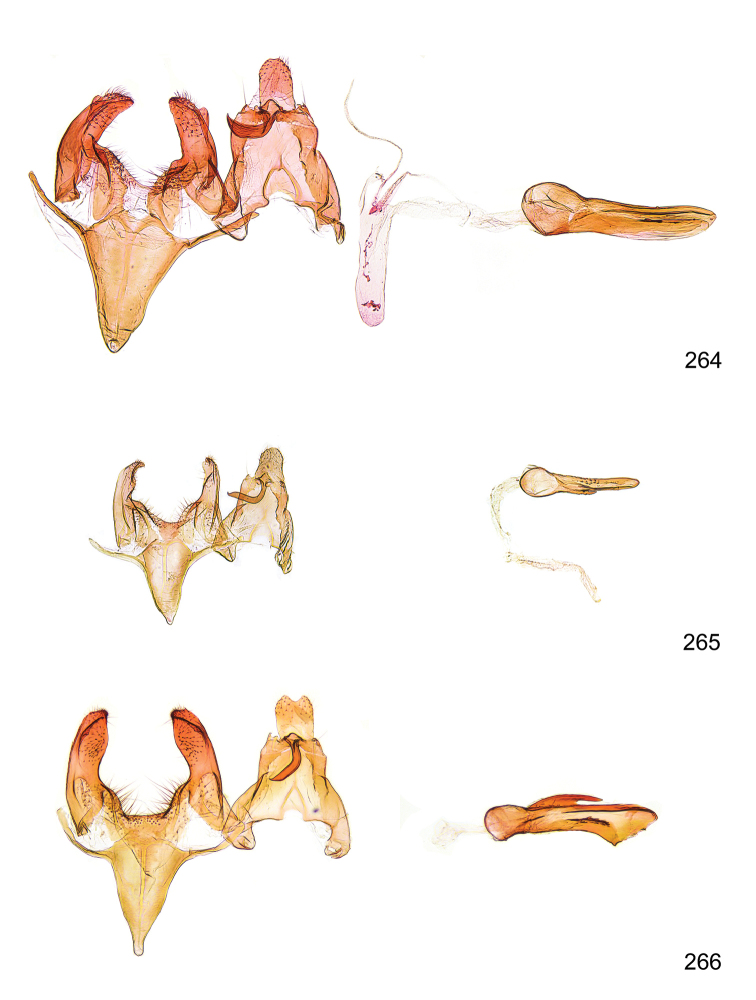
*Megacraspedus* male genitalia. **264***M.grisea* (Filipjev, 1931) – Syntype, China, gen. slide 33663 (BMNH) **265***M.pacificus* sp. n. – Holotype, Afghanistan, gen. slide 16.661 (NHMW) **266***M.armatophallus* sp. n. – Holotype, Afghanistan, GU 17/1497 P.H. (SMNK).

### Figures of *Megacraspedus* female genitalia

**Figures 267–269.**
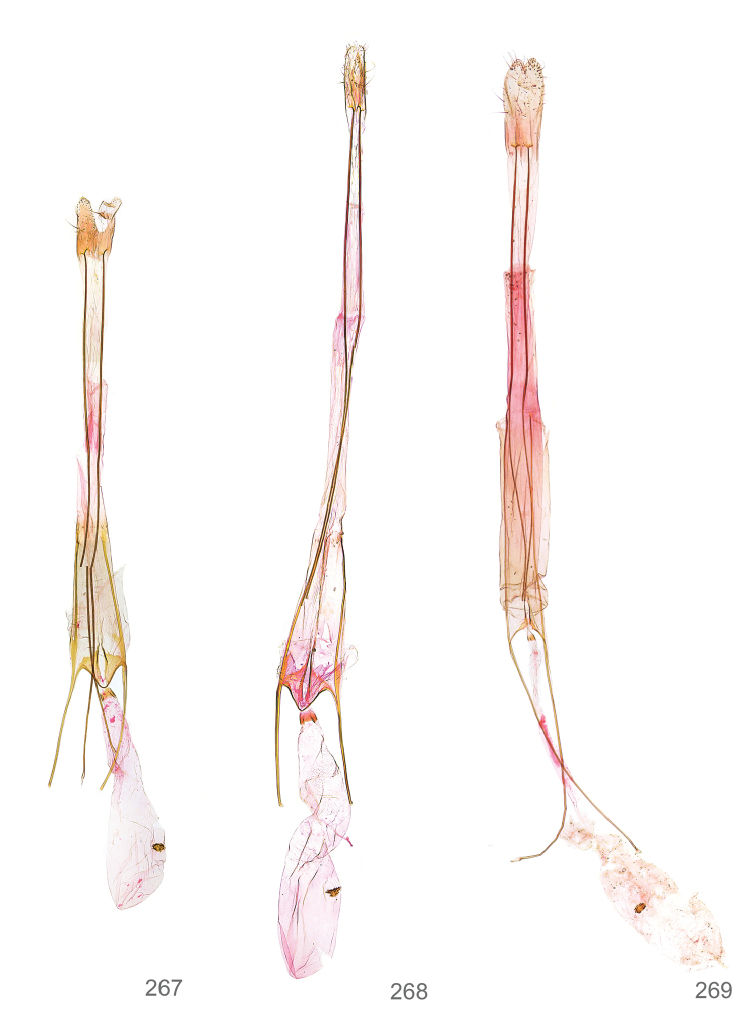
*Megacraspedus* female genitalia. **267***M.lanceolellus* (Zeller, 1850) – France, GU 16/1472 (RCJJ) **268***M.lanceolellus* (Zeller, 1850) – Spain, gen. slide 33658 (BMNH) **269***M.monolorellus* Rebel, 1905 – Turkey, 30 km NE Konya, 27.v.1969, leg. E. Arenberger, GU 18/1507 P.H. (RCEA).

**Figures 270–272.**
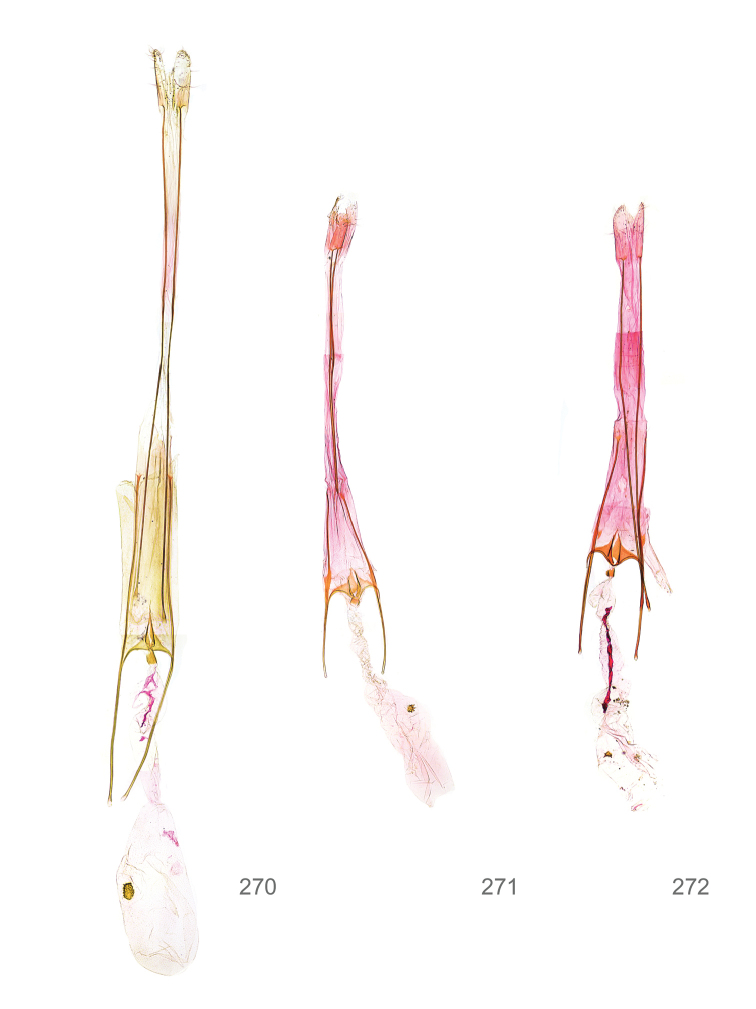
*Megacraspedus* female genitalia. **270***M.similellus* sp. n. – Paratype, Bulgaria, GU 16/1471 P.H. (RCJJ) **271***M.dolosellus* (Zeller, 1839) – Austria, GU 17/1484 P.H. (NHMW) **272***M.dolosellus* (Zeller, 1839) – Greece, GU 16/1467 P.H. (RCJJ).

**Figures 273–275.**
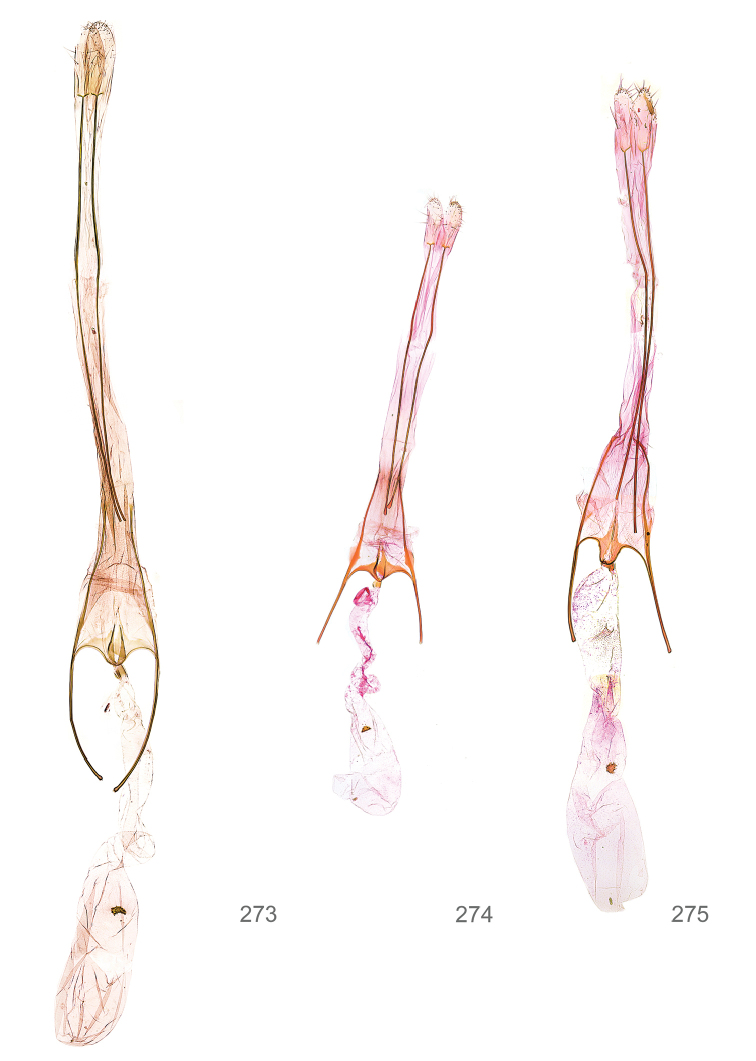
*Megacraspedus* female genitalia. **273***M.dolosellus* (Zeller, 1839) – Bulgaria, gen. slide 16.527 (NHMW) **274***M.dolosellus* (Zeller, 1839) – Russia, GU 17/1483 P.H. (ZMUC) **275***M.faunierensis* sp. n. – Paratype, Italy, GEL 1235 P.H. (TLMF).

**Figures 276–278.**
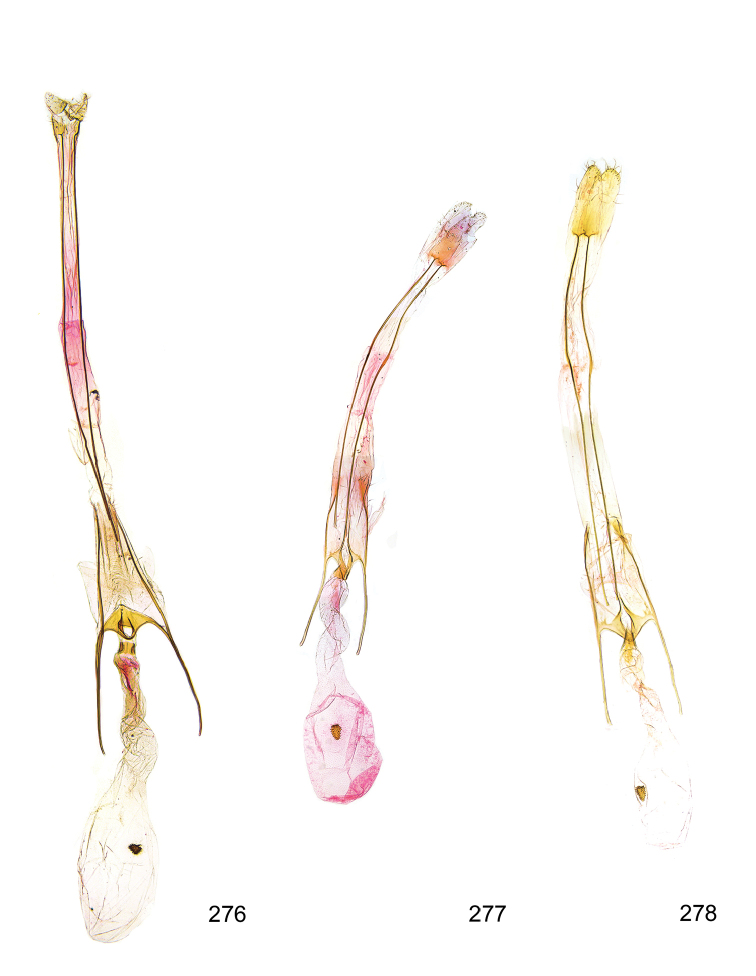
*Megacraspedus* female genitalia. **276***M.spinophallus* sp. n.– Paratype, Spain, gen. slide 33660 (BMNH) **277***M.binotella* (Duponchel, 1843) – Austria, gen. slide 16.667 (NHMW) **278***M.brachypteris* sp. n. – Paratype, Macedonia, gen. slide 16.669 (NHMW).

**Figures 279–281.**
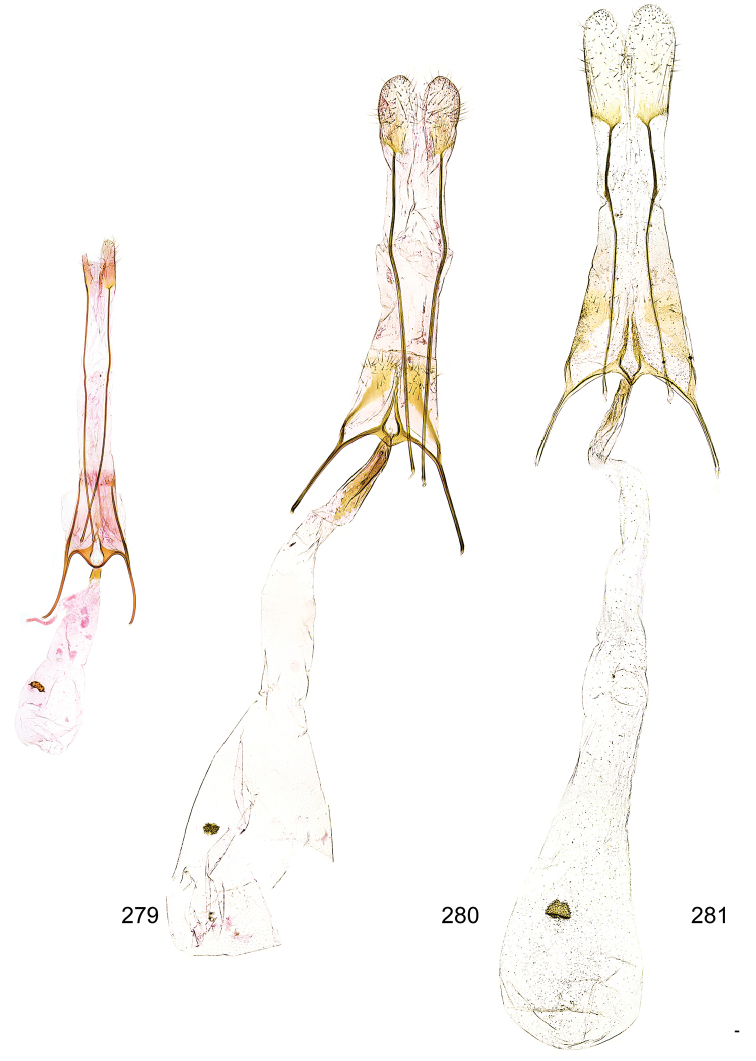
*Megacraspedus* female genitalia. **279***M.sumpichi* sp. n. – Paratype, Spain, GU 15/1397 P.H. (RCZT) **280***M.gallicus* sp. n. – Paratype, France, GEL 1230 P.H. (TLMF) **281***M.ribbeella* (Caradja, 1920) – Spain, gen. slide 16.651 (NHMW).

**Figures 282–284.**
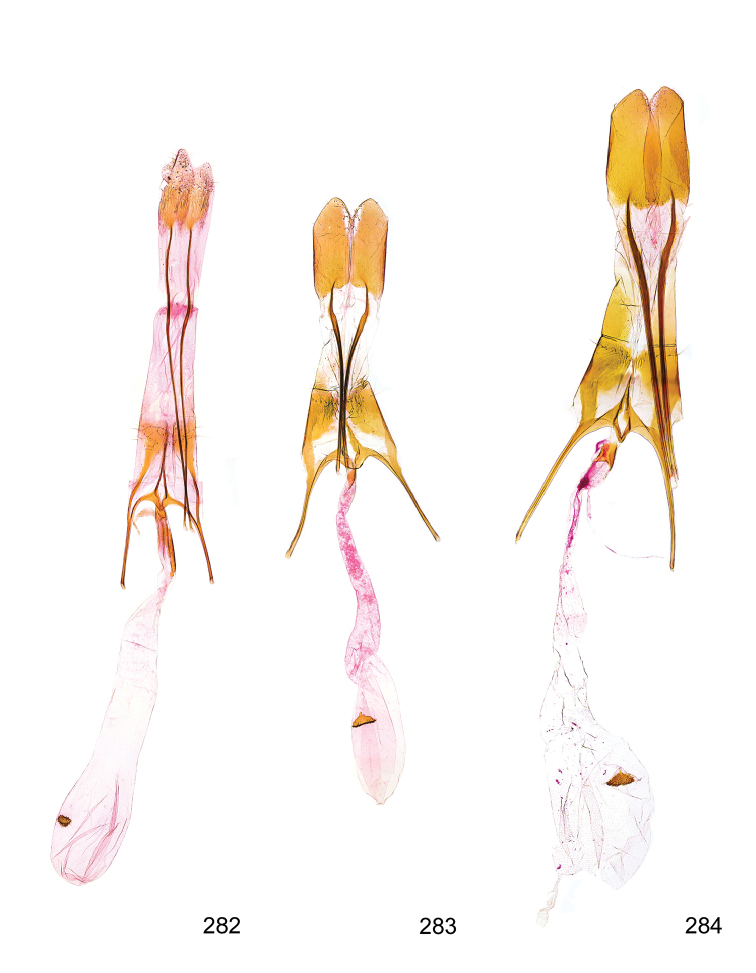
*Megacraspedus* female genitalia. **282***M.libycus* sp. n. – Paratype, Morocco, GEL 1266 P.H. (TLMF) **283***M.albovenata* Junnilainen, 2010 – Czech Republic, GU 17/1474 P.H. (NMPC) **284***M.longipalpella* Junnilainen, 2010 – Ukraine, GU 17/1475 P.H. (RCAB).

**Figures 285–287.**
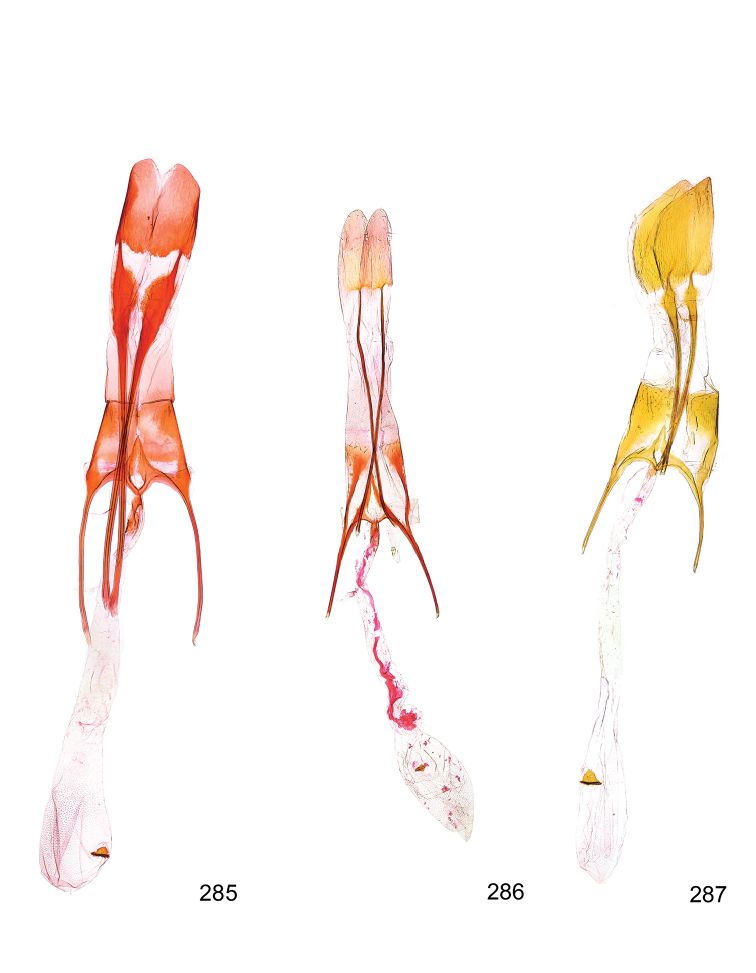
*Megacraspedus* female genitalia. **285***M.niphorrhoa* (Meyrick, 1926) – Russia, GU 17/1493 P.H. (RCKN) **286***M.albella* (Amsel, 1935) – Paratype, Iran, gen. slide 16.664 (NHMW) **287***M.balneariellus* (Chrétien, 1907) – Italy, GU 17/1476 P.H. (RCEA).

**Figures 288–290.**
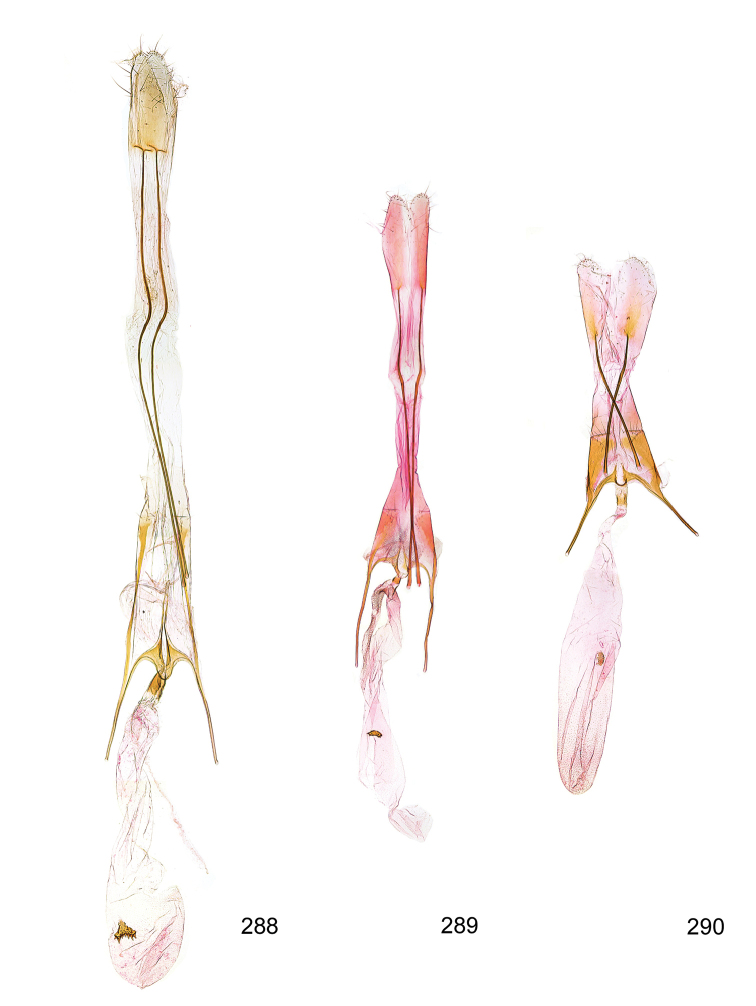
*Megacraspedus* female genitalia. **288***M.glaberipalpus* sp. n. – Paratype, Morocco, gen. slide 16.666 (NHMW) **289***M.imparellus* (Fischer von Röslerstamm, 1843) – Austria, GU 17/1479 P.H. (RCEA) **290***M.multispinella* Junnilainen & Nupponen, 2010 – Paratype, Russia, GU 16/1419 P.H. (MZH).

**Figures 291–293.**
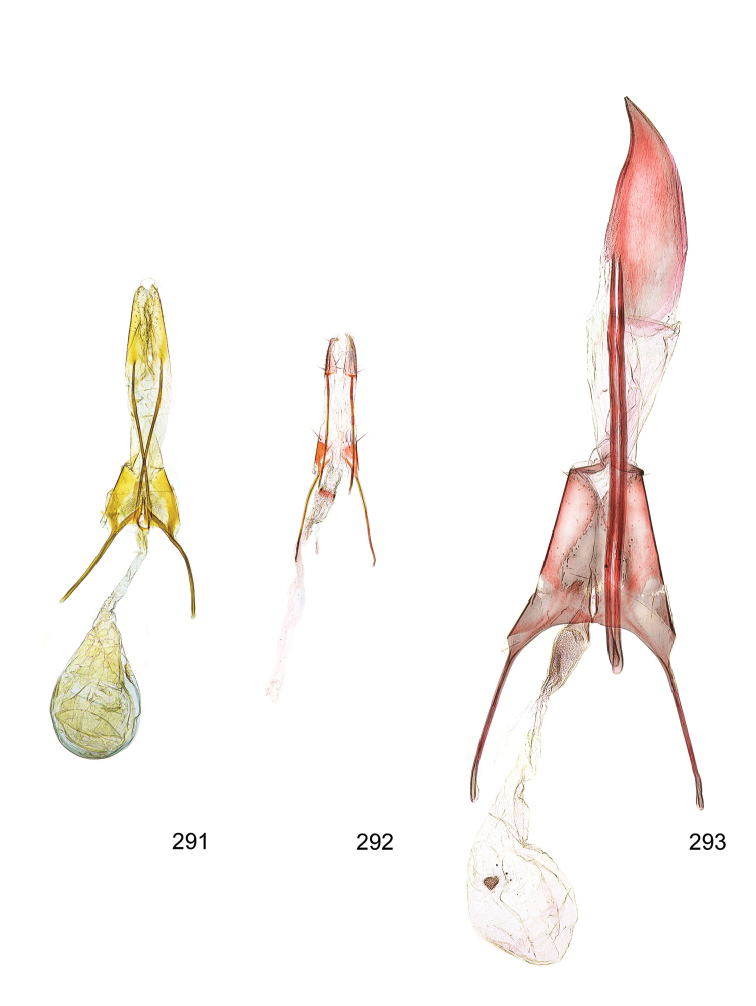
*Megacraspedus* female genitalia. **291***M.nupponeni* sp. n. – Paratype, Russia, gen. slide 4/16.7.2017 K. Nupponen (RCKN) **292***M.leuca* (Filipjev, 1929) – Paratype (*kaszabianus*), Mongolia, GU 16/1447 P.H. (HNHM) **293***M.lagopellus* Herrich-Schäffer, 1860 – Hungary, gen. slide 16.657 (NHMW).

**Figures 294–296.**
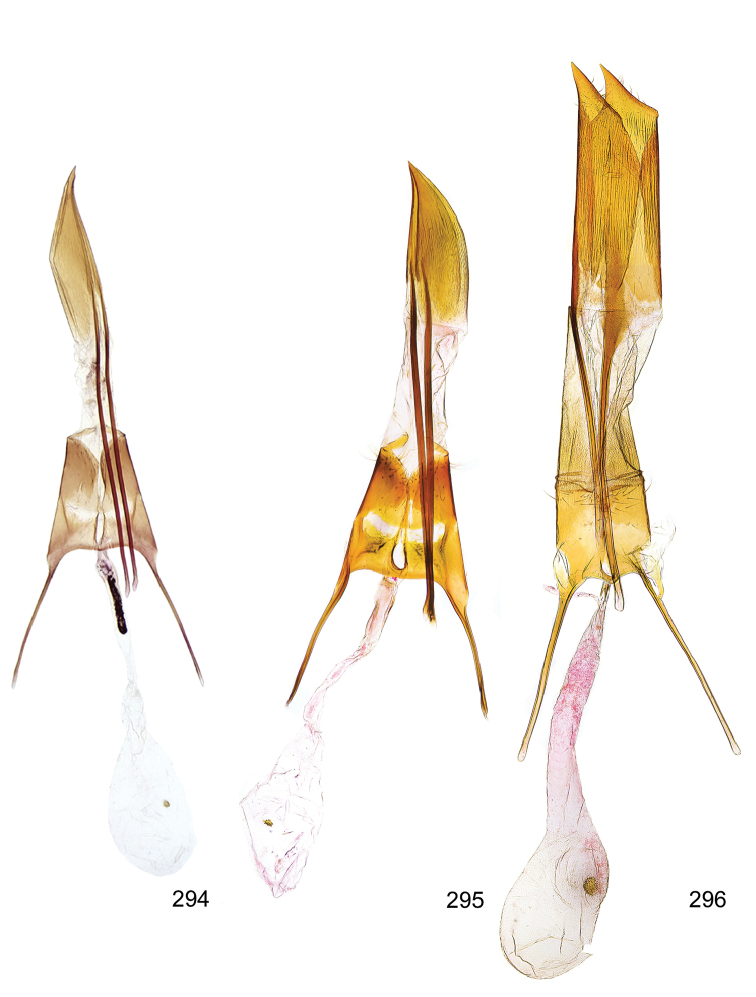
*Megacraspedus* female genitalia. **294***M.coleophorodes* (Li & Zheng, 1995) – Paratype, China, gen. slide L92040 (NKU) **295***M.feminensis* sp. n. – Paratype, Kazakhstan, GU 17/1495 P.H. (RCKN) **296***M.kirgizicus* sp. n. – Paratype, Kyrgyzstan, GU 17/1407 P.H. (LMK).

**Figures 297–299.**
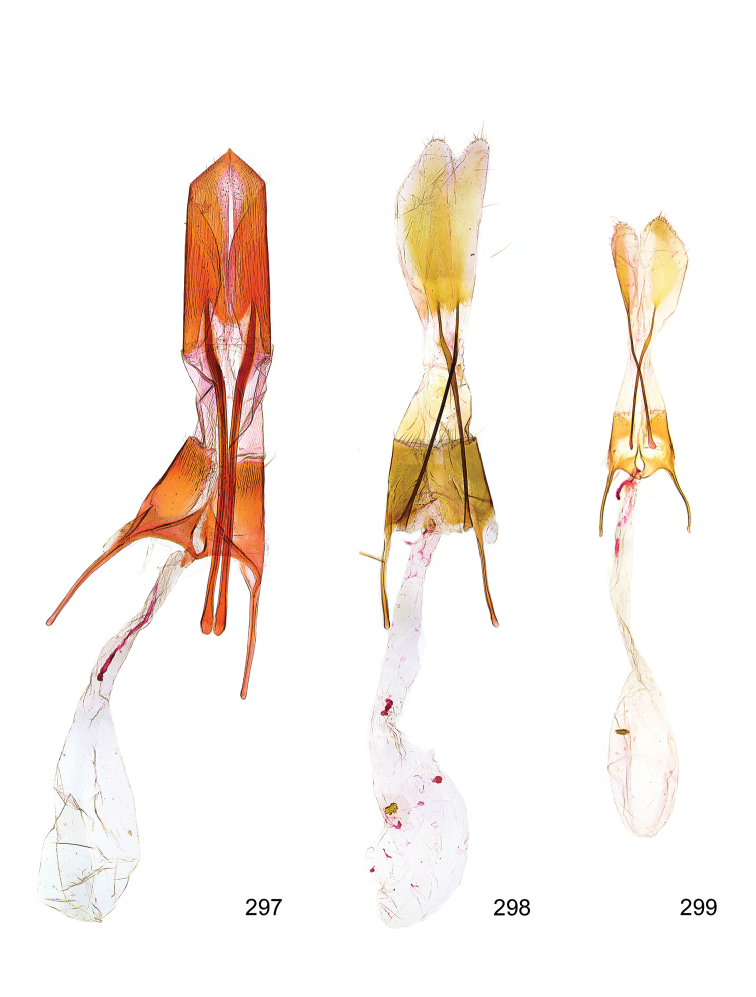
*Megacraspedus* female genitalia. **297***M.argyroneurellus* Staudinger, 1871 – Iran, gen. slide Mus. Vind. 16.659 (NHMW) **298***M.squalida* Meyrick, 1926 – Spain, GU 16/1428 P.H. (NMPC) **299***M.korabicus* sp. n. – Paratype, Macedonia, GEL 1273 P.H. (TLMF).

**Figures 300–302.**
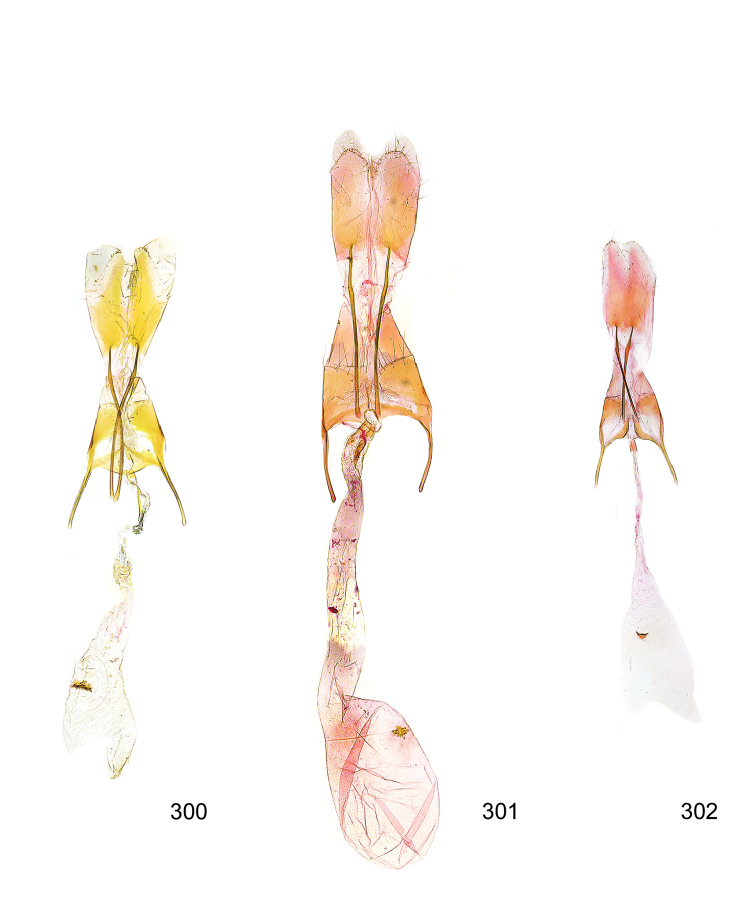
*Megacraspedus* female genitalia. **300***M.quadristictus* Lhomme, 1946 – Spain, GU 17/1486 P.H. (ZMUC) **301***M.eburnellus* Huemer & Karsholt, 2001 – Paratype, Italy, GEL 989 P.H. (TLMF) **302***M.skulei* sp. n. – Paratype, Spain, GU 16/1411 P.H. (NMPC).

**Figures 303–305.**
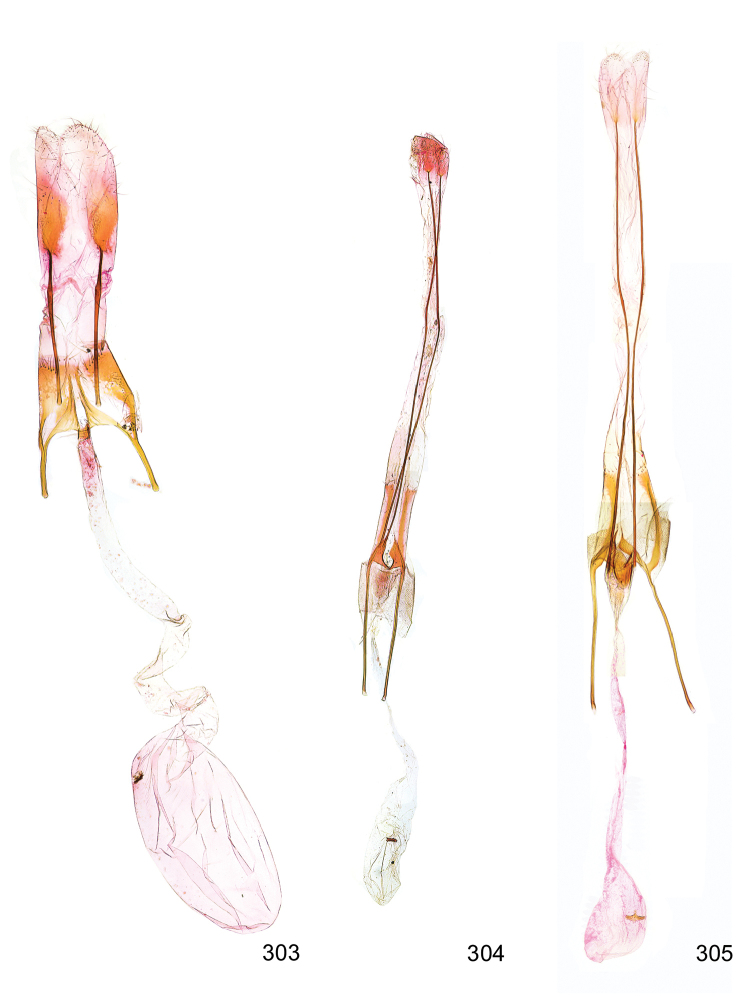
*Megacraspedus* female genitalia. **303***M.peyerimhoffi* Le Cerf, 1925 – Tunisia, GEL 1268 P.H. (TLMF) **304***M.pacificus* sp. n. – Paratype, Afghanistan, gen. slide 16.662 (NHMW) **305***M.armatophallus* sp. n. – Paratype, Afghanistan, GU 18/1503 P.H. (SMNK).

## Supplementary Material

XML Treatment for Megacraspedus lanceolellus

XML Treatment for Megacraspedus bengtssoni

XML Treatment for Megacraspedus homochroa

XML Treatment for Megacraspedus monolorellus

XML Treatment for Megacraspedus junnilaineni

XML Treatment for Megacraspedus uzunsyrtus

XML Treatment for Megacraspedus similellus

XML Treatment for Megacraspedus golestanicus

XML Treatment for Megacraspedus tokari

XML Treatment for Megacraspedus dolosellus

XML Treatment for Megacraspedus neli

XML Treatment for Megacraspedus faunierensis

XML Treatment for Megacraspedus gredosensis

XML Treatment for Megacraspedus cuencellus

XML Treatment for Megacraspedus bidentatus

XML Treatment for Megacraspedus fuscus

XML Treatment for Megacraspedus trineae

XML Treatment for Megacraspedus tristictus

XML Treatment for Megacraspedus alfacarellus

XML Treatment for Megacraspedus pusillus

XML Treatment for Megacraspedus skoui

XML Treatment for Megacraspedus spinophallus

XML Treatment for Megacraspedus occidentellus

XML Treatment for Megacraspedus granadensis

XML Treatment for Megacraspedus heckfordi

XML Treatment for Megacraspedus tenuiuncus

XML Treatment for Megacraspedus lativalvellus

XML Treatment for Megacraspedus dejectella

XML Treatment for Megacraspedus devorator

XML Treatment for Megacraspedus binotella

XML Treatment for Megacraspedus brachypteris

XML Treatment for Megacraspedus barcodiellus

XML Treatment for Megacraspedus bilineatella

XML Treatment for Megacraspedus andreneli

XML Treatment for Megacraspedus sumpichi

XML Treatment for Megacraspedus tabelli

XML Treatment for Megacraspedus gallicus

XML Treatment for Megacraspedus ribbeella

XML Treatment for Megacraspedus libycus

XML Treatment for Megacraspedus numidellus

XML Treatment for Megacraspedus albovenata

XML Treatment for Megacraspedus longipalpella

XML Treatment for Megacraspedus niphorrhoa

XML Treatment for Megacraspedus albella

XML Treatment for Megacraspedus fallax

XML Treatment for Megacraspedus balneariellus

XML Treatment for Megacraspedus podolicus

XML Treatment for Megacraspedus kazakhstanicus

XML Treatment for Megacraspedus knudlarseni

XML Treatment for Megacraspedus majorella

XML Treatment for Megacraspedus latiuncus

XML Treatment for Megacraspedus tenuignathos

XML Treatment for Megacraspedus glaberipalpus

XML Treatment for Megacraspedus imparellus

XML Treatment for Megacraspedus multispinella

XML Treatment for Megacraspedus nupponeni

XML Treatment for Megacraspedus cerussatellus

XML Treatment for Megacraspedus attritellus

XML Treatment for Megacraspedus consortiella

XML Treatment for Megacraspedus pototskii

XML Treatment for Megacraspedus leuca

XML Treatment for Megacraspedus orenburgensis

XML Treatment for Megacraspedus lagopellus

XML Treatment for Megacraspedus coleophorodes

XML Treatment for Megacraspedus feminensis

XML Treatment for Megacraspedus kirgizicus

XML Treatment for Megacraspedus argyroneurellus

XML Treatment for Megacraspedus ibericus

XML Treatment for Megacraspedus violacellum

XML Treatment for Megacraspedus squalida

XML Treatment for Megacraspedus pentheres

XML Treatment for Megacraspedus steineri

XML Treatment for Megacraspedus gibeauxi

XML Treatment for Megacraspedus multipunctellus

XML Treatment for Megacraspedus teriolensis

XML Treatment for Megacraspedus korabicus

XML Treatment for Megacraspedus quadristictus

XML Treatment for Megacraspedus eburnellus

XML Treatment for Megacraspedus skulei

XML Treatment for Megacraspedus longivalvellus

XML Treatment for Megacraspedus peyerimhoffi

XML Treatment for Megacraspedus peslieri

XML Treatment for Megacraspedus grisea

XML Treatment for Megacraspedus pacificus

XML Treatment for Megacraspedus armatophallus

## Supplementary materials

Supplementary material 1 Distribution catalogue Data type: occurrence This is an open access article distributed under the terms of the Creative Commons Attribution License (CC BY 4.0), which permits unrestricted use, distribution, and reproduction in any medium, provided the original author and source are credited.Peter Huemer, Ole Karsholt
