# Supplementary material for: Revision of the genus Megacraspedus Zeller, 1839, a challenging taxonomic tightrope of species delimitation (Lepidoptera, Gelechiidae)
Source: Zookeys. 2018 Nov 29;(800):1–278. doi: 10.3897/zookeys.800.26292 (PMC6284011; doi:10.3897/zookeys.800.26292)
Supplement: Supplementary material 1 — Distribution catalogue [file zookeys-800-001-s001.docx]

**Supplementary material 1**

**Distribution Catalogue**

Acronyms of countries are given below, except for some larger islands. Slovenia is listed as SL and not SI to avoid confusion with Si (Sicily).

Ci Canary Islands

MA Morocco

DZ Algeria

TN Tunisia

LY Libya

PT Portugal

ES Spain

AD Andorra

FR France

Co Corsica

Si Sicily

IT Italy

MT Malta

DE Germany

CH Switzerland

AT Austria

PL Poland

CZ Czech Republic

SK Slovakia

HU Hungary

RO Romania

SL Slovenia

HR Croatia

ME Montenegro

BA Bosnia-Herzegovina

MK Macedonia

AL Albania

BG Bulgaria

GR Greece

Cr Crete

UA Ukraine

RU Russia

TR Turkey

AM Armenia

IL Israel

IR Iran

TM Turkmenistan

AF Afghanistan

KZ Kazakhstan

KG Kyrgyzstan

MN Mongolia

CN China

NP Nepal

x = proved record

L = Literature record

|  | **Ci** | **MA** | **DZ** | **TN** | **LY** | **PT** | **ES** | **AD** | **FR** | **Co** | **Si** | **IT** | **MT** | **DE** | **CH** | **AU** | **PL** | **CZ** | **SK** | **HU** | **RO** | **SL** |
| --- | --- | --- | --- | --- | --- | --- | --- | --- | --- | --- | --- | --- | --- | --- | --- | --- | --- | --- | --- | --- | --- | --- |
| *M. lanceolellus* |  |  |  |  |  |  | x | x | x | L | L | x |  | L |  |  |  |  |  |  |  |  |
| *M. bengtssoni* |  |  |  |  |  |  | x |  |  |  |  |  |  |  |  |  |  |  |  |  |  |  |
| *M. homochroa* |  | x |  |  |  |  |  |  |  |  |  |  |  |  |  |  |  |  |  |  |  |  |
| *M. monolorellus* |  |  |  |  |  |  |  |  |  |  |  |  |  |  |  |  |  |  |  |  |  |  |
| *M. junnilaineni* |  |  |  |  |  |  |  |  |  |  |  |  |  |  |  |  |  |  |  |  |  |  |
| *M. uzunsyrtus* |  |  |  |  |  |  |  |  |  |  |  |  |  |  |  |  |  |  |  |  |  |  |
| *M. similellus* |  |  |  |  |  |  |  |  |  |  |  |  |  |  |  |  |  |  |  |  | x |  |
| *M. golestanicus* |  |  |  |  |  |  |  |  |  |  |  |  |  |  |  |  |  |  |  |  |  |  |
| *M. tokari* |  |  |  |  |  |  |  |  |  |  |  |  |  |  |  |  |  |  |  |  |  |  |
| *M. dolosellus* |  |  |  |  |  |  |  |  | x |  | L | x |  |  |  | x |  |  | x | x | x |  |
| *M. neli* |  |  |  |  |  |  |  |  | x |  |  | x |  |  |  |  |  |  |  |  |  |  |
| *M. faunierensis* |  |  |  |  |  |  |  |  |  |  |  | x |  |  |  |  |  |  |  |  |  |  |
| *M. gredosensis* |  |  |  |  |  |  | x |  |  |  |  |  |  |  |  |  |  |  |  |  |  |  |
| *M. cuencellus* |  |  |  |  |  |  | x |  | x |  |  |  |  |  |  |  |  |  |  |  |  |  |
| *M. bidentatus* |  |  |  |  |  |  | x |  |  |  |  |  |  |  |  |  |  |  |  |  |  |  |
| *M. fuscus* |  |  |  |  |  |  | x |  |  |  |  |  |  |  |  |  |  |  |  |  |  |  |
| *M. trineae* |  |  |  |  |  | x | x |  |  |  |  |  |  |  |  |  |  |  |  |  |  |  |
| *M. tristictus* |  |  |  |  |  |  |  |  | x |  |  | x |  |  |  |  |  |  |  |  |  |  |
| *M. alfacarellus* |  |  |  |  |  |  | x |  |  |  |  |  |  |  |  |  |  |  |  |  |  |  |
| *M. pusillus* |  |  |  |  |  |  | x |  |  |  |  |  |  |  |  |  |  |  |  |  |  |  |
| *M. skoui* |  |  |  |  |  |  | x |  |  |  |  |  |  |  |  |  |  |  |  |  |  |  |
| *M. spinophallus* |  |  |  |  |  |  | x |  |  |  |  |  |  |  |  |  |  |  |  |  |  |  |
| *M. occidentellus* |  |  |  |  |  | x |  |  |  |  |  |  |  |  |  |  |  |  |  |  |  |  |
| *M. granadensis* |  |  |  |  |  |  | x |  |  |  |  |  |  |  |  |  |  |  |  |  |  |  |
| *M. heckfordi* |  |  |  |  |  |  | x |  |  |  |  |  |  |  |  |  |  |  |  |  |  |  |
| *M. tenuiuncus* |  |  |  |  |  |  | x |  | x |  |  |  |  |  |  |  |  |  |  |  |  |  |
| *M. lativalvellus* |  |  |  |  |  |  | x |  |  |  |  |  | x |  |  |  |  |  |  |  |  |  |
| *M. dejectella* |  |  |  |  |  |  | X |  |  |  |  |  |  |  |  |  |  |  |  |  |  |  |
| *M. devorator* |  |  |  |  |  |  |  |  |  |  |  |  |  |  |  |  |  |  |  |  | x |  |
| *M. binotella* |  |  |  |  |  |  |  |  |  |  |  | x |  | x | x | x | x |  | x | x | x | x |
| *M. brachypteris* |  |  |  |  |  |  |  |  |  |  |  |  |  |  |  |  |  |  |  |  |  |  |
| *M. barcodiellus* |  |  |  |  |  |  |  |  |  |  |  |  |  |  |  |  |  |  |  |  |  |  |
| *M. bilineatella* |  |  |  |  |  |  |  |  |  |  |  | x |  |  |  |  |  |  |  |  |  |  |
| *M. andreneli* |  |  |  |  |  |  |  |  | x |  |  |  |  |  |  |  |  |  |  |  |  |  |
| *M. sumpichi* |  |  |  |  |  |  | x |  |  |  |  |  |  |  |  |  |  |  |  |  |  |  |
| *M. tabelli* |  | x |  |  |  |  |  |  |  |  |  |  |  |  |  |  |  |  |  |  |  |  |
| *M. gallicus* |  |  |  |  |  |  | x |  | x |  |  |  |  |  |  |  |  |  |  |  |  |  |
| *M. ribbeella* |  |  |  |  |  |  | x |  |  |  |  |  |  |  |  |  |  |  |  |  |  |  |
| *M. libycus* |  | x |  |  | x |  |  |  |  |  |  |  |  |  |  |  |  |  |  |  |  |  |
| *M. numidellus* | x | x |  | x | x |  |  |  |  |  |  |  |  |  |  |  |  |  |  |  |  |  |
| *M. albovenata* |  |  |  |  |  |  |  |  |  |  |  |  |  |  |  |  |  | x |  |  |  |  |
| *M. longipalpella* |  |  |  |  |  |  |  |  |  |  |  |  |  |  |  |  |  |  |  |  |  |  |
| *M. niphorrhoa* |  |  |  |  |  |  |  |  |  |  |  |  |  |  |  |  |  |  |  |  |  |  |
| *M. albella* |  |  |  |  |  |  |  |  |  |  |  |  |  |  |  |  |  |  |  |  |  |  |
| *M. fallax* |  |  |  |  |  |  |  |  |  |  |  |  |  |  |  |  |  |  |  | x |  |  |
| *M. balneariellus* |  |  |  |  |  |  | x |  | x |  | x |  |  |  |  |  |  |  |  |  |  |  |
| *M. podolicus* |  |  |  |  |  |  |  |  |  |  |  |  |  |  |  | x |  |  |  | x | x |  |
| *M. kazakhstanicus* |  |  |  |  |  |  |  |  |  |  |  |  |  |  |  |  |  |  |  |  |  |  |
| *M. knudlarseni* | x |  |  |  |  |  |  |  |  |  |  |  |  |  |  |  |  |  |  |  |  |  |
| *M. majorella* |  |  |  |  |  |  |  |  |  |  |  |  |  |  |  |  |  |  |  |  |  |  |
| *M. latiuncus* |  |  |  |  |  |  |  |  |  |  |  |  |  |  |  |  |  |  |  |  |  |  |
| *M. tenuignathos* |  | x |  |  |  |  |  |  |  |  |  |  |  |  |  |  |  |  |  |  |  |  |
| *M. glaberipalpus* |  | x |  |  |  |  |  |  |  |  |  |  |  |  |  |  |  |  |  |  |  |  |
| *M. imparellus* |  |  |  |  |  |  |  |  |  |  |  | L |  |  |  | x |  |  | x | x | x |  |
| *M. multispinella* |  |  |  |  |  |  |  |  |  |  |  |  |  |  |  |  |  |  |  |  |  |  |
| *M. nupponeni* |  |  |  |  |  |  |  |  |  |  |  |  |  |  |  |  |  |  |  |  |  |  |
| *M. cerussatellus* |  |  |  |  |  |  |  |  |  |  |  |  |  |  |  |  |  |  |  |  |  |  |
| *M. attritellus* |  |  |  |  |  |  |  |  |  |  |  |  |  |  |  |  |  |  |  |  |  |  |
| *M. consortiella* |  |  |  |  |  |  |  |  |  |  |  |  |  |  |  |  |  |  |  |  |  |  |
| *M. pototskii* |  |  |  |  |  |  |  |  |  |  |  |  |  |  |  |  |  |  |  |  |  |  |
| *M. leuca* |  |  |  |  |  |  |  |  |  |  |  |  |  |  |  |  |  |  |  |  |  |  |
| *M. orenburgensis* |  |  |  |  |  |  |  |  |  |  |  |  |  |  |  |  |  |  |  |  |  |  |
| *M. lagopellus* |  |  |  |  |  |  |  |  |  |  |  |  |  |  |  |  |  |  |  | x |  |  |
| *M. coleophorodes* |  |  |  |  |  |  |  |  |  |  |  |  |  |  |  |  |  |  |  |  |  |  |
| *M. feminensis* |  |  |  |  |  |  |  |  |  |  |  |  |  |  |  |  |  |  |  |  |  |  |
| *M. kirgizicus* |  |  |  |  |  |  |  |  |  |  |  |  |  |  |  |  |  |  |  |  |  |  |
| *M. argyroneurellus* |  |  |  |  |  |  |  |  |  |  |  |  |  |  |  |  |  |  |  | x |  |  |
| *M. ibericus* |  |  |  |  |  | x | x |  |  |  |  |  |  |  |  |  |  |  |  |  |  |  |
| *M. violacellum* |  |  |  | x | x |  |  |  |  |  |  |  |  |  |  |  |  |  |  |  |  |  |
| *M. squalida* |  |  |  | x |  |  | x |  |  |  |  |  |  |  |  |  |  |  |  |  |  |  |
| *M. pentheres* |  |  |  |  |  |  |  |  | x |  |  |  |  |  |  |  |  |  |  |  |  |  |
| *M. steineri* |  | x |  |  |  |  |  |  |  |  |  |  |  |  |  |  |  |  |  |  |  |  |
| *M. gibeauxi* |  |  | x | x |  |  |  |  |  |  |  |  |  |  |  |  |  |  |  |  |  |  |
| *M. multipunctellus* |  |  |  |  |  |  |  |  |  |  |  |  |  |  |  |  |  |  |  |  |  |  |
| *M. teriolensis* |  |  |  |  |  |  |  |  |  |  |  | x |  |  |  |  |  |  |  |  |  | x |
| *M. korabicus* |  |  |  |  |  |  |  |  |  |  |  |  |  |  |  |  |  |  |  |  |  |  |
| *M. quadristictus* |  |  |  |  |  |  | x |  | x |  |  |  |  |  |  |  |  |  |  |  |  |  |
| *M. eburnellus* |  |  |  |  |  |  |  |  |  |  |  | x |  |  |  |  |  |  |  |  |  |  |
| *M. skulei* |  |  |  |  |  |  | x |  |  |  |  |  |  |  |  |  |  |  |  |  |  |  |
| *M. longivalvellus* |  | x |  |  |  |  |  |  |  |  |  |  |  |  |  |  |  |  |  |  |  |  |
| *M. peyerimhoffi* |  |  | x |  |  |  | x |  |  |  |  |  |  |  |  |  |  |  |  |  |  |  |
| *M. peslieri* |  |  |  |  |  |  | x |  | x |  |  |  |  |  |  |  |  |  |  |  |  |  |
| *M. grisea* |  |  |  |  |  |  |  |  |  |  |  |  |  |  |  |  |  |  |  |  |  |  |
| *M. pacificus* |  |  |  |  |  |  |  |  |  |  |  |  |  |  |  |  |  |  |  |  |  |  |
| *M. armatophallus* |  |  |  |  |  |  |  |  |  |  |  |  |  |  |  |  |  |  |  |  |  |  |

|  | **HR** | **ME** | **BA** | **MK** | **AL** | **BG** | **GR** | **Cr** | **UA** | **RU** | **TR** | **AM** | **IL** | **IR** | **TM** | **AF** | **KZ** | **KG** | **MN** | **CN** | **NP** |
| --- | --- | --- | --- | --- | --- | --- | --- | --- | --- | --- | --- | --- | --- | --- | --- | --- | --- | --- | --- | --- | --- |
| *M. lanceolellus* | x | x |  |  |  |  |  |  |  |  |  |  |  |  |  |  |  |  |  |  |  |
| *M. bengtssoni* |  |  |  |  |  |  |  |  |  |  |  |  |  |  |  |  |  |  |  |  |  |
| *M. homochroa* |  |  |  |  |  |  |  |  |  |  |  |  |  |  |  |  |  |  |  |  |  |
| *M. monolorellus* |  |  |  |  |  |  |  |  |  |  | x |  |  |  |  |  |  |  |  |  |  |
| *M. junnilaineni* |  |  |  |  |  |  |  |  |  |  | x |  |  |  |  |  |  |  |  |  |  |
| *M. uzunsyrtus* |  |  |  |  |  |  |  |  | x |  |  |  |  |  |  |  |  |  |  |  |  |
| *M. similellus* |  |  |  |  |  | x |  |  |  |  | x |  |  |  |  |  |  |  |  |  |  |
| *M. golestanicus* |  |  |  |  |  |  |  |  |  |  |  |  |  | x |  |  |  |  |  |  |  |
| *M. tokari* | x |  |  |  |  |  |  |  |  |  |  |  |  |  |  |  |  |  |  |  |  |
| *M. dolosellus* |  | x | x | x | x | x | x | x | x | x | x | L |  |  |  |  |  |  |  |  | x |
| *M. neli* |  |  |  |  |  |  |  |  |  |  |  |  |  |  |  |  |  |  |  |  |  |
| *M. faunierensis* |  |  |  |  |  |  |  |  |  |  |  |  |  |  |  |  |  |  |  |  |  |
| *M. gredosensis* |  |  |  |  |  |  |  |  |  |  |  |  |  |  |  |  |  |  |  |  |  |
| *M. cuencellus* |  |  |  |  |  |  |  |  |  |  |  |  |  |  |  |  |  |  |  |  |  |
| *M. bidentatus* |  |  |  |  |  |  |  |  |  |  |  |  |  |  |  |  |  |  |  |  |  |
| *M. fuscus* |  |  |  |  |  |  |  |  |  |  |  |  |  |  |  |  |  |  |  |  |  |
| *M. trineae* |  |  |  |  |  |  |  |  |  |  |  |  |  |  |  |  |  |  |  |  |  |
| *M. tristictus* |  |  |  |  |  |  |  |  |  |  |  |  |  |  |  |  |  |  |  |  |  |
| *M. alfacarellus* |  |  |  |  |  |  |  |  |  |  |  |  |  |  |  |  |  |  |  |  |  |
| *M. pusillus* |  |  |  |  |  |  |  |  |  |  |  |  |  |  |  |  |  |  |  |  |  |
| *M. skoui* |  |  |  |  |  |  |  |  |  |  |  |  |  |  |  |  |  |  |  |  |  |
| *M. spinophallus* |  |  |  |  |  |  |  |  |  |  |  |  |  |  |  |  |  |  |  |  |  |
| *M. occidentellus* |  |  |  |  |  |  |  |  |  |  |  |  |  |  |  |  |  |  |  |  |  |
| *M. granadensis* |  |  |  |  |  |  |  |  |  |  |  |  |  |  |  |  |  |  |  |  |  |
| *M. heckfordi* |  |  |  |  |  |  |  |  |  |  |  |  |  |  |  |  |  |  |  |  |  |
| *M. tenuiuncus* |  |  |  |  |  |  |  |  |  |  |  |  |  |  |  |  |  |  |  |  |  |
| *M. lativalvellus* |  |  |  |  |  |  |  |  |  |  |  |  |  |  |  |  |  |  |  |  |  |
| *M. dejectella* |  |  |  |  |  |  |  |  |  |  |  |  |  |  |  |  |  |  |  |  |  |
| *M. devorator* |  |  |  |  |  | x |  |  |  |  |  |  |  |  |  |  |  |  |  |  |  |
| *M. binotella* |  |  |  |  |  |  |  |  |  |  |  |  |  |  |  |  |  |  |  |  |  |
| *M. brachypteris* |  | x |  | x | x |  | x |  |  |  |  |  |  |  |  |  |  |  |  |  |  |
| *M. barcodiellus* |  |  |  | x |  |  |  |  |  |  |  |  |  |  |  |  |  |  |  |  |  |
| *M. bilineatella* |  |  |  |  |  |  |  |  |  |  |  |  |  |  |  |  |  |  |  |  |  |
| *M. andreneli* |  |  |  |  |  |  |  |  |  |  |  |  |  |  |  |  |  |  |  |  |  |
| *M. sumpichi* |  |  |  |  |  |  |  |  |  |  |  |  |  |  |  |  |  |  |  |  |  |
| *M. tabelli* |  |  |  |  |  |  |  |  |  |  |  |  |  |  |  |  |  |  |  |  |  |
| *M. gallicus* |  |  |  |  |  |  |  |  |  |  |  |  |  |  |  |  |  |  |  |  |  |
| *M. ribbeella* |  |  |  |  |  |  |  |  |  |  |  |  |  |  |  |  |  |  |  |  |  |
| *M. libycus* |  |  |  |  |  |  |  |  |  |  |  |  |  |  |  |  |  |  |  |  |  |
| *M. numidellus* |  |  |  |  |  |  |  |  |  |  |  |  |  |  |  |  |  |  |  |  |  |
| *M. albovenata* |  |  |  |  |  |  |  |  |  | x |  |  |  |  |  |  |  |  |  |  |  |
| *M. longipalpella* |  |  |  |  |  |  |  |  | x | x |  |  |  |  |  |  |  |  |  |  |  |
| *M. niphorrhoa* |  |  |  |  |  |  |  |  |  | x |  |  |  |  |  |  | x |  |  |  |  |
| *M. albella* |  |  |  |  |  |  |  |  |  |  |  |  | x | x |  |  |  |  |  |  |  |
| *M. fallax* |  |  |  |  |  |  |  |  |  | x | x |  |  |  |  |  | x |  |  | ? |  |
| *M. balneariellus* | x |  |  |  |  |  |  |  |  |  |  |  |  |  |  |  |  |  |  |  |  |
| *M. podolicus* |  |  |  |  |  |  |  |  | x | x |  |  |  |  |  |  |  |  |  |  |  |
| *M. kazakhstanicus* |  |  |  |  |  |  |  |  |  |  |  | x |  |  |  |  | x |  |  |  |  |
| *M. knudlarseni* |  |  |  |  |  |  |  |  |  |  |  |  |  |  |  |  |  |  |  |  |  |
| *M. majorella* |  |  |  |  |  |  |  |  |  |  |  |  |  |  |  |  |  | x |  |  |  |
| *M. latiuncus* |  |  |  |  |  |  |  |  |  |  |  |  |  |  |  |  | x |  |  |  |  |
| *M. tenuignathos* |  |  |  |  |  |  |  |  |  |  |  |  |  |  |  |  |  |  |  |  |  |
| *M. glaberipalpus* |  |  |  |  |  |  |  |  |  |  |  |  |  |  |  |  |  |  |  |  |  |
| *M. imparellus* |  |  |  |  |  | x | x |  |  | x |  |  |  |  |  |  |  |  |  |  |  |
| *M. multispinella* |  |  |  |  |  |  |  |  |  | x |  |  |  |  |  |  |  |  |  |  |  |
| *M. nupponeni* |  |  |  |  |  |  |  |  |  | x |  |  |  |  |  |  |  |  |  |  |  |
| *M. cerussatellus* |  |  |  |  |  | x |  |  |  |  |  |  |  |  |  |  |  |  |  |  |  |
| *M. attritellus* |  |  |  |  |  |  |  |  |  | x |  |  |  |  |  |  |  |  |  |  |  |
| *M. consortiella* |  |  |  |  |  |  |  |  |  |  |  |  |  |  |  |  |  | x |  |  |  |
| *M. pototskii* |  |  |  |  |  |  |  |  |  |  |  |  |  |  |  |  |  | x |  |  |  |
| *M. leuca* |  |  |  |  |  |  |  |  |  | x |  |  |  |  |  |  |  |  | x |  |  |
| *M. orenburgensis* |  |  |  |  |  |  |  |  |  | x |  |  |  |  |  |  |  |  |  |  |  |
| *M. lagopellus* |  |  |  |  |  |  |  |  |  | x |  |  |  |  |  |  | x |  |  |  |  |
| *M. coleophorodes* |  |  |  |  |  |  |  |  |  |  |  |  |  |  |  |  |  |  |  | x |  |
| *M. feminensis* |  |  |  |  |  |  |  |  |  |  |  |  |  |  |  |  | x |  |  |  |  |
| *M. kirgizicus* |  |  |  |  |  |  |  |  |  |  |  | x |  |  |  | x | x | x |  |  |  |
| *M. argyroneurellus* |  |  |  |  |  |  |  |  |  | x | x | x |  | x |  |  | x |  |  |  |  |
| *M. ibericus* |  |  |  |  |  |  |  |  |  |  |  |  |  |  |  |  |  |  |  |  |  |
| *M. violacellum* |  |  |  |  |  |  |  |  |  |  |  |  |  |  |  |  |  |  |  |  |  |
| *M. squalida* |  |  |  |  |  |  |  |  |  |  |  |  |  |  |  |  |  |  |  |  |  |
| *M. pentheres* |  |  |  |  |  |  |  |  |  |  |  |  |  |  |  |  |  |  |  |  |  |
| *M. steineri* |  |  |  |  |  |  |  |  |  |  |  |  |  |  |  |  |  |  |  |  |  |
| *M. gibeauxi* |  |  |  |  |  |  |  |  |  |  |  |  |  |  |  |  |  |  |  |  |  |
| *M. multipunctellus* |  |  |  |  |  |  |  |  |  |  | x |  |  |  |  |  |  |  |  |  |  |
| *M. teriolensis* | x |  |  |  |  |  | x |  |  |  |  |  |  |  |  |  |  |  |  |  |  |
| *M. korabicus* |  |  |  | x |  |  |  |  |  |  |  |  |  |  |  |  |  |  |  |  |  |
| *M. quadristictus* |  |  |  |  |  |  |  |  |  |  |  |  |  |  |  |  |  |  |  |  |  |
| *M. eburnellus* |  |  |  |  |  |  |  |  |  |  |  |  |  |  |  |  |  |  |  |  |  |
| *M. skulei* |  |  |  |  |  |  |  |  |  |  |  |  |  |  |  |  |  |  |  |  |  |
| *M. longivalvellus* |  |  |  |  |  |  |  |  |  |  |  |  |  |  |  |  |  |  |  |  |  |
| *M. peyerimhoffi* |  |  |  |  |  |  |  |  |  |  |  |  |  |  |  |  |  |  |  |  |  |
| *M. peslieri* |  |  |  |  |  |  |  |  |  |  |  |  |  |  |  |  |  |  |  |  |  |
| *M. grisea* |  |  |  |  |  |  |  |  |  |  |  |  |  |  |  |  |  |  |  | x |  |
| *M. pacificus* |  |  |  |  |  |  |  |  |  |  |  |  |  |  |  | x |  |  |  |  |  |
| *M. armatophallus* |  |  |  |  |  |  |  |  |  |  |  |  |  |  |  | x |  |  |  |  |  |
